# Supplementary material for: Deubiquitination of RIPK3 by OTUB2 potentiates neuronal necroptosis after ischemic stroke
Source: EMBO Mol Med. 2025 Feb 28;17(4):679–95. doi: 10.1038/s44321-025-00206-6 (PMC11982199; doi:10.1038/s44321-025-00206-6)
Supplement: Supplementary file 1 — Appendix [file 44321_2025_206_MOESM1_ESM.pdf]

# Appendix for

# Deubiquitination of RIPK3 by OTUB2 potentiates neuronal necroptosis after ischemic stroke

## Table of contents


Table of contents ........................................................ 1
Appendix Figure S1 ........................................................ 3
Appendix Figure S2 ........................................................ 5
Appendix Figure S3 ........................................................ 6
Appendix Figure S4 ........................................................ 7
Appendix Figure S5 ........................................................ 9
Appendix Figure S6 ........................................................ 10
Appendix Figure S7 ........................................................ 11
Appendix Figure S8 ........................................................ 12
Appendix Figure S9 ........................................................ 13
Appendix Figure S10 ........................................................ 14
Appendix Figure S11 ........................................................ 15
Appendix Figure S12 ........................................................ 17
Appendix Figure S13. ........................................................ 18
Appendix Figure S14 ........................................................ 20
Appendix Figure S15 ........................................................ 21
Appendix Table S1 Primers for quantitative Real-Time PCR (qRT-PCR)22

Statistics Tables ........ 24
Appendix Table S2 ........ 24
Appendix Table S3 ........ 25
Appendix Table S4 ........ 25
Appendix Table S5 ........ 26
Appendix Table S6 ........ 26
Appendix Table S7 ........ 26
Appendix Table S8. ........ 27
Appendix Table S9 ........ 27
Appendix Table S10 ........ 27
Appendix Table S11 ........ 28
Appendix Table S12 ........ 28
Appendix Table S13 ........ 28
Appendix Table S14 ........ 29
Appendix Table S15 ........ 29
Appendix Table S16 ........ 29
Appendix Table S17 ........ 29

**Appendix Figure S1**

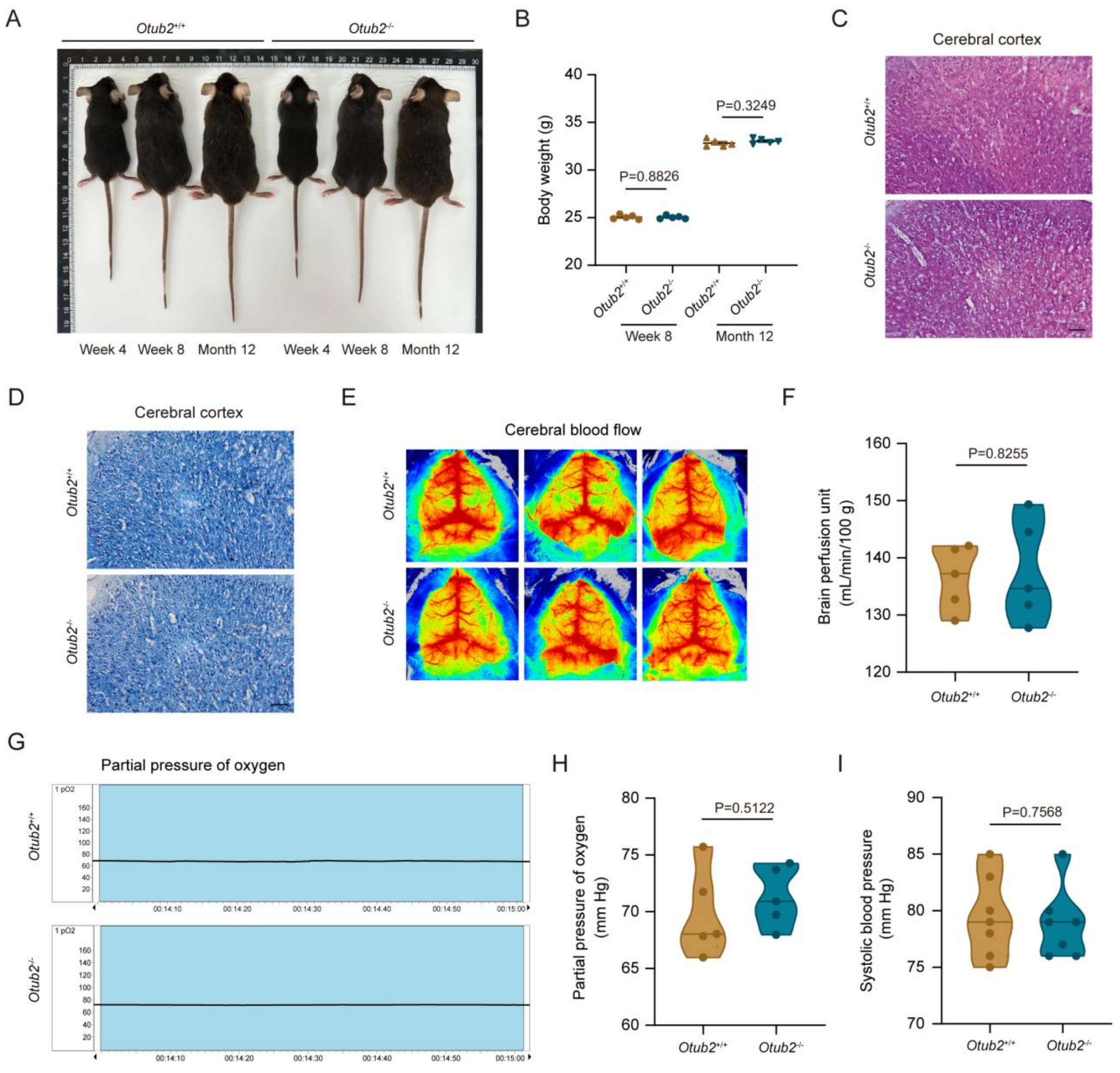


**Appendix Figure S1. OTUB2 deficiency does not affect the growth and development of mice**

(A) A representative image of *Otub2*$^{+/+}$ and *Otub2*$^{-/-}$ mice at indicated time after birth. (B) Body weight of male *Otub2*$^{+/+}$ and *Otub2*$^{-/-}$ mice at indicated time after birth. Multiple Student's t tests, n = 5 mice/group, biological replicates.(C-D) The cerebral cortex of 8-week-old male *Otub2*$^{+/+}$ and *Otub2*$^{-/-}$ mice was examined by H&E (C) and Nissl (D) staining. Scale bar, 100 μm.(E-F) Representative images (E) and statistics (F)

of the cerebral blood flow in 8-week-old male *Otub2*$^{+/+}$ and *Otub2*$^{-/-}$ mice. Unpaired Student's t test, n = 5 mice/group, biological replicates.(G-H) Representative images (G) and statistics (H) of the partial pressure of oxygen in 8-week-old male *Otub2*$^{+/+}$ and *Otub2*$^{-/-}$ mice. Unpaired Student's t test, n = 5 mice/group, biological replicates.(I) Systolic blood pressure of 8-week-old male *Otub2*$^{+/+}$ and *Otub2*$^{-/-}$ mice. Unpaired Student's t test, n = 5 mice/group, biological replicates. Data in (B) show the mean ± SEM. Source data are available online for this figure

**Appendix Figure S2**

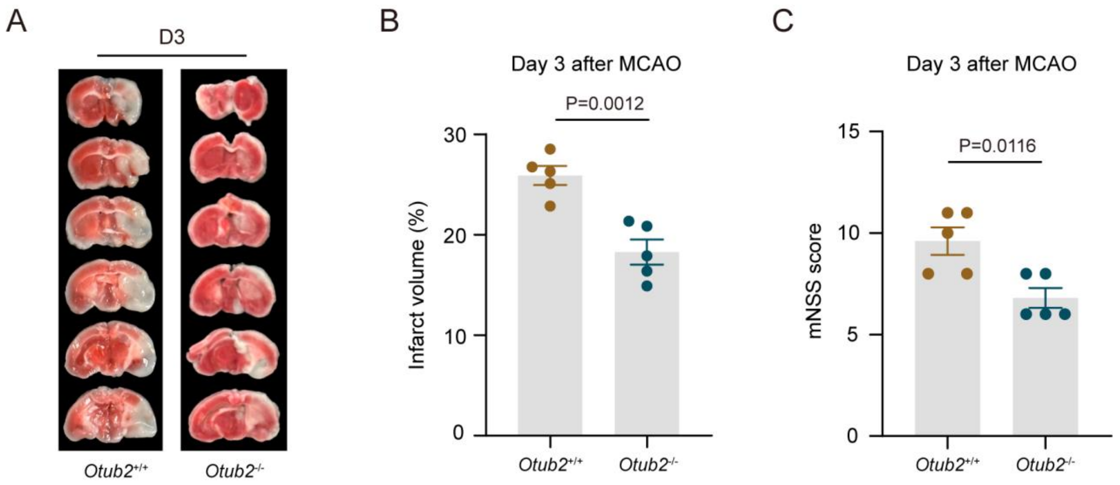


**Appendix Figure S2. OTUB2 deficiency attenuates MCAO-induced cerebral injury in aged mice**

(A) MCAO was induced in 12-month-old male *Otub2*$^{+/+}$ and *Otub2*$^{-/-}$ mice. On day 3 after MCAO, cerebral infarct size was analyzed by TTC staining. (B) Cerebral infarct volume was calculated based on TTC staining. Unpaired Student's t test, n = 5 mice/group, biological replicates. (C) Neurological function was evaluated by mNSS test. Mann-Whitney U test, n = 5 mice/group, biological replicates. Data in (B, C) show the mean ± SEM. Source data are available online for this figure.

## Appendix Figure S3

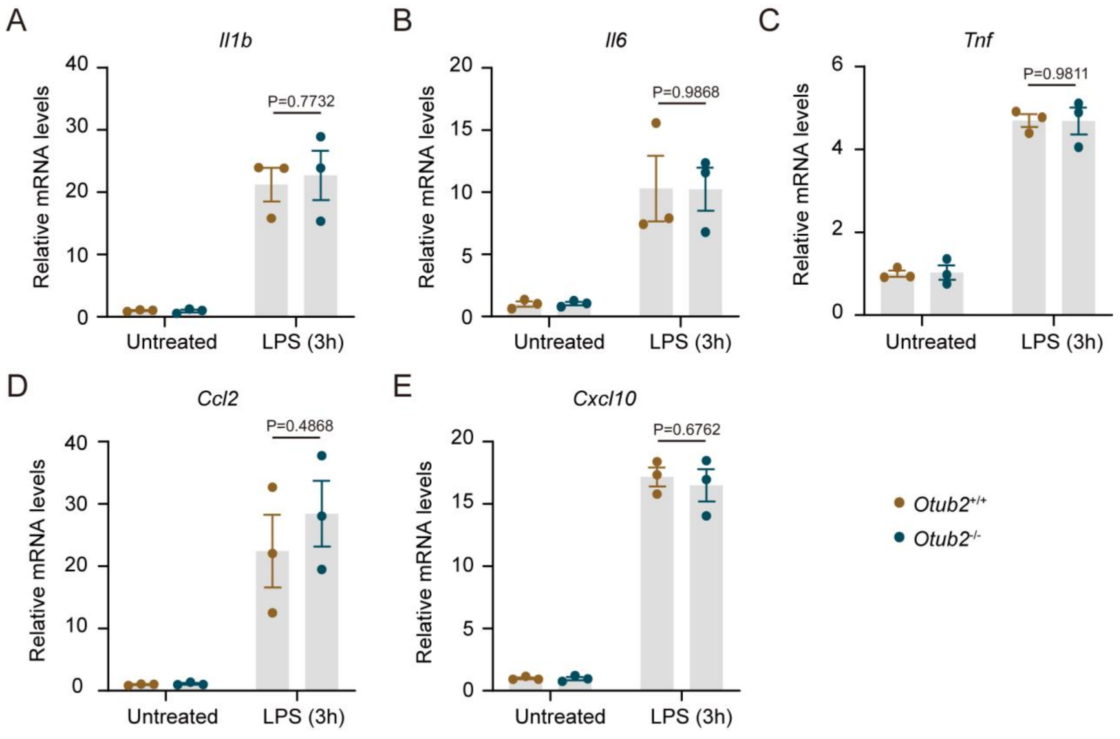


**Appendix Figure S3. OTUB2 deficiency does not affect the production of pro-inflammatory cytokines in microglia**

(A-E) Primary microglia from *Otub2*$^{+/+}$ and *Otub2*$^{-/-}$ mice were stimulated with LPS (500 ng/ml) for 3 h or left untreated. The transcription of *Il1b* (A), *Il6* (B), *Tnf* (C), *Ccl2* (D), and *Cxcl10* (E) was measured by qRT-PCR. Mann-Whitney U test (A) and unpaired Student's t test (B-E), n = 3/group, biological replicates. Data in (A-E) show the mean ± SEM. Source data are available online for this figure.

## Appendix Figure S4

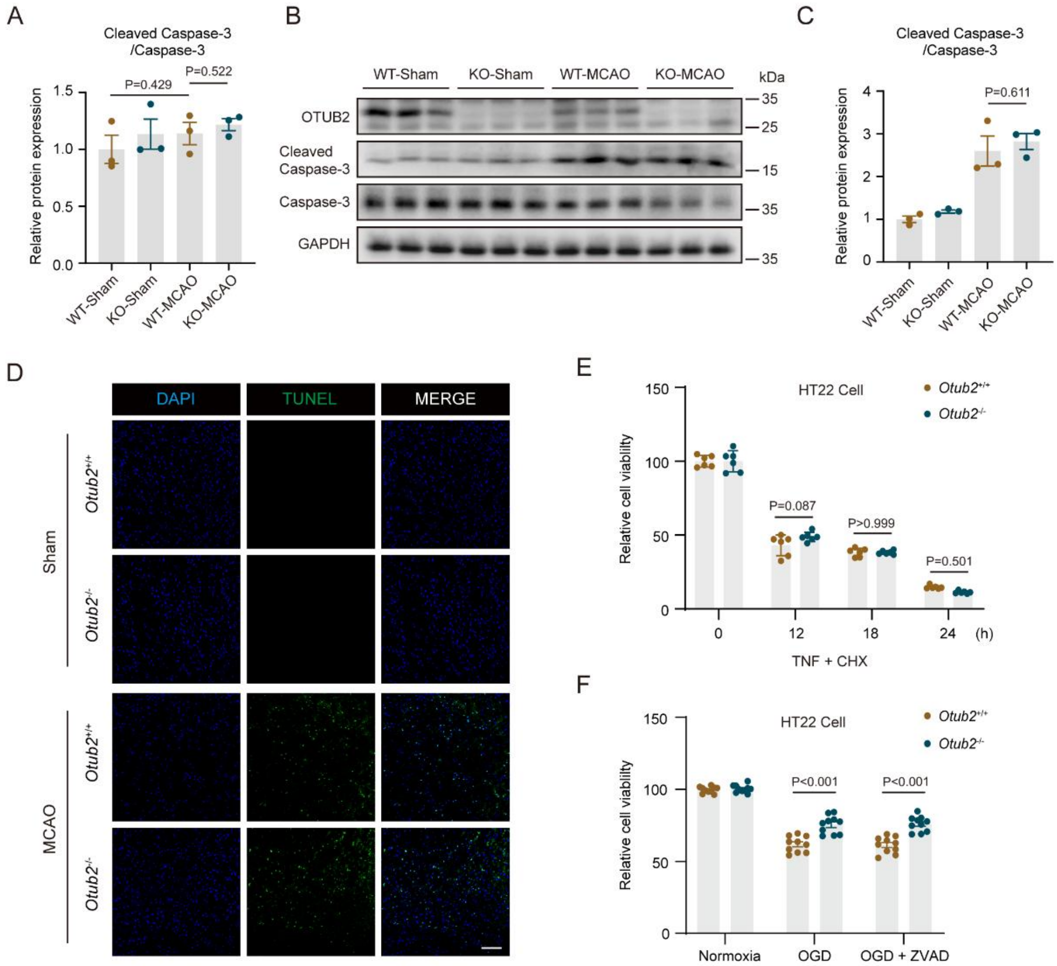


**Appendix Figure S4. OTUB2 deficiency does not affect neuronal apoptosis**

(A) Four hours after MCAO, the relative ratio of cleaved Caspase 3/Caspase 3 in the ischemic cerebral hemisphere was calculated based on Western blot results. Multiple Student's t tests, n = 3/group, biological replicates. (B-C) Twenty-four hours after MCAO, the ischemic cerebral hemisphere was analyzed by Western blot with indicated antibodies. Representative immunoblots (B) and the relative ratio of cleaved Caspase 3/Caspase 3 (C) are shown. Unpaired Student's t test, n = 3/group, biological replicates. (D) Twenty-four hours after MCAO, apoptotic cells in the ischemic

cerebral hemisphere were detected by TUNEL staining. Scale bar, 100 μm. (E) *Otub2*$^{+/+}$ and *Otub2*$^{-/-}$ HT22 cells were treated with TNF-α (20 ng/ml) and CHX (50 μM) for the indicated time. Cell viability was measured by CCK-8 test. Two-way ANOVA, n = 6/group, biological replicates. (F) *Otub2*$^{+/+}$ and *Otub2*$^{-/-}$ HT22 cells were subjected to OGD treatment in the presence or absence of Z-VAD-FMK (20 μM). Cell viability was measured by CCK-8 test. Two-way ANOVA, n = 10/group, biological replicates. Data in (A, C, E, F) show the mean ± SEM. Source data are available online for this figure.

**Appendix Figure S5**

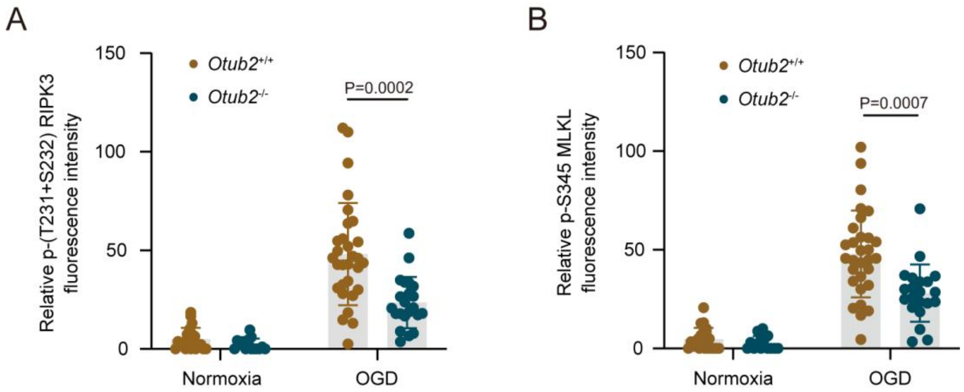


**Appendix Figure S5. OTUB2 deficiency reduces the activation of RIPK3 and MLKL after OGD**

(A-B) After OGD treatment for 6 h followed by reoxygenation for 12 h, *Otub2*$^{+/+}$ and *Otub2*$^{-/-}$ HT22 cells were analyzed by immunofluorescence with p-(T231+S232) RIPK3 and p-S345 MLKL antibodies. Data show the fluorescence intensity of p-RIPK3 (A) and p-MLKL (B). Unpaired Student's t test, n = 15-30/group, biological replicates. Data in (A, B) show the mean ± SEM. Source data are available online for this figure.

## Appendix Figure S6

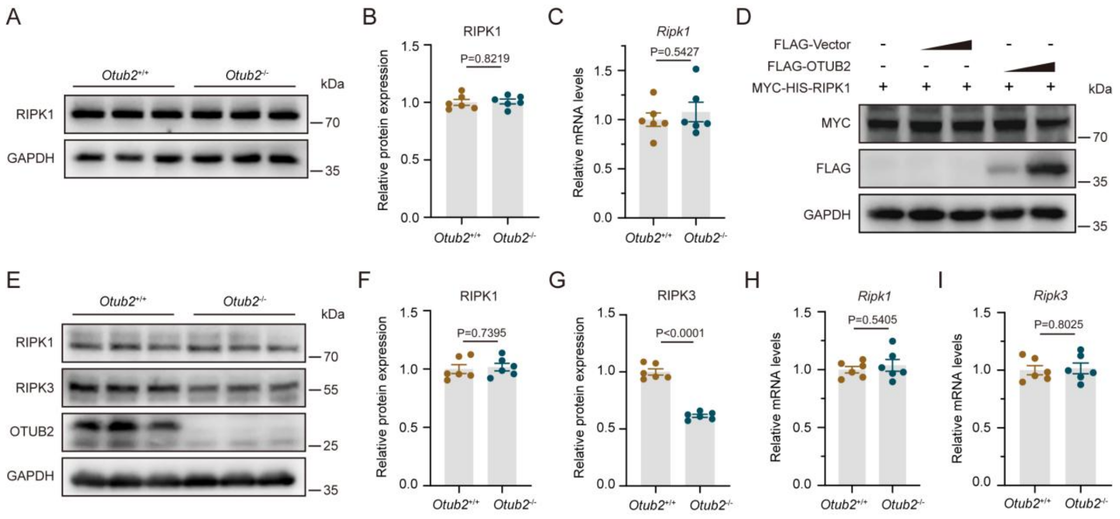


**Appendix Figure S6. OTUB2 deficiency has no impact on RIPK1 abundance**

(A-B) Whole-cell lysates of *Otub2*$^{+/+}$ and *Otub2*$^{-/-}$ HT22 cells were analyzed by Western blot with indicated antibodies. Representative immunoblots (A) and relative RIPK1 quantification (B) are shown. Unpaired Student's t test, n = 6/group, biological replicates. (C) Relative *Ripk1* mRNA levels in *Otub2*$^{+/+}$ and *Otub2*$^{-/-}$ HT22 cells were determined by qRT-PCR. Mann-Whitney U test, n = 6/group, biological replicates. (D) MYC-HIS-RIPK1 plasmids were co-transfected into NIH/3T3 cells with increasing amount of FLAG-Vector or FLAG-OTUB2 plasmids for 24 h. Whole-cell lysates were analyzed by Western blot. (E-G) Brains of untreated *Otub2*$^{+/+}$ and *Otub2*$^{-/-}$ mice were lysed and analyzed by Western blot with indicated antibodies. Representative immunoblots (E) as well as relative quantification of RIPK1 (F) and RIPK3 (G) are shown. Mann-Whitney U test (F) and unpaired Student's t test (G), n = 6/group, biological replicates. (H-I) Relative *Ripk1* (H) and *Ripk3* (I) mRNA levels in brains of

untreated *Otub2*$^{+/+}$ and *Otub2*$^{-/-}$ mice were determined by qRT-PCR. Unpaired Student's t test, n = 6/group, biological replicates. Data in (B, C, F-I) show the mean ± SEM. Source data are available online for this figure.

## Appendix Figure S7

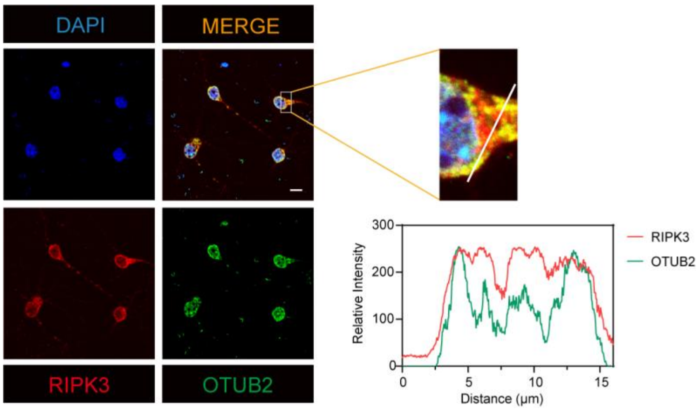


**Appendix Figure S7. Co-localization of OTUB2 and RIPK3 in primary neurons**

Subcellular distribution of OTUB2 (green) and RIPK3 (red) in primary neurons from C57BL/6 mice was analyzed by immunofluorescence. Scale bar, 10 μm. Source data are available online for this figure.

**Appendix Figure S8**

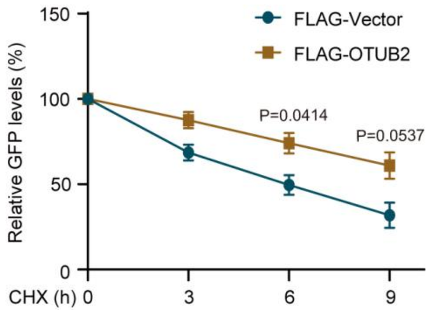


**Appendix Figure S8. OTUB2 inhibits the degradation of RIPK3**

GFP-RIPK3 plasmids were co-transfected into NIH/3T3 cells with FLAG-Vector or FLAG-OTUB2 plasmids for 24 h, followed by treatment with CHX (50 μM) for indicated time. Whole-cell lysates were analyzed by Western blot and relative levels of GFP-RIPK3 were quantified based on Western blot results. Multiple Student's t tests, n = 3/group, biological replicates. Data show the mean ± SEM. Source data are available online for this figure.

## Appendix Figure S9

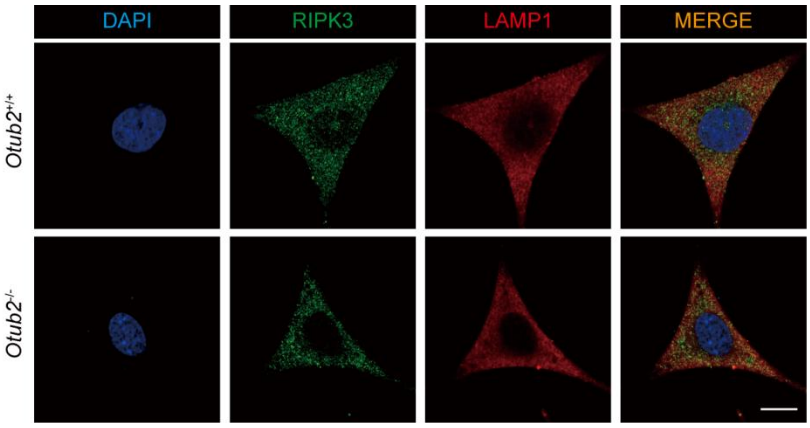


**Appendix Figure S9. OTUB2 deficiency does not increase the co-localization of RIPK3 with LAMP1**

Subcellular distribution of RIPK3 (green) and LAMP1 (red) in HT22 cells was determined by immunofluorescence. Scale bar, 10 μm. Source data are available online for this figure.

**Appendix Figure S10**

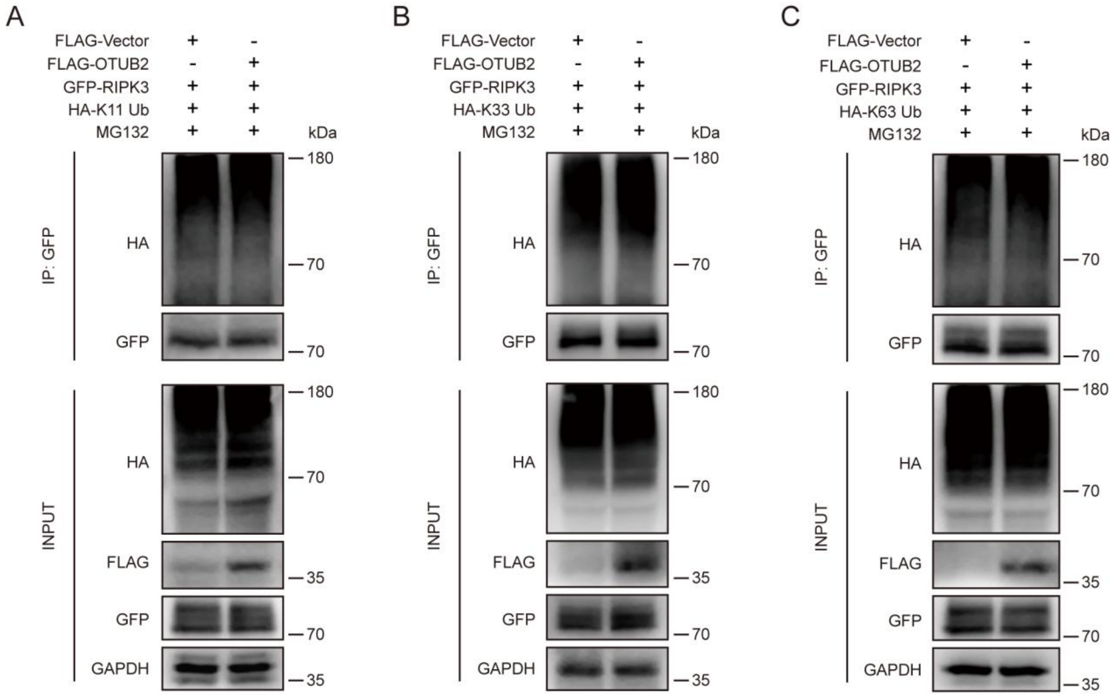


**Appendix Figure S10. OTUB2 does not affect the K11, K33, and K63 ubiquitination of RIPK3**

(A-C) NIH/3T3 cells were transfected with indicated plasmids for 24 h, followed by treatment with MG132 (5 μM) for 6 h. Immunocomplexes were harvested from whole-cell lysates by immunoprecipitation and analyzed by Western blot. Source data are available online for this figure.

## Appendix Figure S11

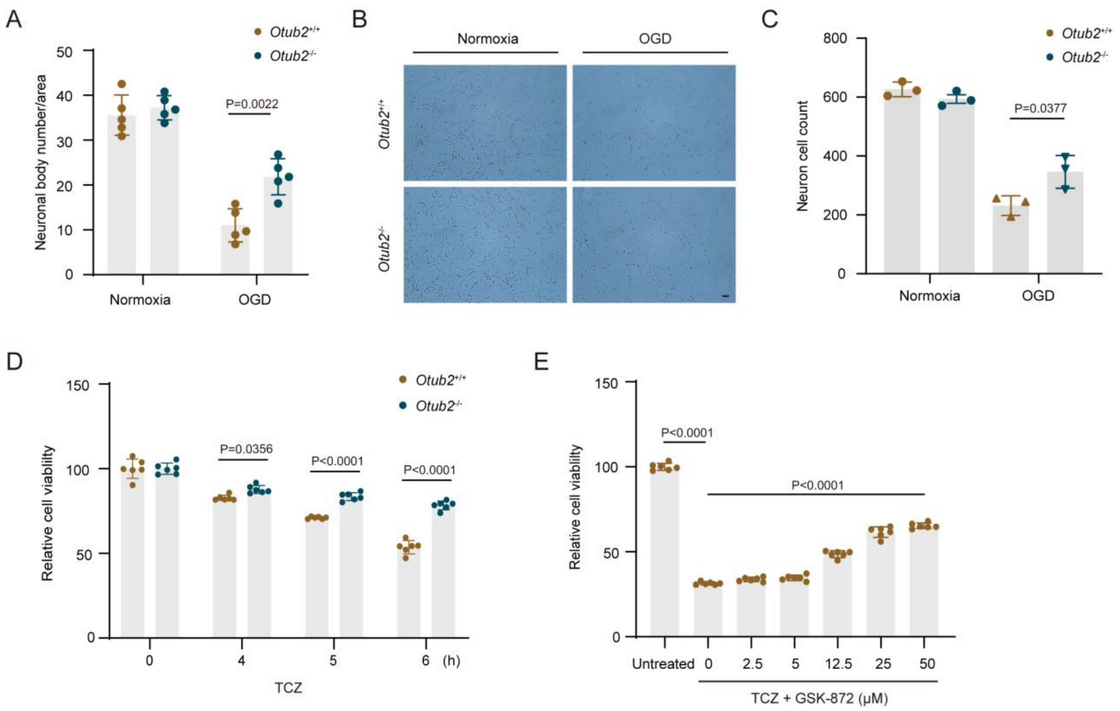


**Appendix Figure S11. OTUB2 deficiency attenuates necroptosis**

(A) Primary neurons from *Otub2*$^{+/+}$ and *Otub2*$^{-/-}$ mice were subjected to OGD for 6 h followed by reoxygenation for 12 h. The number of neurons was determined by immunofluorescence with anti-MAP2 antibody. Unpaired Student's t test, n = 5/group, biological replicates. (B-C) Primary neurons from *Otub2*$^{+/+}$ and *Otub2*$^{-/-}$ mice were subjected to OGD for 6 h followed by reoxygenation for 12 h. The number of neurons was counted with a brightfield microscope. Representative images (B) and cell number (C) are shown. Unpaired Student's t test, n = 3 /group, biological replicates. (D) *Otub2*$^{+/+}$ and *Otub2*$^{-/-}$ HT22 cells were stimulated with TNF-α (20 ng/ml) + CHX (50 μM) + Z-VAD-FMK (20 μM) for indicated time. Cell viability was determined by CCK-8 test. Two-way ANOVA, n = 6/group, biological replicates. (E) HT22 cells

were stimulated with TNF-α (20 ng/ml) + CHX (50 μM) + Z-VAD-FMK (20 μM) in the presence of indicated concentrations of GSK-872 for 6 h. Cell viability was determined by CCK-8 test. Multiple Student's t tests, n = 6/group, biological replicates. Data in (A, C, D, E) show the mean ± SEM. Source data are available online for this figure.

**Appendix Figure S12**

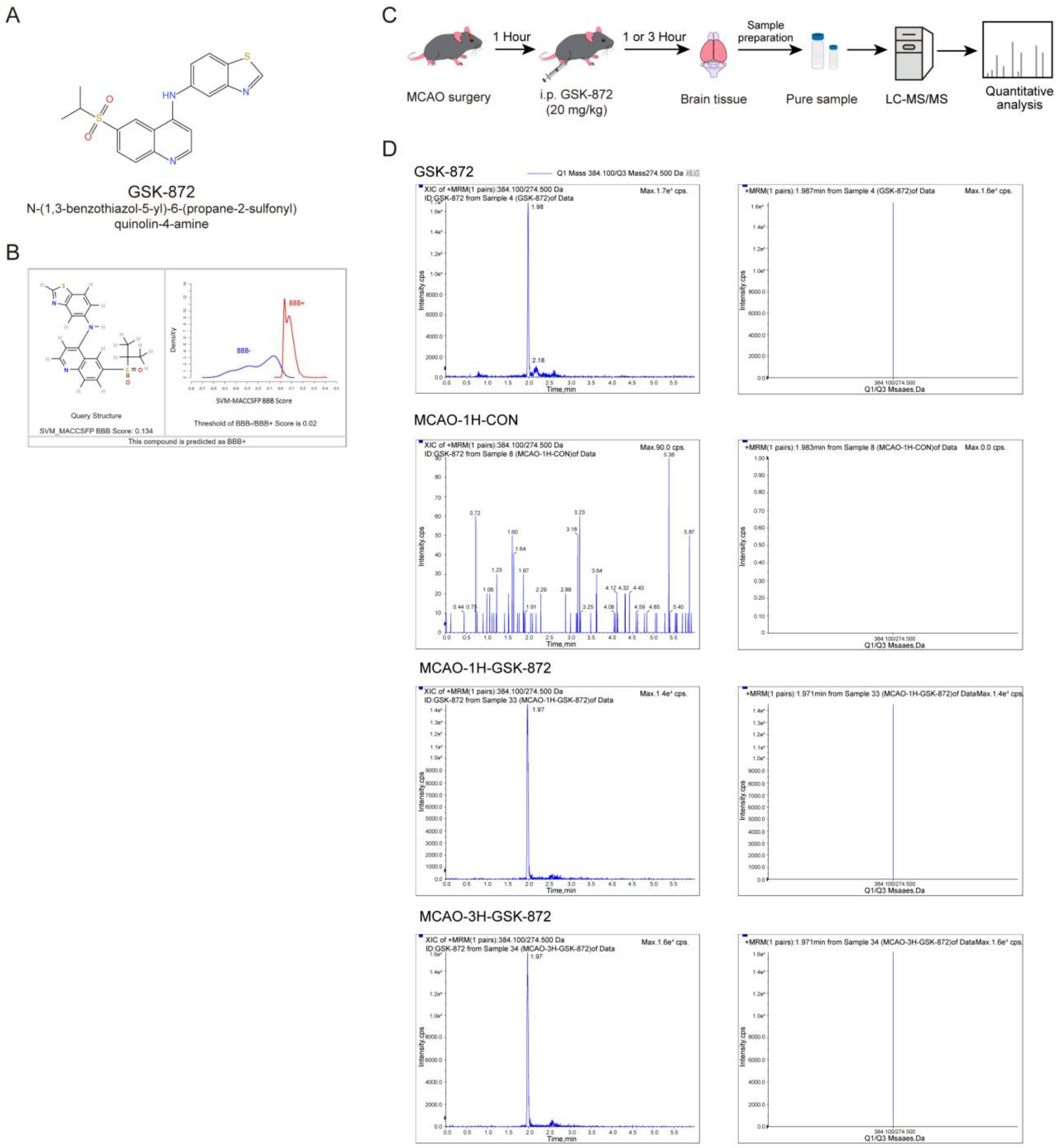


## Appendix Figure S12. GSK-872 can pass the BBB

(A) Chemical structure of GSK-872. (B) GSK-872 was predicted to pass the BBB by the online tool (GraphBBB). (C) Experimental flowchart for the analysis of GSK-872 in brain cells. (D) Results of the LC-MS/MS analysis. Source data are available online for this figure.

**Appendix Figure S13.**

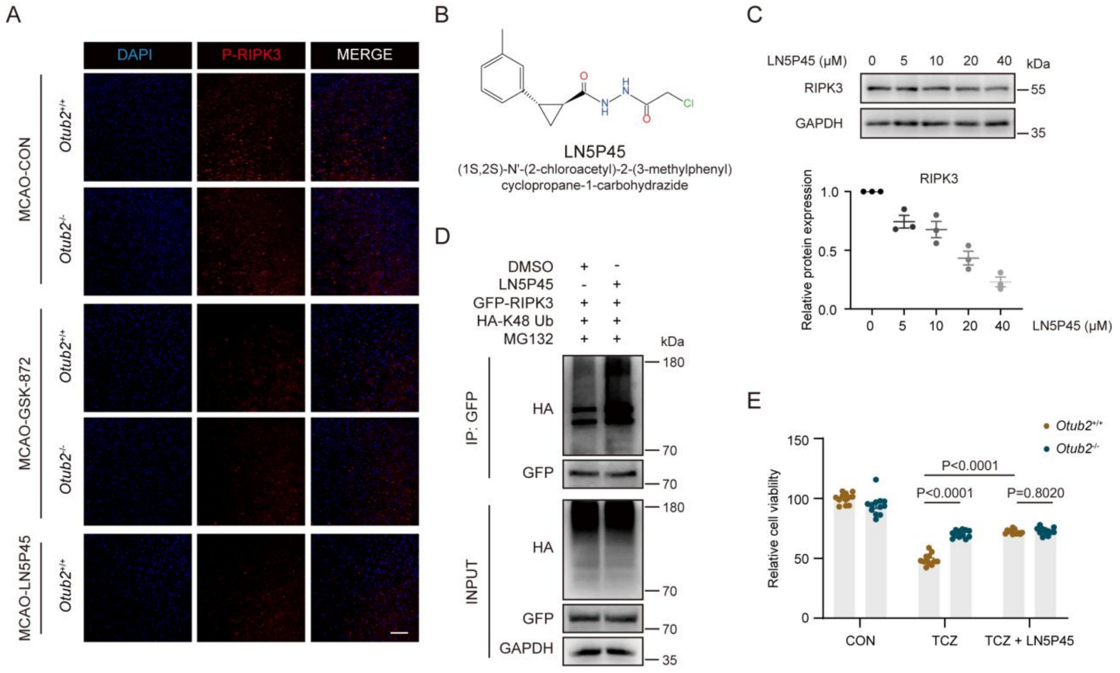


**Appendix Figure S13. Pharmacological inhibition of OTUB2 reduces RIPK3-mediated necroptosis**

(A) Mice were intraperitoneally injected with LN5P45 (10 mg/kg) prior to MCAO surgery. The ischemic penumbra was analyzed by immunofluorescence at 6 h after MCAO. Scale bar, 100 μm. (B) Chemical structure of LN5P45. (C) HT22 cells were treated with indicated concentrations of LN5P45 for 6 h before lysis. Whole-cell lysates were analyzed by Western blot. Representative immunoblots (upper panel) and relative RIPK3 protein levels (lower panel) are shown. (D) NIH/3T3 cells were transfected with indicated plasmids for 24 h. After transfection, cells were treated with MG132 (5 μM) in the presence or absence of LN5P45 (20 μM) for 6 h. Immunocomplexes and whole-cell lysates were analyzed by Western blot. (E) After treatment with LN5P45 (20 μM) for 6 h, HT22 cells were stimulated with TNF-α (20

ng/ml) + CHX (50 μM) + Z-VAD-FMK (20 μM) for another 6 h. Cell viability was determined by CCK-8 test. Mann-Whitney U test, n = 12/group, biological replicates. Data in (C, E) show the mean ± SEM. Source data are available online for this figure.

**Appendix Figure S14**

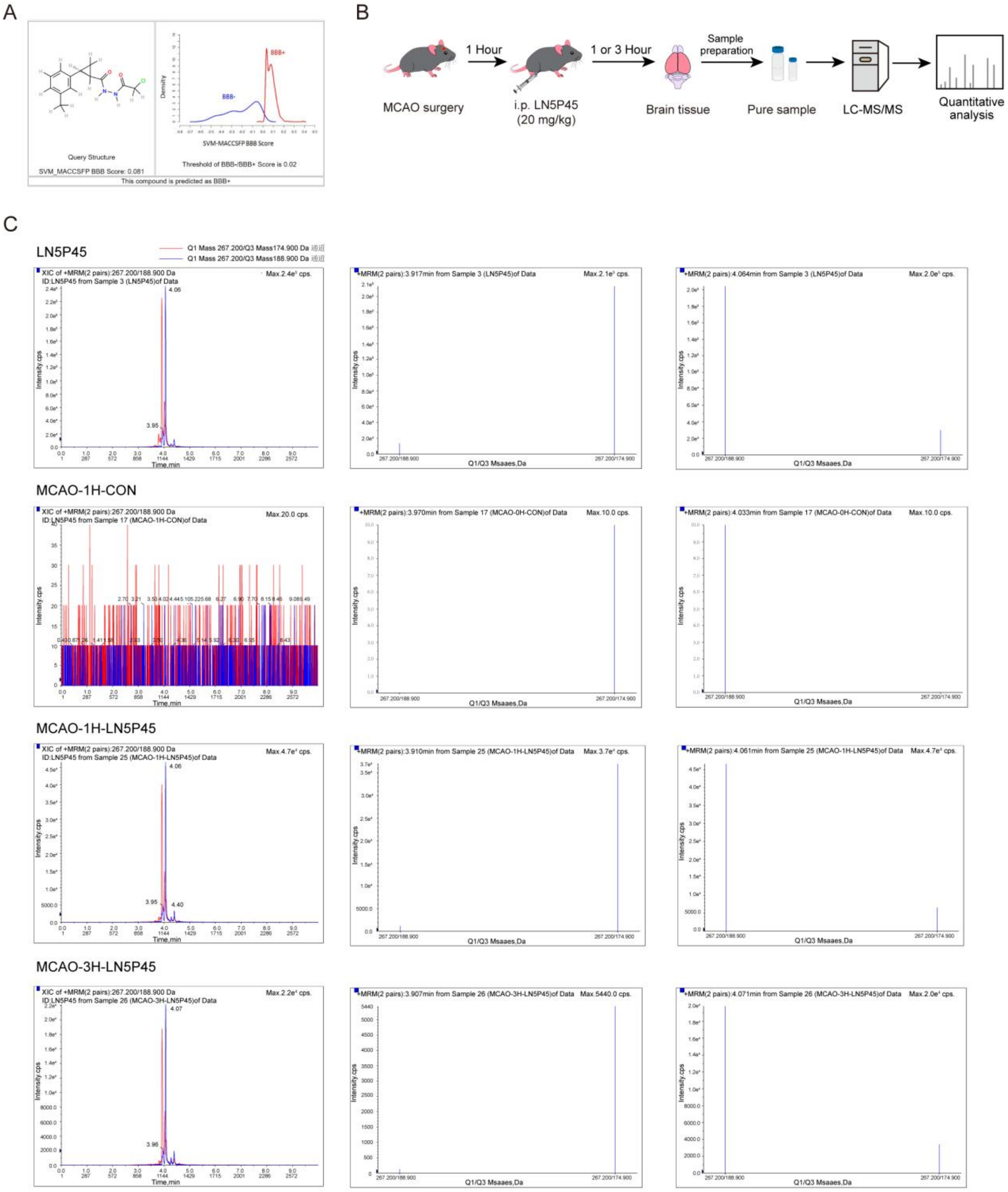


**Appendix Figure S14. LN5P45 can pass the BBB**

(A) LN5P45 was predicted to pass the BBB by the online tool (GraphBBB). (B) Experimental flowchart for the detection of LN5P45 in brain cells. (C) Results of the LC-MS/MS analysis. Source data are available online for this figure.

**Appendix Figure S15**

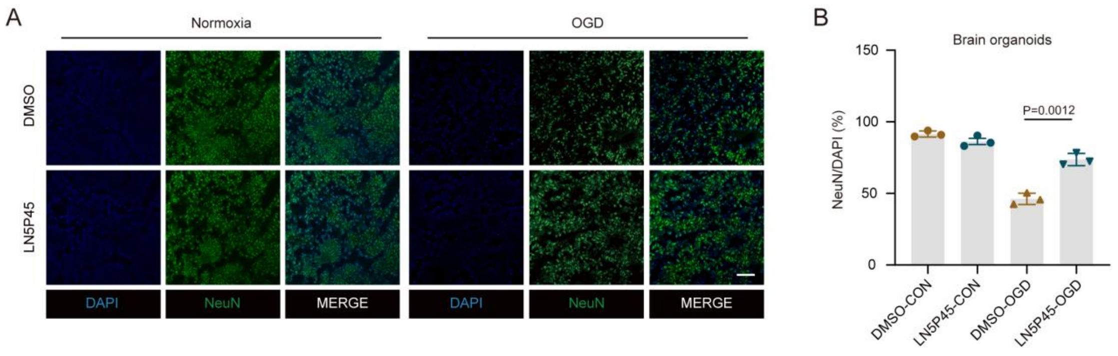


**Appendix Figure S15. Pharmacological inhibition of OTUB2 reduces OGD-induced neuronal loss in human brain organoids**

(A-B) On day 30 after culture, brain organoids were subjected to OGD for 12 h, followed by 12 h of reoxygenation. The brain organoids were then analyzed by immunofluorescence with indicated antibodies. Representative images (A) and quantification (B) are shown. Scale bar, 50 µm. Unpaired Student's t test, n = 3/group, biological replicates. Data in (B) show the mean ± SEM. Source data are available online for this figure.

212 **Appendix Table S1 Primers for quantitative Real-Time PCR (qRT-PCR)**

| Reagent/Resource | Reference or source | Identifier or Catalog Number |
|---|---|---|
| *Ccl2* reverse primer (M. musculus) | 5'-GCATTAGCTTCAGA TTTACGGGT-3' | N/A |
| *Ccl2* forward primer (M. musculus) | 5'-TAAAAACCTGGAT CGGAACCAAA-3' | N/A |
| *Cxcl10* reverse primer (M. musculus) | 5'-CCTATGGCCCTCAT TCTCAC-3' | N/A |
| *Cxcl10* forward primer (M. musculus) | 5'-CTCATCCTGCTGGG TCTGAG-3' | N/A |
| *Il1b* reverse primer (M. musculus) | 5'-TGAGGCCCAAGGCC ACAGGT-3' | N/A |
| *Il1b* forward primer (M. musculus) | 5'-AAGCCTCGTGCTGT CGGACC-3' | N/A |
| *Il6* reverse primer (M. musculus) | 5'-AAGTGCATCATCGT TGTTCATACA-3' | N/A |
| *Il6* forward primer (M. musculus) | 5'-ACACATGTTCTCTG GGAAATCGT-3' | N/A |
| *Otub2* reverse primer (M. musculus) | 5'-GTAAGACAAGACGG AGAACAGAAGG-3' | N/A |
| *Otub2* forward primer (M. musculus) | 5'-ACCTCATTCCTCGTT CCATCTG-3' | N/A |
| *Ripk1* reverse primer (M. musculus) | 5'-TGCCCAAAGGACAA TGCCAAAG-3' | N/A |
| *Ripk1* forward primer (M. musculus) | 5'-AGCAGCACCACTAA GAAGAACAATG-3' | N/A |

| Reagent/Resource | Reference or source | Identifier or Catalog Number |
|---|---|---|
| *Ripk3* reverse primer (M. musculus) | 5'-TCAGAACAGTTGTT GAAGACGAGAG-3' | N/A |
| *Ripk3* forward primer (M. musculus) | 5'-ACACGGCACTCCTT GGTATCC-3' | N/A |
| *Tnf* reverse primer (M. musculus) | 5'-ATAGCAAATCGGCT GACGGT-3' | N/A |
| *Tnf* forward primer (M. musculus) | 5'-ACGTCGTAGCAAA CCACCAA-3' | N/A |
| *β-actin* reverse primer (M. musculus) | 5'-CACAGCTTCTCTTT GATGTCAC-3' | N/A |
| *β-actin* forward primer (M. musculus) | 5'-CTACCTCATGAAGA TCCTGACC-3' | N/A |

213

## Statistics Tables

## Appendix Table S2

| Figure | Analysis of normality | Statistical method | Power | p value |
|---|---|---|---|---|
| Fig 1 B | Yes | two-tailed unpaired Student's t test | 1.000 | <0.0001 |
| Fig 1 F(day3/day7) | Yes | two-way ANOVA followed by the Sidak post-test | 0.945/0.644 | 0.0004/0.0310 |
| Fig 1 G (day1/day3/day7) | Yes | two-way ANOVA followed by the Sidak post-test | 0.925/0.717/0.903 | 0.0052/0.0393/0.0052 |
| Fig 1 H (day1/day3/day7) | Yes | two-way ANOVA followed by the Sidak post-test | 0.855/0.953/0.807 | 0.0019/0.0015/0.0070 |
| Fig 1 I (day1/day3/day7) | Yes | two-way ANOVA followed by the Sidak post-test | 0.963/0.292/0.566 | 0.0289/0.2859/0.1794 |
| Fig 1 J (day1/day3/day7) | Yes | two-way ANOVA followed by the Sidak post-test | 0.949/0.822/0.836 | 0.0004/0.0080/0.1074 |
| Fig 1 K (day1/day3/day7) | Yes | two-way ANOVA followed by the Sidak post-test | 0.755/0.310/0.212 | 0.0410/0.2718/0.5269 |

## Appendix Table S3

| Figure | Analysis of normality | Statistical Method | Power | p value |
|---|---|---|---|---|
| Fig 2 C | Yes | two-tailed unpaired Student's t test | 0.568 | 0.0438 |
| Fig 2 D | Yes | two-tailed unpaired Student's t test | 0.102 | 0.4637 |
| Fig 2 F | Yes | two-tailed unpaired Student's t test | 0.999 | 0.0004 |
| Fig 2 I | No | Mann-Whitney U test | 0.758 | 0.0164 |
| Fig 2 J | Yes | two-tailed unpaired Student's t test | 0.708 | 0.0435 |
| Fig 2 K | Yes | two-tailed unpaired Student's t test | 0.945 | 0.0022 |
| Fig 2 L | Yes | two-tailed unpaired Student's t test | 0.520 | 0.0492 |
| Fig 2 M | Yes | two-tailed unpaired Student's t test | 0.364 | 0.1056 |

## Appendix Table S4

| Figure | Analysis of normality | Statistical method | Power | p value |
|---|---|---|---|---|
| Fig 3 B | Yes | two-way ANOVA followed by the Sidak post-test | 1.000/0.221 | 0.0003/0.3863 |
| Fig 3 C | Yes | two-way ANOVA followed by the Sidak post-test | 1.000/0.604 | 0.0011/0.0329 |
| Fig 3 D | Yes | two-way ANOVA followed by the Sidak post-test | 1.000/0.585 | 0.0011/0.0477 |
| Fig 3 F | Yes | two-way ANOVA followed by the Sidak post-test | 0.848/0.970 | 0.0202/0.0461 |
| Fig 3 I | Yes | two-tailed unpaired Student's t test | 1.000 | 0.0008 |
| Fig 3 J | Yes | two-tailed unpaired Student's t test | 0.610 | 0.0341 |
| Fig 3 K | Yes | two-tailed unpaired Student's t test | 0.999 | 0.0004 |
| Fig 3 L | Yes | two-tailed unpaired Student's t test | 0.586 | 0.0382 |
| Fig 3 N | Yes | two-tailed unpaired Student's t test | 0.956 | 0.0030 |
| Fig 3 O | Yes | two-way ANOVA followed by the Sidak post-test | 0.866/0.070 | 0.0005/0.9566 |

## Appendix Table S5

| Figure | Analysis of normality | Statistical method | Power | p value |
|---|---|---|---|---|
| Fig 4 B | Yes | two-tailed unpaired Student's t test | 1.000 | <0.0001 |
| Fig 4 C | Yes | two-tailed unpaired Student's t test | 0.104 | 0.4711 |

## Appendix Table S6

| Figure | Analysis of normality | Statistical method | Power | p value |
|---|---|---|---|---|
| Fig 6 A | Yes | two-tailed unpaired Student's t test | 1.000 | <0.0001 |
| Fig 6 E | No | Mann-Whitney U test | 0.996 | <0.0001 |
| Fig 6 F | Yes | two-tailed unpaired Student's t test | 0.953 | <0.0001 |
| Fig 6 G | Yes | two-way ANOVA followed by the Sidak post-test | 1.000/0.606/0.311 | <0.0001/0.0603/0.6878 |
| Fig 6 H | Yes | two-way ANOVA followed by the Sidak post-test | 0.995/0.112 | 0.0005/0.7816 |

## Appendix Table S7

| Figure | Analysis of normality | Statistical method | Power | p value |
|---|---|---|---|---|
| Fig 7 C | Yes | Multiple Student's t tests | 0.997/0.904/0.984 | 0.0006/0.0057/0.0015 |
| Fig 7 D | Yes | Multiple Student's t tests | 0.878/0.675/0.963 | 0.0072/0.0249/0.0026 |
| Fig 7 H | Yes | Multiple Student's t tests | 0.992/0.864/0.971 | 0.0010/0.0081/0.0022 |
| Fig 7 I | No | Mann-Whitney U test | 0.891/0.759/0.979 | 0.0063/0.0193/0.0064 |
| Fig 7 N | Yes | two-tailed unpaired Student's t test | 0.986 | 0.0045 |

**Appendix Table S8.**

| Figure | Analysis of normality | Statistical method | Power | p value |
|---|---|---|---|---|
| Appendix Fig S1 B | Yes | Multiple Student’s t tests | 0.052/0.153 | 0.8826/0.3249 |
| Appendix Fig S1 F | Yes | two-tailed unpaired Student’s t test | 0.055 | 0.8255 |
| Appendix Fig S1 H | Yes | two-tailed unpaired Student’s t test | 0.093 | 0.5122 |
| Appendix Fig S1 I | Yes | two-tailed unpaired Student’s t test | 0.060 | 0.7568 |

**Appendix Table S9**

| Figure | Analysis of normality | Statistical method | Power | p value |
|---|---|---|---|---|
| Appendix Fig S2 B | Yes | two-tailed unpaired Student’s t test | 0.989 | 0.0012 |
| Appendix Fig S2 C | No | Mann-Whitney U test | 0.810 | 0.0116 |

**Appendix Table S10**

| Figure | Analysis of normality | Statistical method | Power | p value |
|---|---|---|---|---|
| Appendix Fig S3 A | No | Mann-Whitney U test | 0.056 | 0.7732 |
| Appendix Fig S3 B | Yes | two-tailed unpaired Student’s t test | 0.050 | 0.9868 |
| Appendix Fig S3 C | Yes | two-tailed unpaired Student’s t test | 0.050 | 0.9811 |
| Appendix Fig S3 D | Yes | two-tailed unpaired Student’s t test | 0.092 | 0.4868 |
| Appendix Fig S3 E | Yes | two-tailed unpaired Student’s t test | 0.064 | 0.6762 |

## Appendix Table S11

| Figure | Analysis of normality | Statistical method | Power | p value |
|---|---|---|---|---|
| Appendix Fig S4 A | Yes | Multiple Student’s t tests | 0.106/0.085 | 0.4289/0.5225 |
| Appendix Fig S4 C | Yes | two-tailed unpaired Student’s t test | 0.072 | 0.6115 |
| Appendix Fig S4 E | Yes | two-way ANOVA followed by the Sidak post-test | 0.372/0.050/0.999 | 0.0869/>0.9999/0.5012 |
| Appendix Fig S4 F | Yes | two-way ANOVA followed by the Sidak post-test | 0.998/1.000 | <0.0001/<0.0001 |

## Appendix Table S12

| Figure | Analysis of normality | Statistical method | Power | p value |
|---|---|---|---|---|
| Appendix Fig S5 A | Yes | two-tailed unpaired Student’s t test | 0.992 | 0.0002 |
| Appendix Fig S5 B | Yes | two-tailed unpaired Student’s t test | 0.967 | 0.0007 |

## Appendix Table S13

| Figure | Analysis of normality | Statistical method | Power | p value |
|---|---|---|---|---|
| Appendix Fig S6 B | Yes | two-tailed unpaired Student’s t test | 0.055 | 0.8219 |
| Appendix Fig S6 C | No | Mann-Whitney U test | 0.086 | 0.5427 |
| Appendix Fig S6 F | No | Mann-Whitney U test | 0.060 | 0.7395 |
| Appendix Fig S6 G | Yes | two-tailed unpaired Student’s t test | 1.000 | <0.0001 |
| Appendix Fig S6 H | Yes | two-tailed unpaired Student’s t test | 0.089 | 0.5405 |
| Appendix Fig S6 I | Yes | two-tailed unpaired Student’s t test | 0.056 | 0.8205 |

## Appendix Table S14

| Figure | Analysis of normality | Statistical method | Power | p value |
|---|---|---|---|---|
| Appendix Fig S8 | Yes | Multiple Student's t tests | 0.609/0.537 | 0.0414/0.0537 |

## Appendix Table S15

| Figure | Analysis of normality | Statistical method | Power | p value |
|---|---|---|---|---|
| Appendix Fig S11 A | Yes | two-tailed unpaired Student's t test | 0.971 | 0.0022 |
| Appendix Fig S11 C | Yes | two-tailed unpaired Student's t test | 0.635 | 0.0377 |
| Appendix Fig S11 D | Yes | two-way ANOVA followed by the Sidak post-test | 0.975/1.000/1.000 | 0.0356/<0.0001/<0.0001 |
| Appendix Fig S11 E | Yes | Multiple Student's t tests | 1.000/1.000 | <0.0001/<0.0001 |

## Appendix Table S16

| Figure | Analysis of normality | Statistical method | Power | p value |
|---|---|---|---|---|
| Appendix Fig S13 E | No | Mann-Whitney U test | 1.000/1.000/0.056 | <0.0001/<0.0001/0.8020 |

## Appendix Table S17

| Figure | Analysis of normality | Statistical method | Power | p value |
|---|---|---|---|---|
| Appendix Fig S15 B | Yes | two-tailed unpaired Student's t test | 1.000 | 0.0012 |
