# Supplementary figures and images for: Deubiquitination of RIPK3 by OTUB2 potentiates neuronal necroptosis after ischemic stroke (part 1 of 3)
Source: EMBO Mol Med. 2025 Feb 28;17(4):679–95. doi: 10.1038/s44321-025-00206-6 (PMC11982199; doi:10.1038/s44321-025-00206-6)

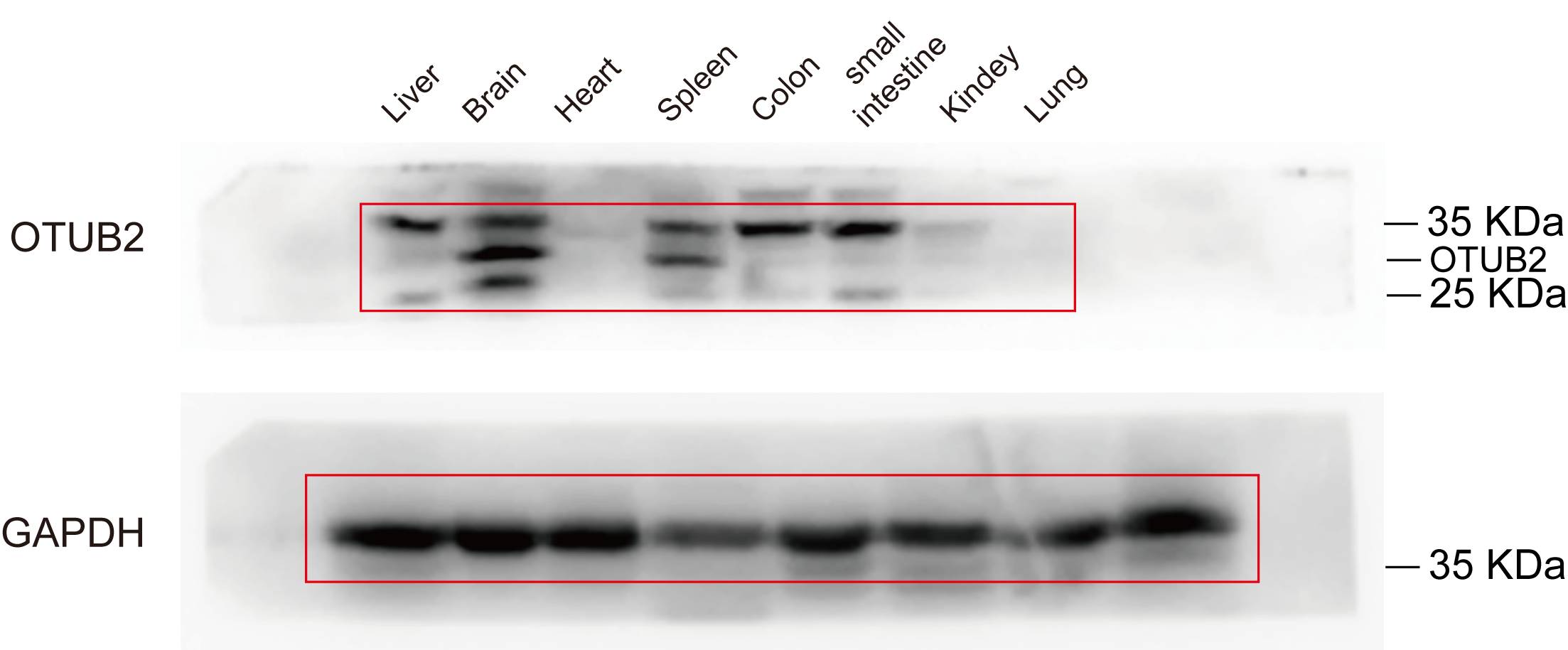

Supplement: Supplementary file 3 — Source data Fig. 1 [file 44321_2025_206_MOESM3_ESM.zip › Source Data Fig 1/Fig 1/1A/1A.jpg]

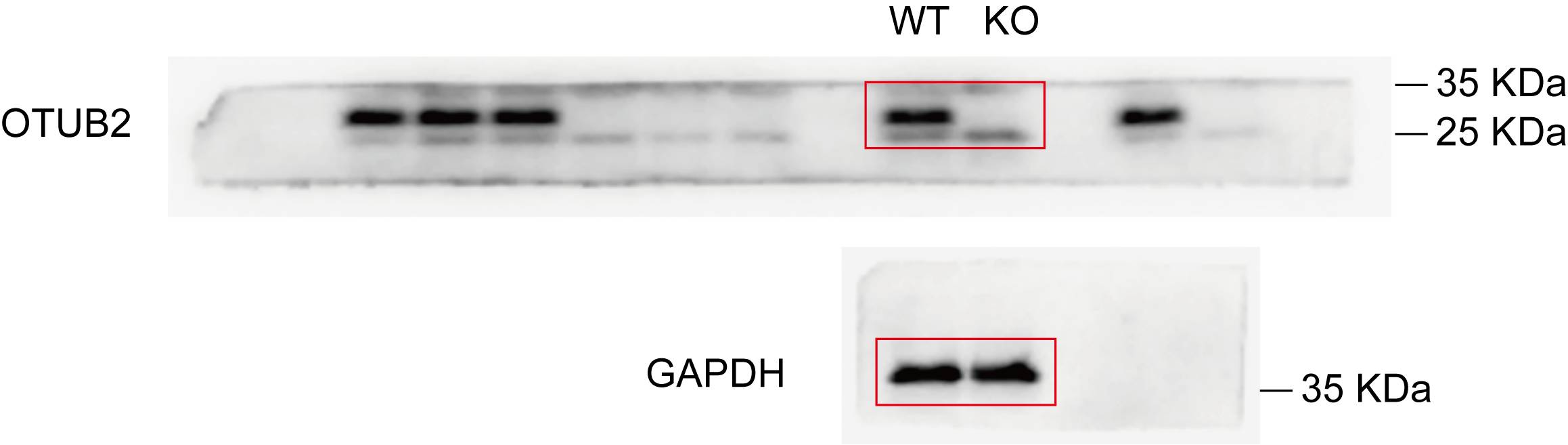

Supplement: Supplementary file 3 — Source data Fig. 1 [file 44321_2025_206_MOESM3_ESM.zip › Source Data Fig 1/Fig 1/1B/1B.jpg]

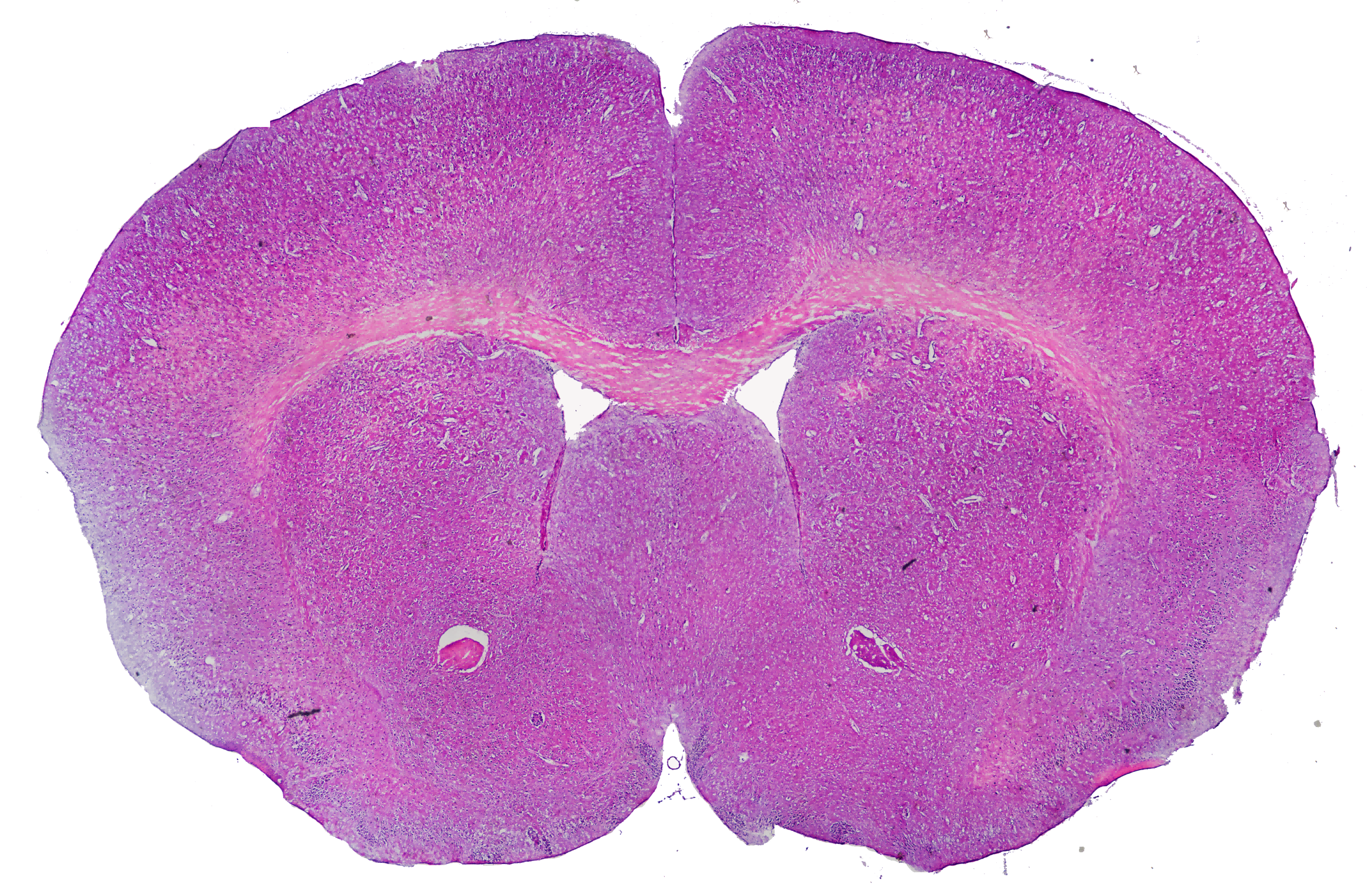

Supplement: Supplementary file 3 — Source data Fig. 1 [file 44321_2025_206_MOESM3_ESM.zip › Source Data Fig 1/Fig 1/1C/KO-HE.png]

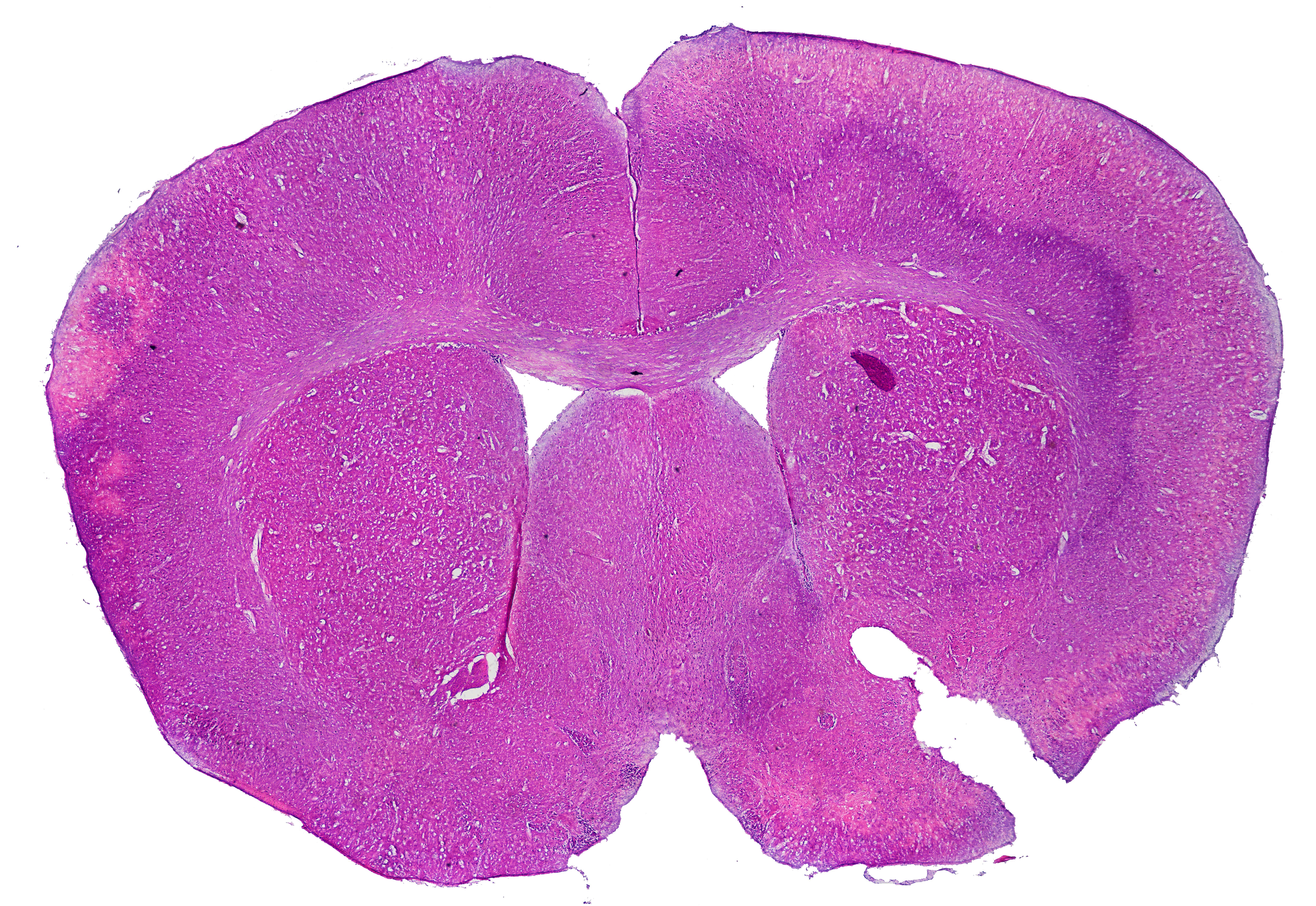

Supplement: Supplementary file 3 — Source data Fig. 1 [file 44321_2025_206_MOESM3_ESM.zip › Source Data Fig 1/Fig 1/1C/WT-HE.tif]

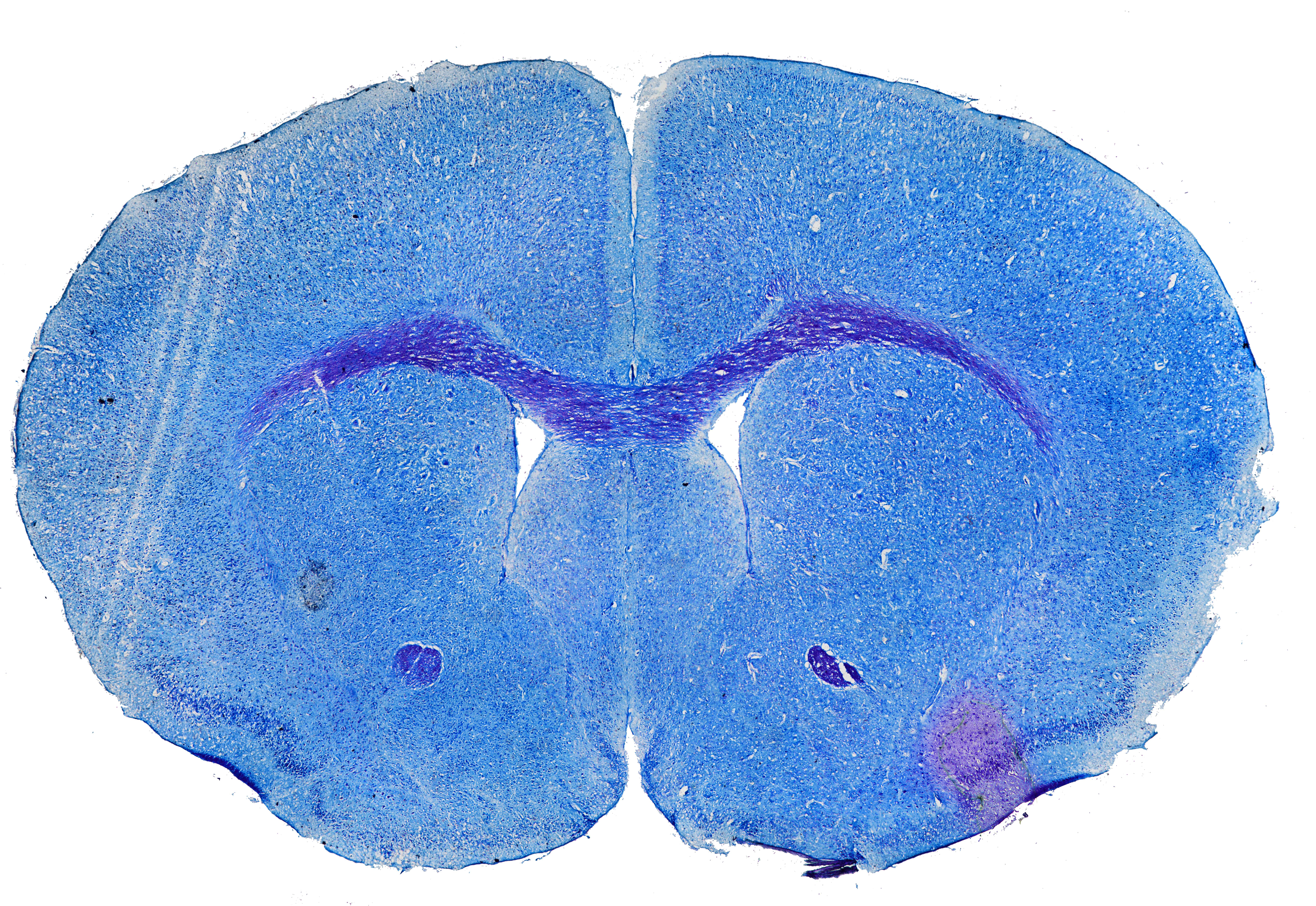

Supplement: Supplementary file 3 — Source data Fig. 1 [file 44321_2025_206_MOESM3_ESM.zip › Source Data Fig 1/Fig 1/1D/KO-Nissl.tif]

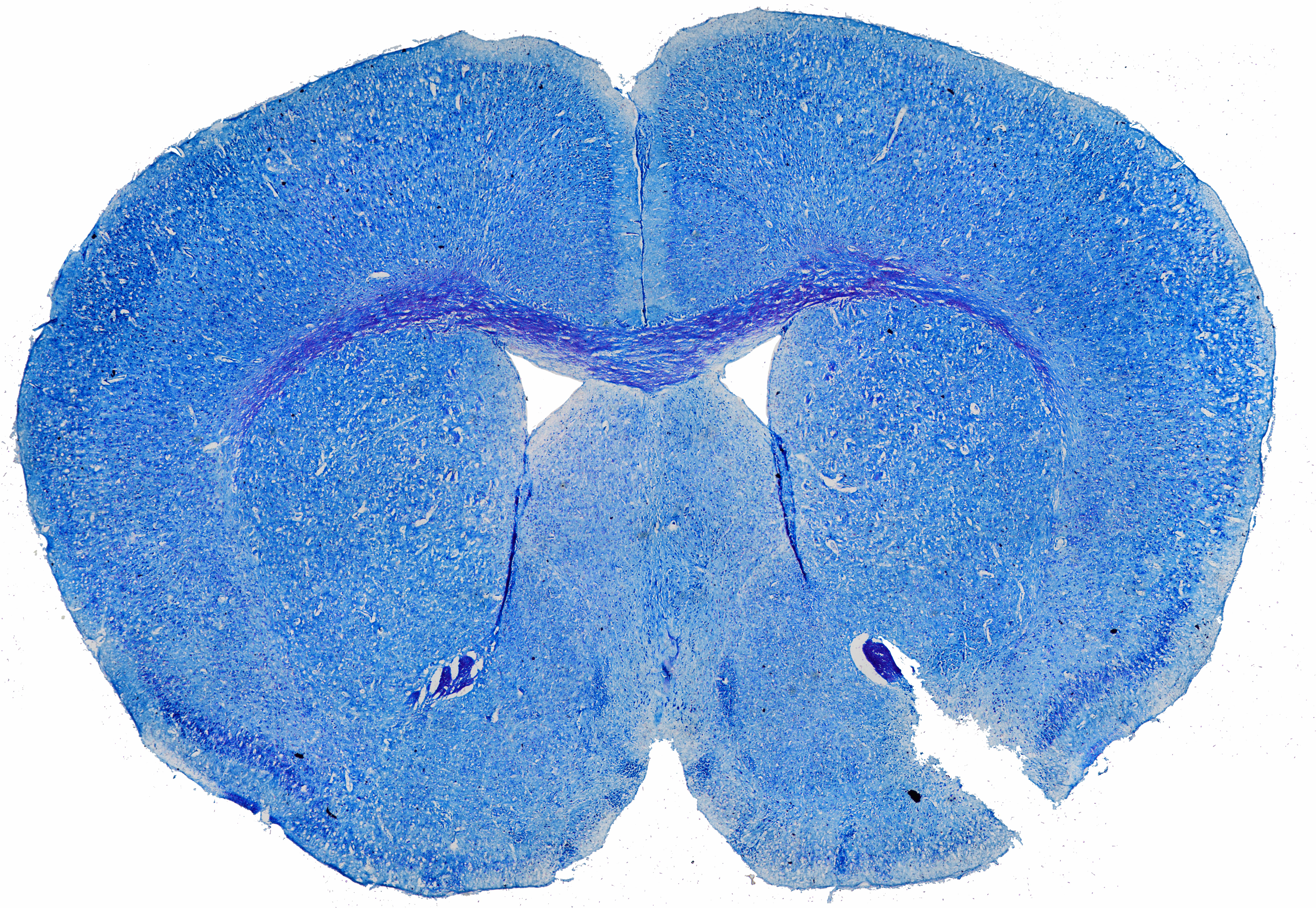

Supplement: Supplementary file 3 — Source data Fig. 1 [file 44321_2025_206_MOESM3_ESM.zip › Source Data Fig 1/Fig 1/1D/WT-Nissl.tif]

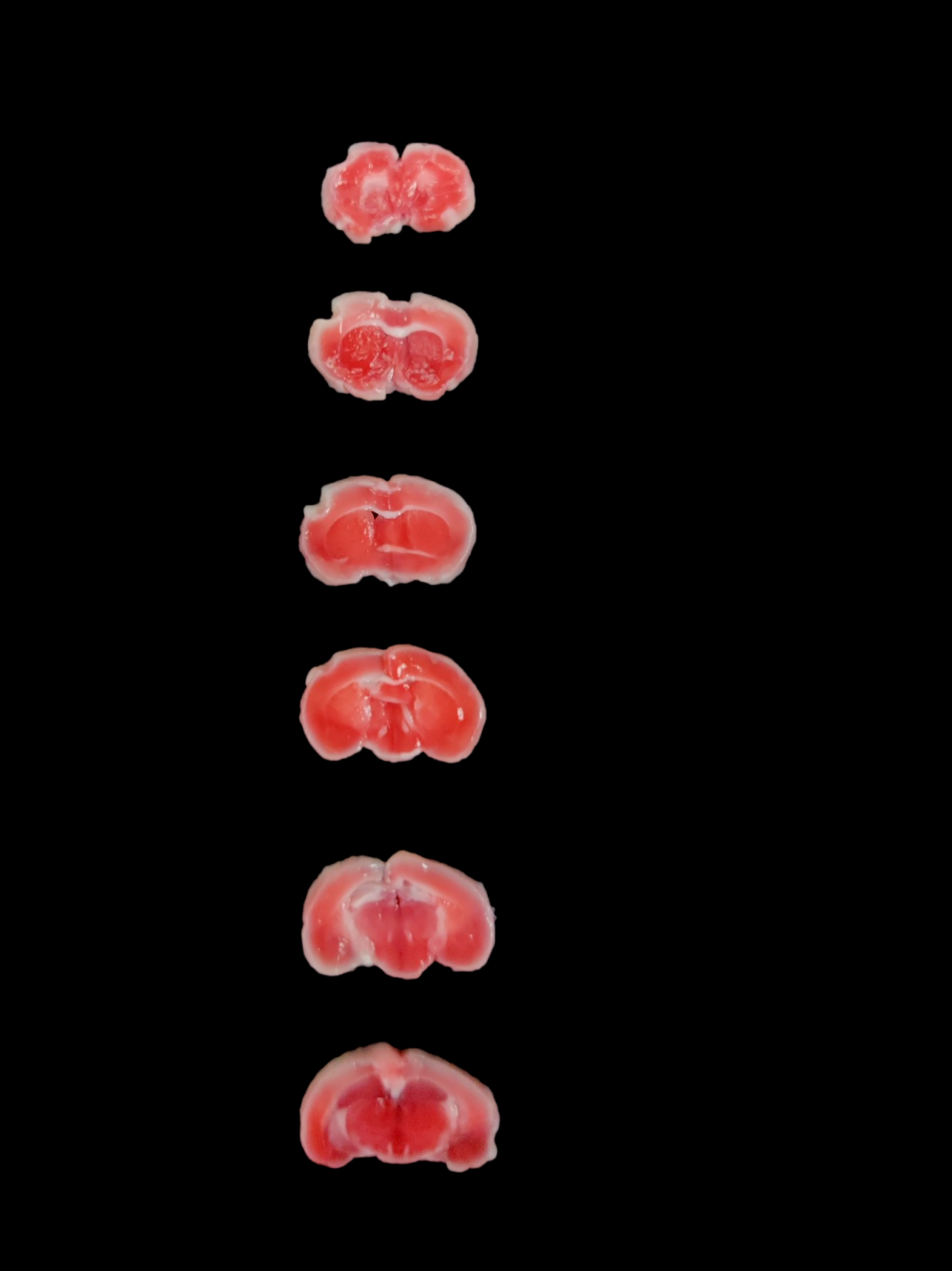

Supplement: Supplementary file 3 — Source data Fig. 1 [file 44321_2025_206_MOESM3_ESM.zip › Source Data Fig 1/Fig 1/1E/KO-D0.png]

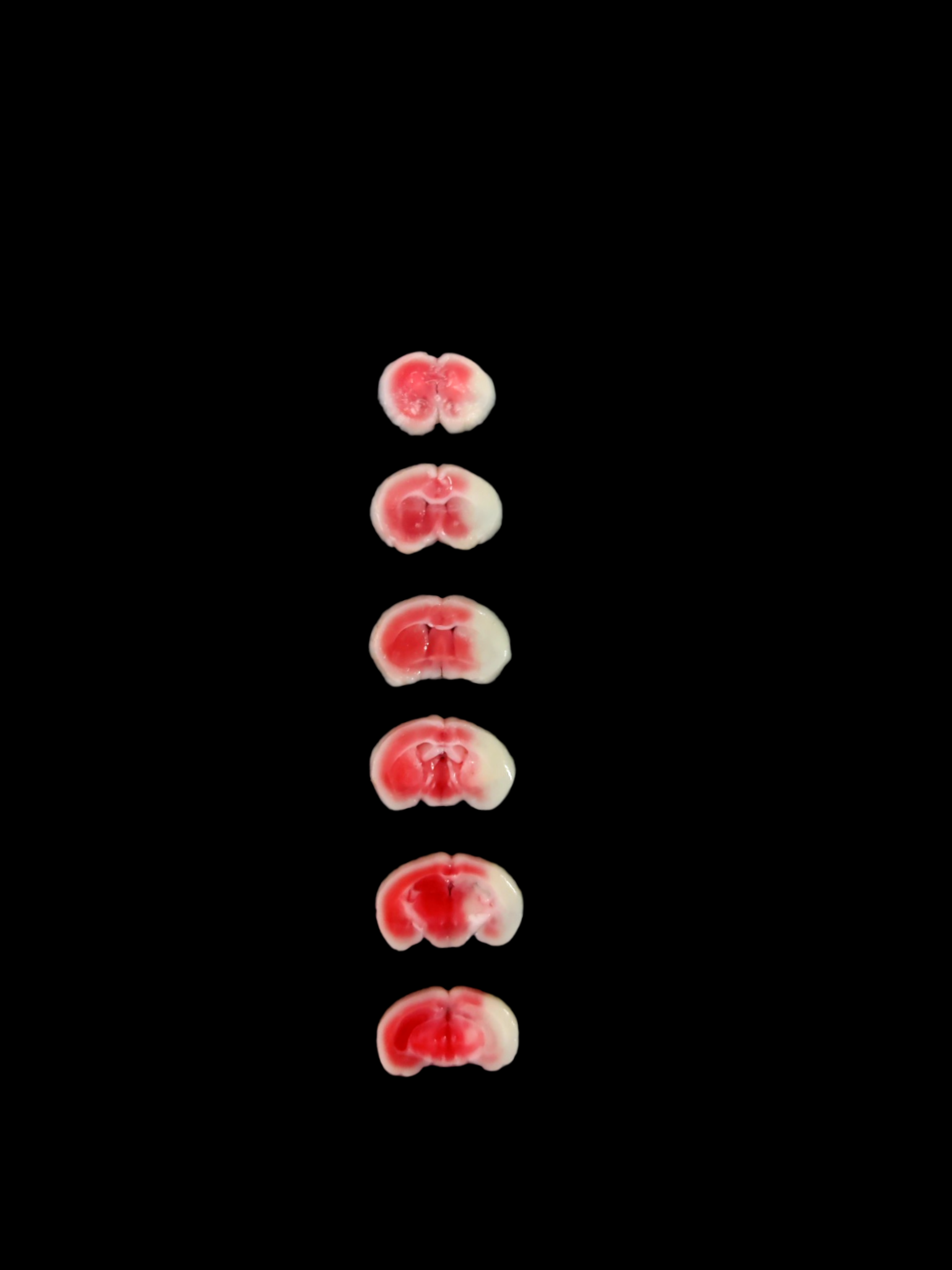

Supplement: Supplementary file 3 — Source data Fig. 1 [file 44321_2025_206_MOESM3_ESM.zip › Source Data Fig 1/Fig 1/1E/KO-D3.tif]

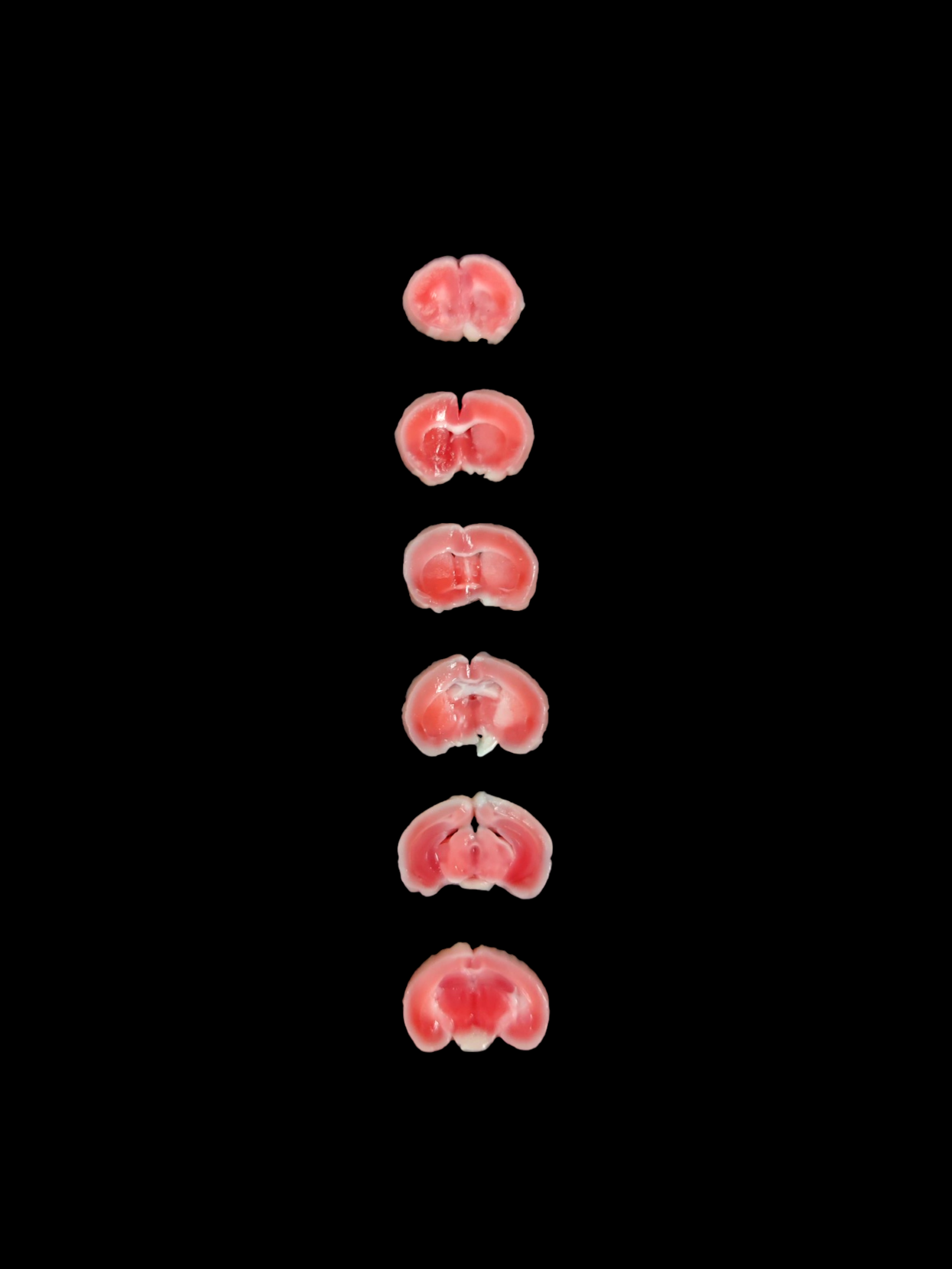

Supplement: Supplementary file 3 — Source data Fig. 1 [file 44321_2025_206_MOESM3_ESM.zip › Source Data Fig 1/Fig 1/1E/KO-D7.tif]

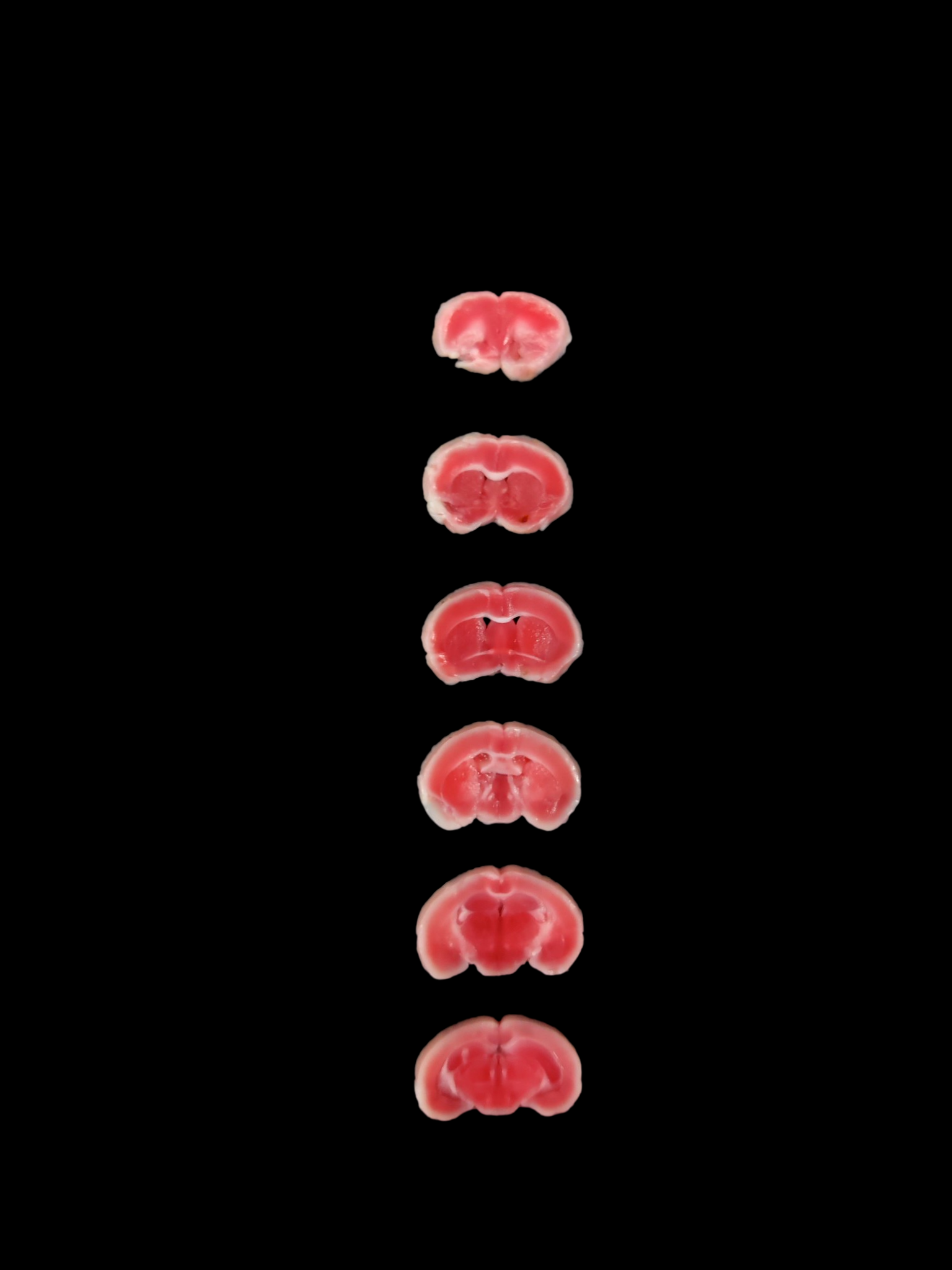

Supplement: Supplementary file 3 — Source data Fig. 1 [file 44321_2025_206_MOESM3_ESM.zip › Source Data Fig 1/Fig 1/1E/WT-D0.tif]

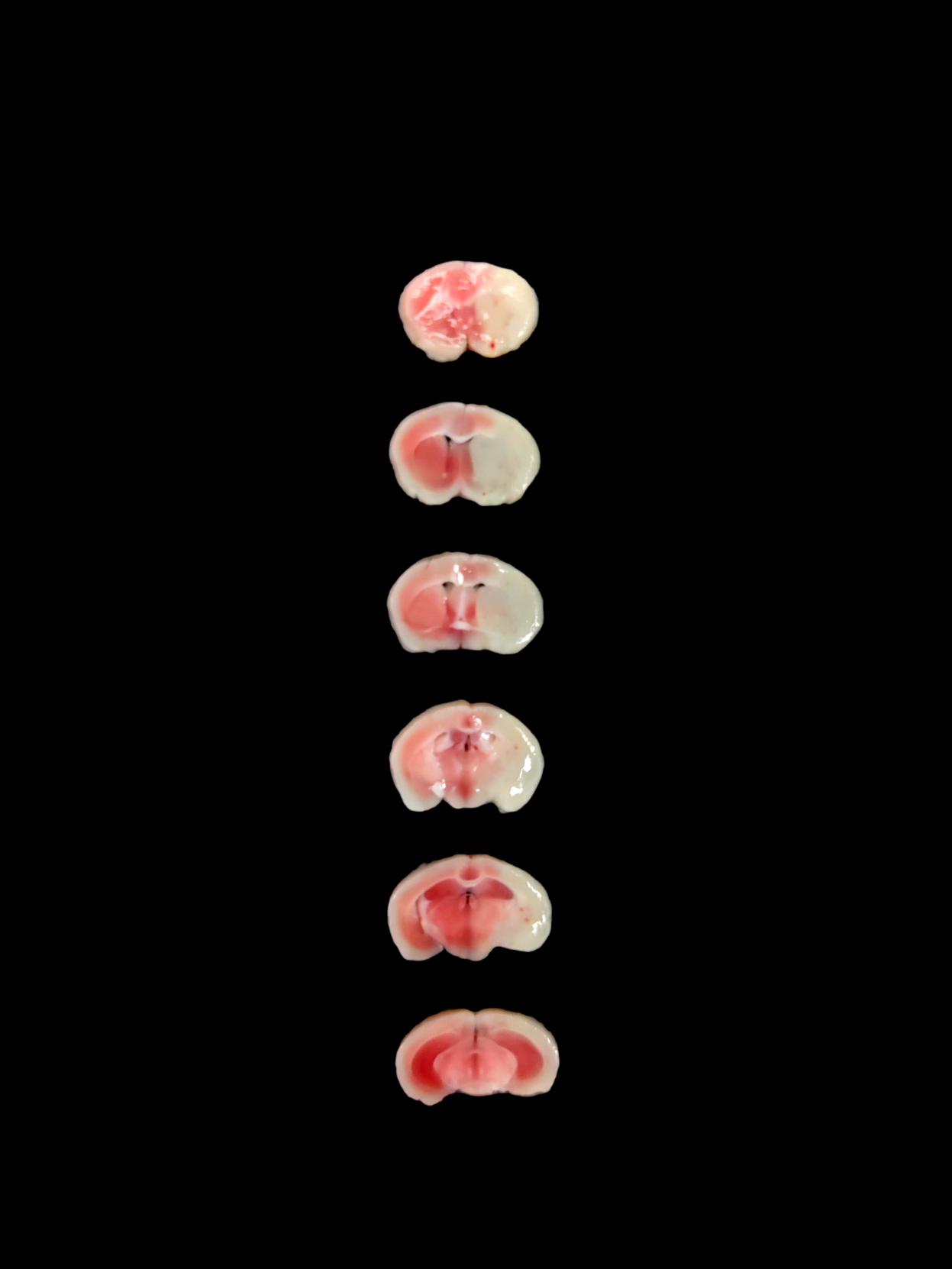

Supplement: Supplementary file 3 — Source data Fig. 1 [file 44321_2025_206_MOESM3_ESM.zip › Source Data Fig 1/Fig 1/1E/WT-D3.tif]

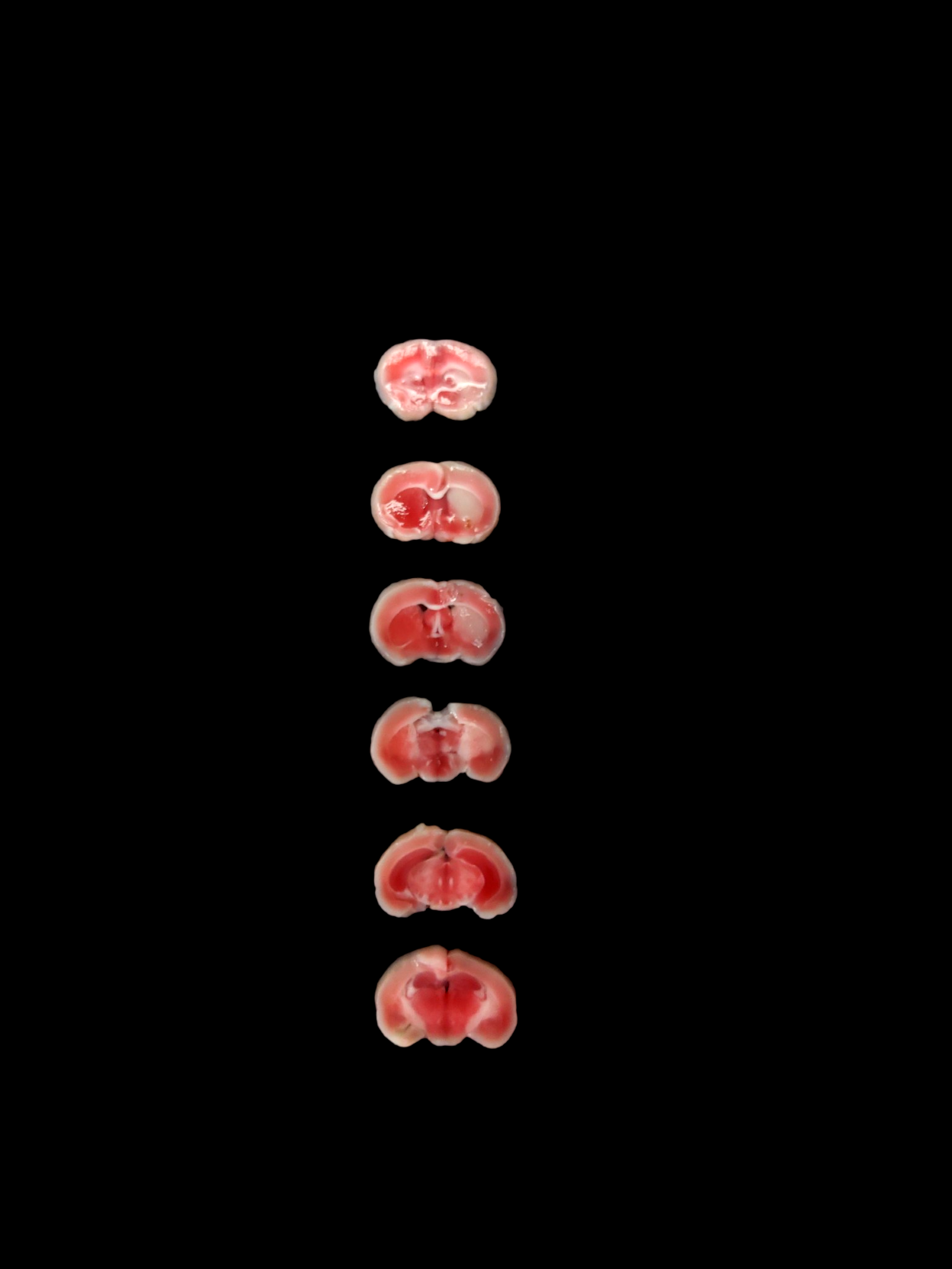

Supplement: Supplementary file 3 — Source data Fig. 1 [file 44321_2025_206_MOESM3_ESM.zip › Source Data Fig 1/Fig 1/1E/WT-D7.tif]

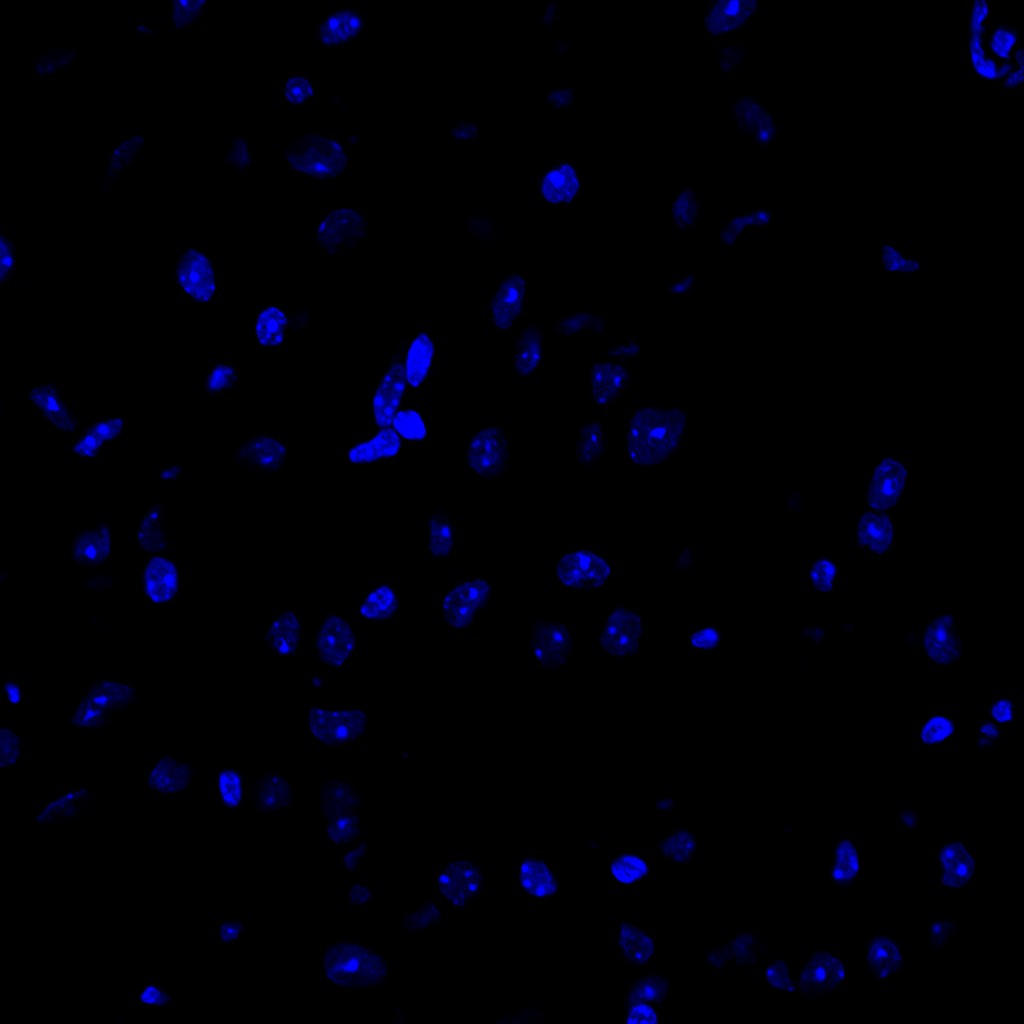

Supplement: Supplementary file 4 — Source data Fig. 2 [file 44321_2025_206_MOESM4_ESM.zip › Source Data Fig 2/Fig 2/2A/DAPI.tif]

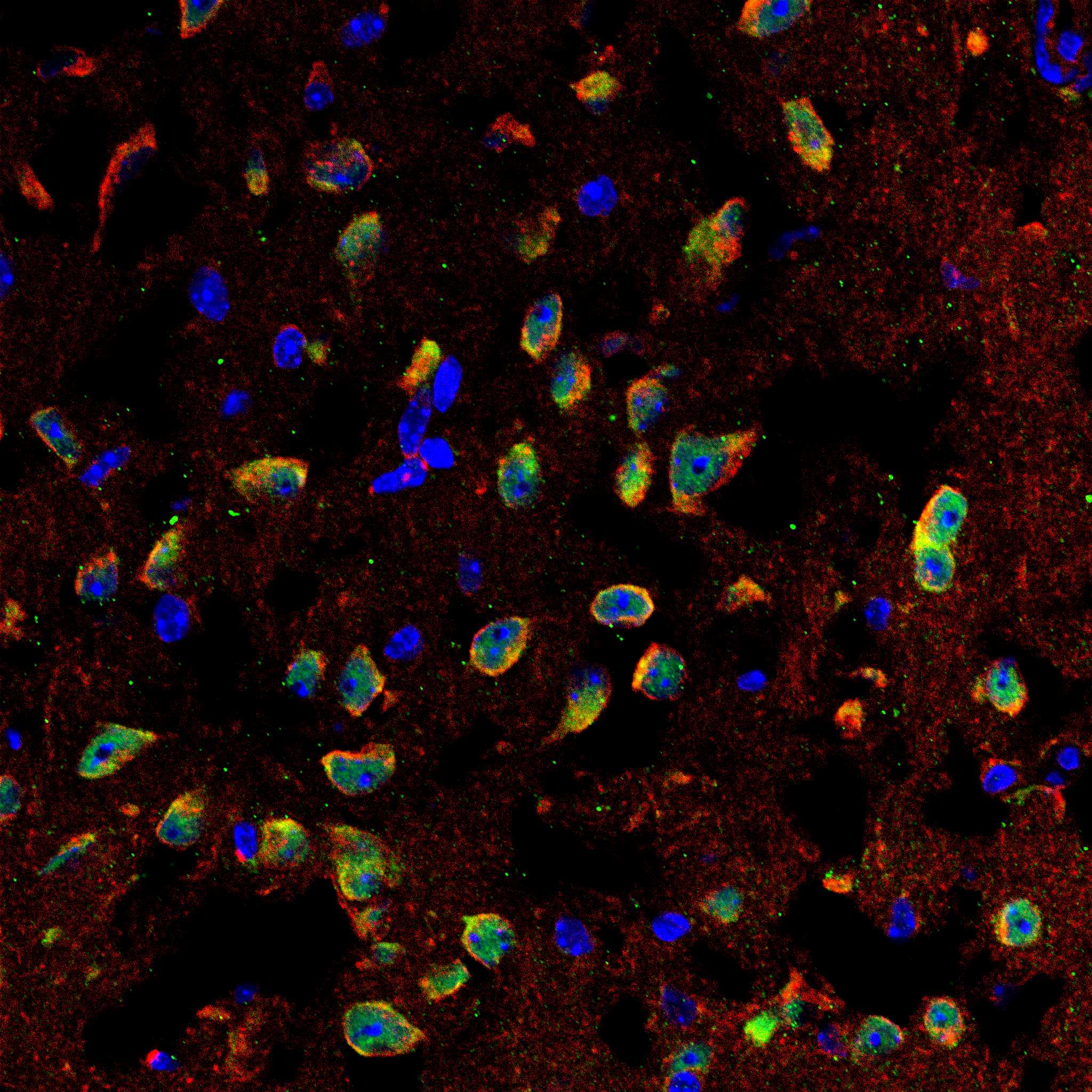

Supplement: Supplementary file 4 — Source data Fig. 2 [file 44321_2025_206_MOESM4_ESM.zip › Source Data Fig 2/Fig 2/2A/MERGE.jpg]

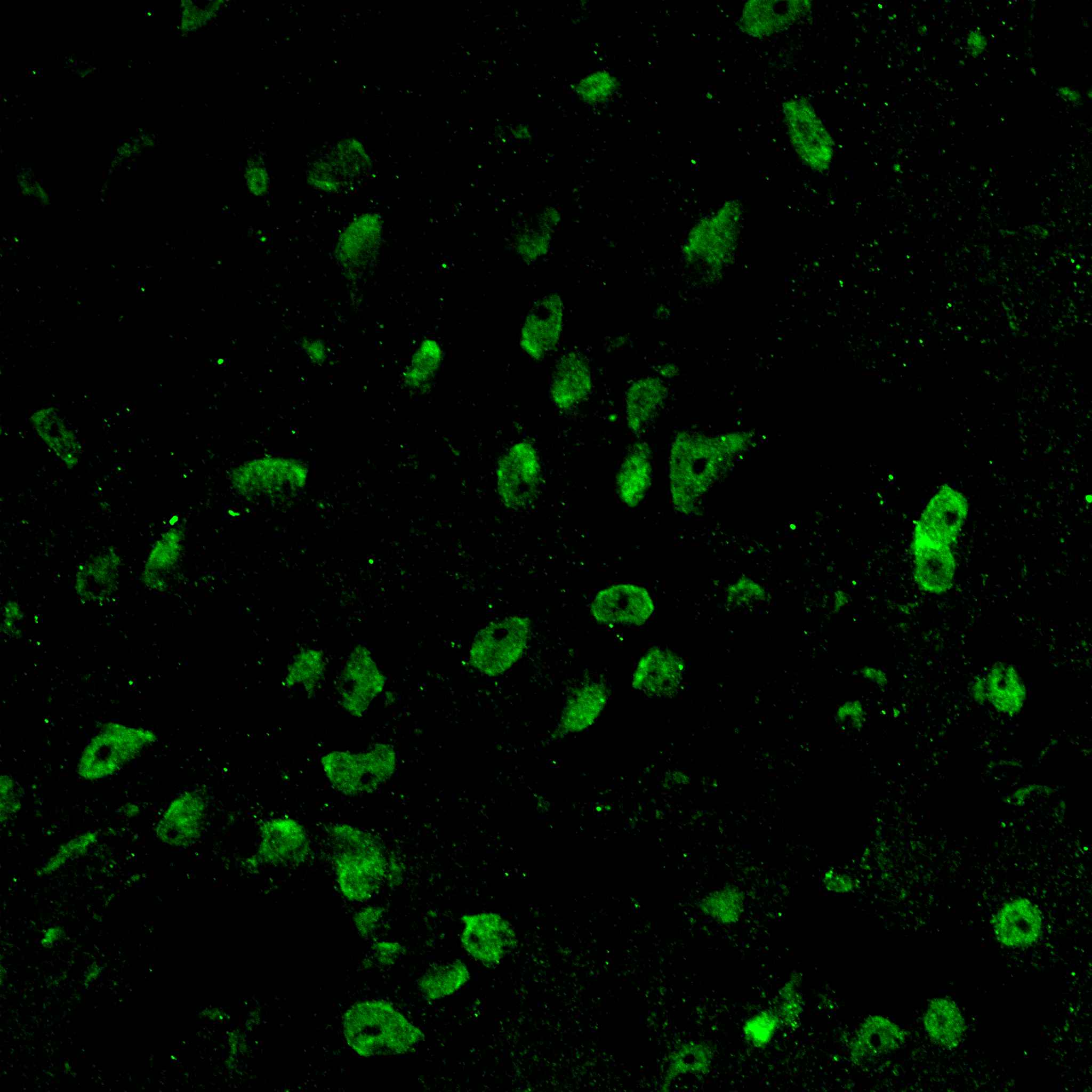

Supplement: Supplementary file 4 — Source data Fig. 2 [file 44321_2025_206_MOESM4_ESM.zip › Source Data Fig 2/Fig 2/2A/NEUN.tif]

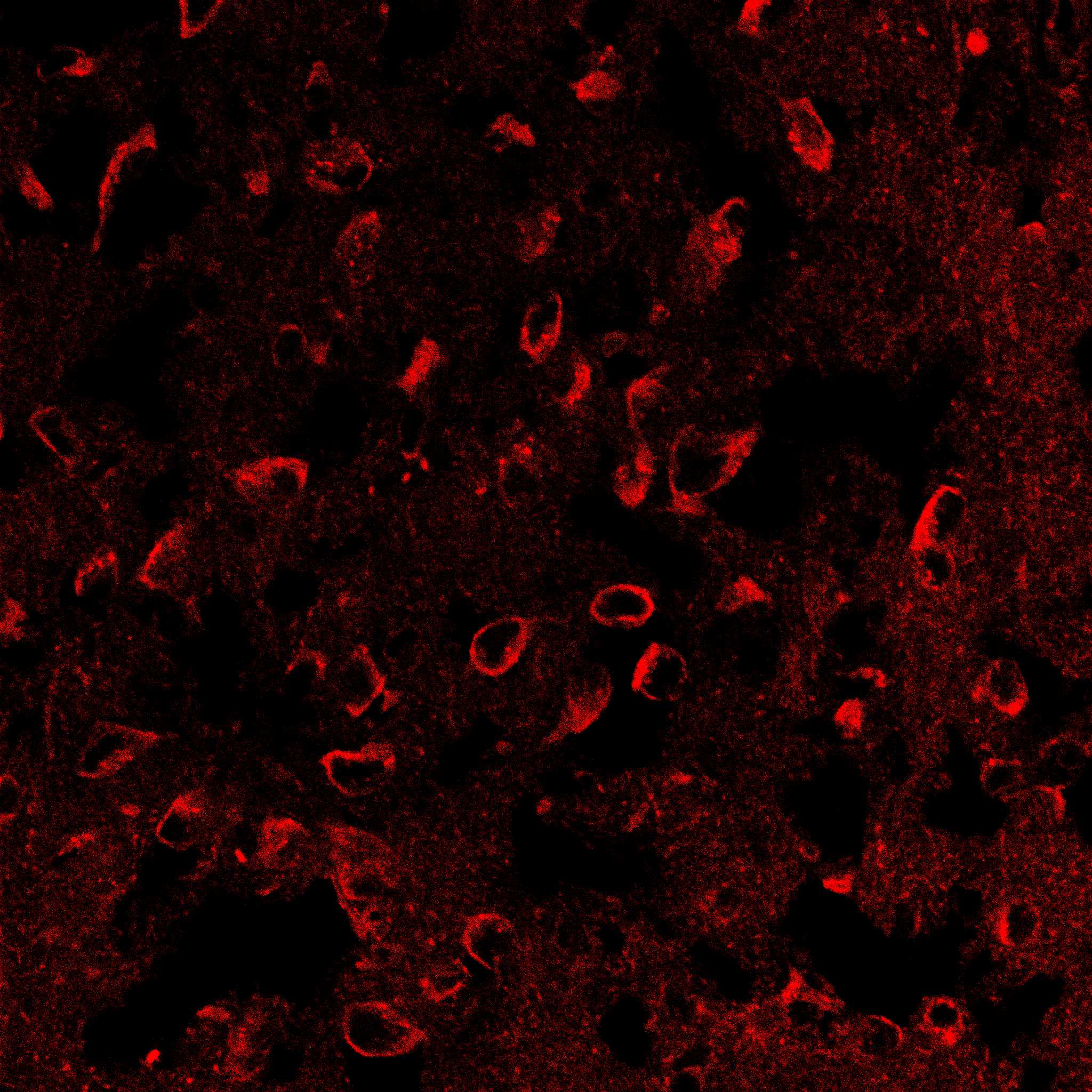

Supplement: Supplementary file 4 — Source data Fig. 2 [file 44321_2025_206_MOESM4_ESM.zip › Source Data Fig 2/Fig 2/2A/OTUB2.tif]

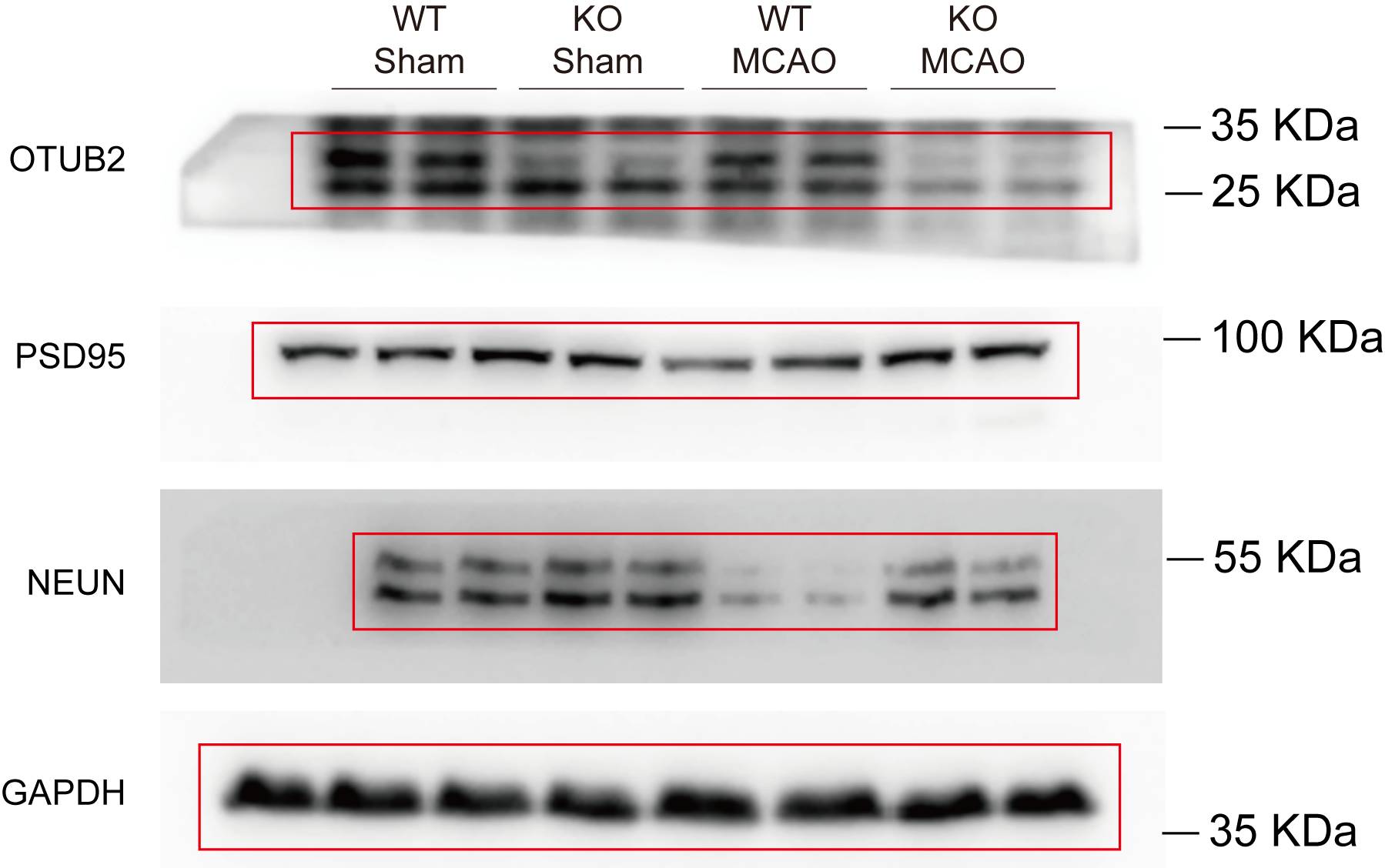

Supplement: Supplementary file 4 — Source data Fig. 2 [file 44321_2025_206_MOESM4_ESM.zip › Source Data Fig 2/Fig 2/2B/2B.tif]

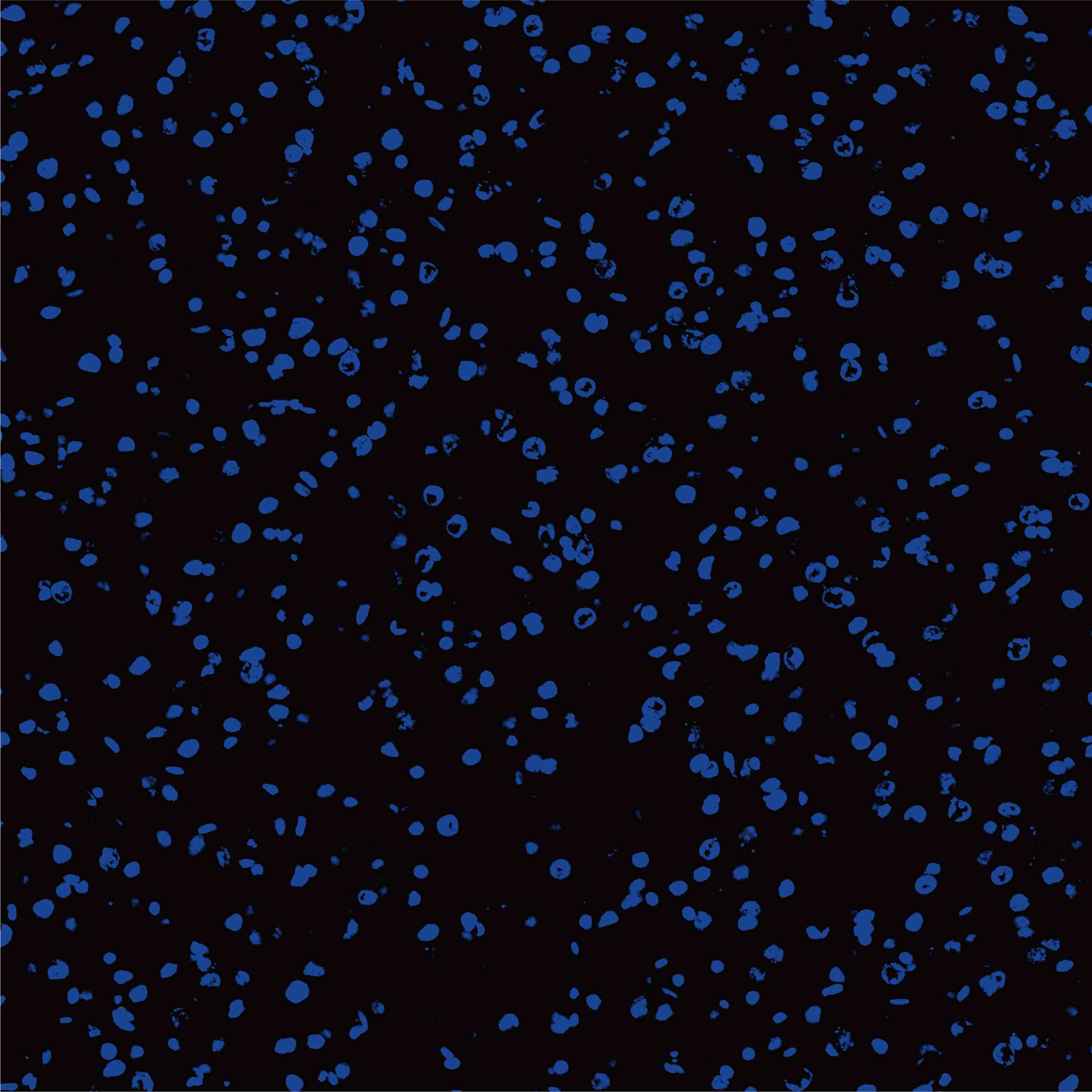

Supplement: Supplementary file 4 — Source data Fig. 2 [file 44321_2025_206_MOESM4_ESM.zip › Source Data Fig 2/Fig 2/2E/KO-MCAO-DAPI.tif]

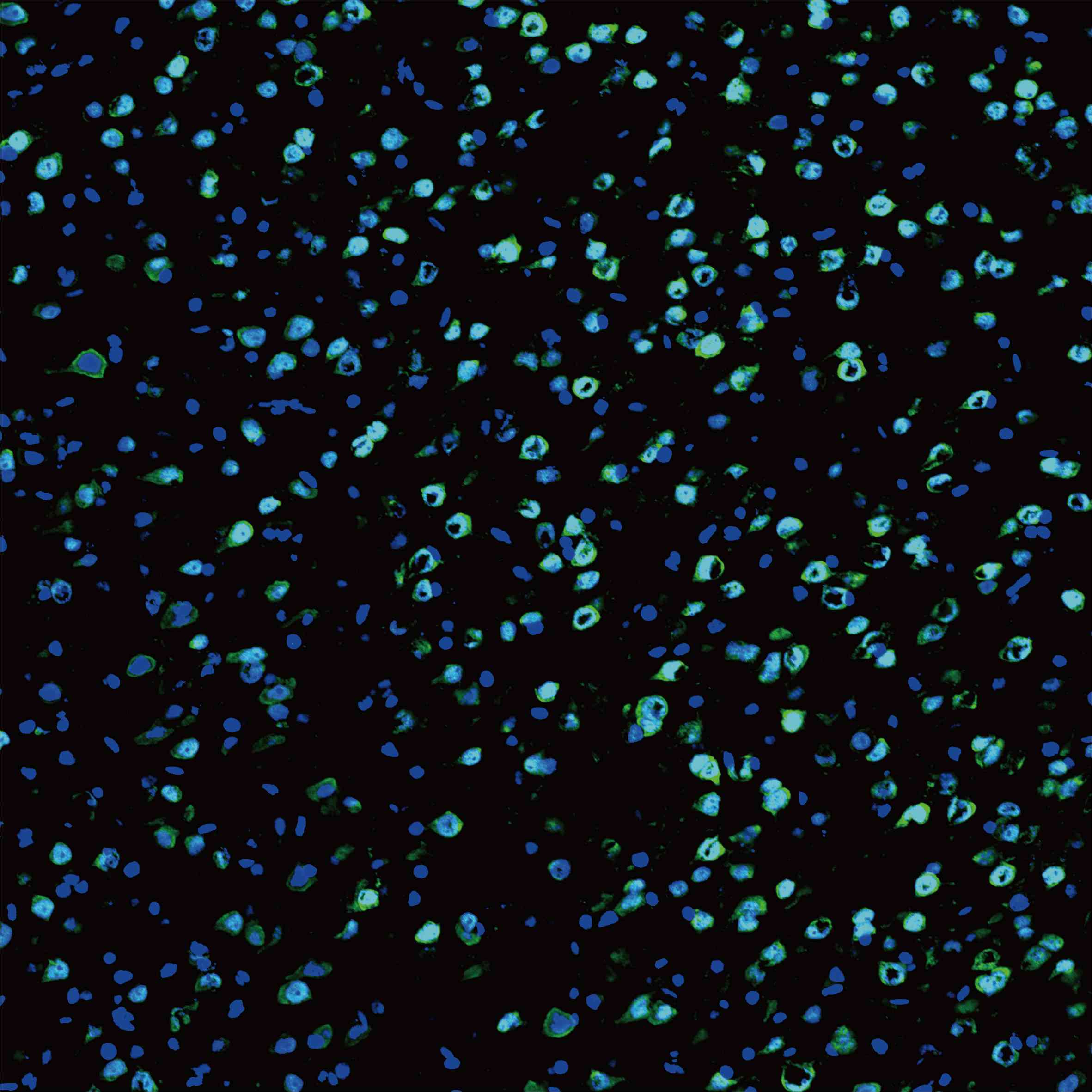

Supplement: Supplementary file 4 — Source data Fig. 2 [file 44321_2025_206_MOESM4_ESM.zip › Source Data Fig 2/Fig 2/2E/KO-MCAO-Merge.tif]

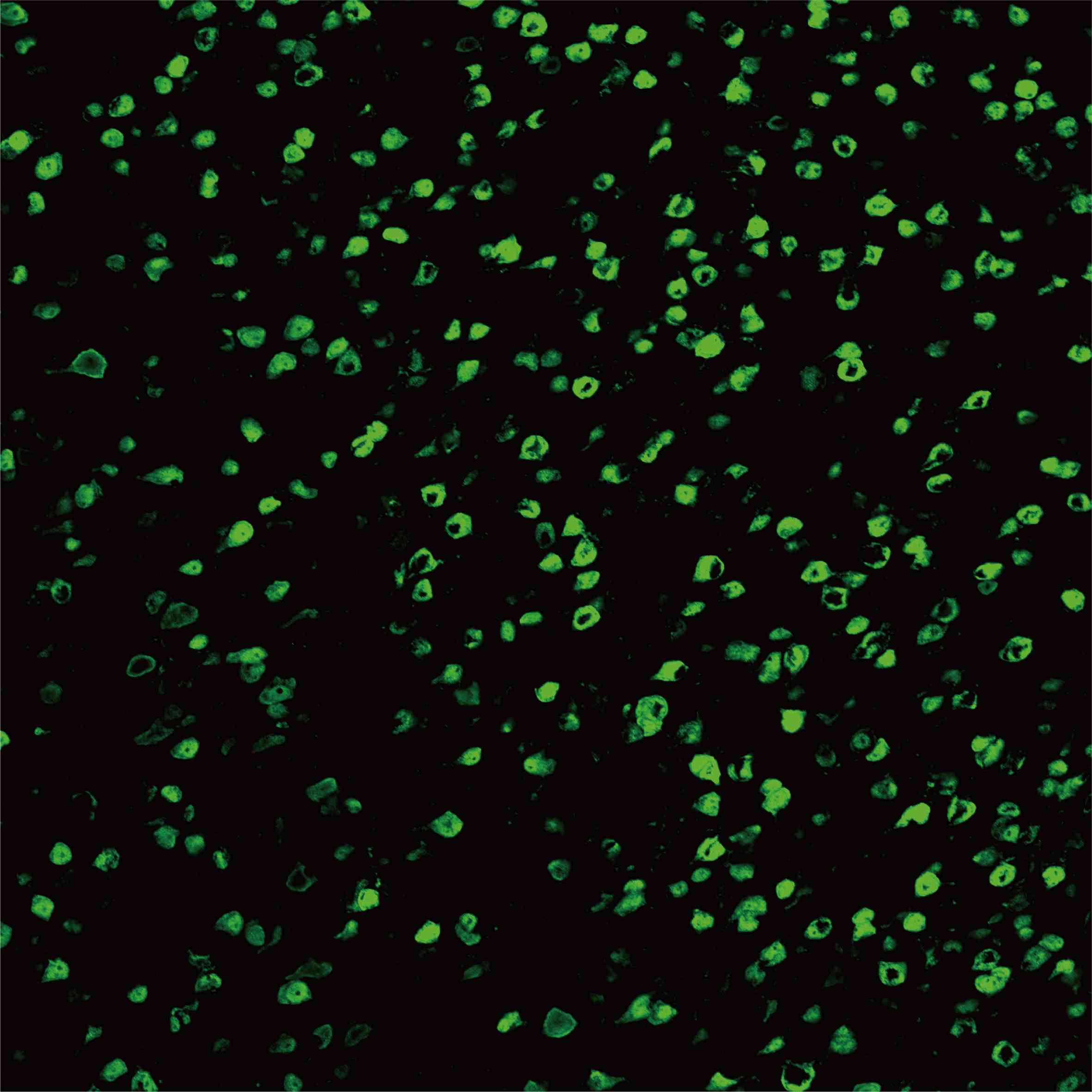

Supplement: Supplementary file 4 — Source data Fig. 2 [file 44321_2025_206_MOESM4_ESM.zip › Source Data Fig 2/Fig 2/2E/KO-MCAO-NEUN.tif]

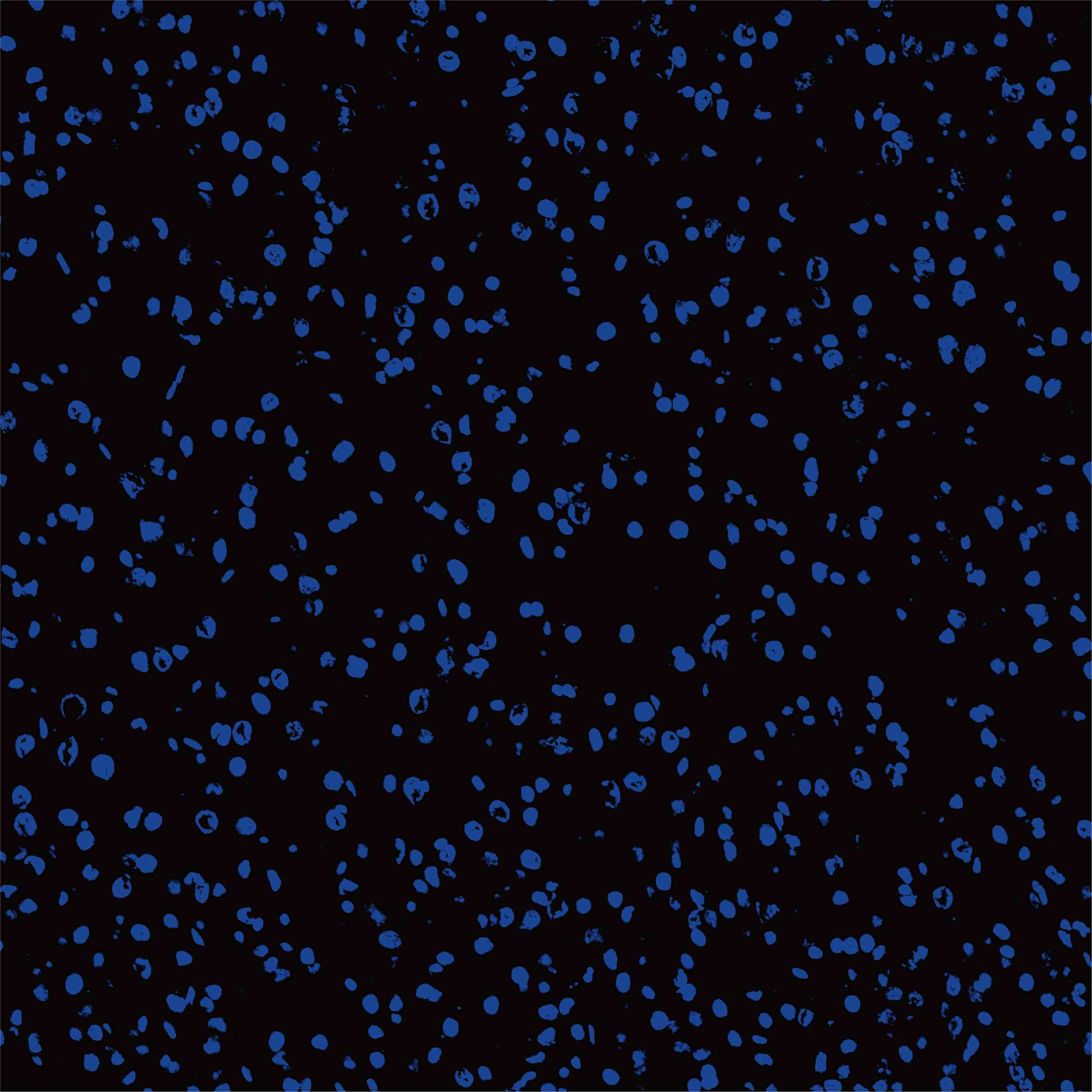

Supplement: Supplementary file 4 — Source data Fig. 2 [file 44321_2025_206_MOESM4_ESM.zip › Source Data Fig 2/Fig 2/2E/KO-Sham-DAPI.tif]

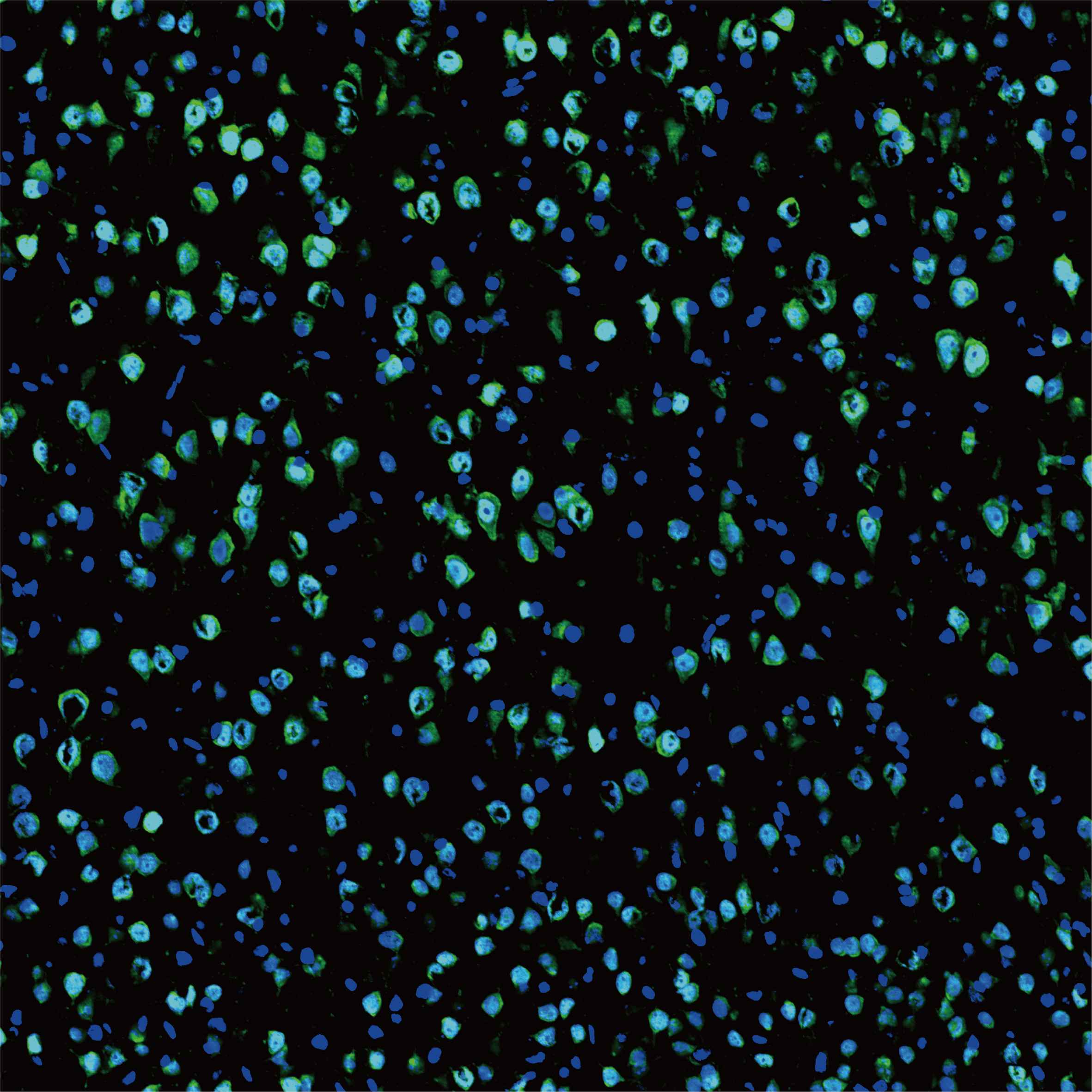

Supplement: Supplementary file 4 — Source data Fig. 2 [file 44321_2025_206_MOESM4_ESM.zip › Source Data Fig 2/Fig 2/2E/KO-Sham-Merge.tif]

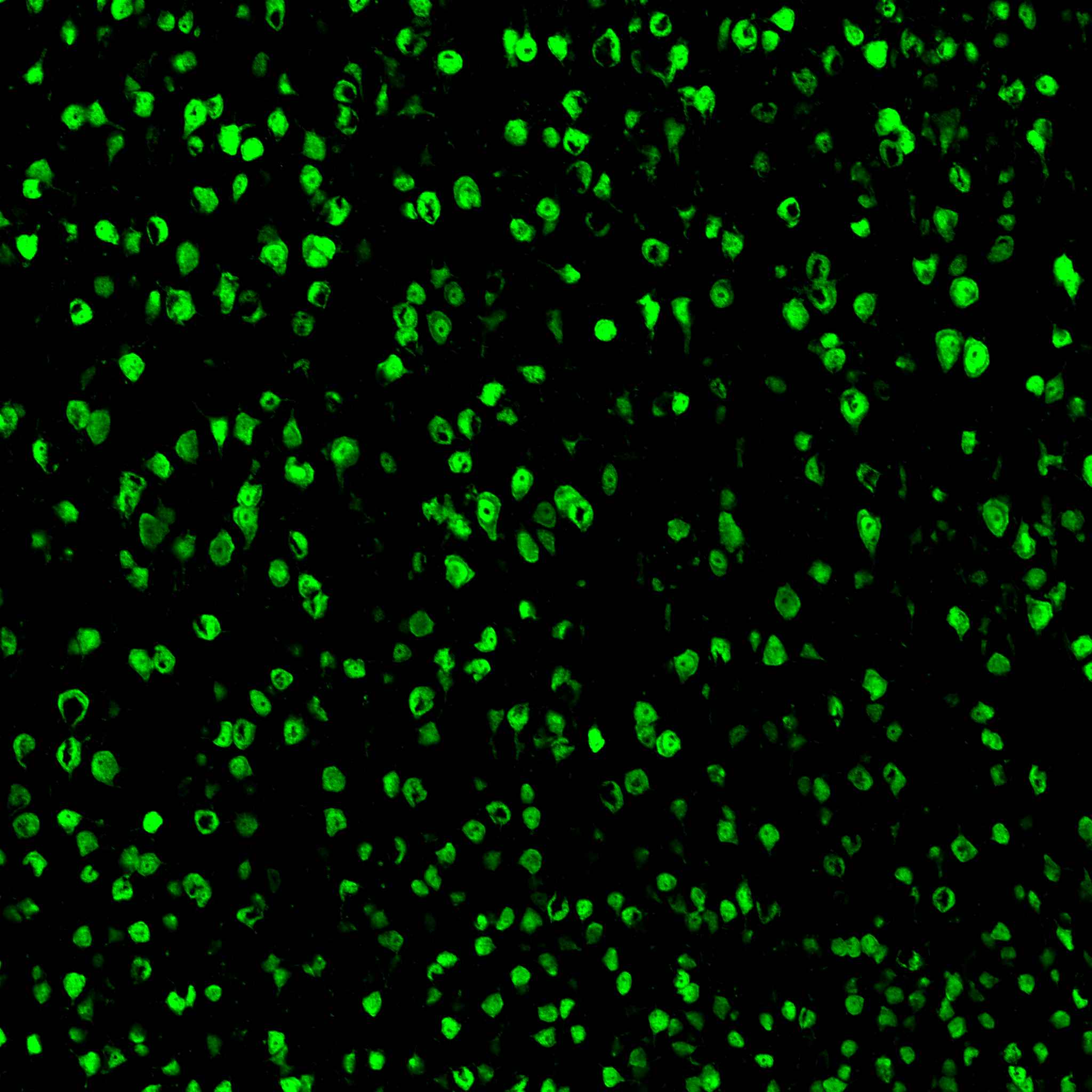

Supplement: Supplementary file 4 — Source data Fig. 2 [file 44321_2025_206_MOESM4_ESM.zip › Source Data Fig 2/Fig 2/2E/KO-Sham-NEUN.tif]

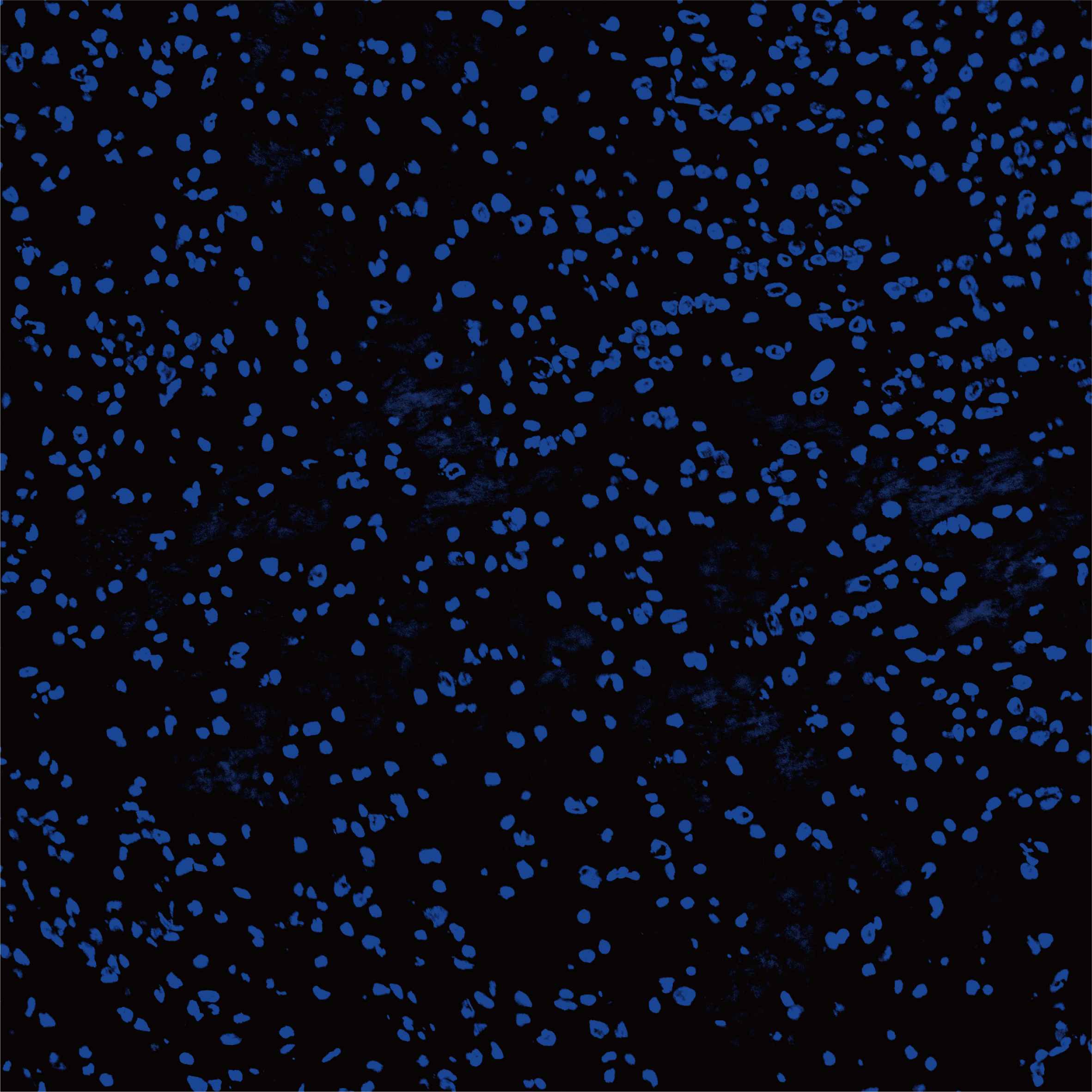

Supplement: Supplementary file 4 — Source data Fig. 2 [file 44321_2025_206_MOESM4_ESM.zip › Source Data Fig 2/Fig 2/2E/WT-MCAO-DAPI.tif]

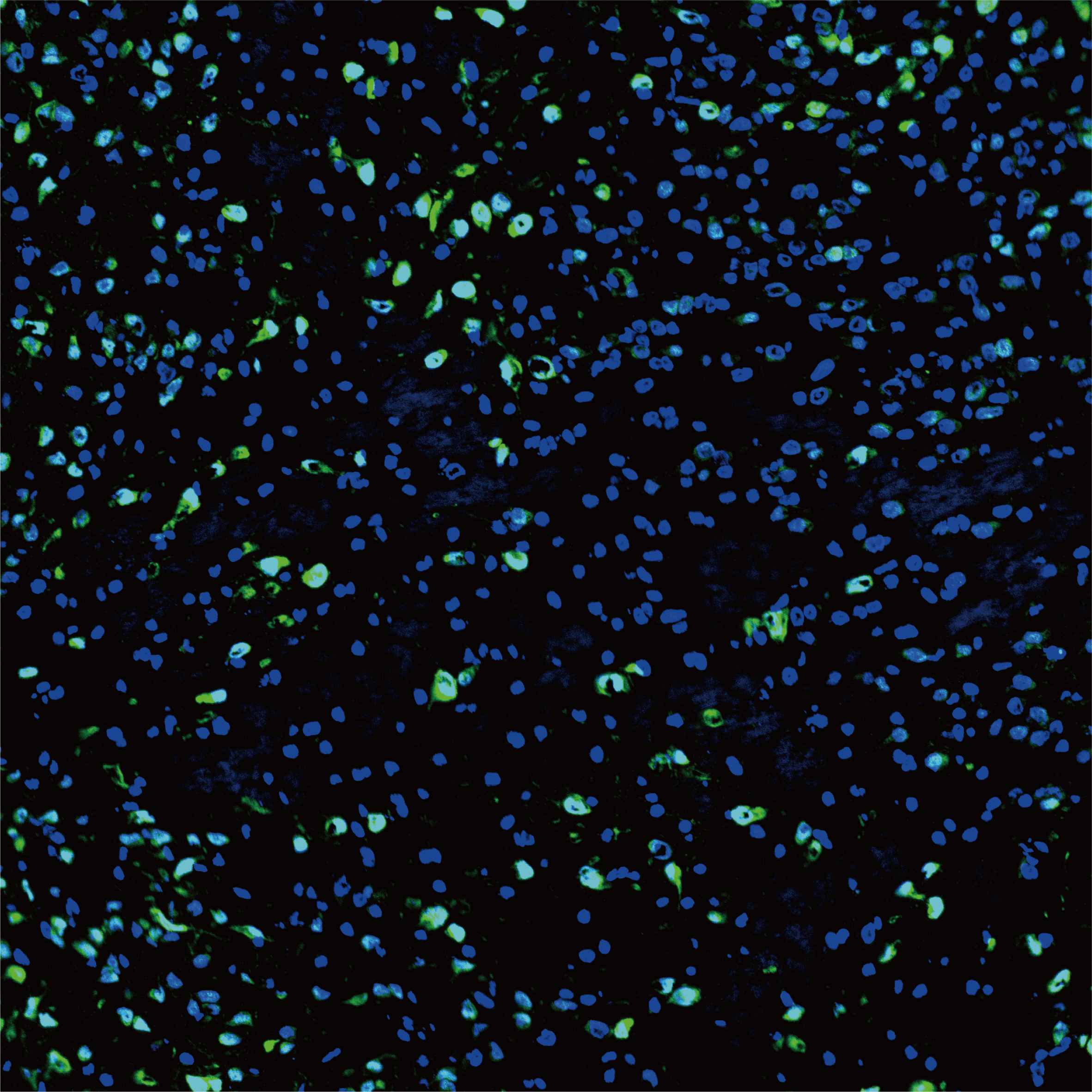

Supplement: Supplementary file 4 — Source data Fig. 2 [file 44321_2025_206_MOESM4_ESM.zip › Source Data Fig 2/Fig 2/2E/WT-MCAO-Merge.tif]

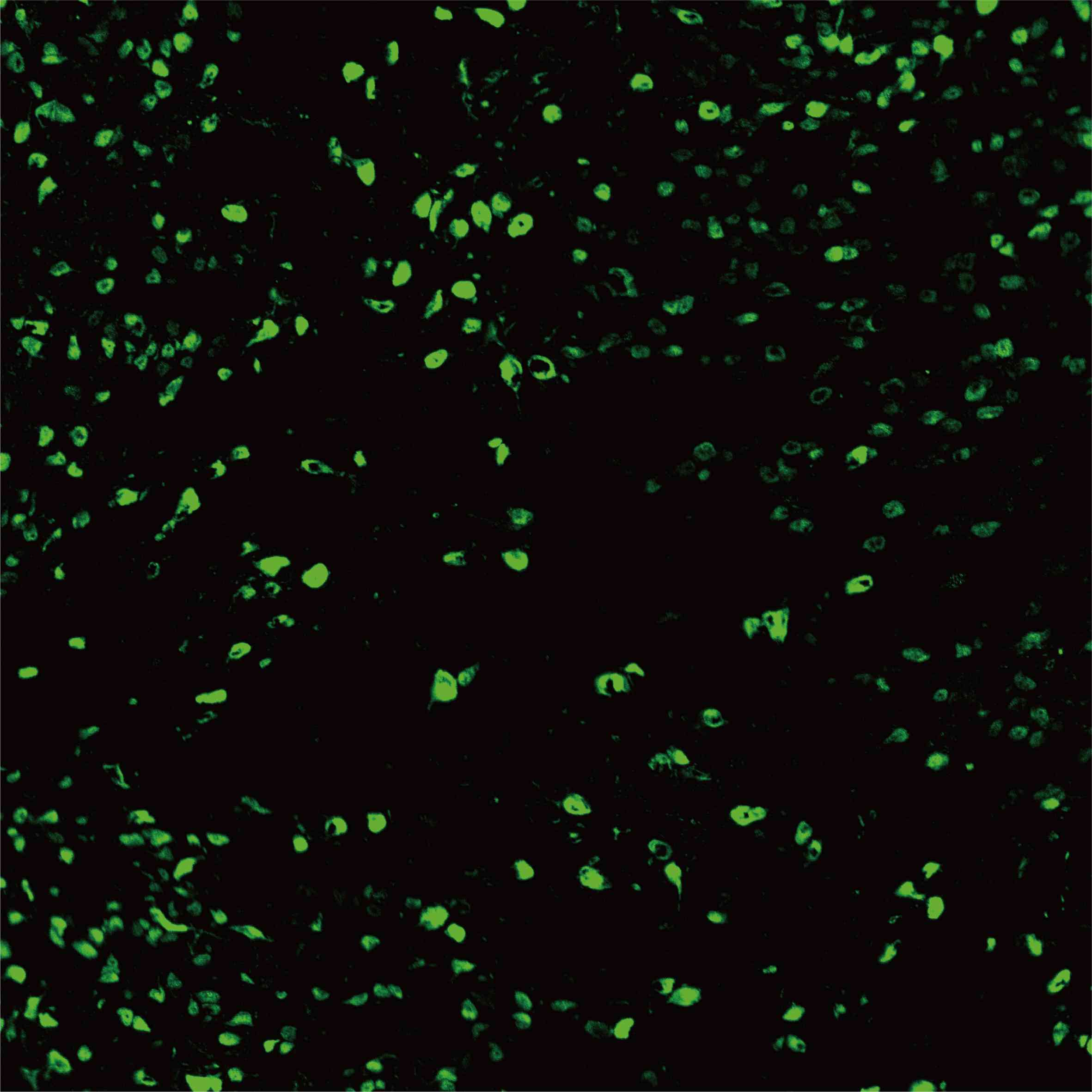

Supplement: Supplementary file 4 — Source data Fig. 2 [file 44321_2025_206_MOESM4_ESM.zip › Source Data Fig 2/Fig 2/2E/WT-MCAO-NEUN.tif]

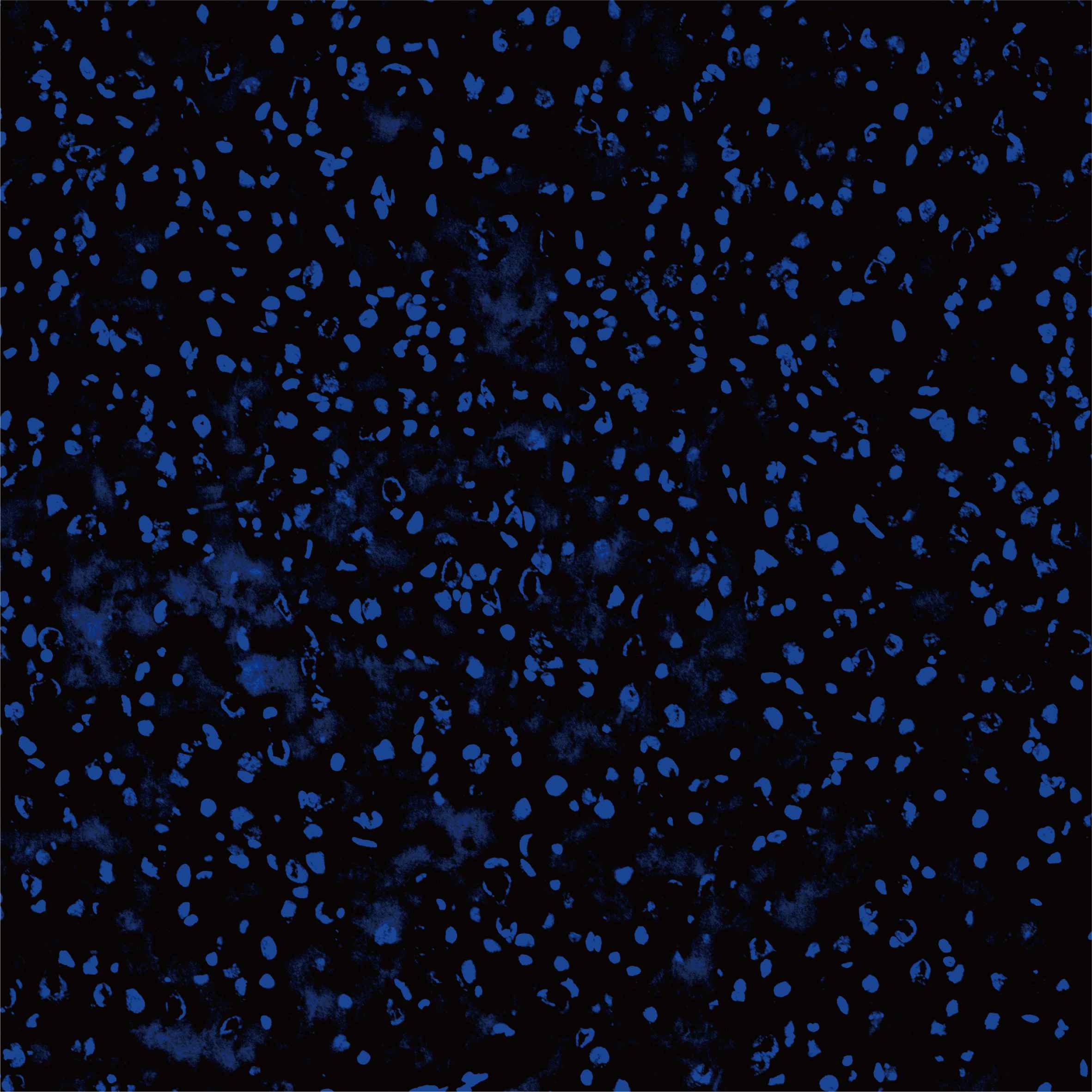

Supplement: Supplementary file 4 — Source data Fig. 2 [file 44321_2025_206_MOESM4_ESM.zip › Source Data Fig 2/Fig 2/2E/WT-Sham-DAPI.tif]

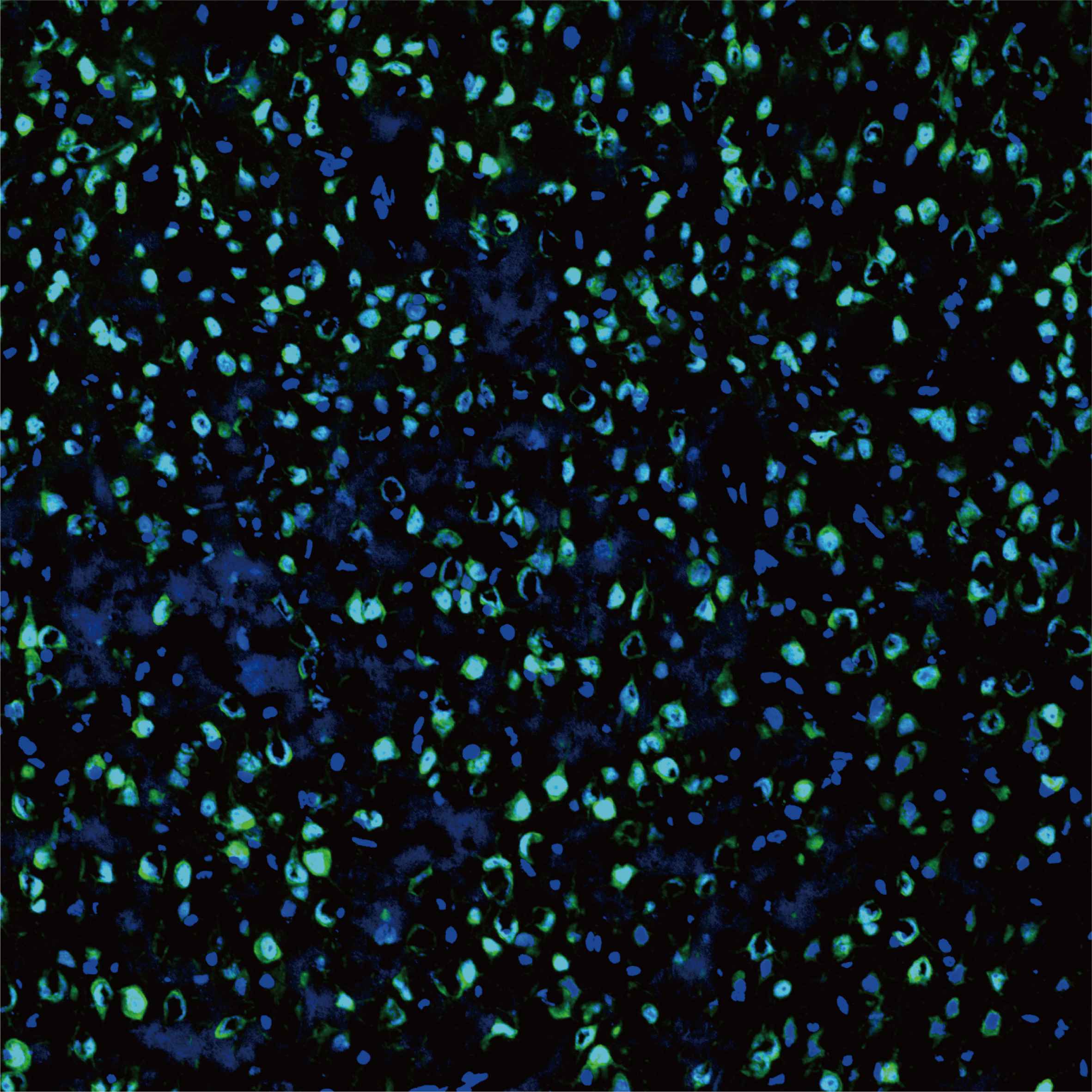

Supplement: Supplementary file 4 — Source data Fig. 2 [file 44321_2025_206_MOESM4_ESM.zip › Source Data Fig 2/Fig 2/2E/WT-Sham-Merge.tif]

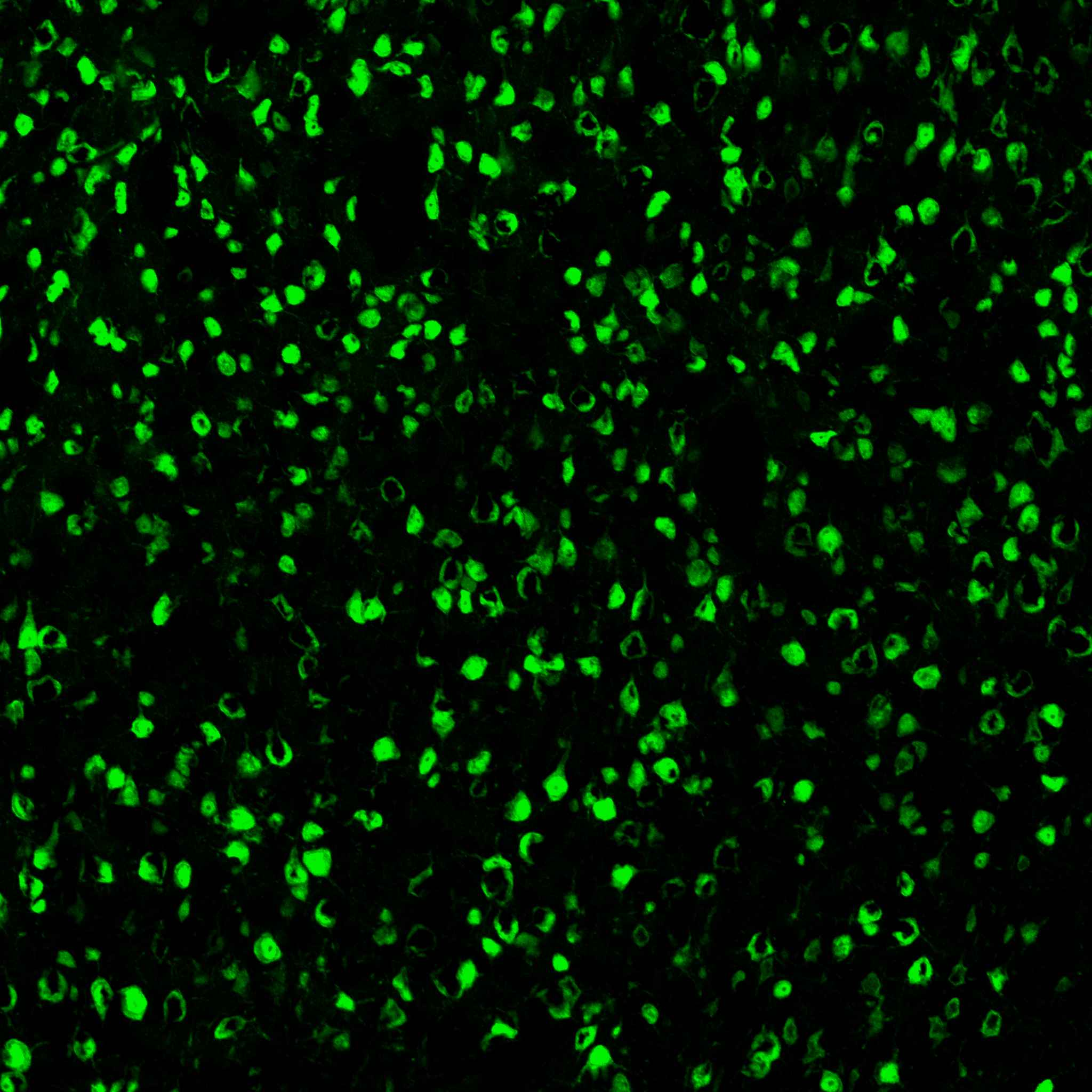

Supplement: Supplementary file 4 — Source data Fig. 2 [file 44321_2025_206_MOESM4_ESM.zip › Source Data Fig 2/Fig 2/2E/WT-Sham-NEUN.tif]

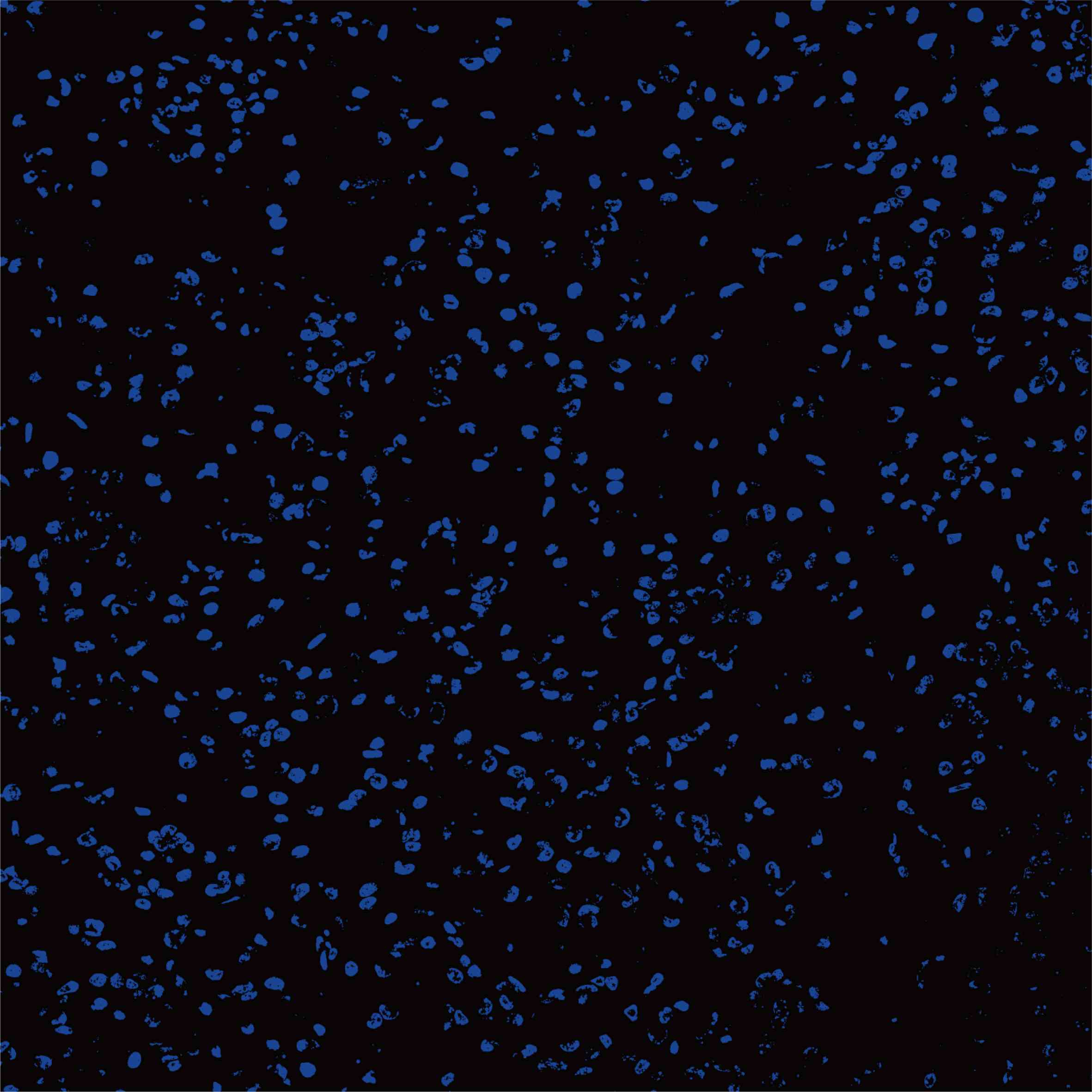

Supplement: Supplementary file 4 — Source data Fig. 2 [file 44321_2025_206_MOESM4_ESM.zip › Source Data Fig 2/Fig 2/2G/KO-MCAO DAPI.tif]

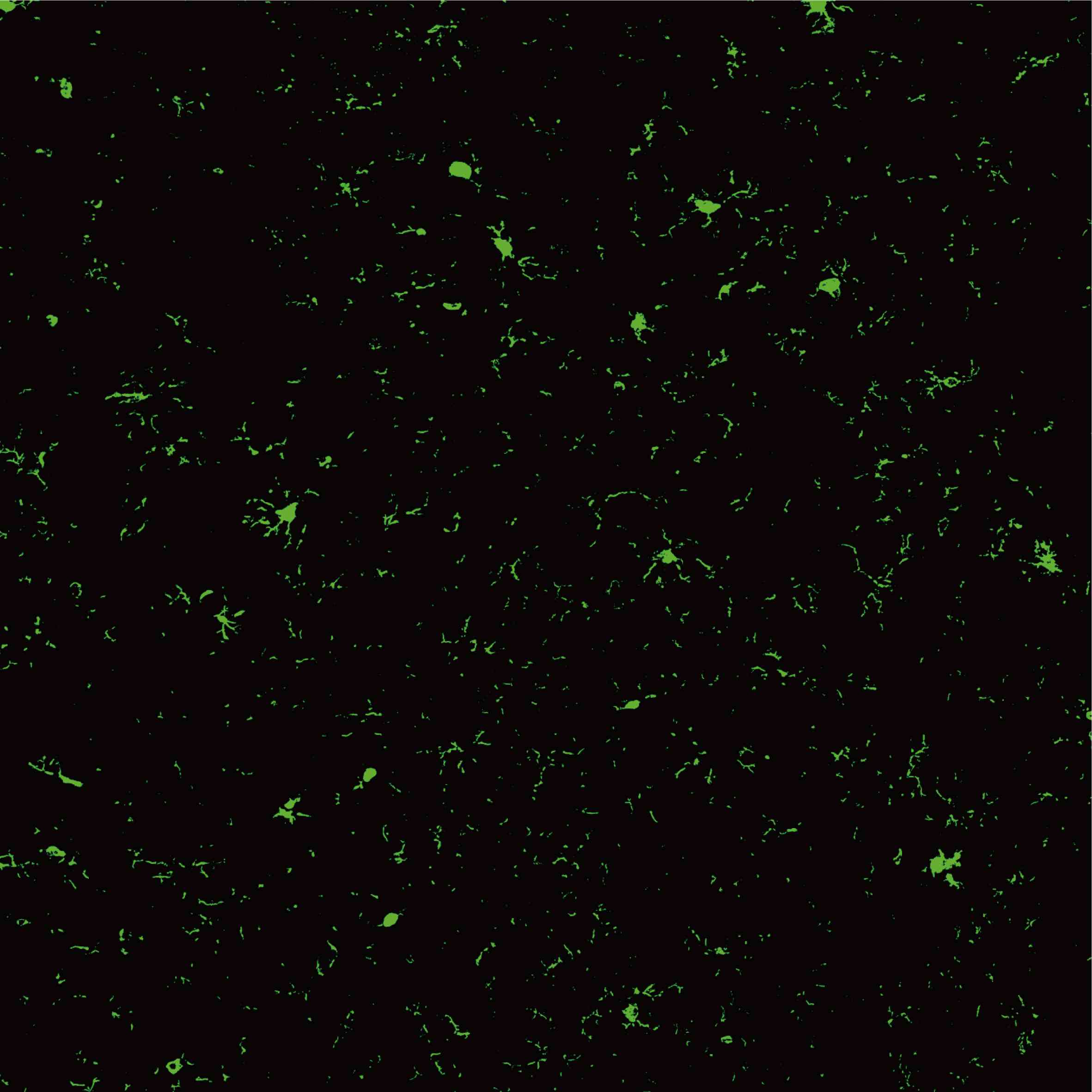

Supplement: Supplementary file 4 — Source data Fig. 2 [file 44321_2025_206_MOESM4_ESM.zip › Source Data Fig 2/Fig 2/2G/KO-MCAO IBA-1.tif]

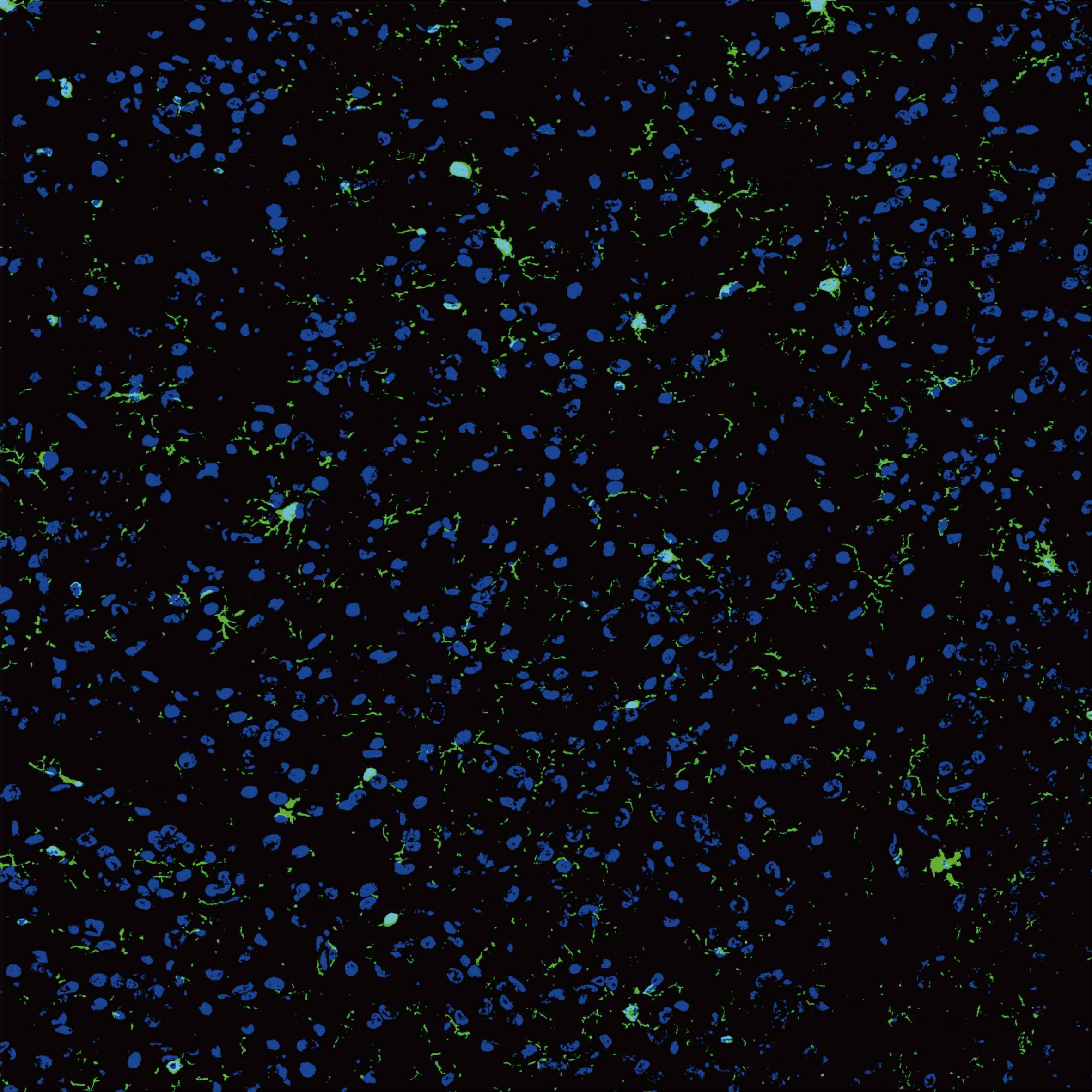

Supplement: Supplementary file 4 — Source data Fig. 2 [file 44321_2025_206_MOESM4_ESM.zip › Source Data Fig 2/Fig 2/2G/KO-MCAO Merge.tif]

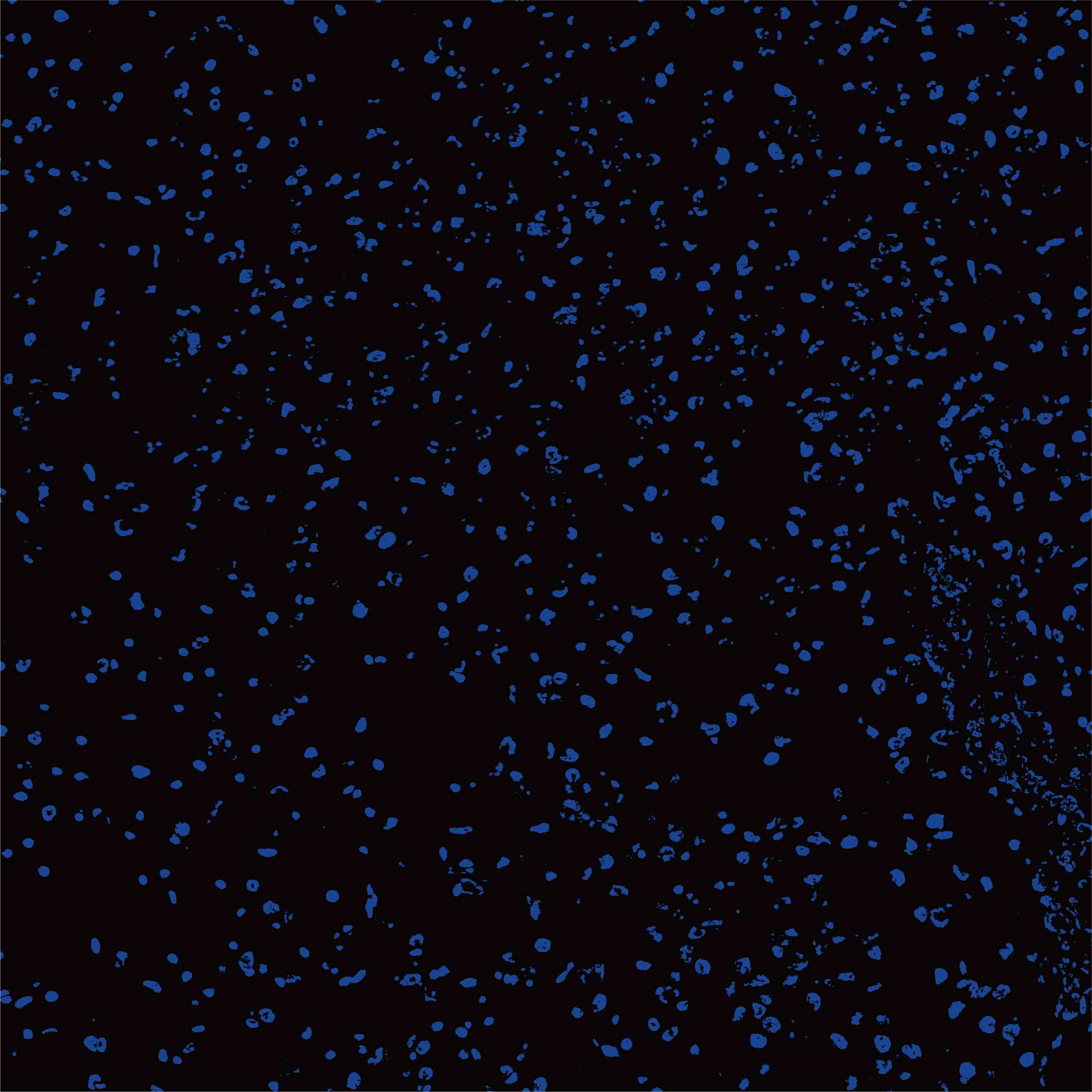

Supplement: Supplementary file 4 — Source data Fig. 2 [file 44321_2025_206_MOESM4_ESM.zip › Source Data Fig 2/Fig 2/2G/KO-Sham DAPI.tif]

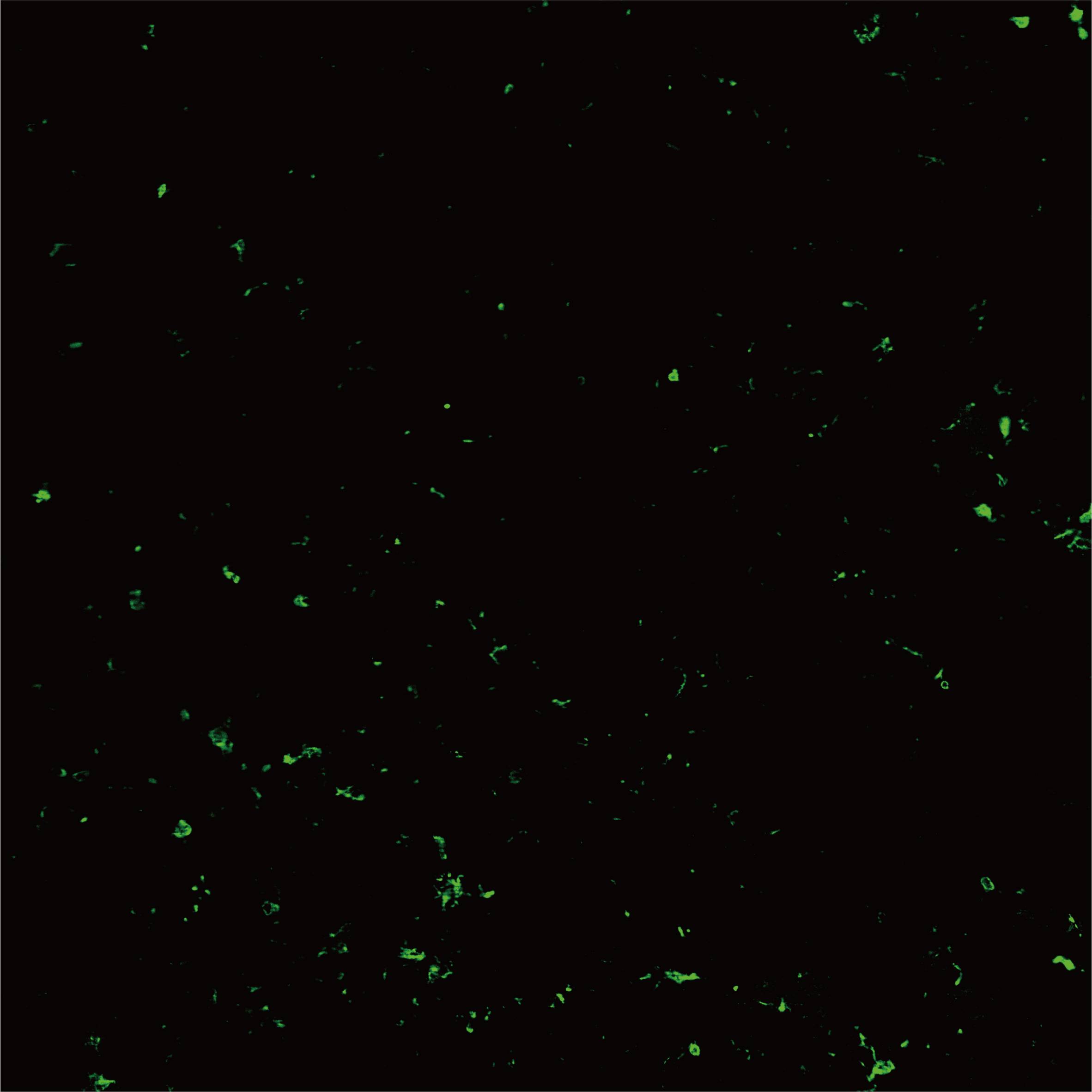

Supplement: Supplementary file 4 — Source data Fig. 2 [file 44321_2025_206_MOESM4_ESM.zip › Source Data Fig 2/Fig 2/2G/KO-Sham IBA-1.tif]

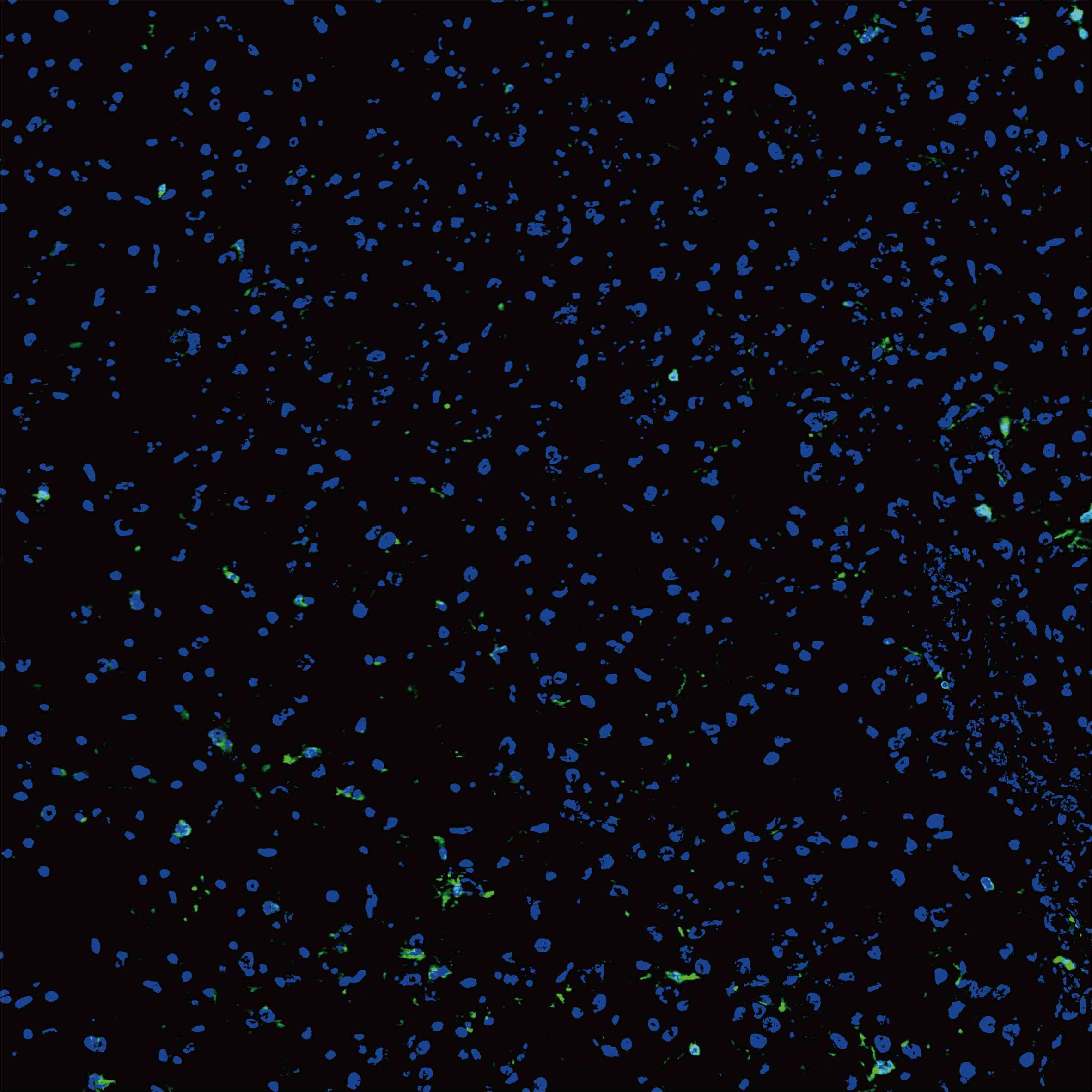

Supplement: Supplementary file 4 — Source data Fig. 2 [file 44321_2025_206_MOESM4_ESM.zip › Source Data Fig 2/Fig 2/2G/KO-Sham Merge.tif]

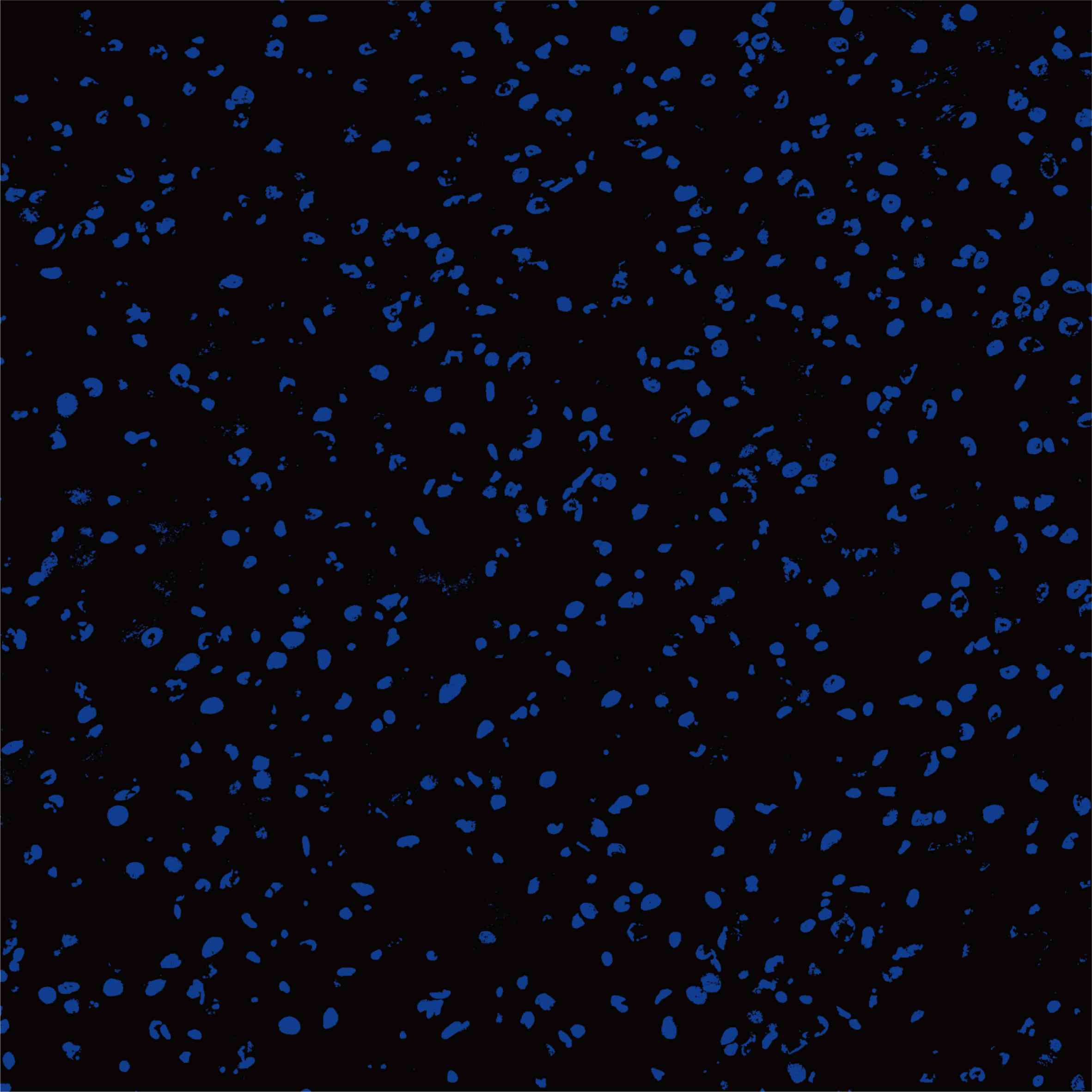

Supplement: Supplementary file 4 — Source data Fig. 2 [file 44321_2025_206_MOESM4_ESM.zip › Source Data Fig 2/Fig 2/2G/WT -MCAO DAPI.tif]

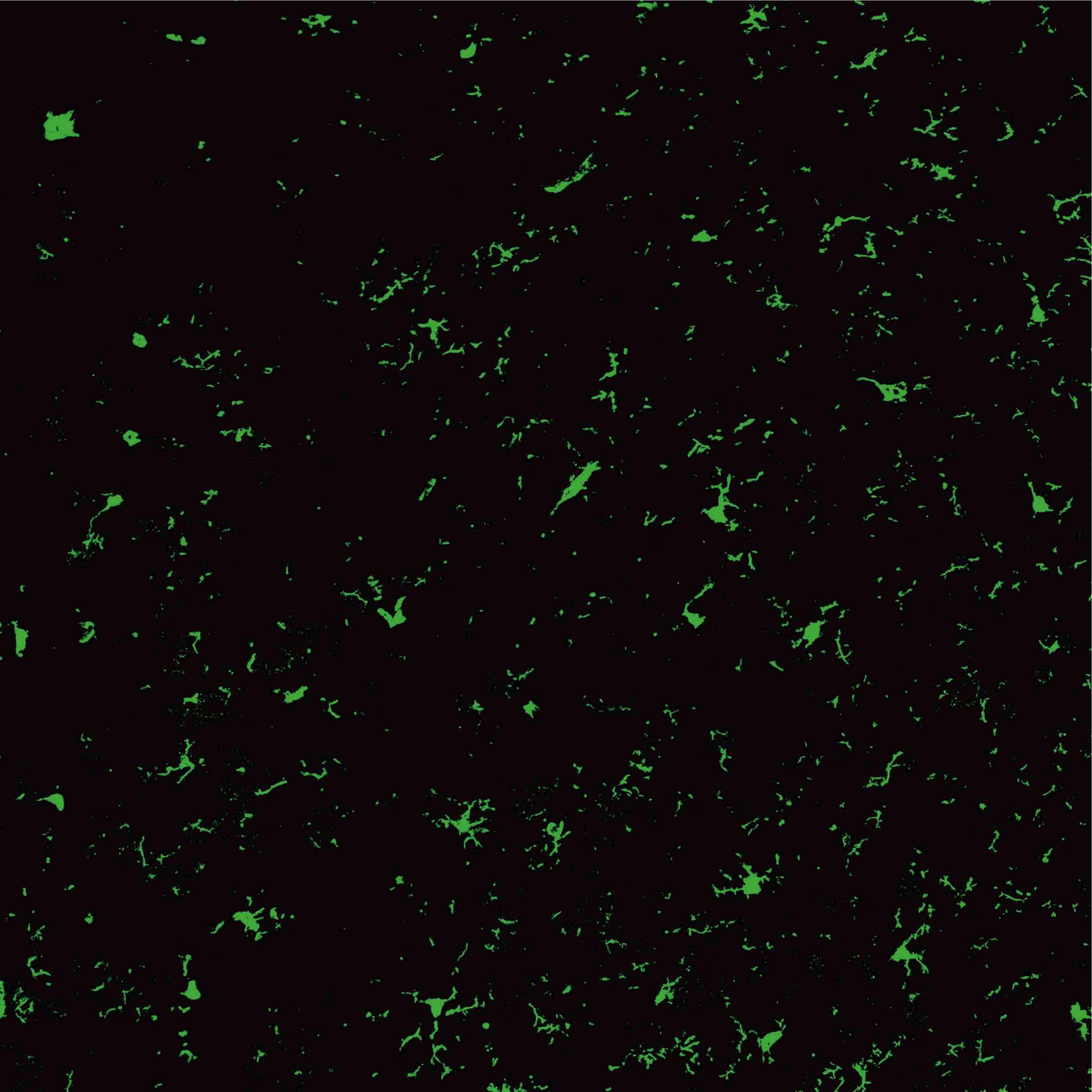

Supplement: Supplementary file 4 — Source data Fig. 2 [file 44321_2025_206_MOESM4_ESM.zip › Source Data Fig 2/Fig 2/2G/WT -MCAO IBA-1.tif]

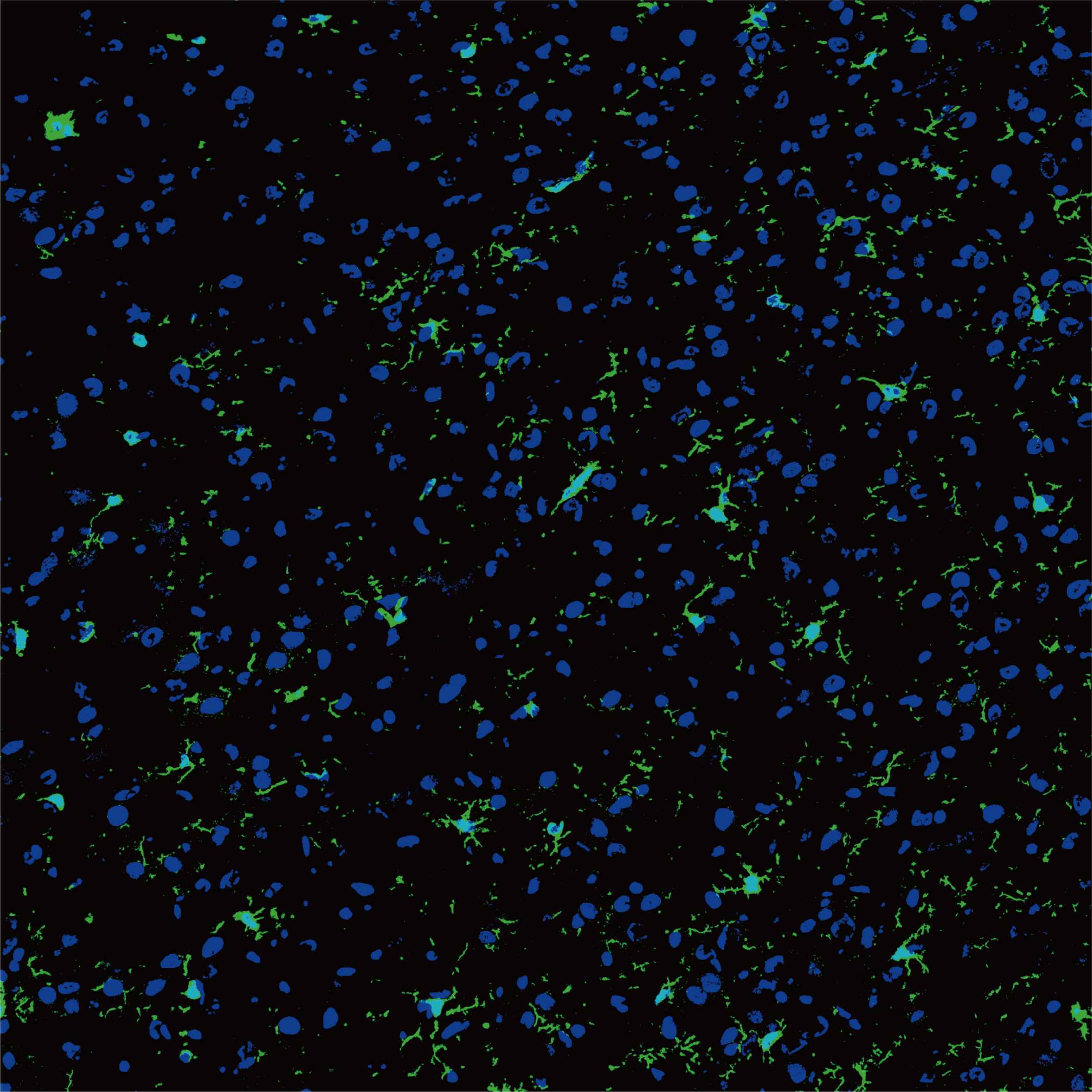

Supplement: Supplementary file 4 — Source data Fig. 2 [file 44321_2025_206_MOESM4_ESM.zip › Source Data Fig 2/Fig 2/2G/WT -MCAO Merge.tif]

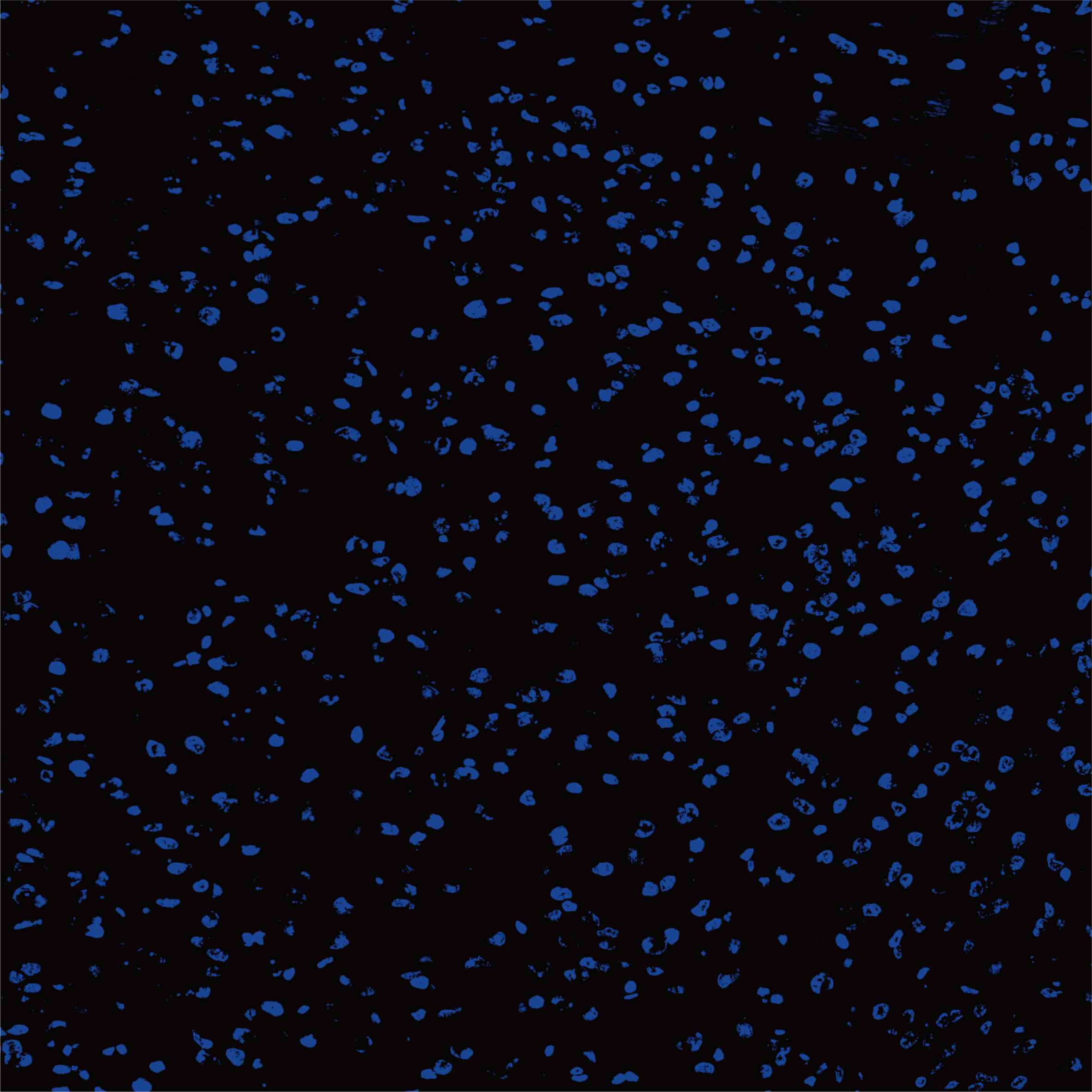

Supplement: Supplementary file 4 — Source data Fig. 2 [file 44321_2025_206_MOESM4_ESM.zip › Source Data Fig 2/Fig 2/2G/WT-Sham DAPI.tif]

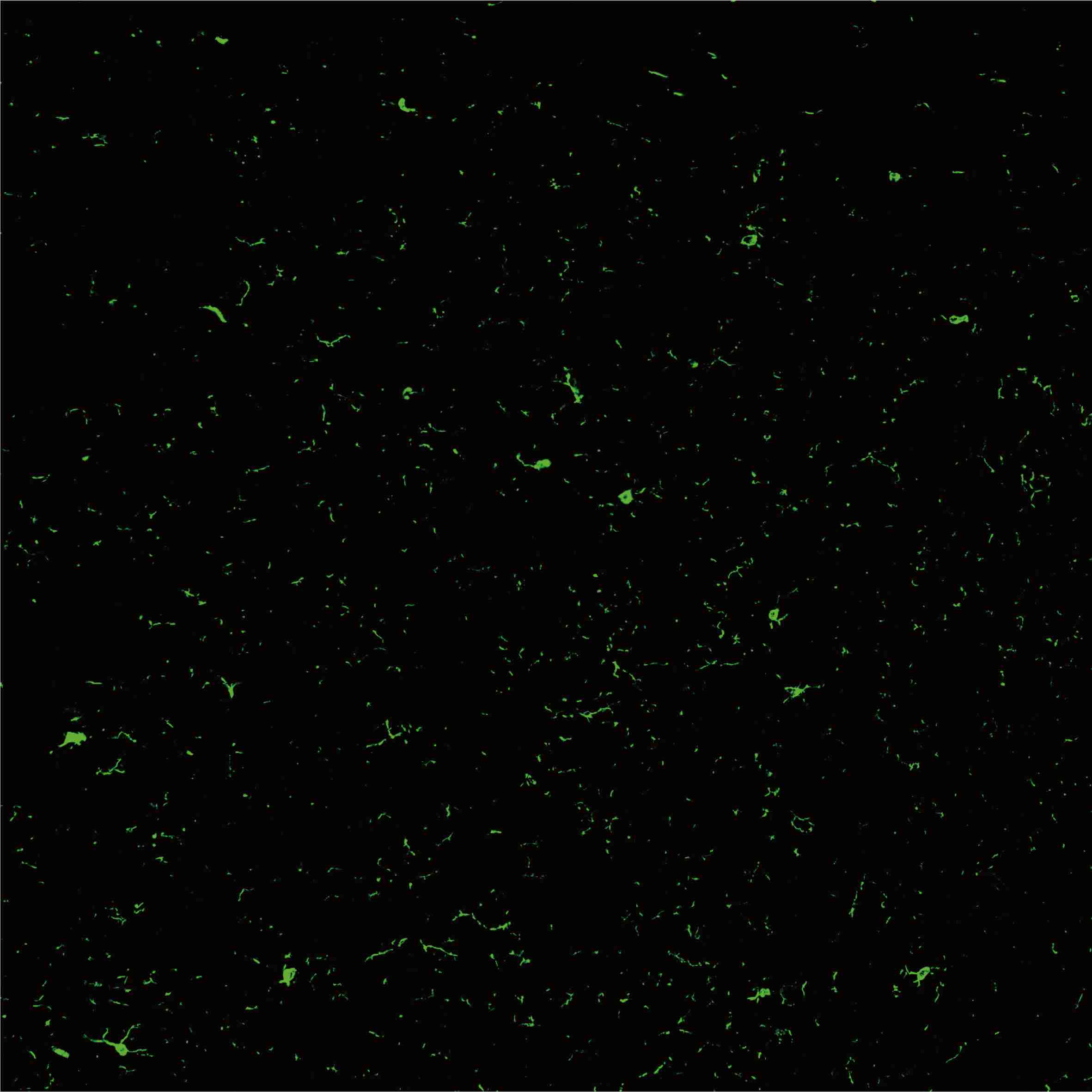

Supplement: Supplementary file 4 — Source data Fig. 2 [file 44321_2025_206_MOESM4_ESM.zip › Source Data Fig 2/Fig 2/2G/WT-Sham IBA-1.tif]

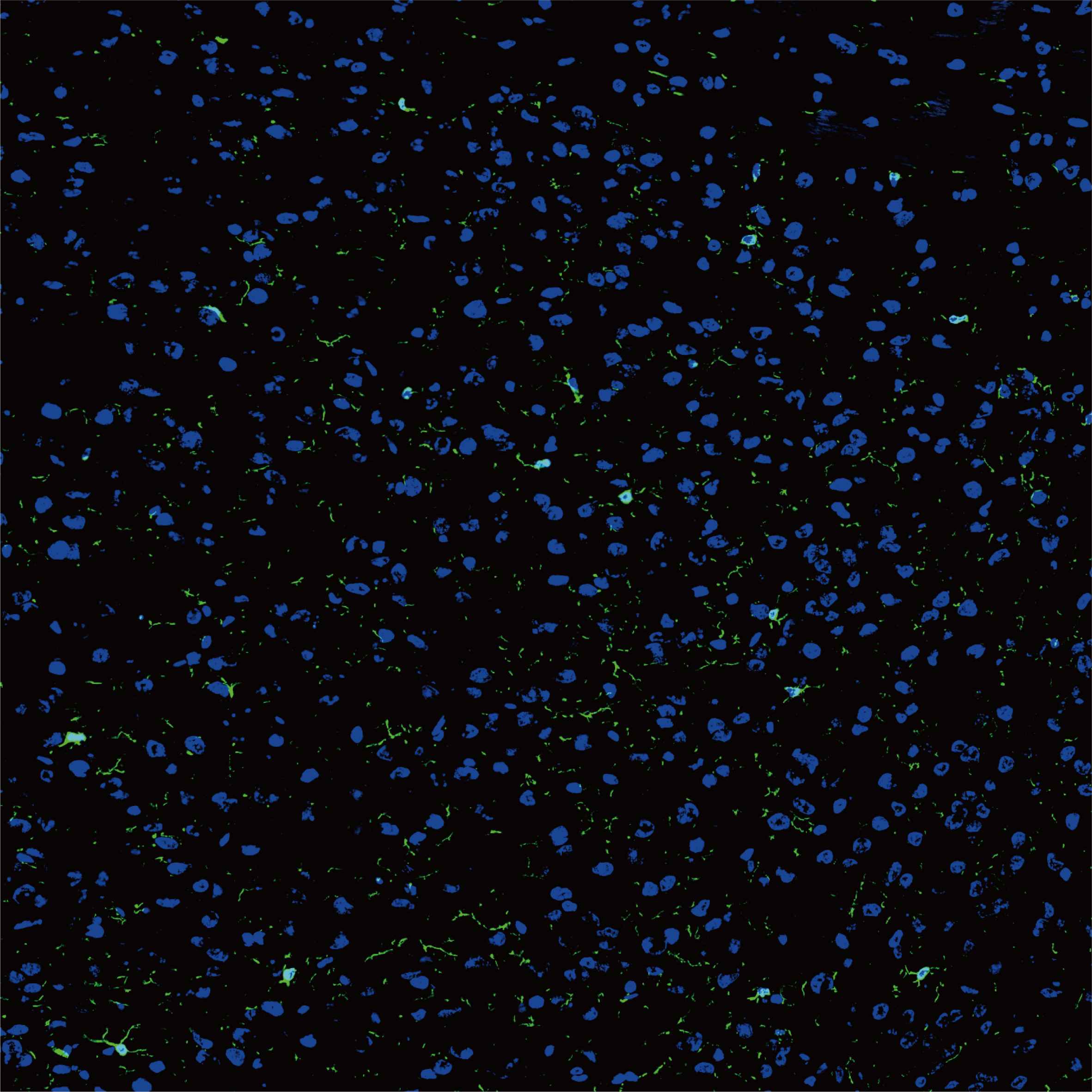

Supplement: Supplementary file 4 — Source data Fig. 2 [file 44321_2025_206_MOESM4_ESM.zip › Source Data Fig 2/Fig 2/2G/WT-Sham Merge.tif]

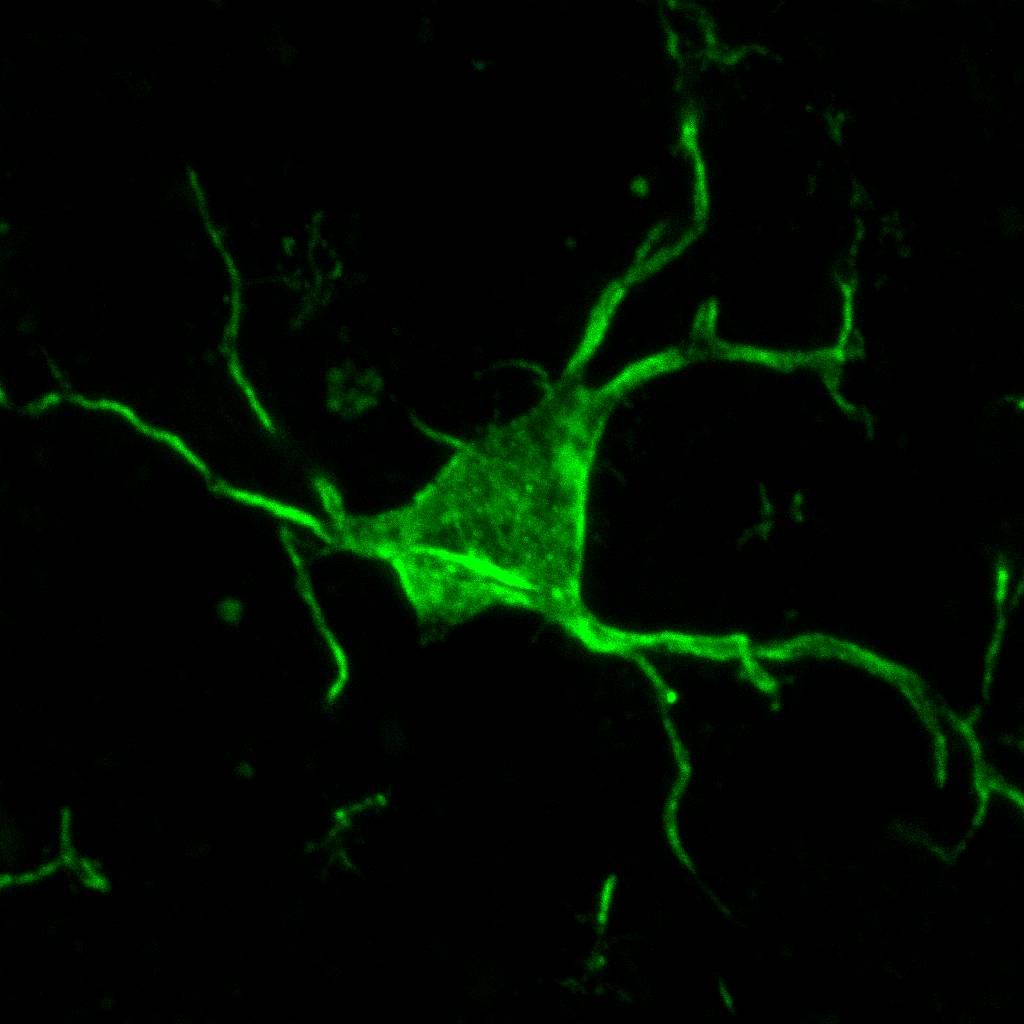

Supplement: Supplementary file 4 — Source data Fig. 2 [file 44321_2025_206_MOESM4_ESM.zip › Source Data Fig 2/Fig 2/2H/KO MCAO IBA-1.tif]

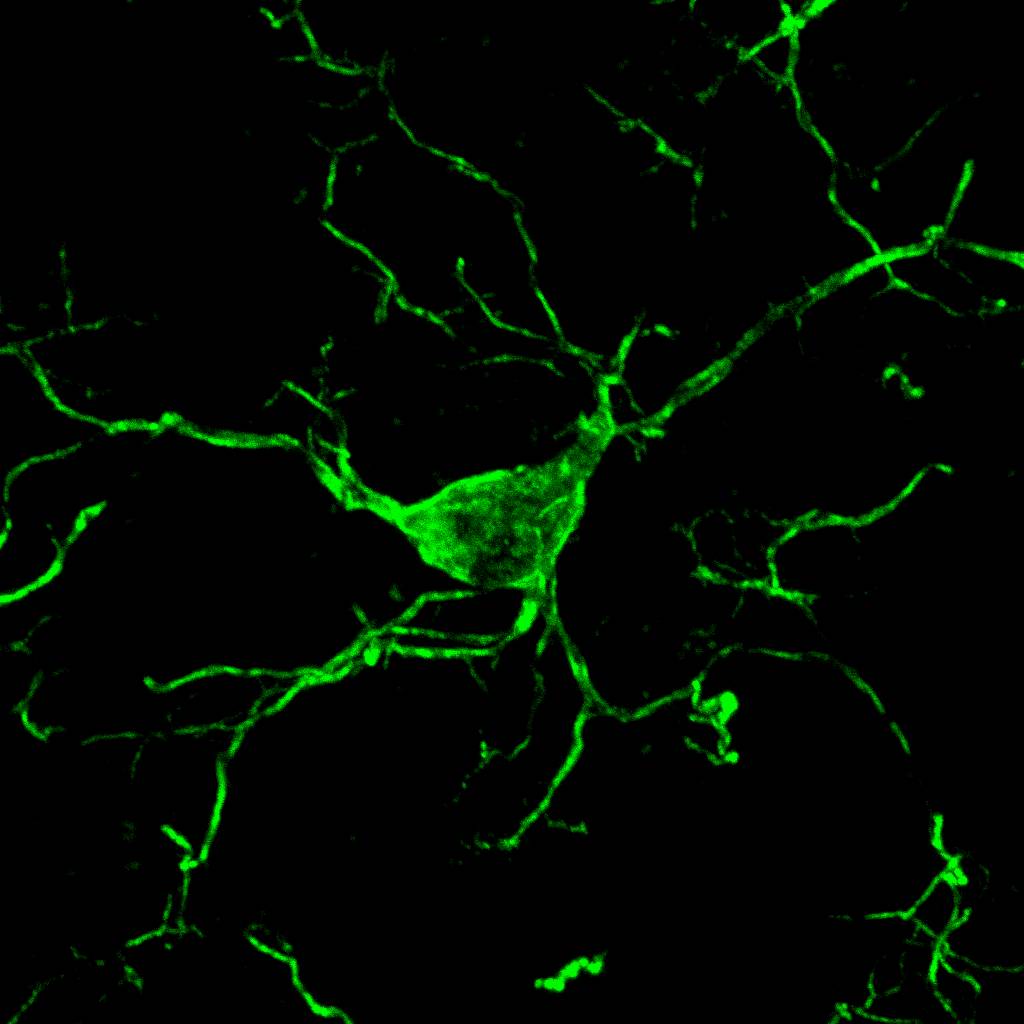

Supplement: Supplementary file 4 — Source data Fig. 2 [file 44321_2025_206_MOESM4_ESM.zip › Source Data Fig 2/Fig 2/2H/KO Sham IBA-1.tif]

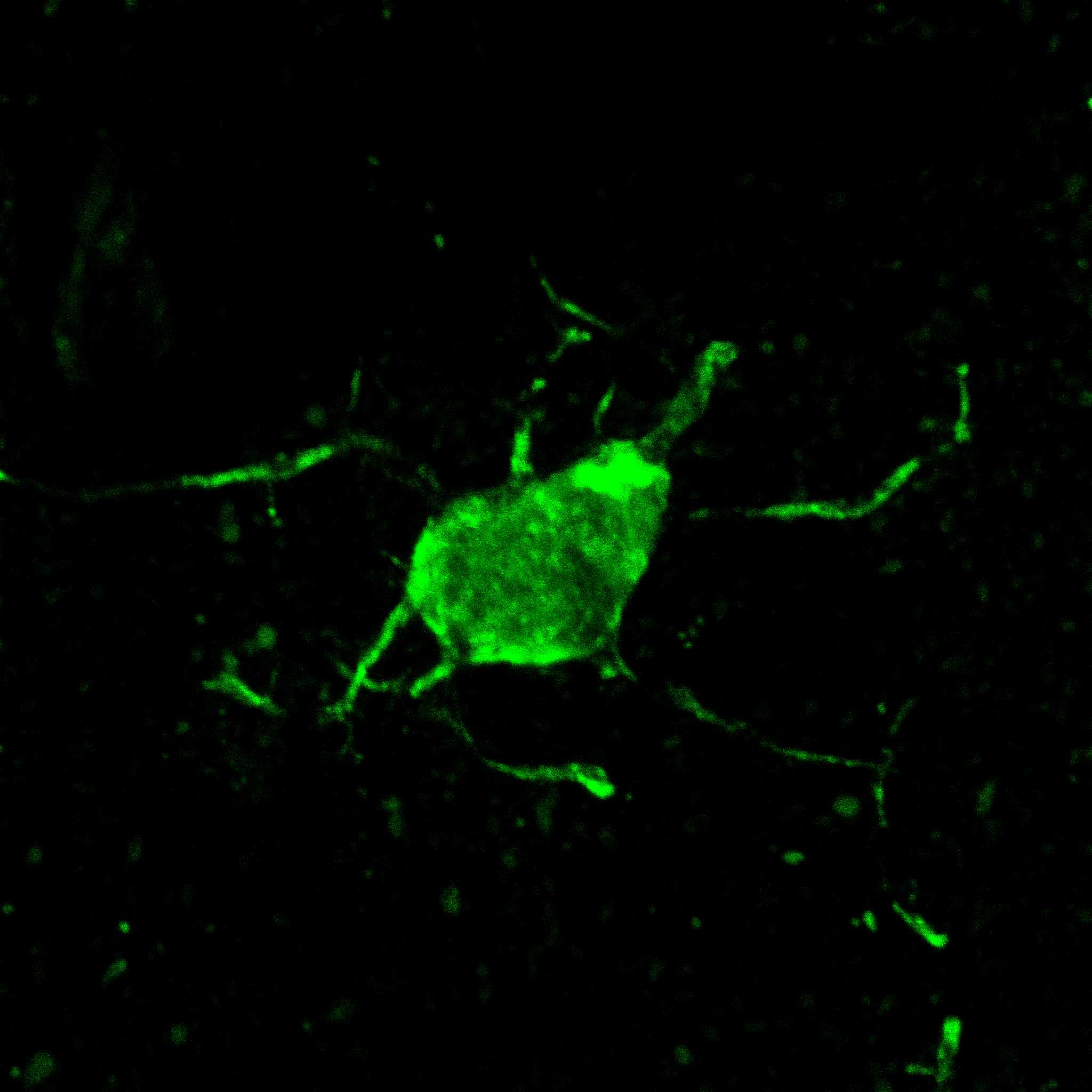

Supplement: Supplementary file 4 — Source data Fig. 2 [file 44321_2025_206_MOESM4_ESM.zip › Source Data Fig 2/Fig 2/2H/WT-MCAO IBA-1.tif]

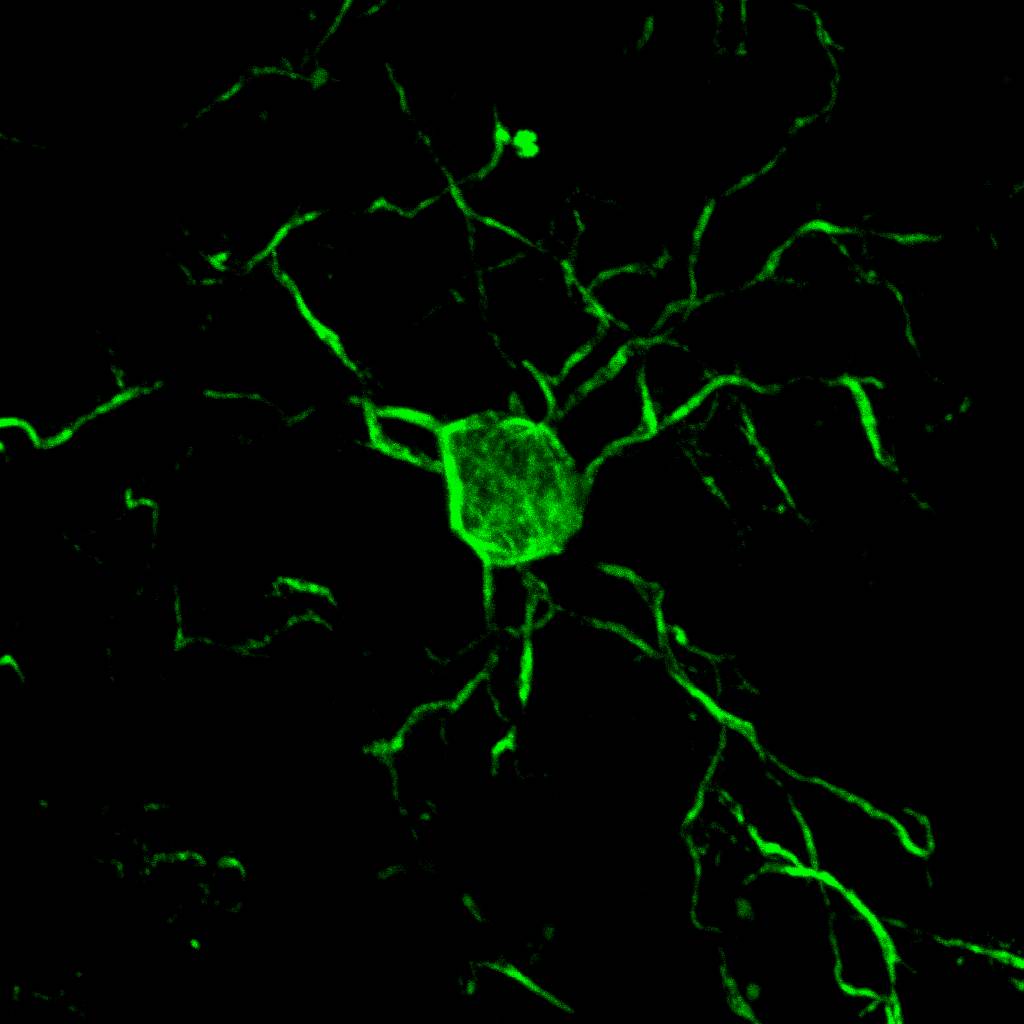

Supplement: Supplementary file 4 — Source data Fig. 2 [file 44321_2025_206_MOESM4_ESM.zip › Source Data Fig 2/Fig 2/2H/WT-Sham IBA-1.tif]

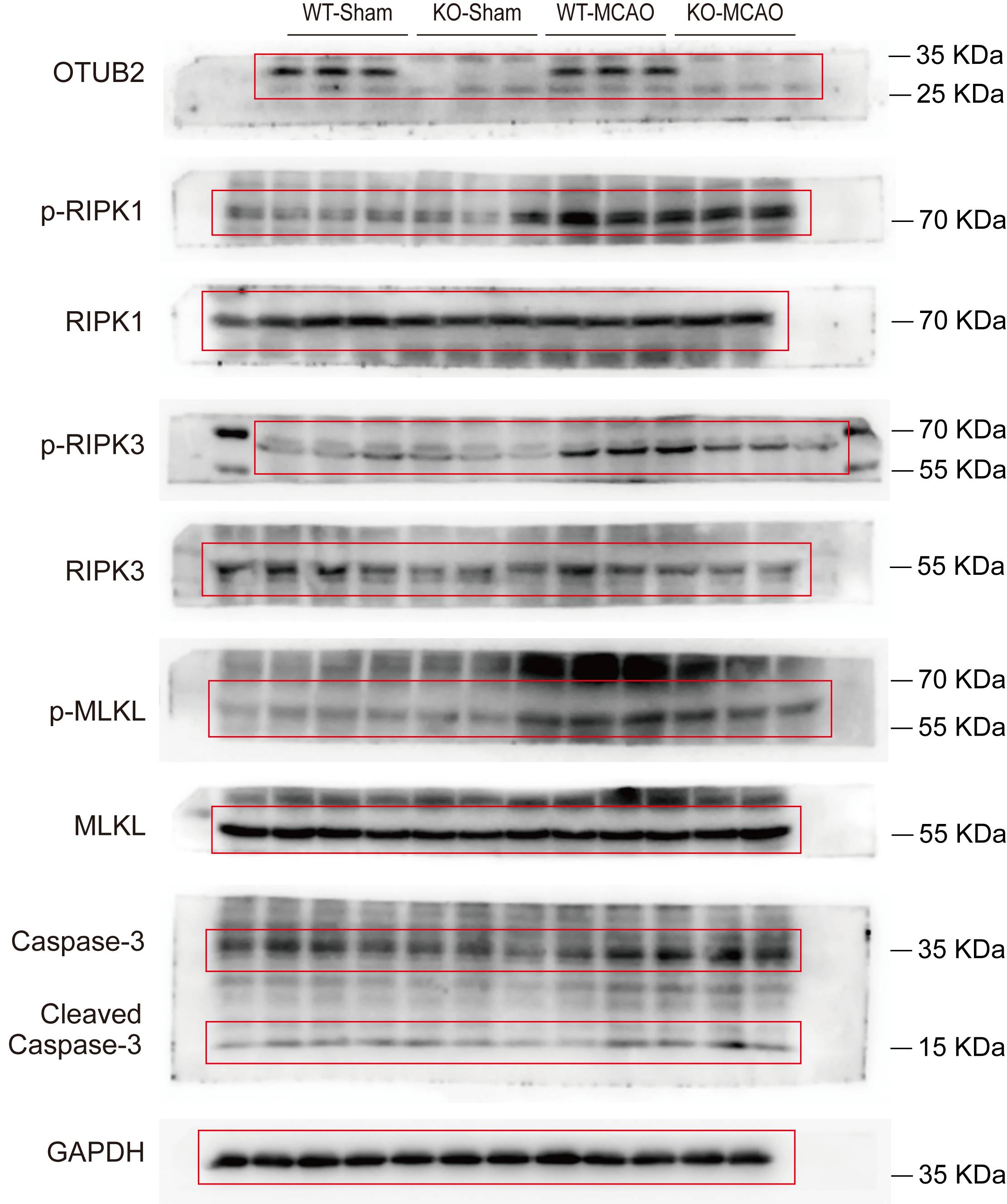

Supplement: Supplementary file 5 — Source data Fig. 3 [file 44321_2025_206_MOESM5_ESM.zip › Source Data Fig 3/Fig 3/3A/3A.tif]

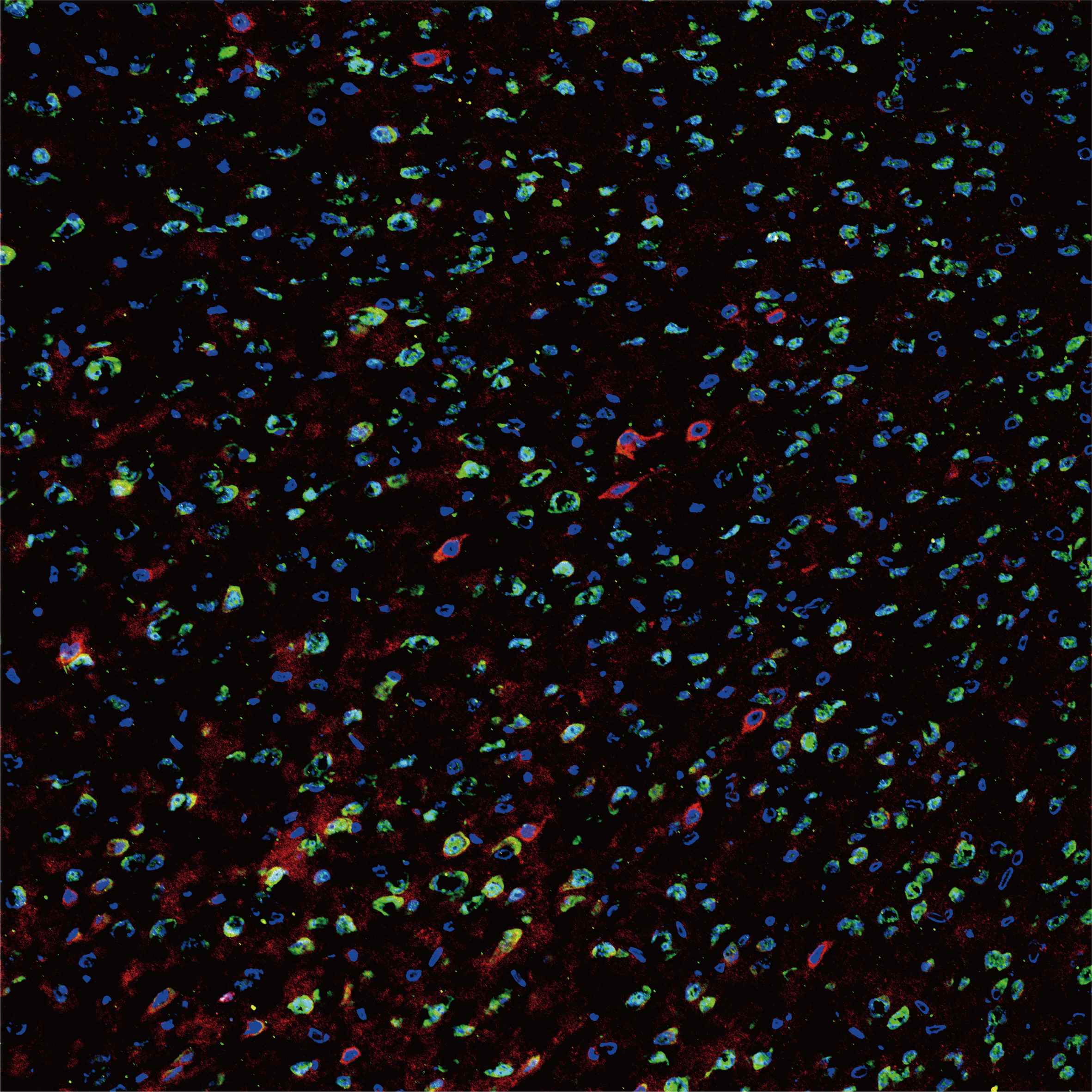

Supplement: Supplementary file 5 — Source data Fig. 3 [file 44321_2025_206_MOESM5_ESM.zip › Source Data Fig 3/Fig 3/3H/KO-MCAO-Merge.tif]

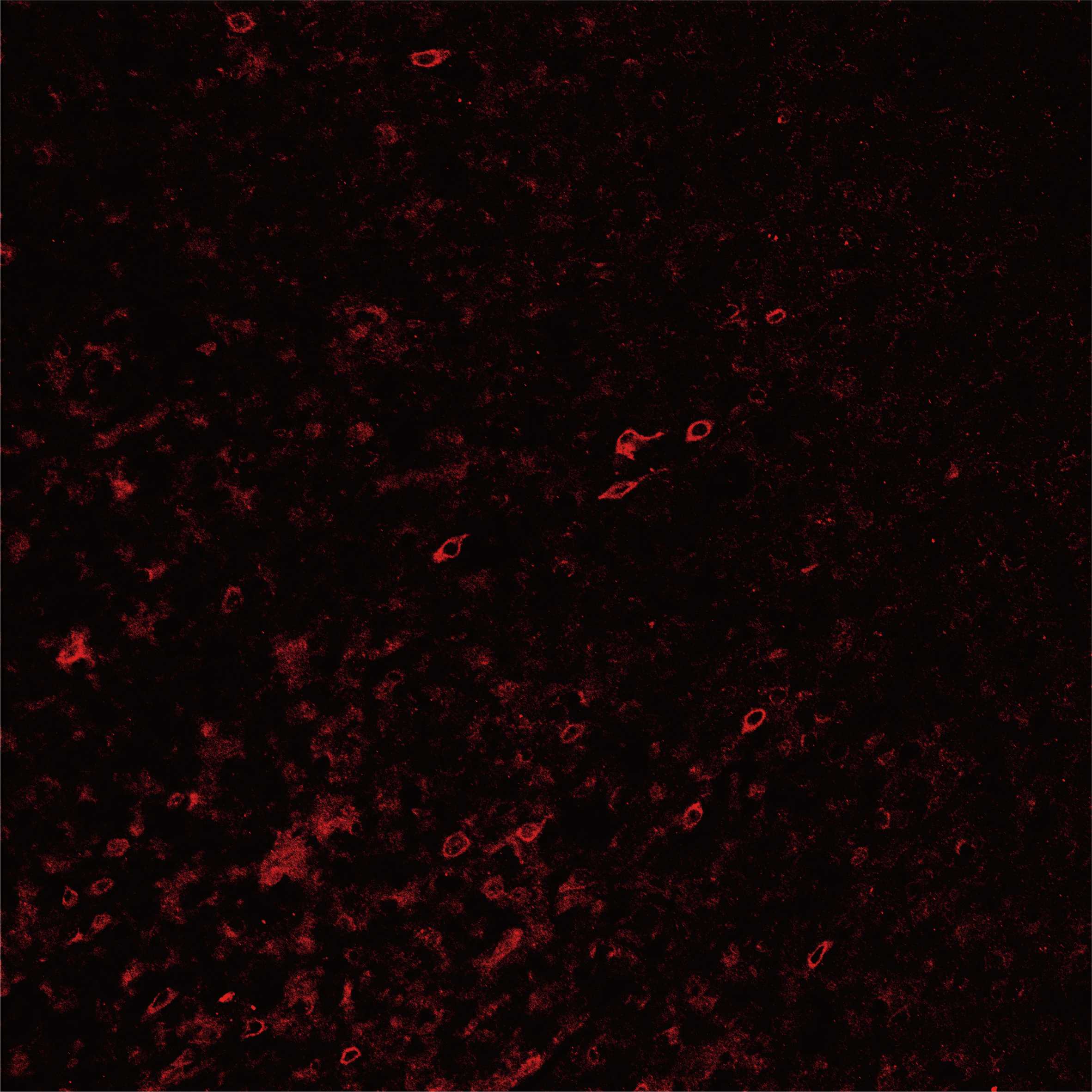

Supplement: Supplementary file 5 — Source data Fig. 3 [file 44321_2025_206_MOESM5_ESM.zip › Source Data Fig 3/Fig 3/3H/KO-MCAO-MLKL.tif]

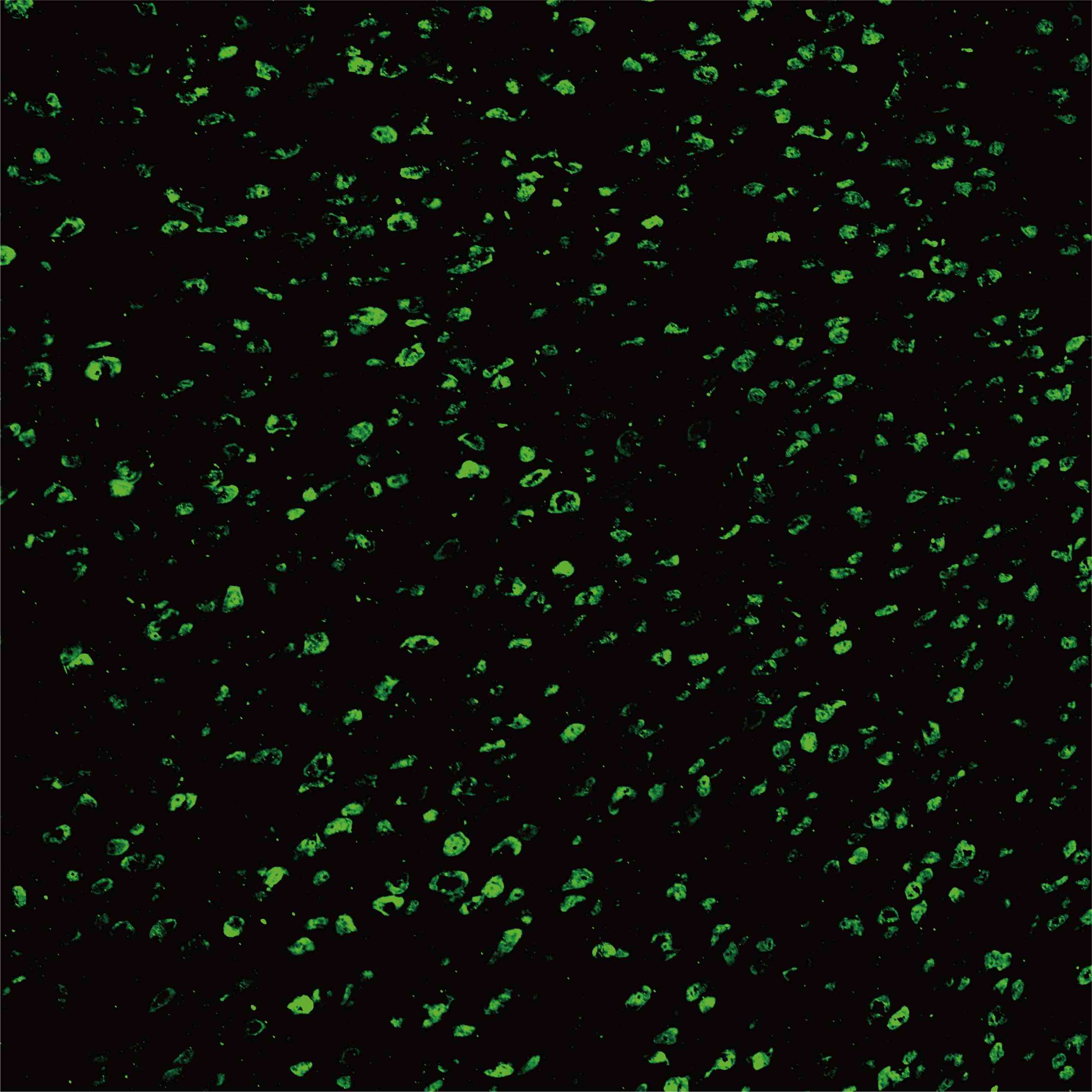

Supplement: Supplementary file 5 — Source data Fig. 3 [file 44321_2025_206_MOESM5_ESM.zip › Source Data Fig 3/Fig 3/3H/KO-MCAO-NEUN.tif]

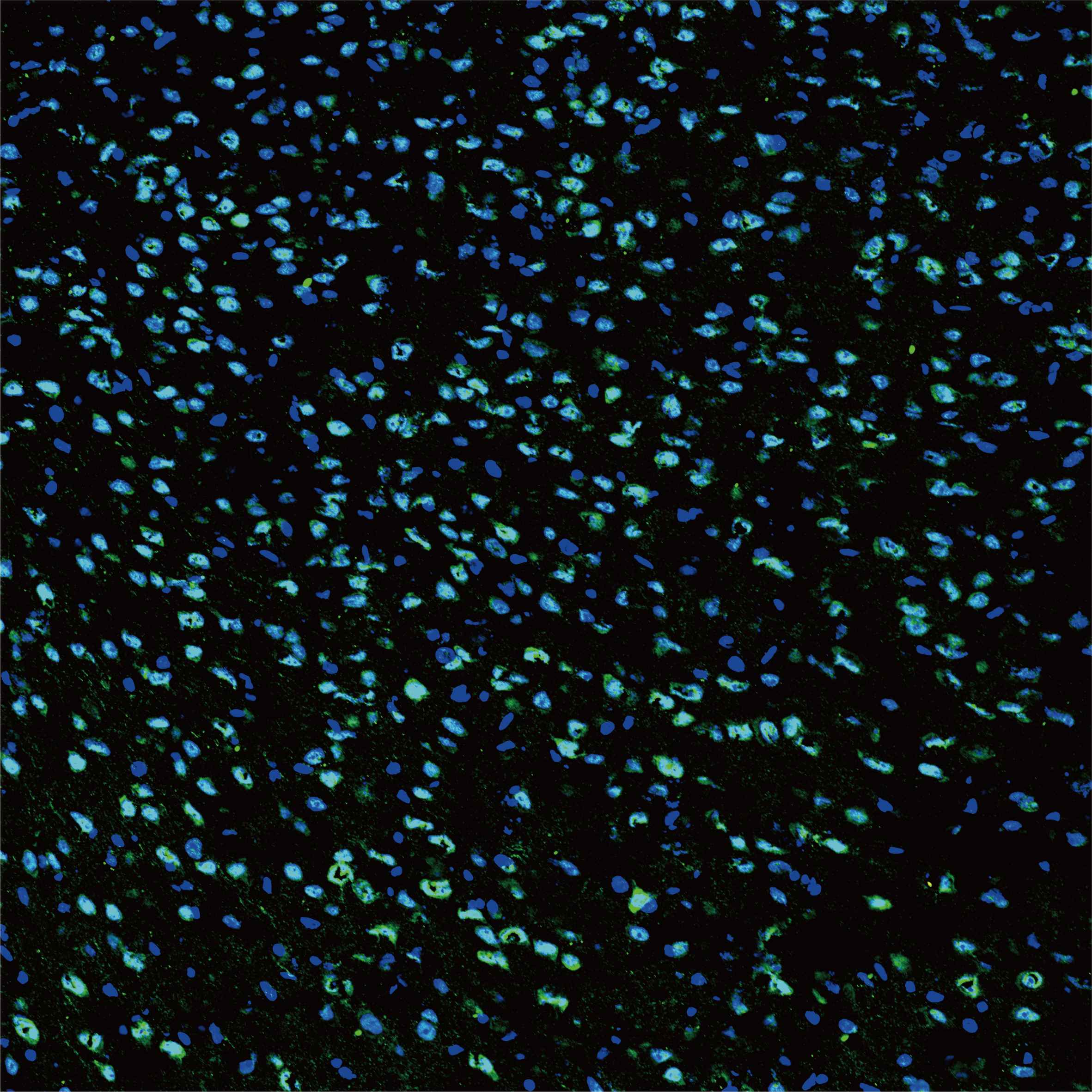

Supplement: Supplementary file 5 — Source data Fig. 3 [file 44321_2025_206_MOESM5_ESM.zip › Source Data Fig 3/Fig 3/3H/KO-Sham-Merge.tif]

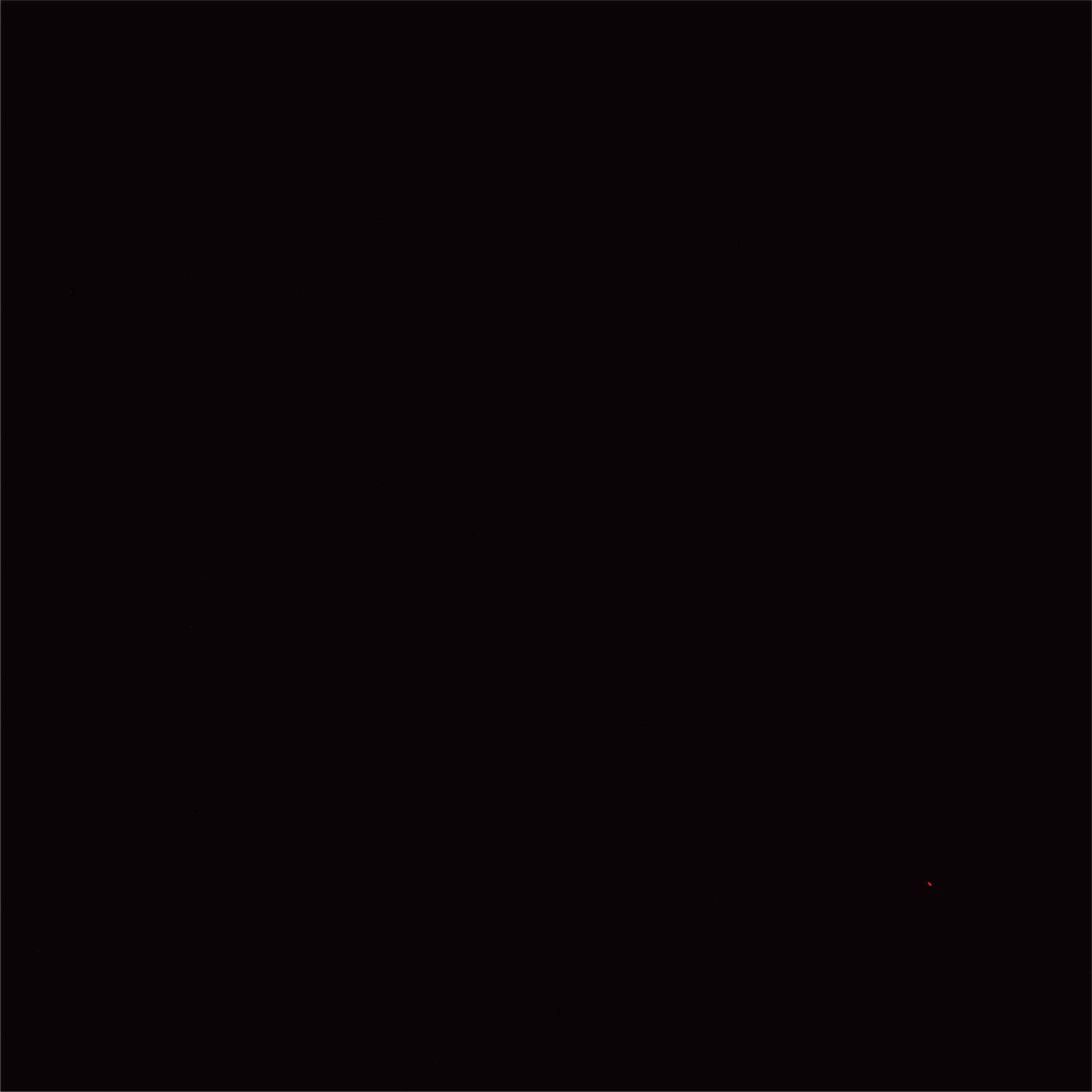

Supplement: Supplementary file 5 — Source data Fig. 3 [file 44321_2025_206_MOESM5_ESM.zip › Source Data Fig 3/Fig 3/3H/KO-Sham-MLKL.tif]

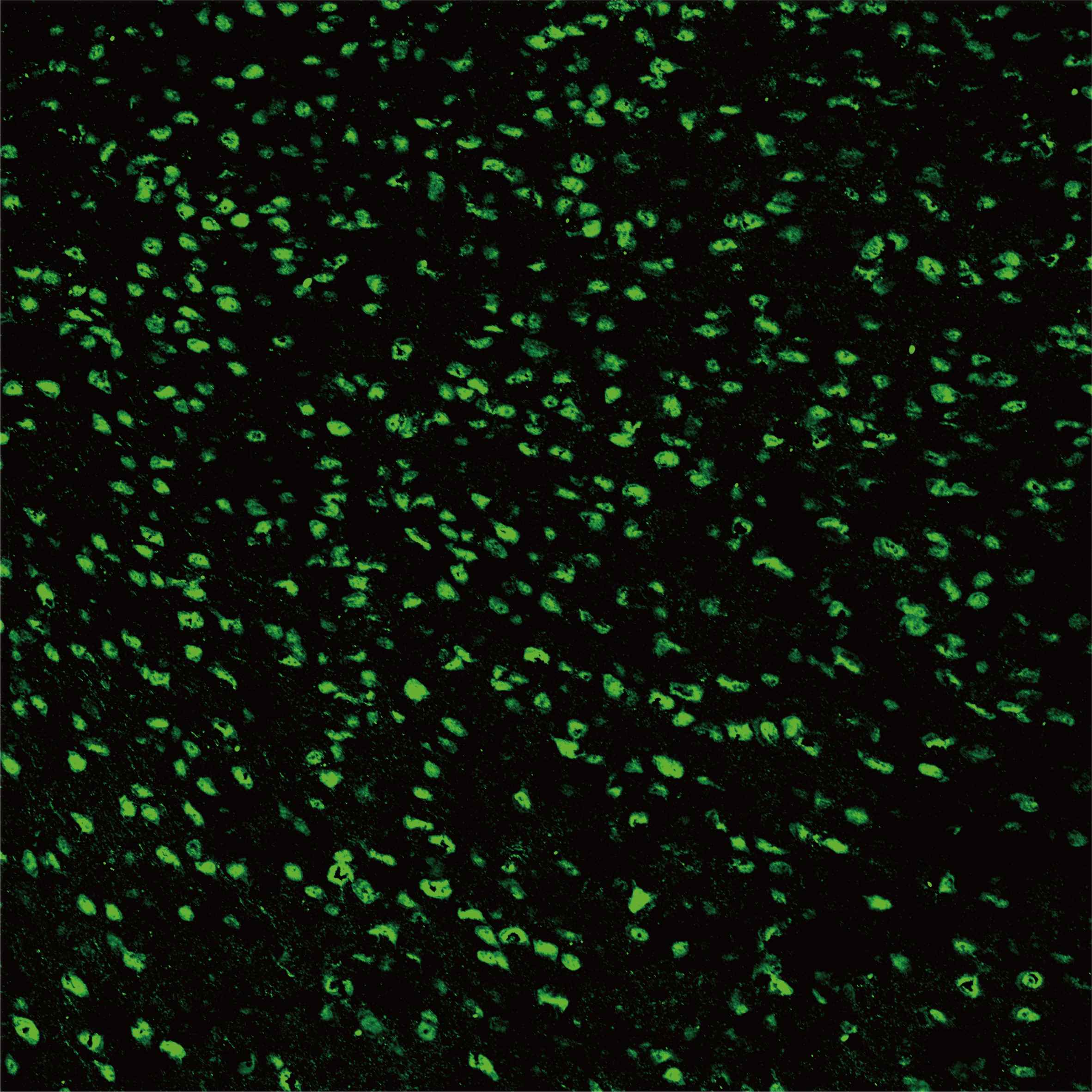

Supplement: Supplementary file 5 — Source data Fig. 3 [file 44321_2025_206_MOESM5_ESM.zip › Source Data Fig 3/Fig 3/3H/KO-Sham-NEUN.tif]

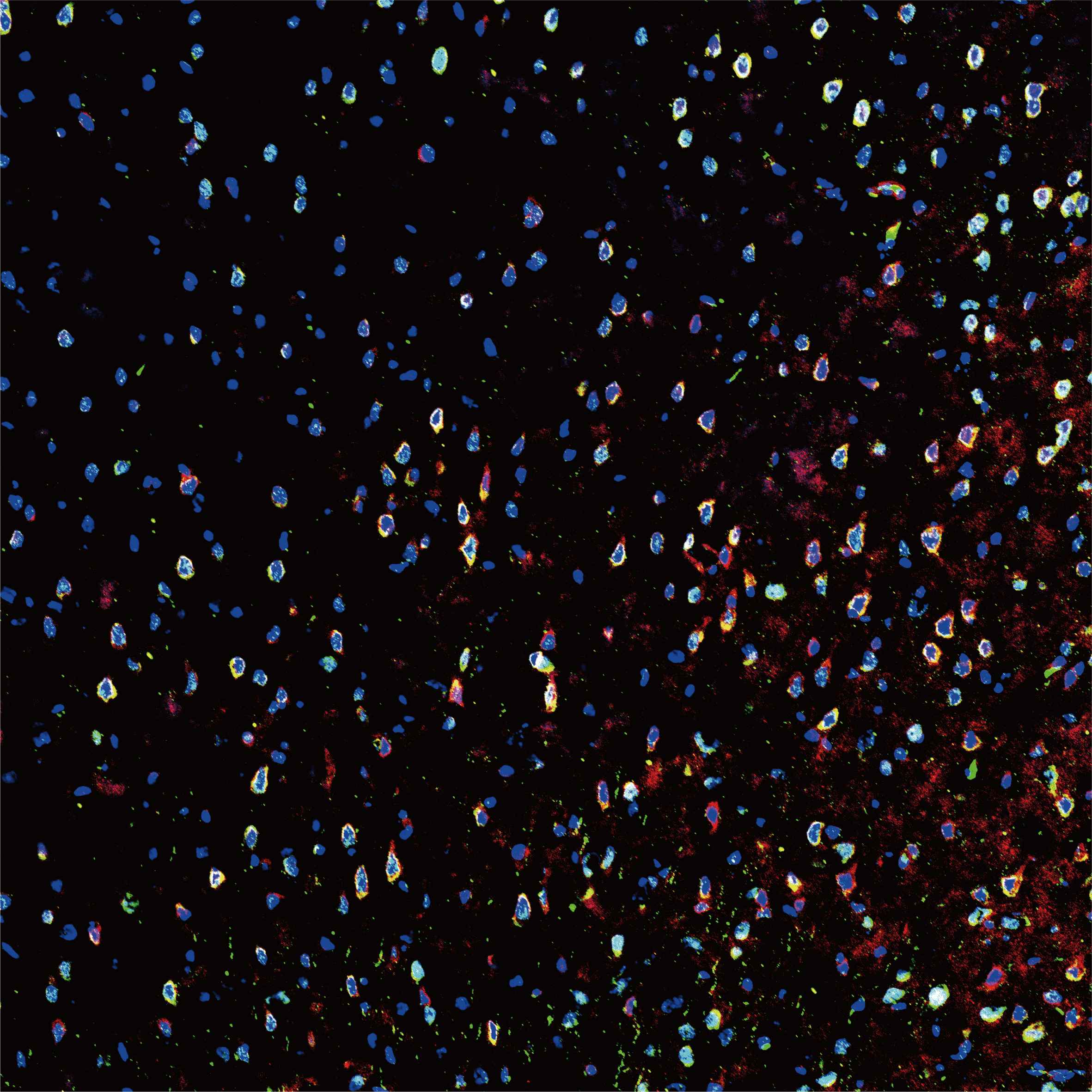

Supplement: Supplementary file 5 — Source data Fig. 3 [file 44321_2025_206_MOESM5_ESM.zip › Source Data Fig 3/Fig 3/3H/WT-MCAO-Merge.tif]

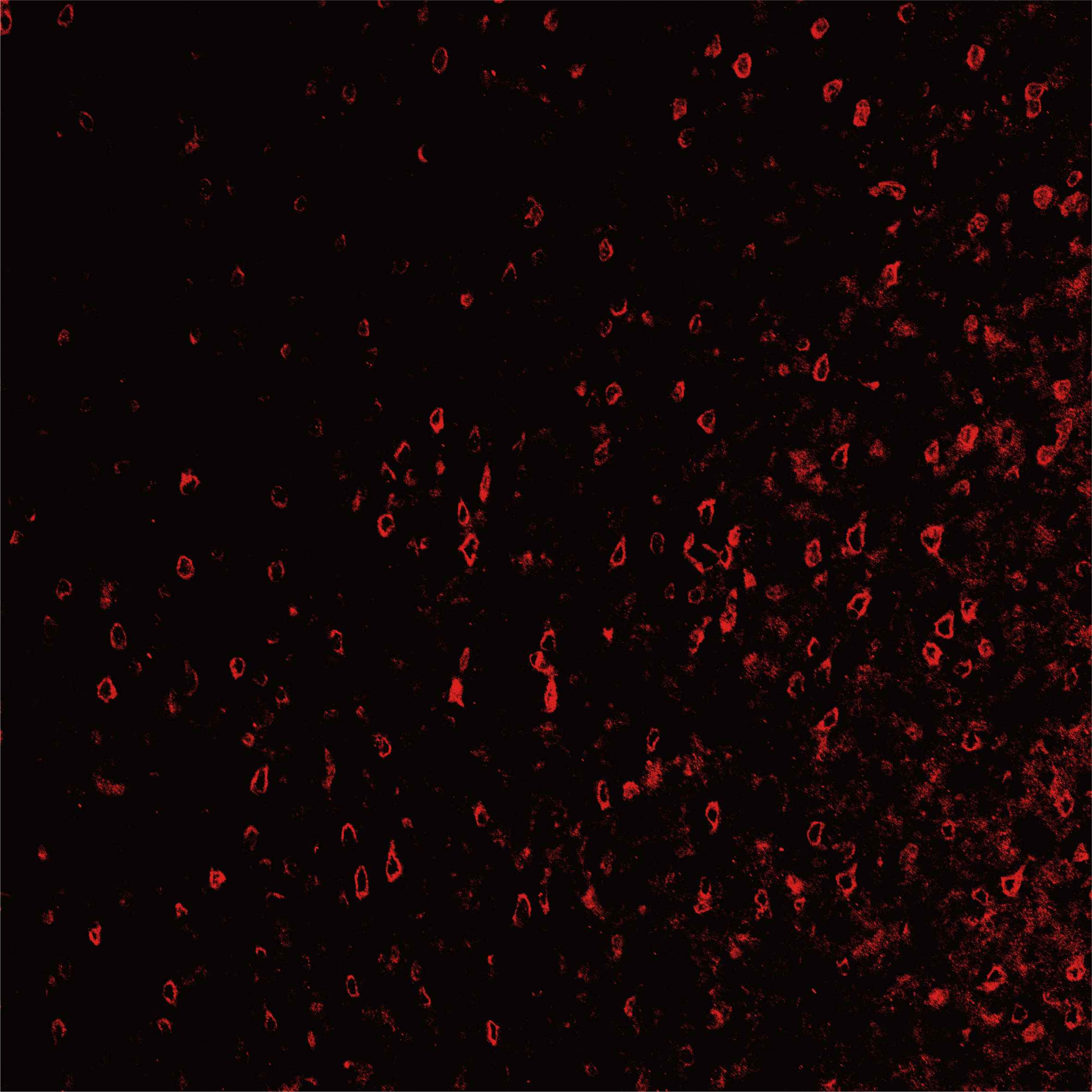

Supplement: Supplementary file 5 — Source data Fig. 3 [file 44321_2025_206_MOESM5_ESM.zip › Source Data Fig 3/Fig 3/3H/WT-MCAO-MLKL.tif]

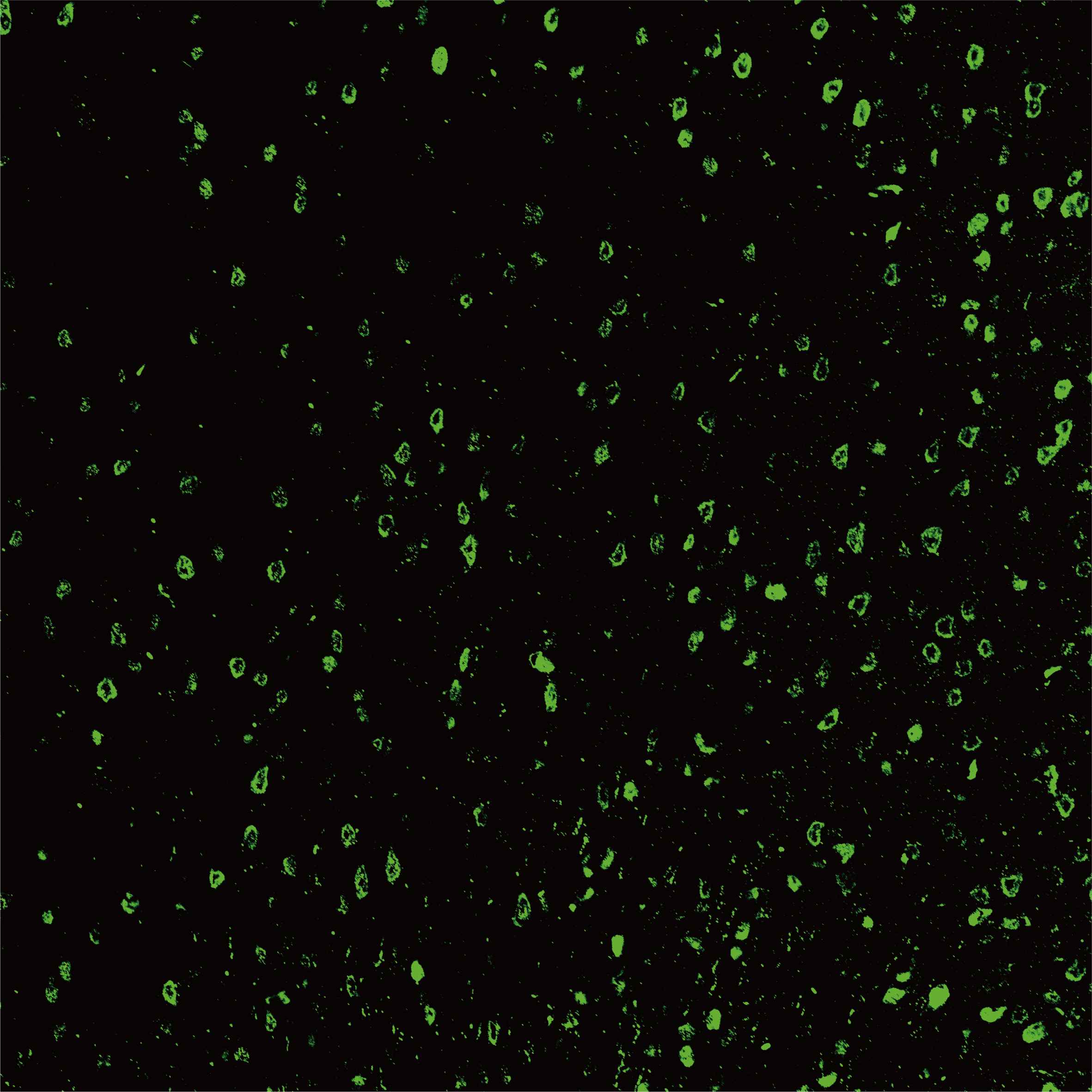

Supplement: Supplementary file 5 — Source data Fig. 3 [file 44321_2025_206_MOESM5_ESM.zip › Source Data Fig 3/Fig 3/3H/WT-MCAO-NEUN.tif]

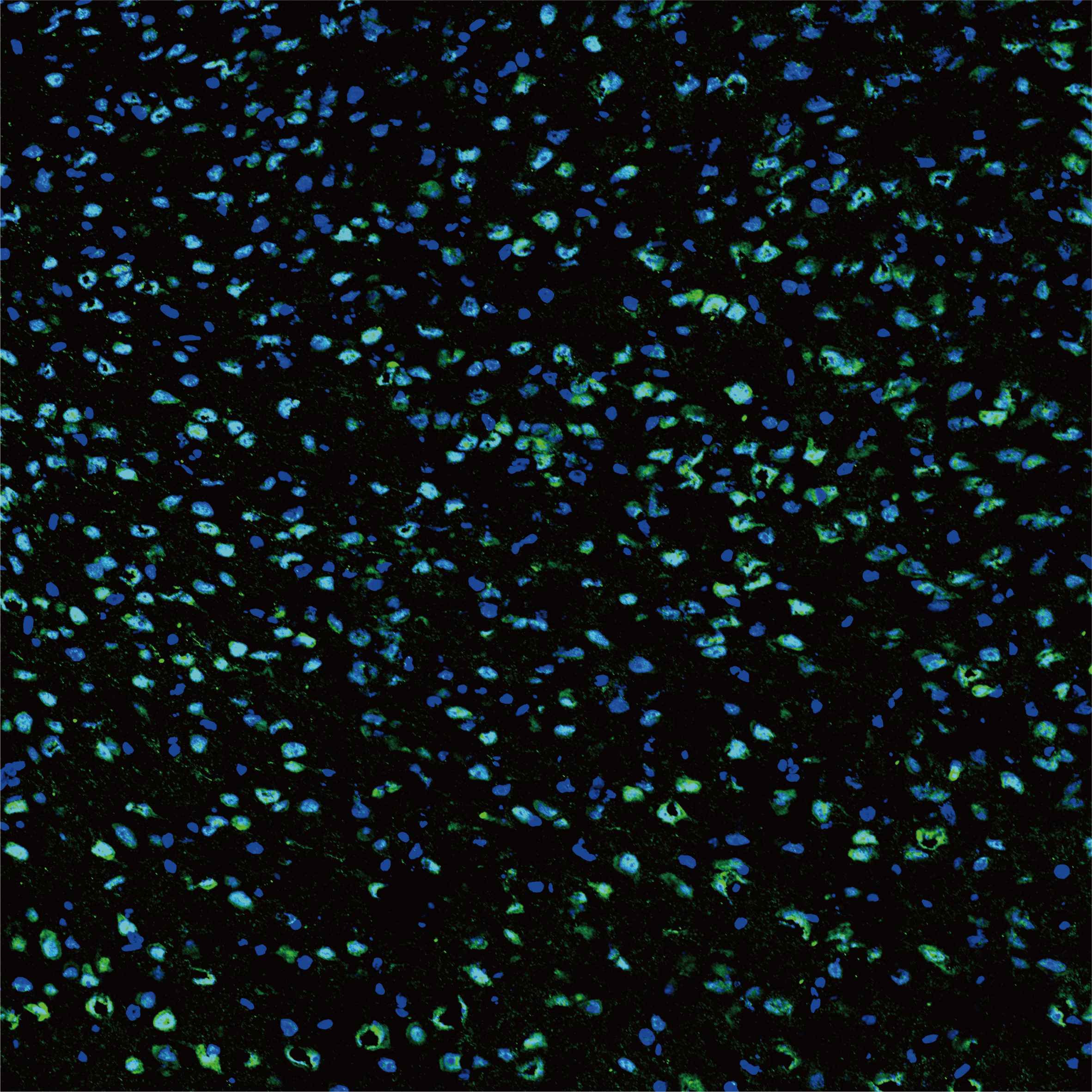

Supplement: Supplementary file 5 — Source data Fig. 3 [file 44321_2025_206_MOESM5_ESM.zip › Source Data Fig 3/Fig 3/3H/WT-Sham-Merge.tif]

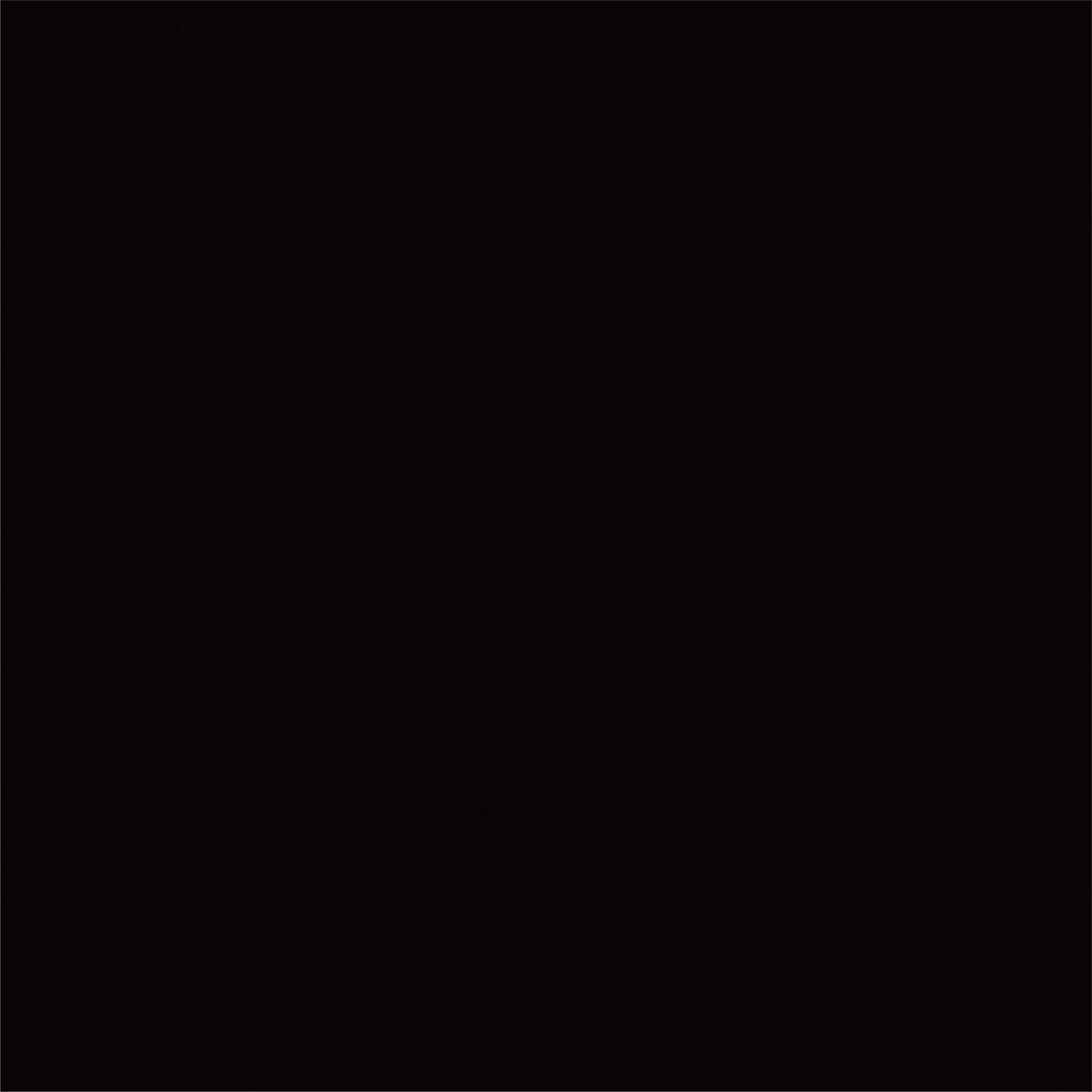

Supplement: Supplementary file 5 — Source data Fig. 3 [file 44321_2025_206_MOESM5_ESM.zip › Source Data Fig 3/Fig 3/3H/WT-Sham-MLKL.tif]

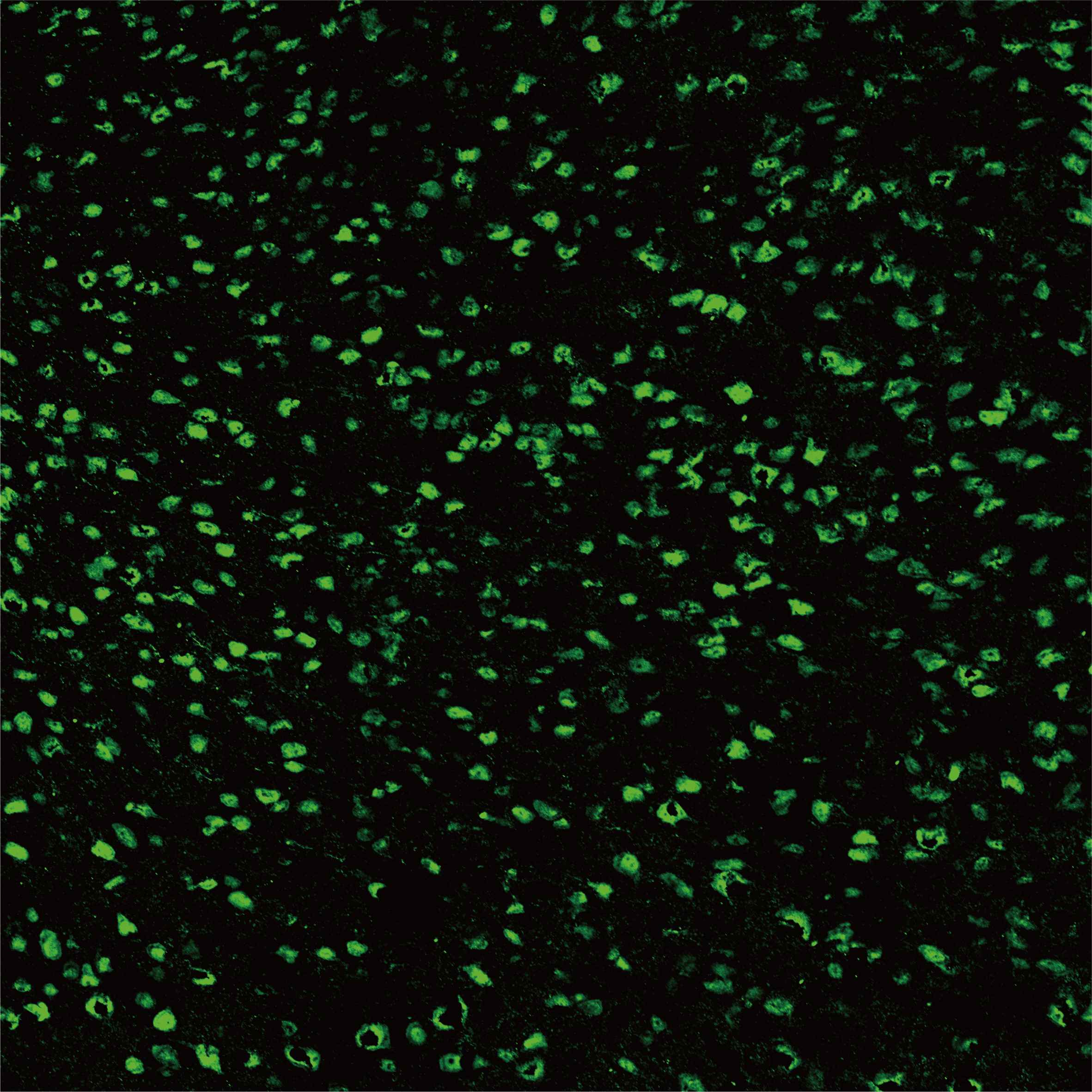

Supplement: Supplementary file 5 — Source data Fig. 3 [file 44321_2025_206_MOESM5_ESM.zip › Source Data Fig 3/Fig 3/3H/WT-Sham-NEUN.tif]

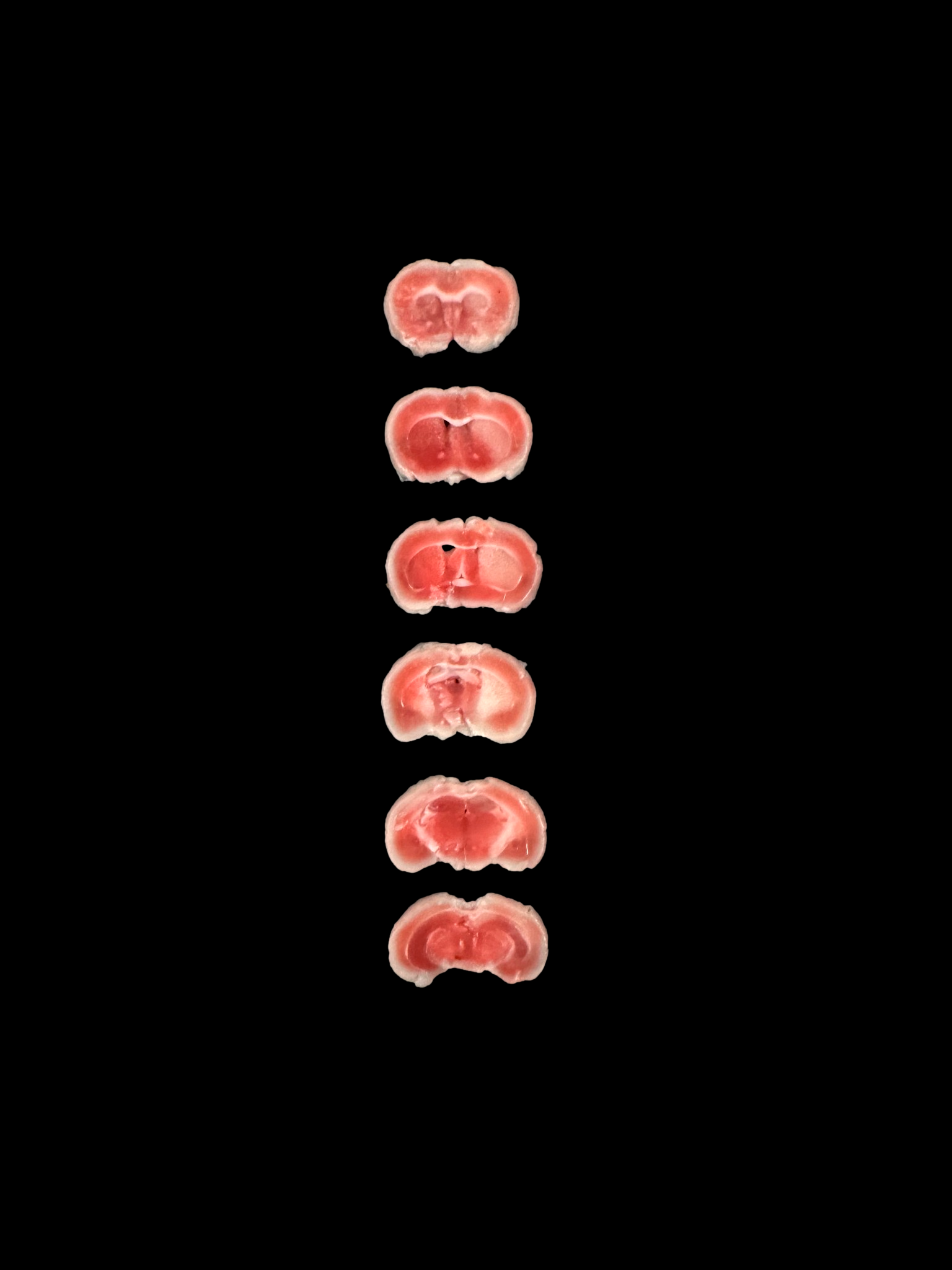

Supplement: Supplementary file 5 — Source data Fig. 3 [file 44321_2025_206_MOESM5_ESM.zip › Source Data Fig 3/Fig 3/3M-N/KO-D1.tif]

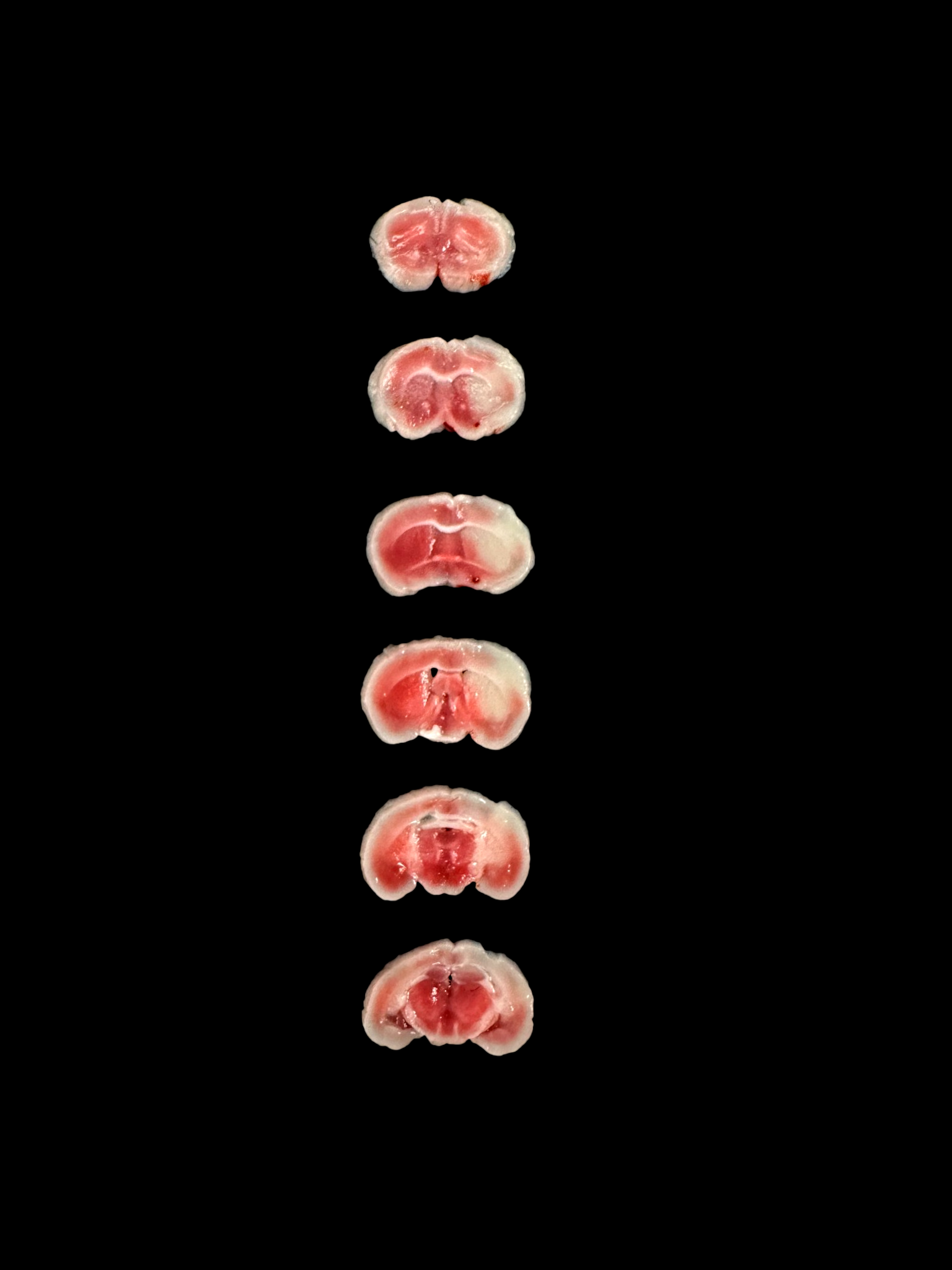

Supplement: Supplementary file 5 — Source data Fig. 3 [file 44321_2025_206_MOESM5_ESM.zip › Source Data Fig 3/Fig 3/3M-N/WT-D1.tif]

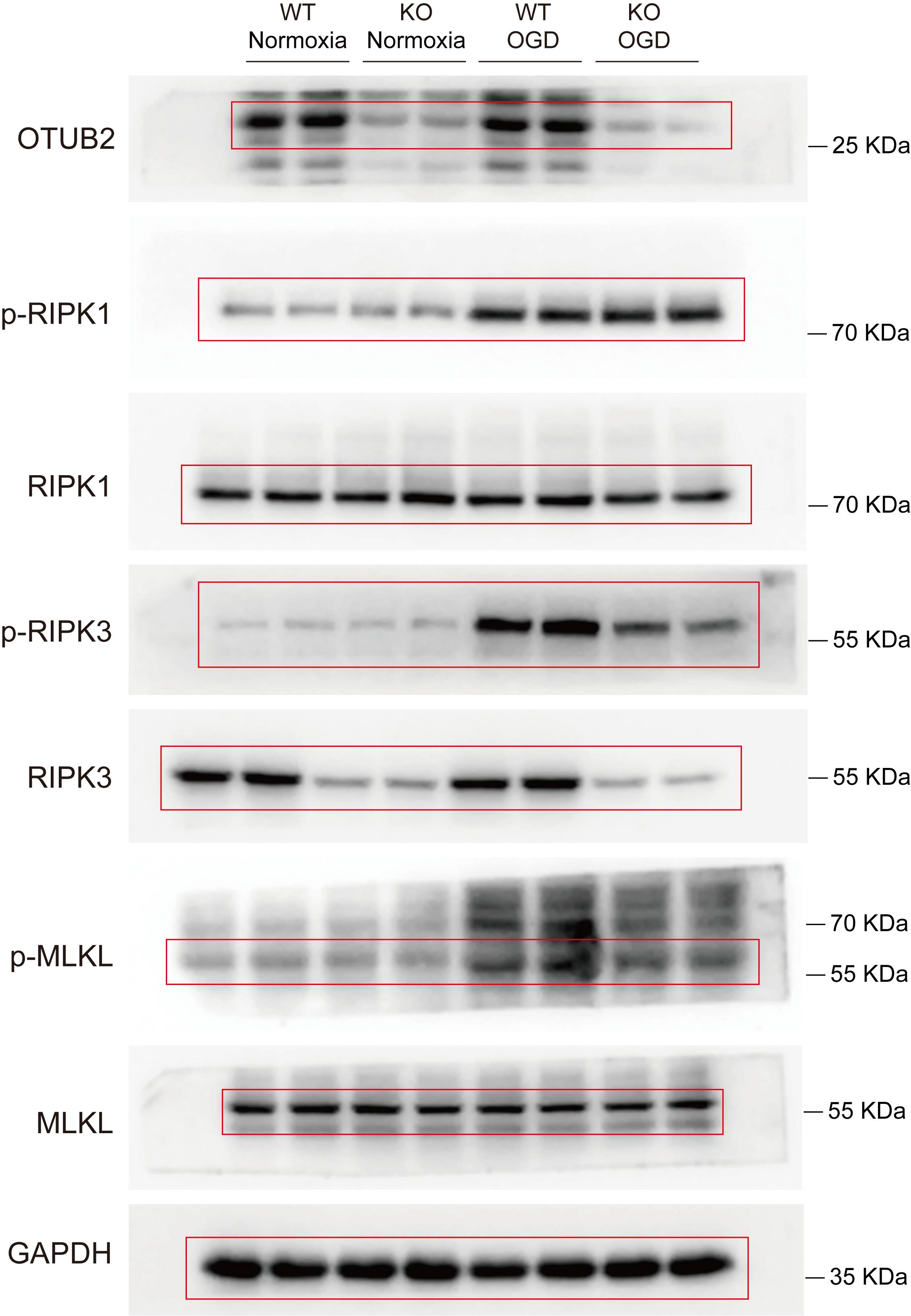

Supplement: Supplementary file 5 — Source data Fig. 3 [file 44321_2025_206_MOESM5_ESM.zip › Source Data Fig 3/Fig 3/3P/3P.tif]

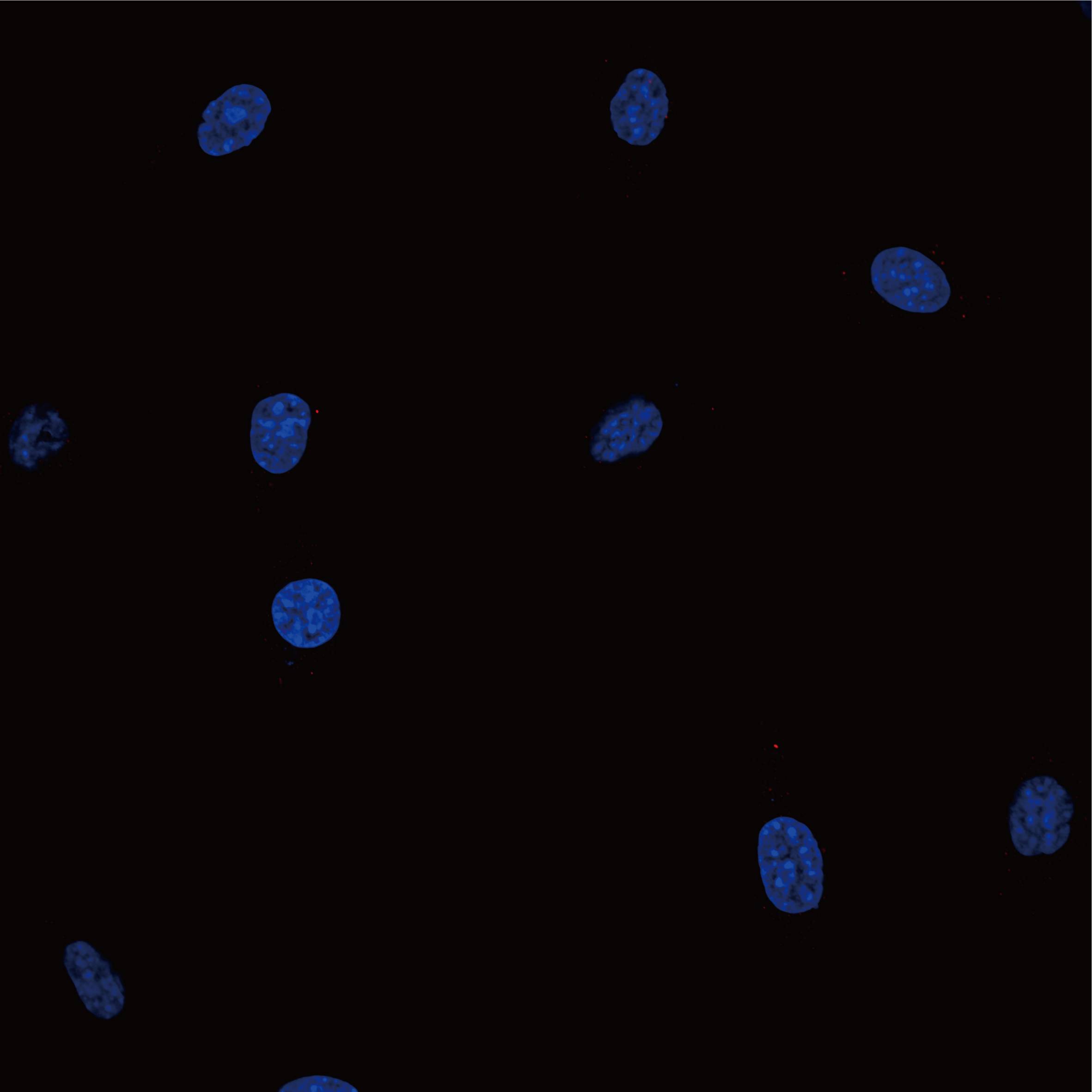

Supplement: Supplementary file 5 — Source data Fig. 3 [file 44321_2025_206_MOESM5_ESM.zip › Source Data Fig 3/Fig 3/3Q/KO-Normoxia-P-MLKL-DAPI.tif]

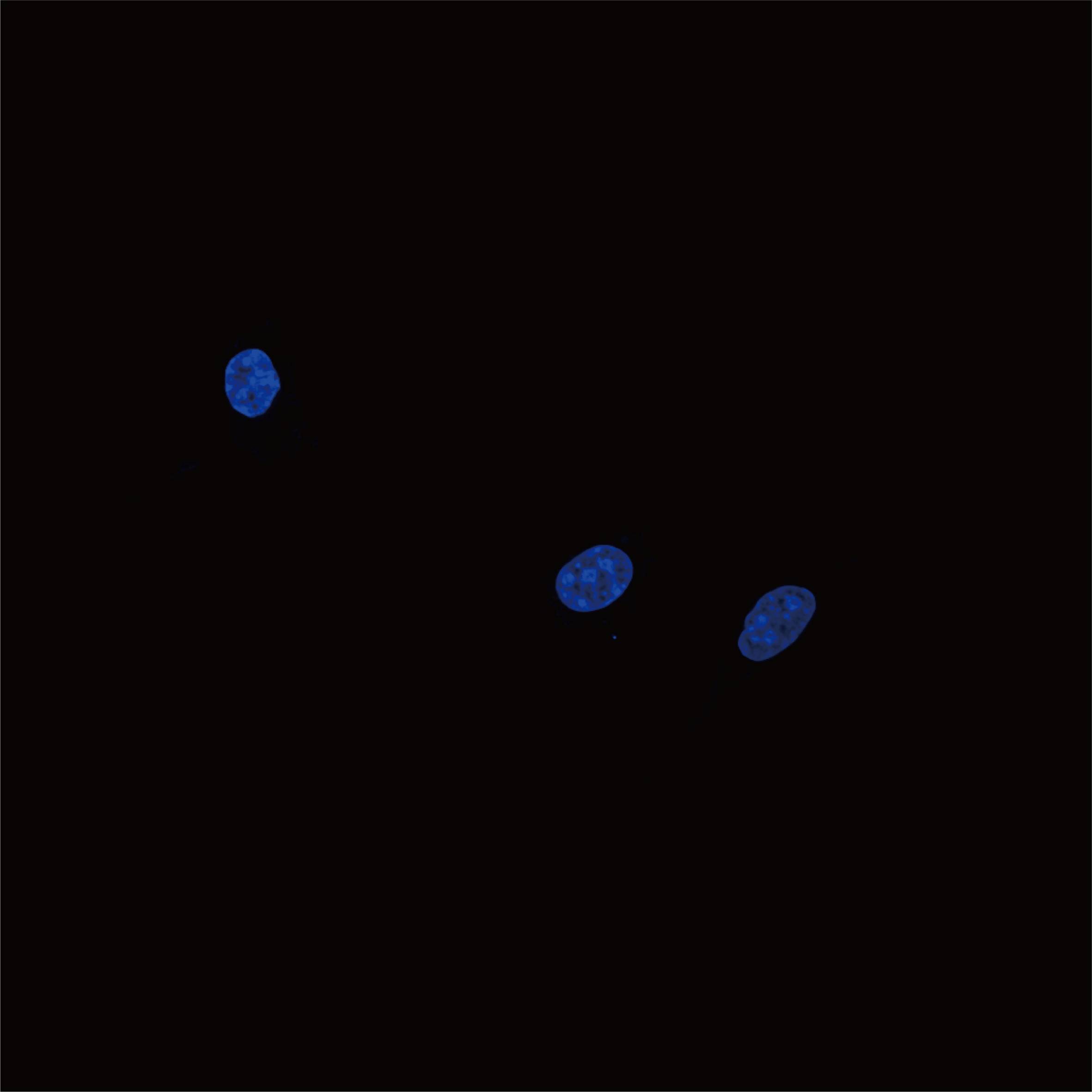

Supplement: Supplementary file 5 — Source data Fig. 3 [file 44321_2025_206_MOESM5_ESM.zip › Source Data Fig 3/Fig 3/3Q/KO-Normoxia-P-RIPK3-DAPI.tif]

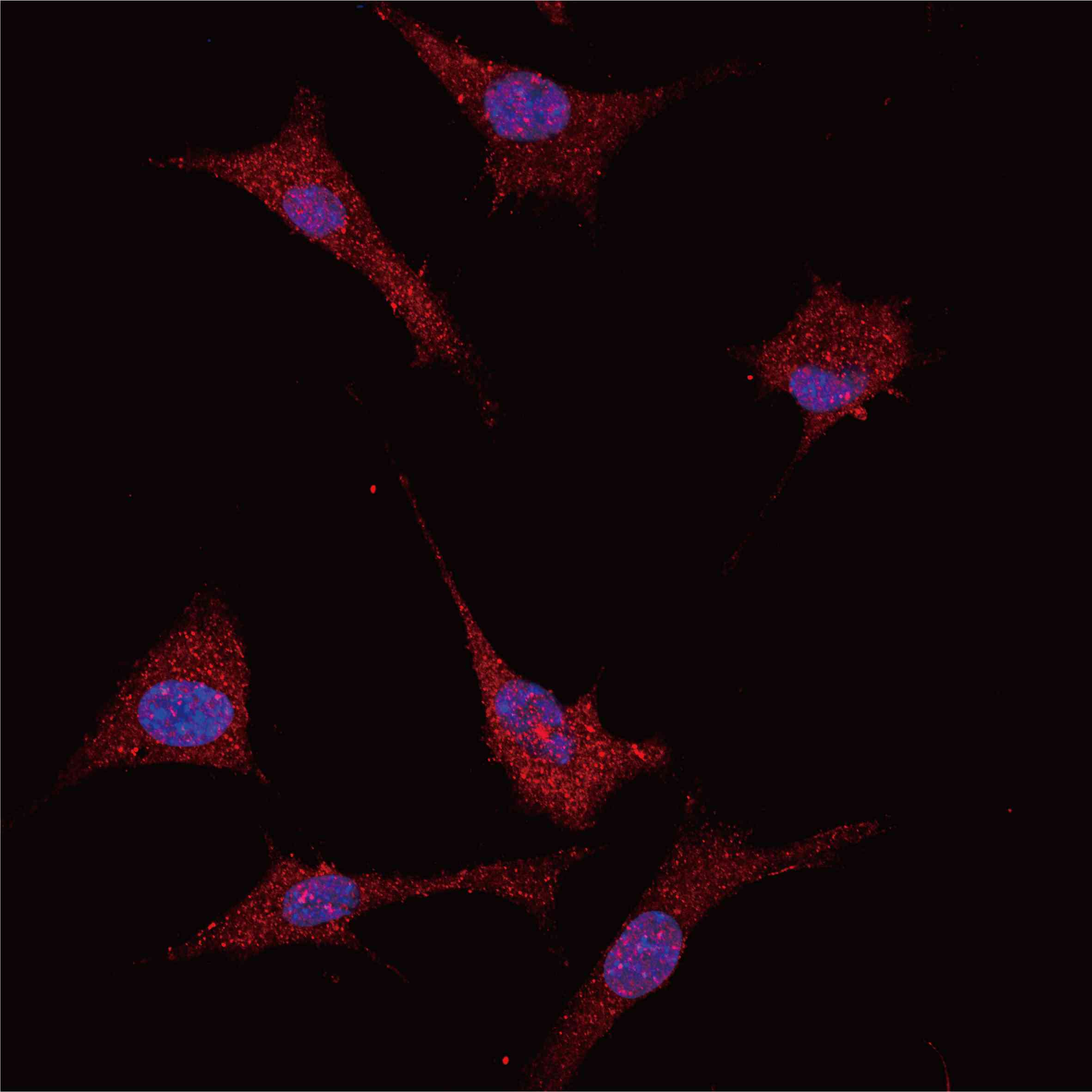

Supplement: Supplementary file 5 — Source data Fig. 3 [file 44321_2025_206_MOESM5_ESM.zip › Source Data Fig 3/Fig 3/3Q/KO-OGD-P-MLKL-DAPI.tif]

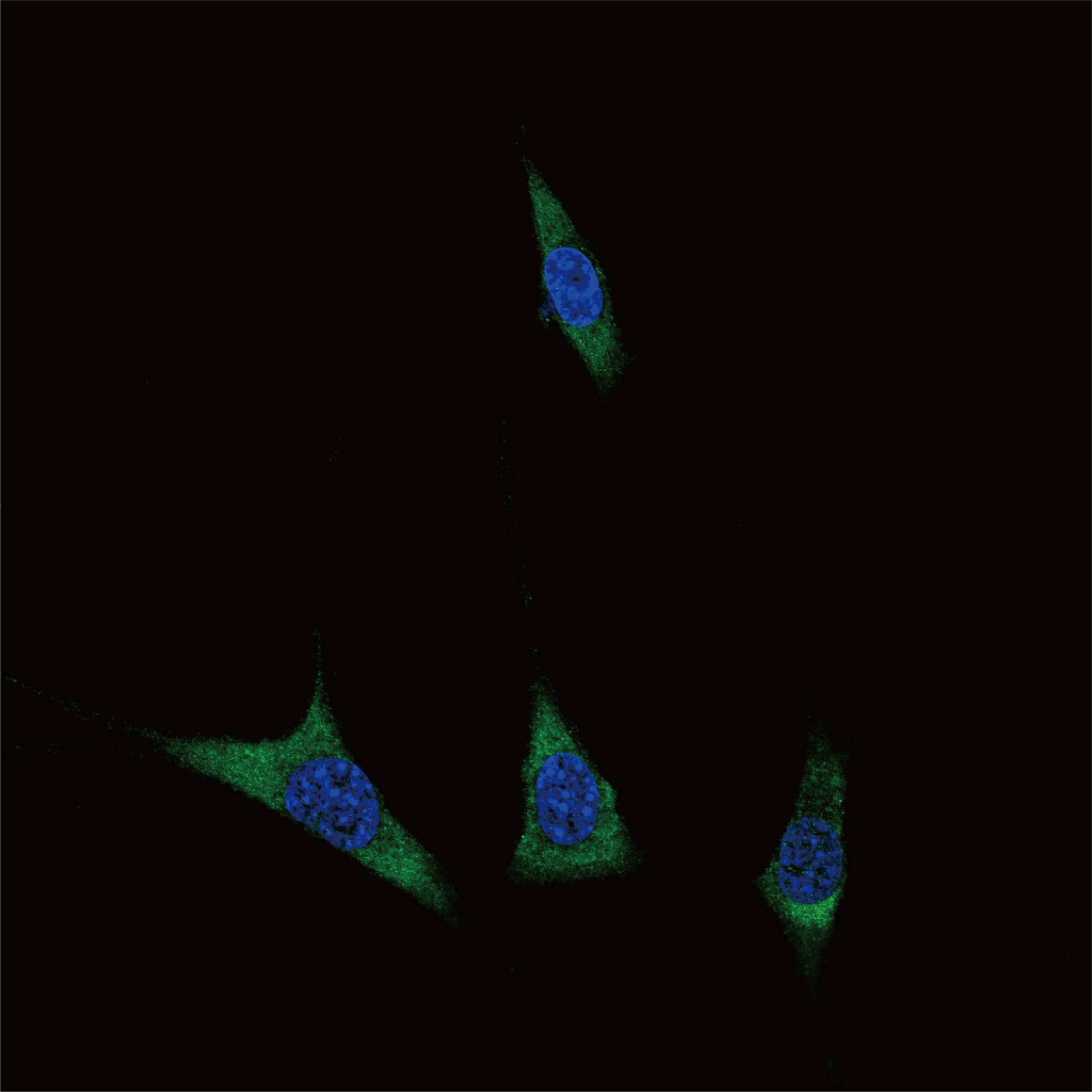

Supplement: Supplementary file 5 — Source data Fig. 3 [file 44321_2025_206_MOESM5_ESM.zip › Source Data Fig 3/Fig 3/3Q/KO-OGD-P-RIPK3-DAPI.tif]

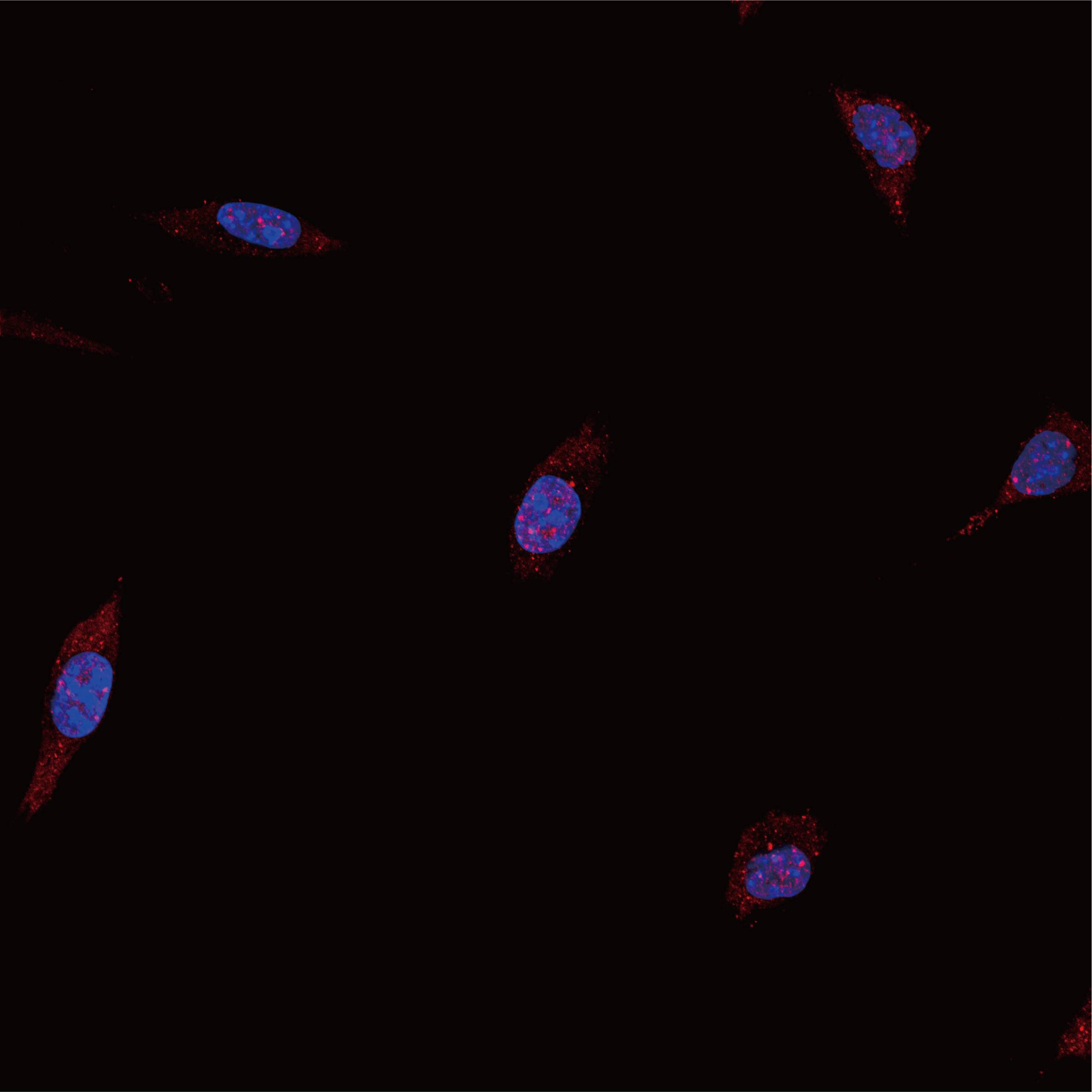

Supplement: Supplementary file 5 — Source data Fig. 3 [file 44321_2025_206_MOESM5_ESM.zip › Source Data Fig 3/Fig 3/3Q/WT-Normoxia-P-MLKL-DAPI.tif]

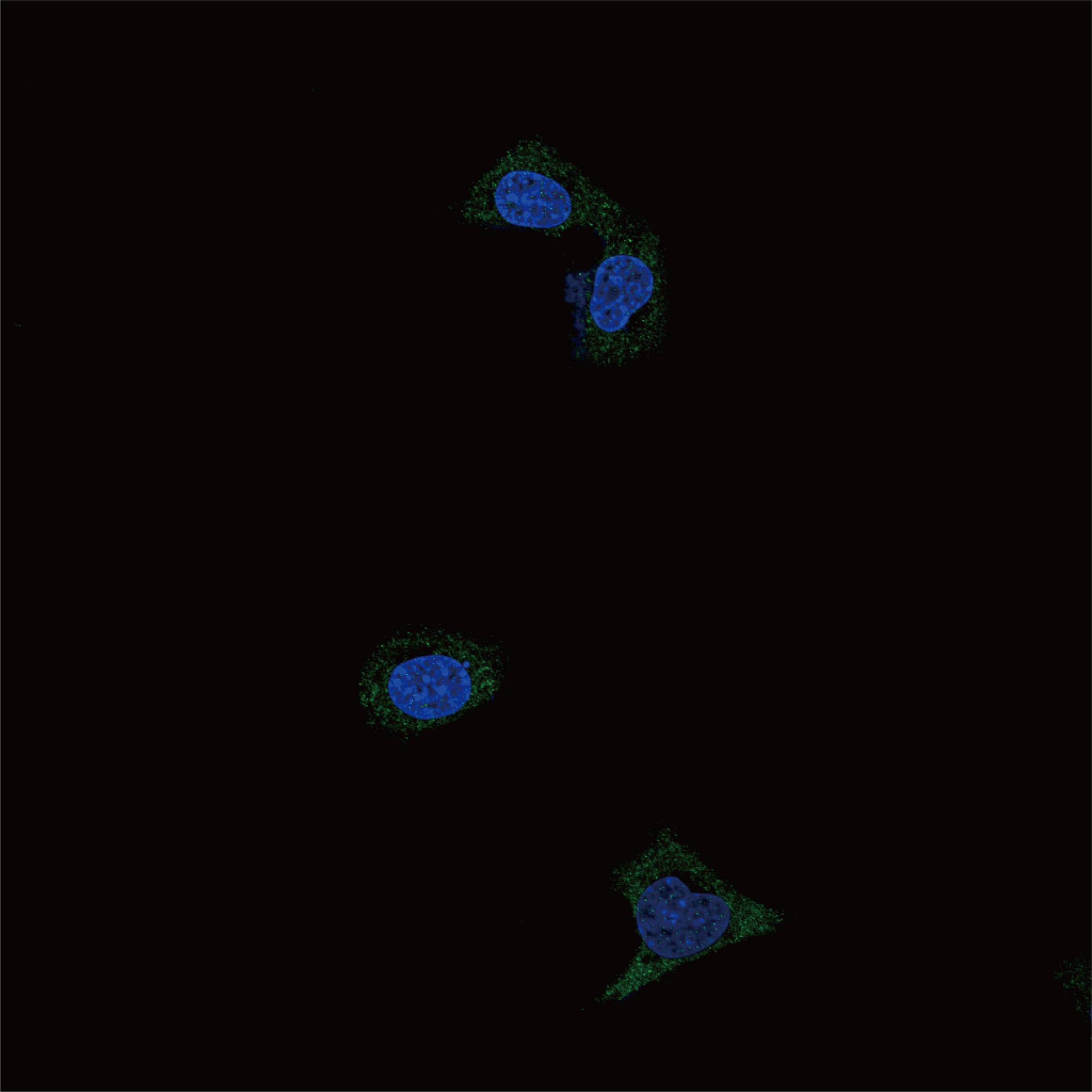

Supplement: Supplementary file 5 — Source data Fig. 3 [file 44321_2025_206_MOESM5_ESM.zip › Source Data Fig 3/Fig 3/3Q/WT-Normoxia-P-RIPK3-DAPI.tif]

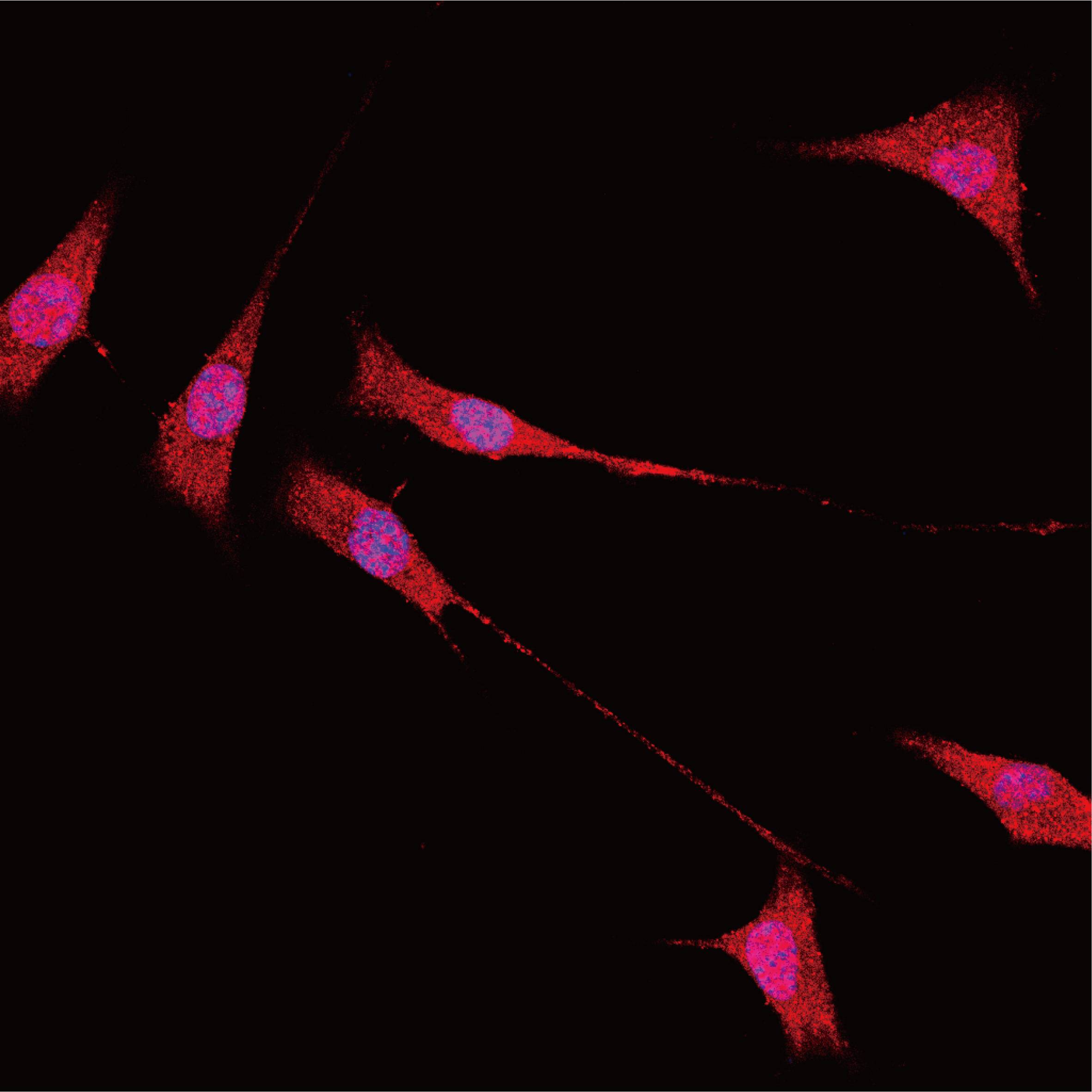

Supplement: Supplementary file 5 — Source data Fig. 3 [file 44321_2025_206_MOESM5_ESM.zip › Source Data Fig 3/Fig 3/3Q/WT-OGD-P-MLKL-DAPI.tif]

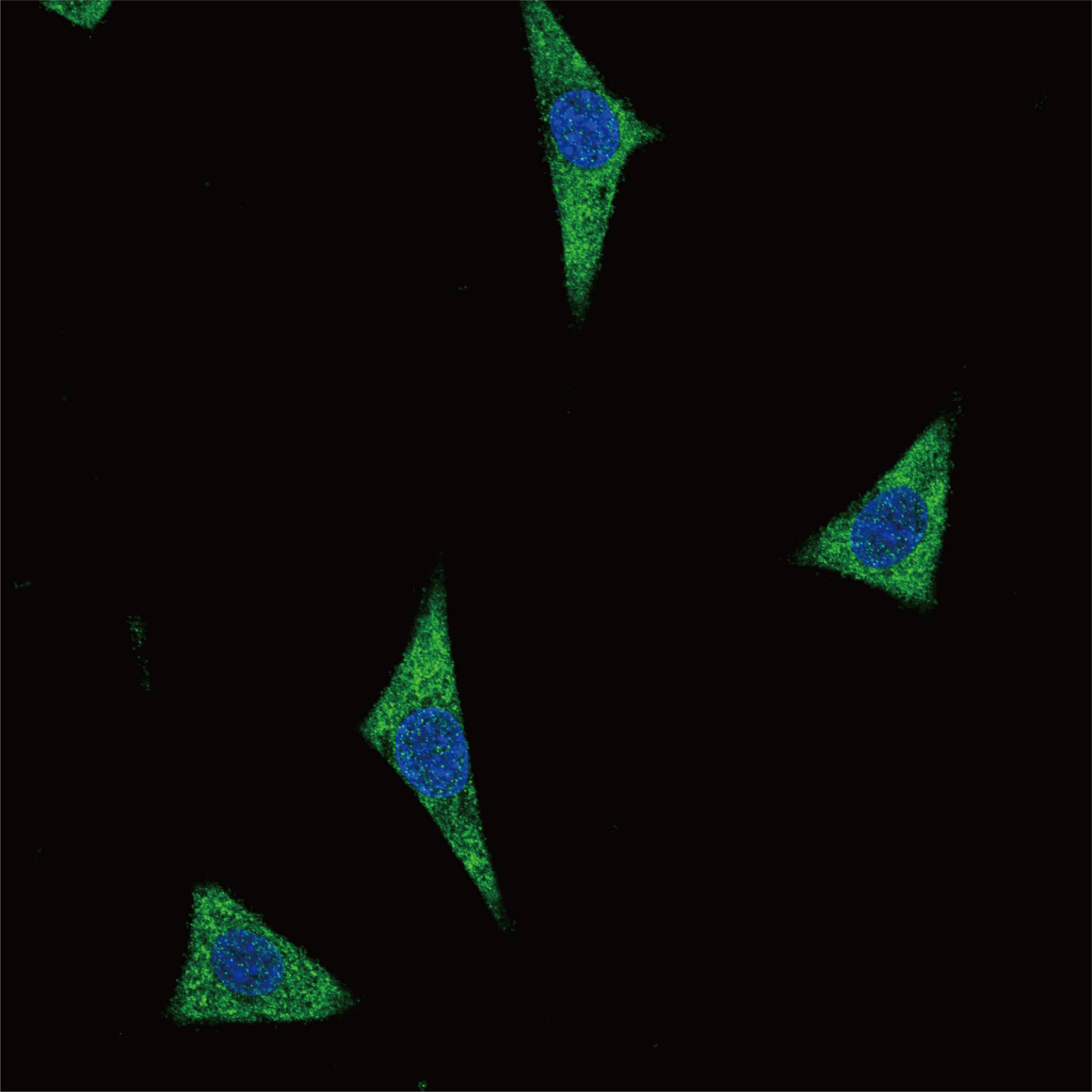

Supplement: Supplementary file 5 — Source data Fig. 3 [file 44321_2025_206_MOESM5_ESM.zip › Source Data Fig 3/Fig 3/3Q/WT-OGD-P-RIPK3-DAPI.tif]

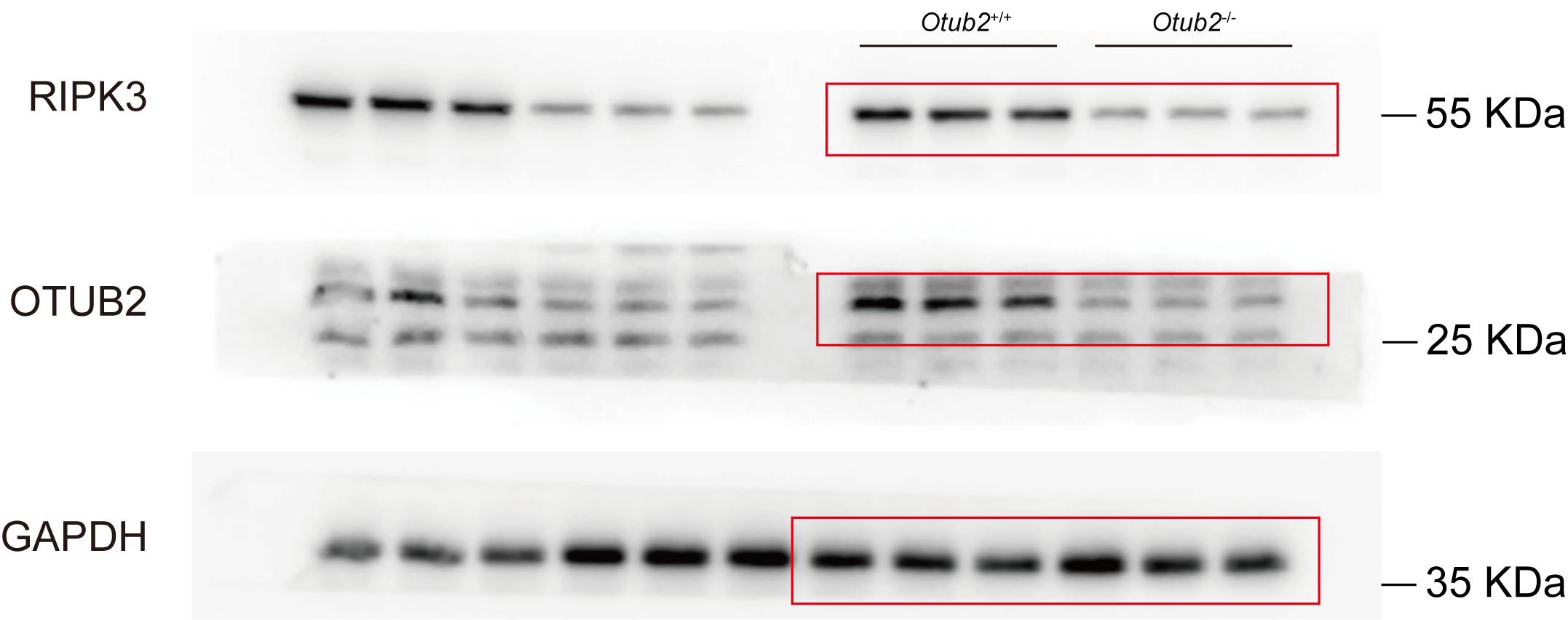

Supplement: Supplementary file 6 — Source data Fig. 4 [file 44321_2025_206_MOESM6_ESM.zip › Source Data Fig 4/Fig 4/4A/4A.tif]

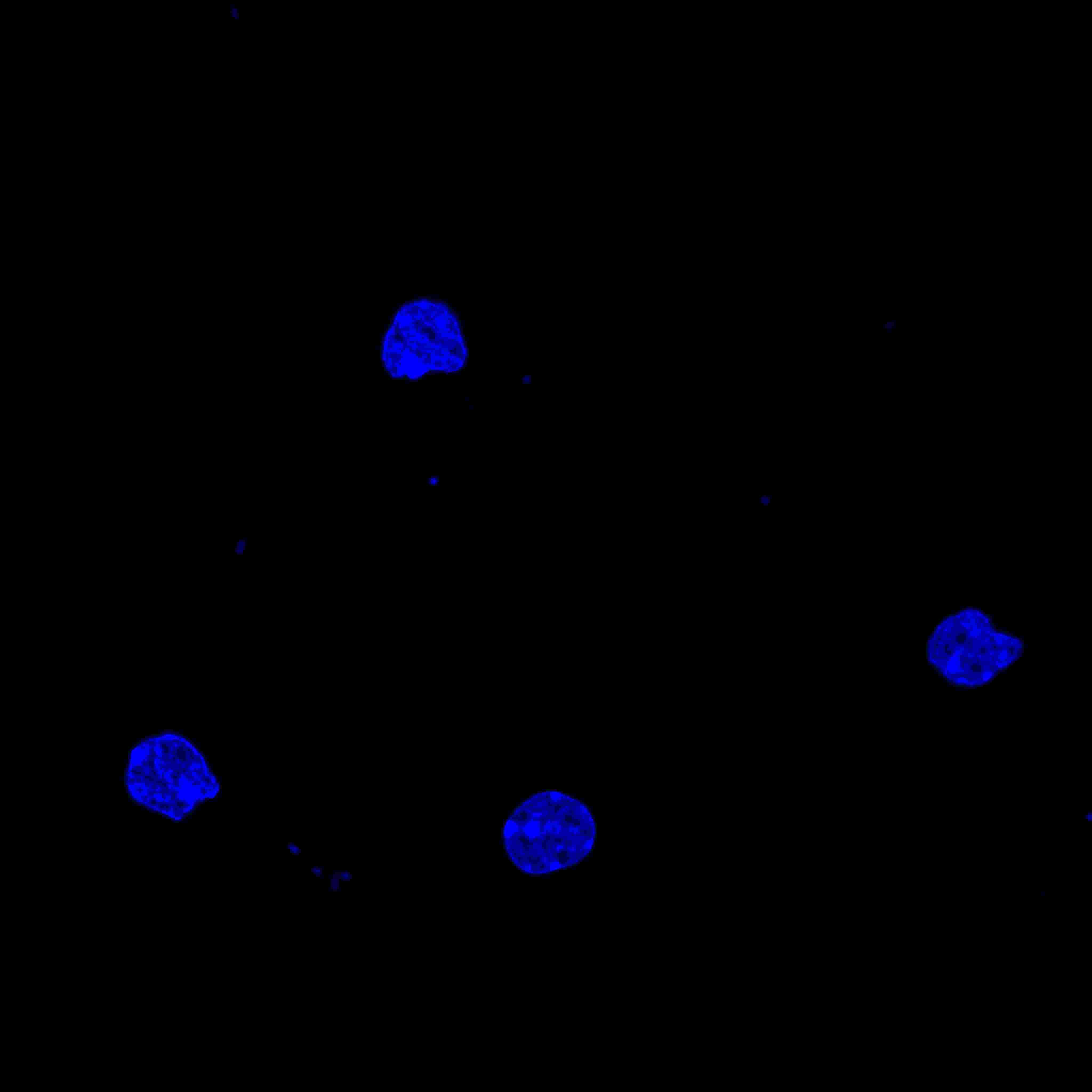

Supplement: Supplementary file 6 — Source data Fig. 4 [file 44321_2025_206_MOESM6_ESM.zip › Source Data Fig 4/Fig 4/4D/KO DAPI.tif]

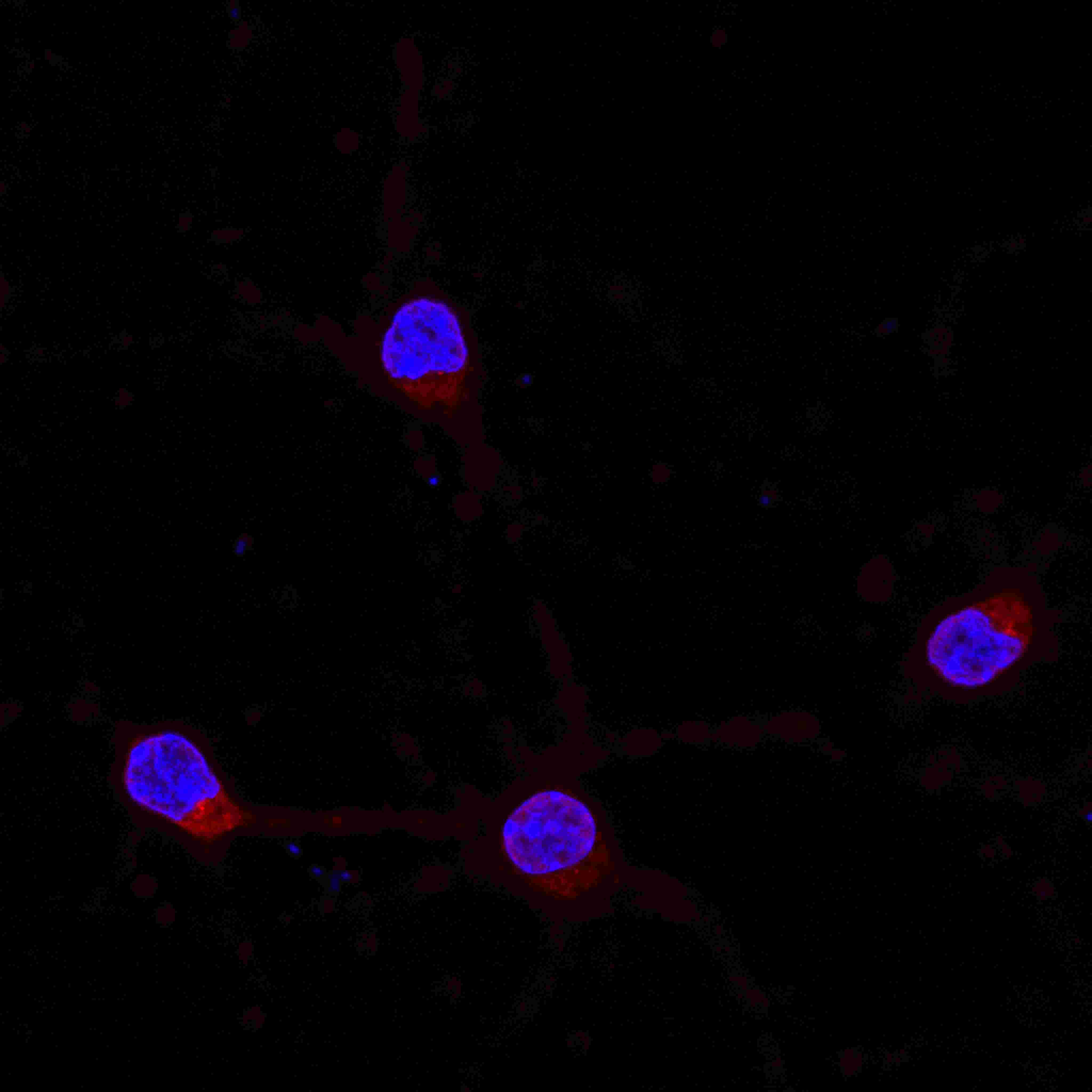

Supplement: Supplementary file 6 — Source data Fig. 4 [file 44321_2025_206_MOESM6_ESM.zip › Source Data Fig 4/Fig 4/4D/KO Merge.tif]

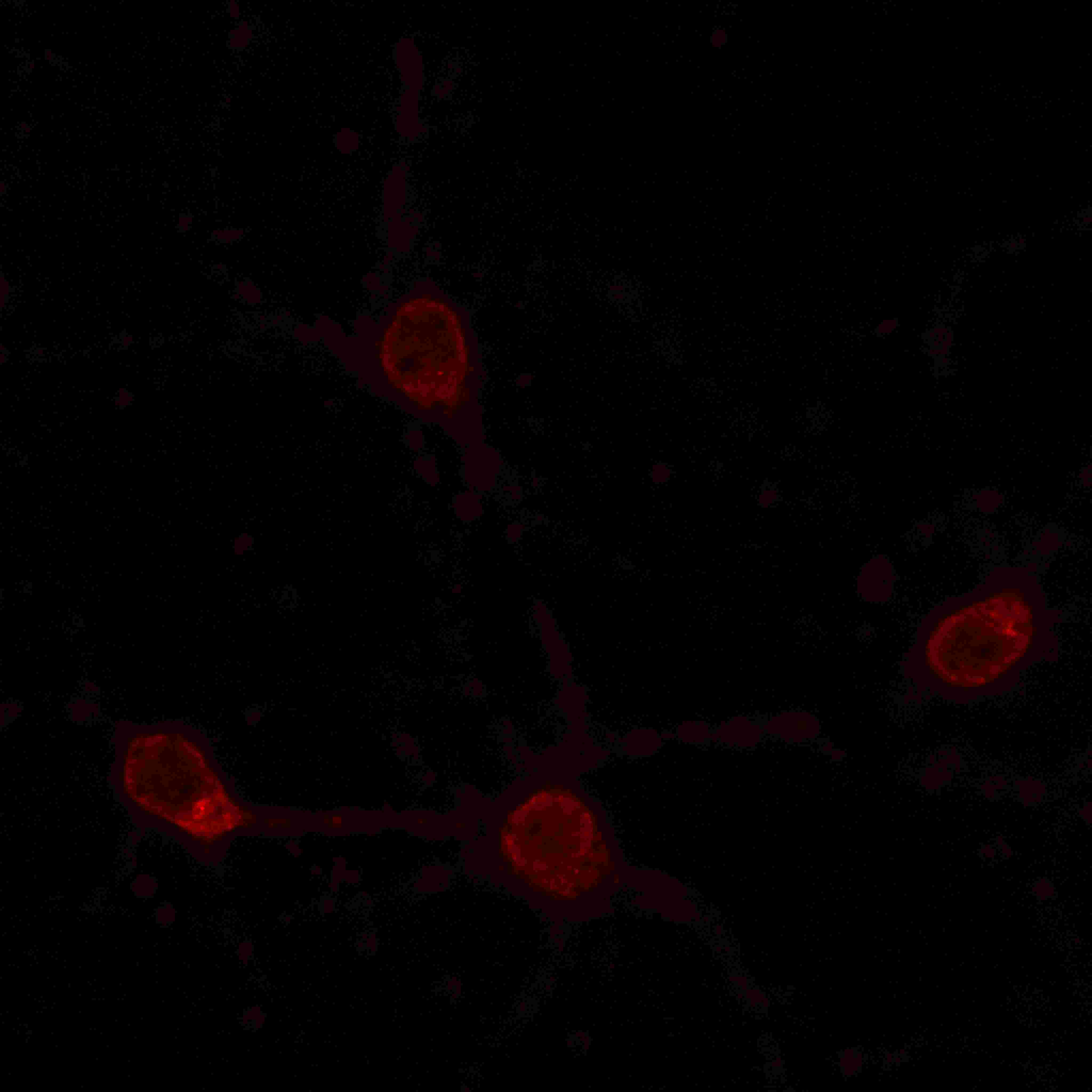

Supplement: Supplementary file 6 — Source data Fig. 4 [file 44321_2025_206_MOESM6_ESM.zip › Source Data Fig 4/Fig 4/4D/KO RIPK3.tif]

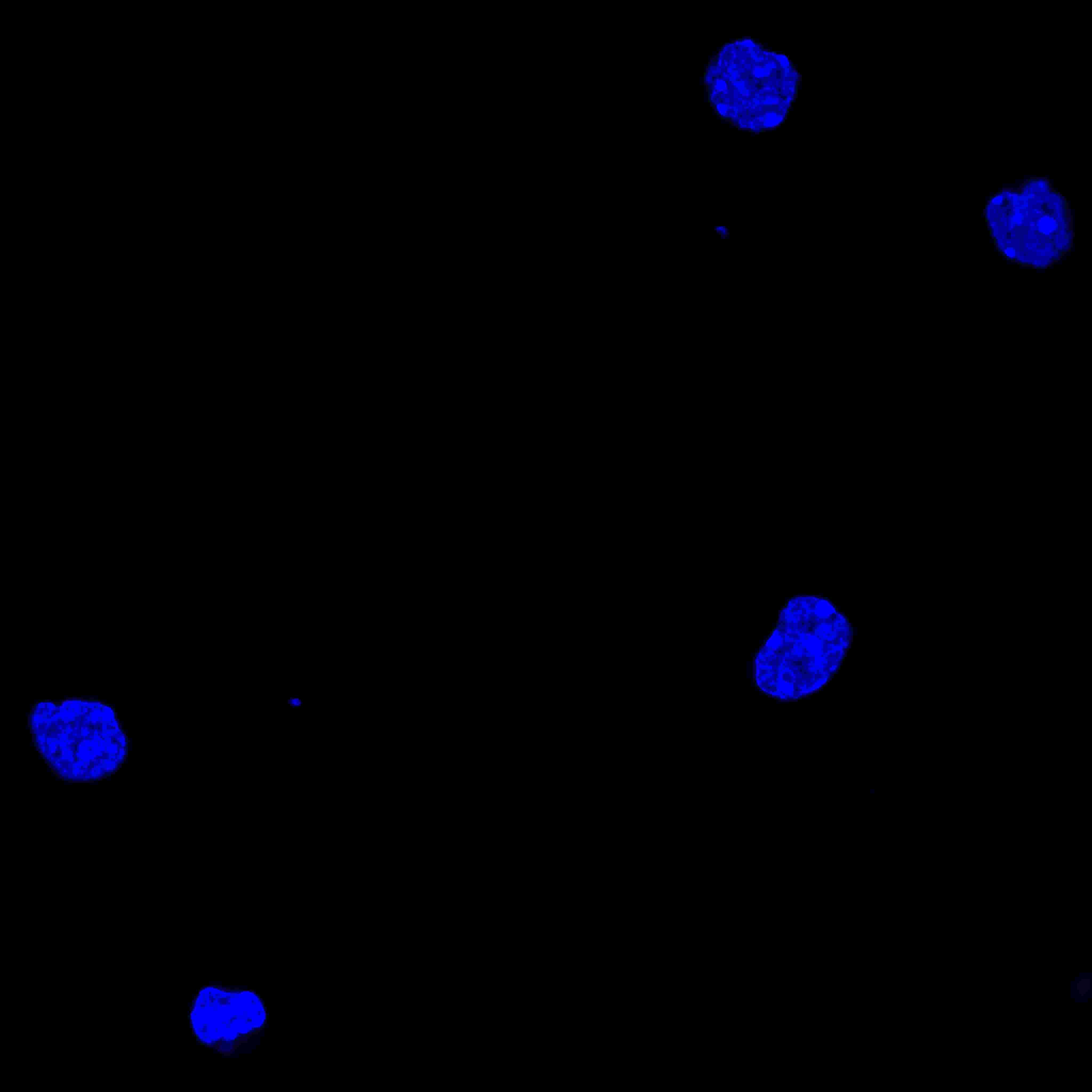

Supplement: Supplementary file 6 — Source data Fig. 4 [file 44321_2025_206_MOESM6_ESM.zip › Source Data Fig 4/Fig 4/4D/WT DAPI.tif]

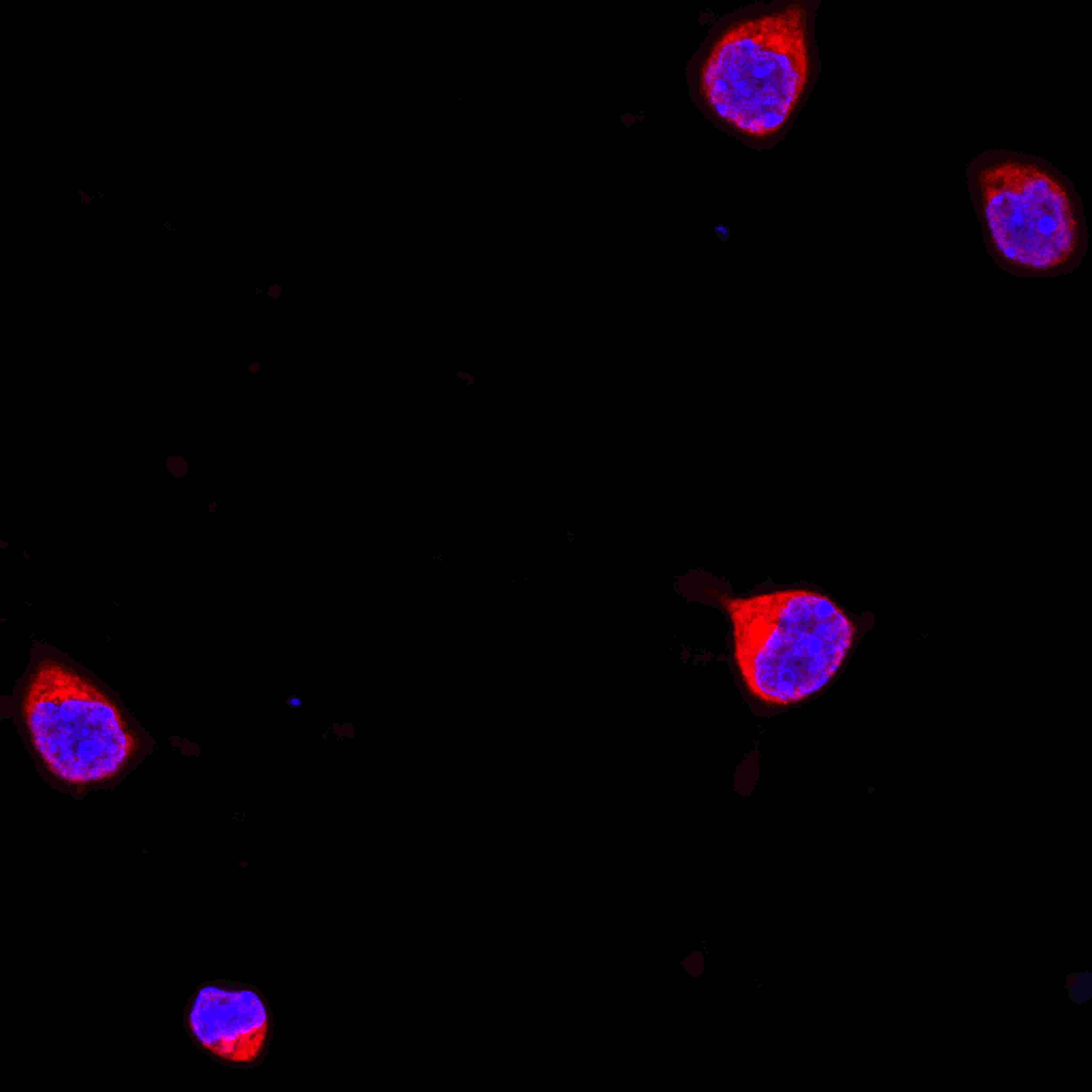

Supplement: Supplementary file 6 — Source data Fig. 4 [file 44321_2025_206_MOESM6_ESM.zip › Source Data Fig 4/Fig 4/4D/WT Merge.tif]

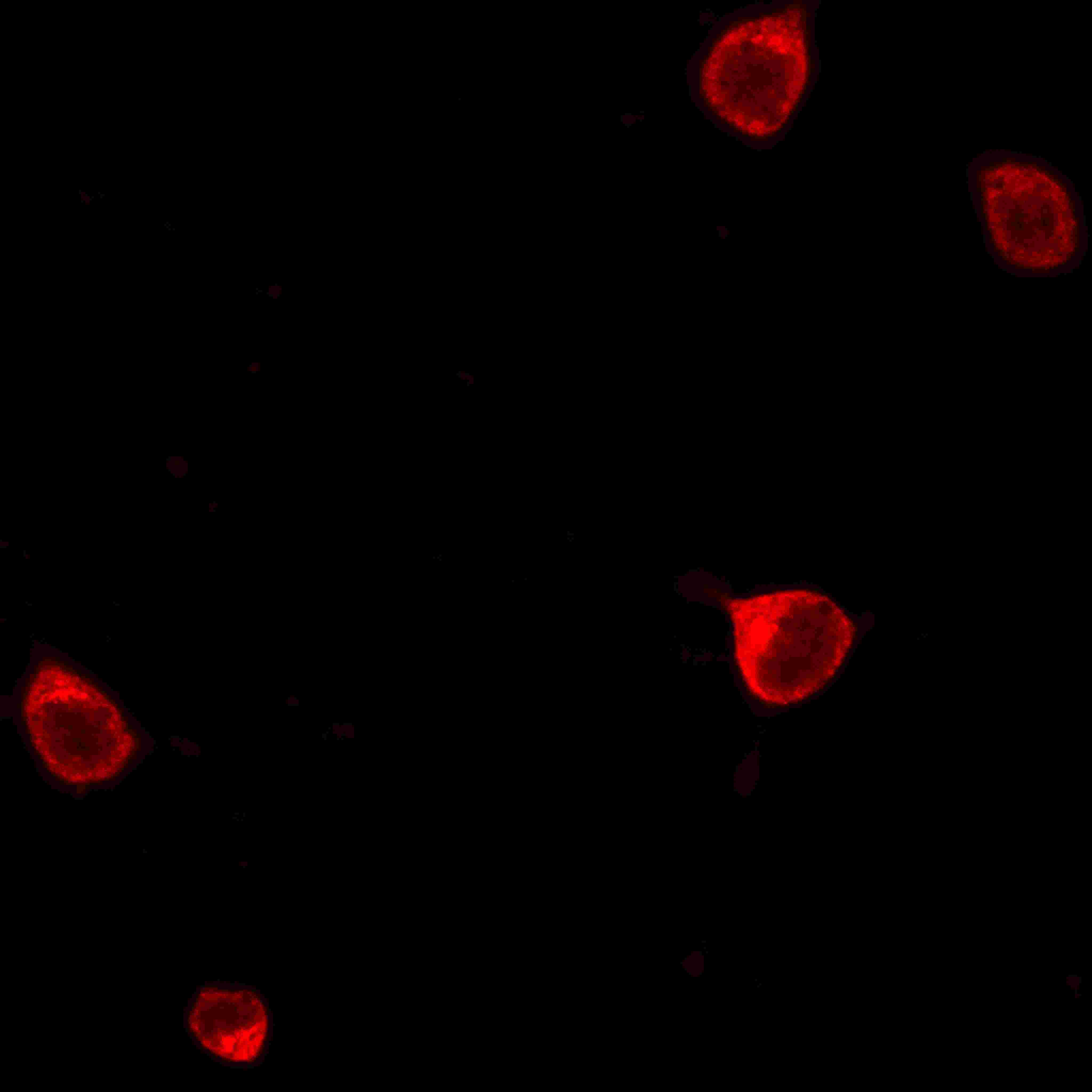

Supplement: Supplementary file 6 — Source data Fig. 4 [file 44321_2025_206_MOESM6_ESM.zip › Source Data Fig 4/Fig 4/4D/WT RIPK3.tif]

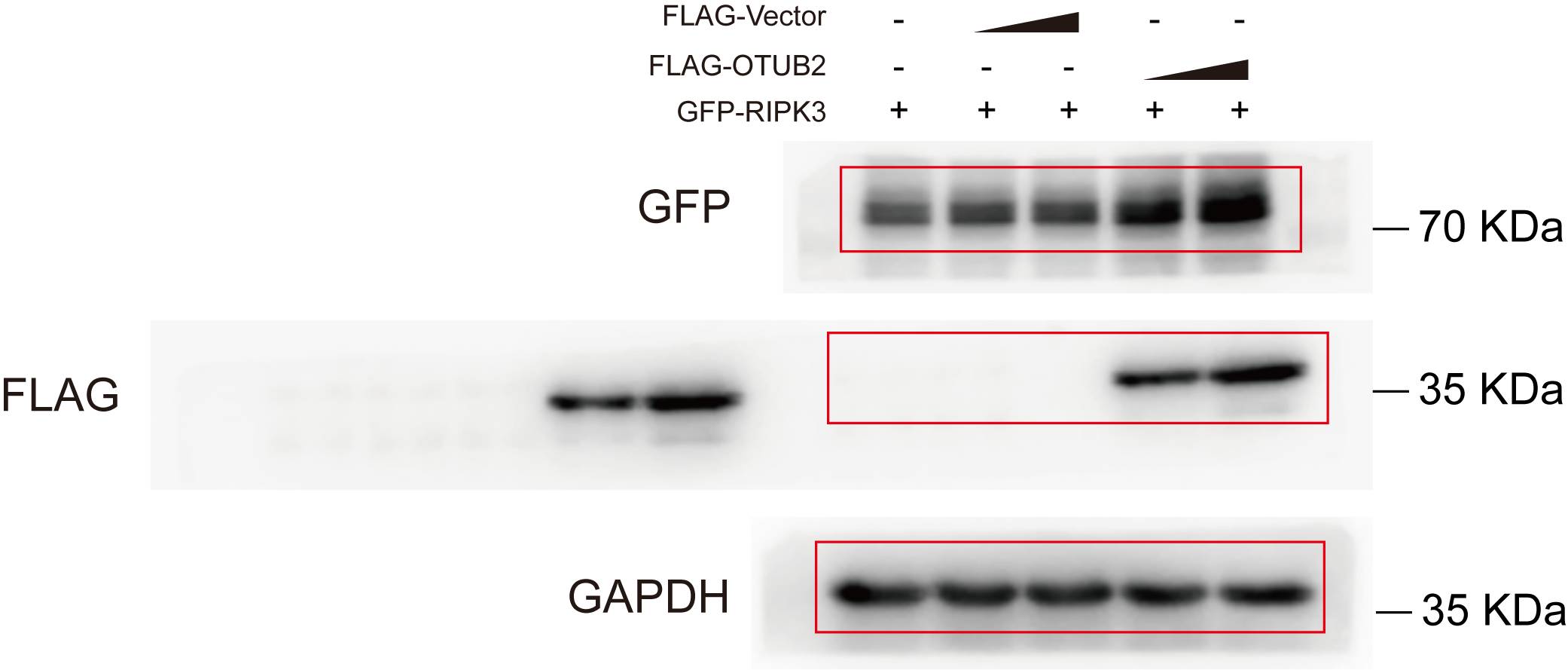

Supplement: Supplementary file 6 — Source data Fig. 4 [file 44321_2025_206_MOESM6_ESM.zip › Source Data Fig 4/Fig 4/4E/4E.tif]

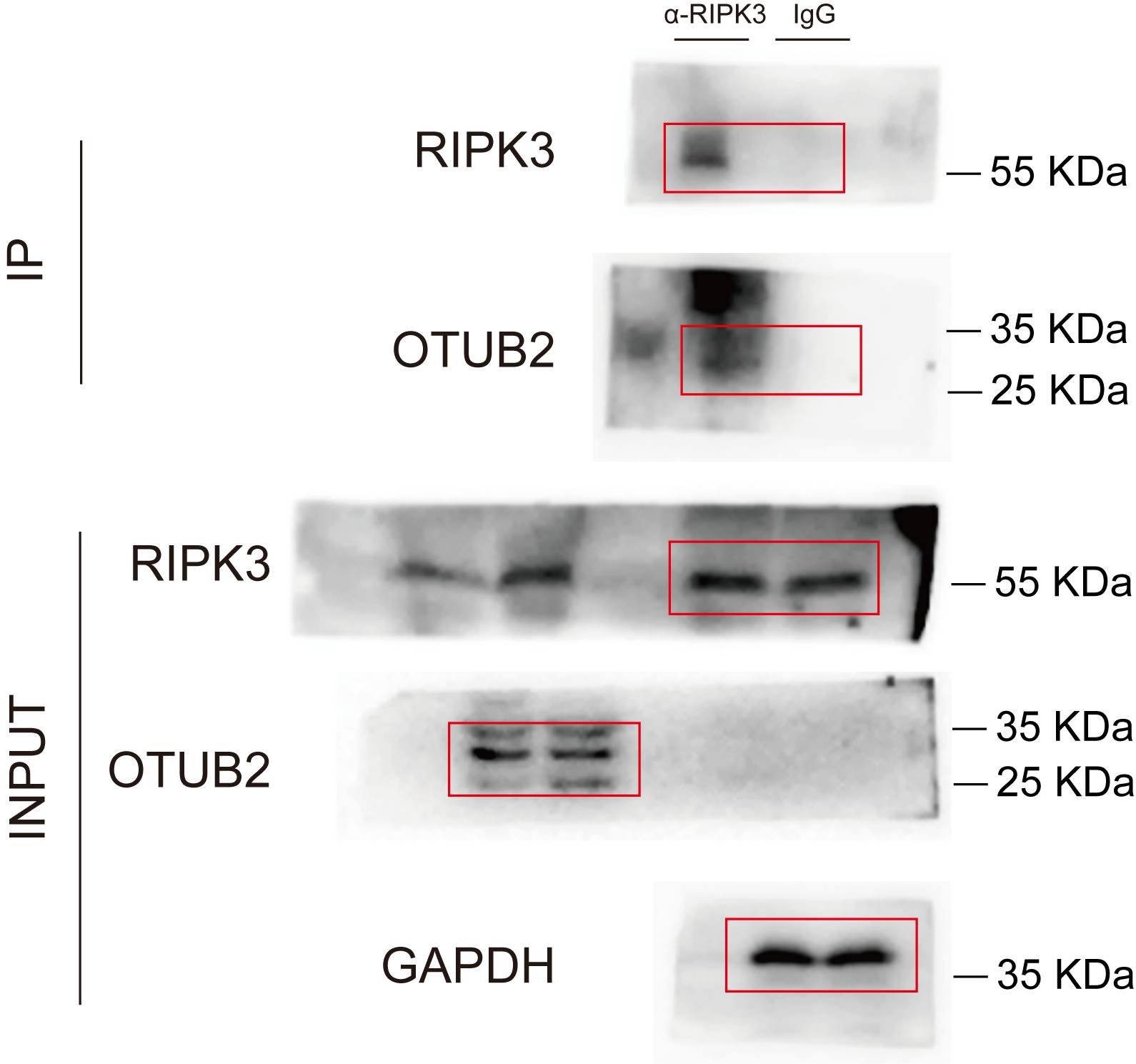

Supplement: Supplementary file 6 — Source data Fig. 4 [file 44321_2025_206_MOESM6_ESM.zip › Source Data Fig 4/Fig 4/4F/4F.tif]

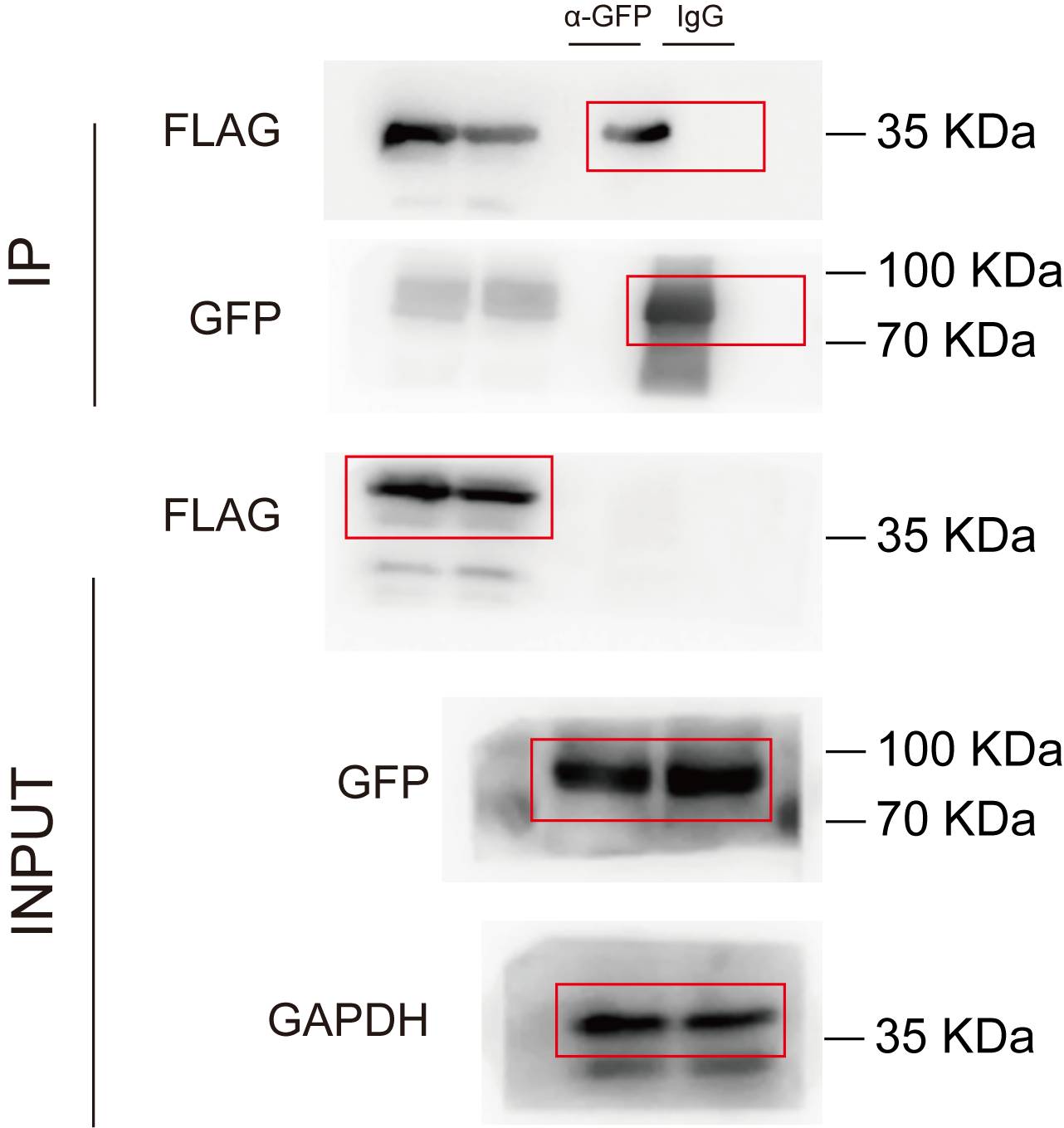

Supplement: Supplementary file 6 — Source data Fig. 4 [file 44321_2025_206_MOESM6_ESM.zip › Source Data Fig 4/Fig 4/4G/4G.tif]

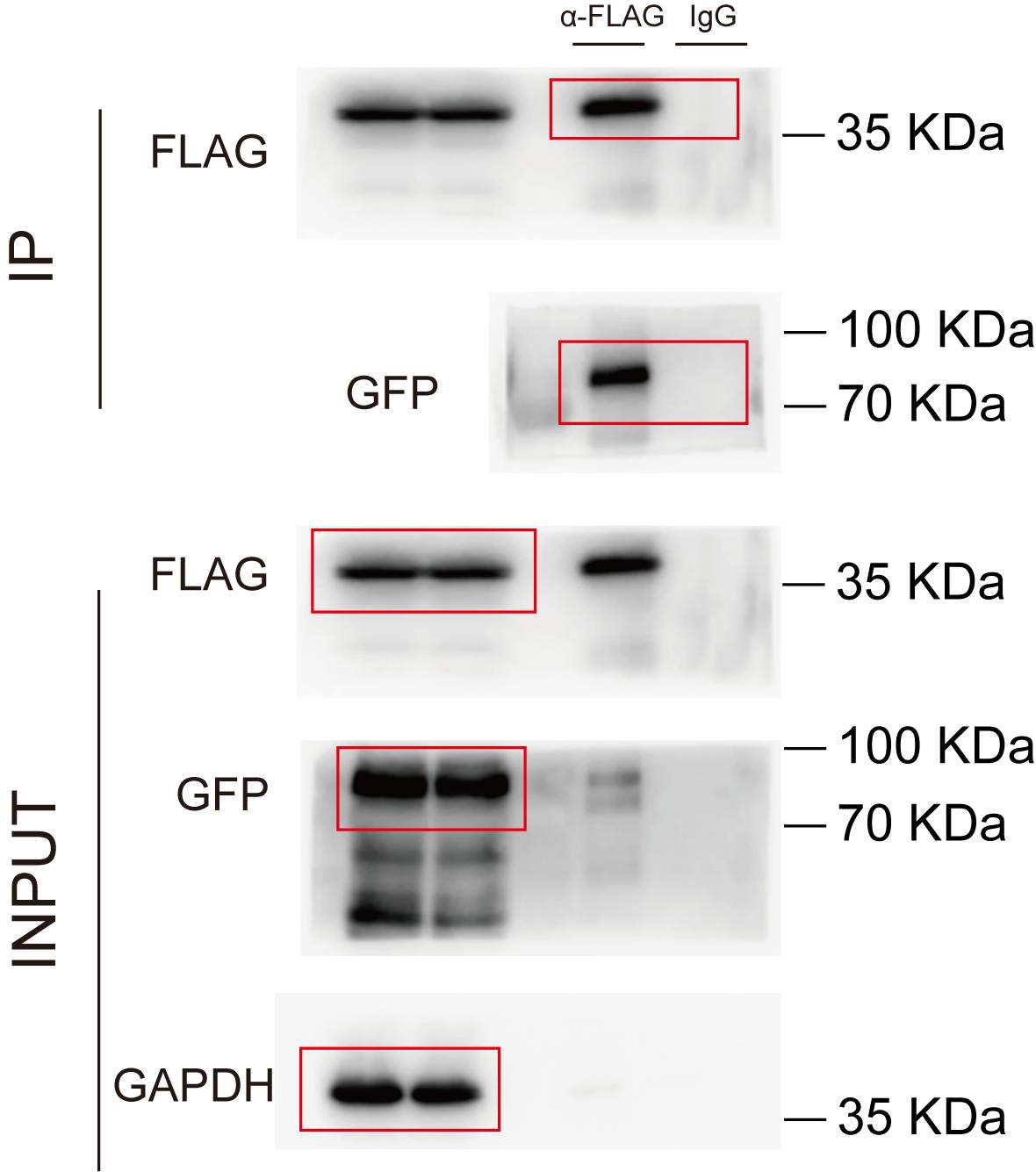

Supplement: Supplementary file 6 — Source data Fig. 4 [file 44321_2025_206_MOESM6_ESM.zip › Source Data Fig 4/Fig 4/4H/4H.tif]

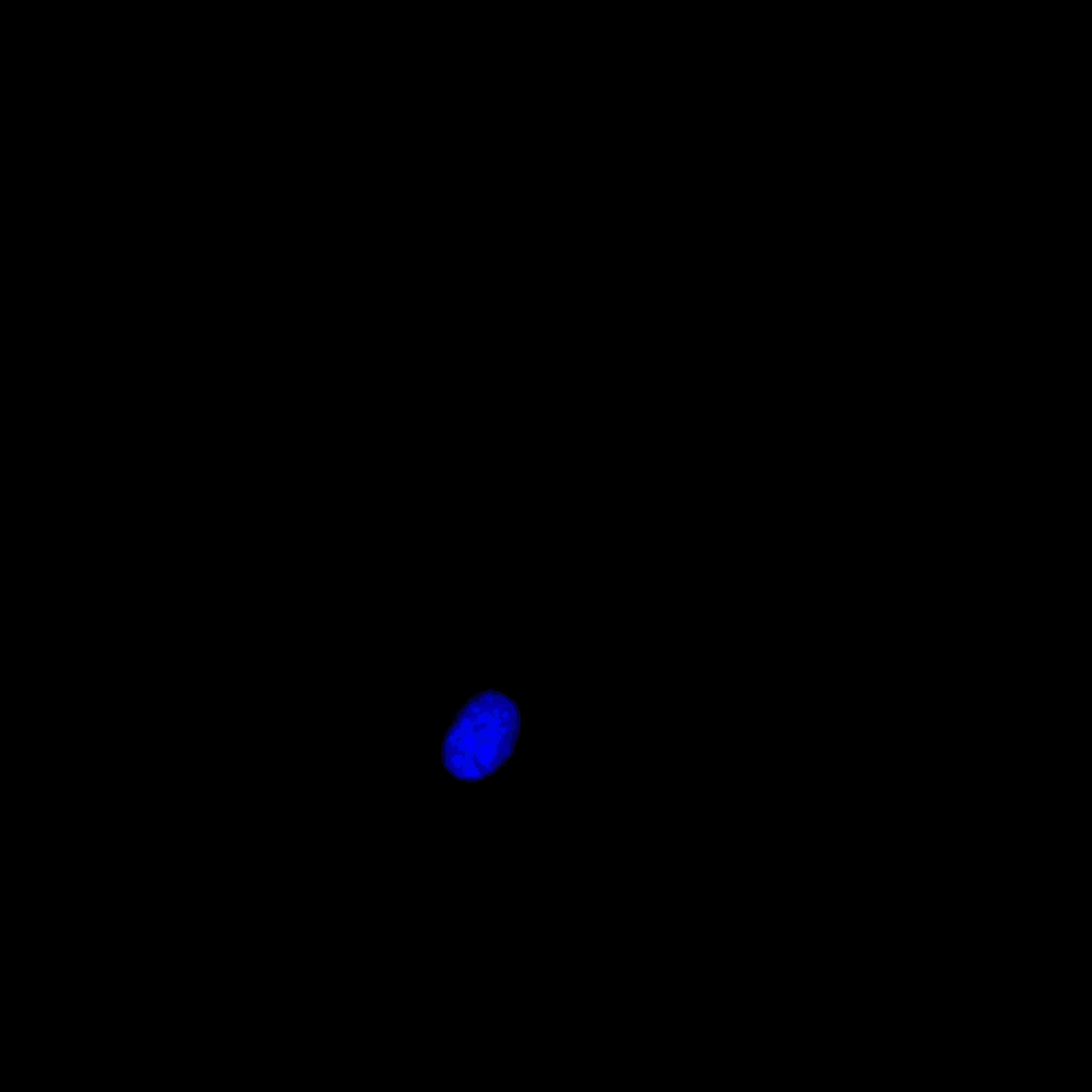

Supplement: Supplementary file 6 — Source data Fig. 4 [file 44321_2025_206_MOESM6_ESM.zip › Source Data Fig 4/Fig 4/4I/DAPI.tif]

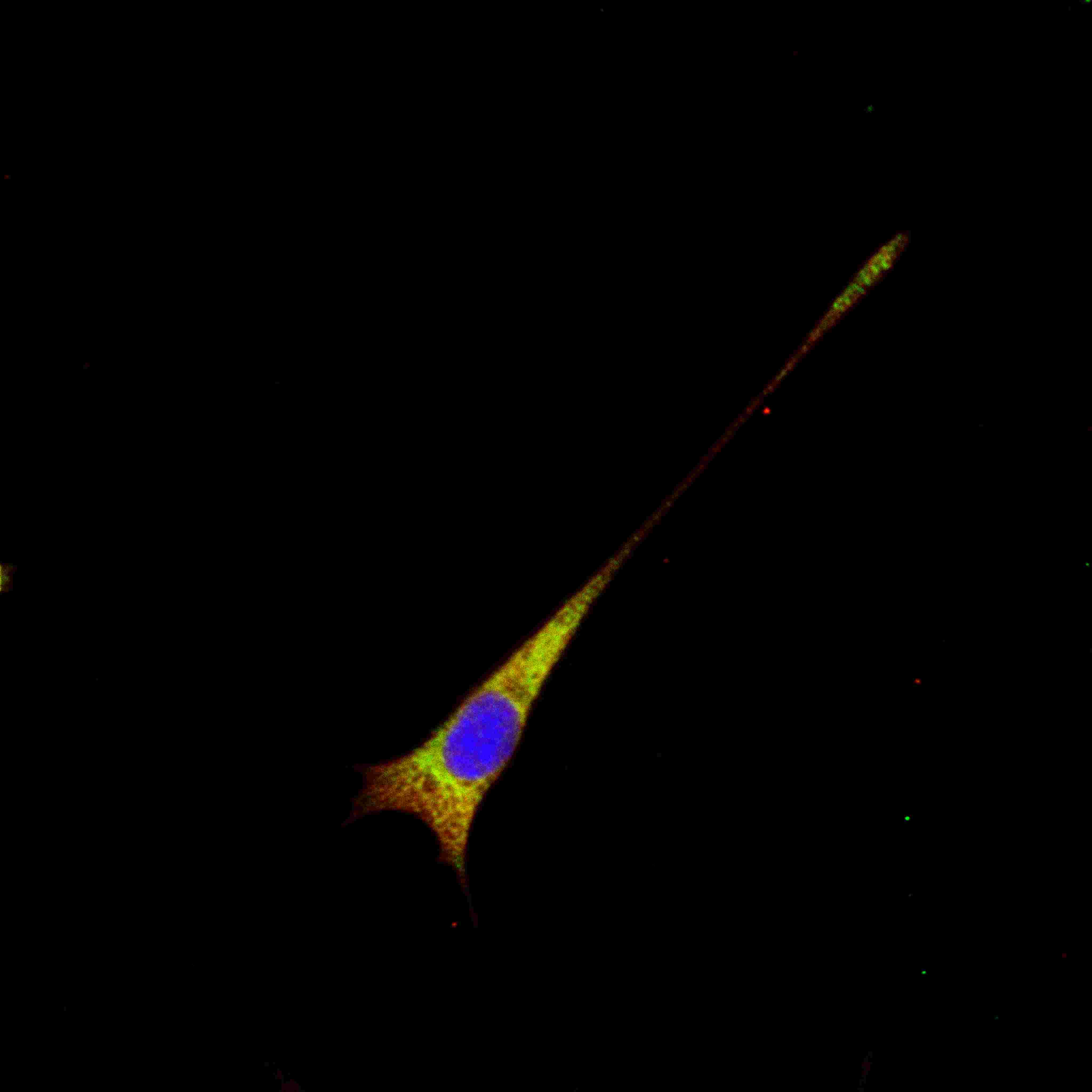

Supplement: Supplementary file 6 — Source data Fig. 4 [file 44321_2025_206_MOESM6_ESM.zip › Source Data Fig 4/Fig 4/4I/Merge.tif]

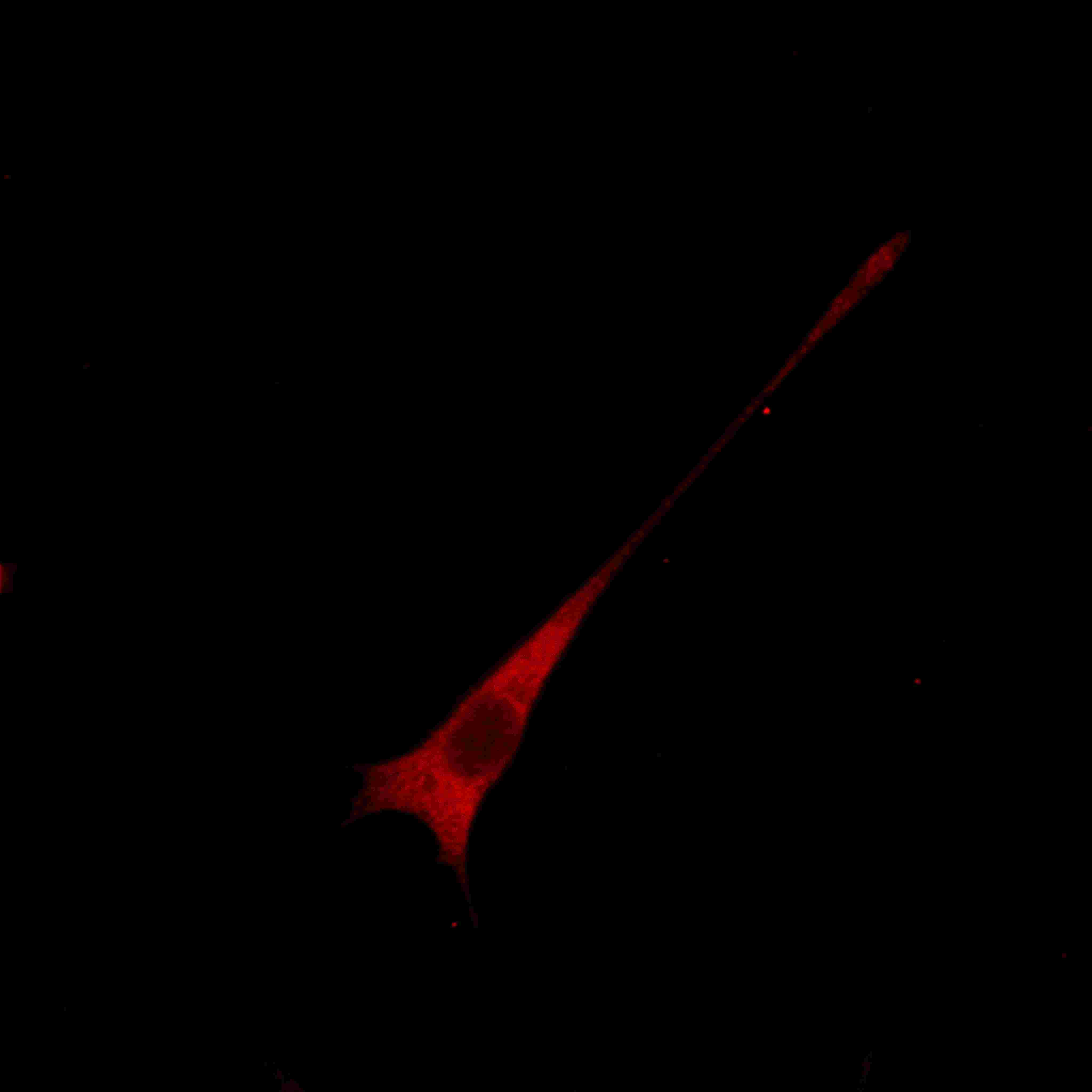

Supplement: Supplementary file 6 — Source data Fig. 4 [file 44321_2025_206_MOESM6_ESM.zip › Source Data Fig 4/Fig 4/4I/OTUB2.tif]

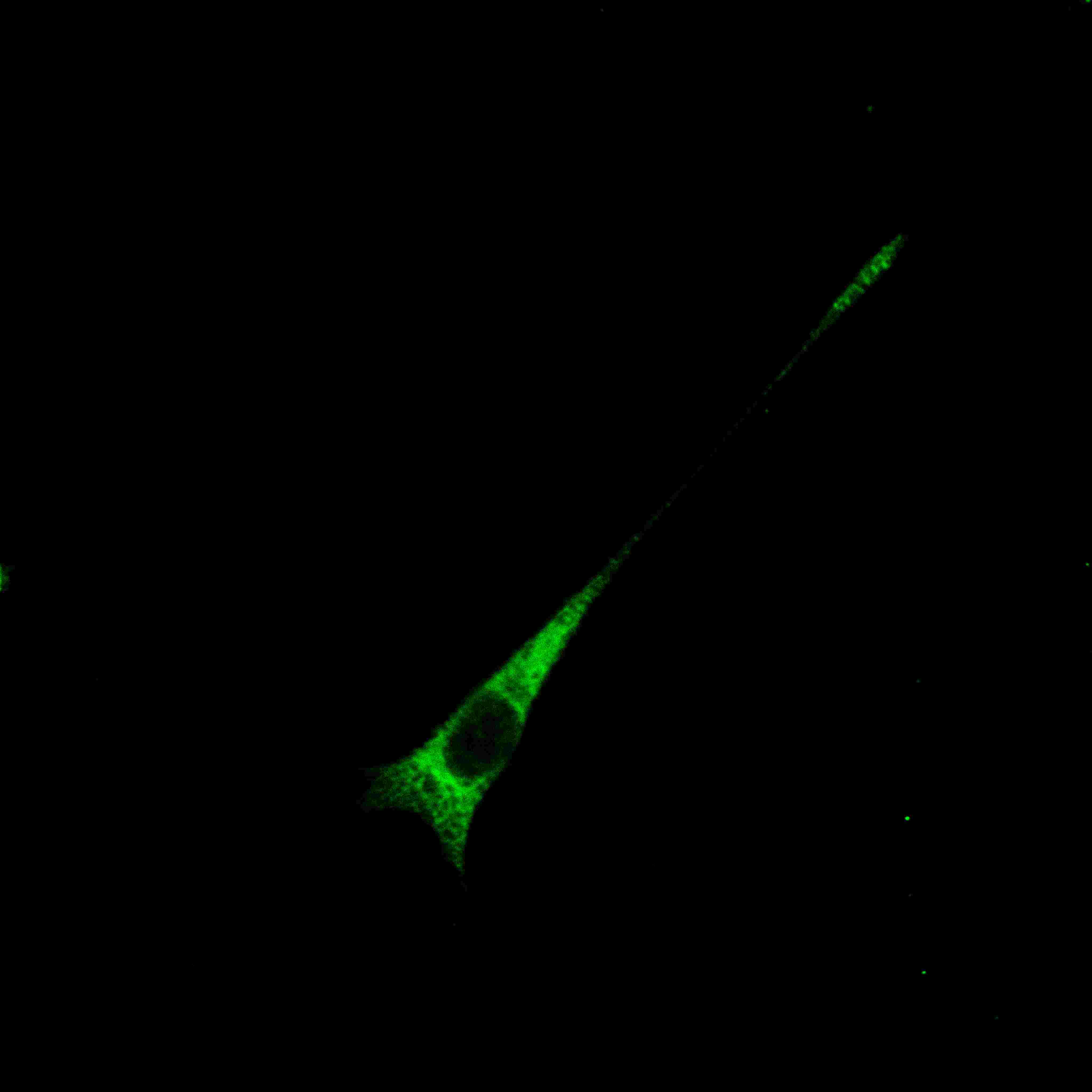

Supplement: Supplementary file 6 — Source data Fig. 4 [file 44321_2025_206_MOESM6_ESM.zip › Source Data Fig 4/Fig 4/4I/RIPK3.tif]

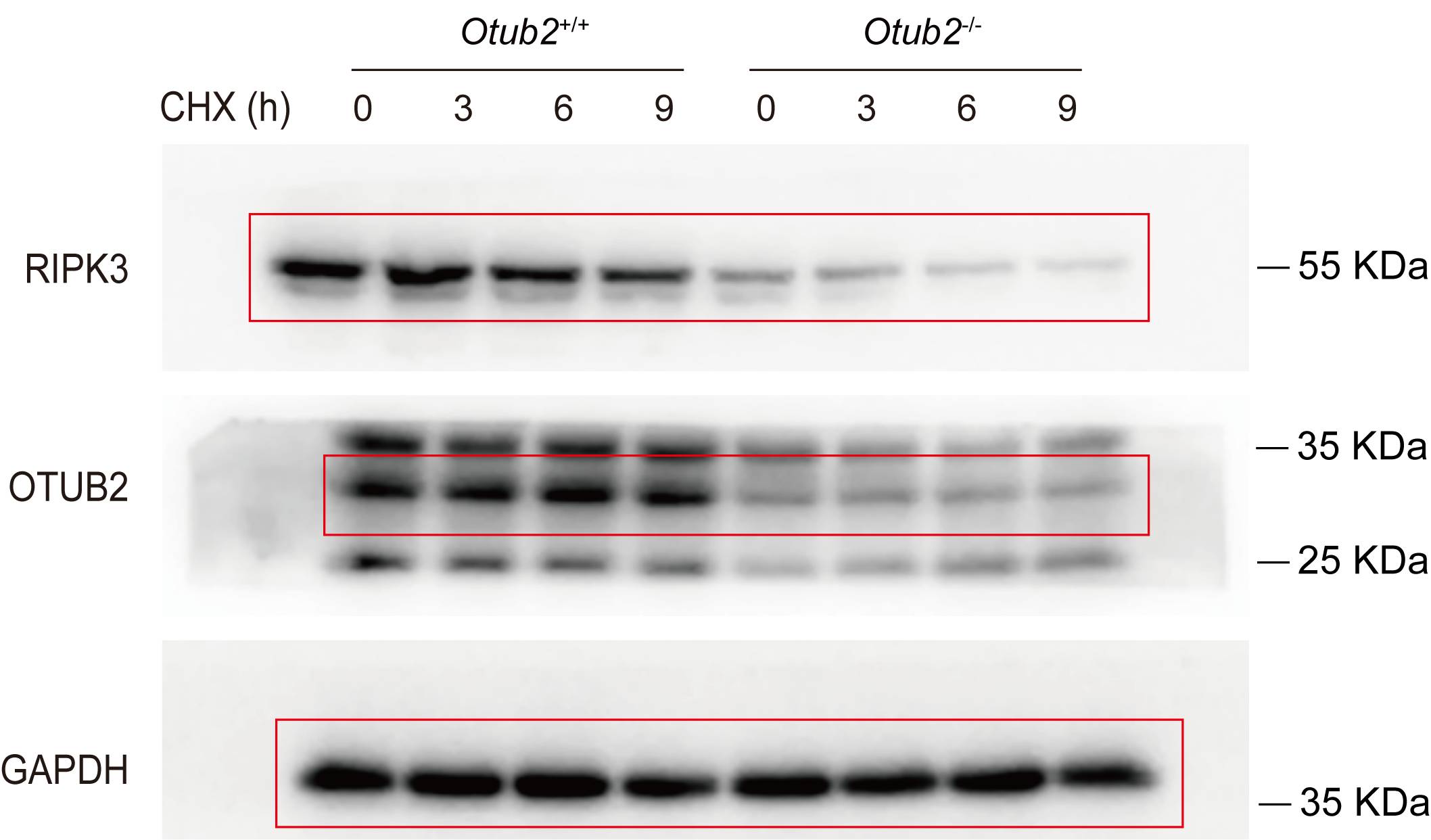

Supplement: Supplementary file 6 — Source data Fig. 4 [file 44321_2025_206_MOESM6_ESM.zip › Source Data Fig 4/Fig 4/4J/4J.tif]

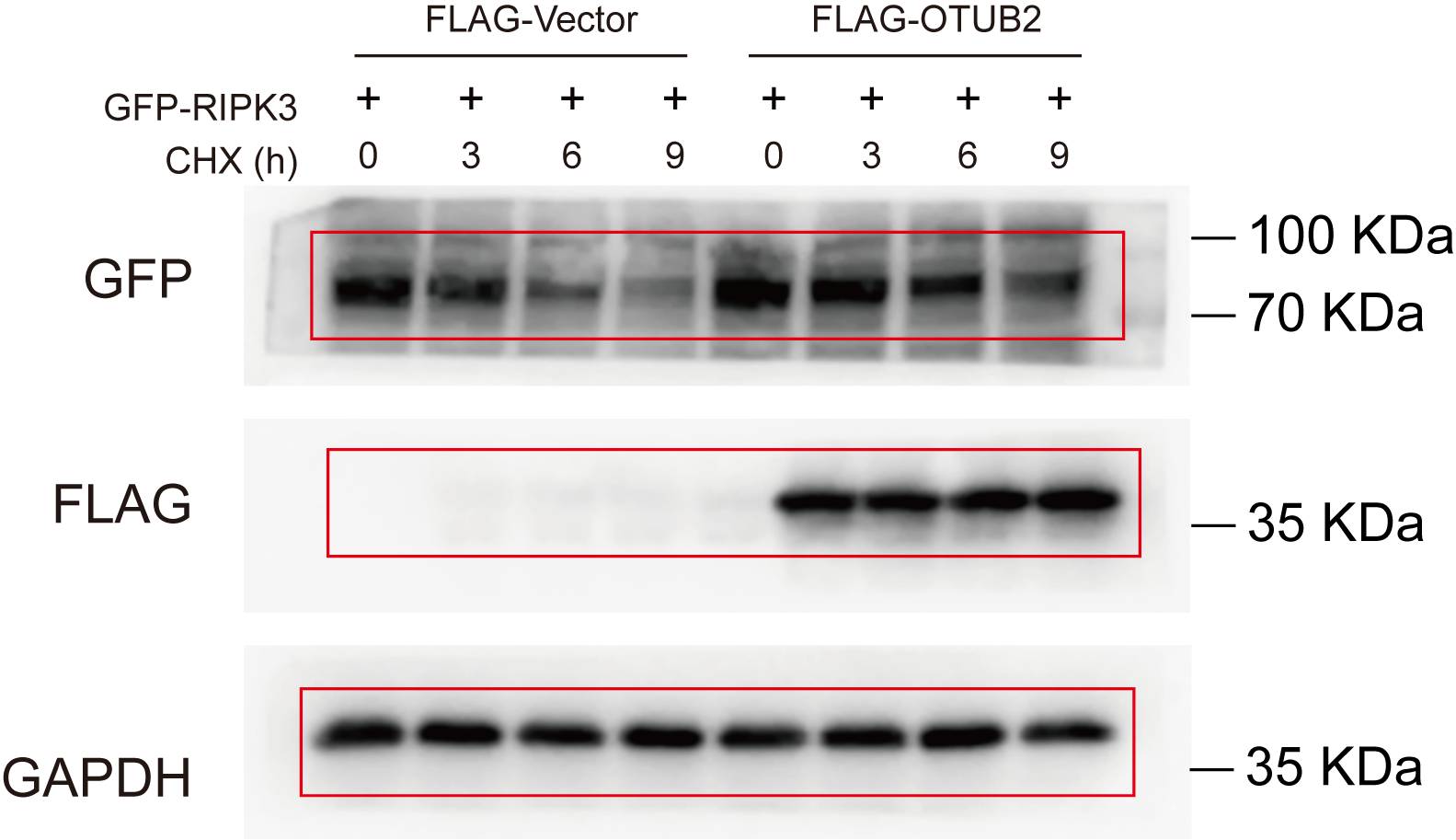

Supplement: Supplementary file 6 — Source data Fig. 4 [file 44321_2025_206_MOESM6_ESM.zip › Source Data Fig 4/Fig 4/4K/4K.tif]

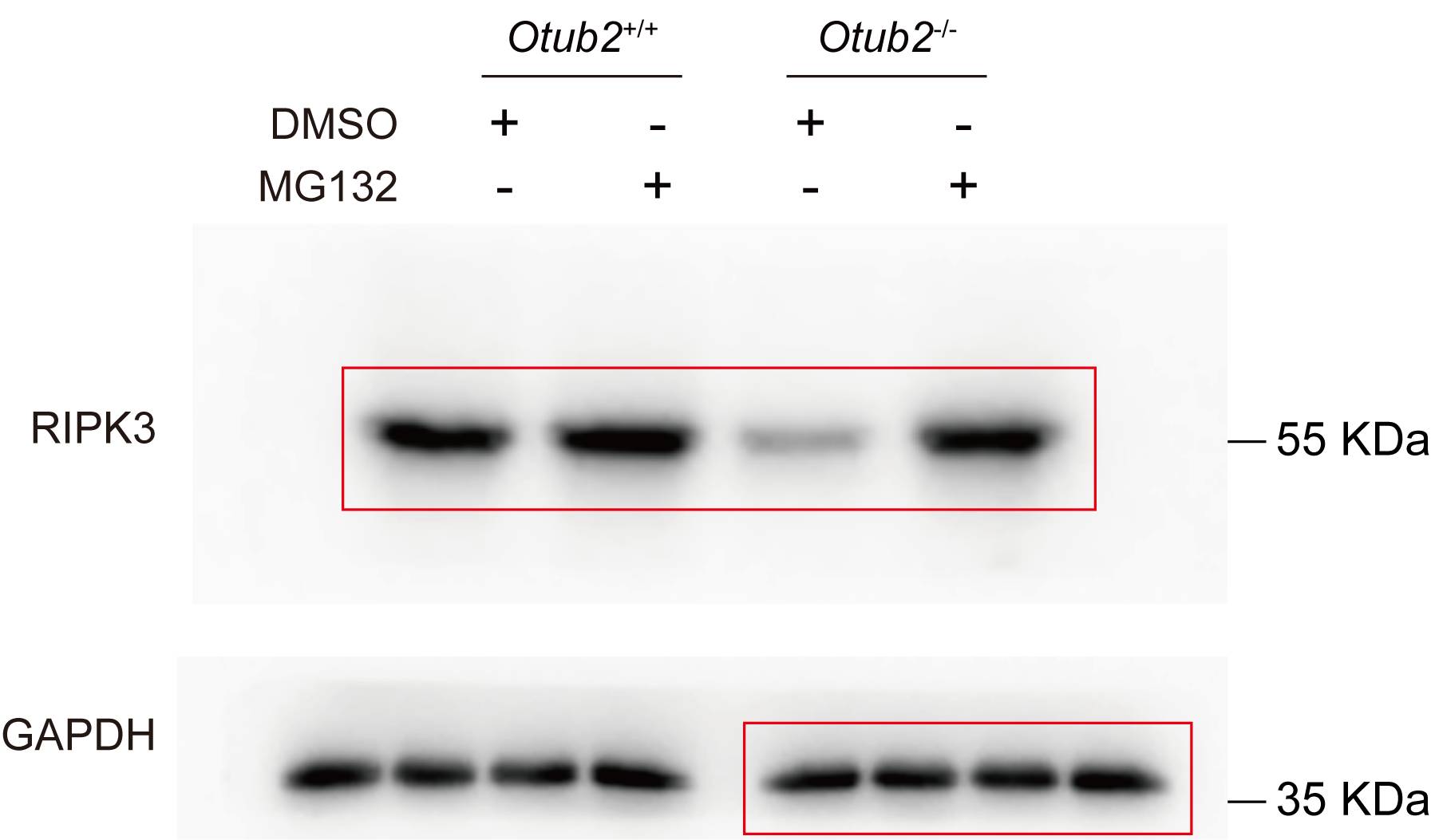

Supplement: Supplementary file 7 — Source data Fig. 5 [file 44321_2025_206_MOESM7_ESM.zip › Source data Fig 5/Fig 5/5A/5A.tif]

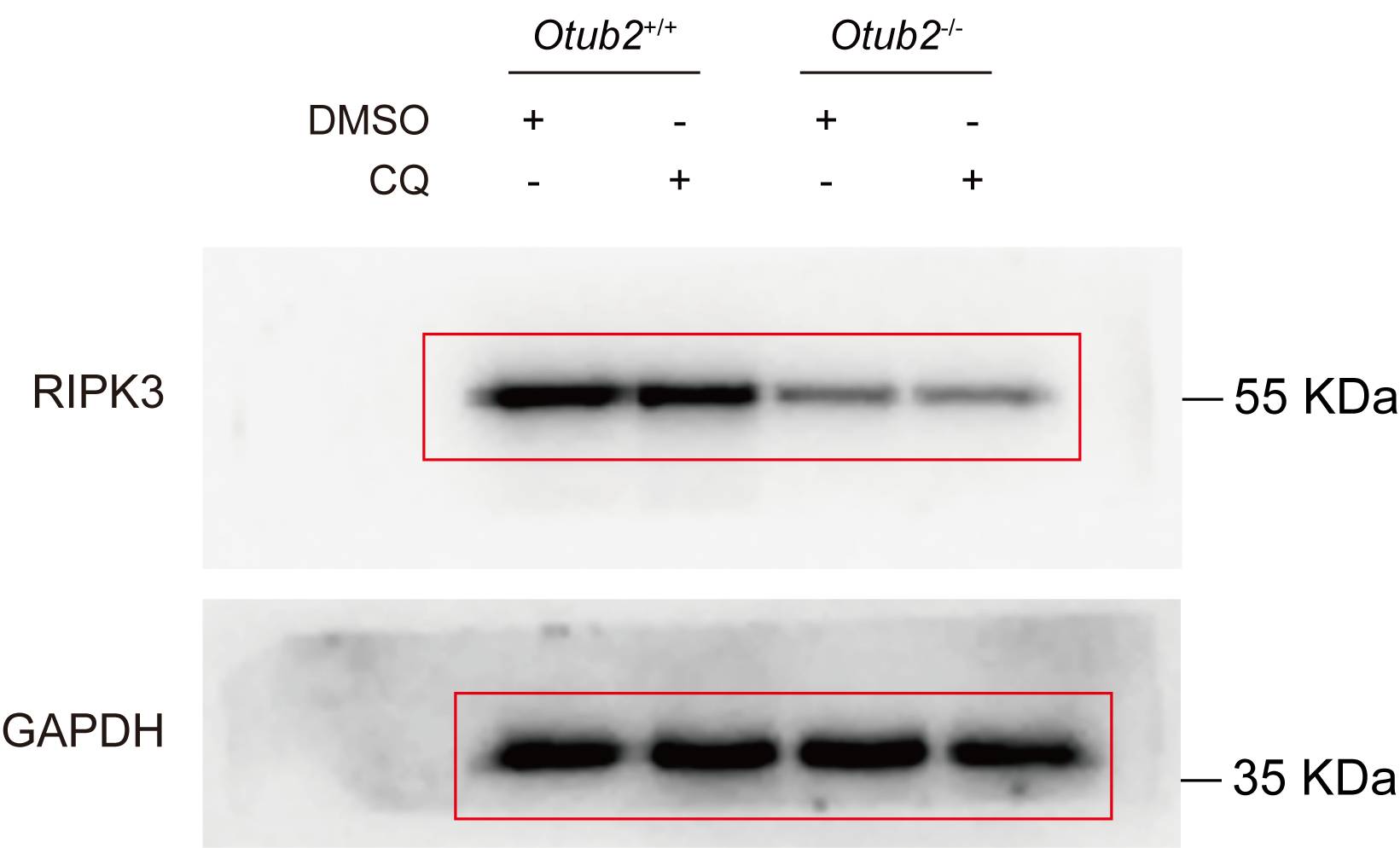

Supplement: Supplementary file 7 — Source data Fig. 5 [file 44321_2025_206_MOESM7_ESM.zip › Source data Fig 5/Fig 5/5B/5B.tif]

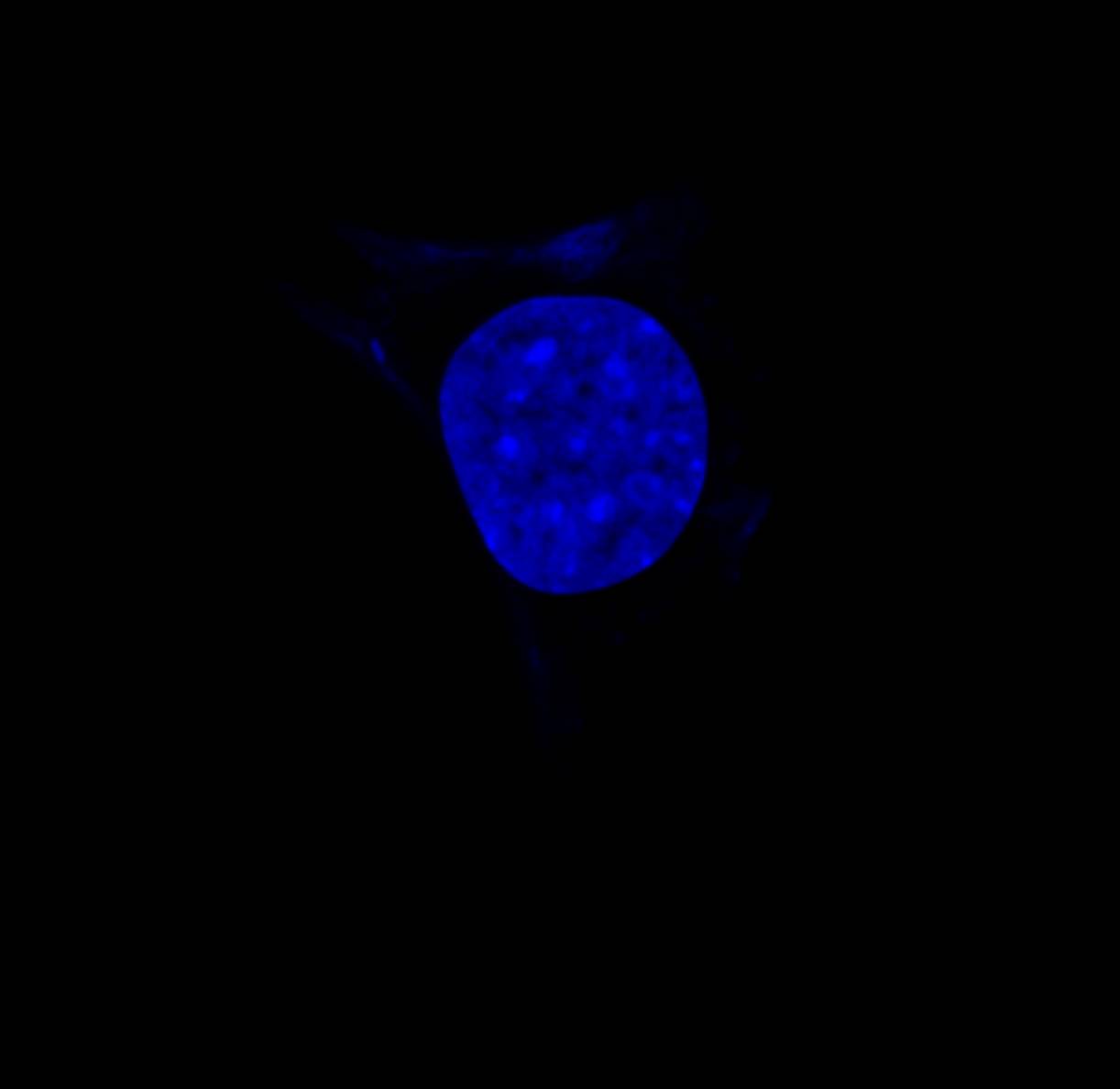

Supplement: Supplementary file 7 — Source data Fig. 5 [file 44321_2025_206_MOESM7_ESM.zip › Source data Fig 5/Fig 5/5C/KO PSMD7 DAPI.tif]

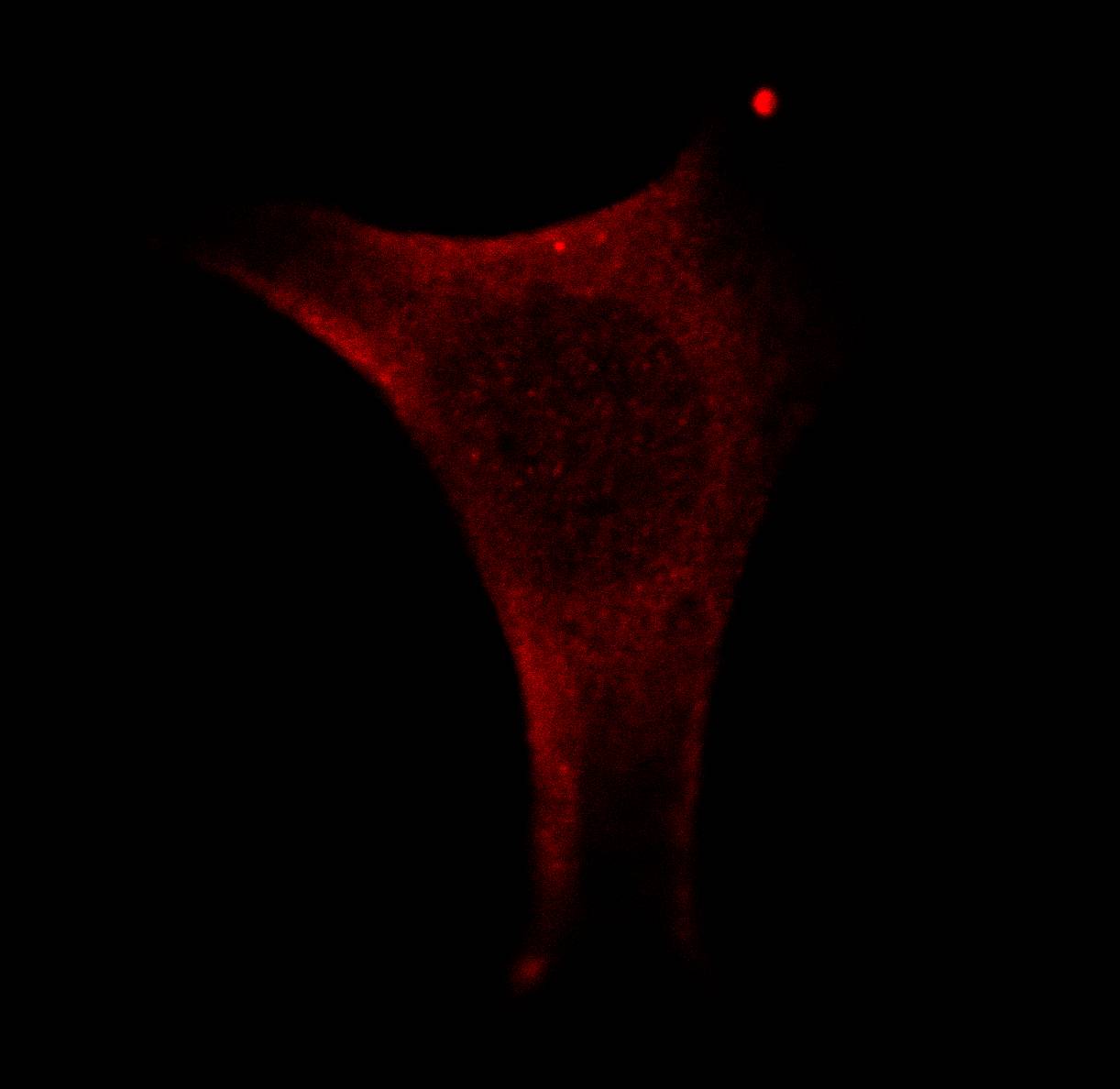

Supplement: Supplementary file 7 — Source data Fig. 5 [file 44321_2025_206_MOESM7_ESM.zip › Source data Fig 5/Fig 5/5C/KO PSMD7 PSMD7.tif]

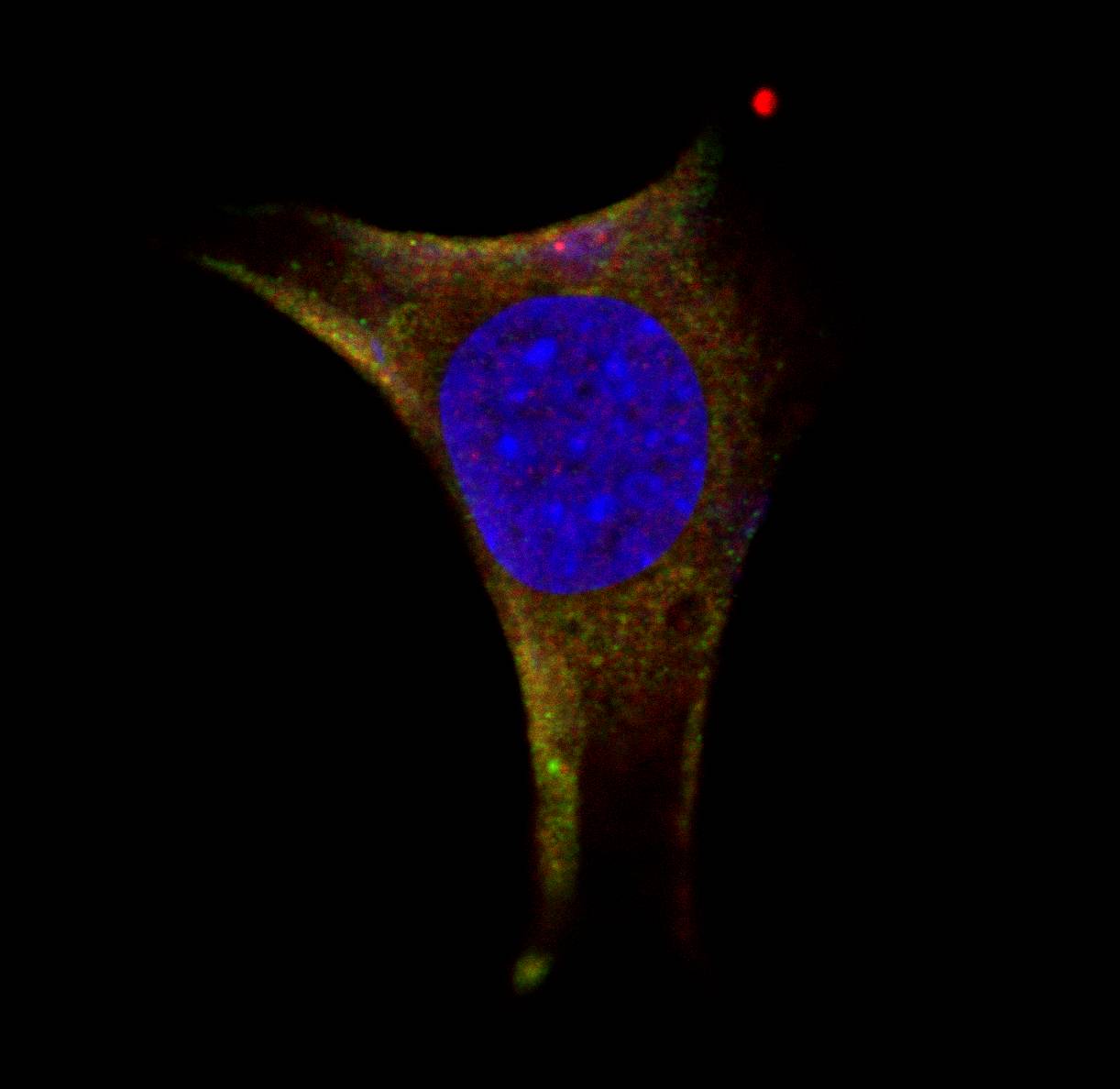

Supplement: Supplementary file 7 — Source data Fig. 5 [file 44321_2025_206_MOESM7_ESM.zip › Source data Fig 5/Fig 5/5C/KO PSMD7 Merge.tif]

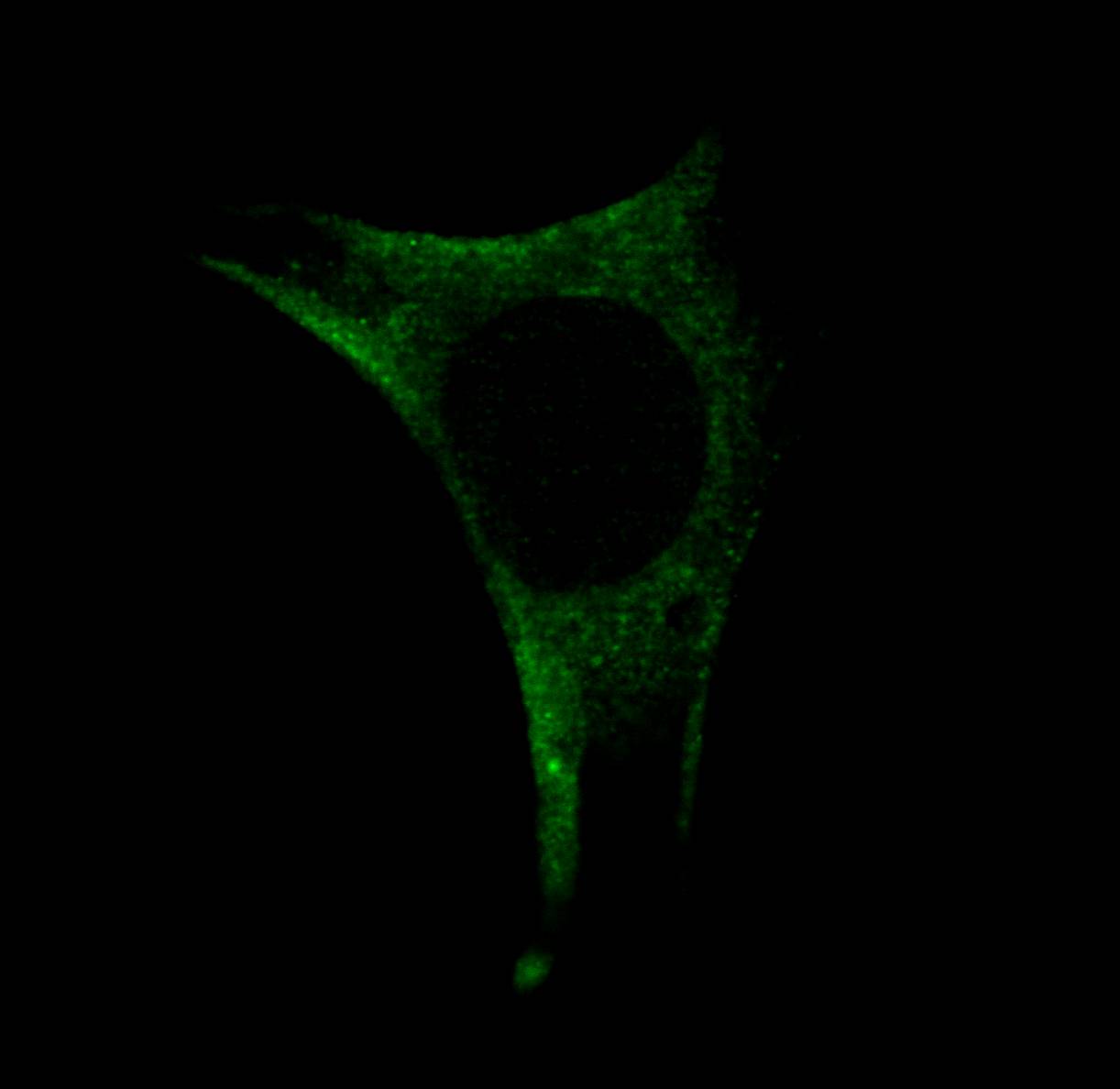

Supplement: Supplementary file 7 — Source data Fig. 5 [file 44321_2025_206_MOESM7_ESM.zip › Source data Fig 5/Fig 5/5C/KO PSMD7 RIPK3.tif]

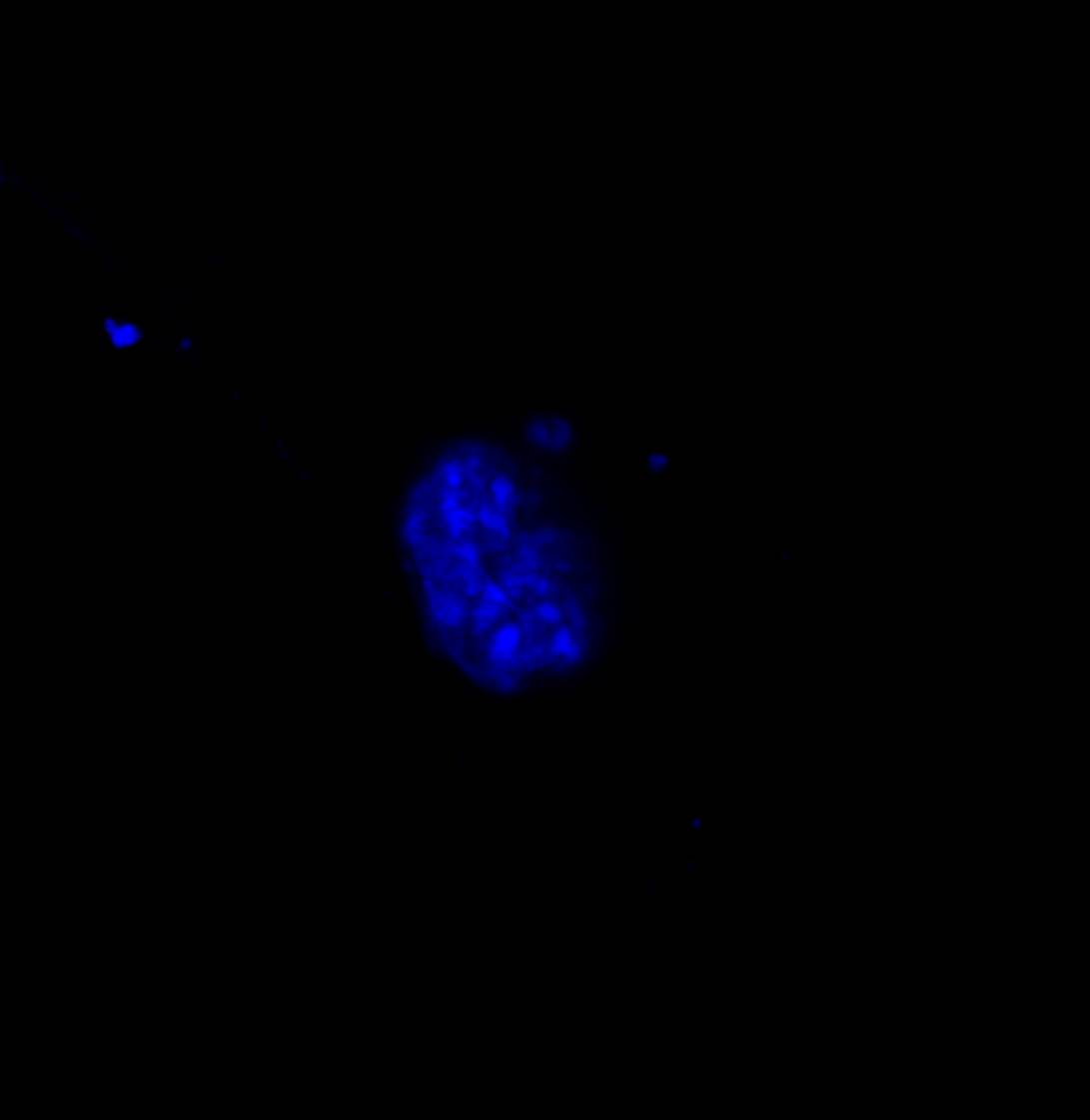

Supplement: Supplementary file 7 — Source data Fig. 5 [file 44321_2025_206_MOESM7_ESM.zip › Source data Fig 5/Fig 5/5C/WT PSMD7 DAPI.tif]

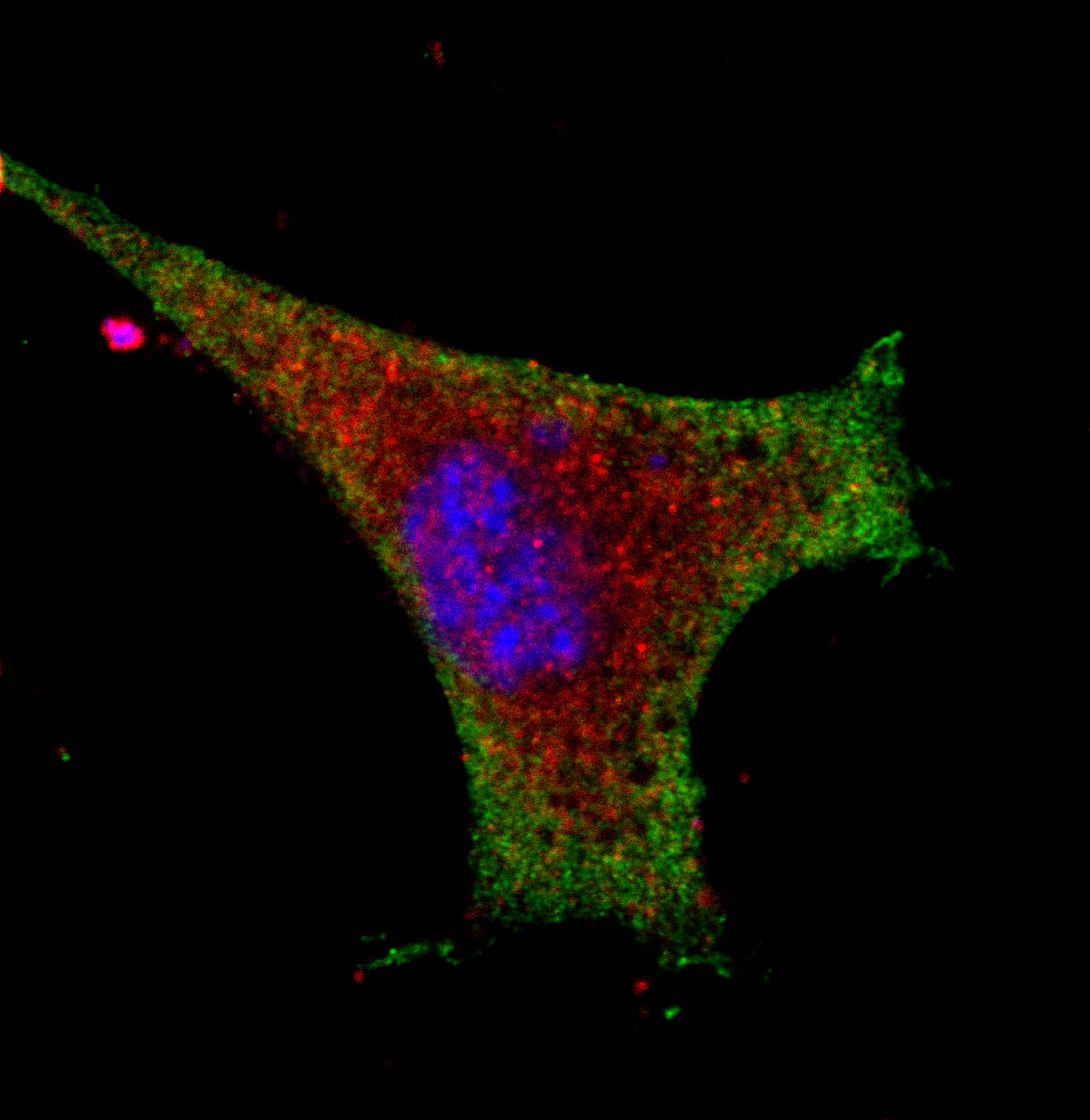

Supplement: Supplementary file 7 — Source data Fig. 5 [file 44321_2025_206_MOESM7_ESM.zip › Source data Fig 5/Fig 5/5C/WT PSMD7 Merge.tif]

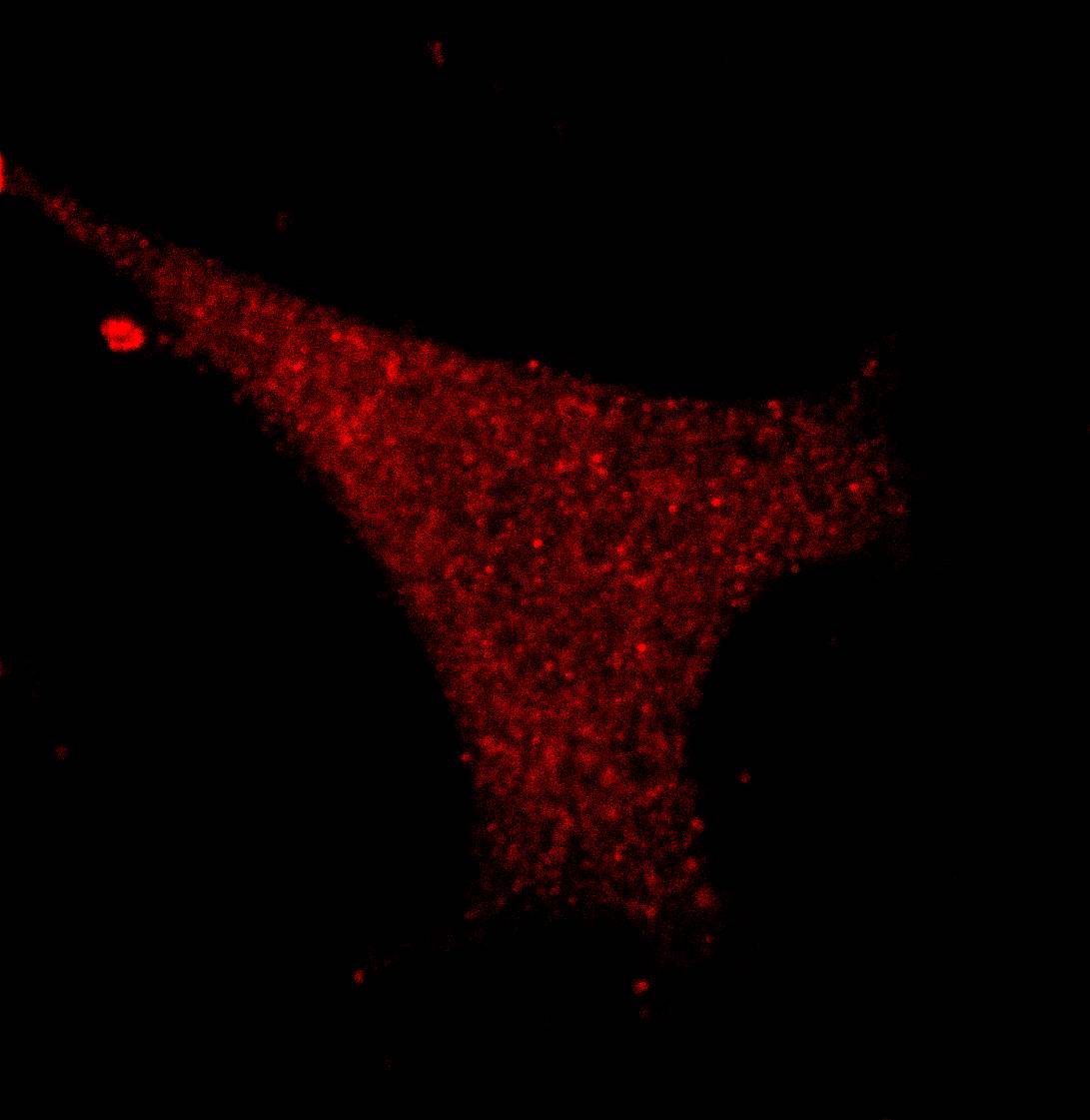

Supplement: Supplementary file 7 — Source data Fig. 5 [file 44321_2025_206_MOESM7_ESM.zip › Source data Fig 5/Fig 5/5C/WT PSMD7 PSMD7.tif]

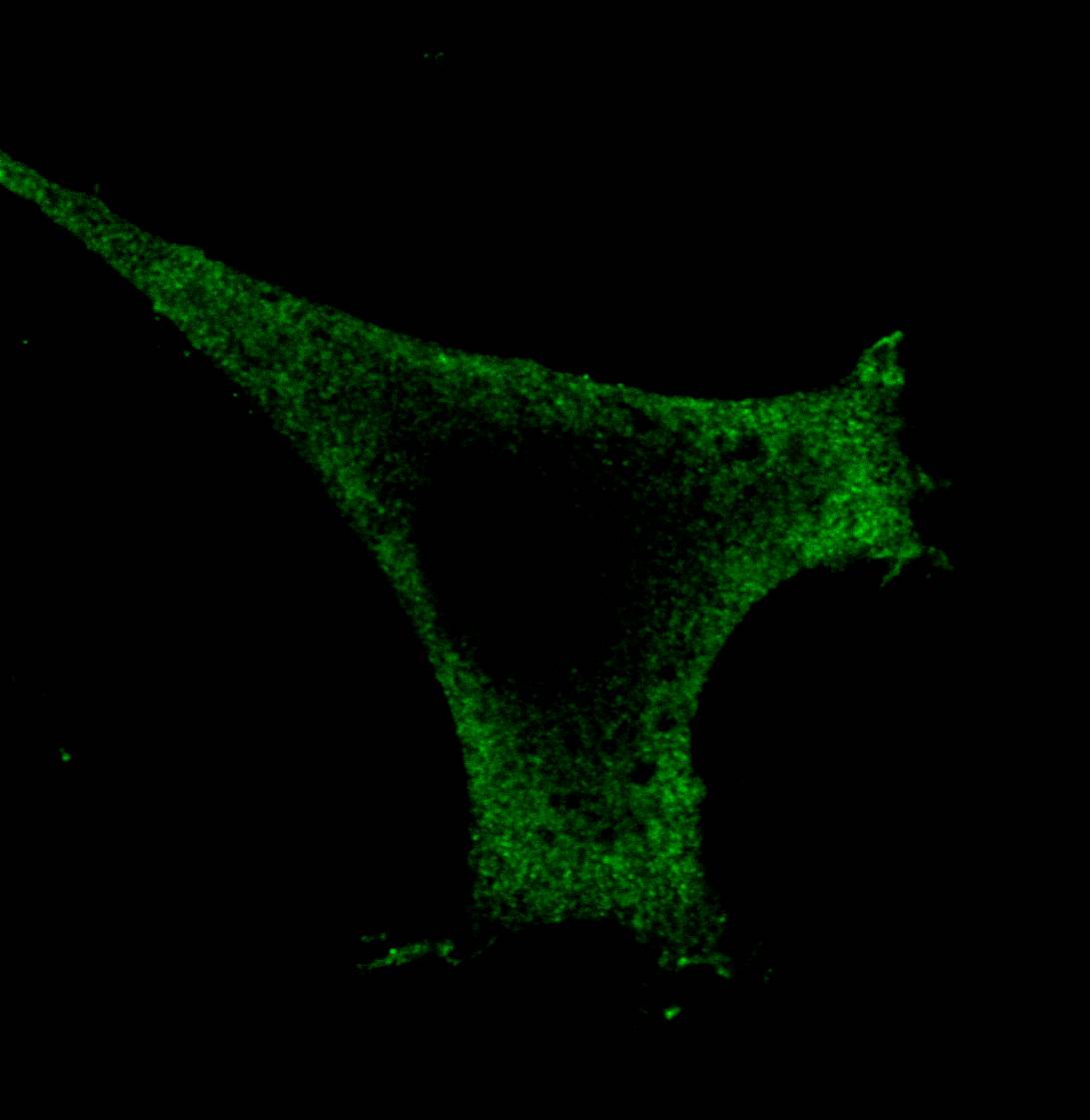

Supplement: Supplementary file 7 — Source data Fig. 5 [file 44321_2025_206_MOESM7_ESM.zip › Source data Fig 5/Fig 5/5C/WT PSMD7 RIPK3.tif]

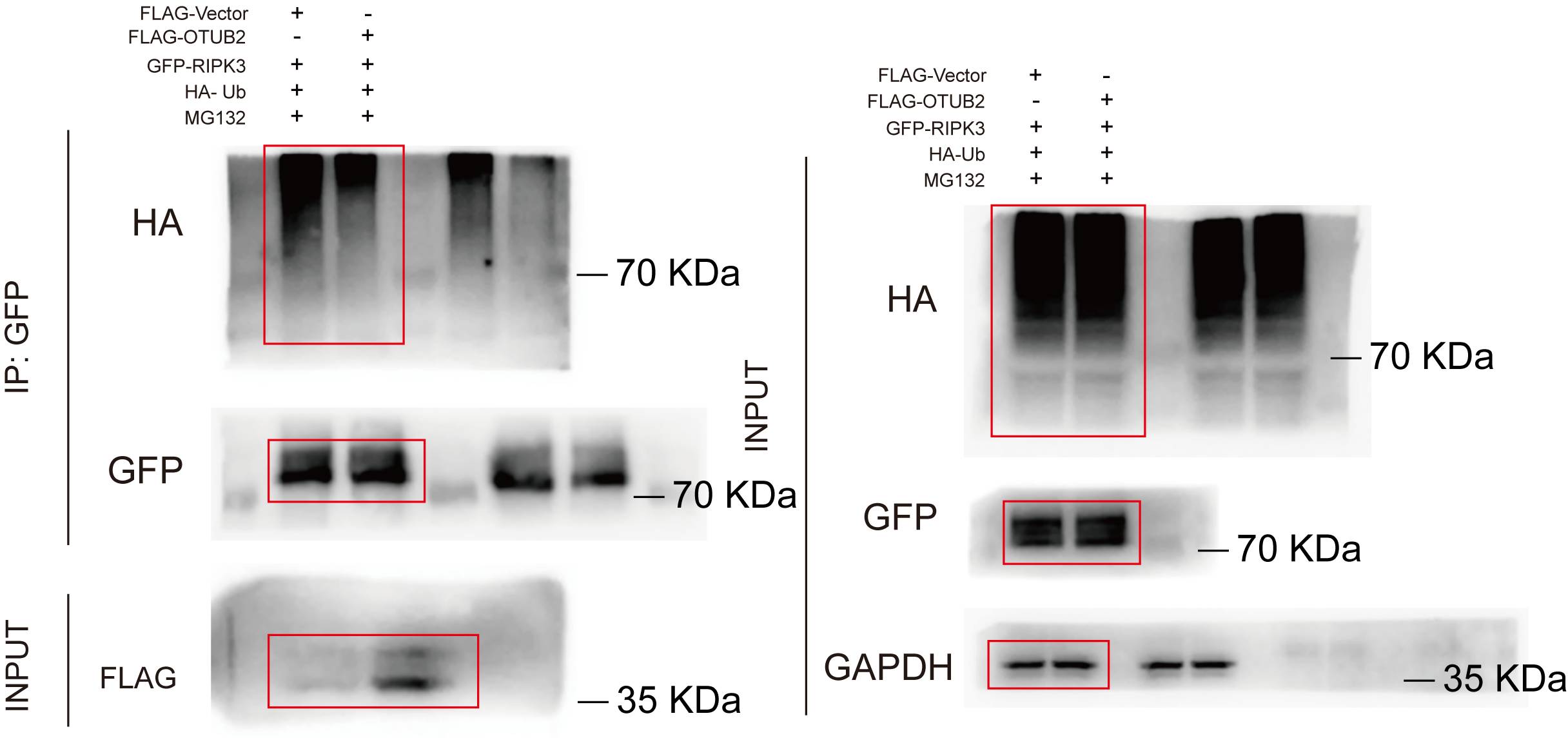

Supplement: Supplementary file 7 — Source data Fig. 5 [file 44321_2025_206_MOESM7_ESM.zip › Source data Fig 5/Fig 5/5D/5D.tif]

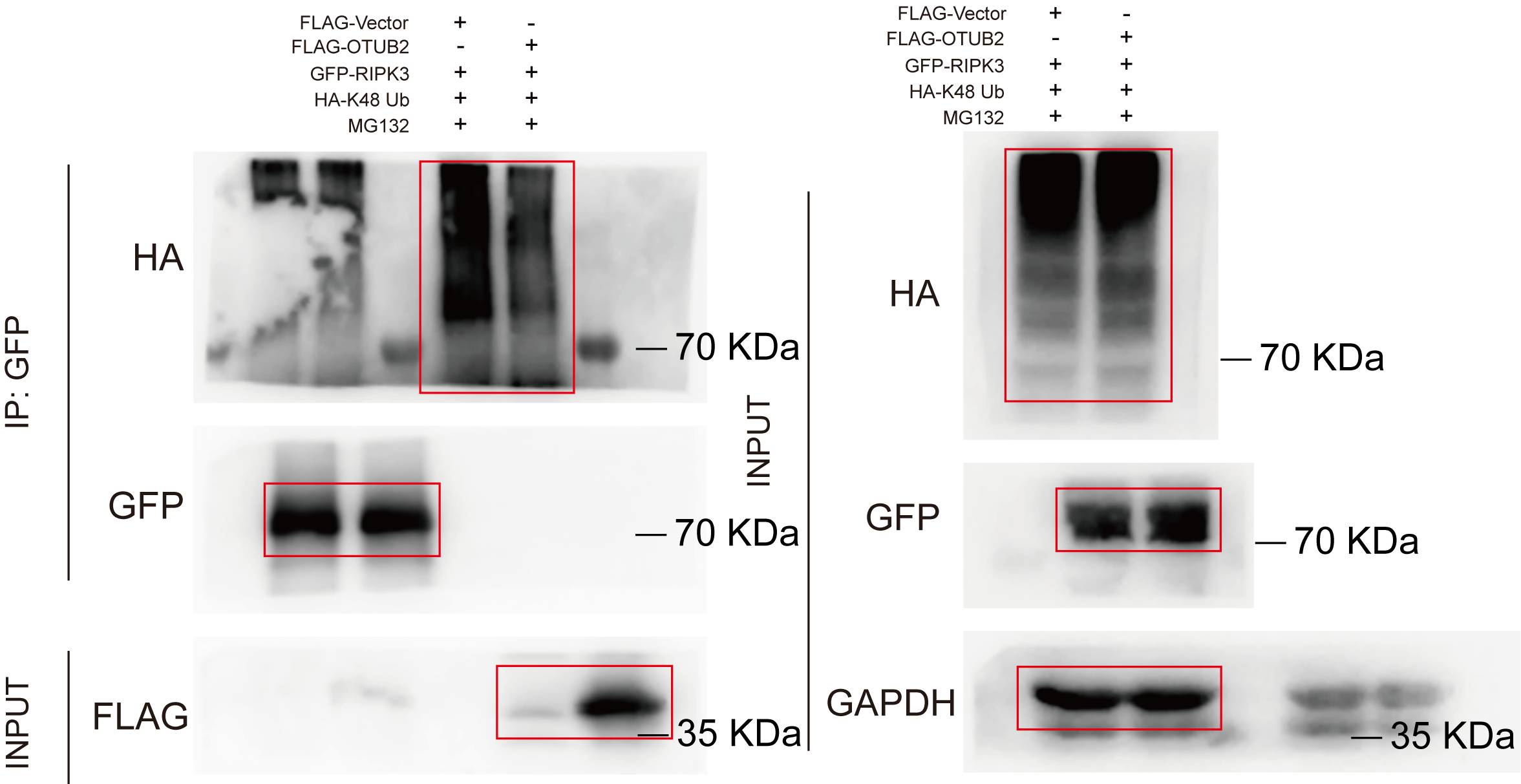

Supplement: Supplementary file 7 — Source data Fig. 5 [file 44321_2025_206_MOESM7_ESM.zip › Source data Fig 5/Fig 5/5E/5E.tif]

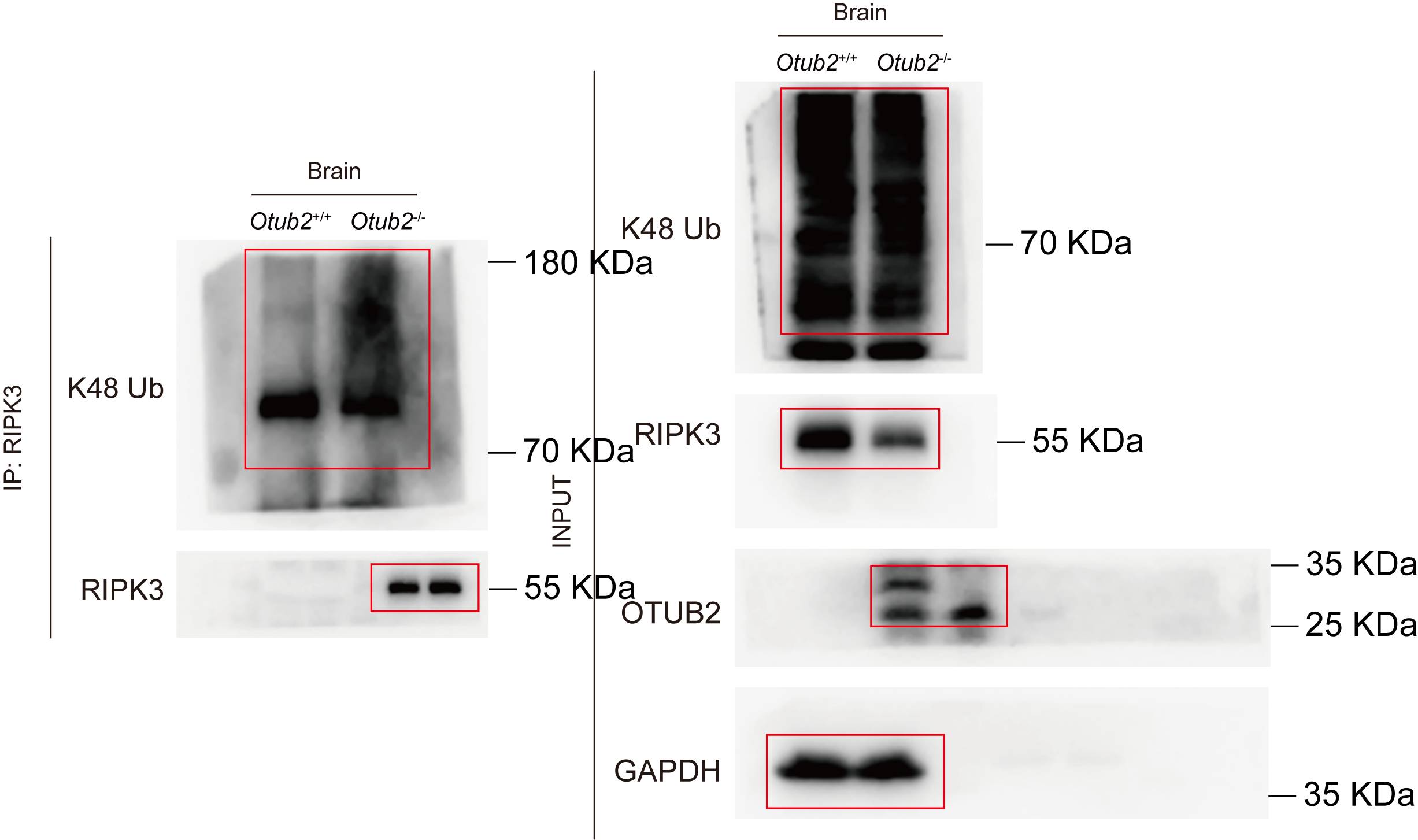

Supplement: Supplementary file 7 — Source data Fig. 5 [file 44321_2025_206_MOESM7_ESM.zip › Source data Fig 5/Fig 5/5F/5F.tif]

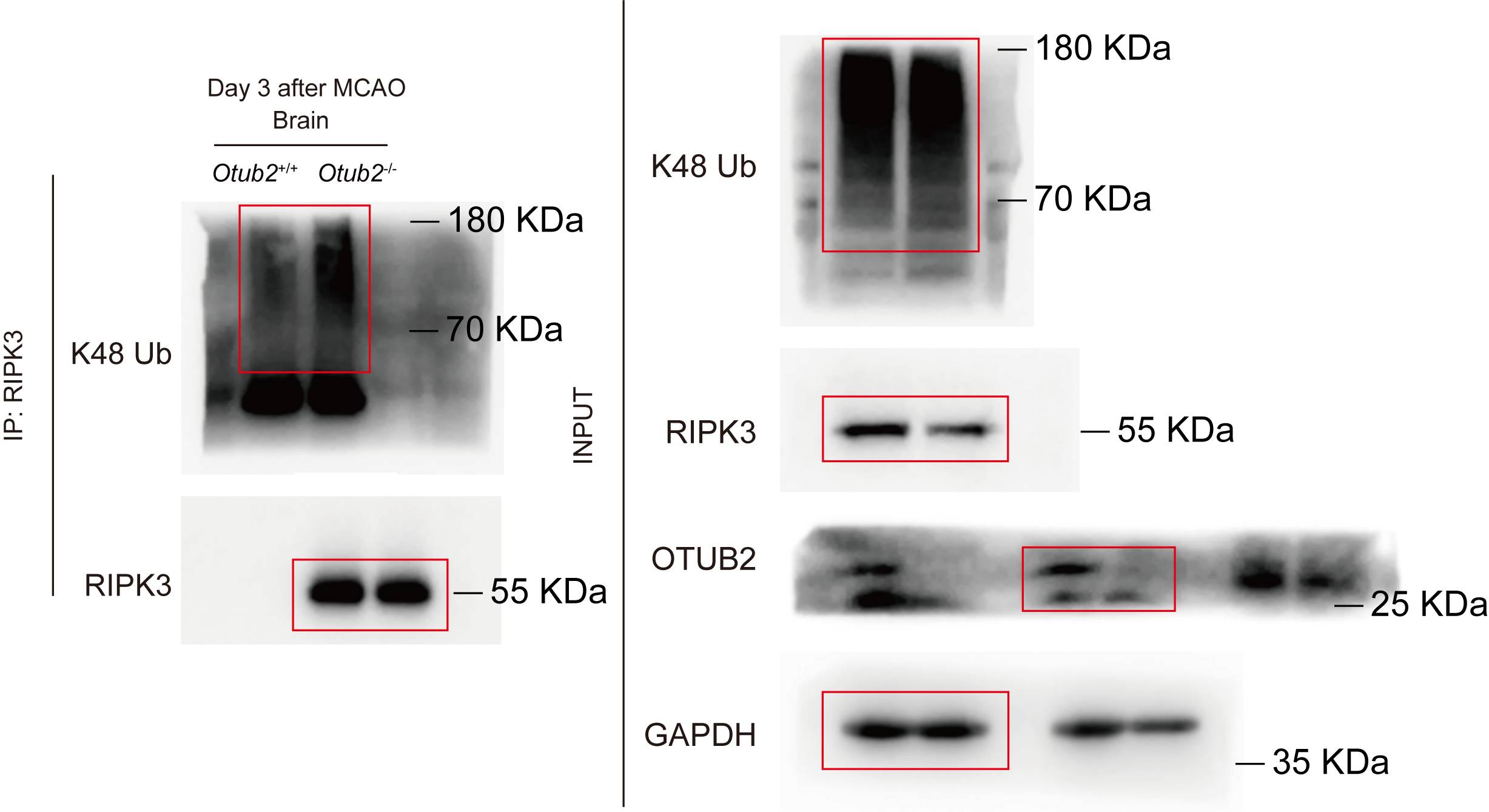

Supplement: Supplementary file 7 — Source data Fig. 5 [file 44321_2025_206_MOESM7_ESM.zip › Source data Fig 5/Fig 5/5G/SG.tif]
