# Supplementary figures and images for: Deubiquitination of RIPK3 by OTUB2 potentiates neuronal necroptosis after ischemic stroke (part 2 of 3)
Source: EMBO Mol Med. 2025 Feb 28;17(4):679–95. doi: 10.1038/s44321-025-00206-6 (PMC11982199; doi:10.1038/s44321-025-00206-6)

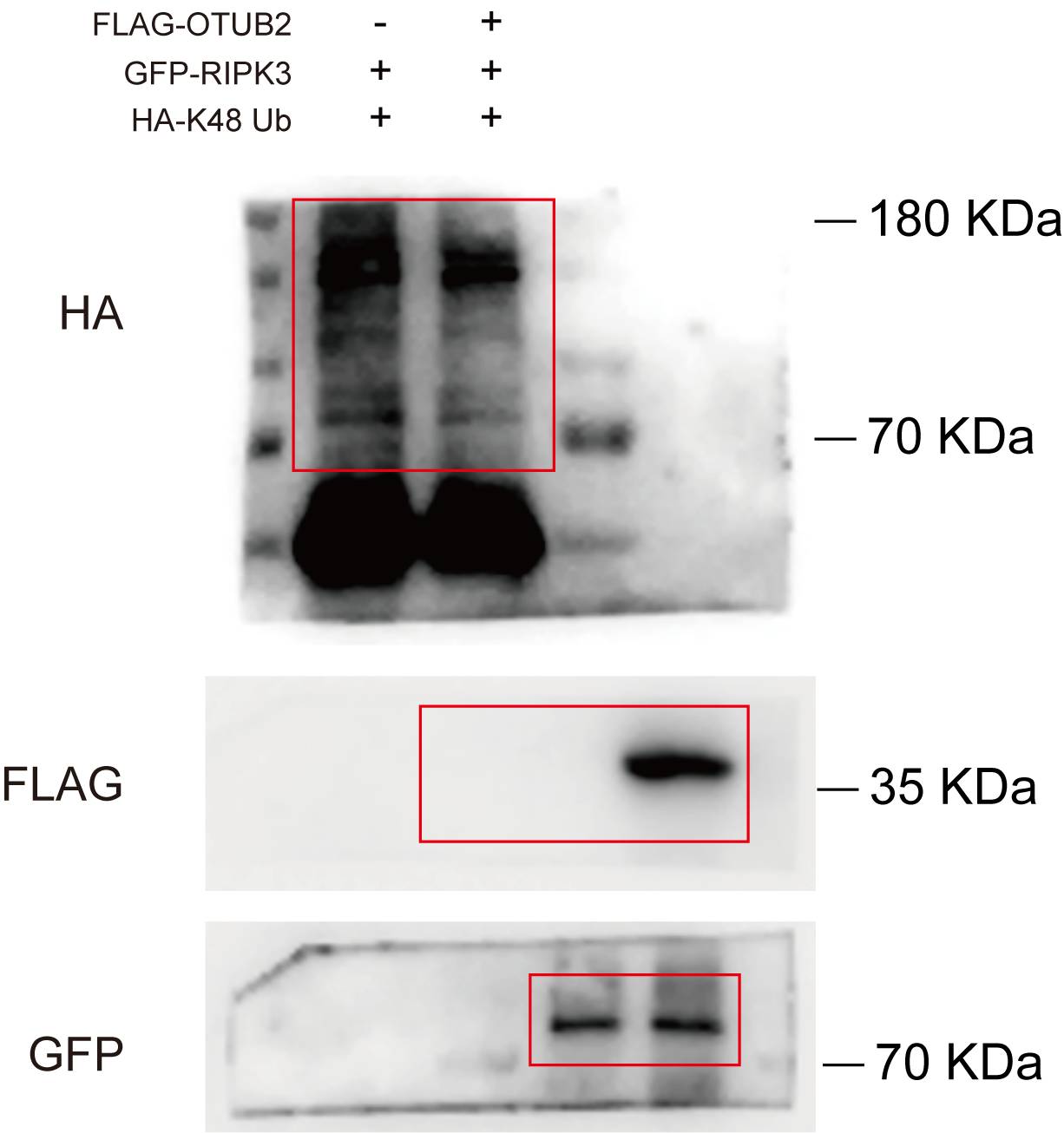

Supplement: Supplementary file 7 — Source data Fig. 5 [file 44321_2025_206_MOESM7_ESM.zip › Source data Fig 5/Fig 5/5H/5H.tif]

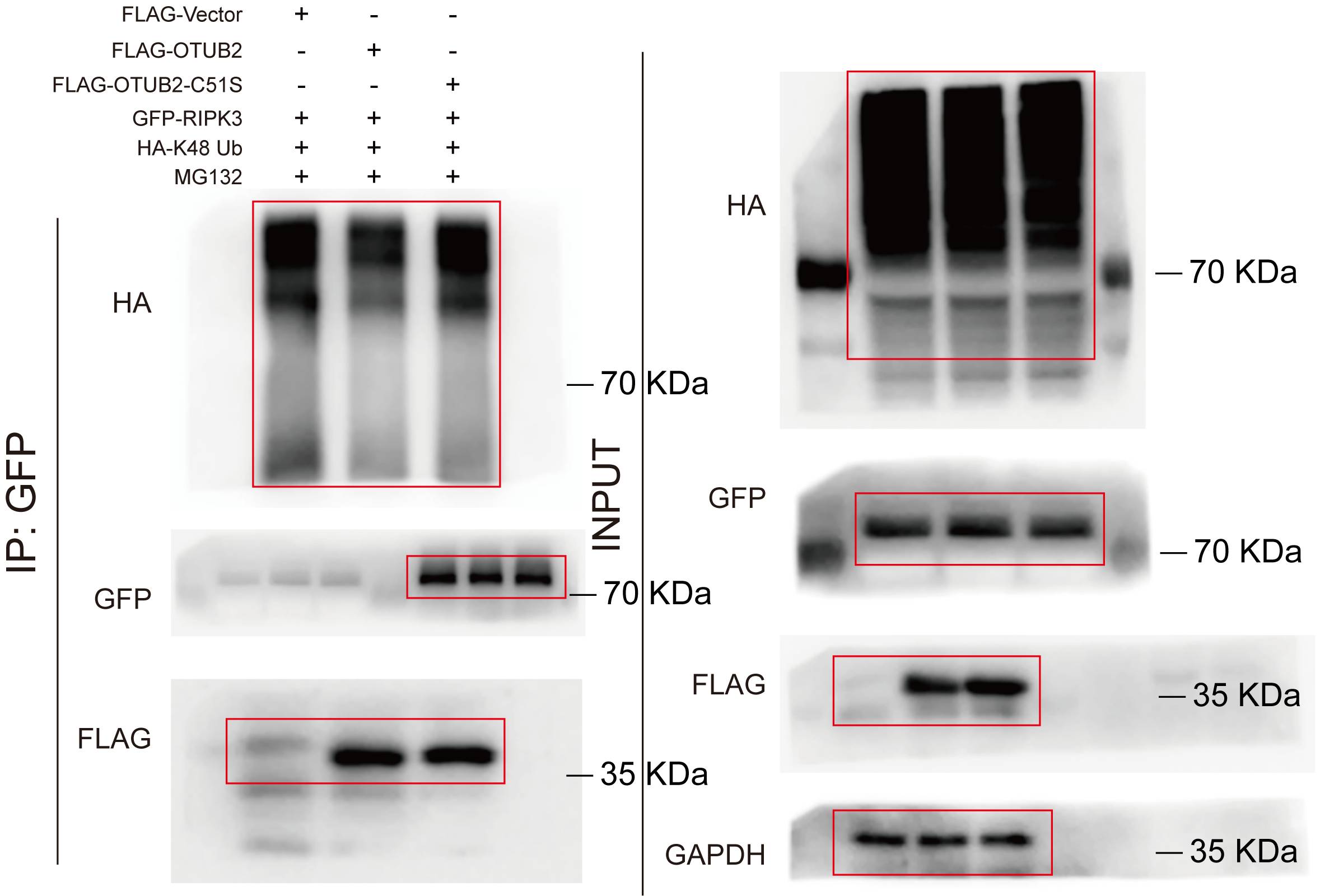

Supplement: Supplementary file 7 — Source data Fig. 5 [file 44321_2025_206_MOESM7_ESM.zip › Source data Fig 5/Fig 5/5J/5J.tif]

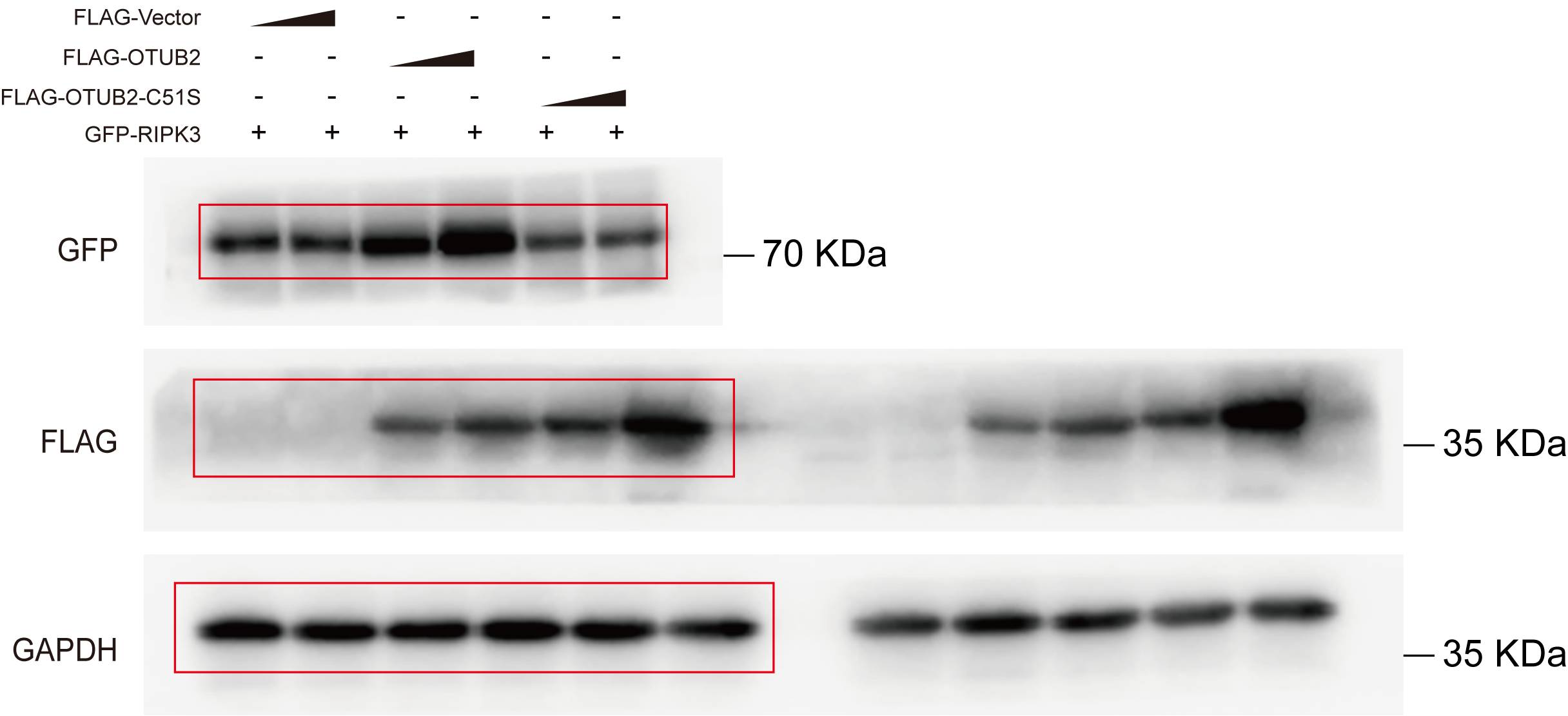

Supplement: Supplementary file 7 — Source data Fig. 5 [file 44321_2025_206_MOESM7_ESM.zip › Source data Fig 5/Fig 5/5K/5K.tif]

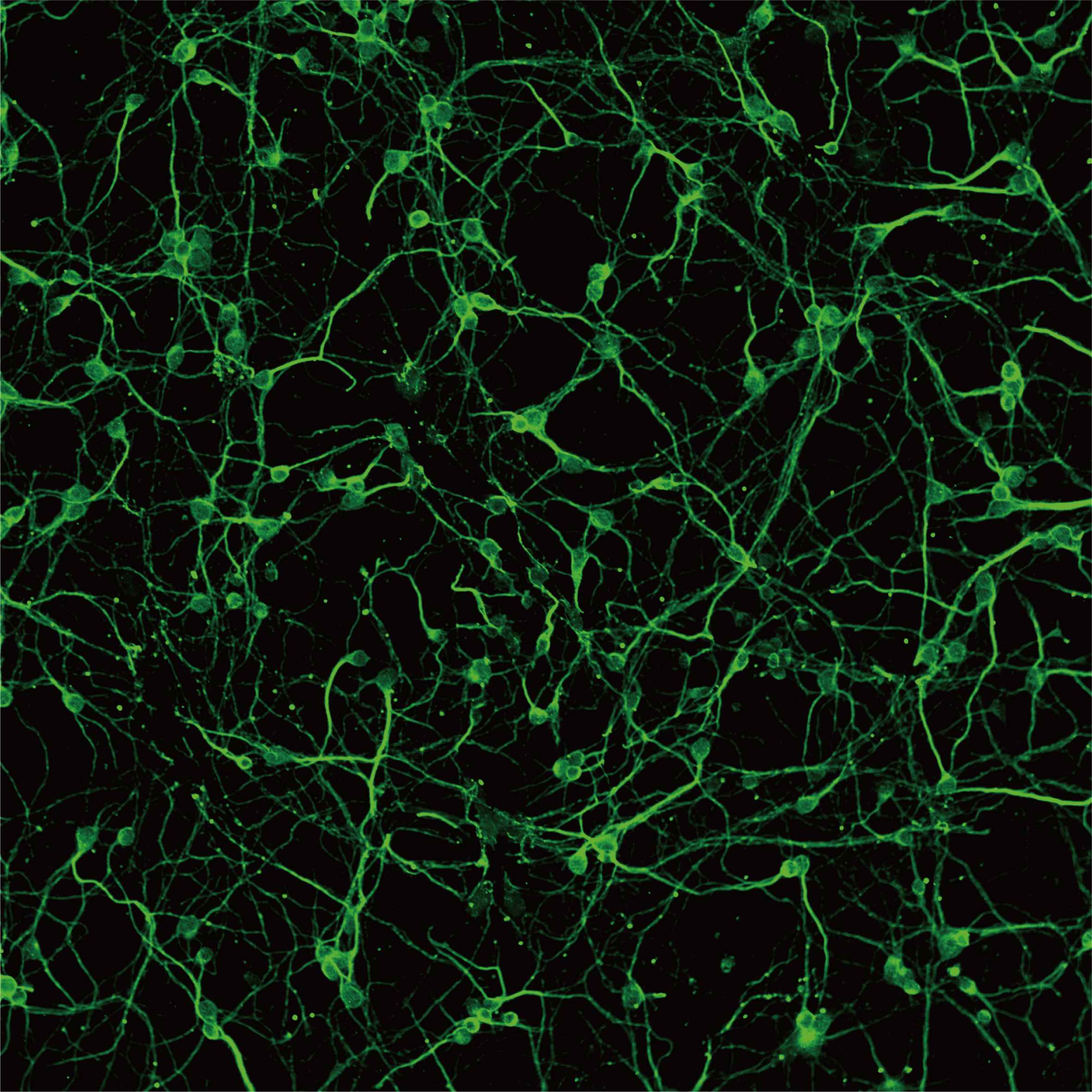

Supplement: Supplementary file 8 — Source data Fig. 6 [file 44321_2025_206_MOESM8_ESM.zip › Source data Fig 6/Fig 6/6B/KO-Normoxia- MAP2.tif]

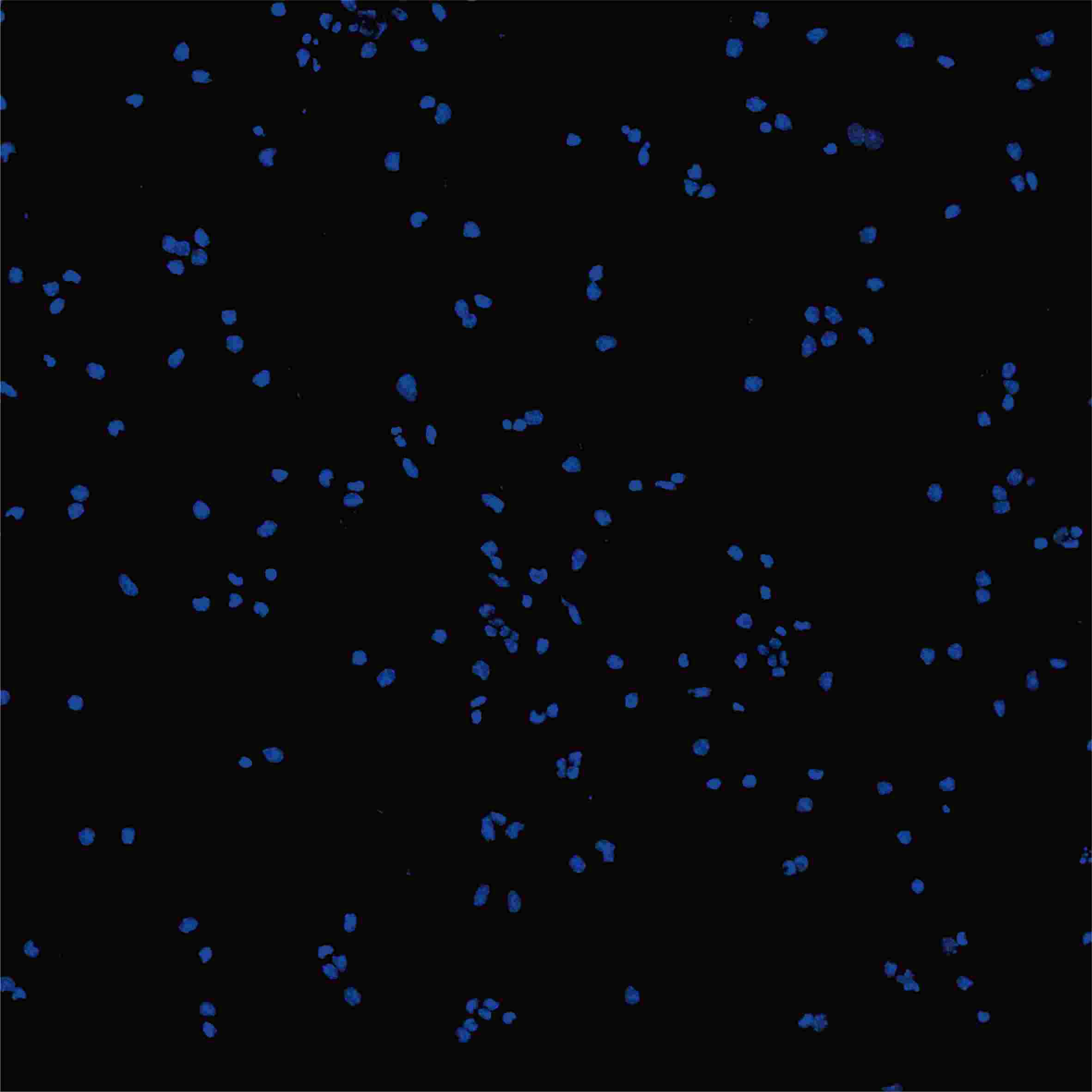

Supplement: Supplementary file 8 — Source data Fig. 6 [file 44321_2025_206_MOESM8_ESM.zip › Source data Fig 6/Fig 6/6B/KO-Normoxia-DAPI.tif]

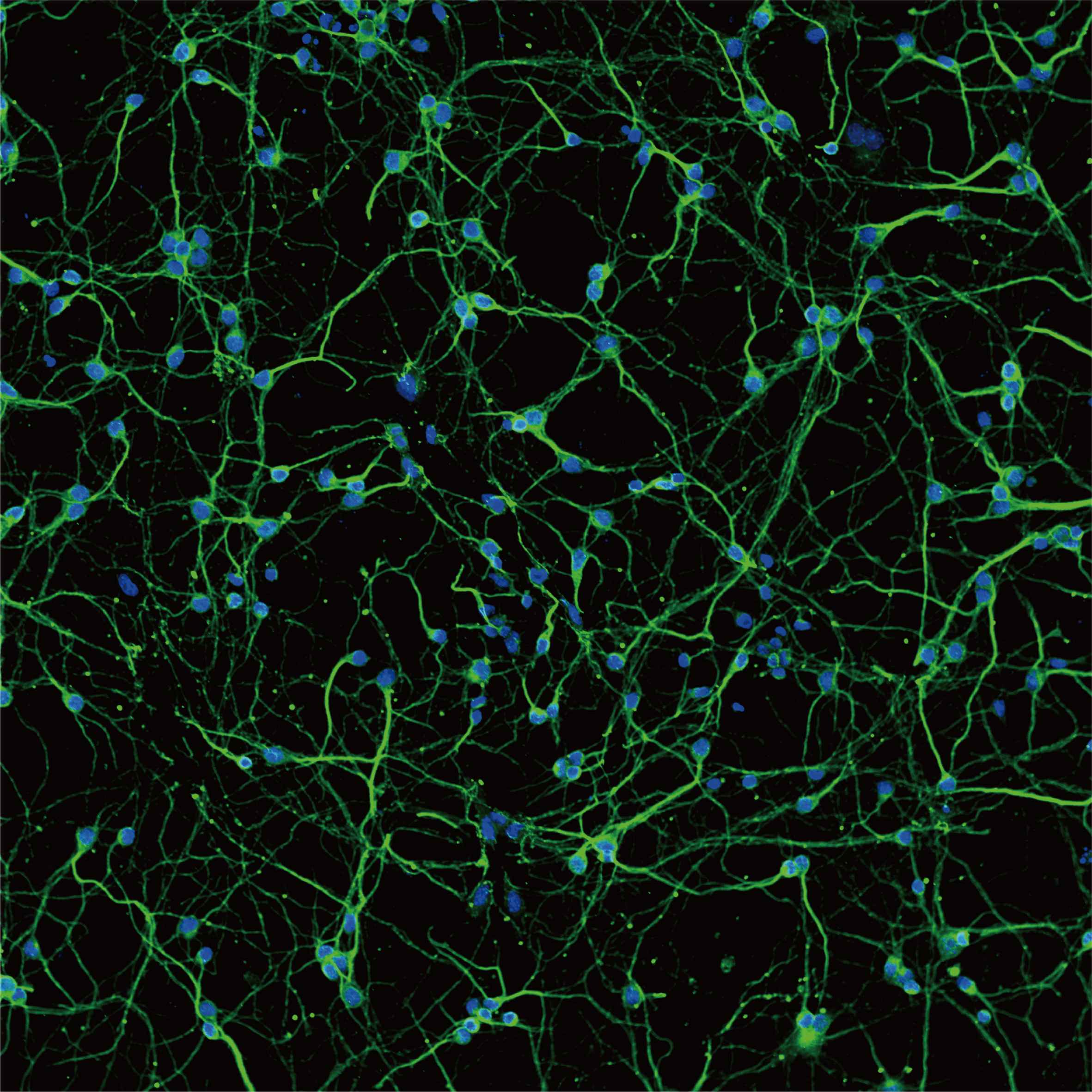

Supplement: Supplementary file 8 — Source data Fig. 6 [file 44321_2025_206_MOESM8_ESM.zip › Source data Fig 6/Fig 6/6B/KO-Normoxia-MERGE.tif]

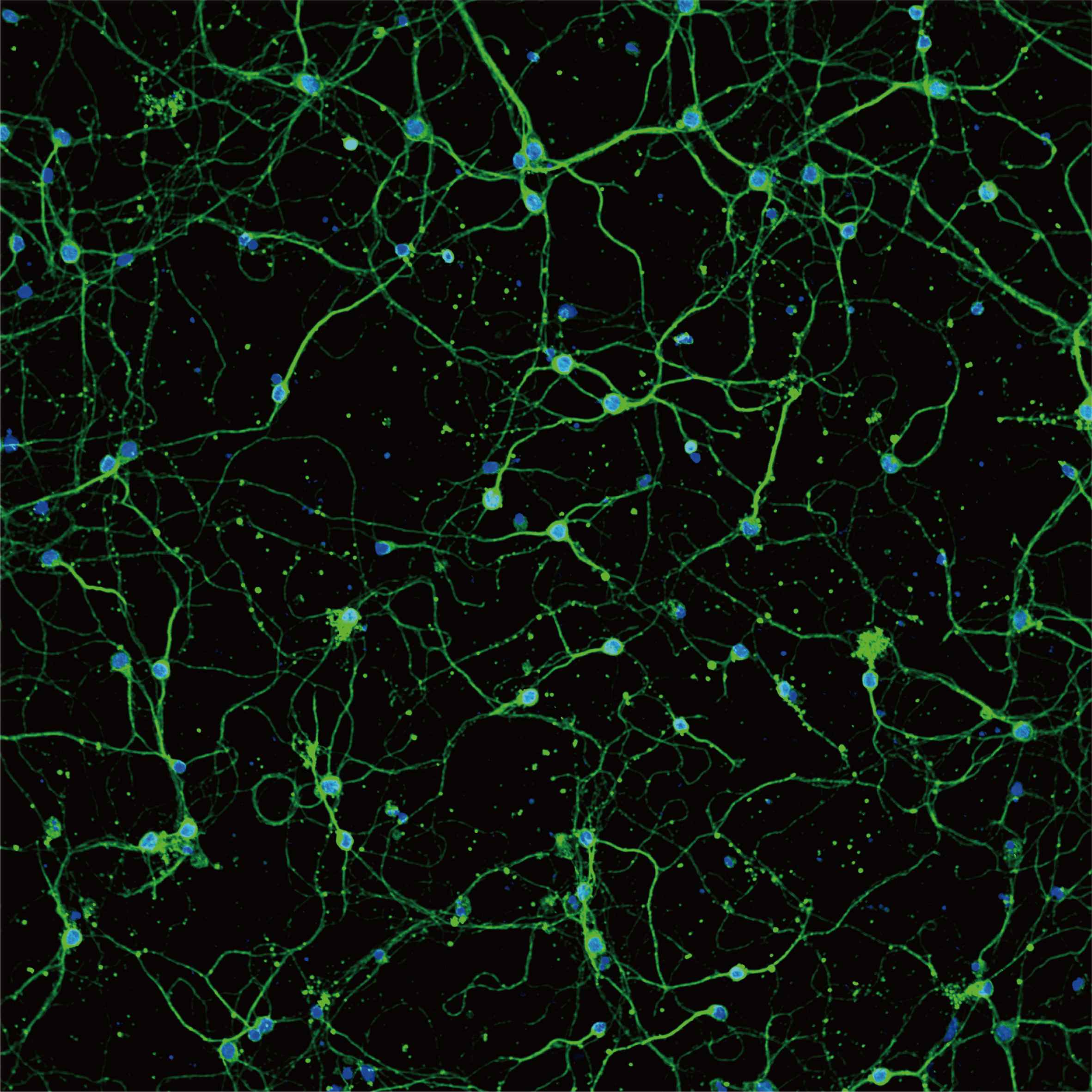

Supplement: Supplementary file 8 — Source data Fig. 6 [file 44321_2025_206_MOESM8_ESM.zip › Source data Fig 6/Fig 6/6B/KO-OGD MERGE.tif]

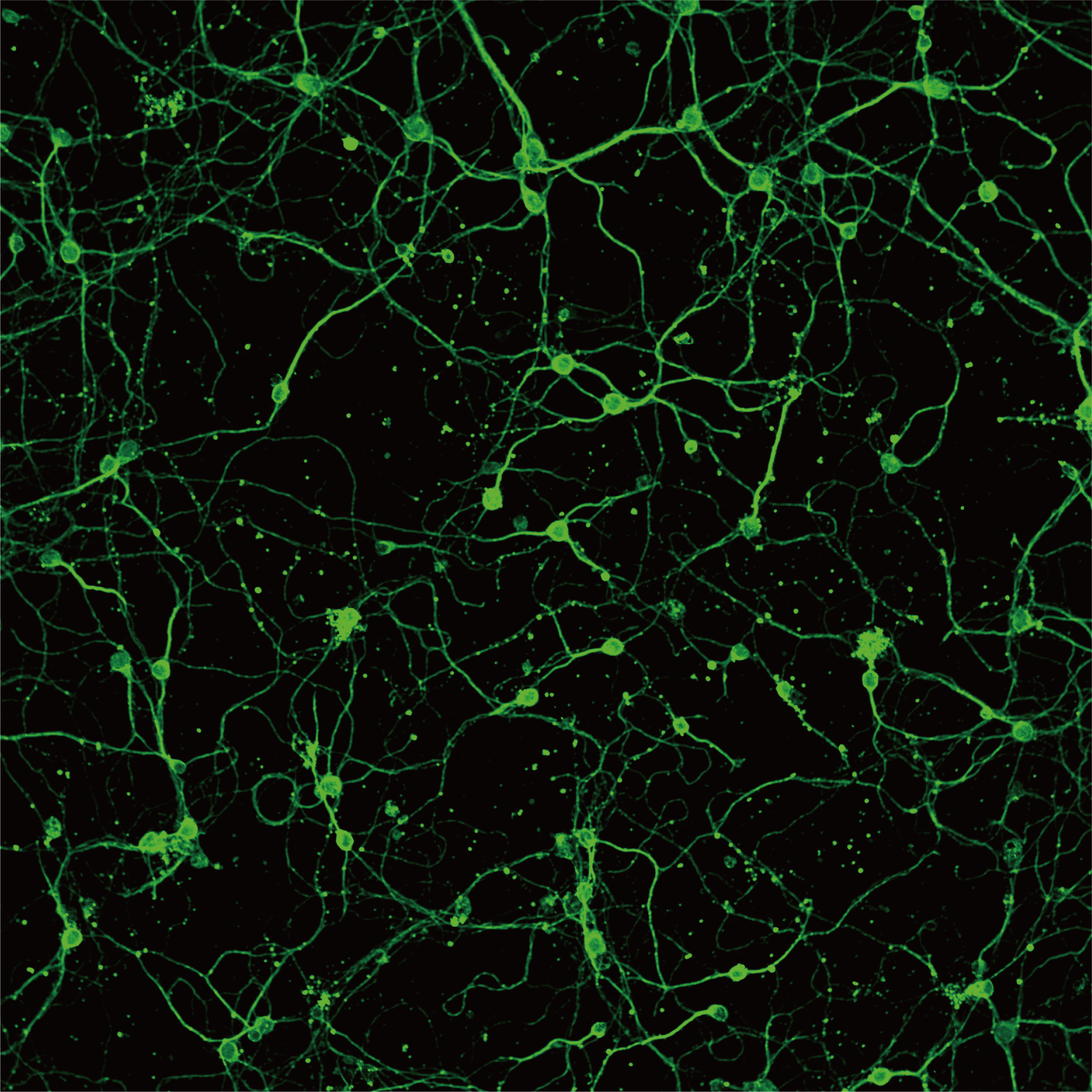

Supplement: Supplementary file 8 — Source data Fig. 6 [file 44321_2025_206_MOESM8_ESM.zip › Source data Fig 6/Fig 6/6B/KO-OGD MAP2.tif]

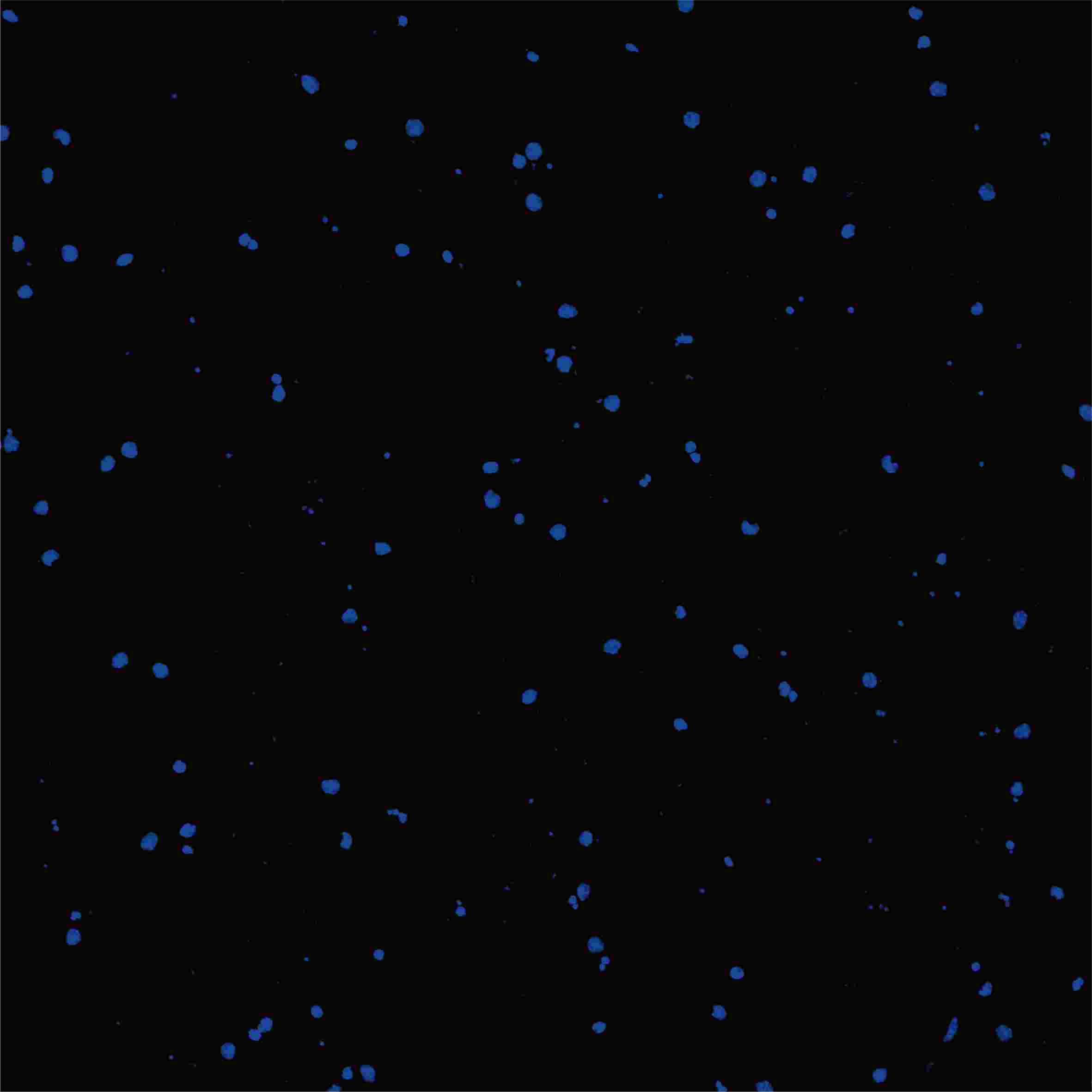

Supplement: Supplementary file 8 — Source data Fig. 6 [file 44321_2025_206_MOESM8_ESM.zip › Source data Fig 6/Fig 6/6B/KO-OGD- DAPI.tif]

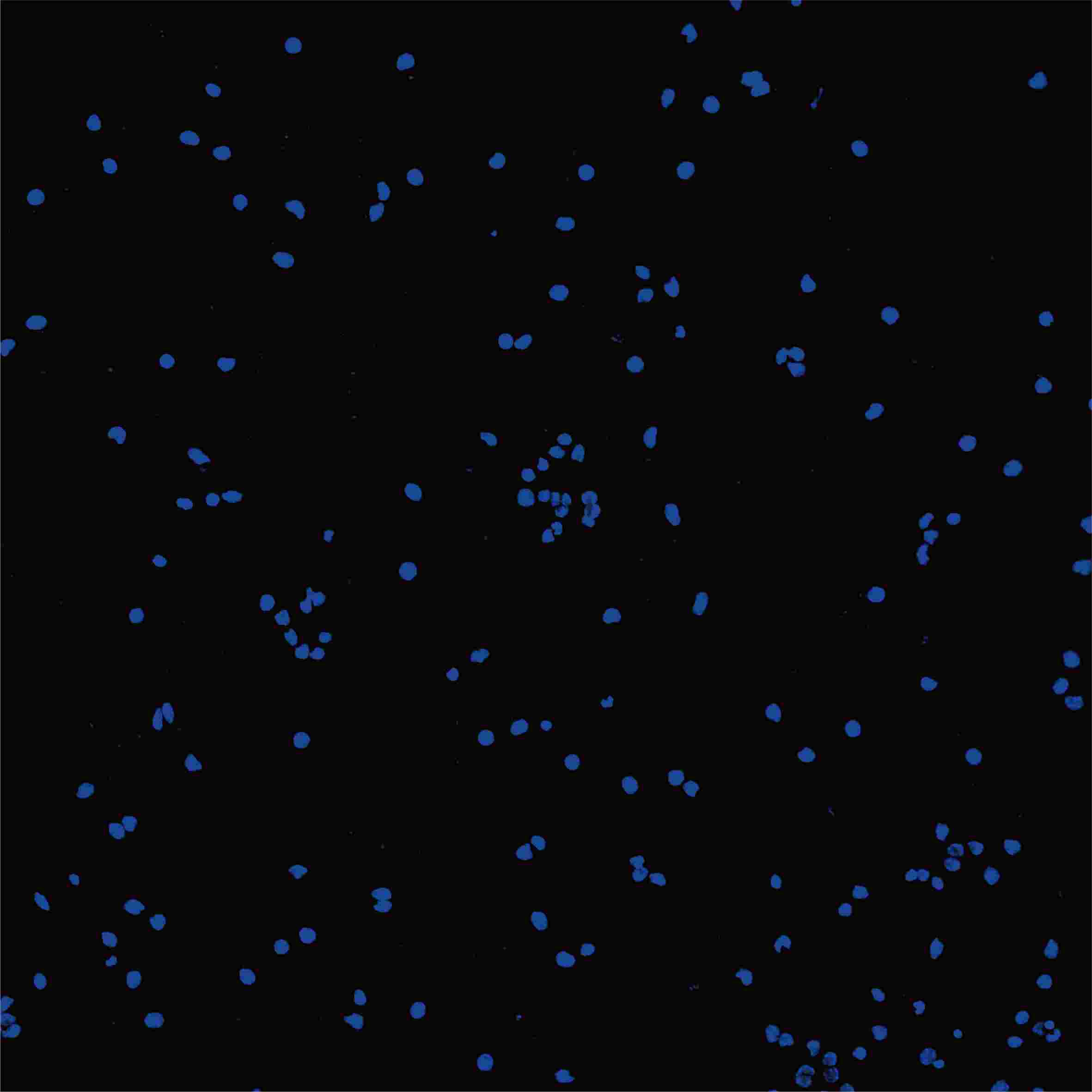

Supplement: Supplementary file 8 — Source data Fig. 6 [file 44321_2025_206_MOESM8_ESM.zip › Source data Fig 6/Fig 6/6B/WT-Normoxia-DAPI.tif]

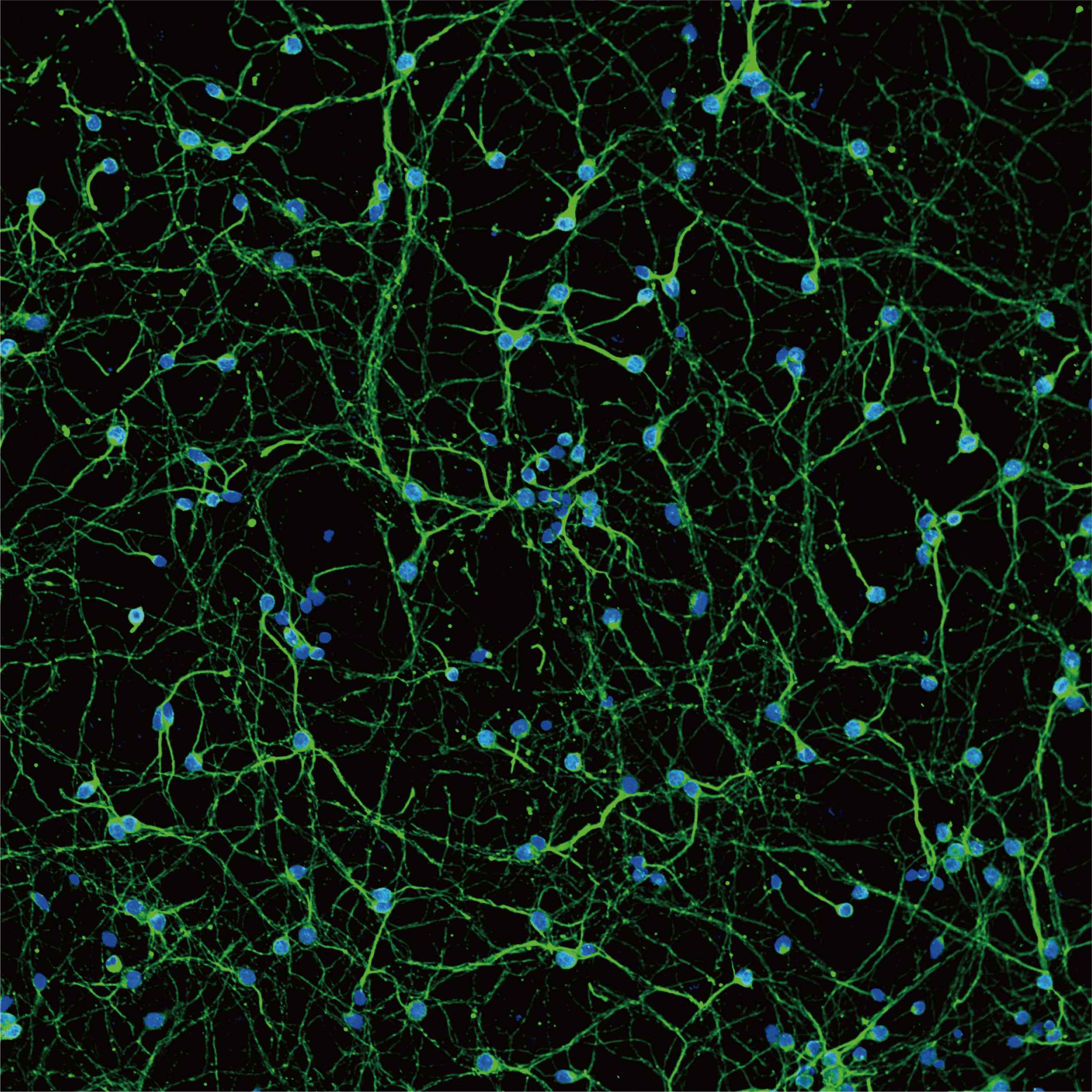

Supplement: Supplementary file 8 — Source data Fig. 6 [file 44321_2025_206_MOESM8_ESM.zip › Source data Fig 6/Fig 6/6B/WT-Normoxia-MERGE.tif]

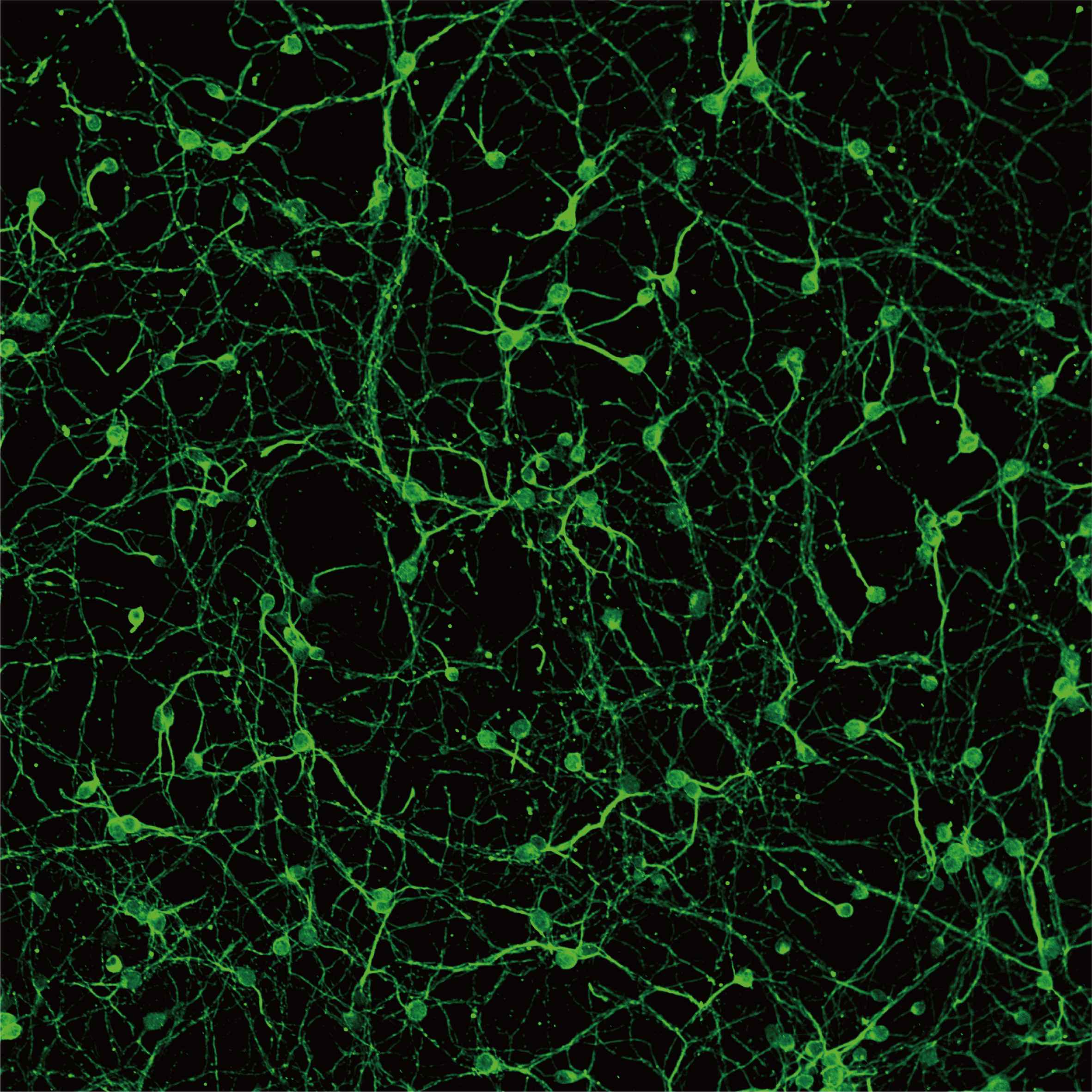

Supplement: Supplementary file 8 — Source data Fig. 6 [file 44321_2025_206_MOESM8_ESM.zip › Source data Fig 6/Fig 6/6B/WT-Normoxia-NEUN.tif]

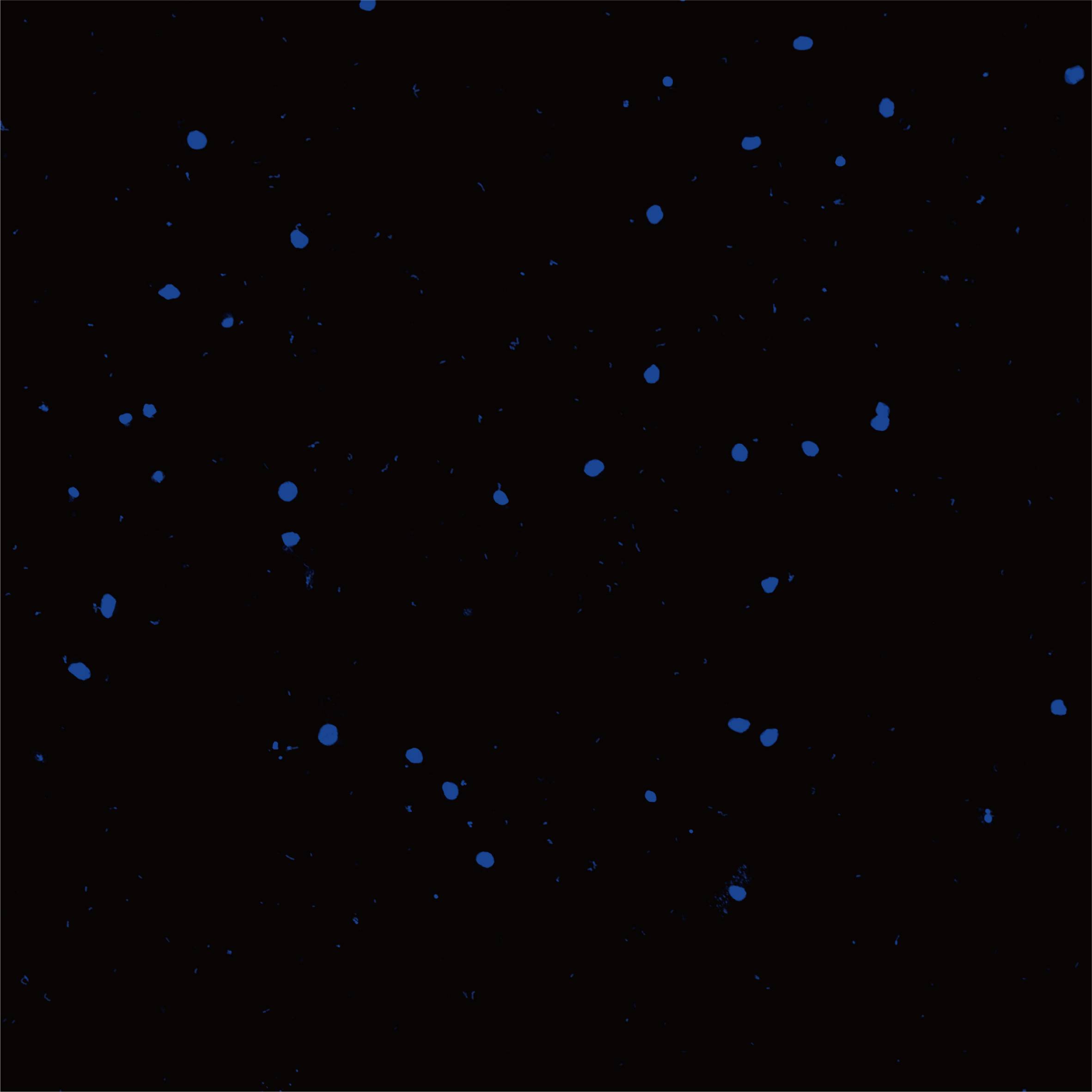

Supplement: Supplementary file 8 — Source data Fig. 6 [file 44321_2025_206_MOESM8_ESM.zip › Source data Fig 6/Fig 6/6B/WT-OGD DAPI.tif]

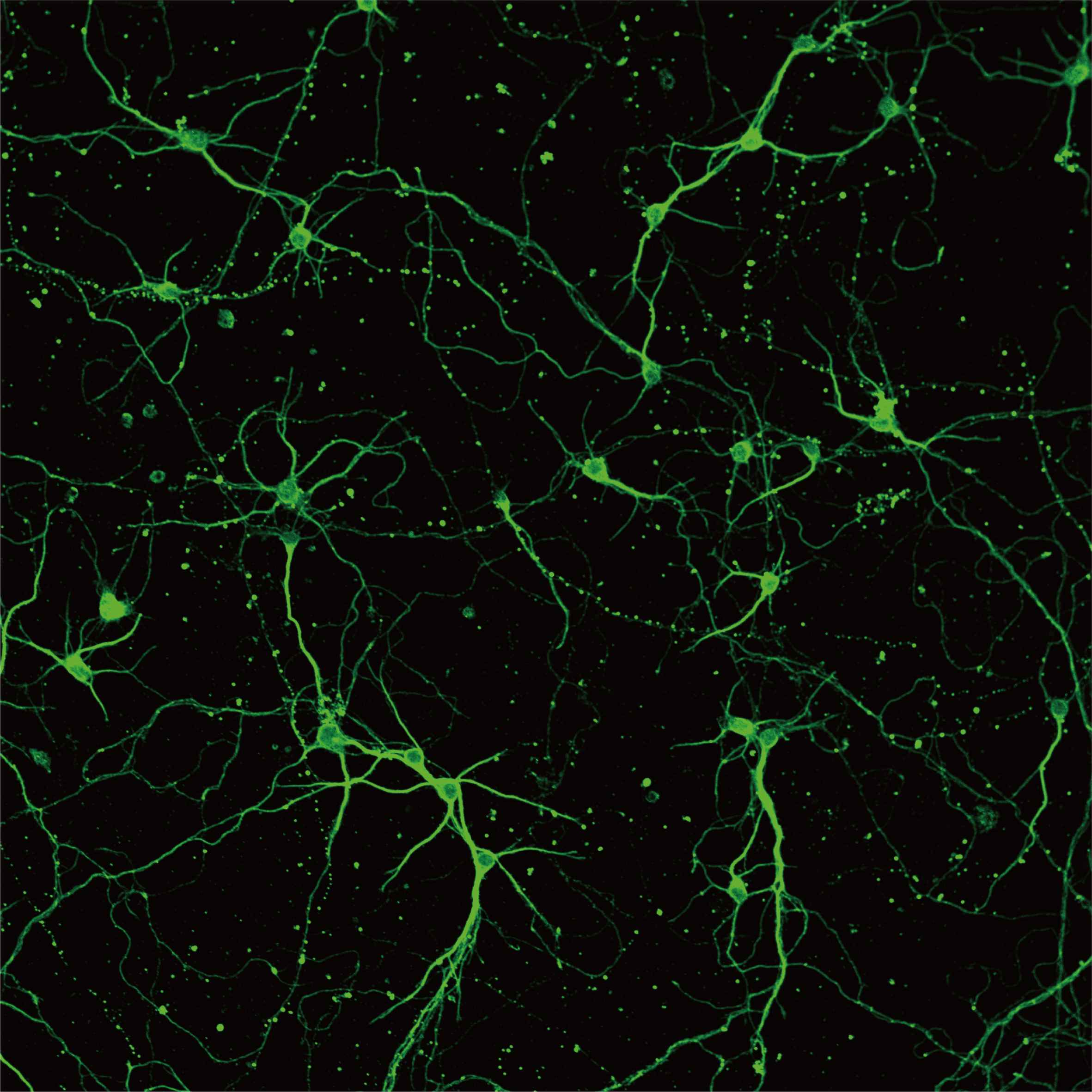

Supplement: Supplementary file 8 — Source data Fig. 6 [file 44321_2025_206_MOESM8_ESM.zip › Source data Fig 6/Fig 6/6B/WT-OGD MAP2.tif]

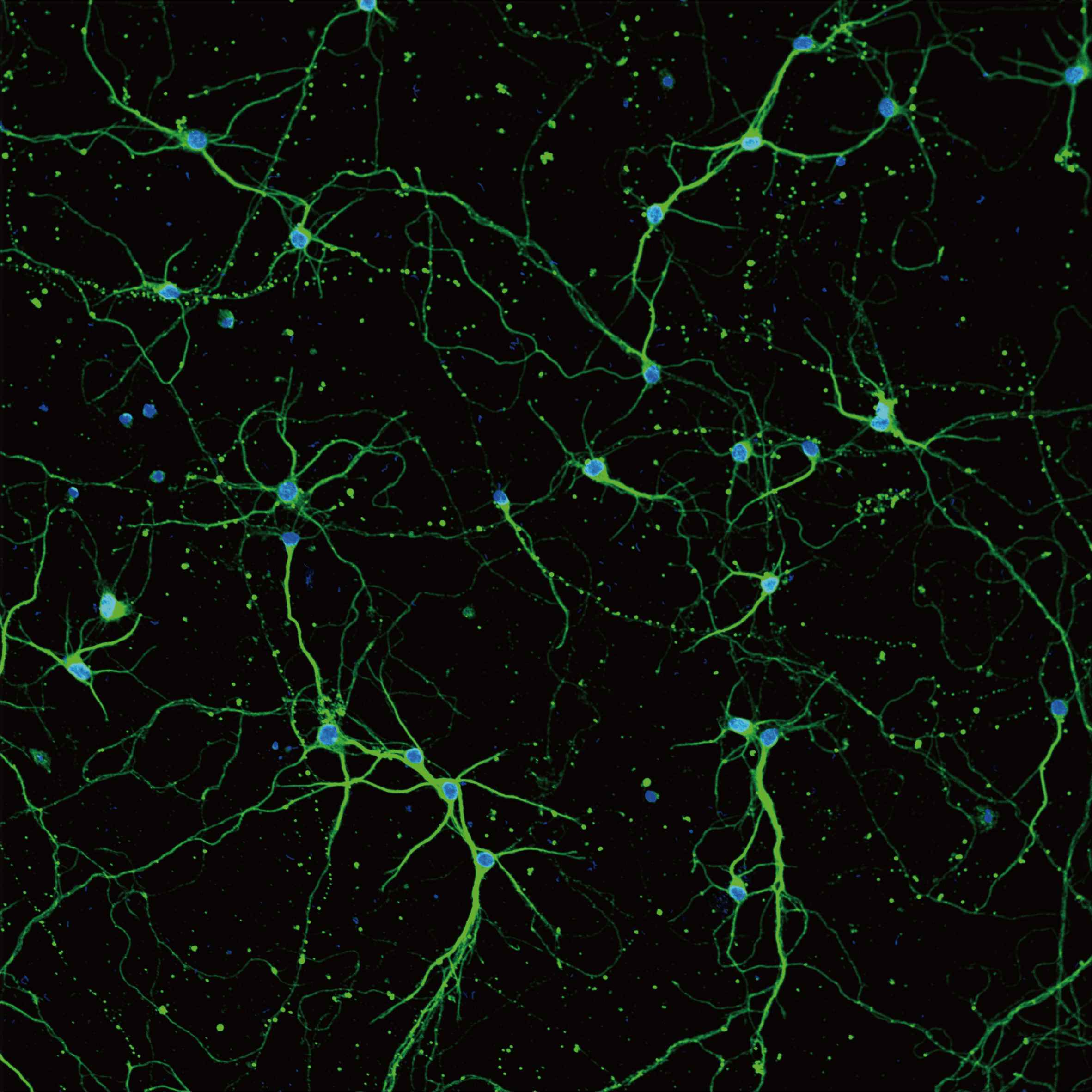

Supplement: Supplementary file 8 — Source data Fig. 6 [file 44321_2025_206_MOESM8_ESM.zip › Source data Fig 6/Fig 6/6B/WT-OGD MERGE.tif]

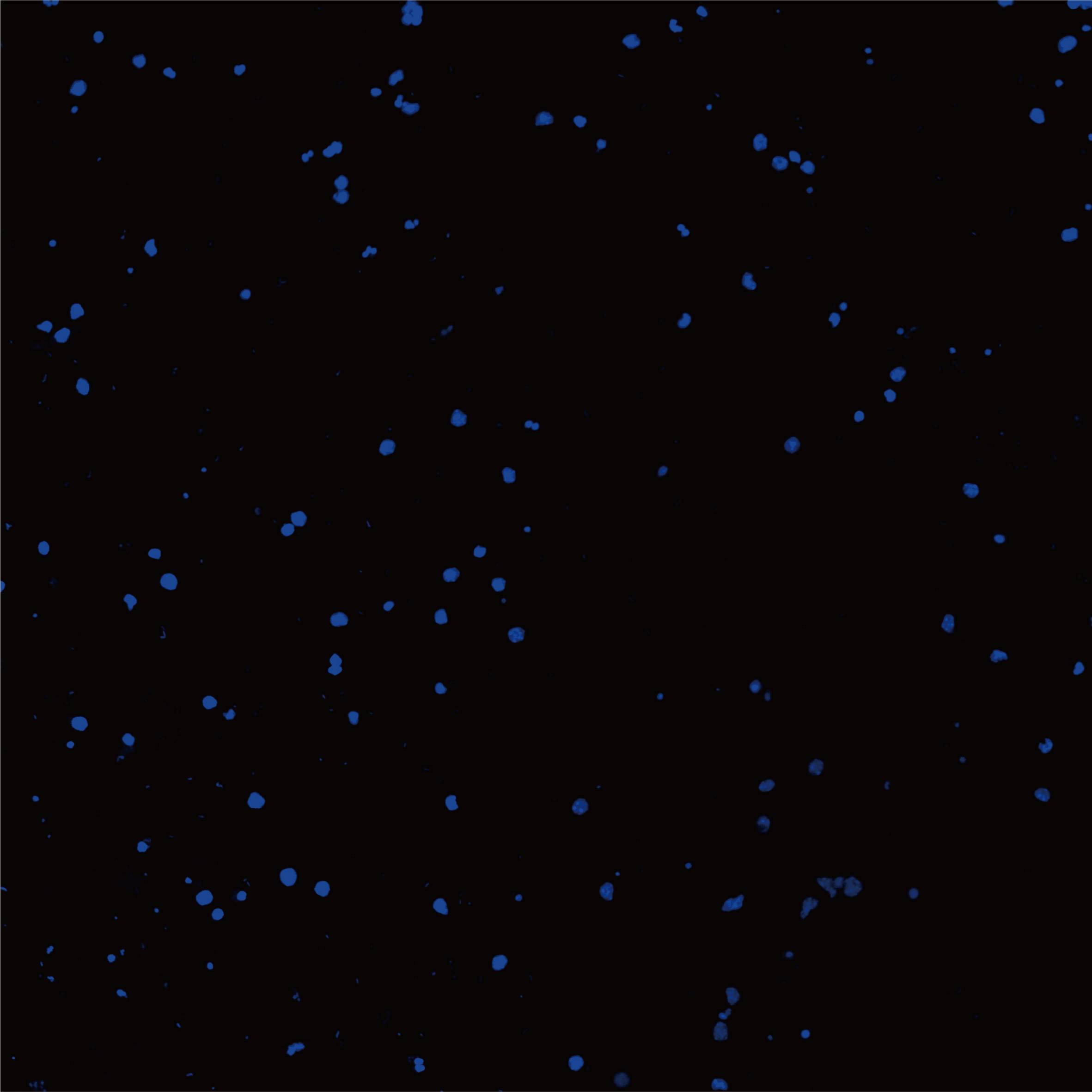

Supplement: Supplementary file 8 — Source data Fig. 6 [file 44321_2025_206_MOESM8_ESM.zip › Source data Fig 6/Fig 6/6C/KO-Normoxia DAPI.tif]

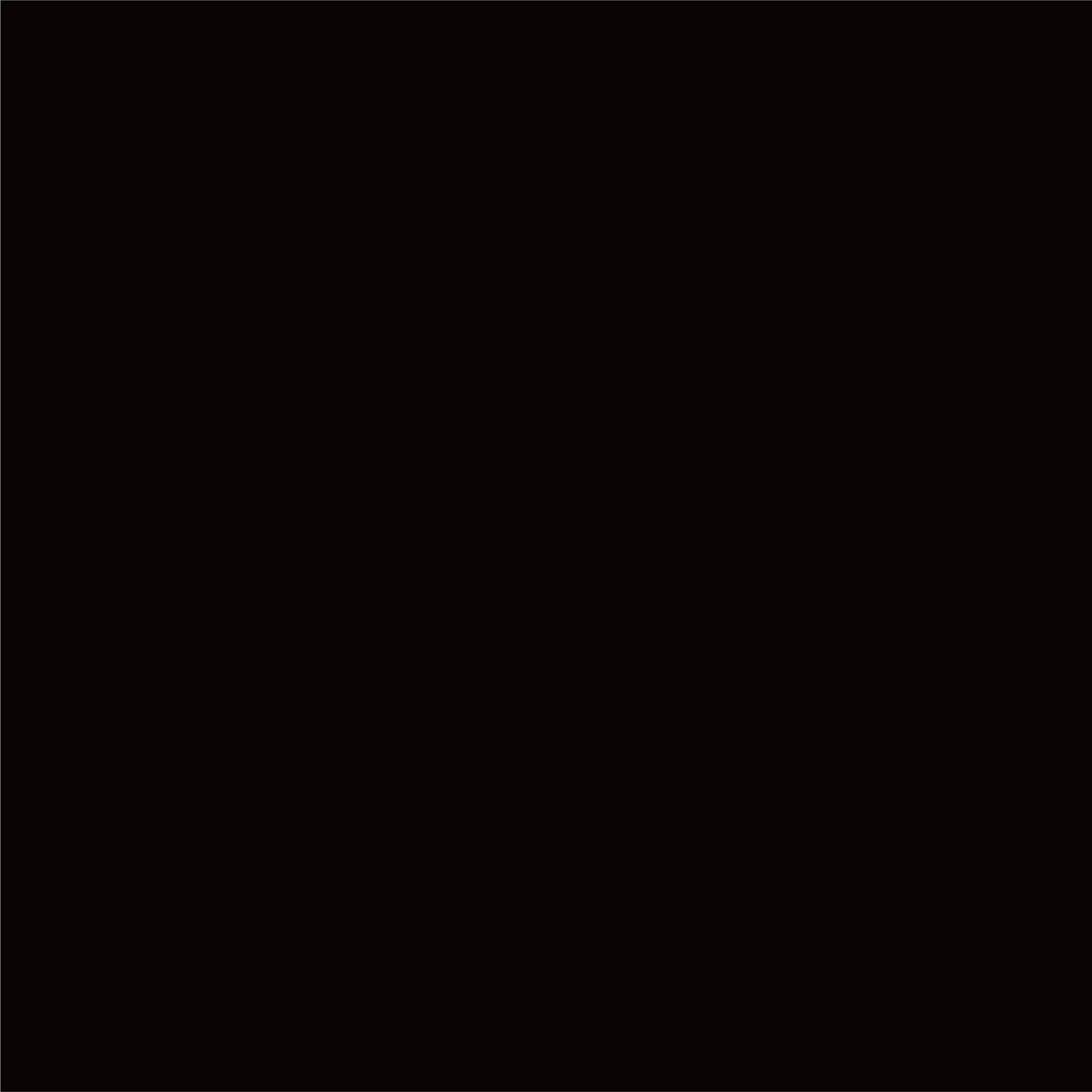

Supplement: Supplementary file 8 — Source data Fig. 6 [file 44321_2025_206_MOESM8_ESM.zip › Source data Fig 6/Fig 6/6C/KO-Normoxia P-RIPK3.tif]

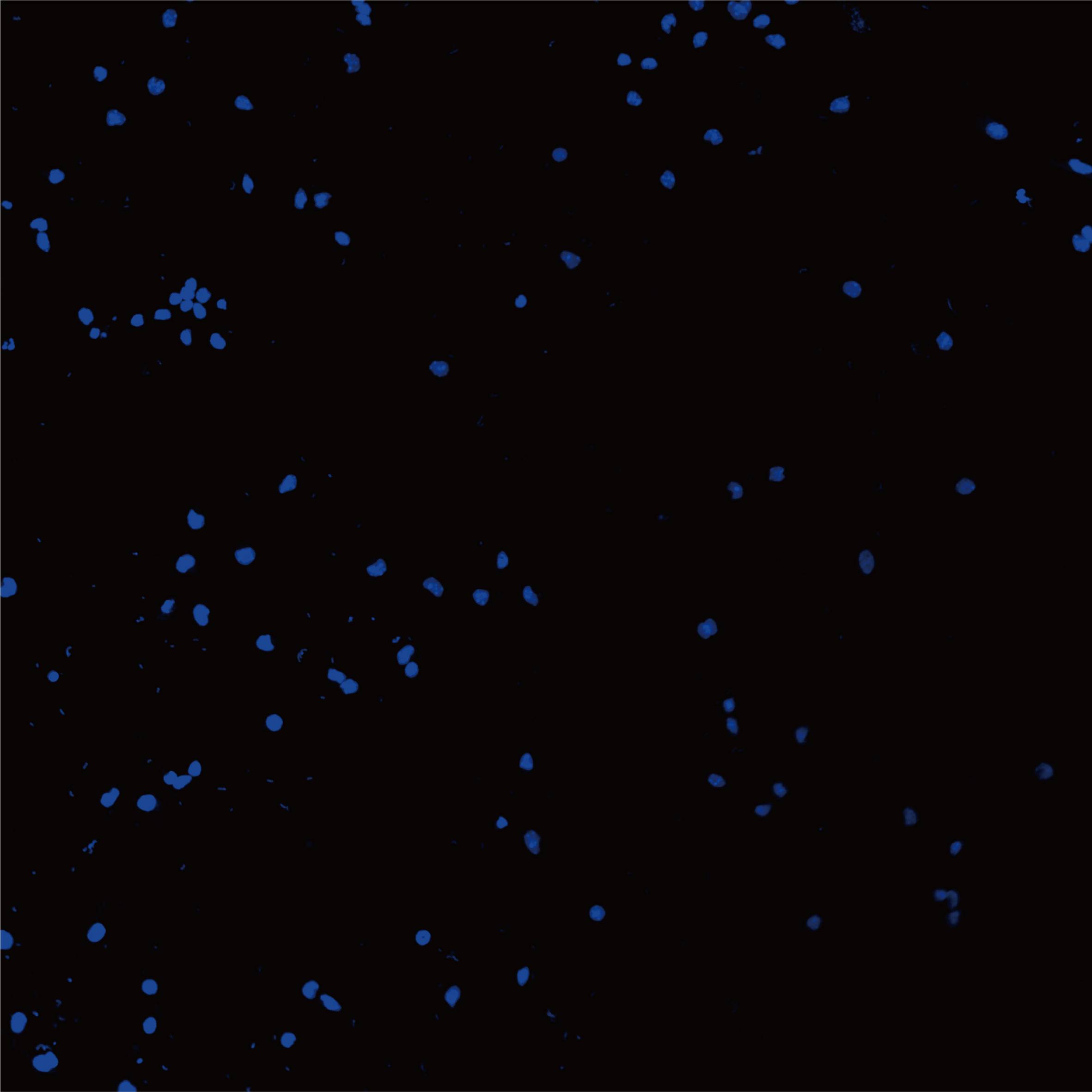

Supplement: Supplementary file 8 — Source data Fig. 6 [file 44321_2025_206_MOESM8_ESM.zip › Source data Fig 6/Fig 6/6C/KO-OGD DAPI.tif]

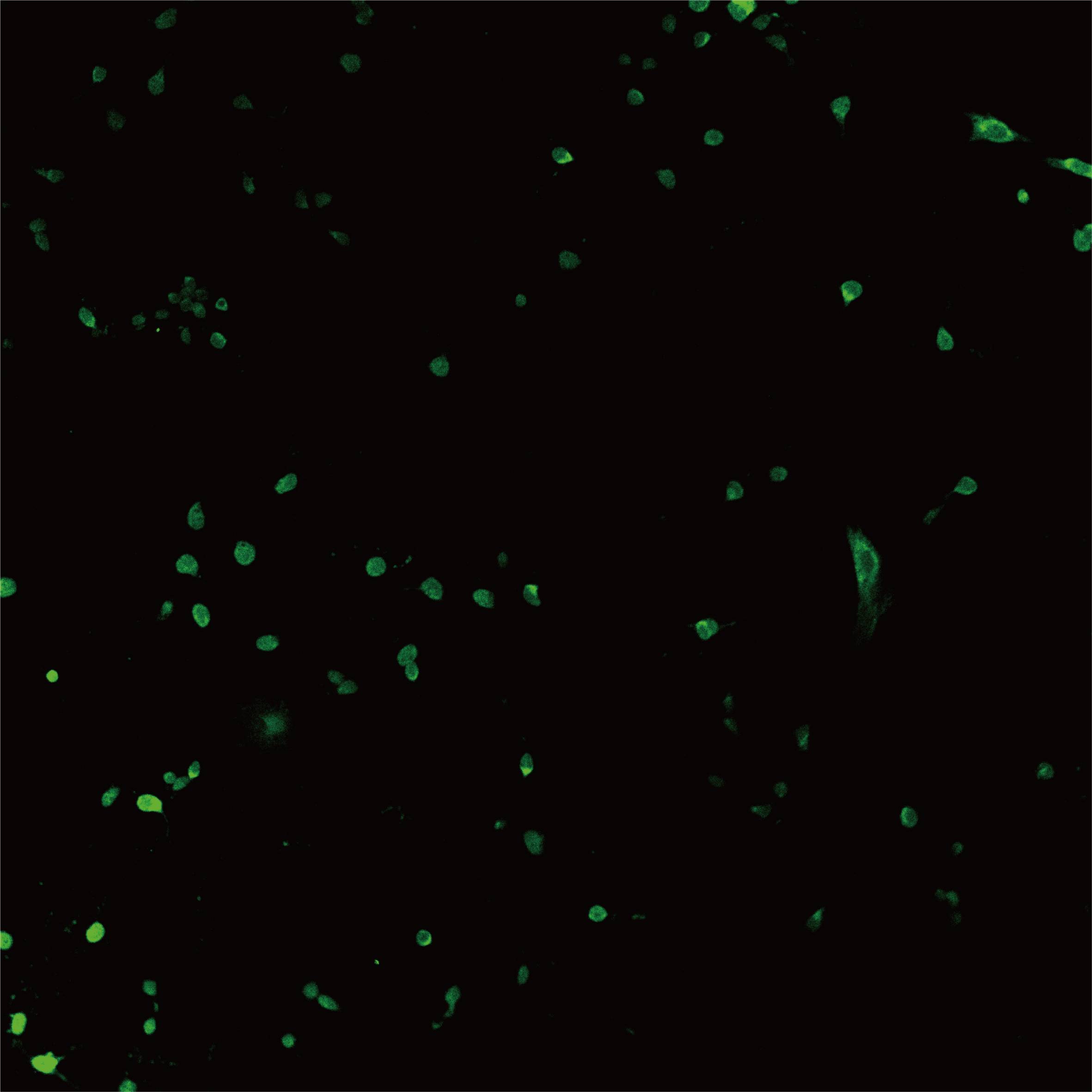

Supplement: Supplementary file 8 — Source data Fig. 6 [file 44321_2025_206_MOESM8_ESM.zip › Source data Fig 6/Fig 6/6C/KO-OGD P-RIPK3.tif]

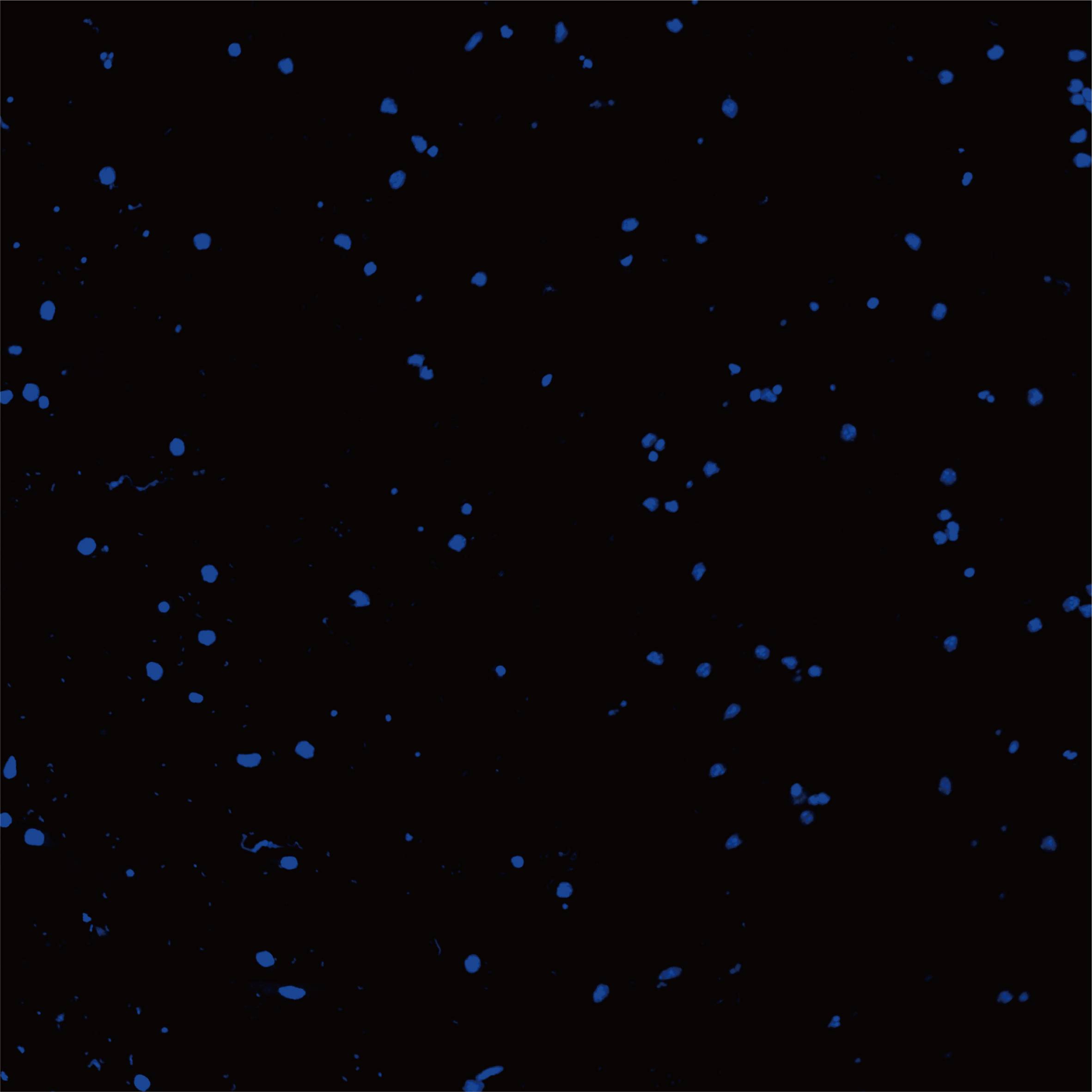

Supplement: Supplementary file 8 — Source data Fig. 6 [file 44321_2025_206_MOESM8_ESM.zip › Source data Fig 6/Fig 6/6C/WT-Normoxia DAPI.tif]

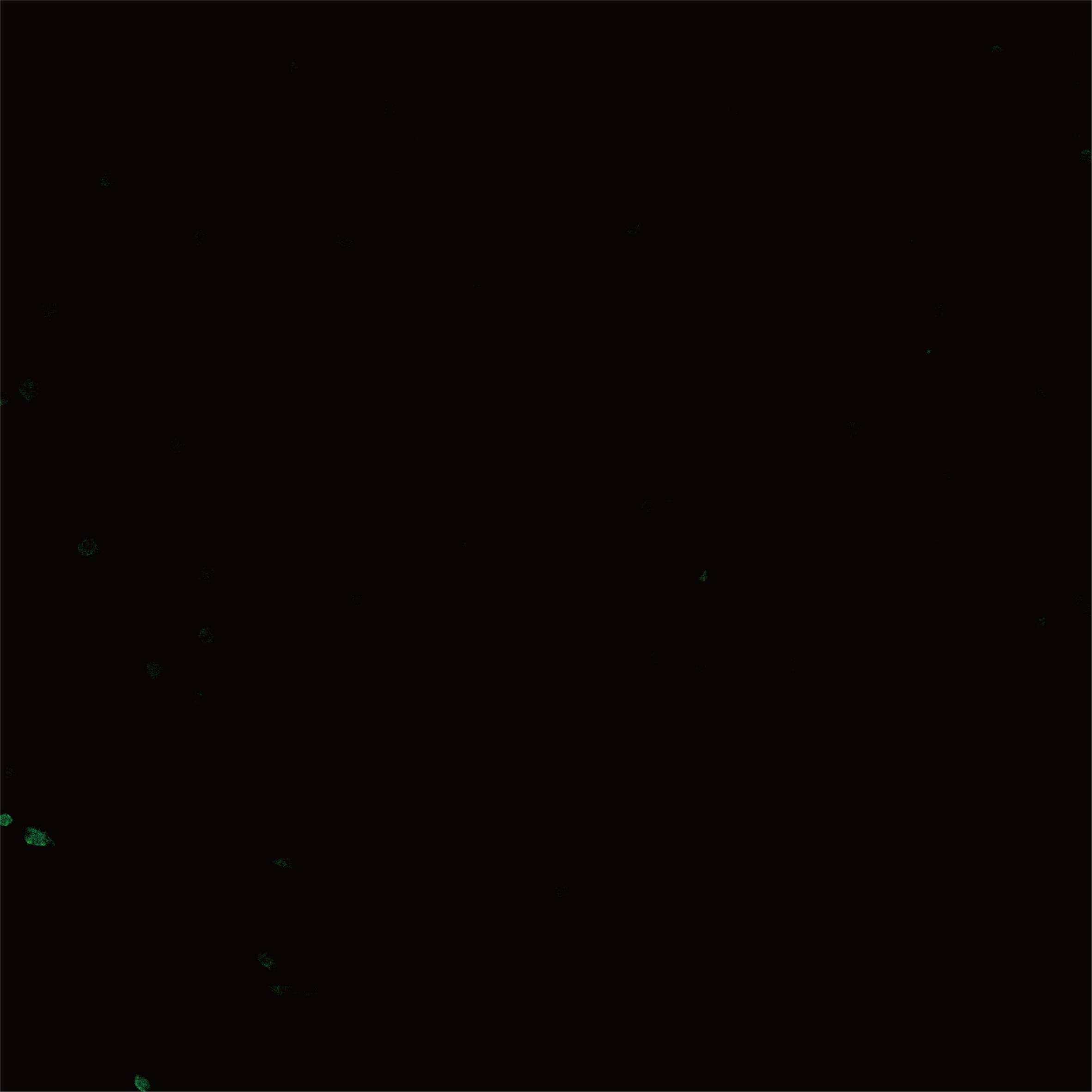

Supplement: Supplementary file 8 — Source data Fig. 6 [file 44321_2025_206_MOESM8_ESM.zip › Source data Fig 6/Fig 6/6C/WT-Normoxia P-RIPK3.tif]

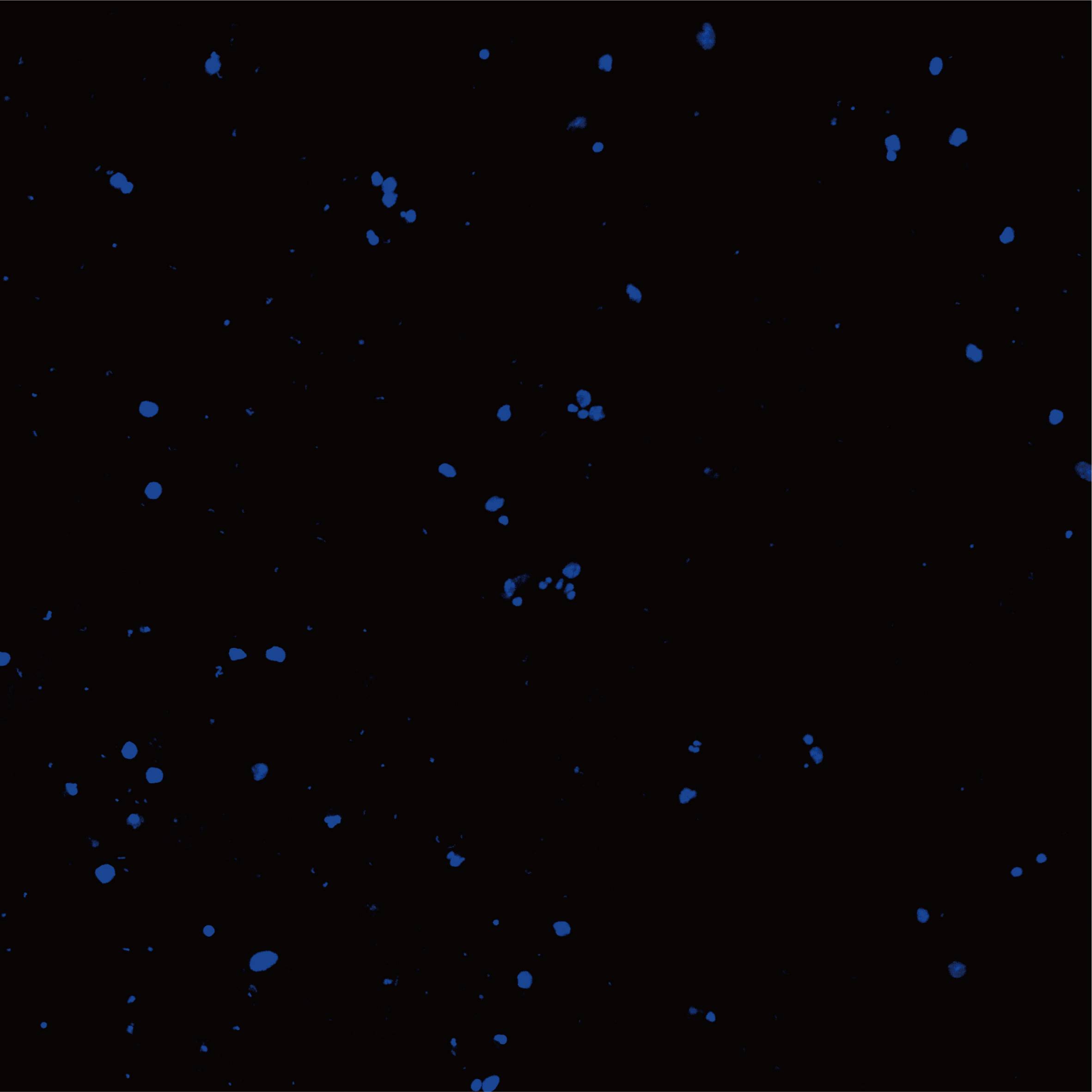

Supplement: Supplementary file 8 — Source data Fig. 6 [file 44321_2025_206_MOESM8_ESM.zip › Source data Fig 6/Fig 6/6C/WT-OGD DAPI.tif]

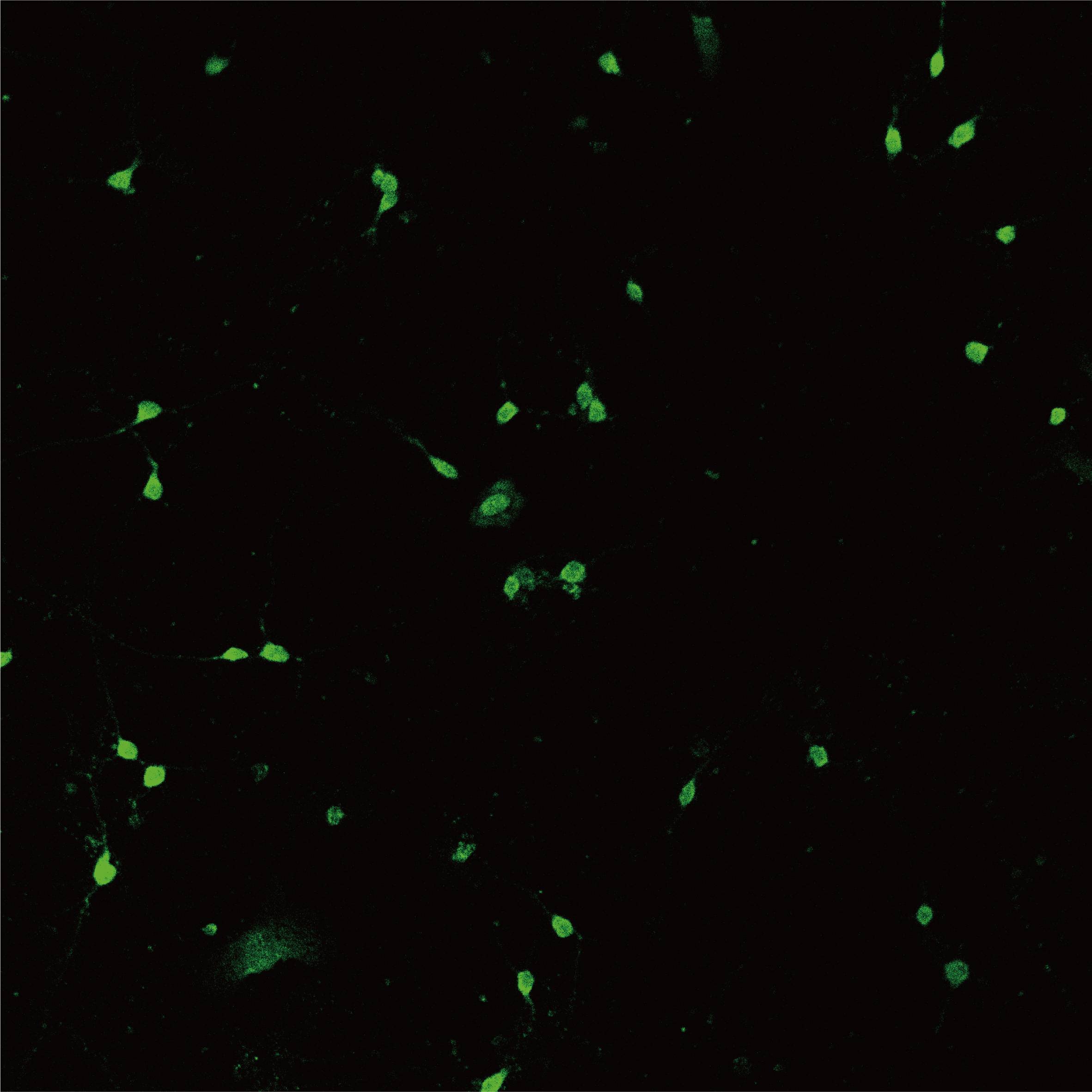

Supplement: Supplementary file 8 — Source data Fig. 6 [file 44321_2025_206_MOESM8_ESM.zip › Source data Fig 6/Fig 6/6C/WT-OGD P-RIPK3.tif]

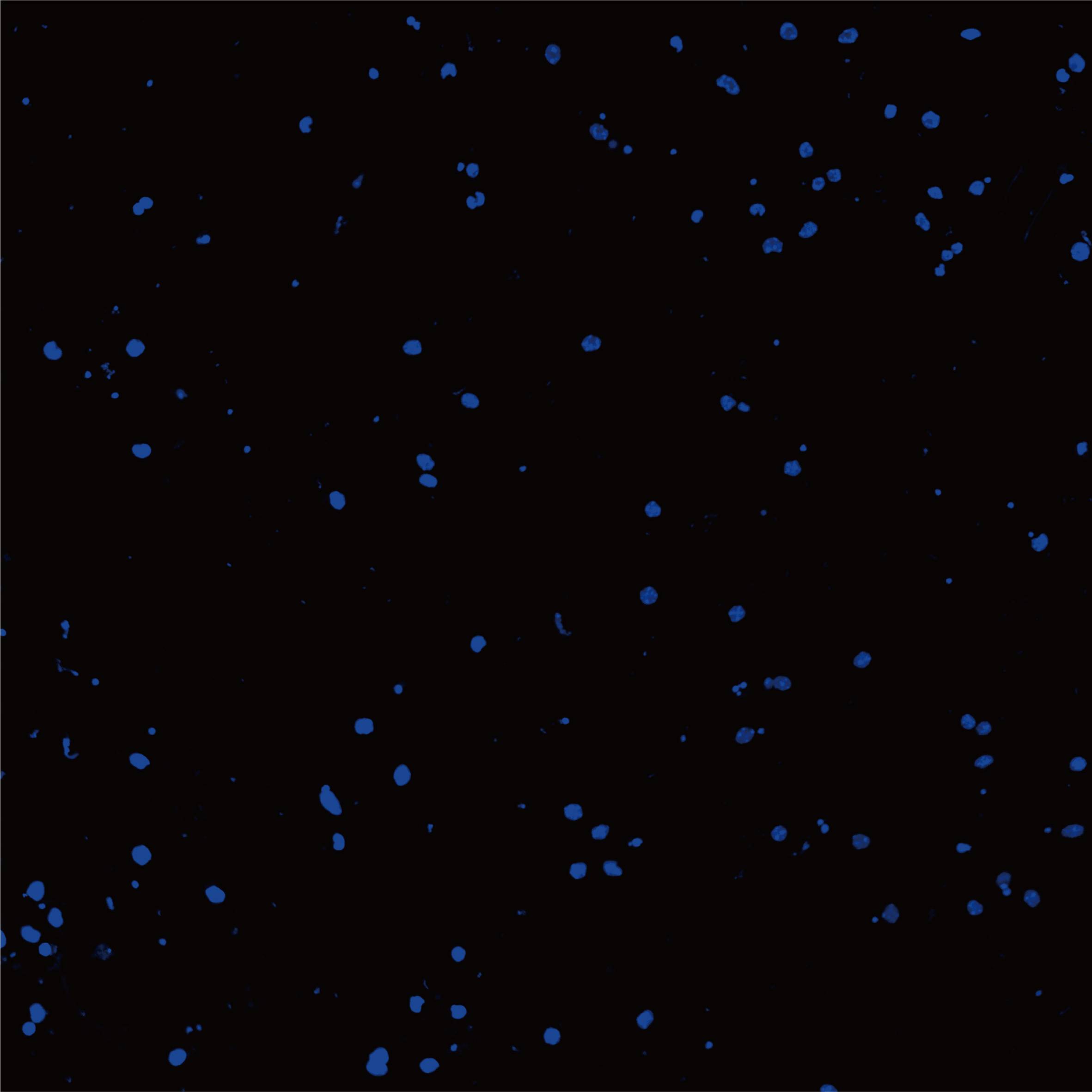

Supplement: Supplementary file 8 — Source data Fig. 6 [file 44321_2025_206_MOESM8_ESM.zip › Source data Fig 6/Fig 6/6D/KO-Normoxia-DAPI.tif]

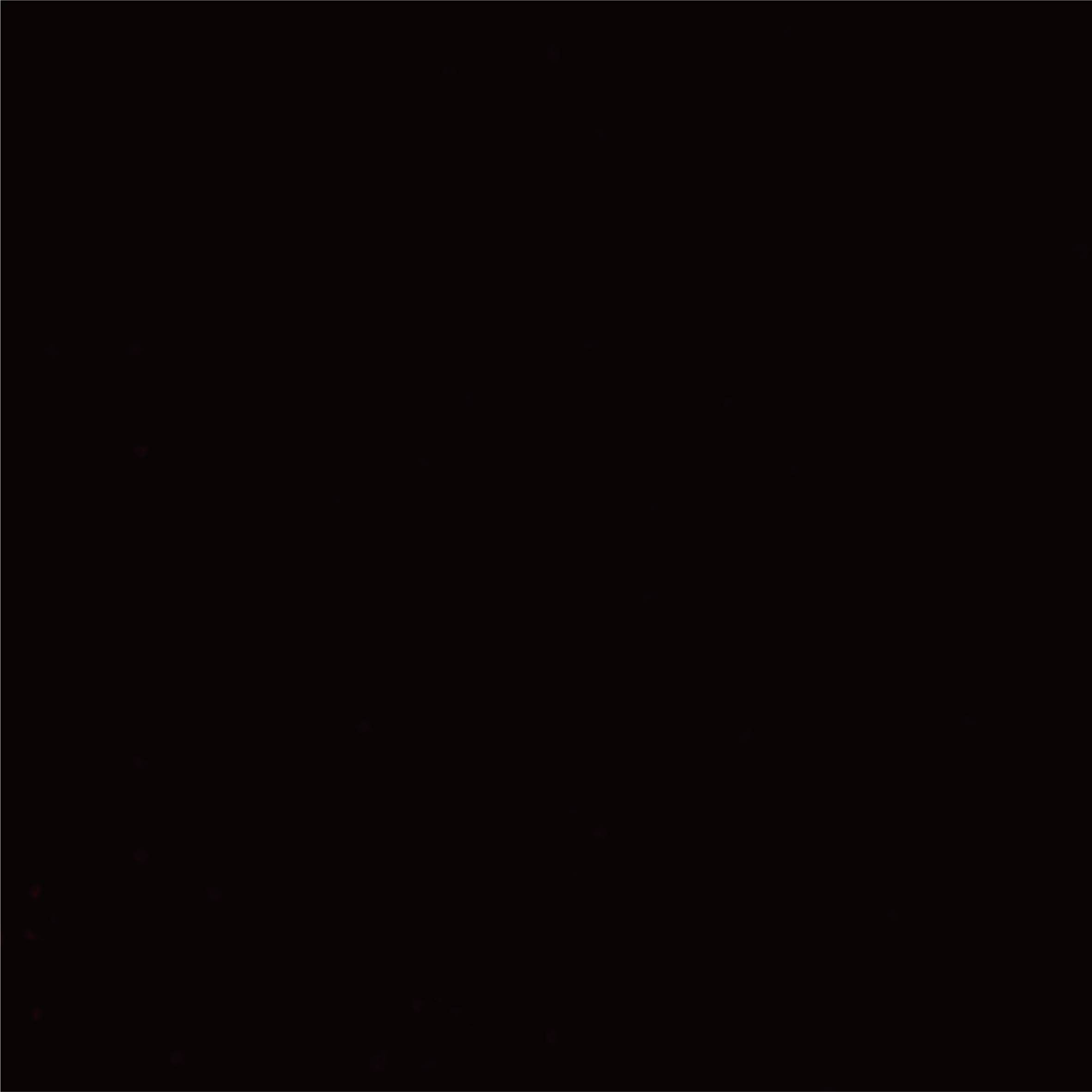

Supplement: Supplementary file 8 — Source data Fig. 6 [file 44321_2025_206_MOESM8_ESM.zip › Source data Fig 6/Fig 6/6D/KO-Normoxia-P-MLKL.tif]

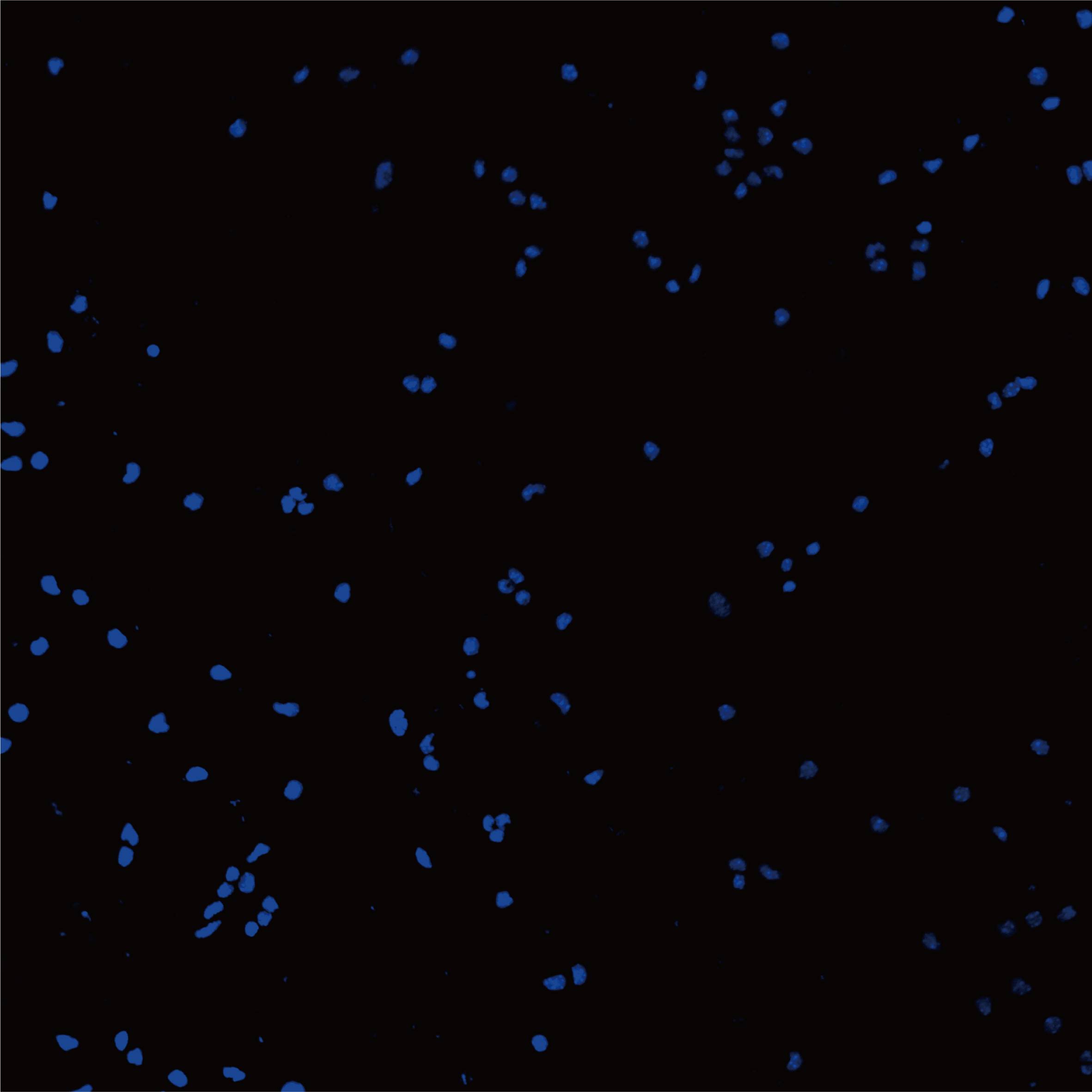

Supplement: Supplementary file 8 — Source data Fig. 6 [file 44321_2025_206_MOESM8_ESM.zip › Source data Fig 6/Fig 6/6D/KO-OGD-DAPI.tif]

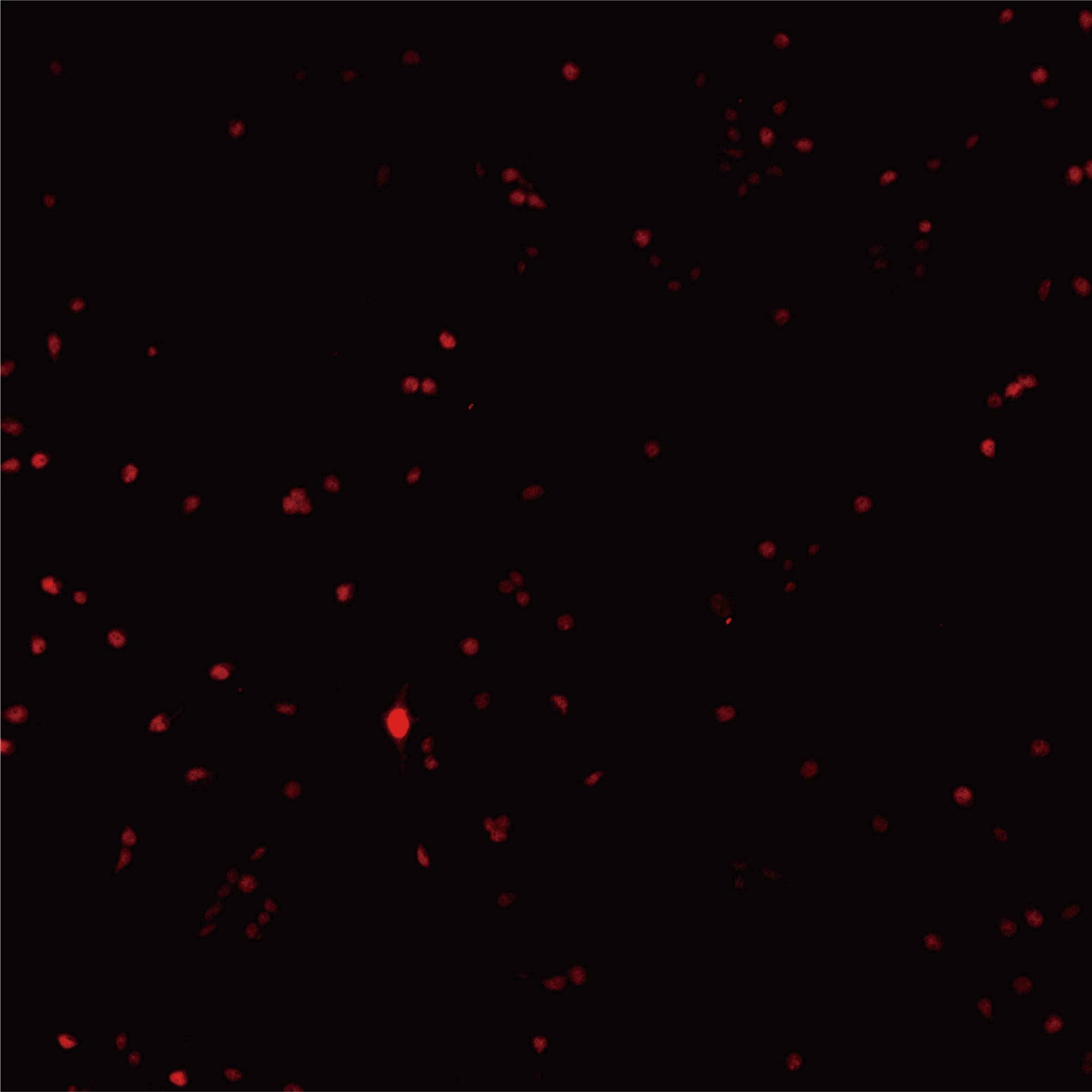

Supplement: Supplementary file 8 — Source data Fig. 6 [file 44321_2025_206_MOESM8_ESM.zip › Source data Fig 6/Fig 6/6D/KO-OGD-P-MLKL.tif]

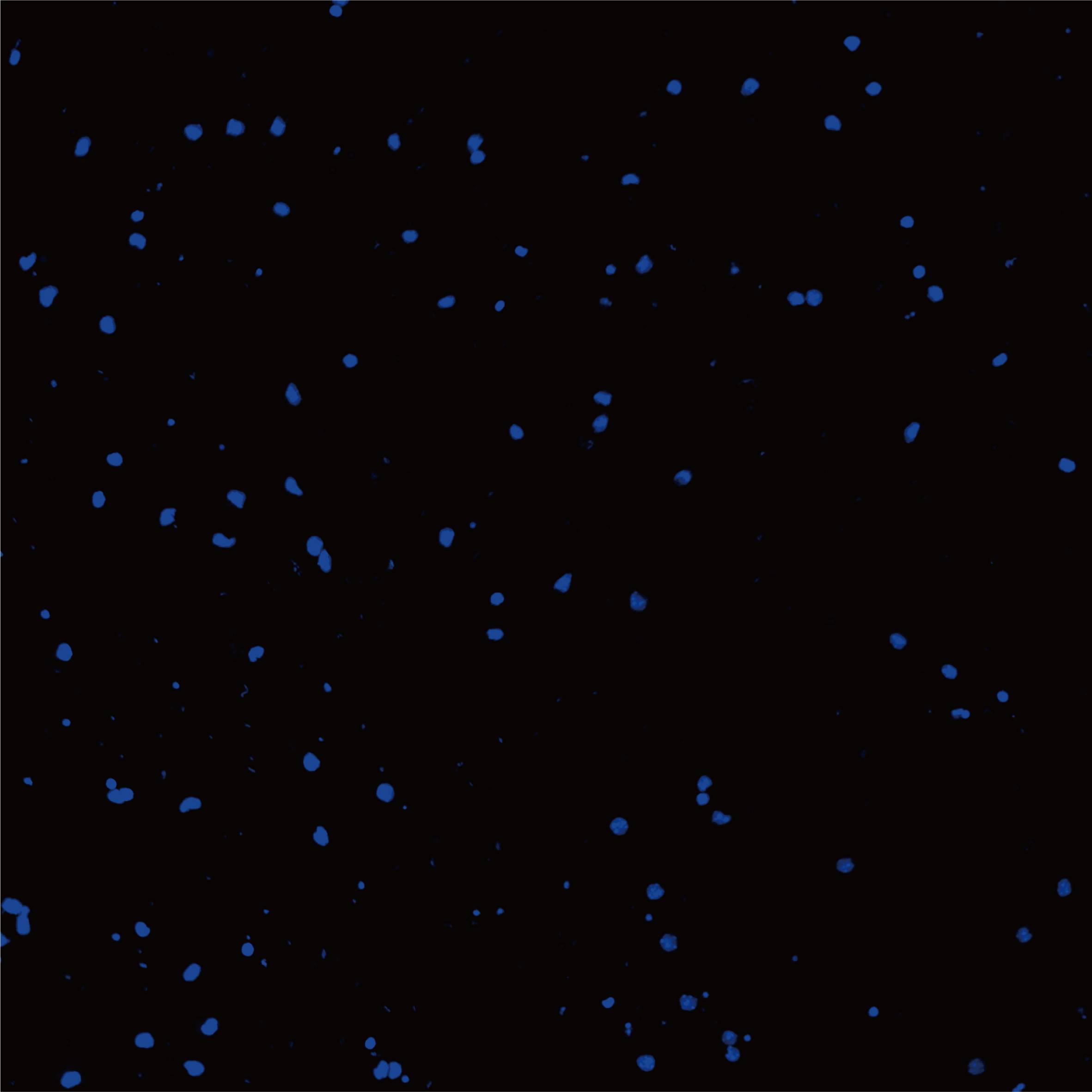

Supplement: Supplementary file 8 — Source data Fig. 6 [file 44321_2025_206_MOESM8_ESM.zip › Source data Fig 6/Fig 6/6D/WT-Normoxia-DAPI.tif]

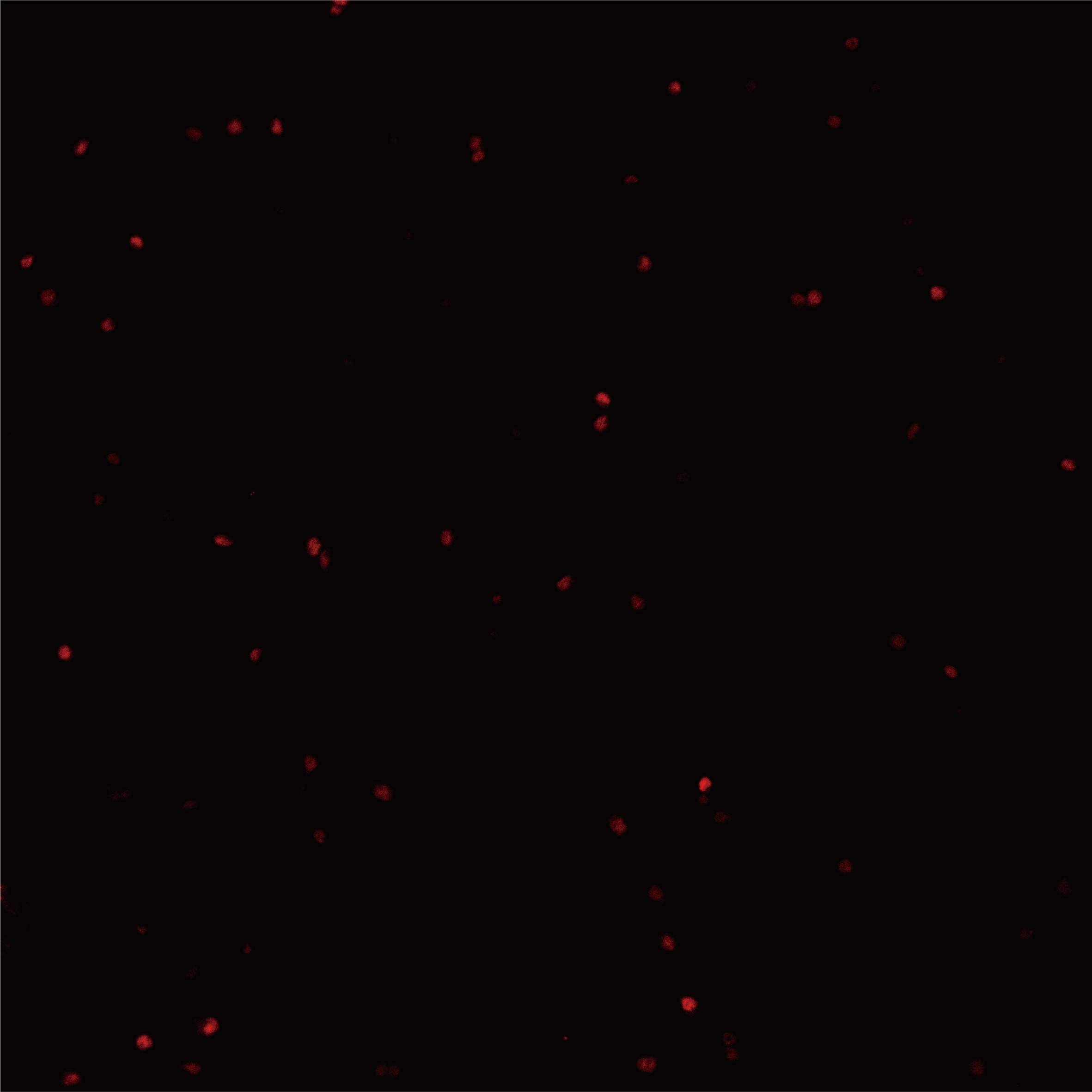

Supplement: Supplementary file 8 — Source data Fig. 6 [file 44321_2025_206_MOESM8_ESM.zip › Source data Fig 6/Fig 6/6D/WT-Normoxia-P-MLKL.tif]

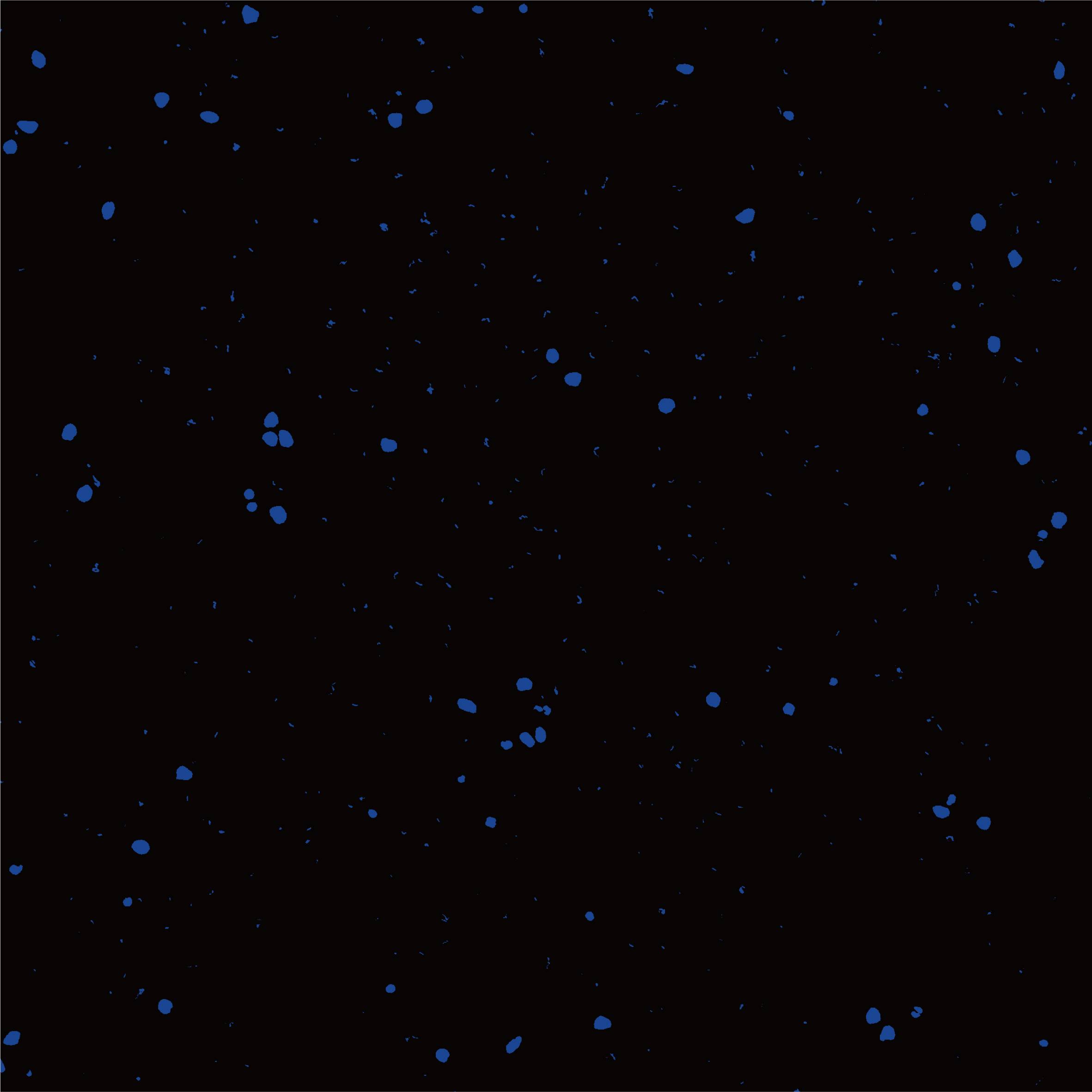

Supplement: Supplementary file 8 — Source data Fig. 6 [file 44321_2025_206_MOESM8_ESM.zip › Source data Fig 6/Fig 6/6D/WT-OGD-DAPI.tif]

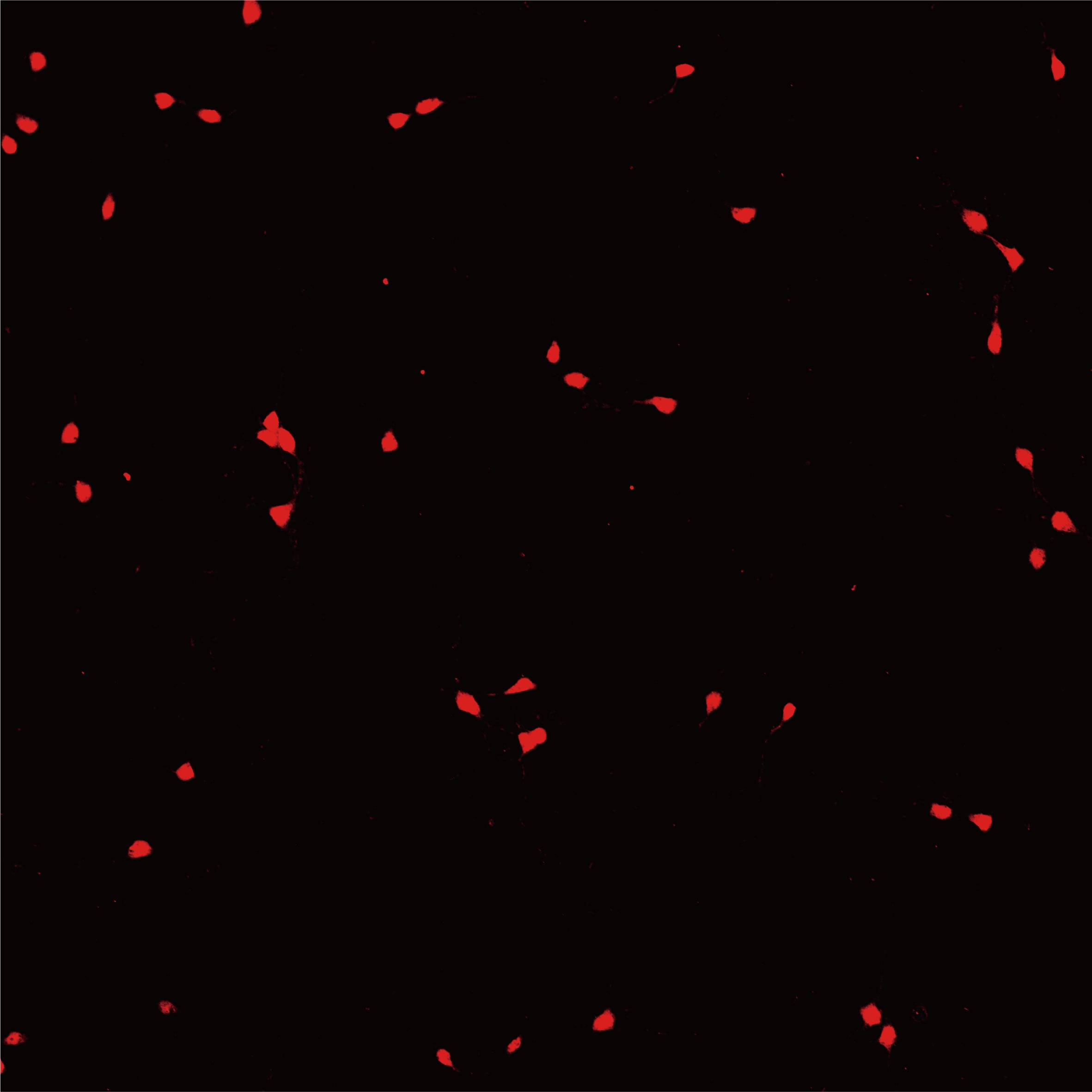

Supplement: Supplementary file 8 — Source data Fig. 6 [file 44321_2025_206_MOESM8_ESM.zip › Source data Fig 6/Fig 6/6D/WT-OGD-P-MLKL.tif]

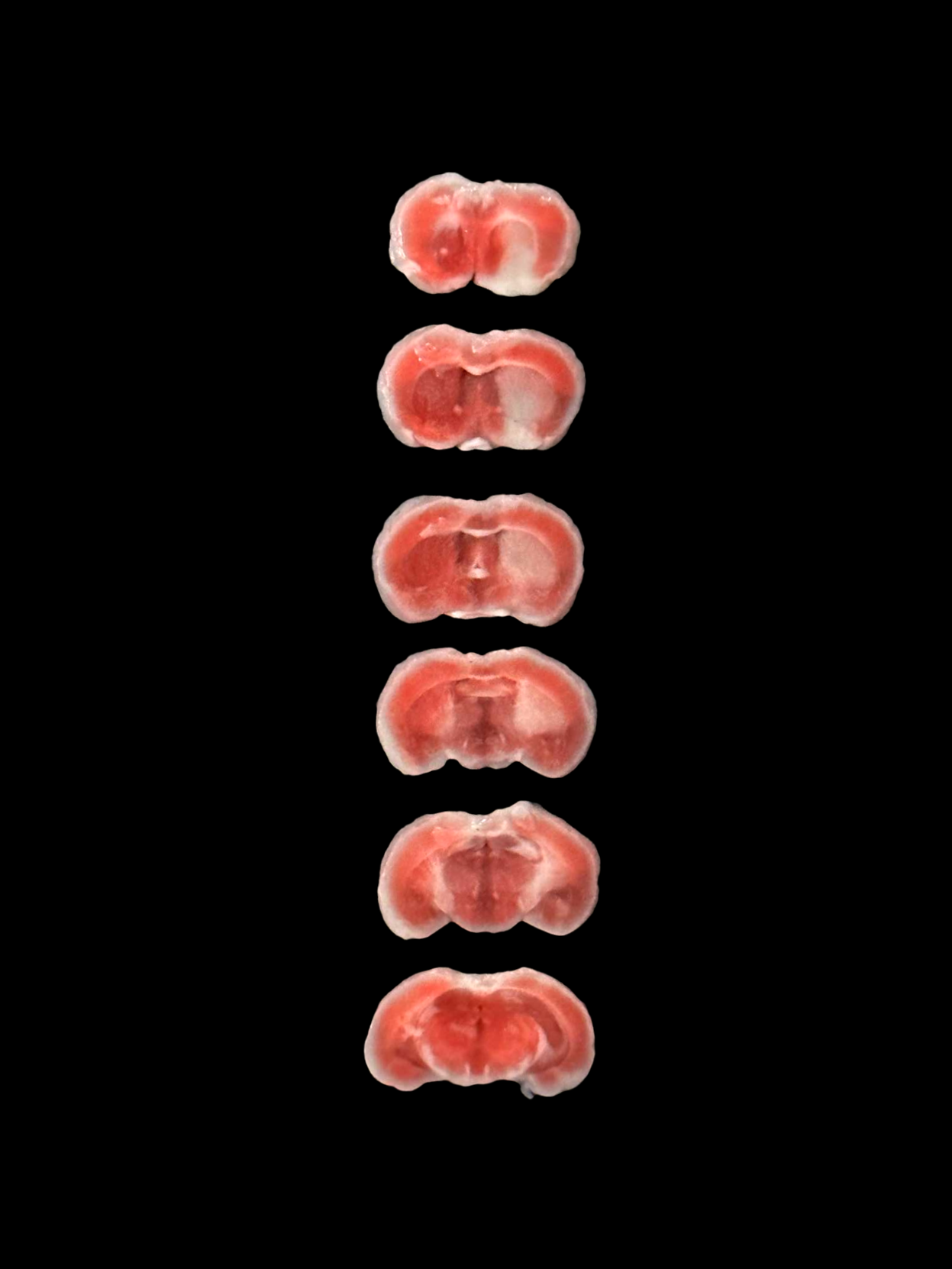

Supplement: Supplementary file 9 — Source data Fig. 7 [file 44321_2025_206_MOESM9_ESM.zip › Source data Fig 7/Fig 7/7B/MCAO-CON-KO.tif]

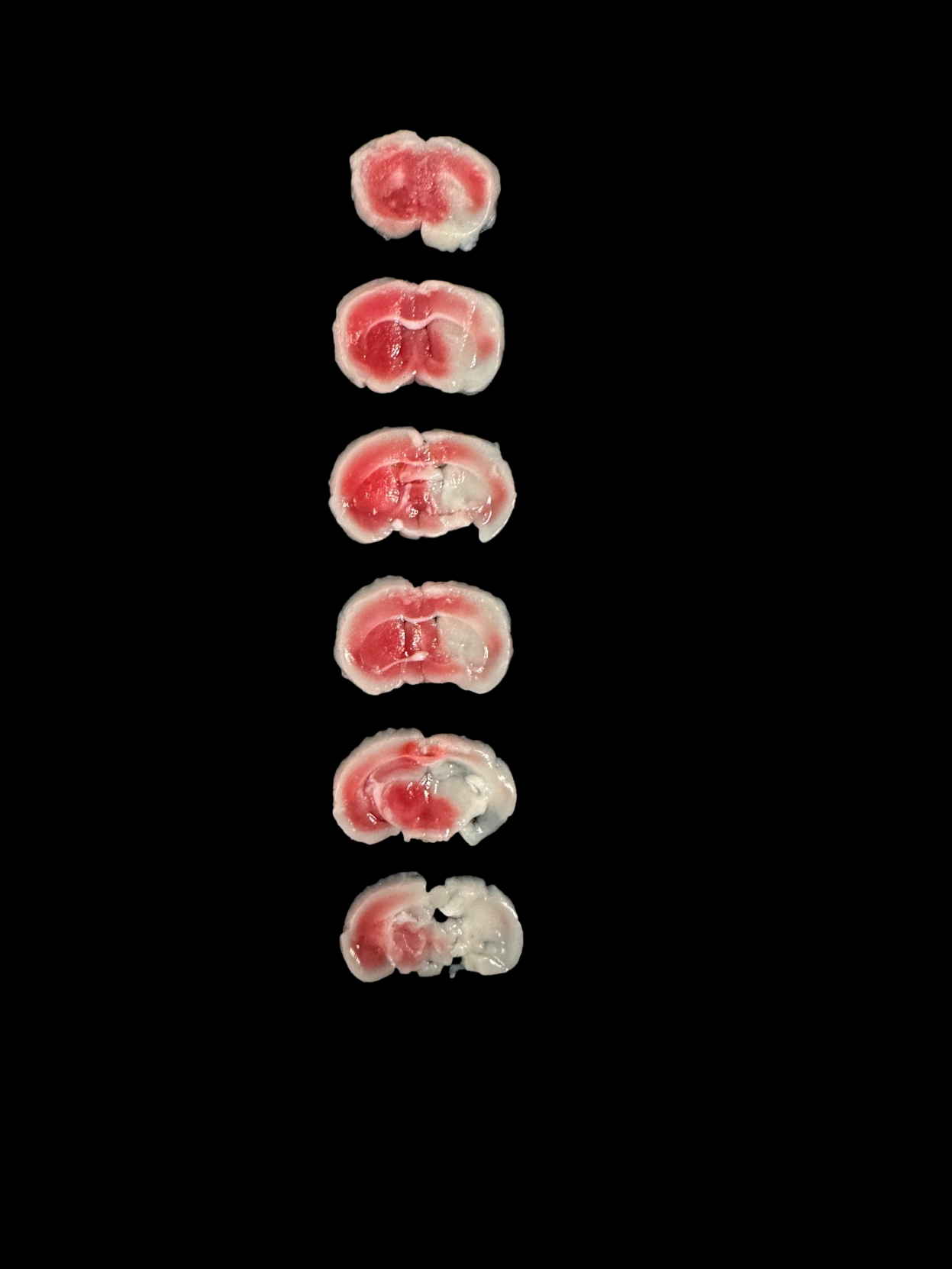

Supplement: Supplementary file 9 — Source data Fig. 7 [file 44321_2025_206_MOESM9_ESM.zip › Source data Fig 7/Fig 7/7B/MCAO-CON-WT.tif]

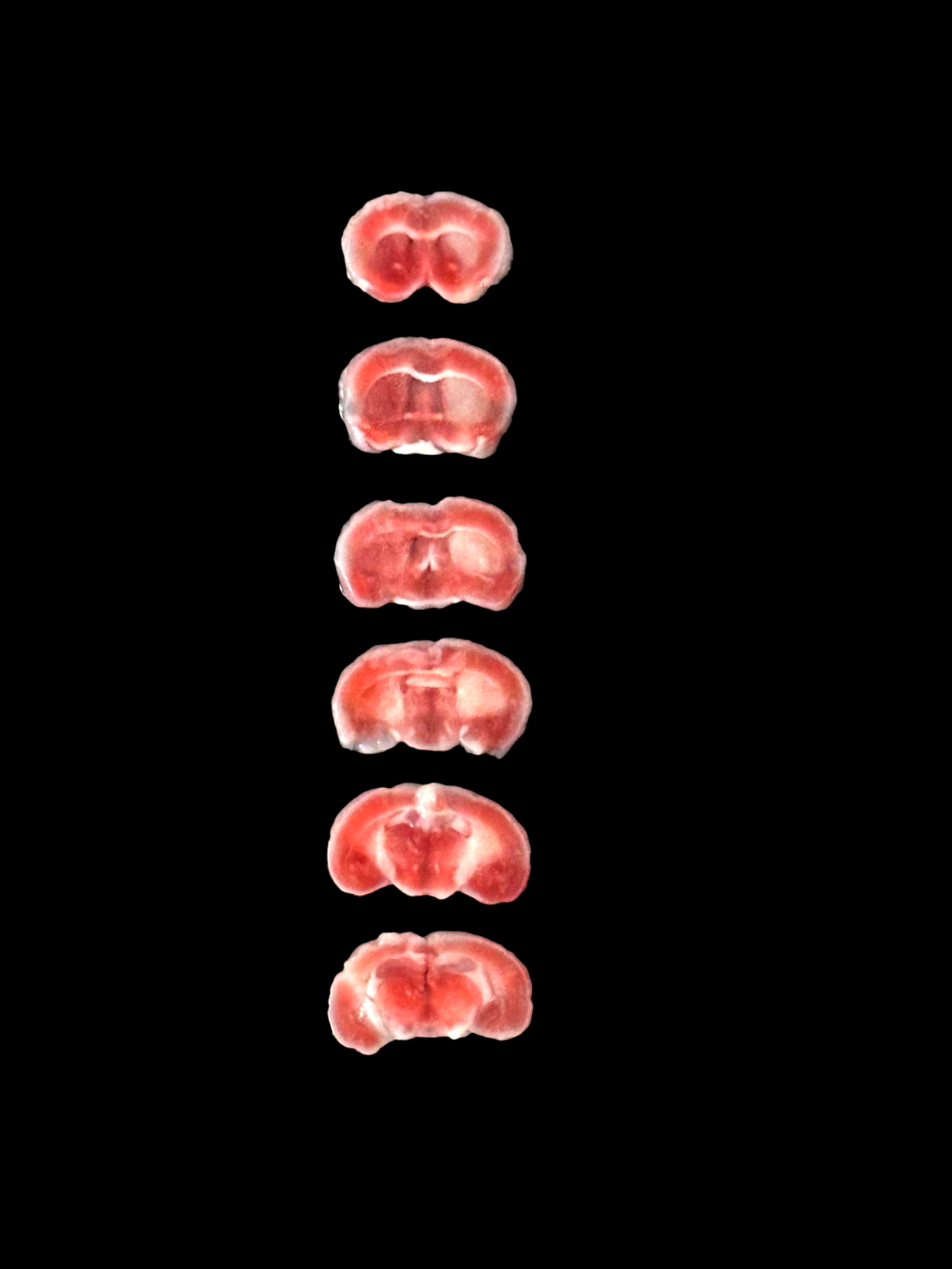

Supplement: Supplementary file 9 — Source data Fig. 7 [file 44321_2025_206_MOESM9_ESM.zip › Source data Fig 7/Fig 7/7B/MCAO-GSK-872-KO-10.tif]

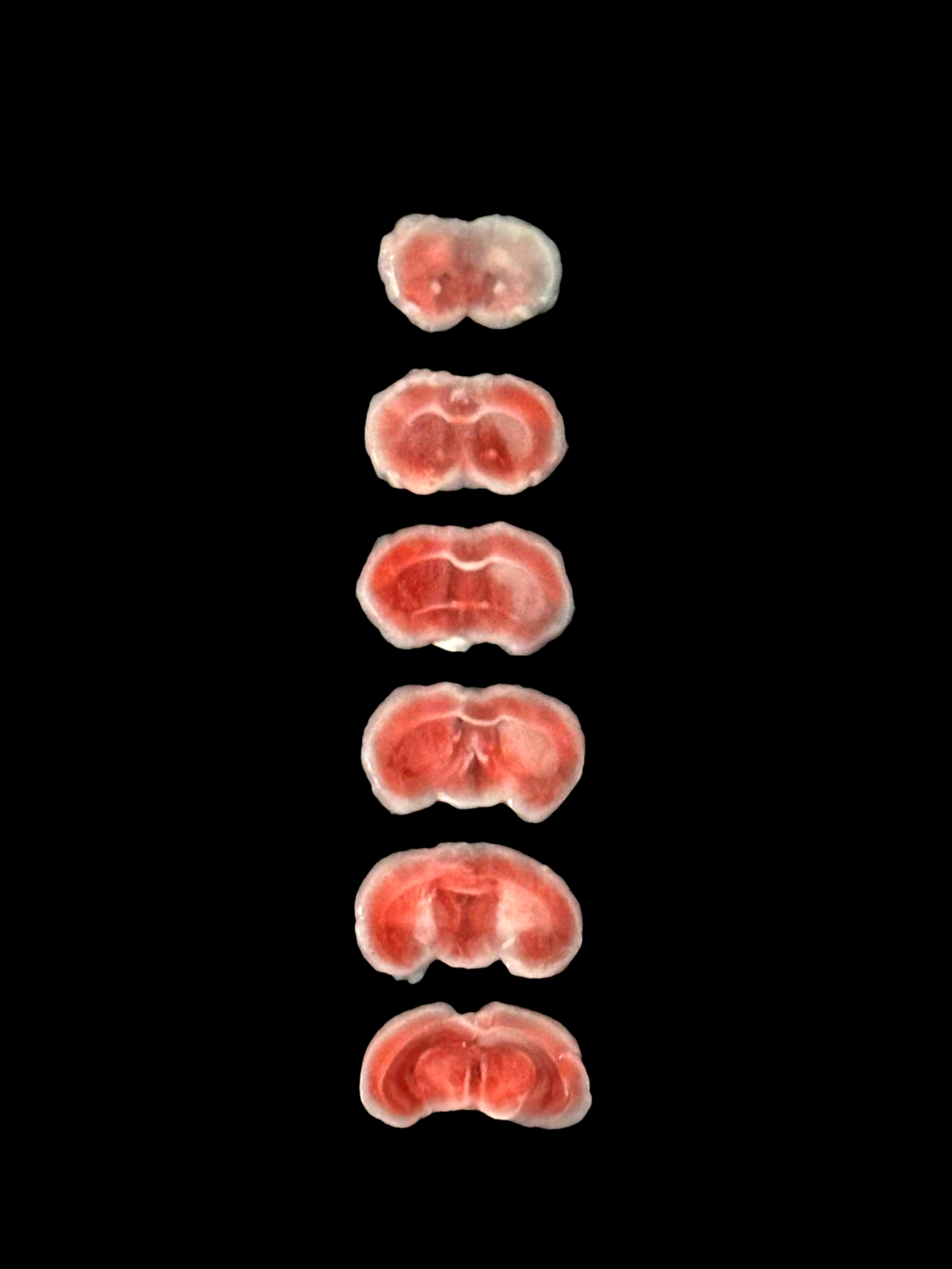

Supplement: Supplementary file 9 — Source data Fig. 7 [file 44321_2025_206_MOESM9_ESM.zip › Source data Fig 7/Fig 7/7B/MCAO-GSK-872-KO-20.tif]

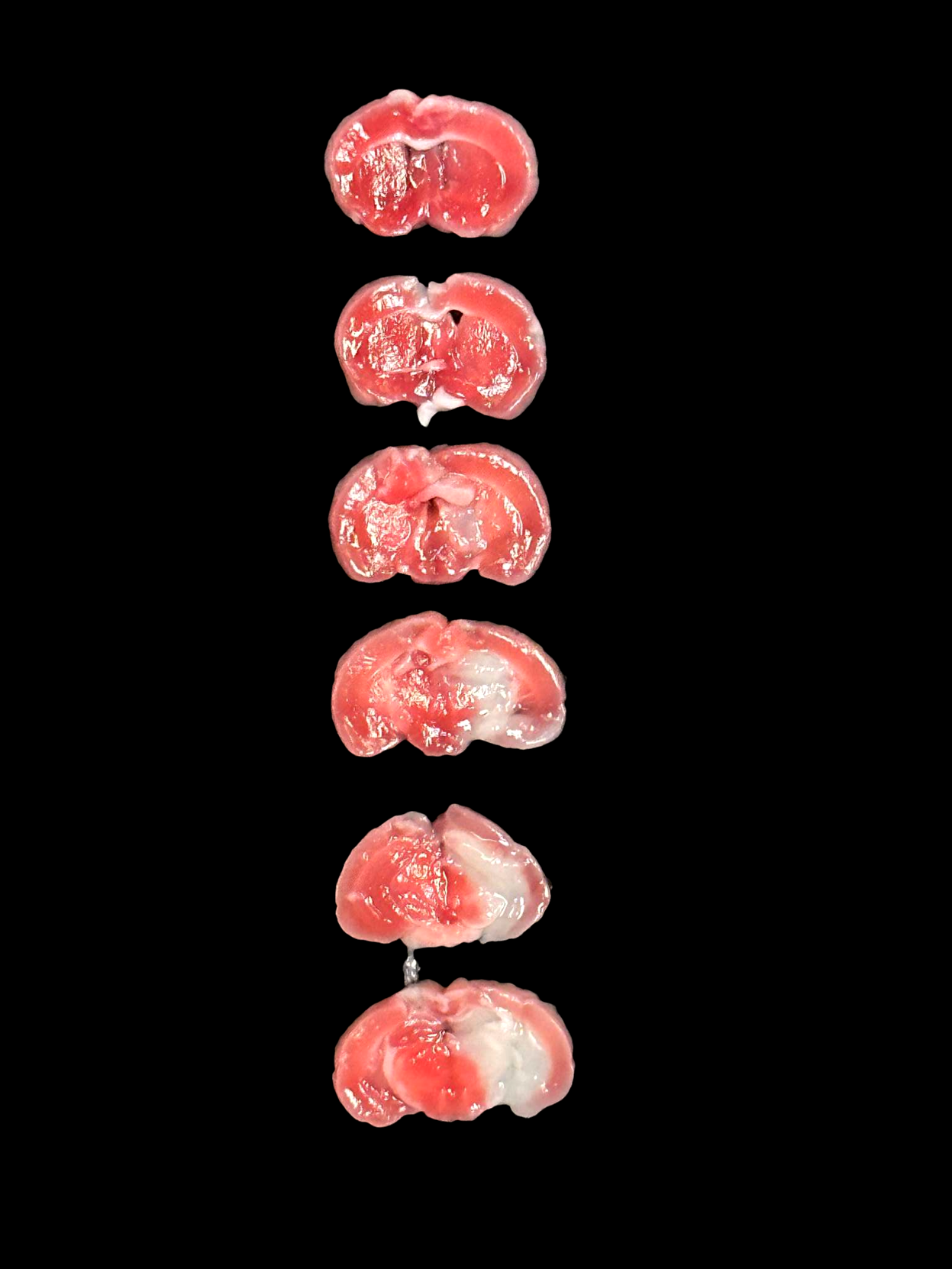

Supplement: Supplementary file 9 — Source data Fig. 7 [file 44321_2025_206_MOESM9_ESM.zip › Source data Fig 7/Fig 7/7B/MCAO-GSK-872-WT-10.tif]

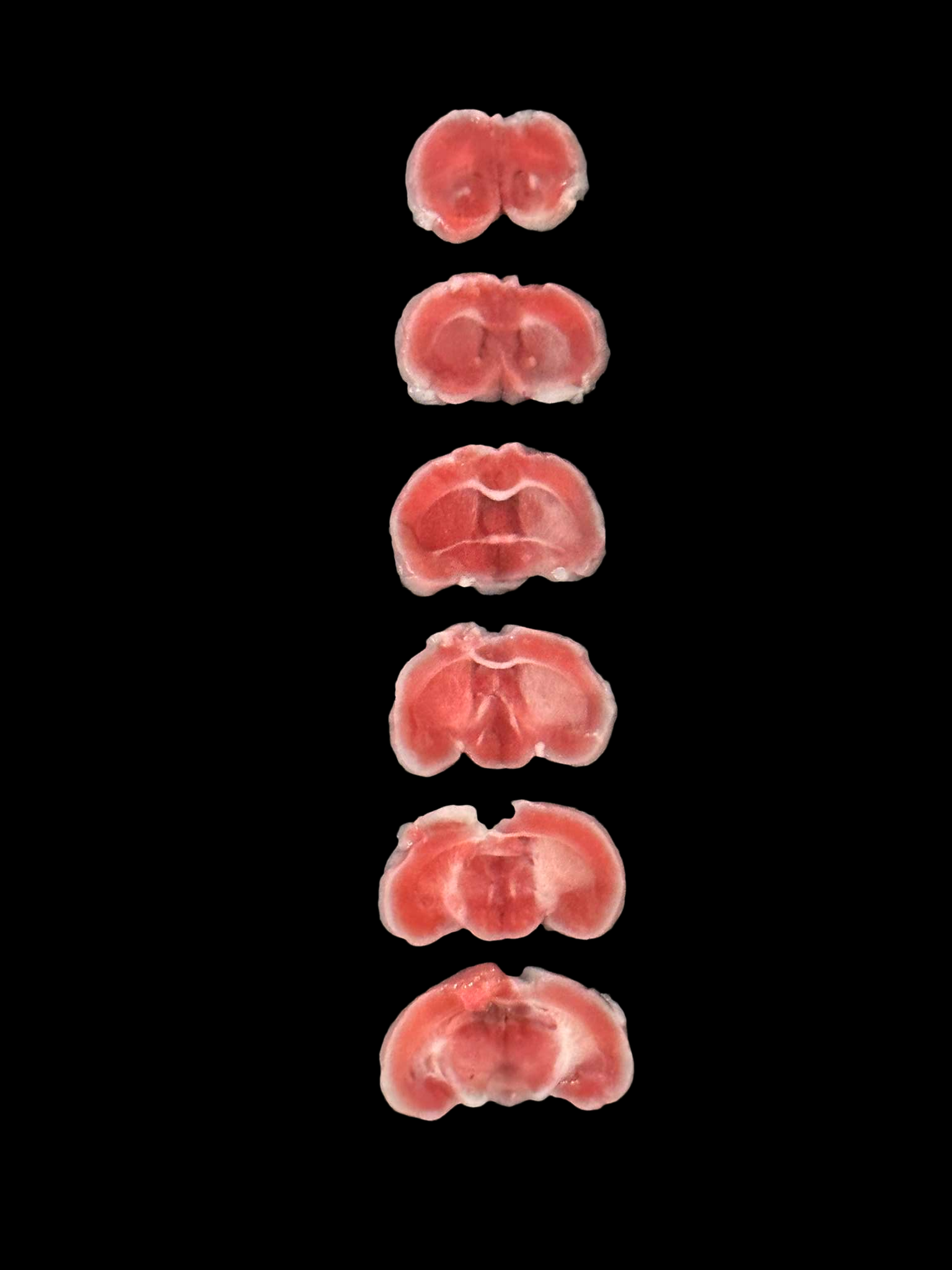

Supplement: Supplementary file 9 — Source data Fig. 7 [file 44321_2025_206_MOESM9_ESM.zip › Source data Fig 7/Fig 7/7B/MCAO-GSK-872-WT-20.tif]

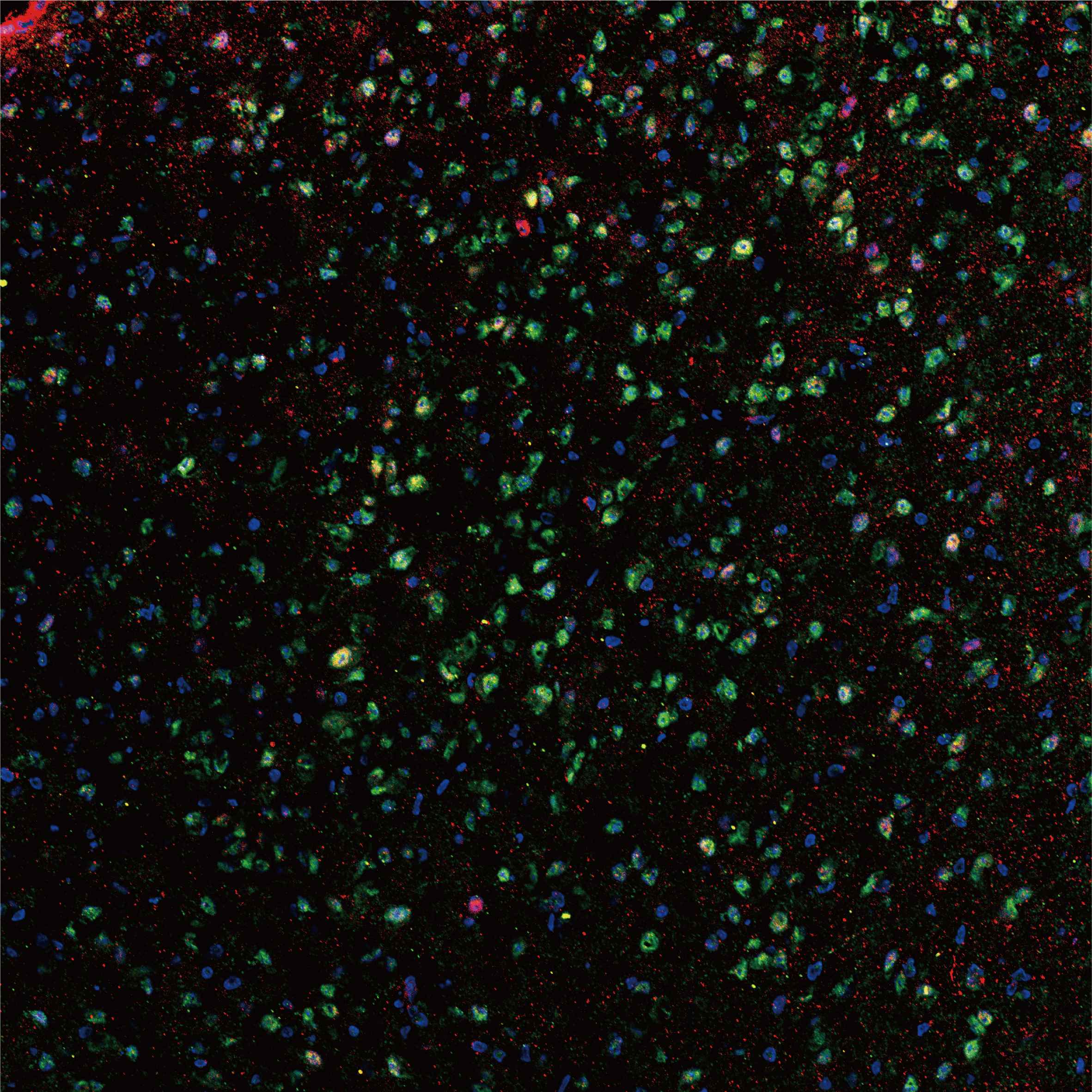

Supplement: Supplementary file 9 — Source data Fig. 7 [file 44321_2025_206_MOESM9_ESM.zip › Source data Fig 7/Fig 7/7E/KO-MCAO-CON MERGE.tif]

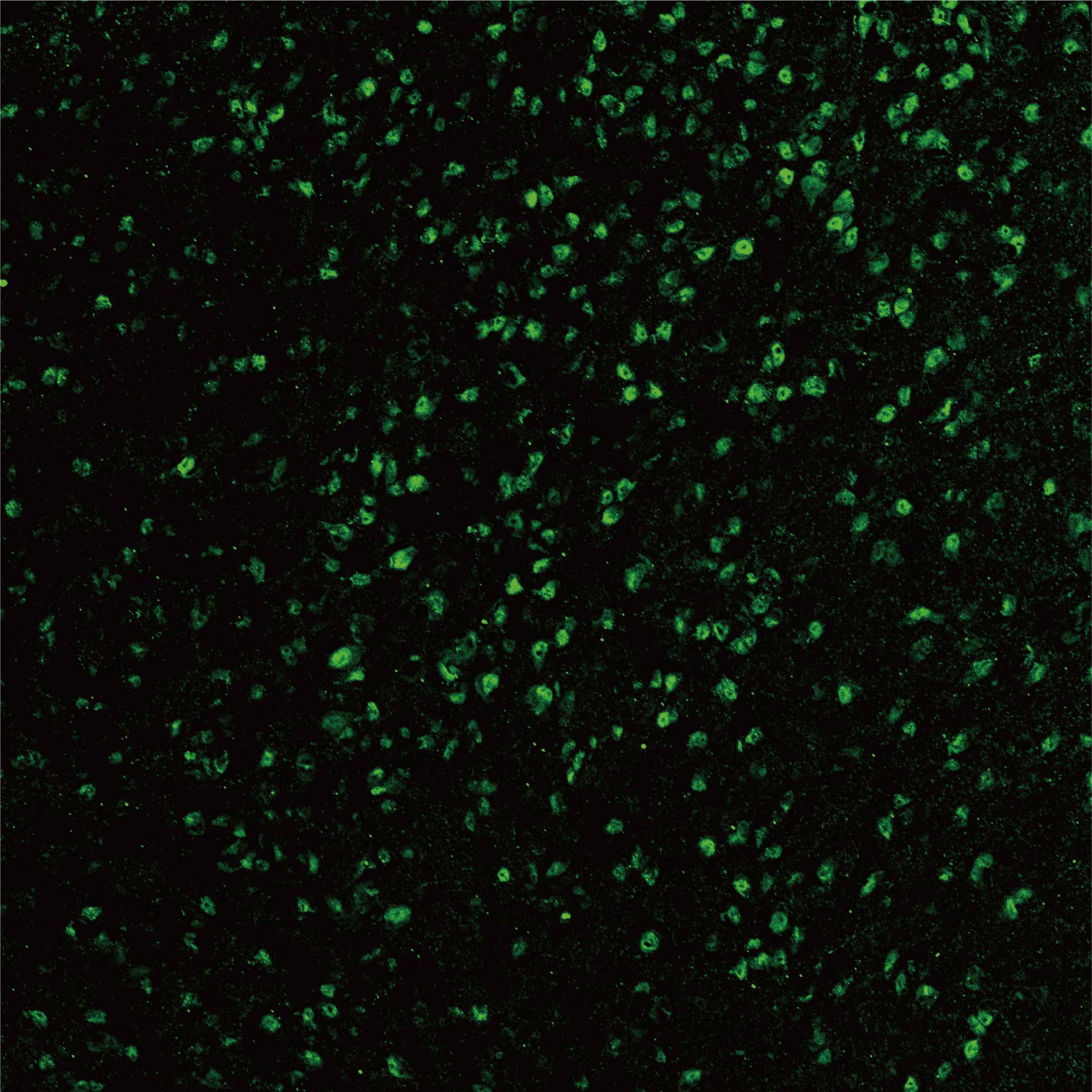

Supplement: Supplementary file 9 — Source data Fig. 7 [file 44321_2025_206_MOESM9_ESM.zip › Source data Fig 7/Fig 7/7E/KO-MCAO-CON NEUN.tif]

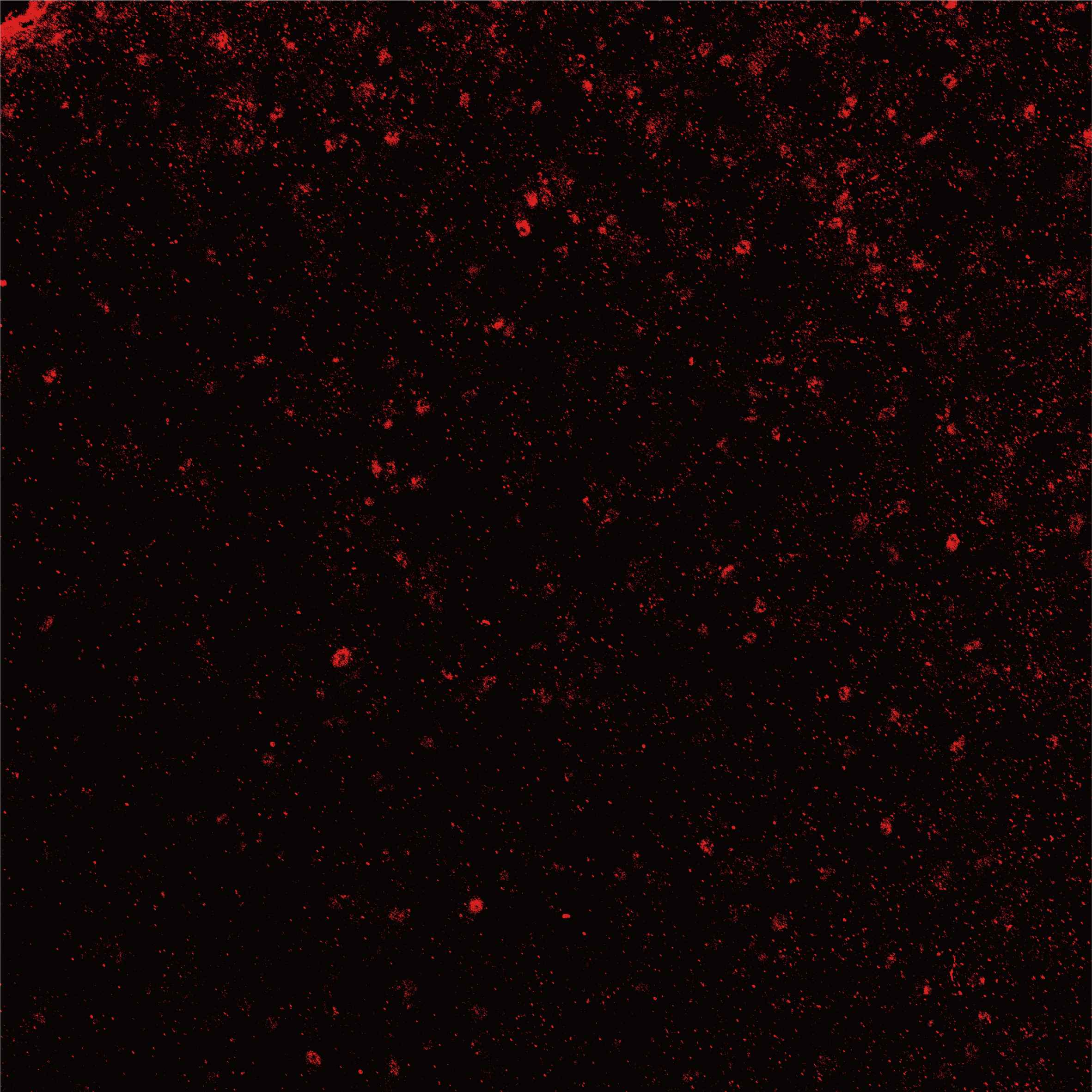

Supplement: Supplementary file 9 — Source data Fig. 7 [file 44321_2025_206_MOESM9_ESM.zip › Source data Fig 7/Fig 7/7E/KO-MCAO-CON P-MLKL.tif]

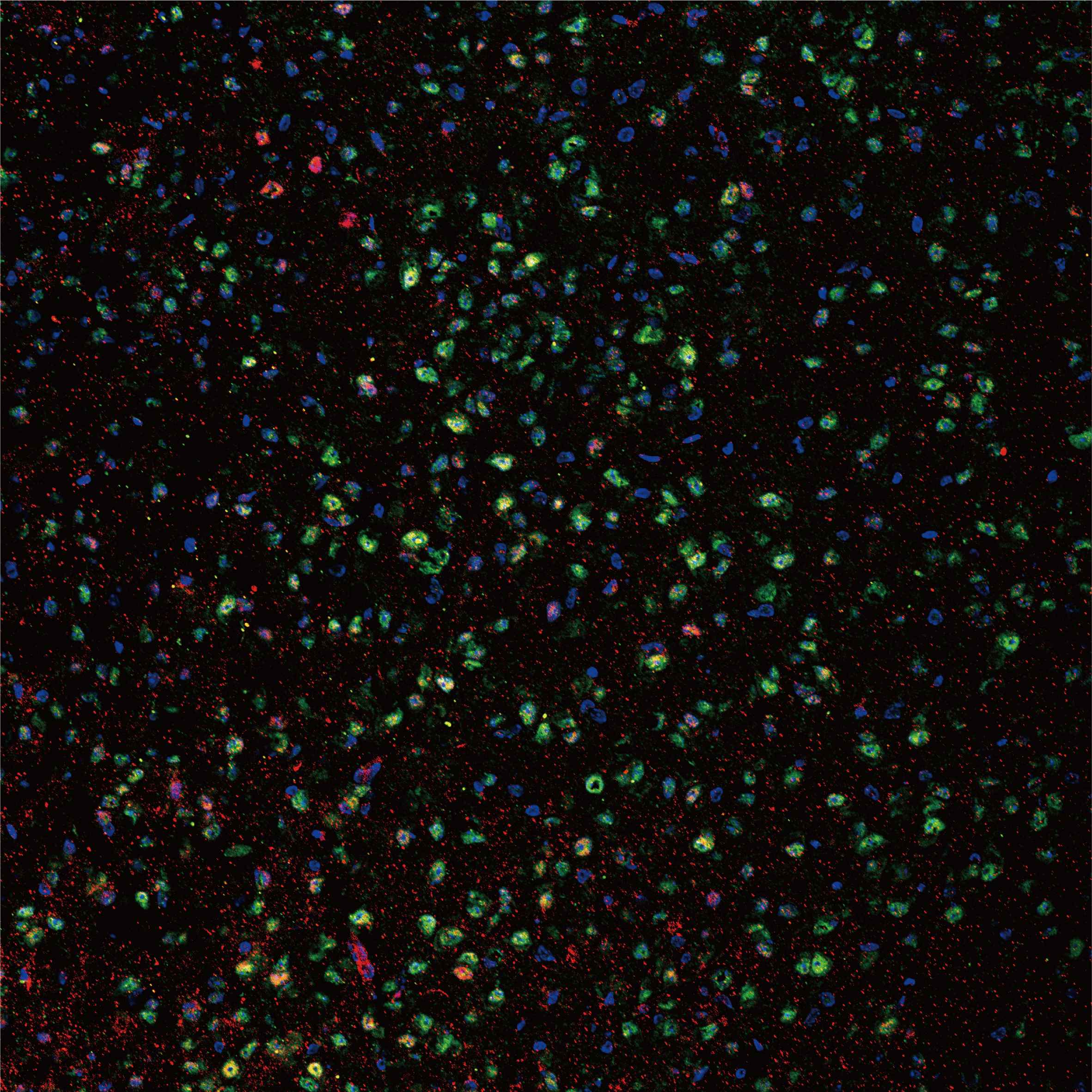

Supplement: Supplementary file 9 — Source data Fig. 7 [file 44321_2025_206_MOESM9_ESM.zip › Source data Fig 7/Fig 7/7E/KO-MCAO-GSK-872 MERGE.tif]

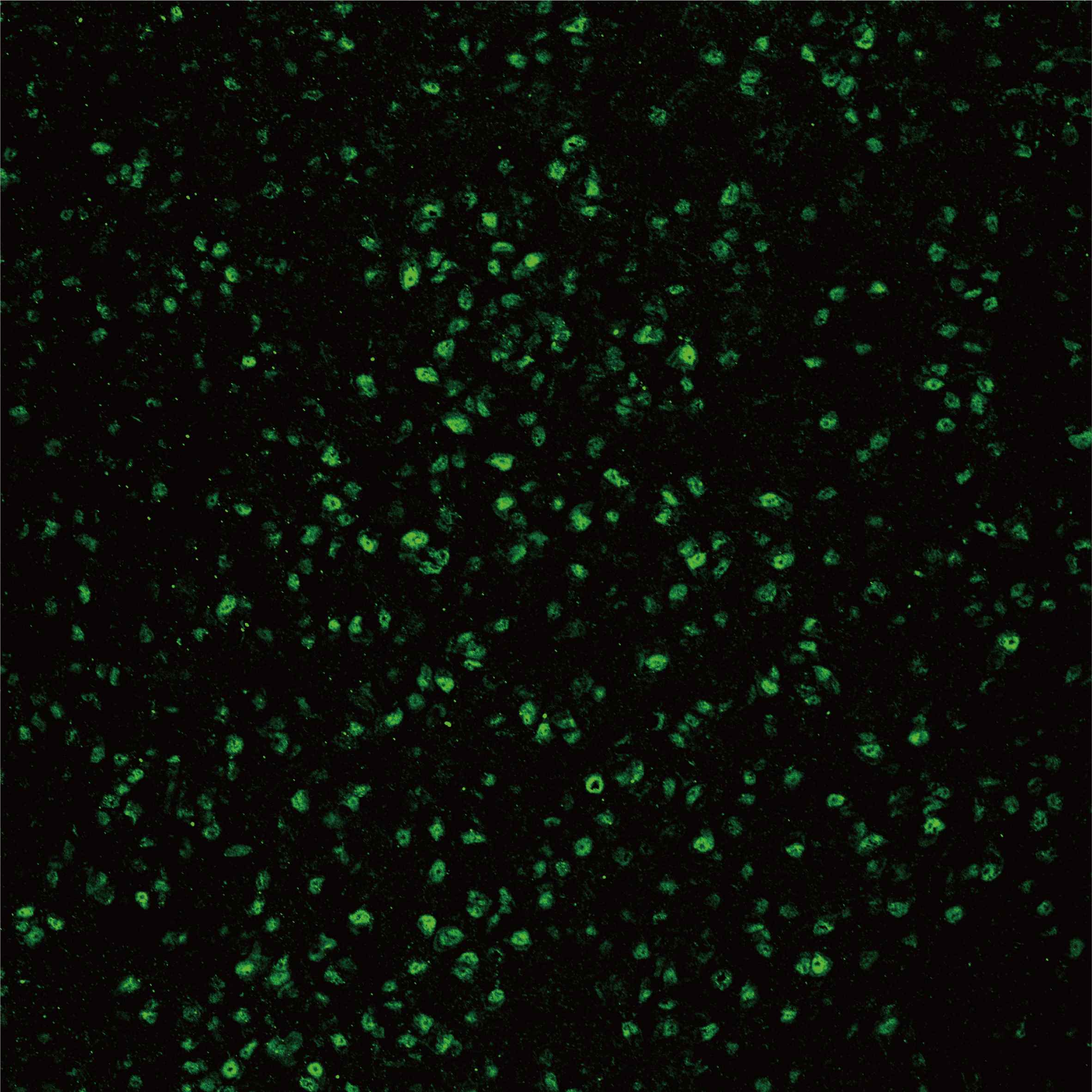

Supplement: Supplementary file 9 — Source data Fig. 7 [file 44321_2025_206_MOESM9_ESM.zip › Source data Fig 7/Fig 7/7E/KO-MCAO-GSK-872 NEUN.tif]

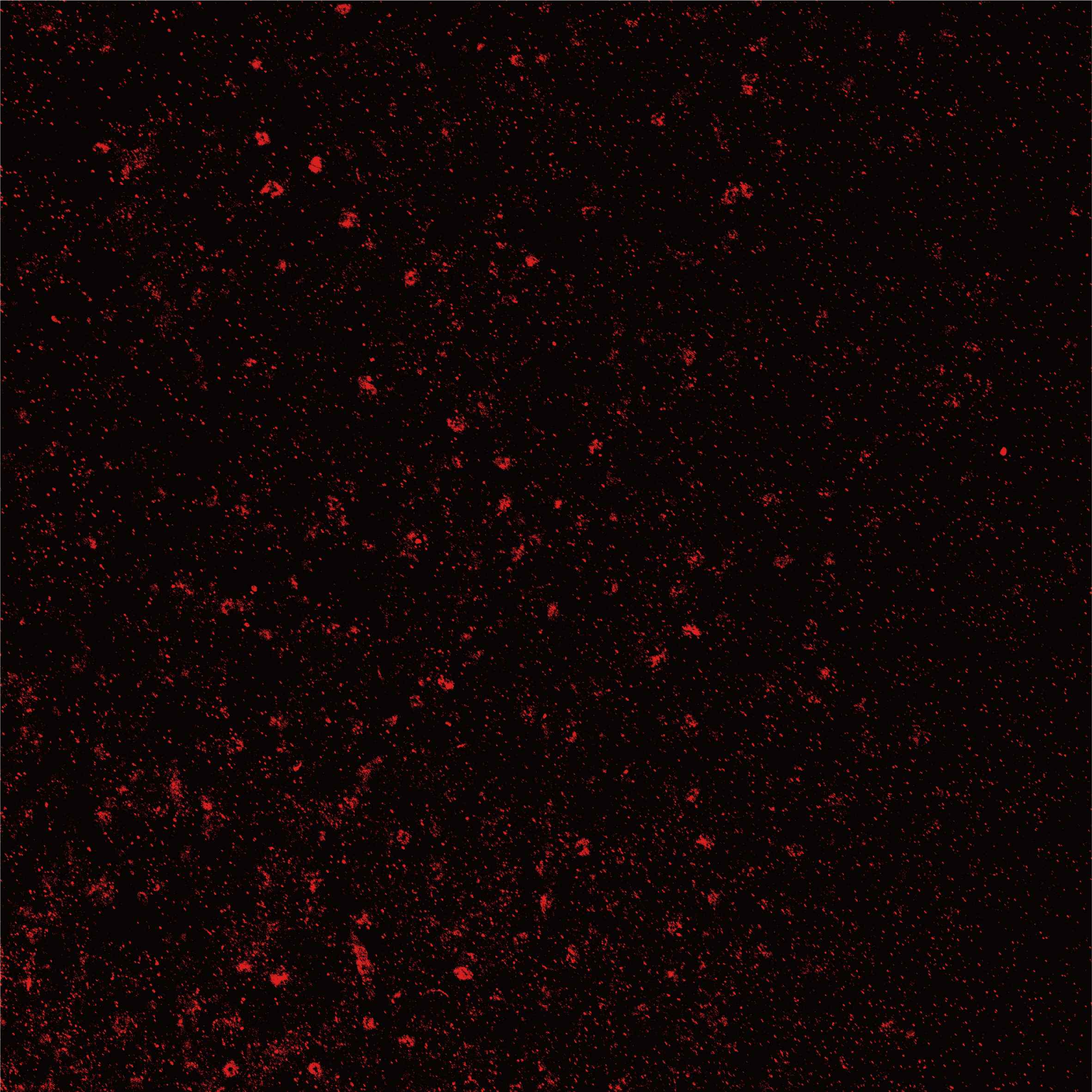

Supplement: Supplementary file 9 — Source data Fig. 7 [file 44321_2025_206_MOESM9_ESM.zip › Source data Fig 7/Fig 7/7E/KO-MCAO-GSK-872 P-MLKL.tif]

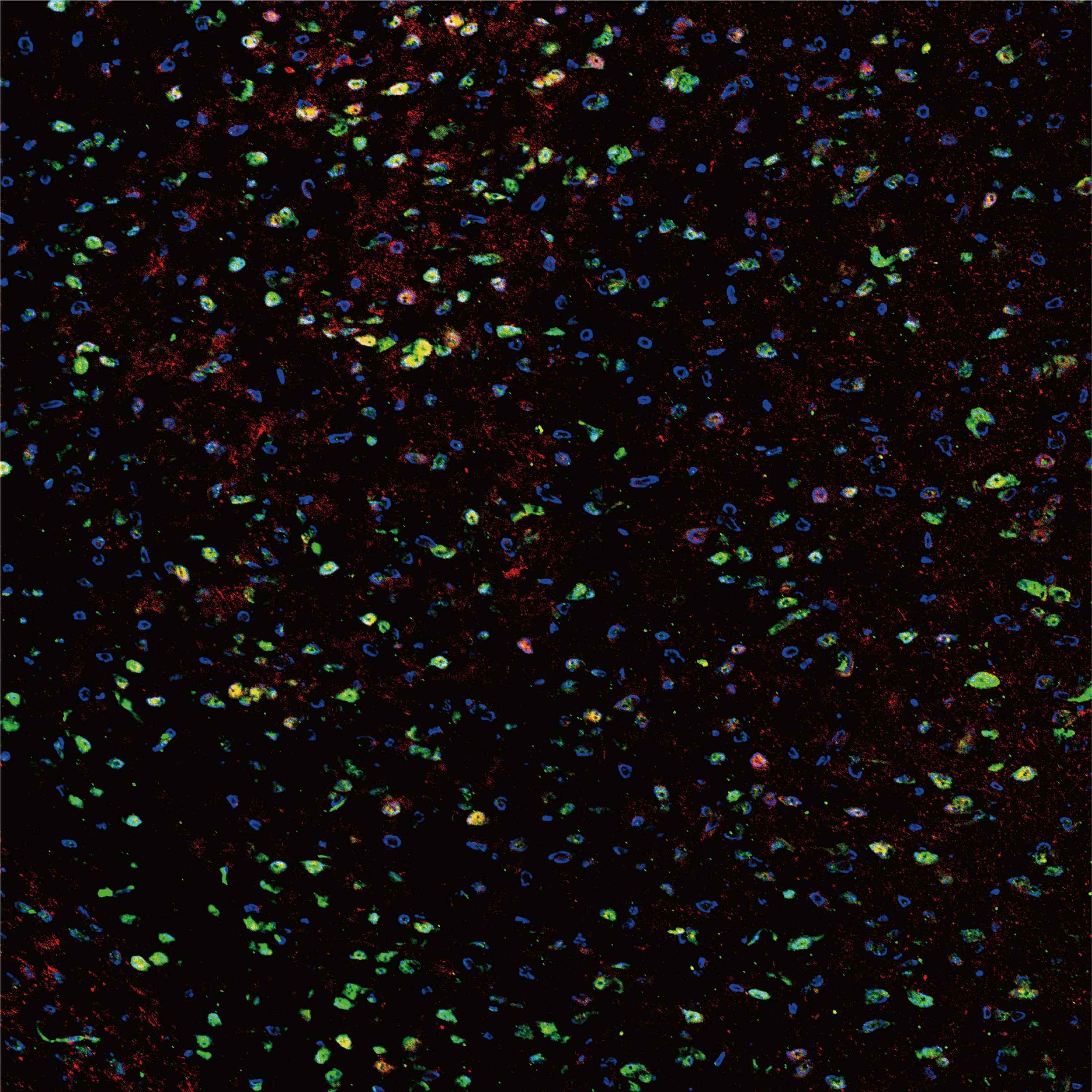

Supplement: Supplementary file 9 — Source data Fig. 7 [file 44321_2025_206_MOESM9_ESM.zip › Source data Fig 7/Fig 7/7E/WT-MCAO-CON MERGE.tif]

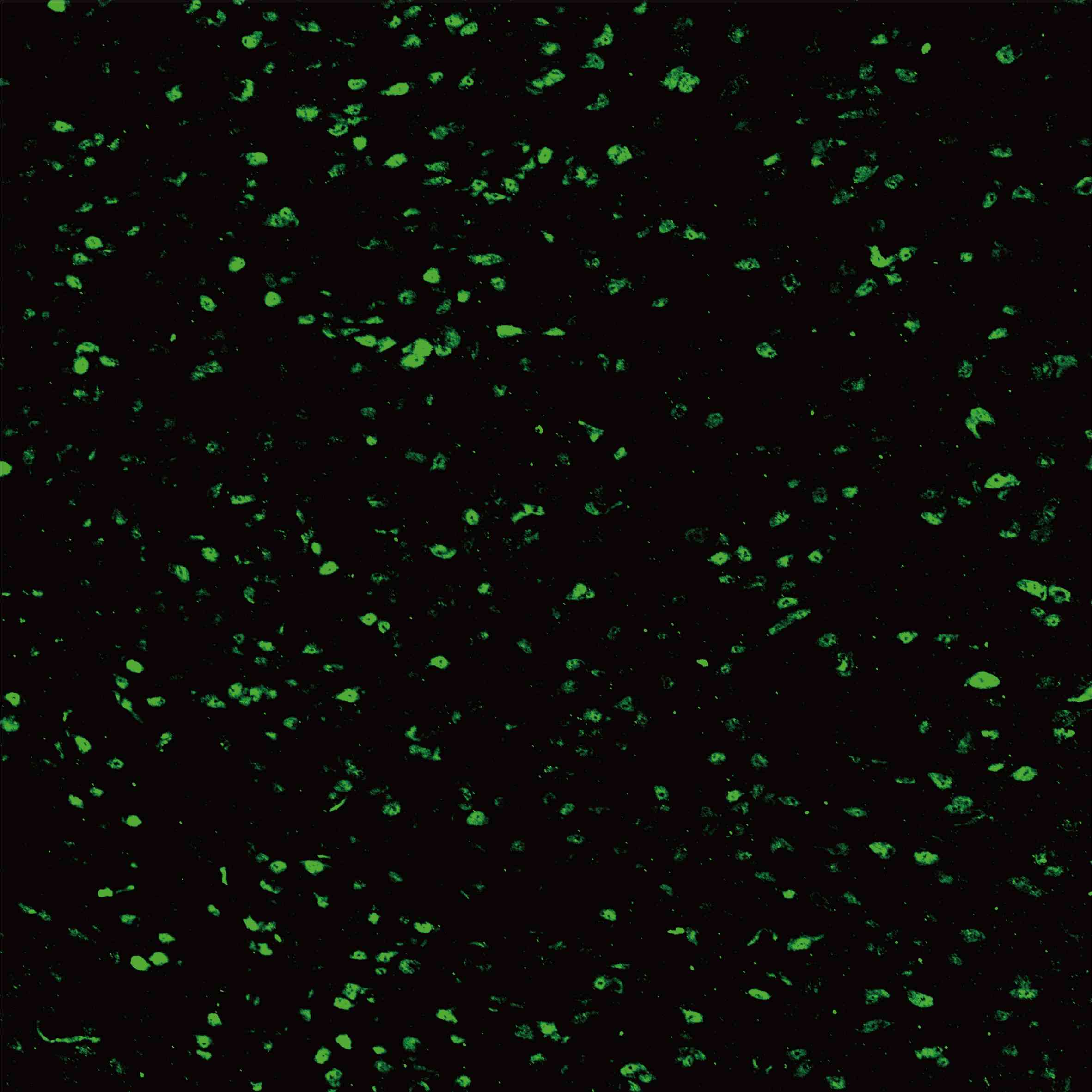

Supplement: Supplementary file 9 — Source data Fig. 7 [file 44321_2025_206_MOESM9_ESM.zip › Source data Fig 7/Fig 7/7E/WT-MCAO-CON NEUN.tif]

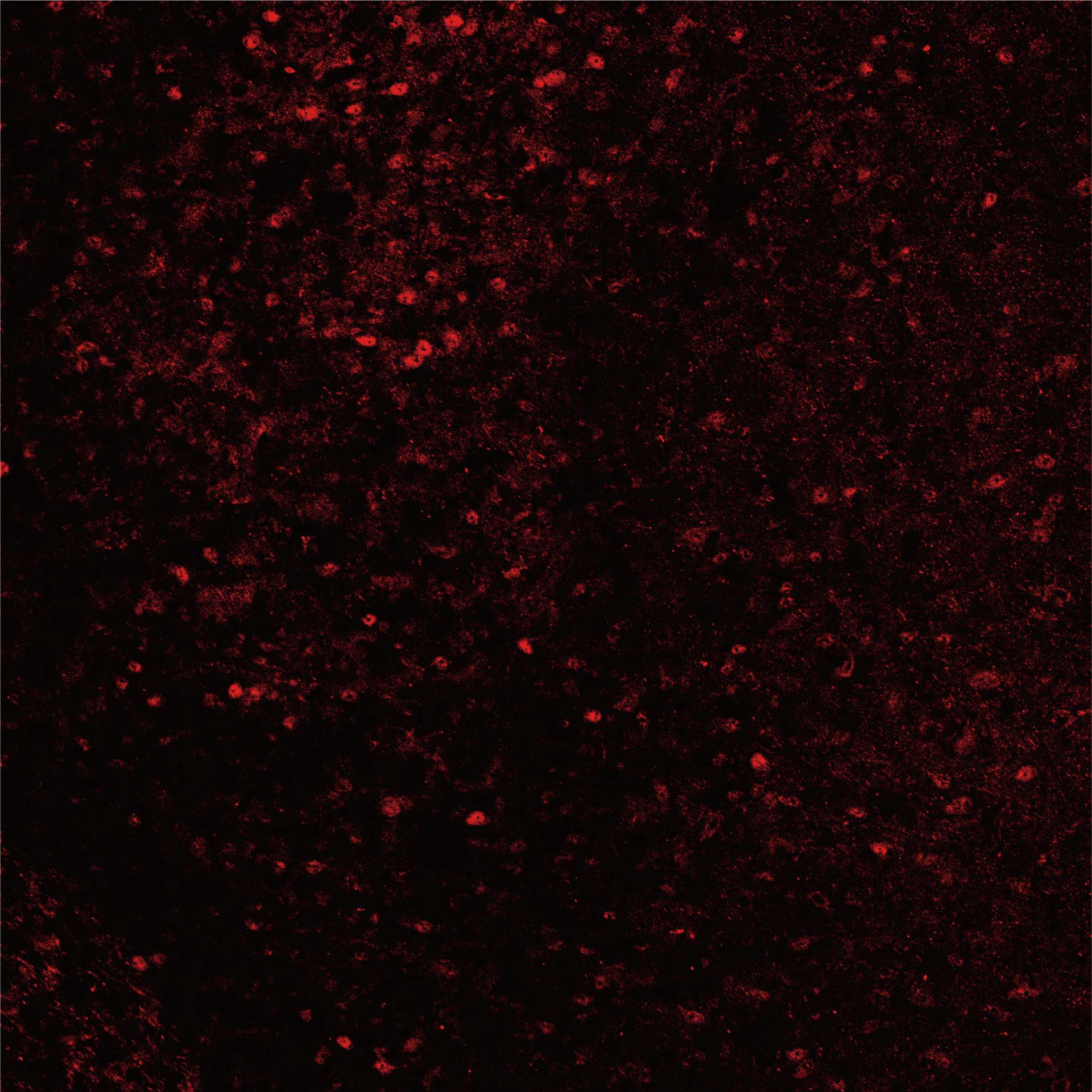

Supplement: Supplementary file 9 — Source data Fig. 7 [file 44321_2025_206_MOESM9_ESM.zip › Source data Fig 7/Fig 7/7E/WT-MCAO-CON P-MLKL.tif]

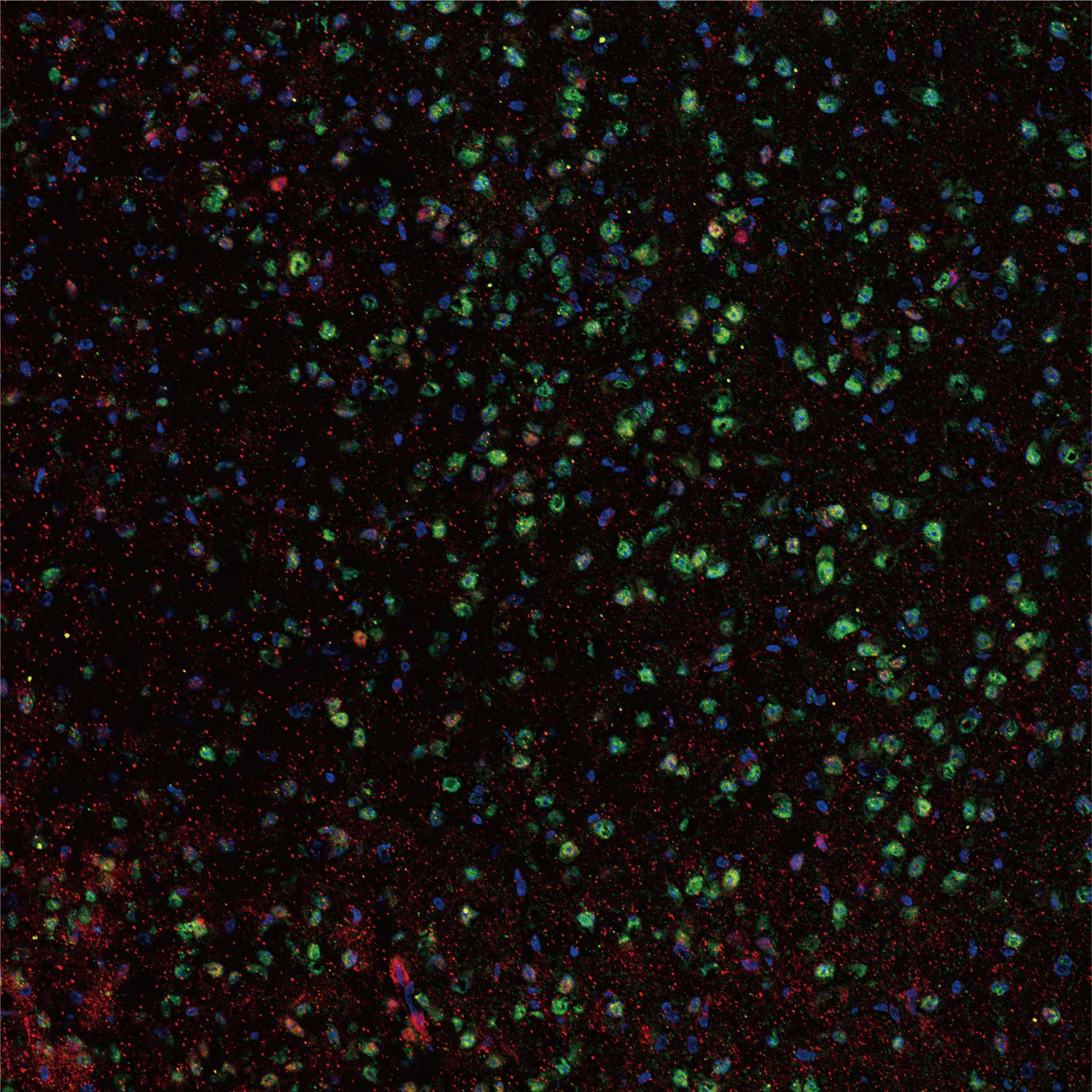

Supplement: Supplementary file 9 — Source data Fig. 7 [file 44321_2025_206_MOESM9_ESM.zip › Source data Fig 7/Fig 7/7E/WT-MCAO-GSK-872 MERGE.tif]

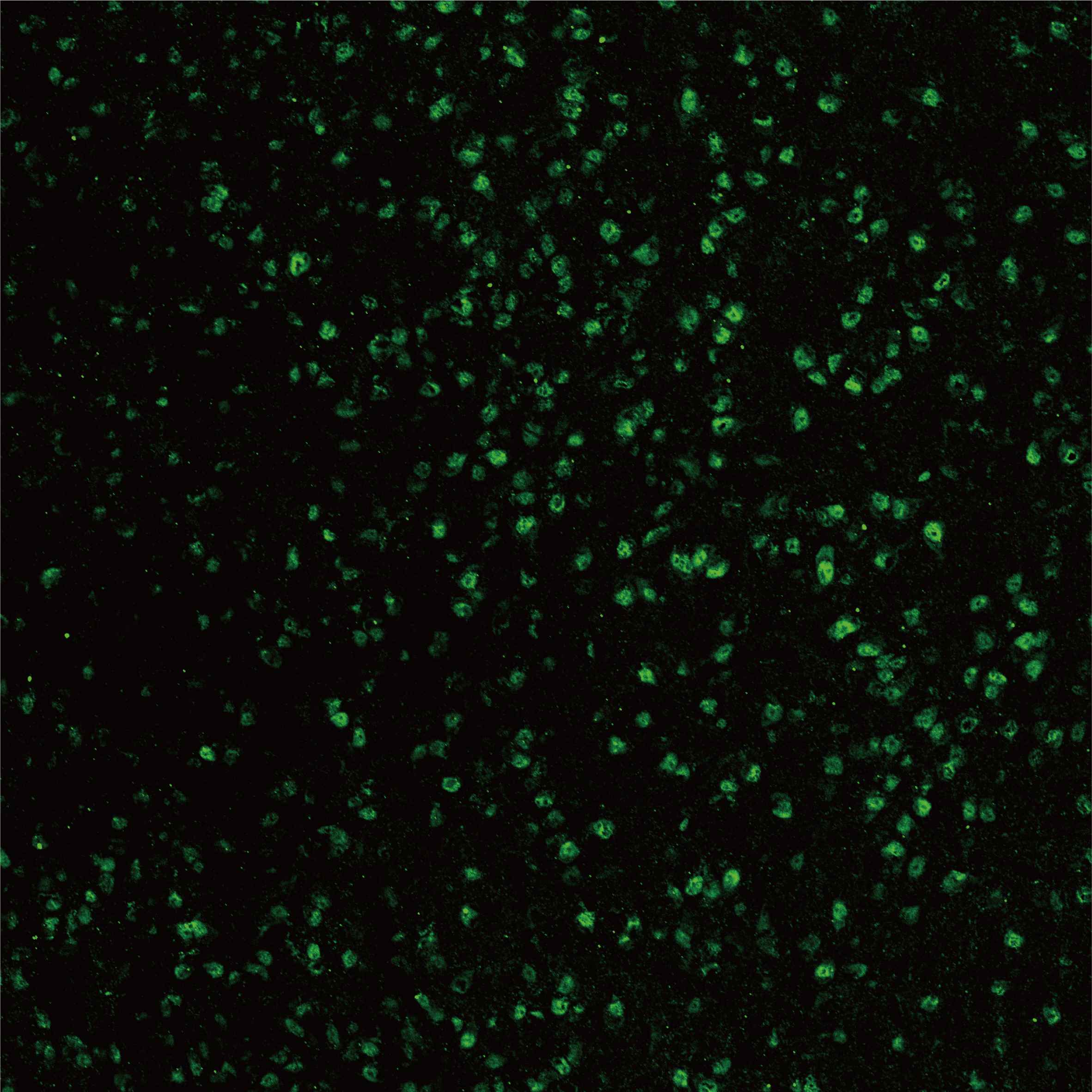

Supplement: Supplementary file 9 — Source data Fig. 7 [file 44321_2025_206_MOESM9_ESM.zip › Source data Fig 7/Fig 7/7E/WT-MCAO-GSK-872 NEUN.tif]

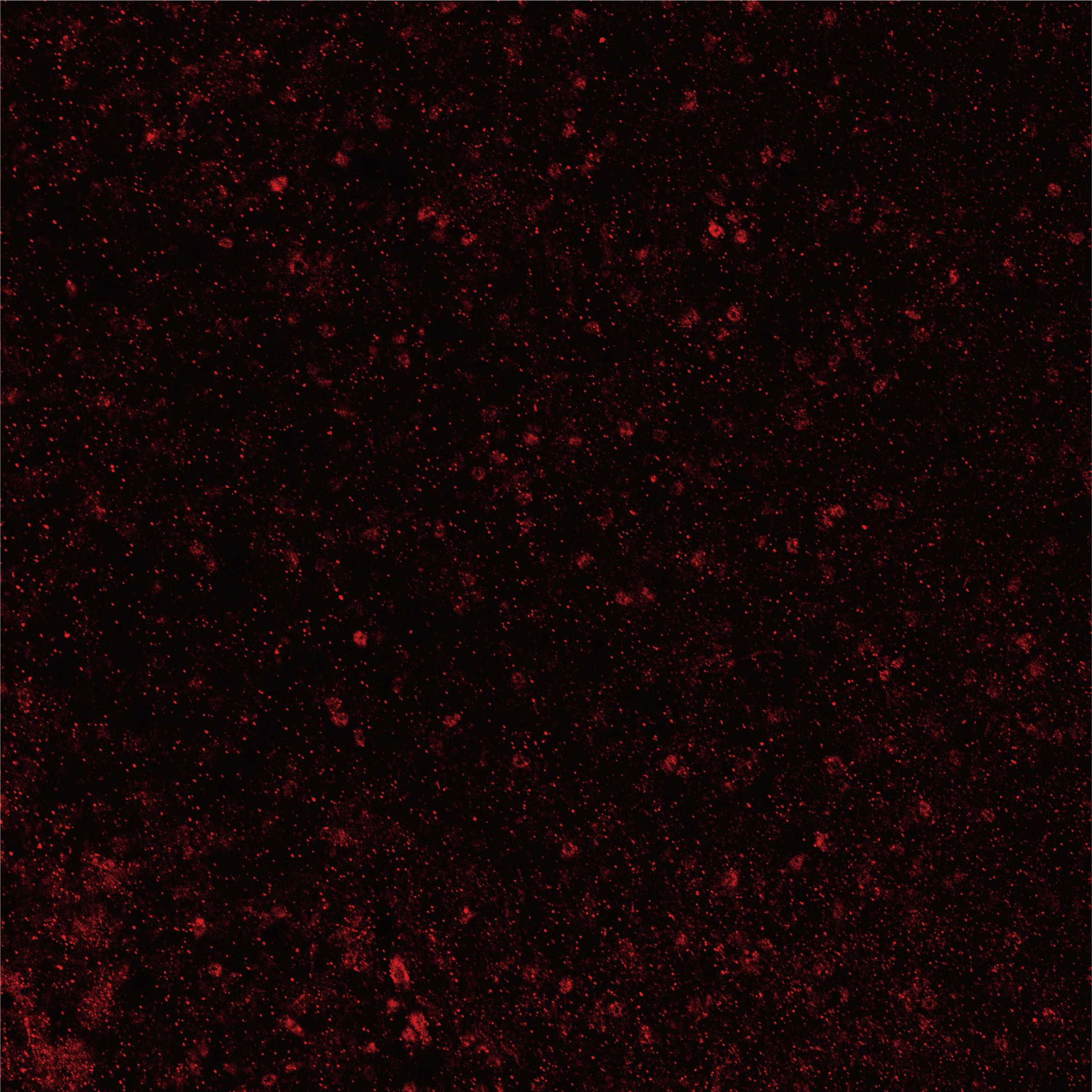

Supplement: Supplementary file 9 — Source data Fig. 7 [file 44321_2025_206_MOESM9_ESM.zip › Source data Fig 7/Fig 7/7E/WT-MCAO-GSK-872 P-MLKL.tif]

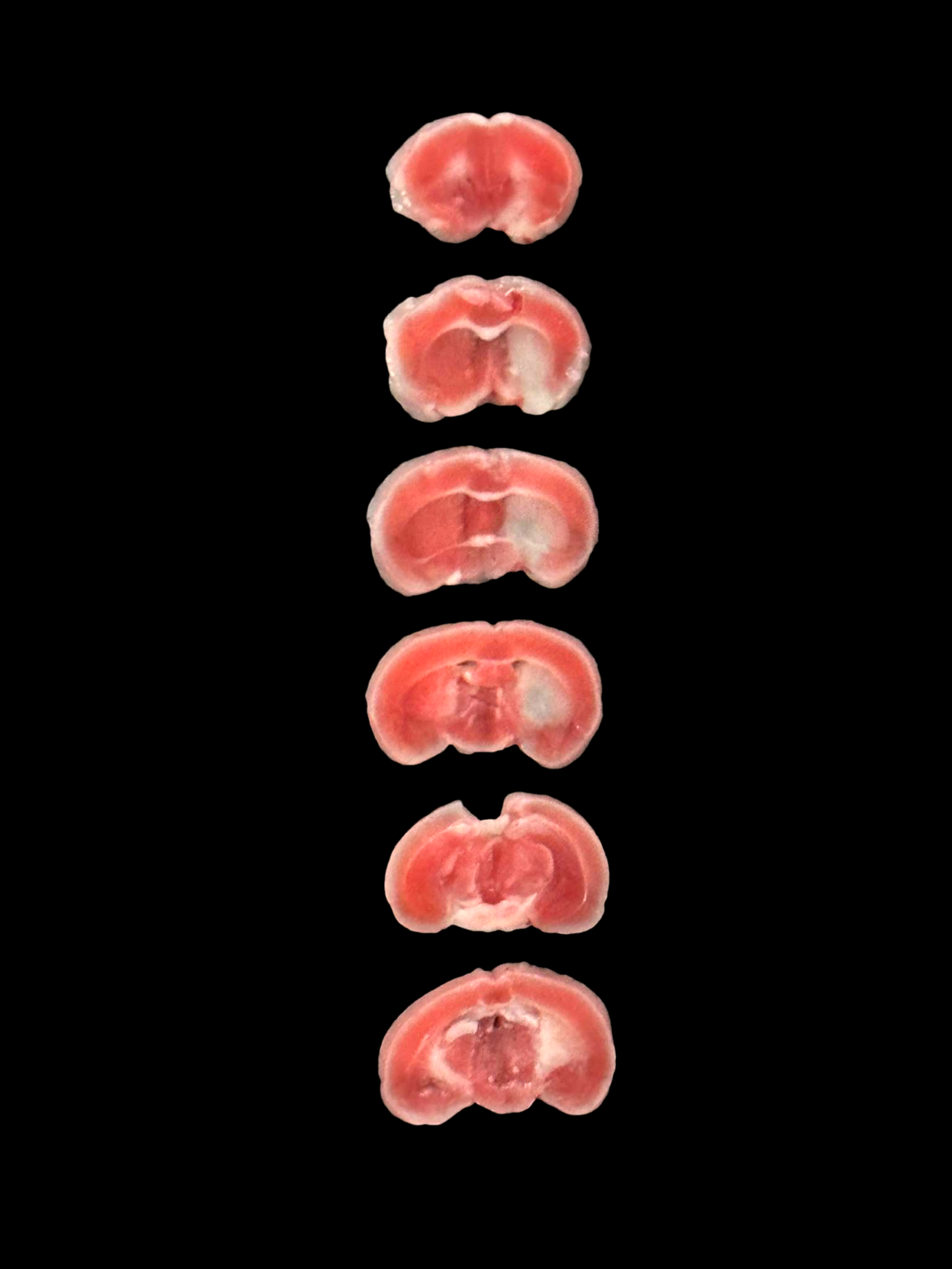

Supplement: Supplementary file 9 — Source data Fig. 7 [file 44321_2025_206_MOESM9_ESM.zip › Source data Fig 7/Fig 7/7G/MCAO-CON-KO.tif]

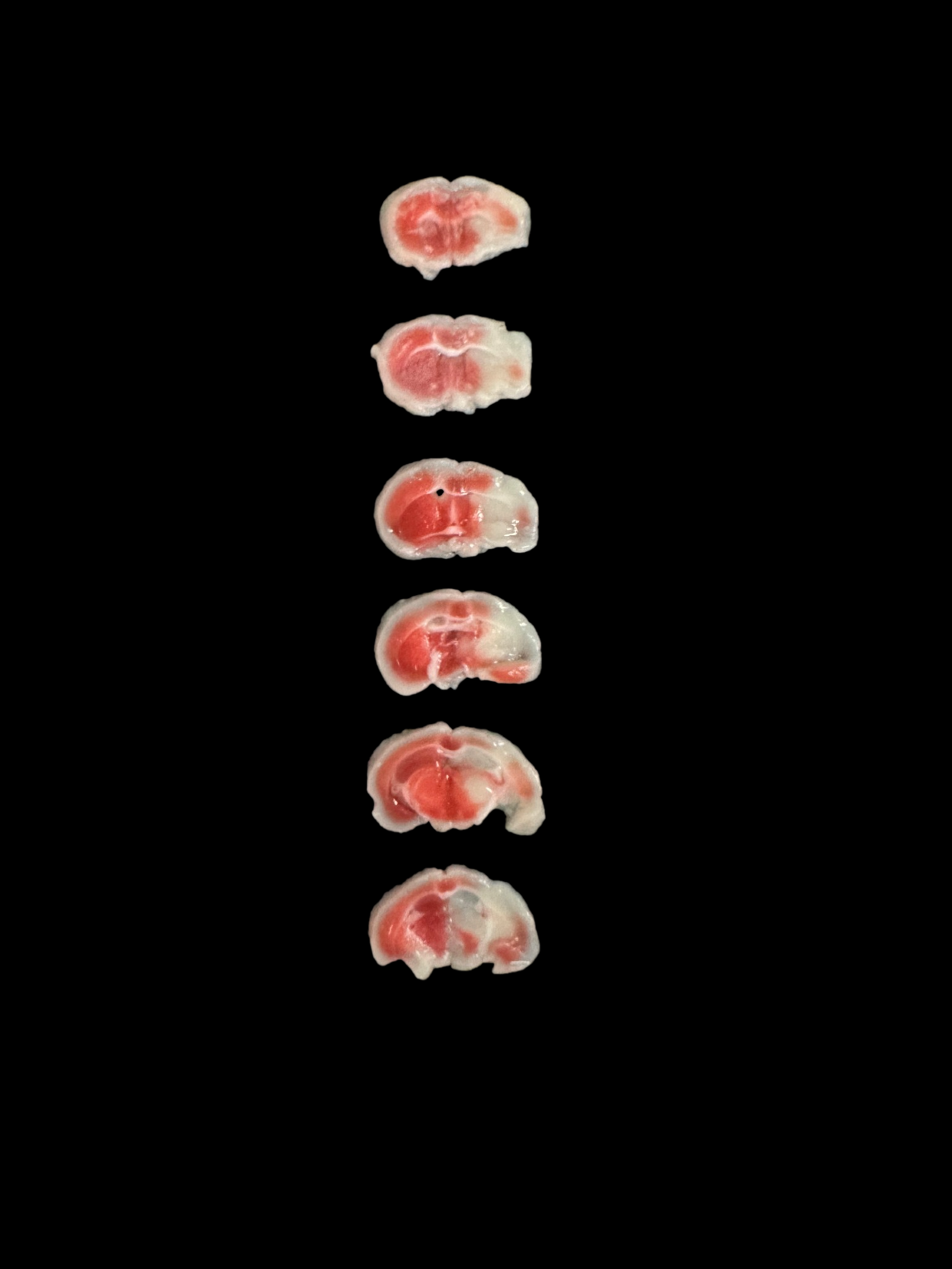

Supplement: Supplementary file 9 — Source data Fig. 7 [file 44321_2025_206_MOESM9_ESM.zip › Source data Fig 7/Fig 7/7G/MCAO-CON-WT.tif]

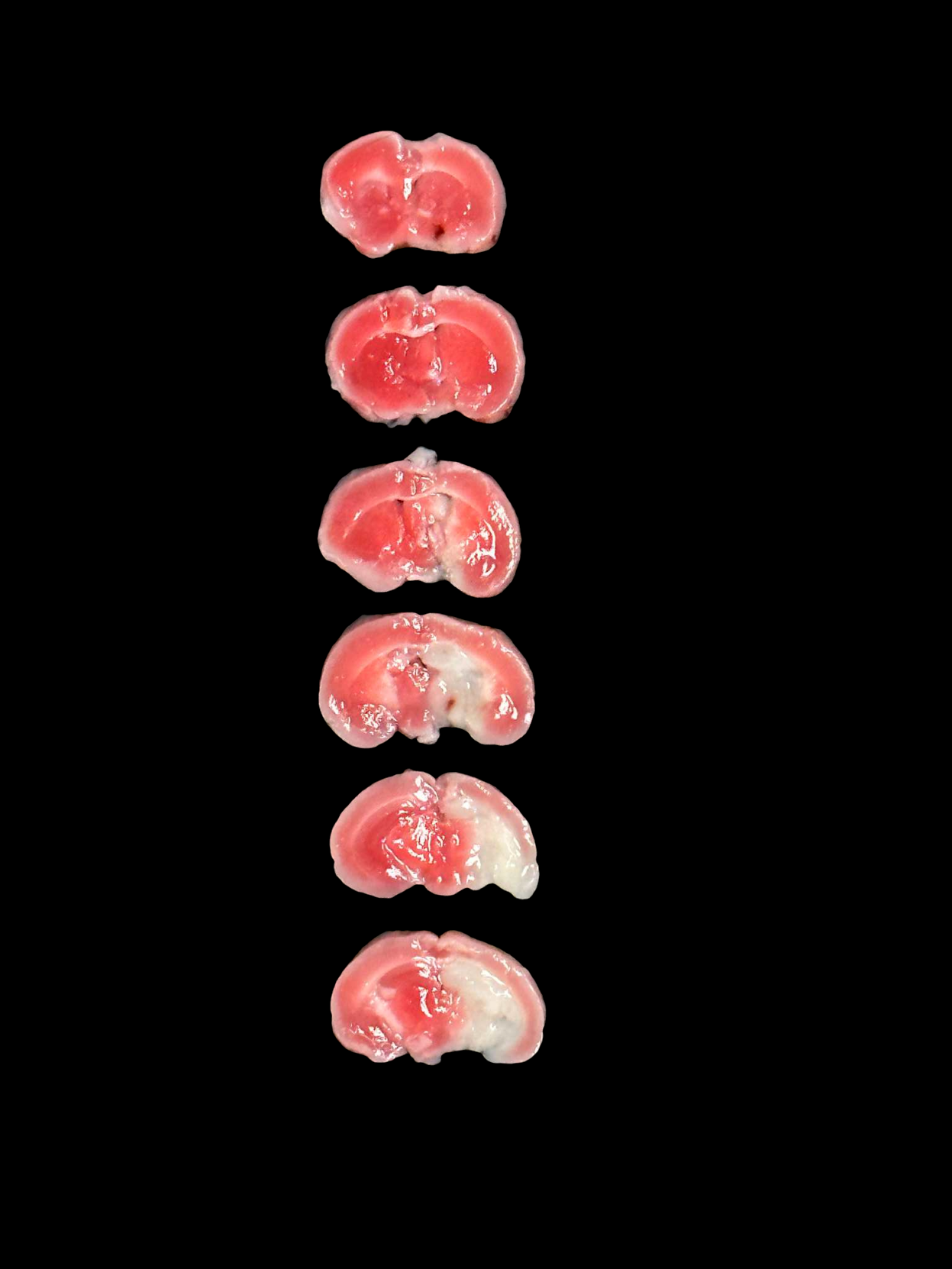

Supplement: Supplementary file 9 — Source data Fig. 7 [file 44321_2025_206_MOESM9_ESM.zip › Source data Fig 7/Fig 7/7G/MCAO-LN5P45-10.tif]

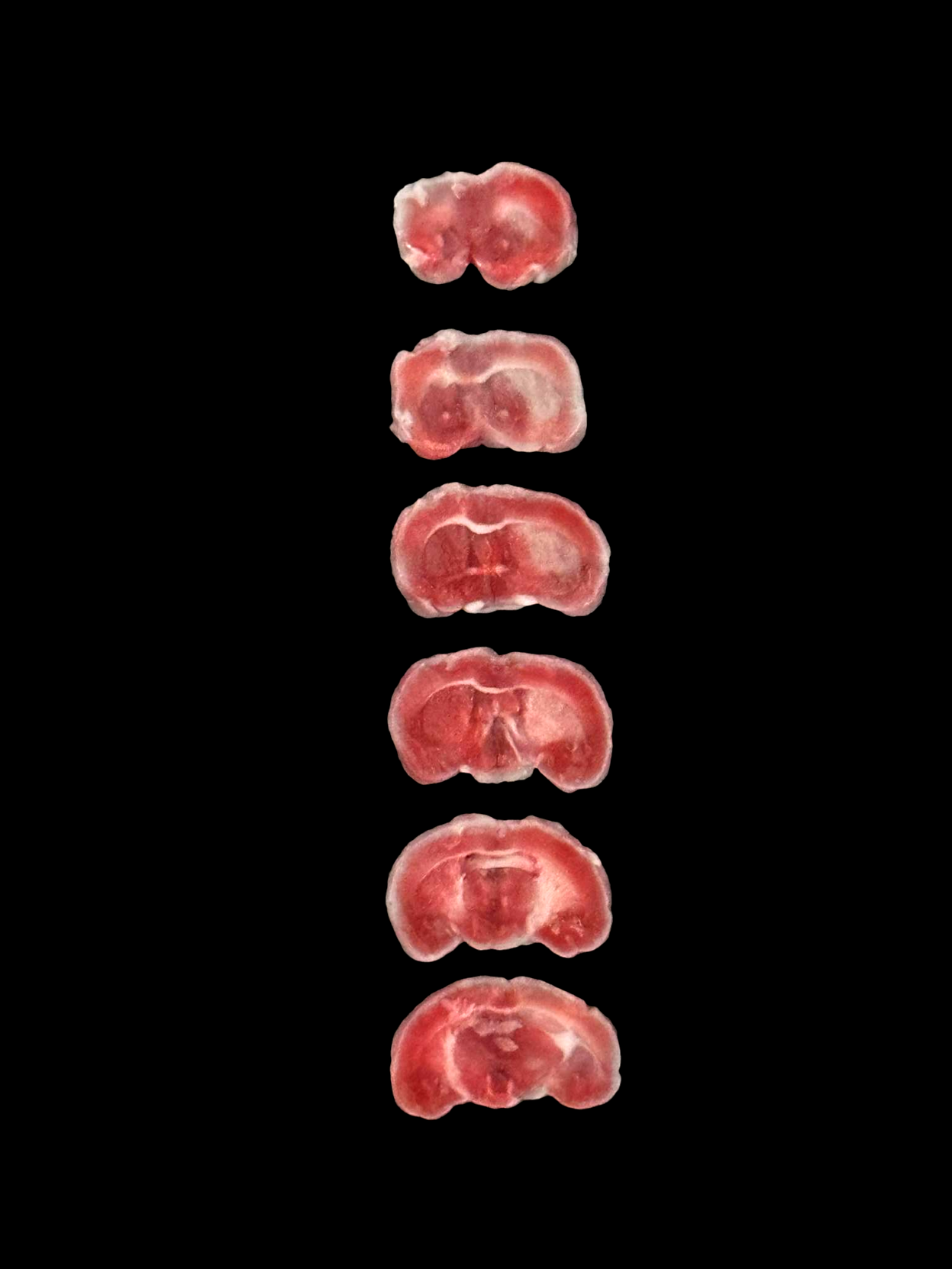

Supplement: Supplementary file 9 — Source data Fig. 7 [file 44321_2025_206_MOESM9_ESM.zip › Source data Fig 7/Fig 7/7G/MCAO-LN5P45-20.tif]

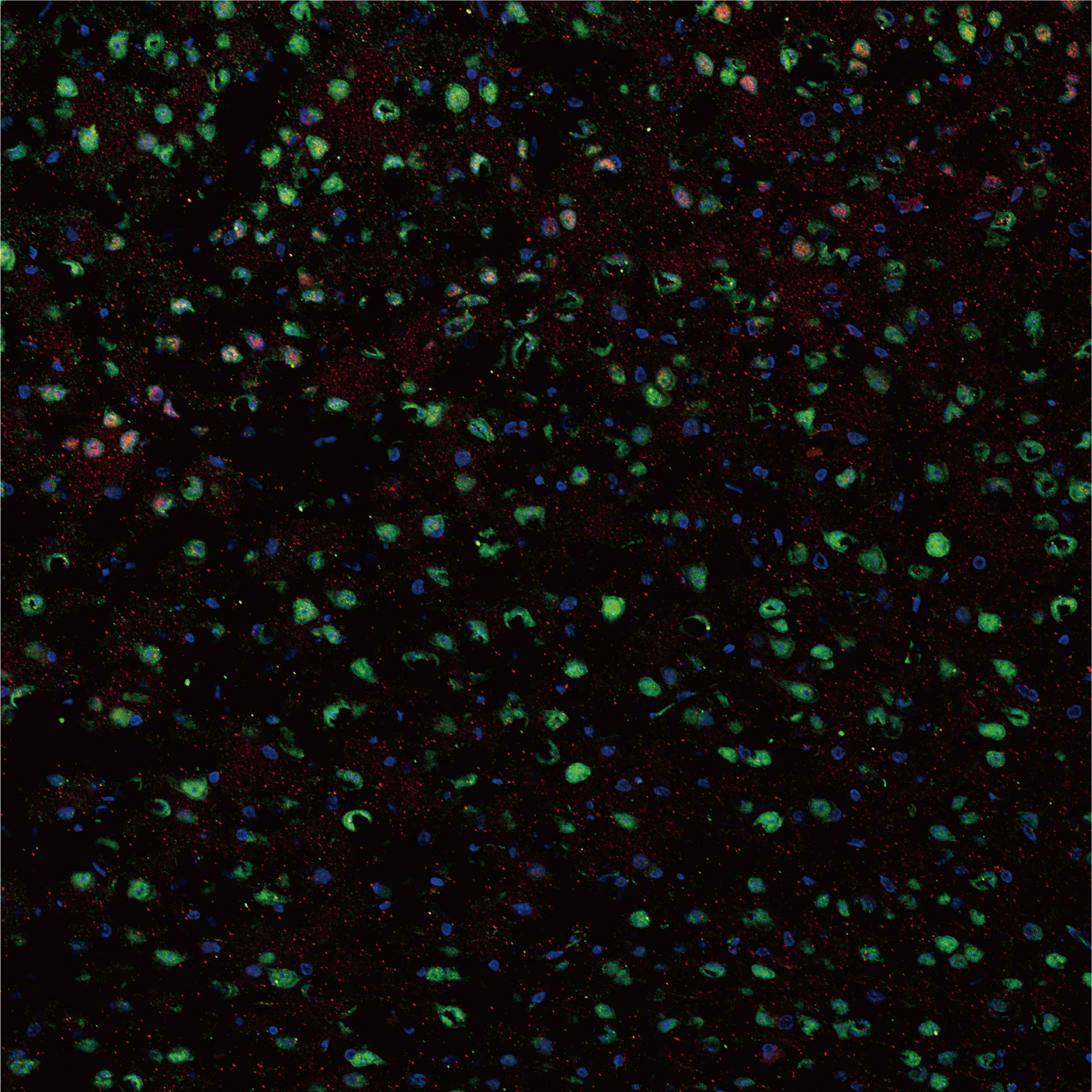

Supplement: Supplementary file 9 — Source data Fig. 7 [file 44321_2025_206_MOESM9_ESM.zip › Source data Fig 7/Fig 7/7J/KO-MCAO-CON-MERGE.tif]

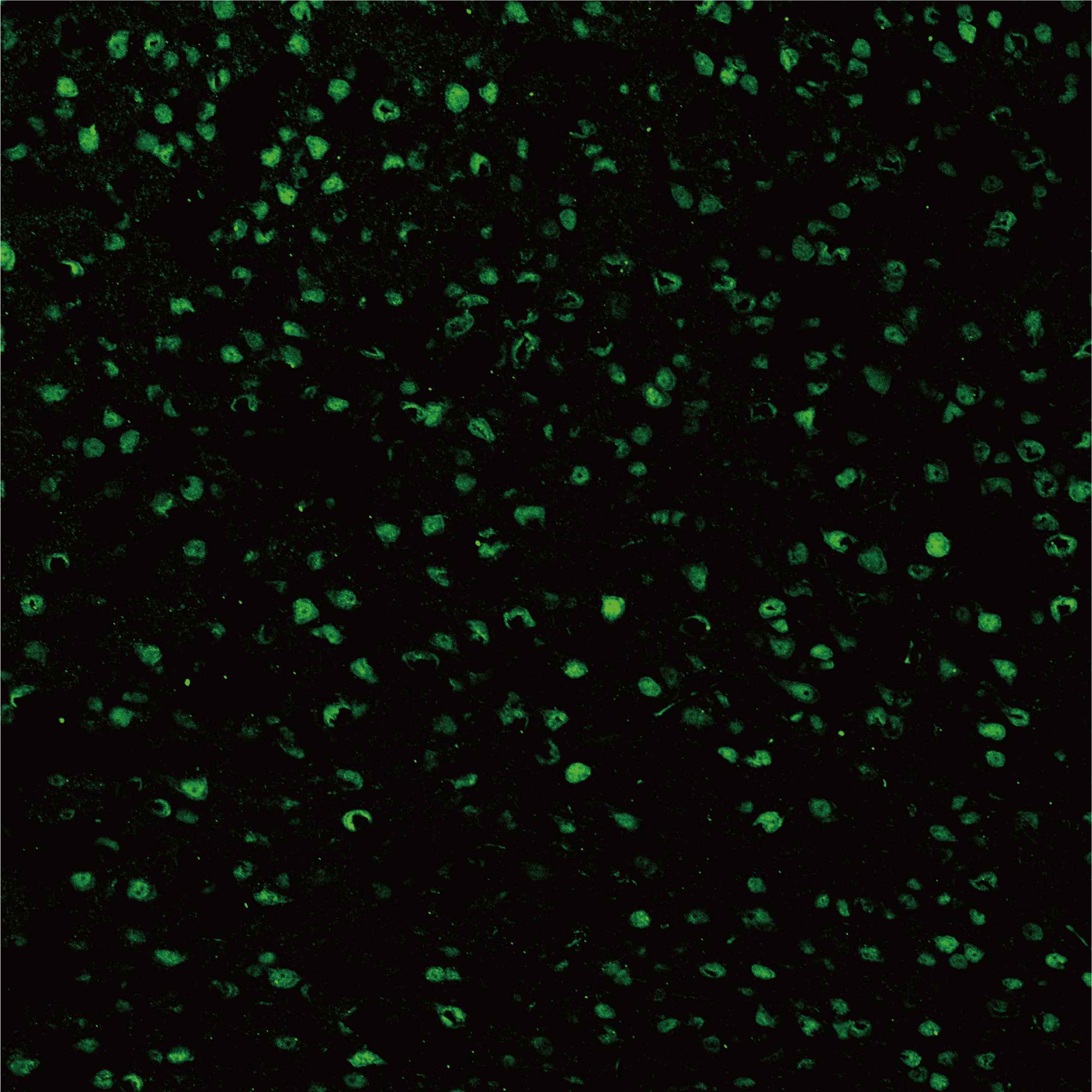

Supplement: Supplementary file 9 — Source data Fig. 7 [file 44321_2025_206_MOESM9_ESM.zip › Source data Fig 7/Fig 7/7J/KO-MCAO-CON-NEUN.tif]

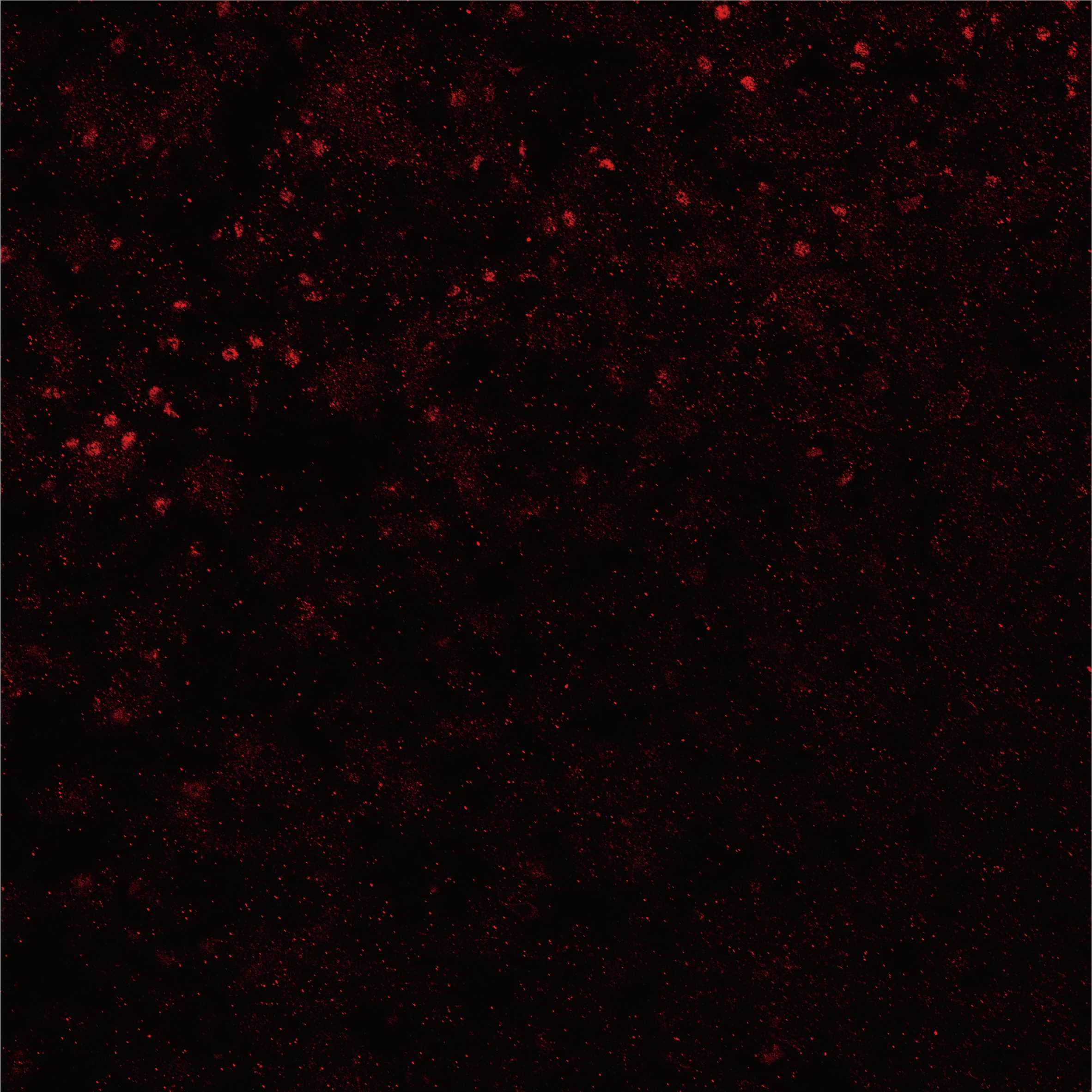

Supplement: Supplementary file 9 — Source data Fig. 7 [file 44321_2025_206_MOESM9_ESM.zip › Source data Fig 7/Fig 7/7J/KO-MCAO-CON-P-MLKL.tif]

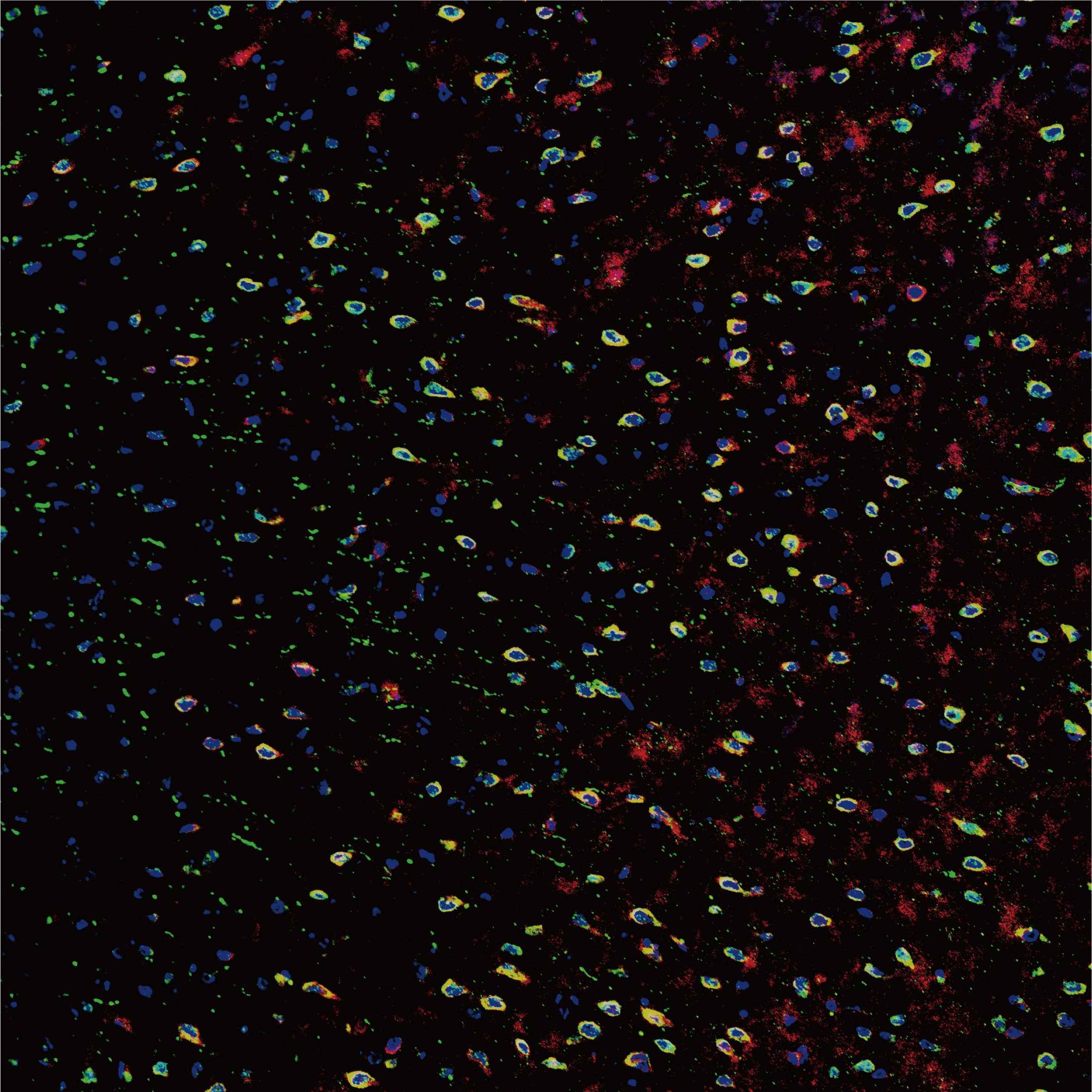

Supplement: Supplementary file 9 — Source data Fig. 7 [file 44321_2025_206_MOESM9_ESM.zip › Source data Fig 7/Fig 7/7J/WT-MCAO-CON-MERGE.tif]

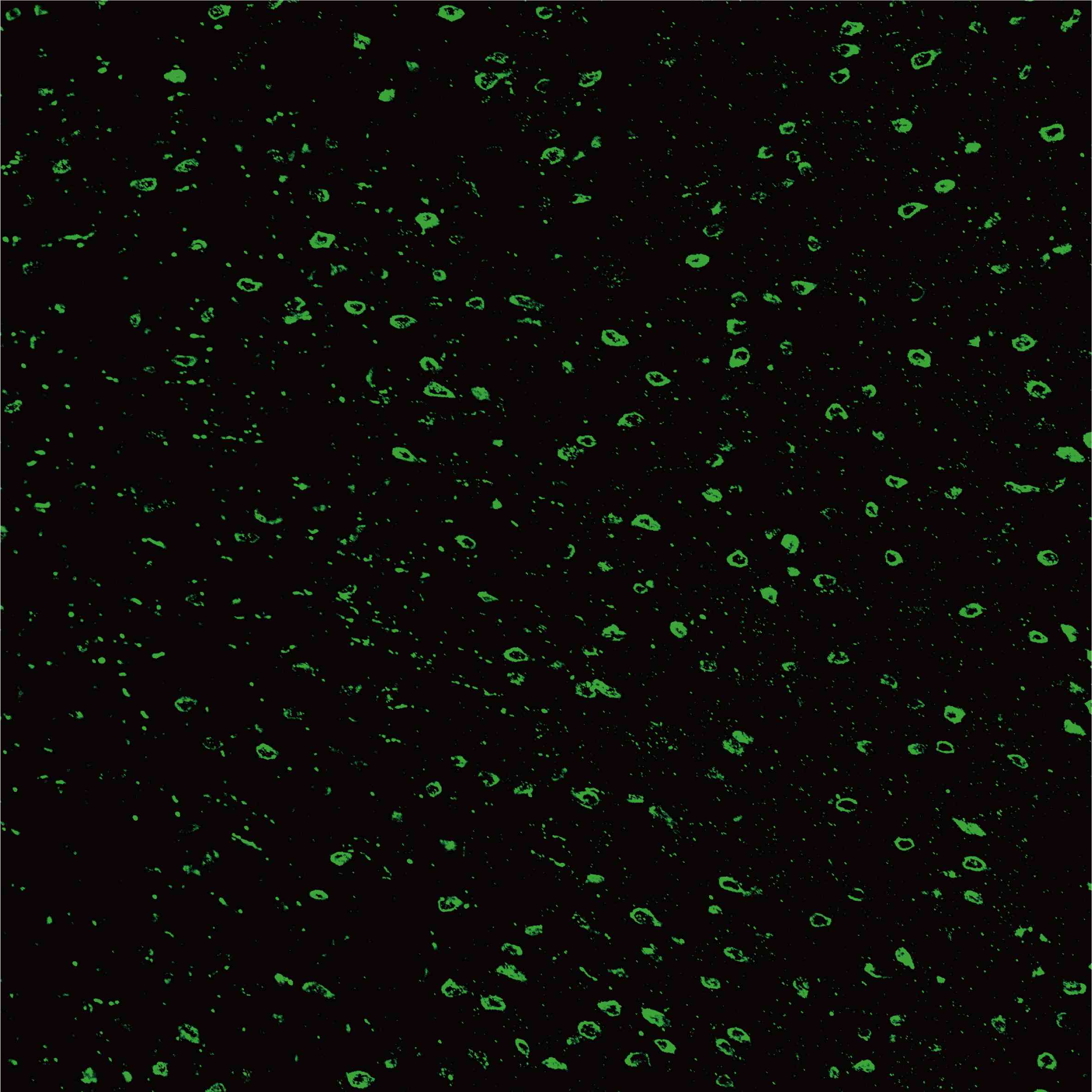

Supplement: Supplementary file 9 — Source data Fig. 7 [file 44321_2025_206_MOESM9_ESM.zip › Source data Fig 7/Fig 7/7J/WT-MCAO-CON-NEUN.tif]

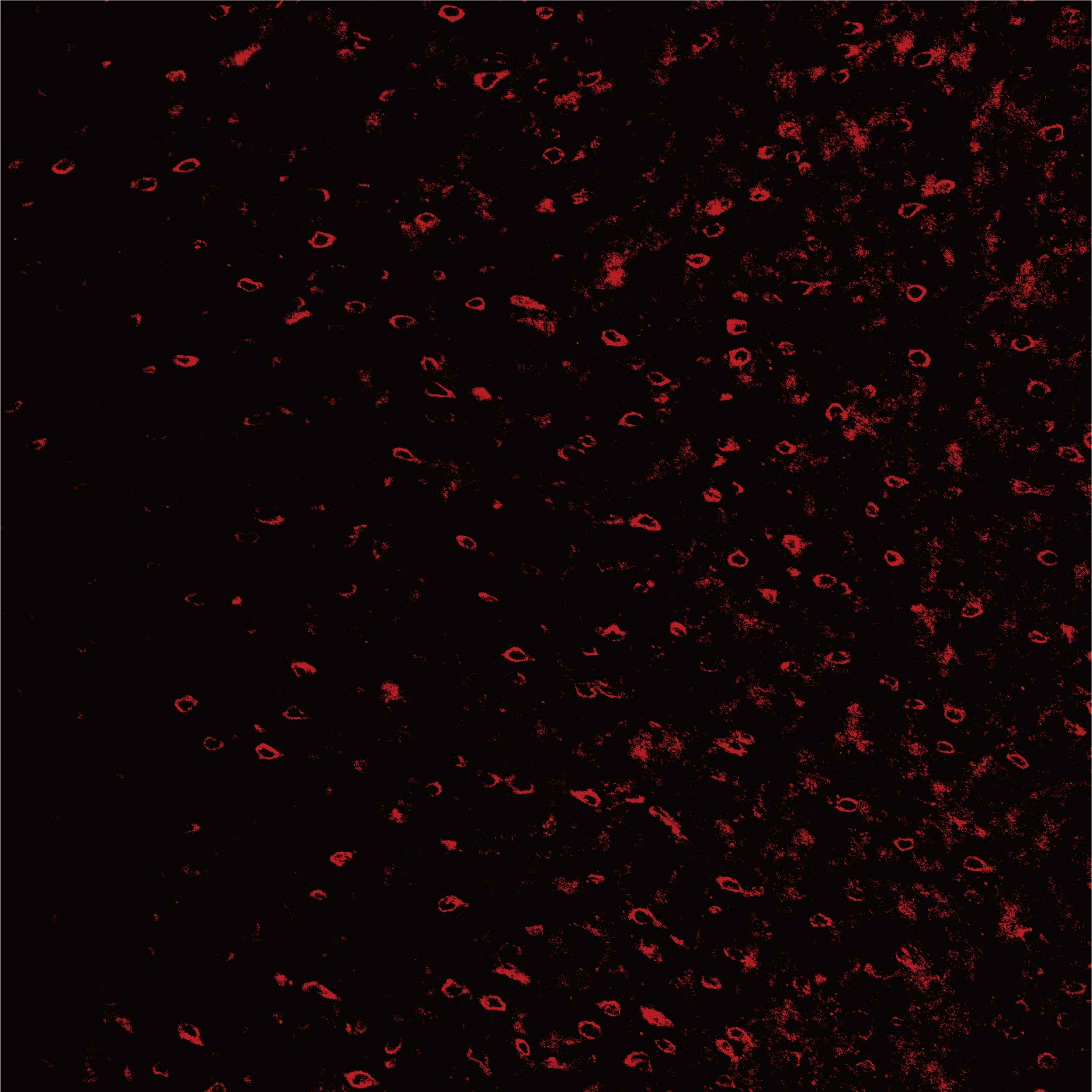

Supplement: Supplementary file 9 — Source data Fig. 7 [file 44321_2025_206_MOESM9_ESM.zip › Source data Fig 7/Fig 7/7J/WT-MCAO-CON-P-MLKL.tif]

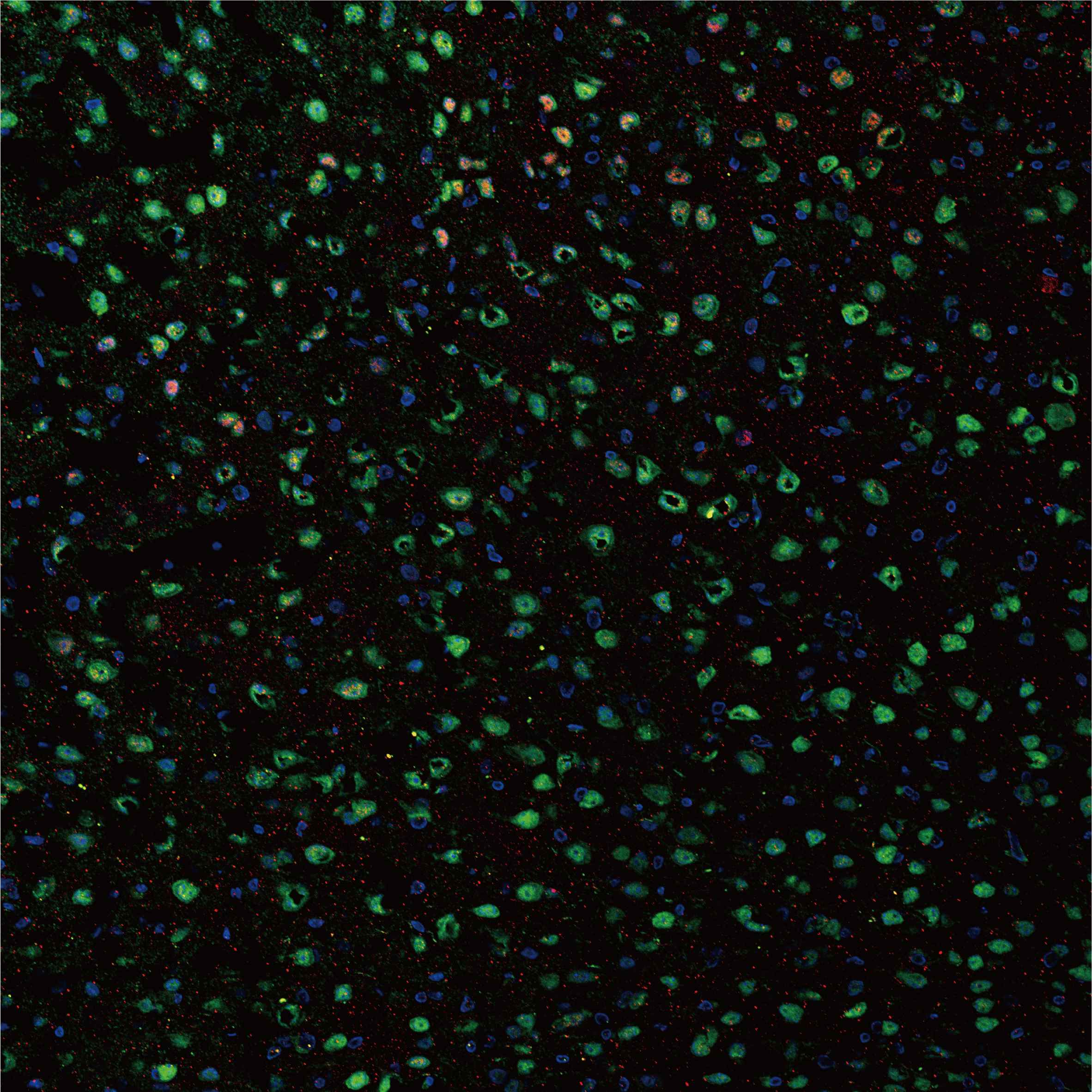

Supplement: Supplementary file 9 — Source data Fig. 7 [file 44321_2025_206_MOESM9_ESM.zip › Source data Fig 7/Fig 7/7J/WT-MCAO-LN5P45-MERGE.tif]

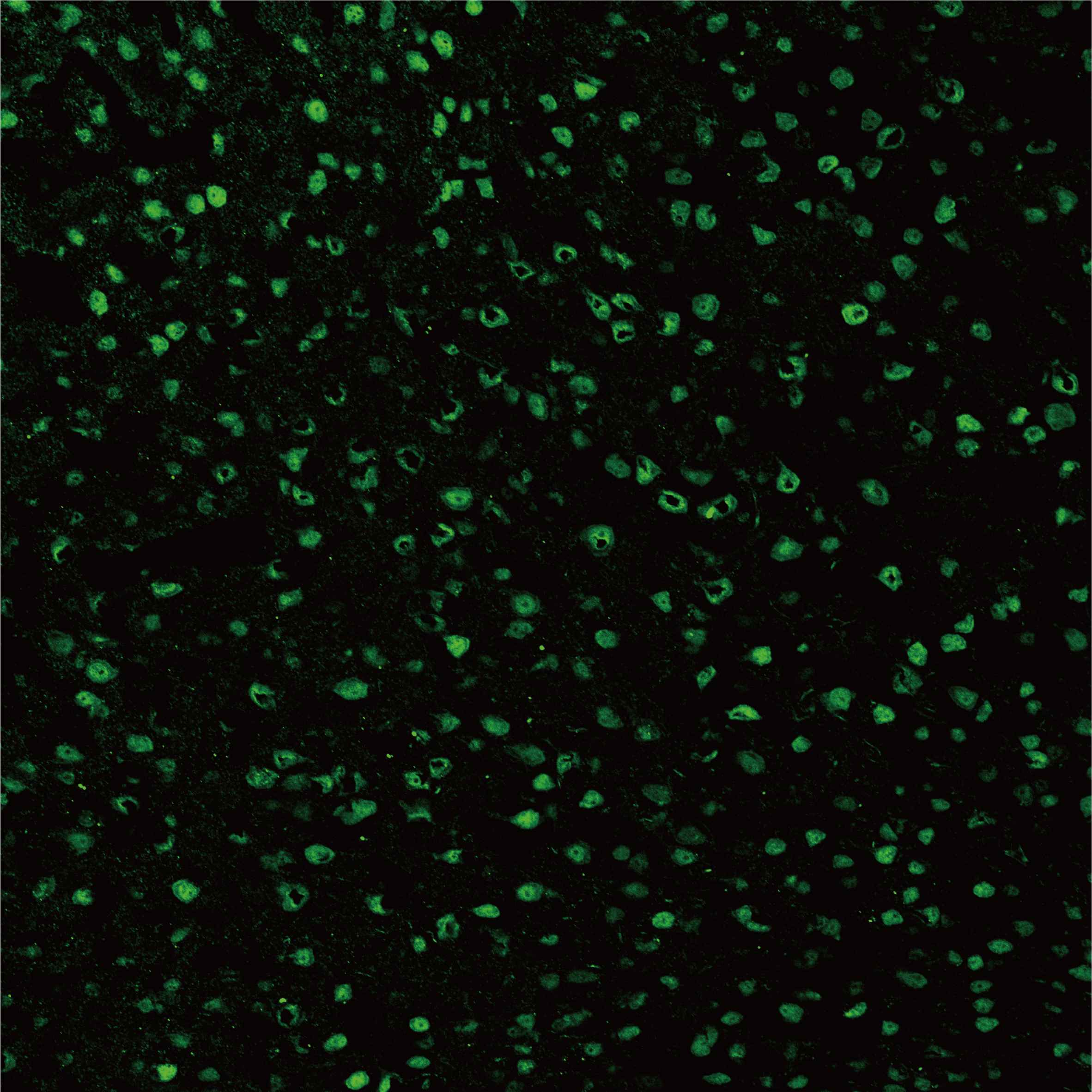

Supplement: Supplementary file 9 — Source data Fig. 7 [file 44321_2025_206_MOESM9_ESM.zip › Source data Fig 7/Fig 7/7J/WT-MCAO-LN5P45-NEUN.tif]

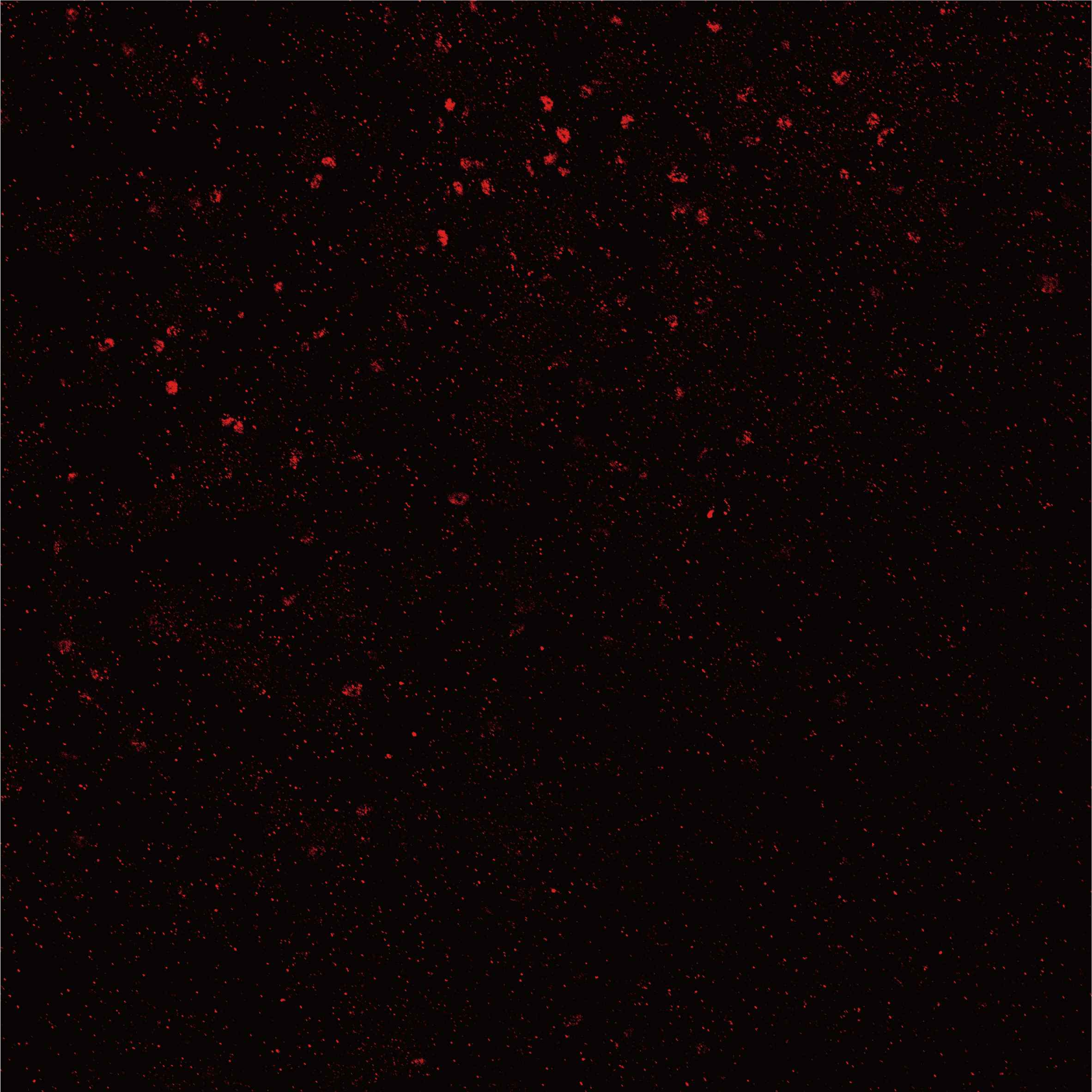

Supplement: Supplementary file 9 — Source data Fig. 7 [file 44321_2025_206_MOESM9_ESM.zip › Source data Fig 7/Fig 7/7J/WT-MCAO-LN5P45-P-MLKL.tif]

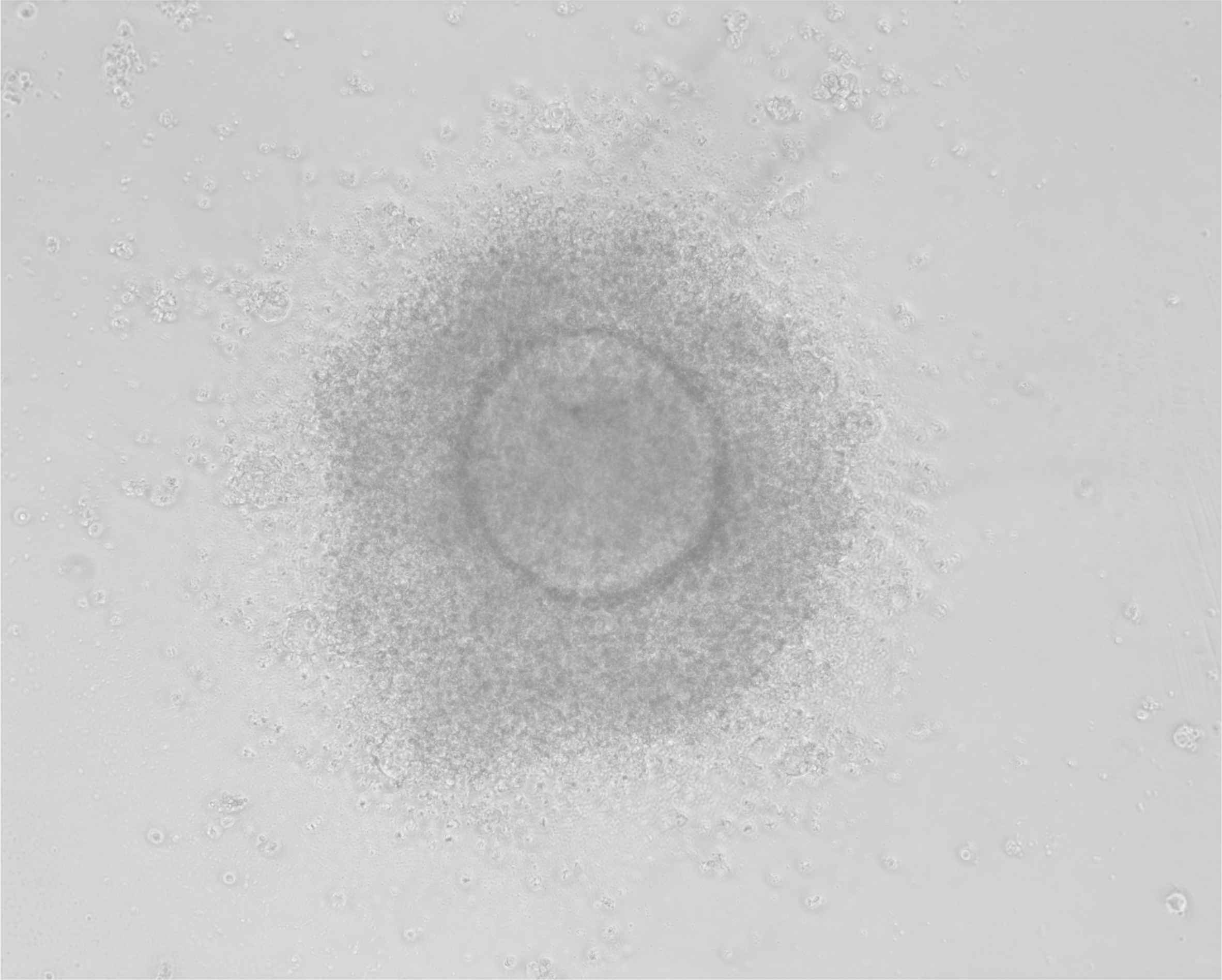

Supplement: Supplementary file 9 — Source data Fig. 7 [file 44321_2025_206_MOESM9_ESM.zip › Source data Fig 7/Fig 7/7K/7K-D1.jpg]

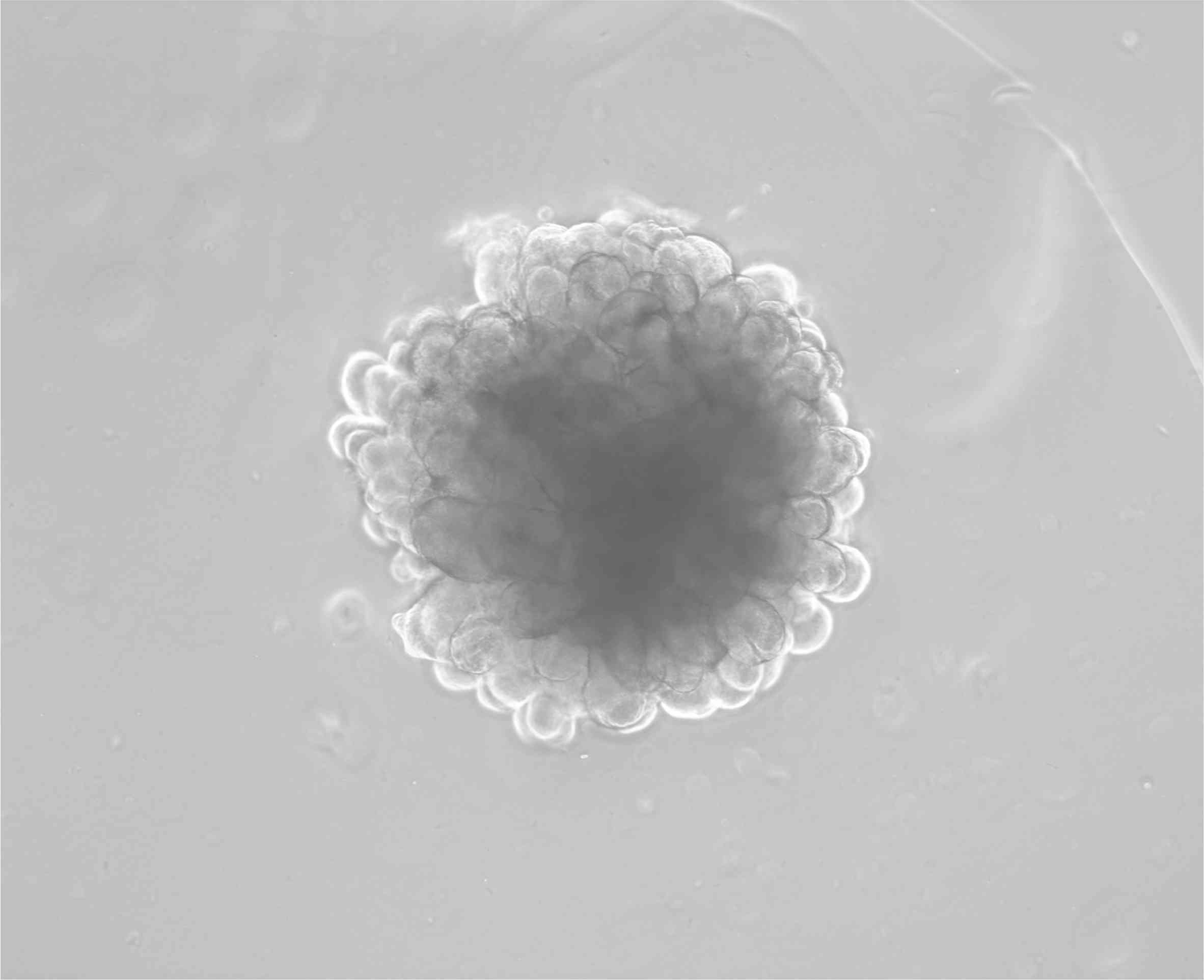

Supplement: Supplementary file 9 — Source data Fig. 7 [file 44321_2025_206_MOESM9_ESM.zip › Source data Fig 7/Fig 7/7K/7K-D10.tif]

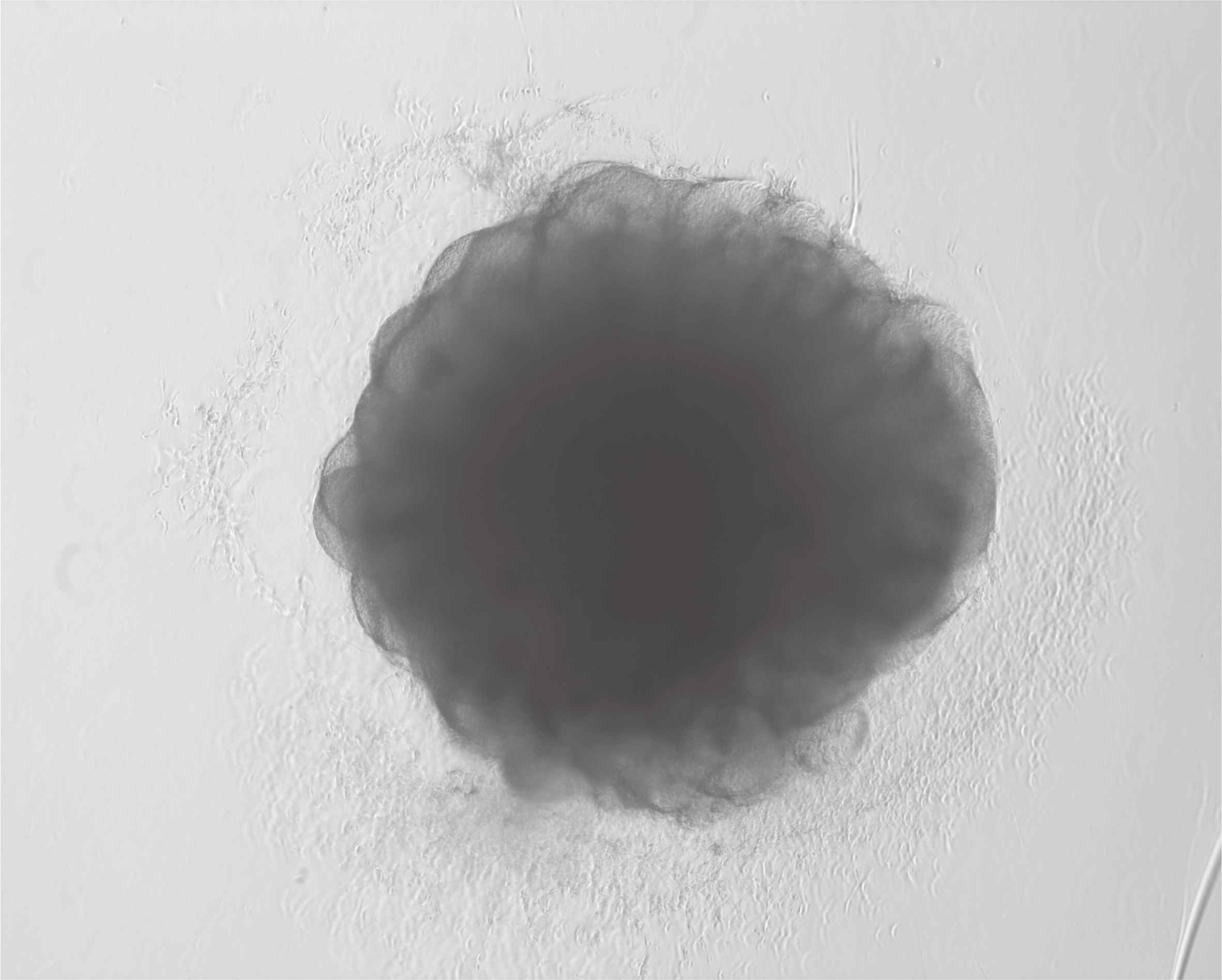

Supplement: Supplementary file 9 — Source data Fig. 7 [file 44321_2025_206_MOESM9_ESM.zip › Source data Fig 7/Fig 7/7K/7K-D15.tif]

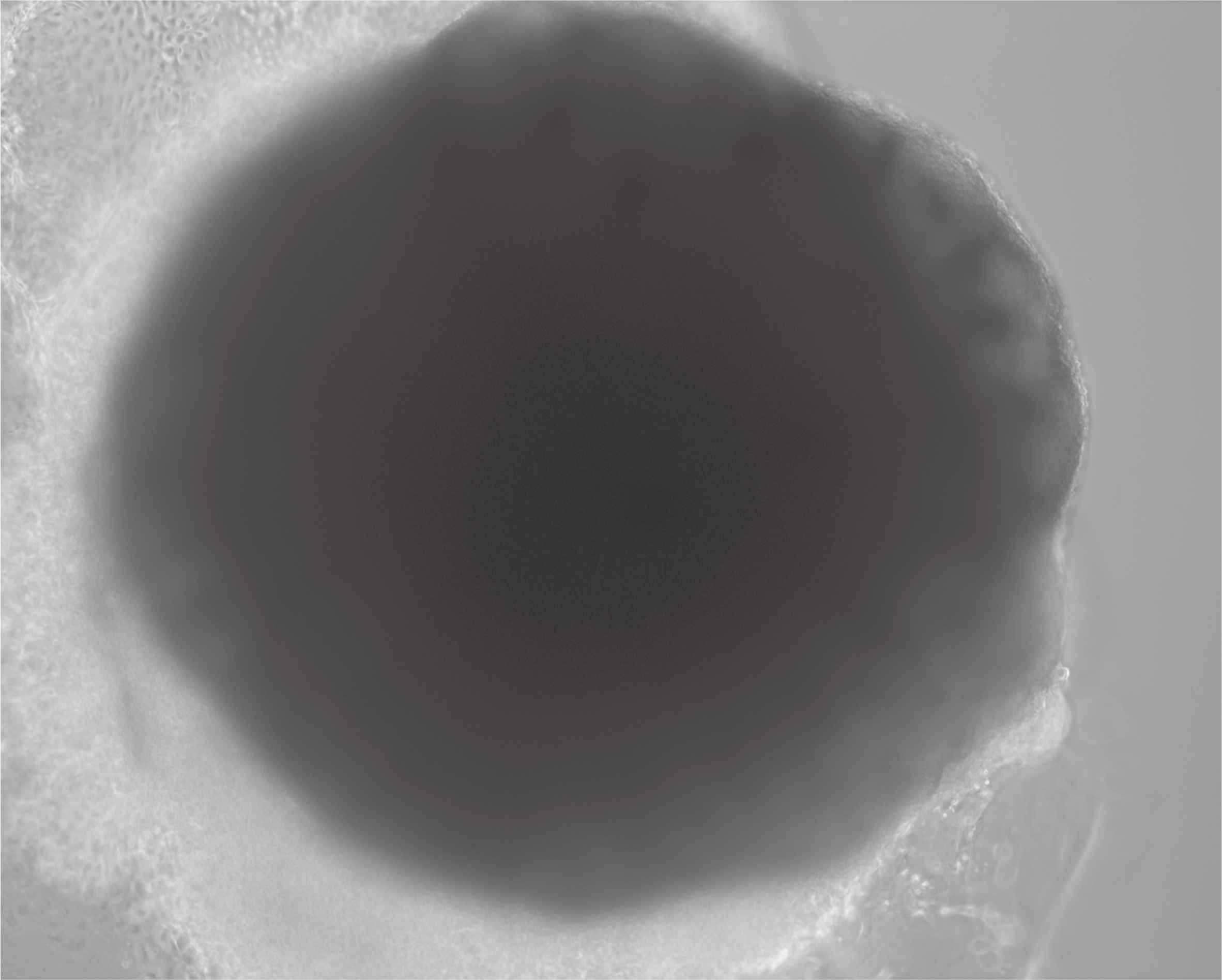

Supplement: Supplementary file 9 — Source data Fig. 7 [file 44321_2025_206_MOESM9_ESM.zip › Source data Fig 7/Fig 7/7K/7K-D25.tif]

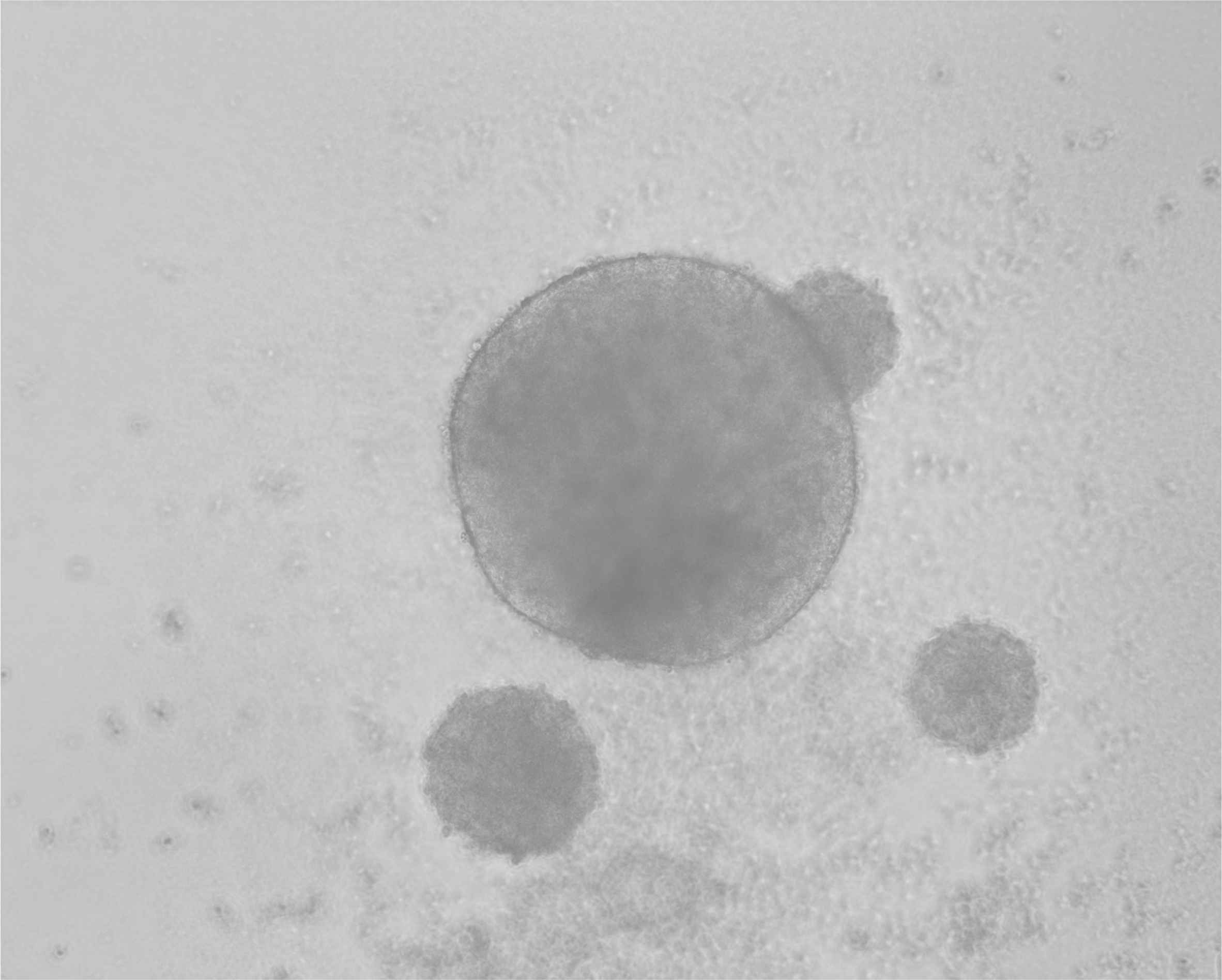

Supplement: Supplementary file 9 — Source data Fig. 7 [file 44321_2025_206_MOESM9_ESM.zip › Source data Fig 7/Fig 7/7K/7K-D5.tif]

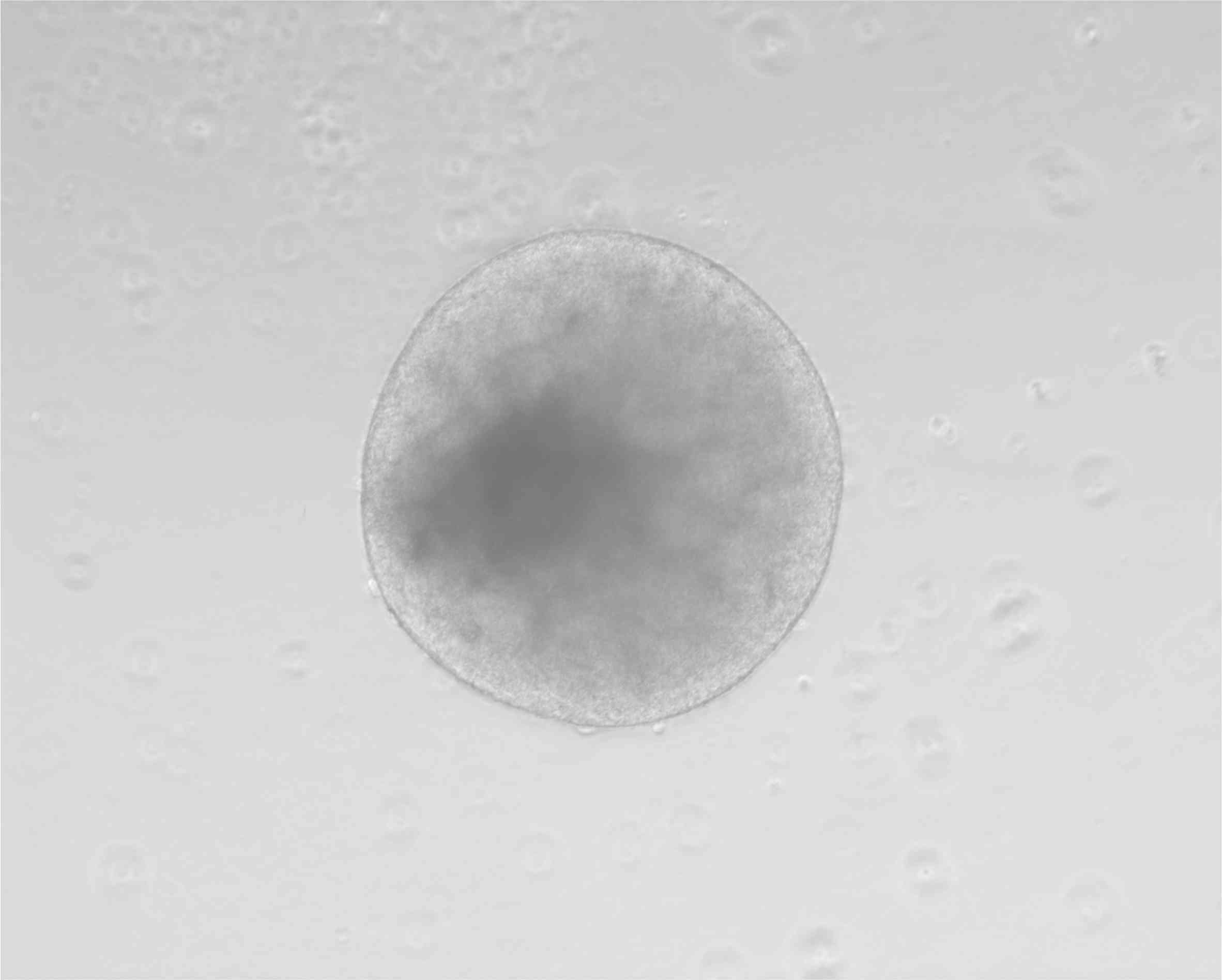

Supplement: Supplementary file 9 — Source data Fig. 7 [file 44321_2025_206_MOESM9_ESM.zip › Source data Fig 7/Fig 7/7K/7K-D7.tif]

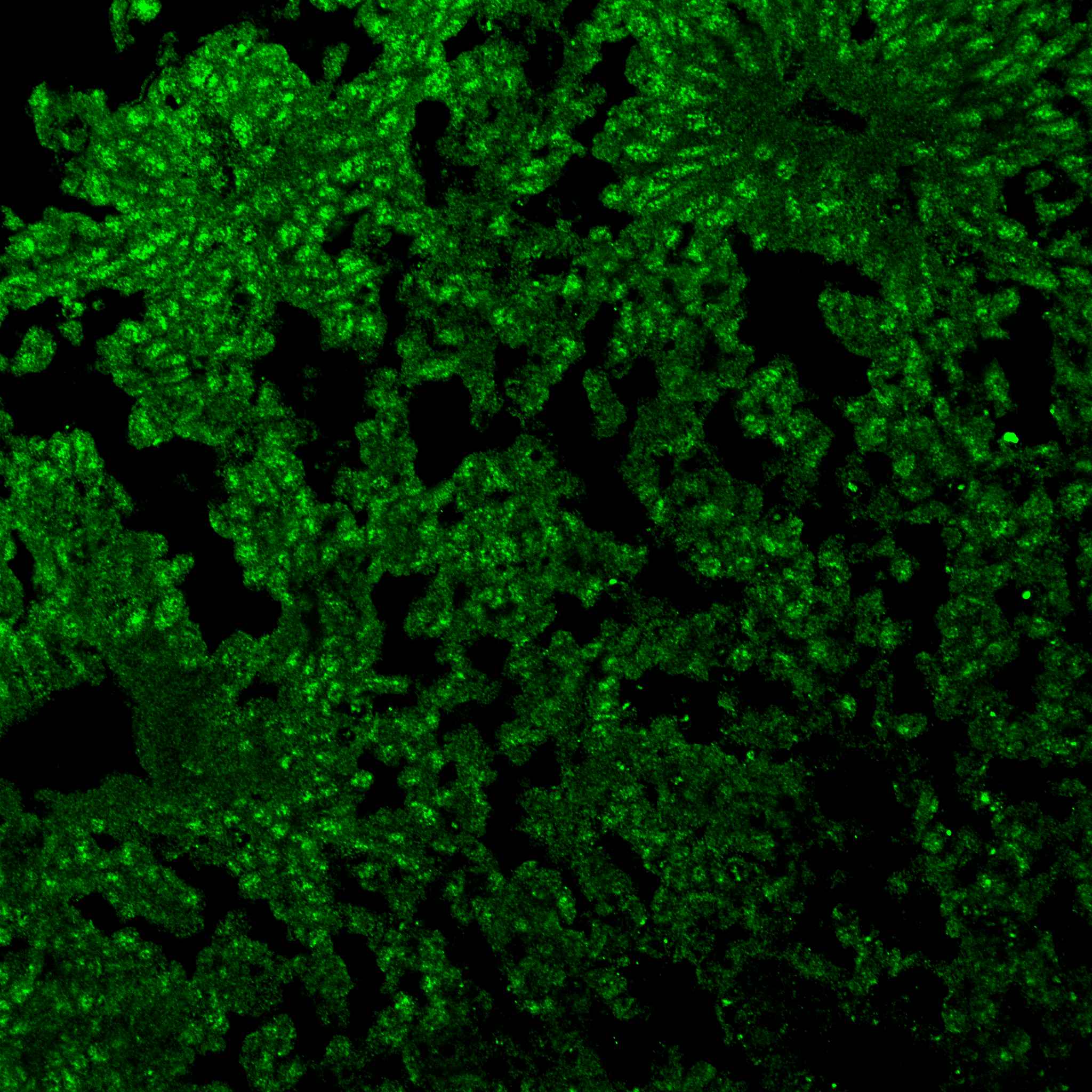

Supplement: Supplementary file 9 — Source data Fig. 7 [file 44321_2025_206_MOESM9_ESM.zip › Source data Fig 7/Fig 7/7M/LN5P45-Normoxia- NEUN.tif]

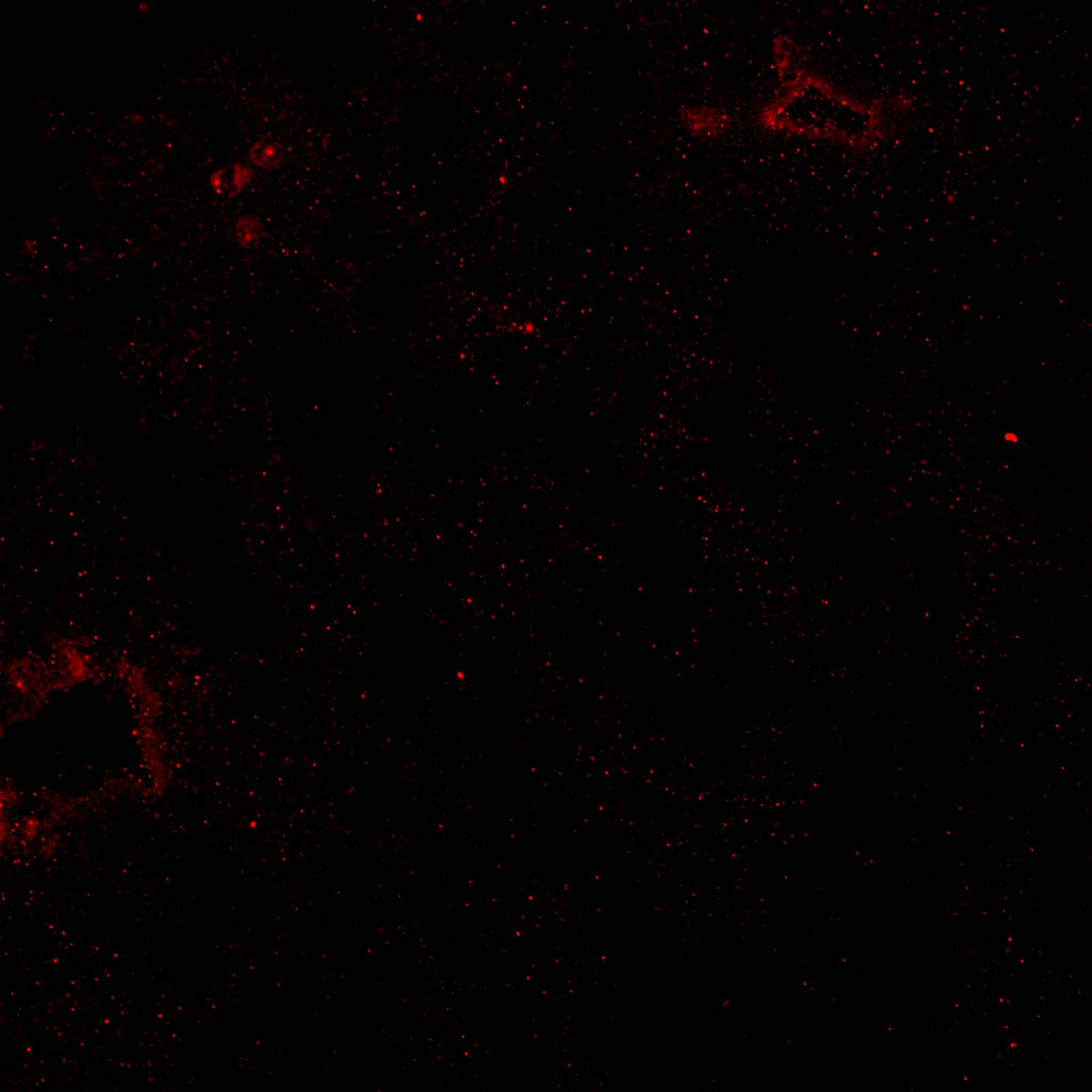

Supplement: Supplementary file 9 — Source data Fig. 7 [file 44321_2025_206_MOESM9_ESM.zip › Source data Fig 7/Fig 7/7M/LN5P45-Normoxia- P-MLKL.tif]

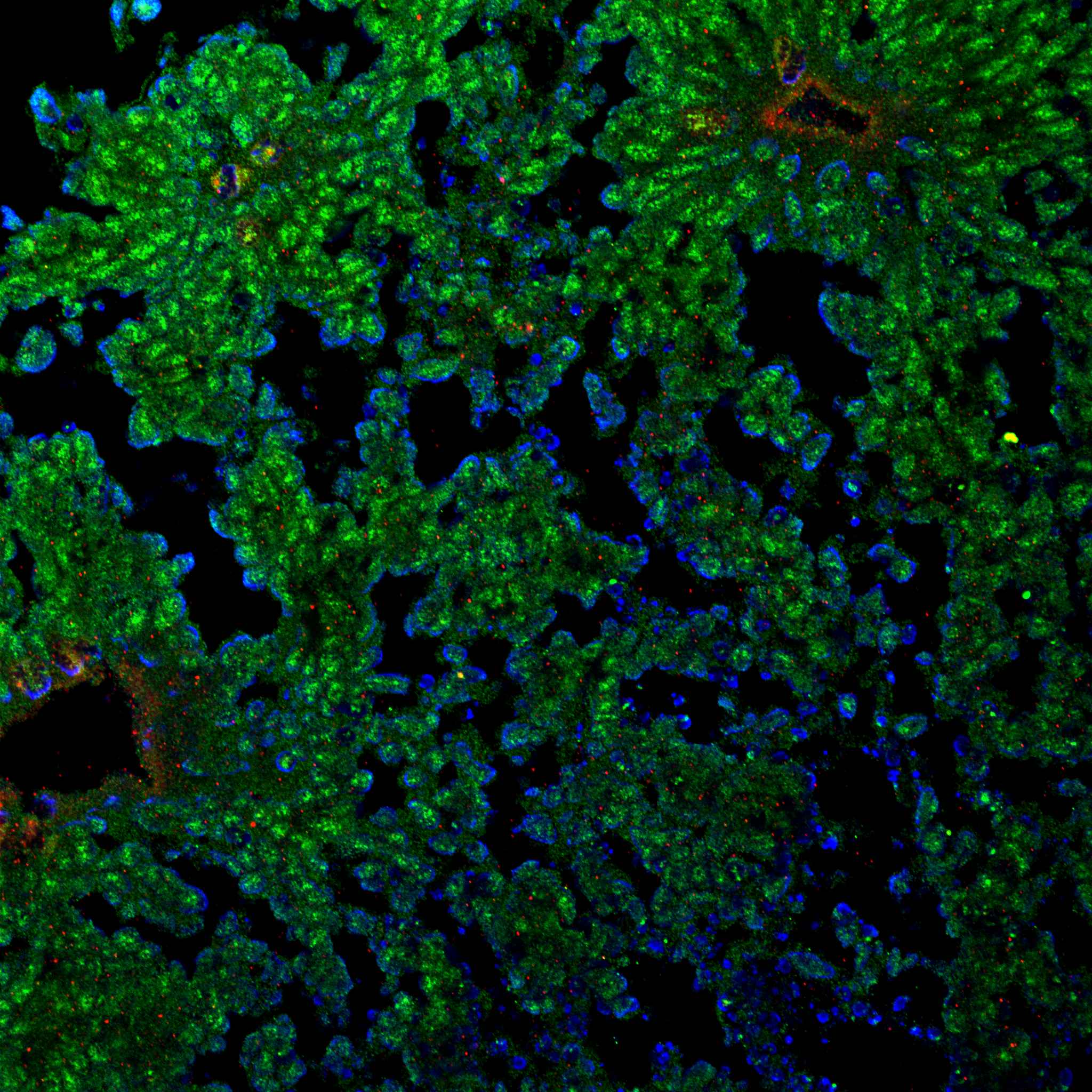

Supplement: Supplementary file 9 — Source data Fig. 7 [file 44321_2025_206_MOESM9_ESM.zip › Source data Fig 7/Fig 7/7M/LN5P45-Normoxia-MERGE.tif]

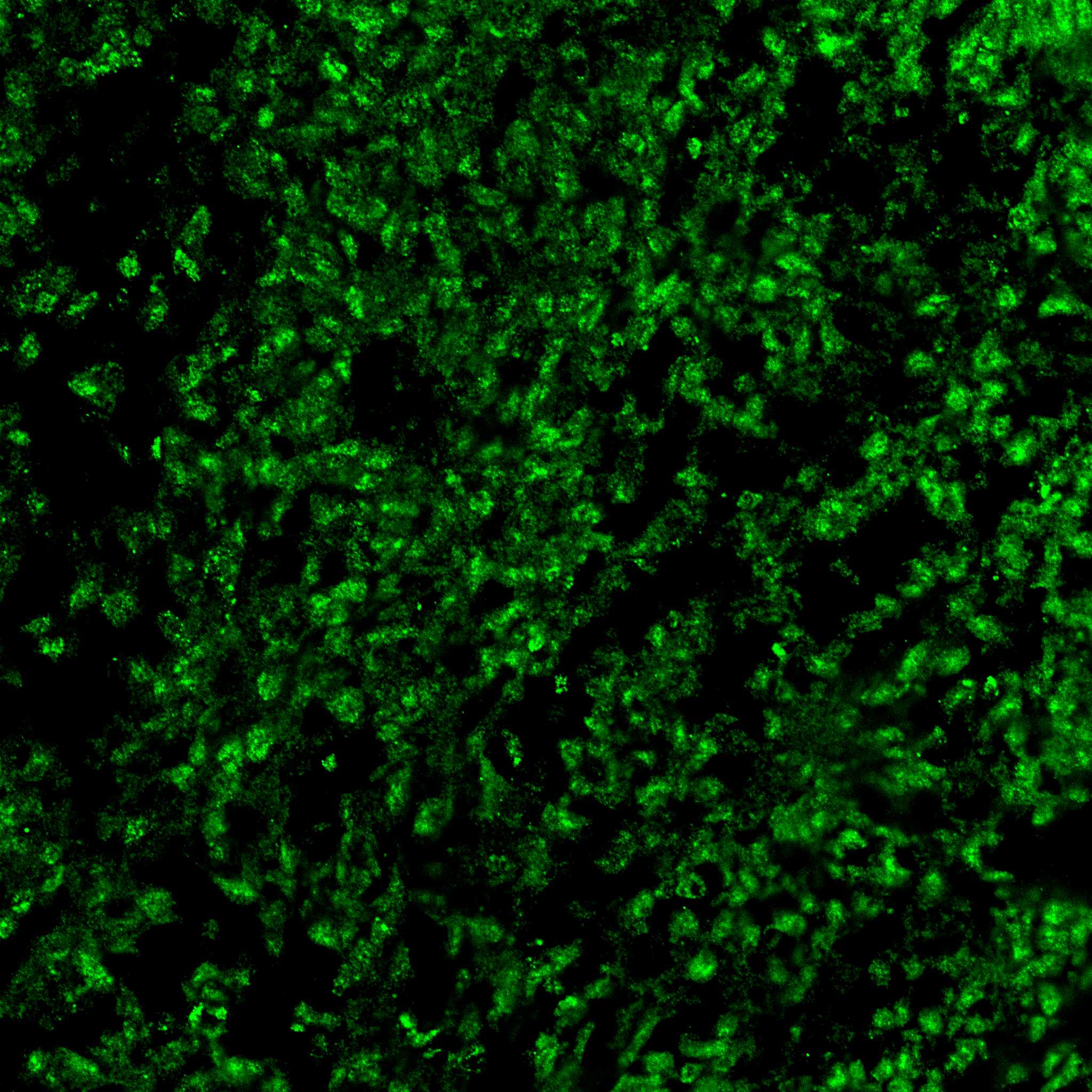

Supplement: Supplementary file 9 — Source data Fig. 7 [file 44321_2025_206_MOESM9_ESM.zip › Source data Fig 7/Fig 7/7M/LN5P45-OGD NEUN.tif]

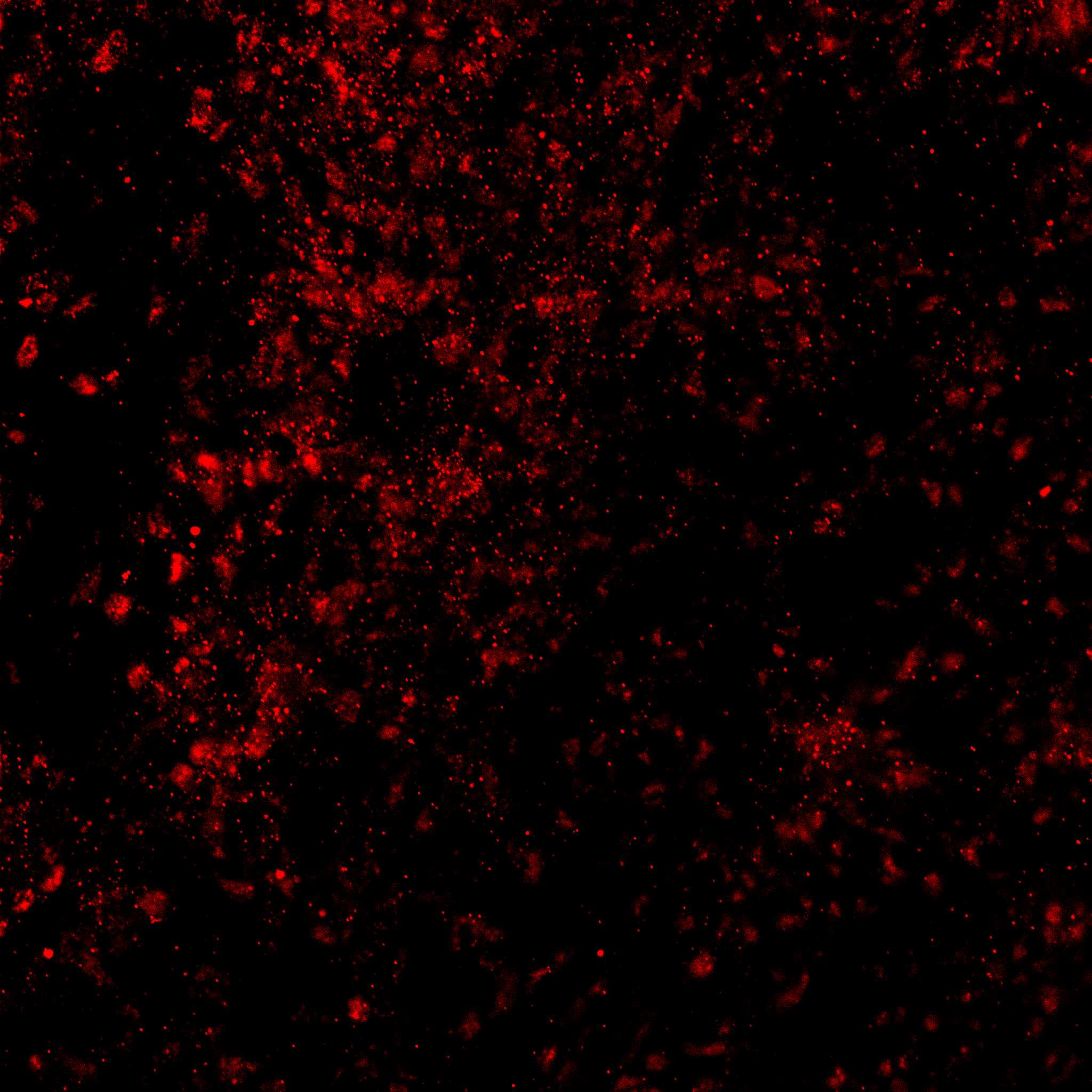

Supplement: Supplementary file 9 — Source data Fig. 7 [file 44321_2025_206_MOESM9_ESM.zip › Source data Fig 7/Fig 7/7M/LN5P45-OGD P-MLKL.tif]

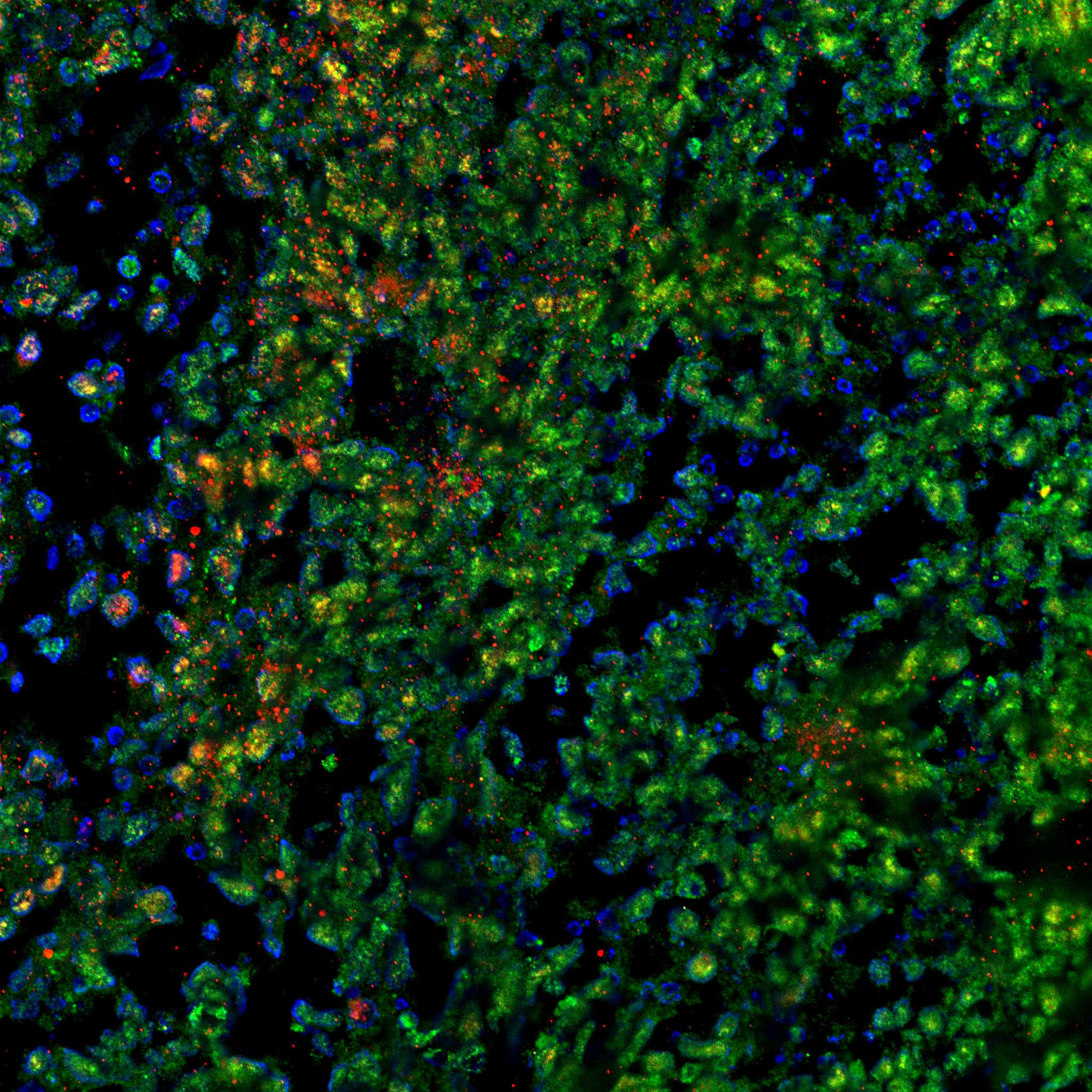

Supplement: Supplementary file 9 — Source data Fig. 7 [file 44321_2025_206_MOESM9_ESM.zip › Source data Fig 7/Fig 7/7M/LN5P45-OGD MERGE.tif]

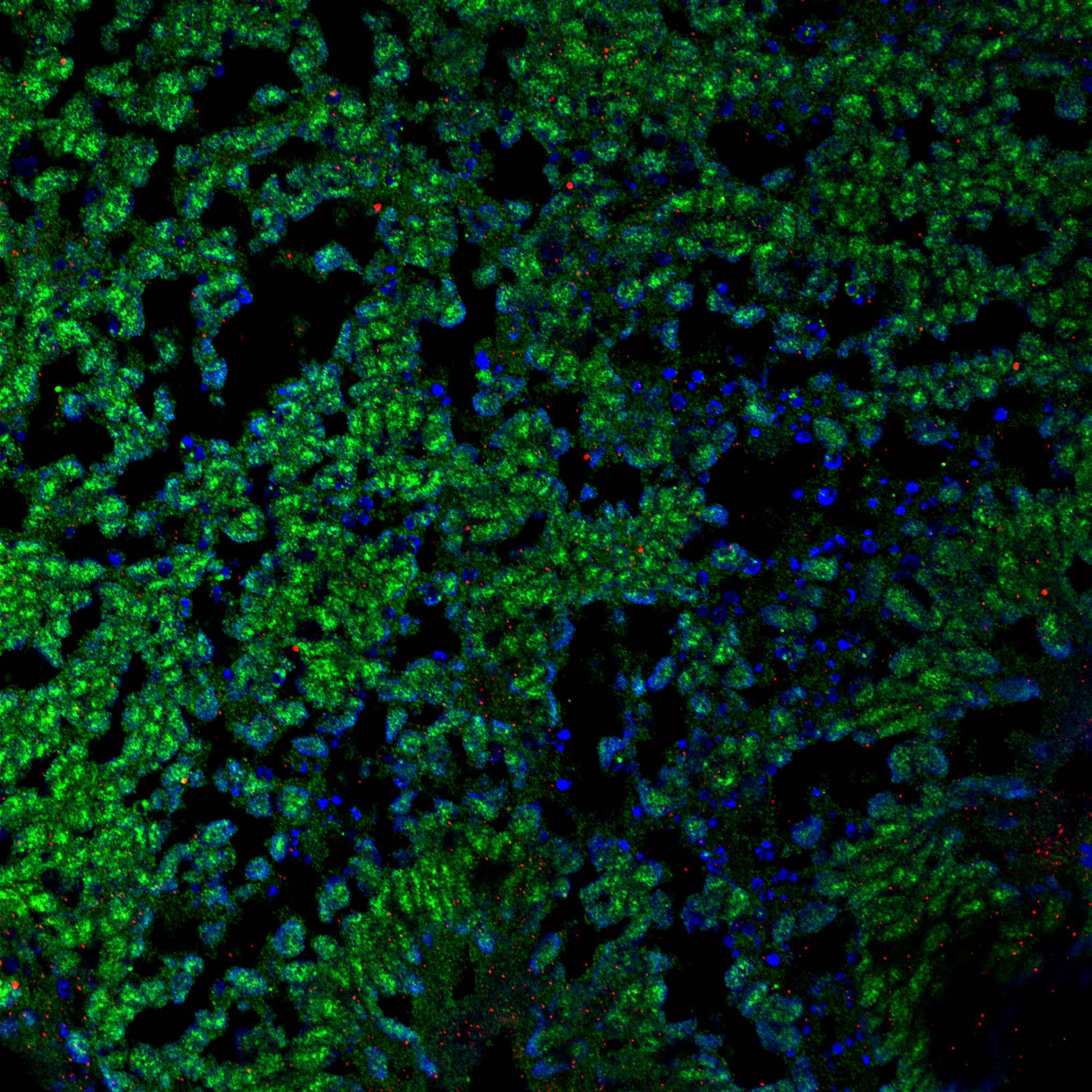

Supplement: Supplementary file 9 — Source data Fig. 7 [file 44321_2025_206_MOESM9_ESM.zip › Source data Fig 7/Fig 7/7M/WT-Normoxia-MERGE.tif]

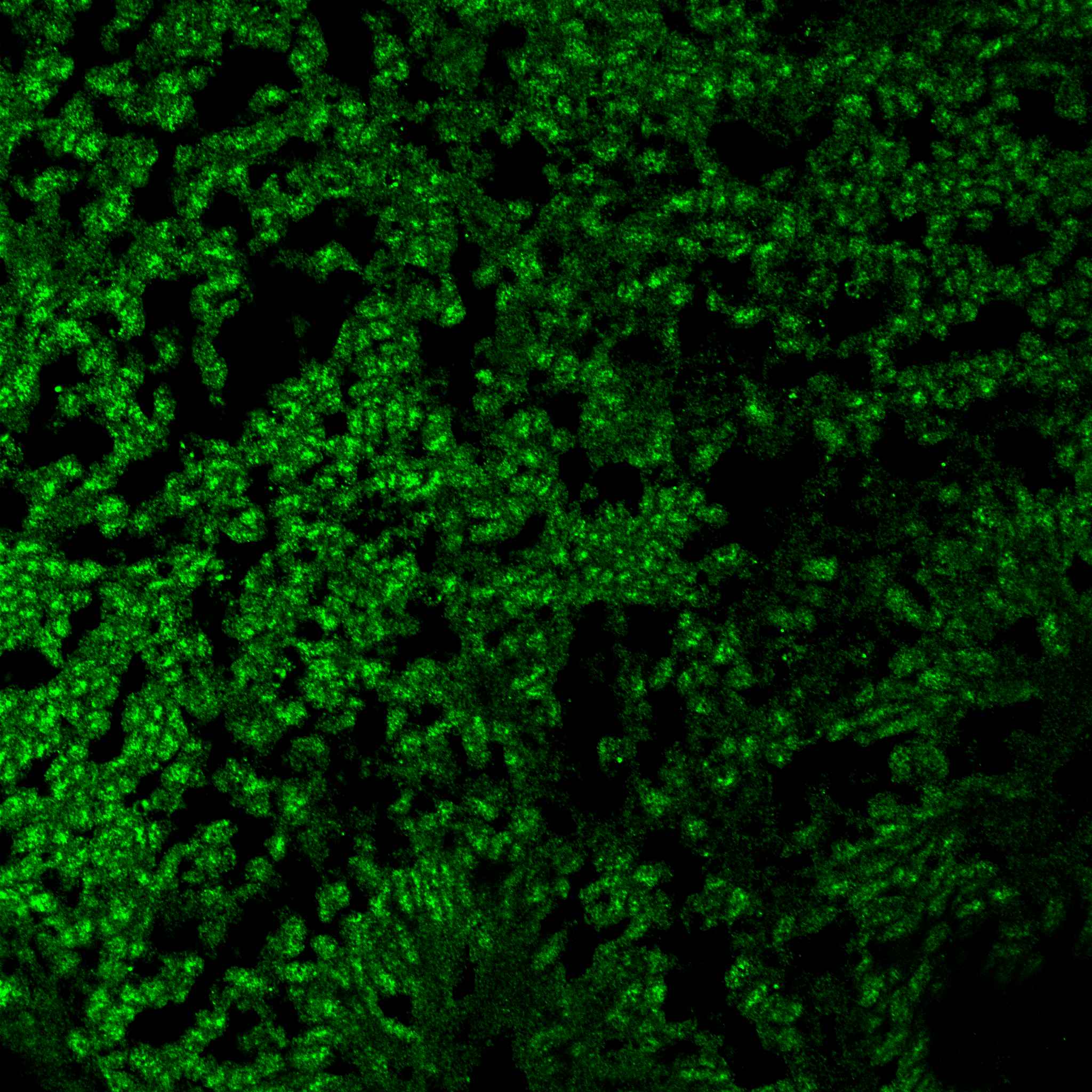

Supplement: Supplementary file 9 — Source data Fig. 7 [file 44321_2025_206_MOESM9_ESM.zip › Source data Fig 7/Fig 7/7M/WT-Normoxia-NEUN.tif]

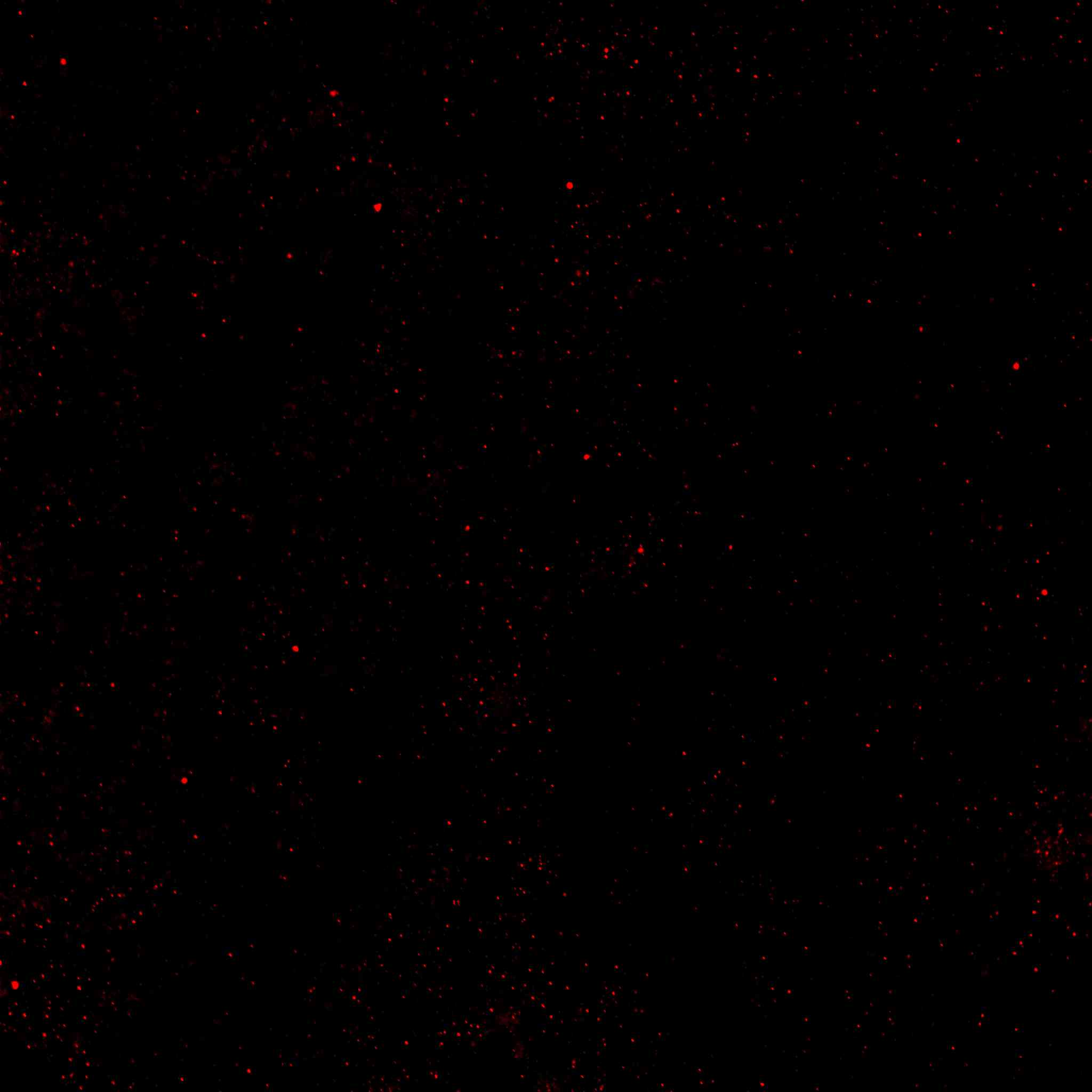

Supplement: Supplementary file 9 — Source data Fig. 7 [file 44321_2025_206_MOESM9_ESM.zip › Source data Fig 7/Fig 7/7M/WT-Normoxia-P-MLKL.tif]

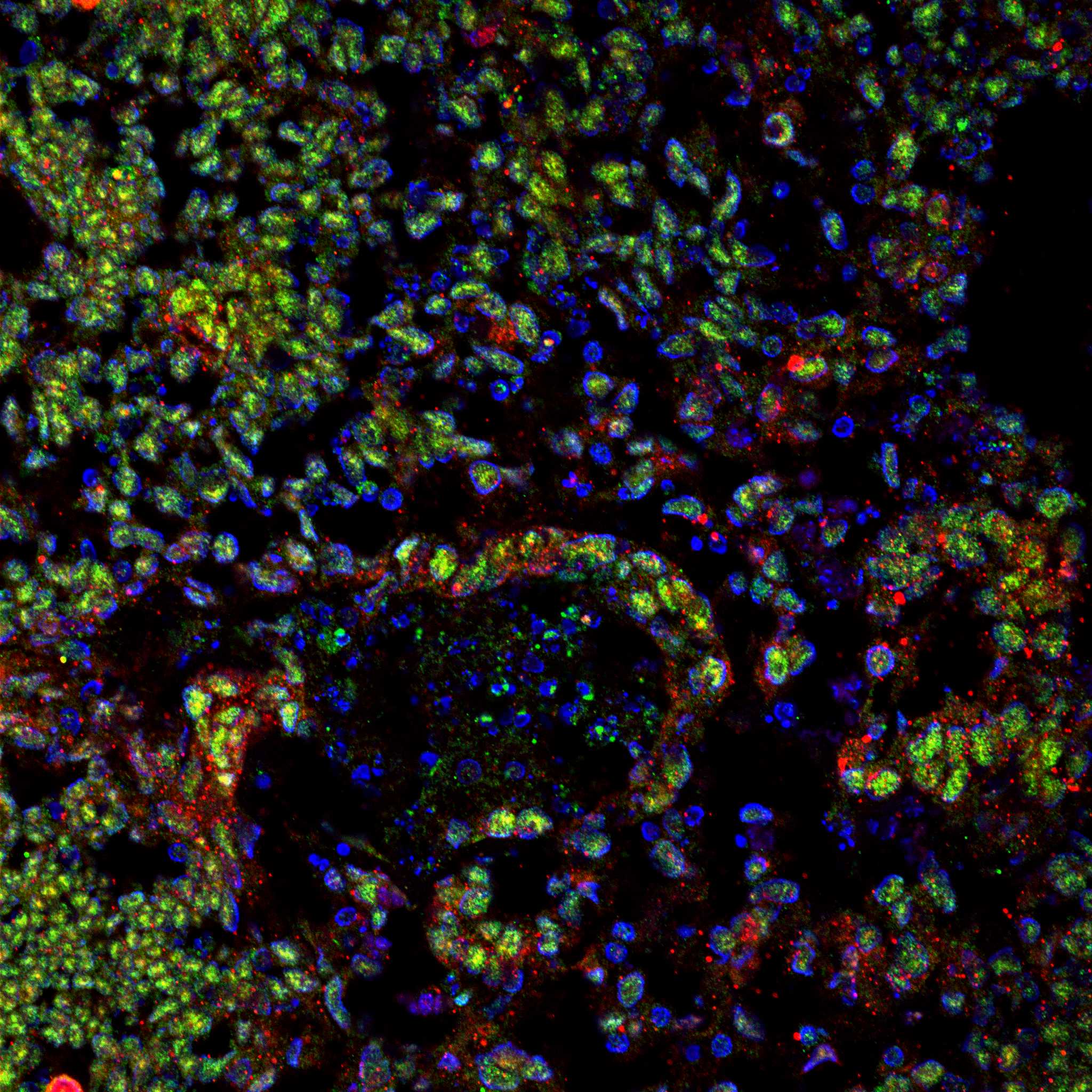

Supplement: Supplementary file 9 — Source data Fig. 7 [file 44321_2025_206_MOESM9_ESM.zip › Source data Fig 7/Fig 7/7M/WT-OGD MERGE.tif]

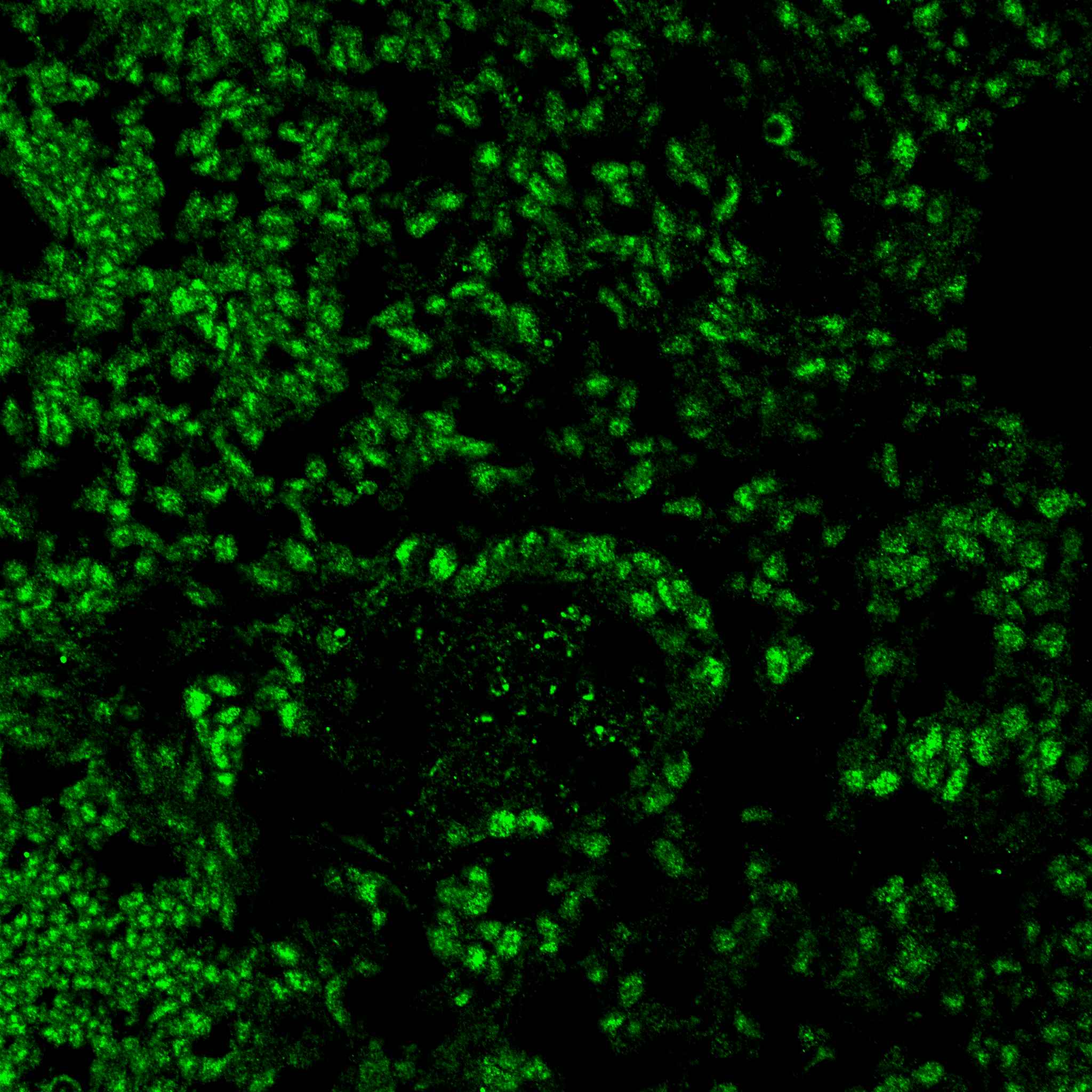

Supplement: Supplementary file 9 — Source data Fig. 7 [file 44321_2025_206_MOESM9_ESM.zip › Source data Fig 7/Fig 7/7M/WT-OGD NEUN.tif]

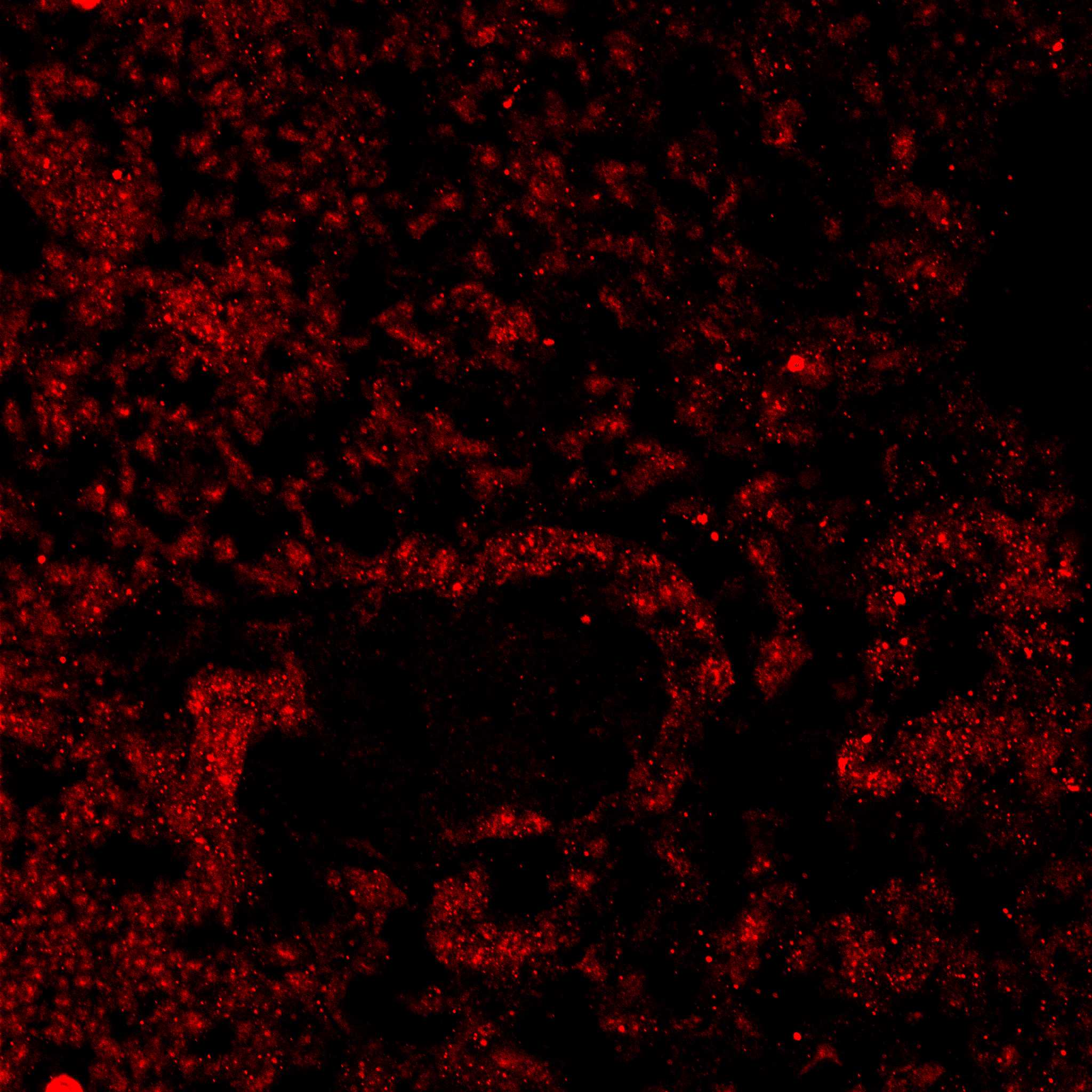

Supplement: Supplementary file 9 — Source data Fig. 7 [file 44321_2025_206_MOESM9_ESM.zip › Source data Fig 7/Fig 7/7M/WT-OGD P-MLKL.tif]

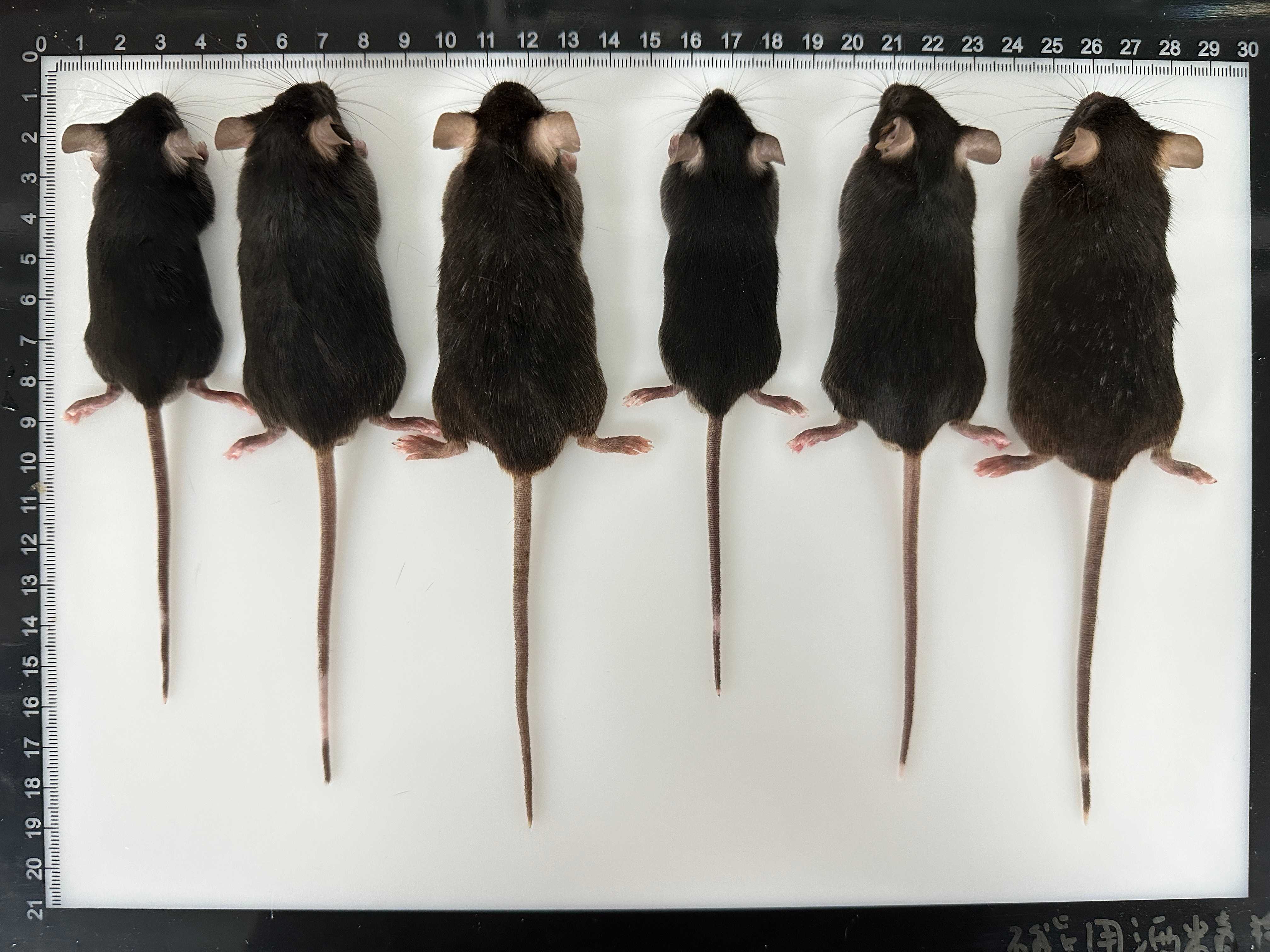

Supplement: Supplementary file 10 — Appendix Figure Source Data [file 44321_2025_206_MOESM10_ESM.zip › Appendix Figures Source Data/Appendix Fig. S1/S1-A-B/S1-A.jpg]

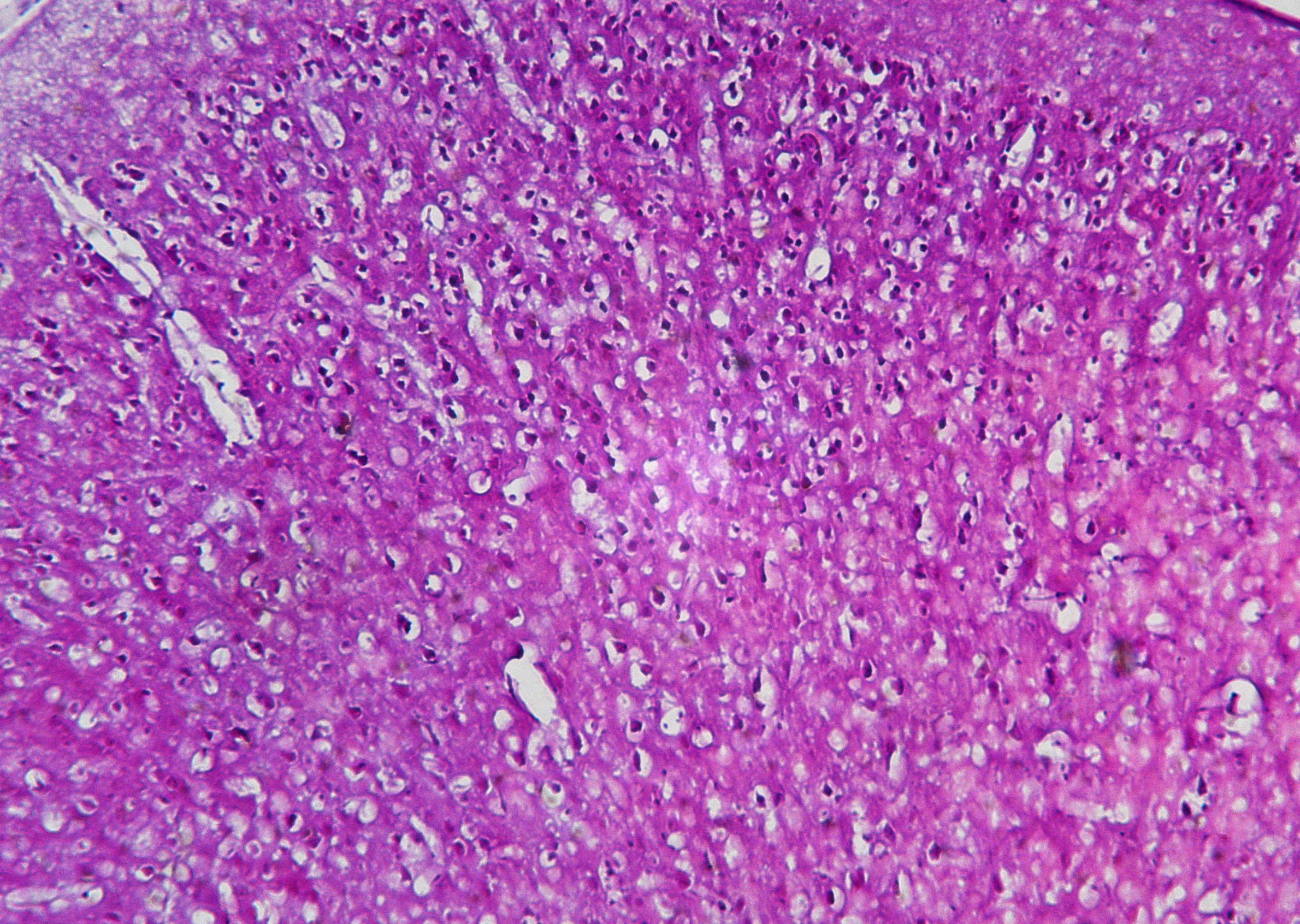

Supplement: Supplementary file 10 — Appendix Figure Source Data [file 44321_2025_206_MOESM10_ESM.zip › Appendix Figures Source Data/Appendix Fig. S1/S1-C-D/KO-HE.tif]

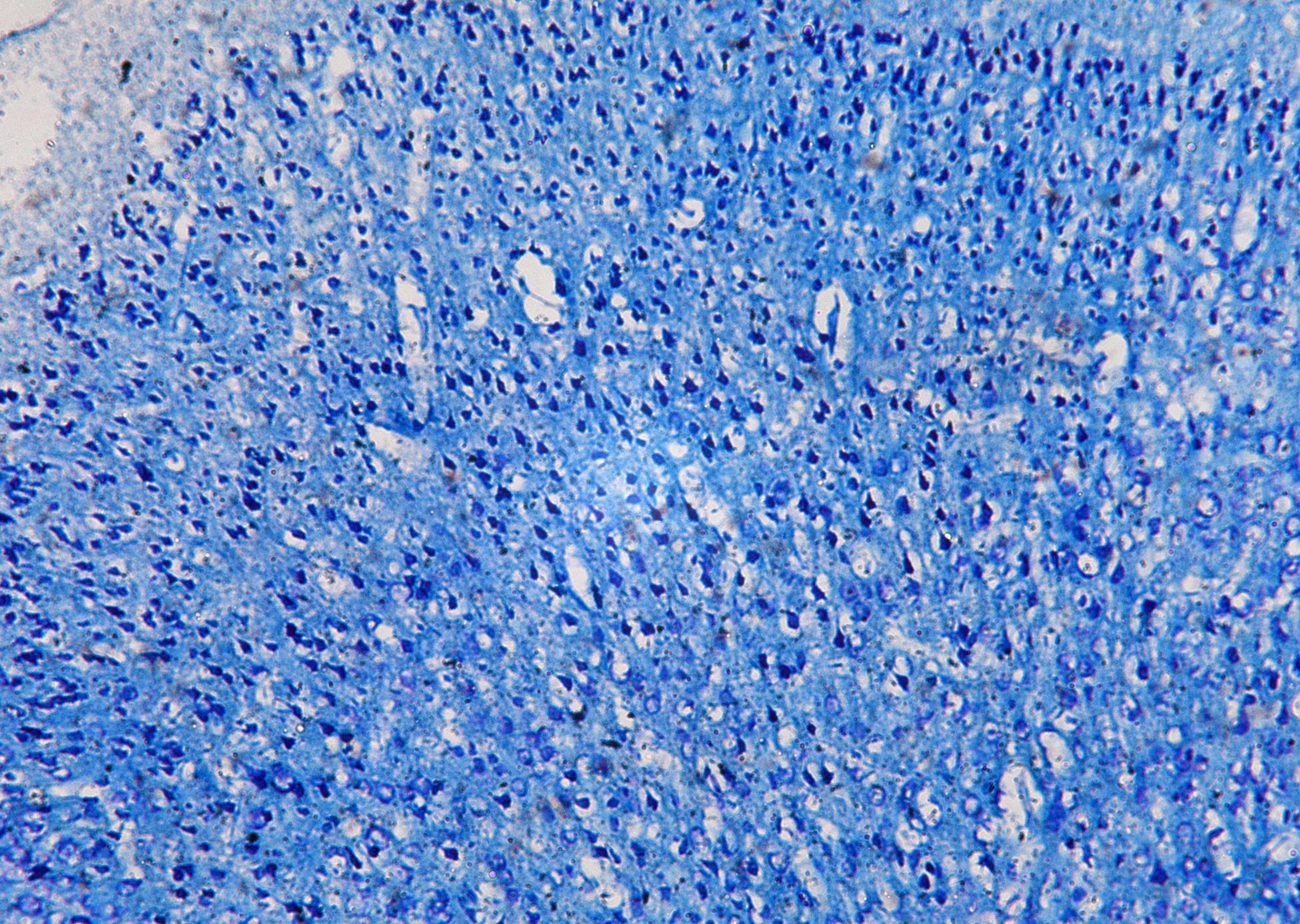

Supplement: Supplementary file 10 — Appendix Figure Source Data [file 44321_2025_206_MOESM10_ESM.zip › Appendix Figures Source Data/Appendix Fig. S1/S1-C-D/KO-Nissl.tif]

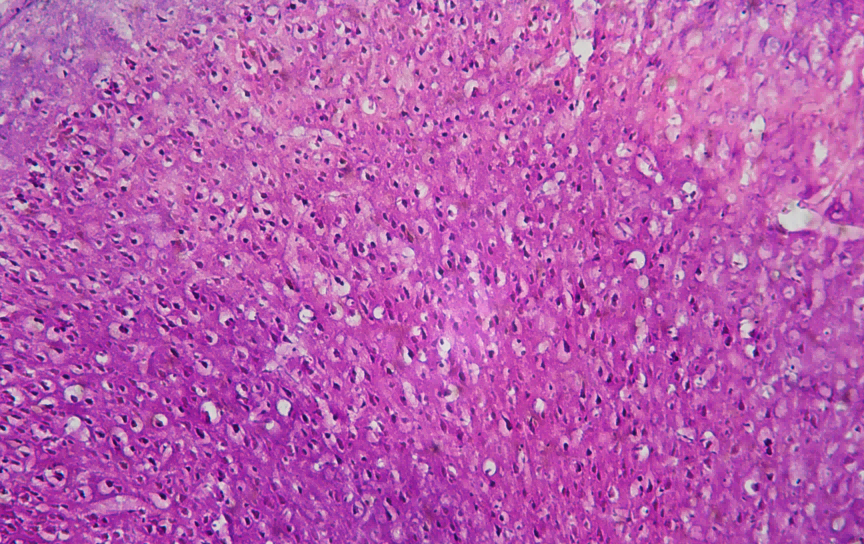

Supplement: Supplementary file 10 — Appendix Figure Source Data [file 44321_2025_206_MOESM10_ESM.zip › Appendix Figures Source Data/Appendix Fig. S1/S1-C-D/WT-HE.png]

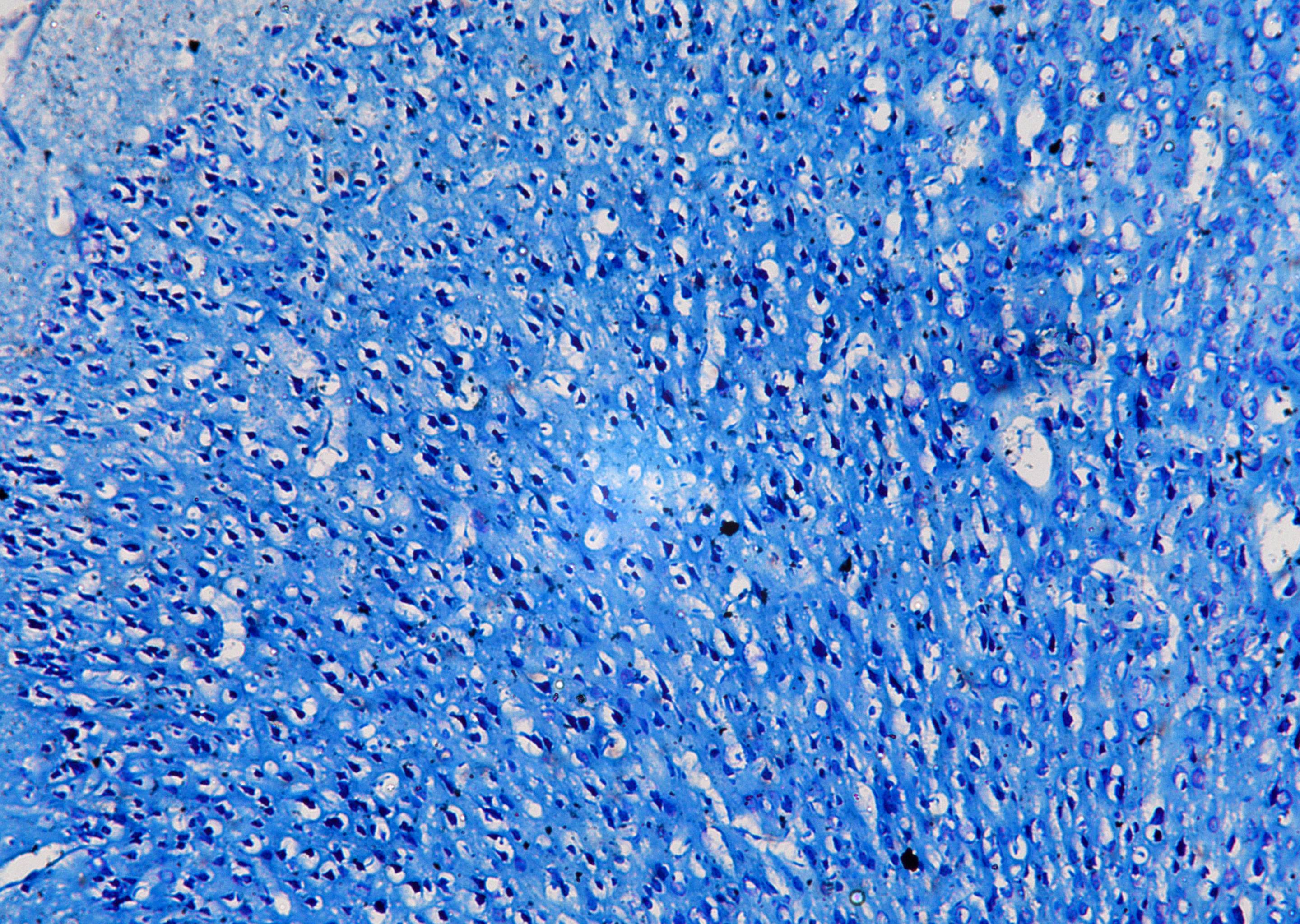

Supplement: Supplementary file 10 — Appendix Figure Source Data [file 44321_2025_206_MOESM10_ESM.zip › Appendix Figures Source Data/Appendix Fig. S1/S1-C-D/WT-Nissl.tif]

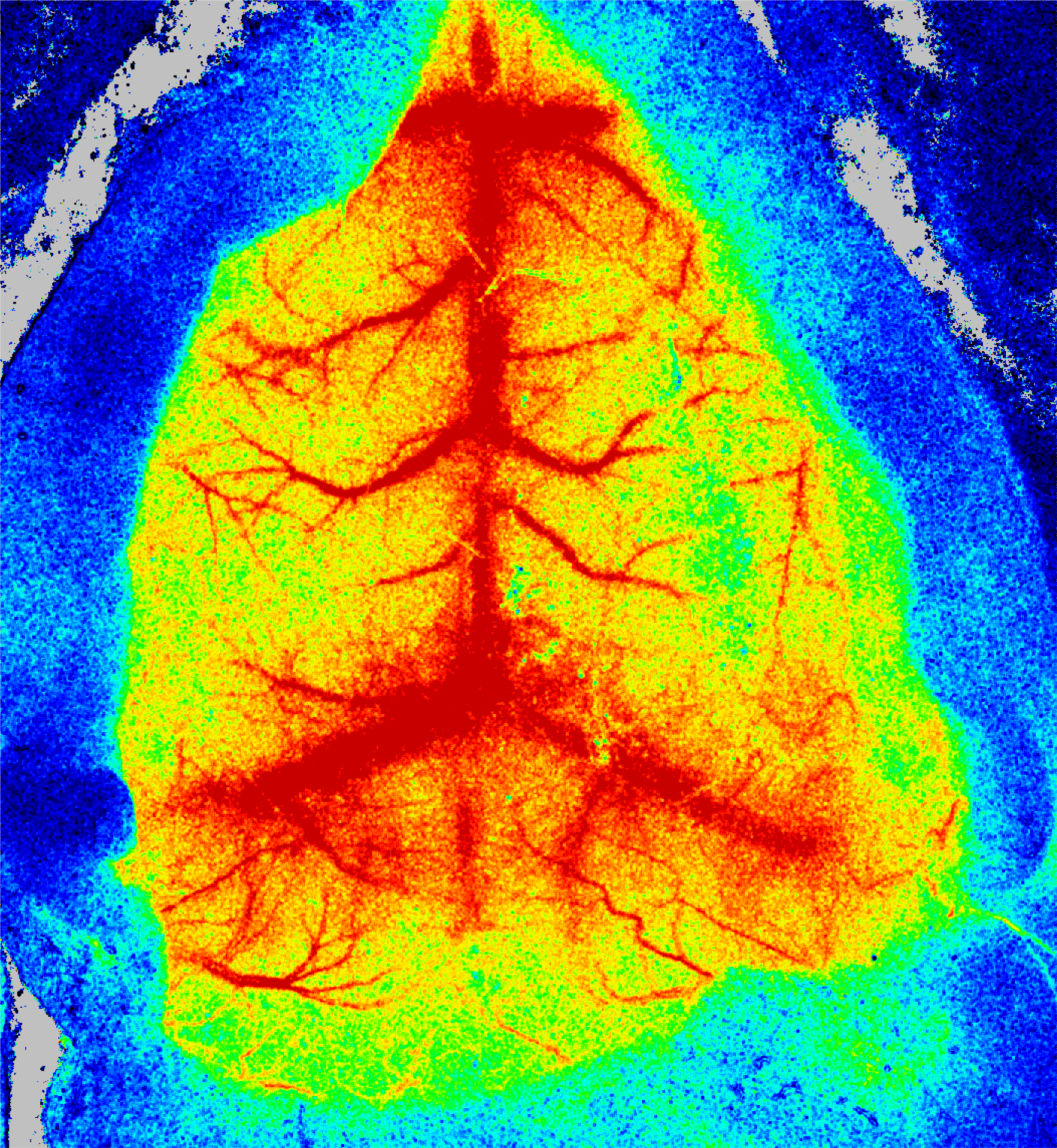

Supplement: Supplementary file 10 — Appendix Figure Source Data [file 44321_2025_206_MOESM10_ESM.zip › Appendix Figures Source Data/Appendix Fig. S1/S1-E-F/KO1.png]

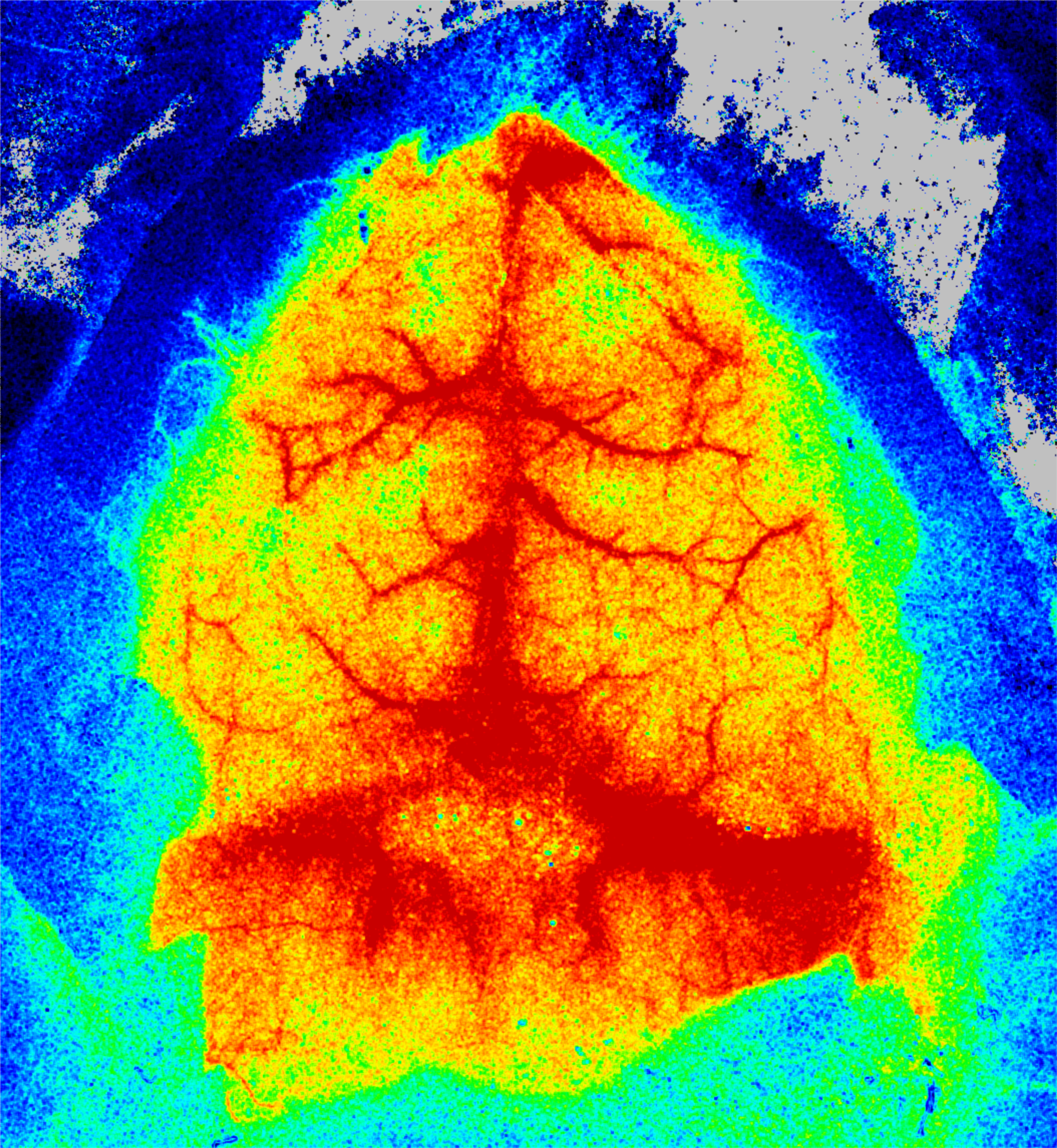

Supplement: Supplementary file 10 — Appendix Figure Source Data [file 44321_2025_206_MOESM10_ESM.zip › Appendix Figures Source Data/Appendix Fig. S1/S1-E-F/KO2.png]

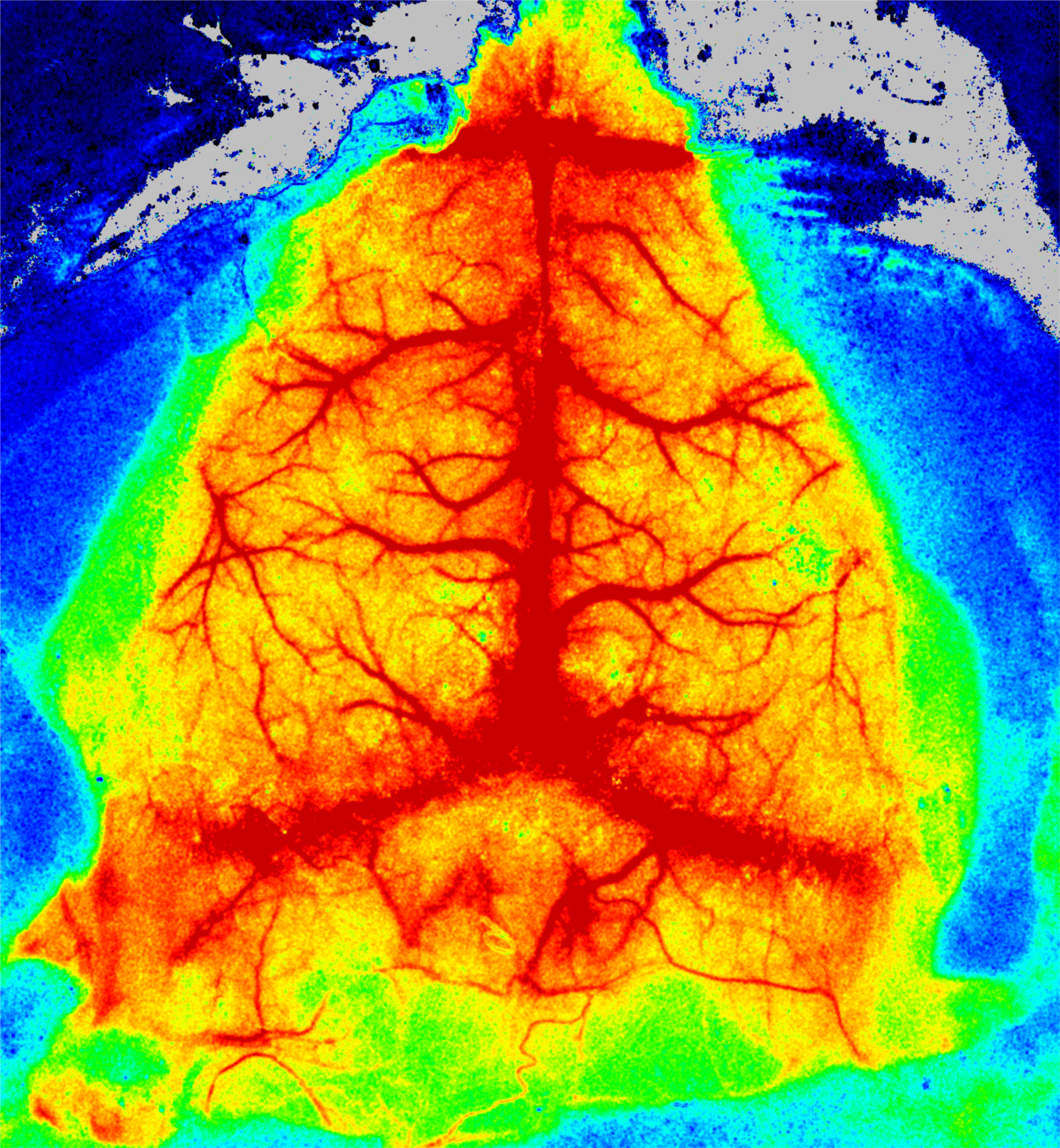

Supplement: Supplementary file 10 — Appendix Figure Source Data [file 44321_2025_206_MOESM10_ESM.zip › Appendix Figures Source Data/Appendix Fig. S1/S1-E-F/KO3.png]

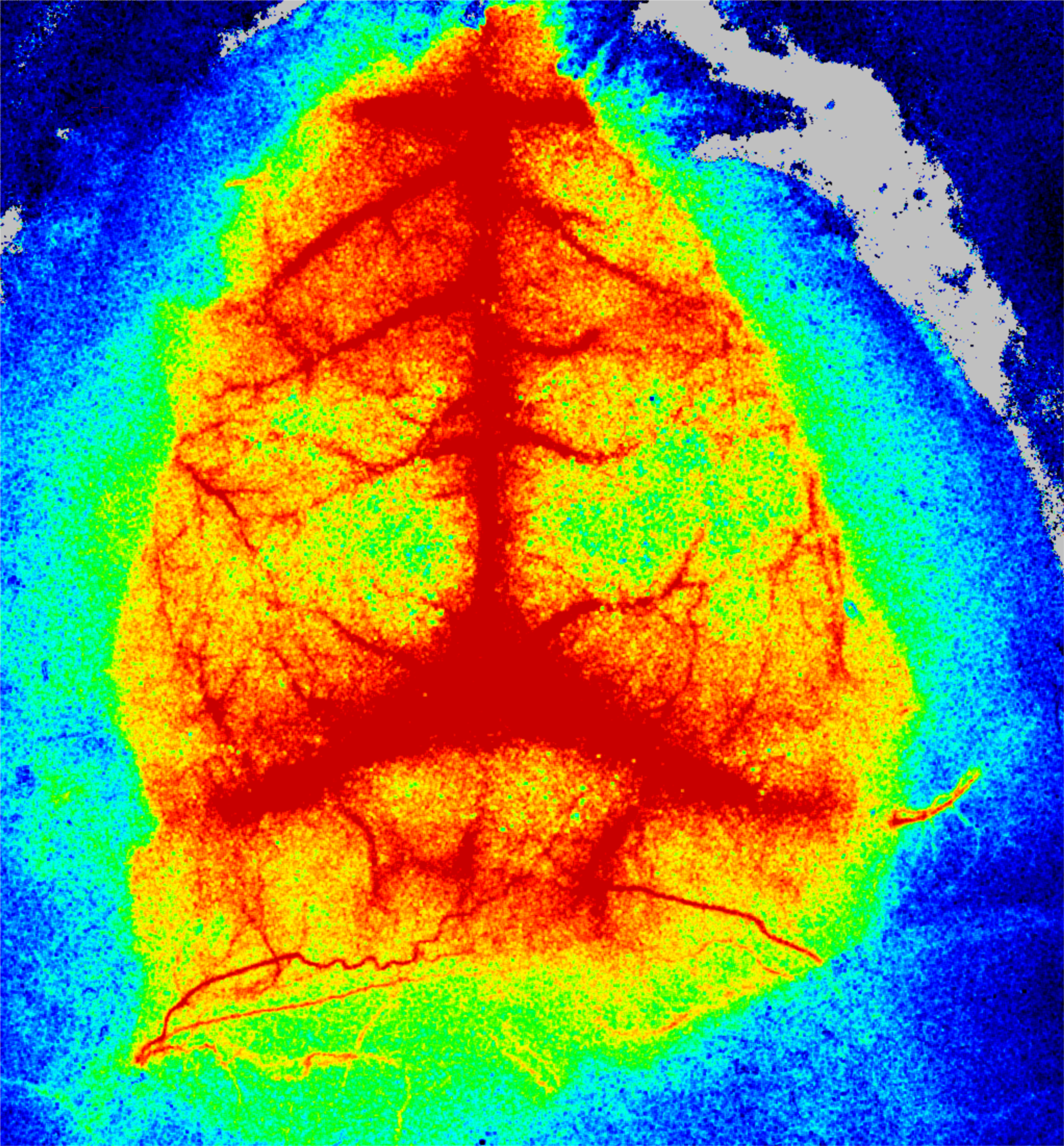

Supplement: Supplementary file 10 — Appendix Figure Source Data [file 44321_2025_206_MOESM10_ESM.zip › Appendix Figures Source Data/Appendix Fig. S1/S1-E-F/WT1.png]

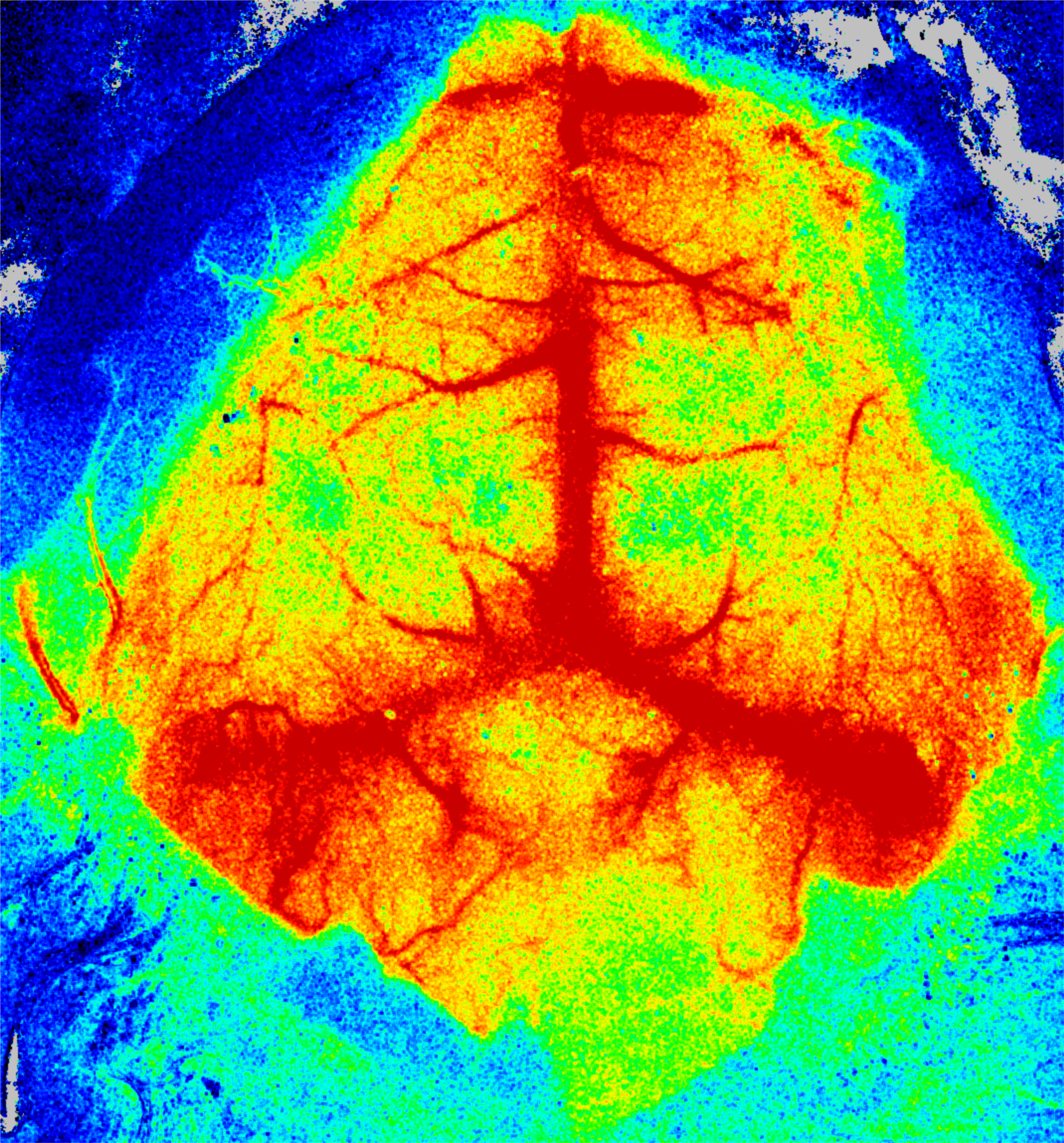

Supplement: Supplementary file 10 — Appendix Figure Source Data [file 44321_2025_206_MOESM10_ESM.zip › Appendix Figures Source Data/Appendix Fig. S1/S1-E-F/WT2.png]

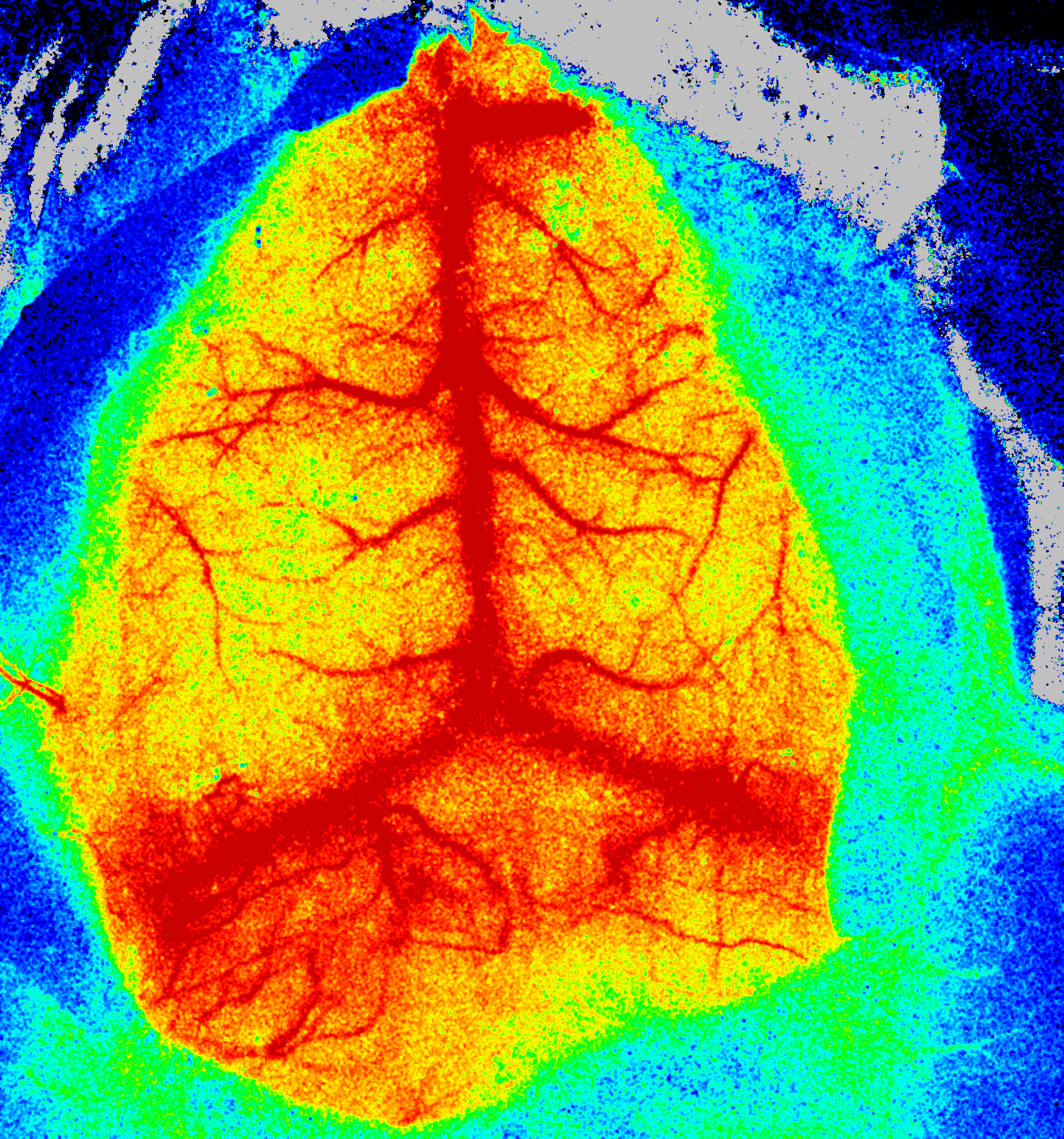

Supplement: Supplementary file 10 — Appendix Figure Source Data [file 44321_2025_206_MOESM10_ESM.zip › Appendix Figures Source Data/Appendix Fig. S1/S1-E-F/WT3.png]

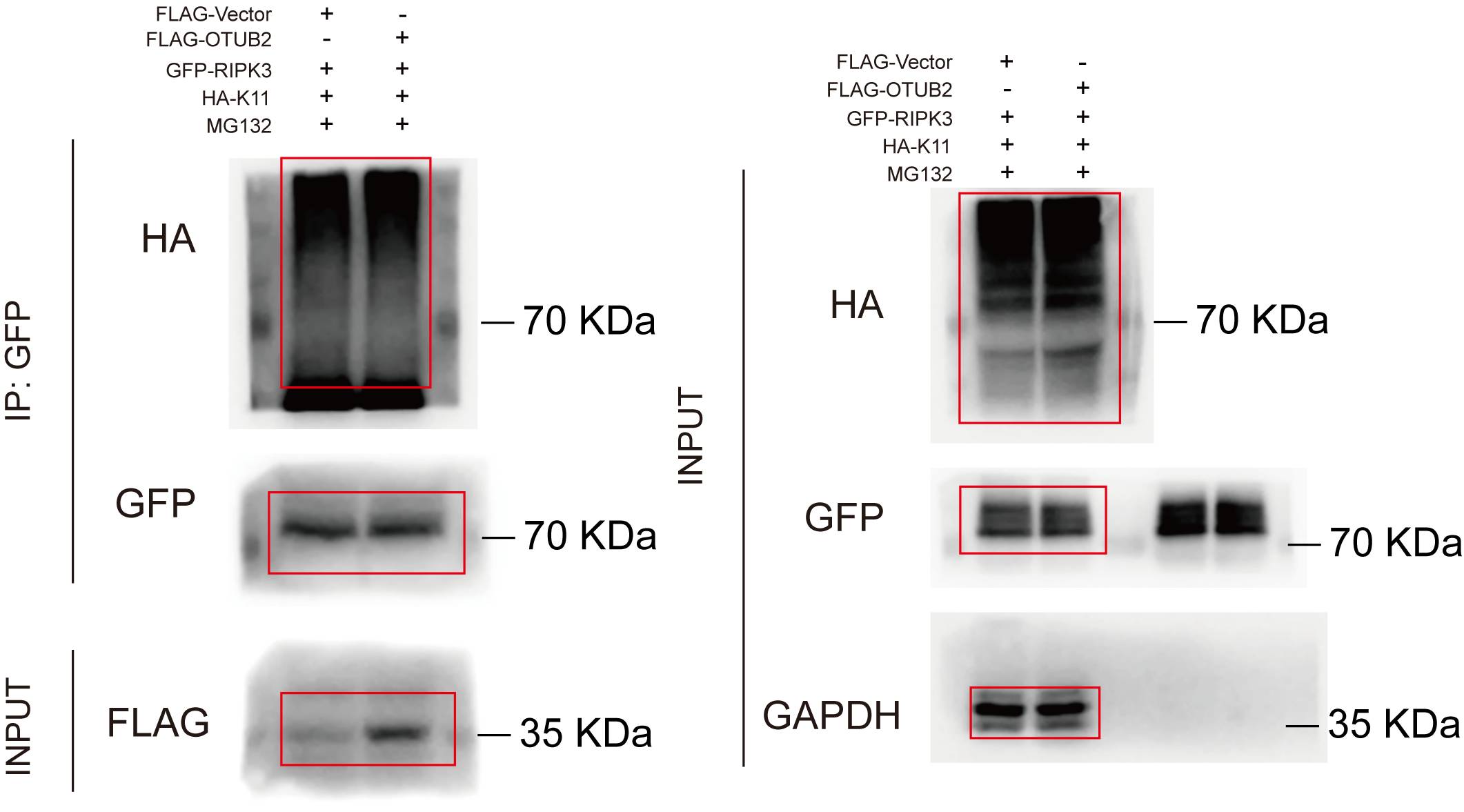

Supplement: Supplementary file 10 — Appendix Figure Source Data [file 44321_2025_206_MOESM10_ESM.zip › Appendix Figures Source Data/Appendix Fig. S10/S10-A.tif]

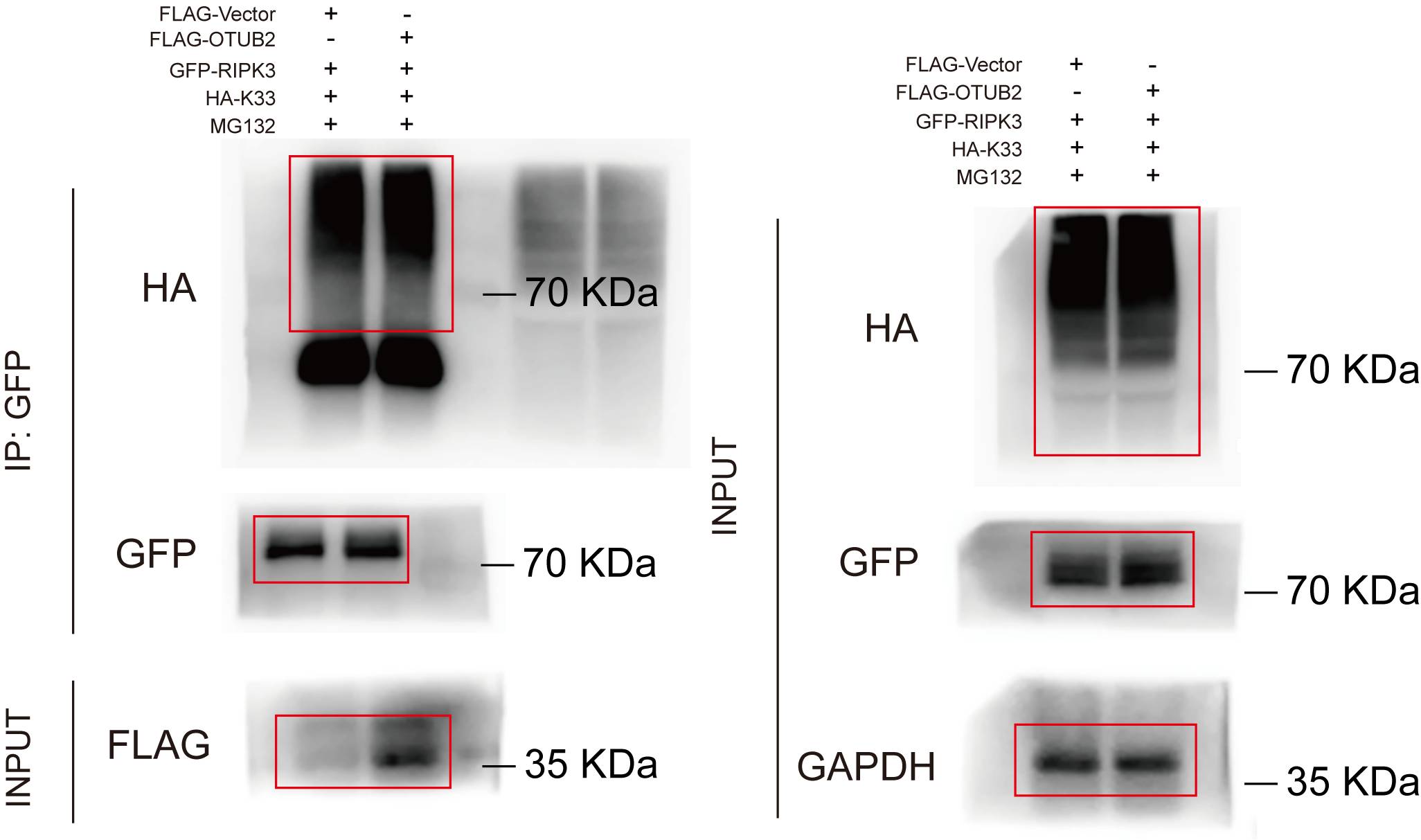

Supplement: Supplementary file 10 — Appendix Figure Source Data [file 44321_2025_206_MOESM10_ESM.zip › Appendix Figures Source Data/Appendix Fig. S10/S10-B.tif]

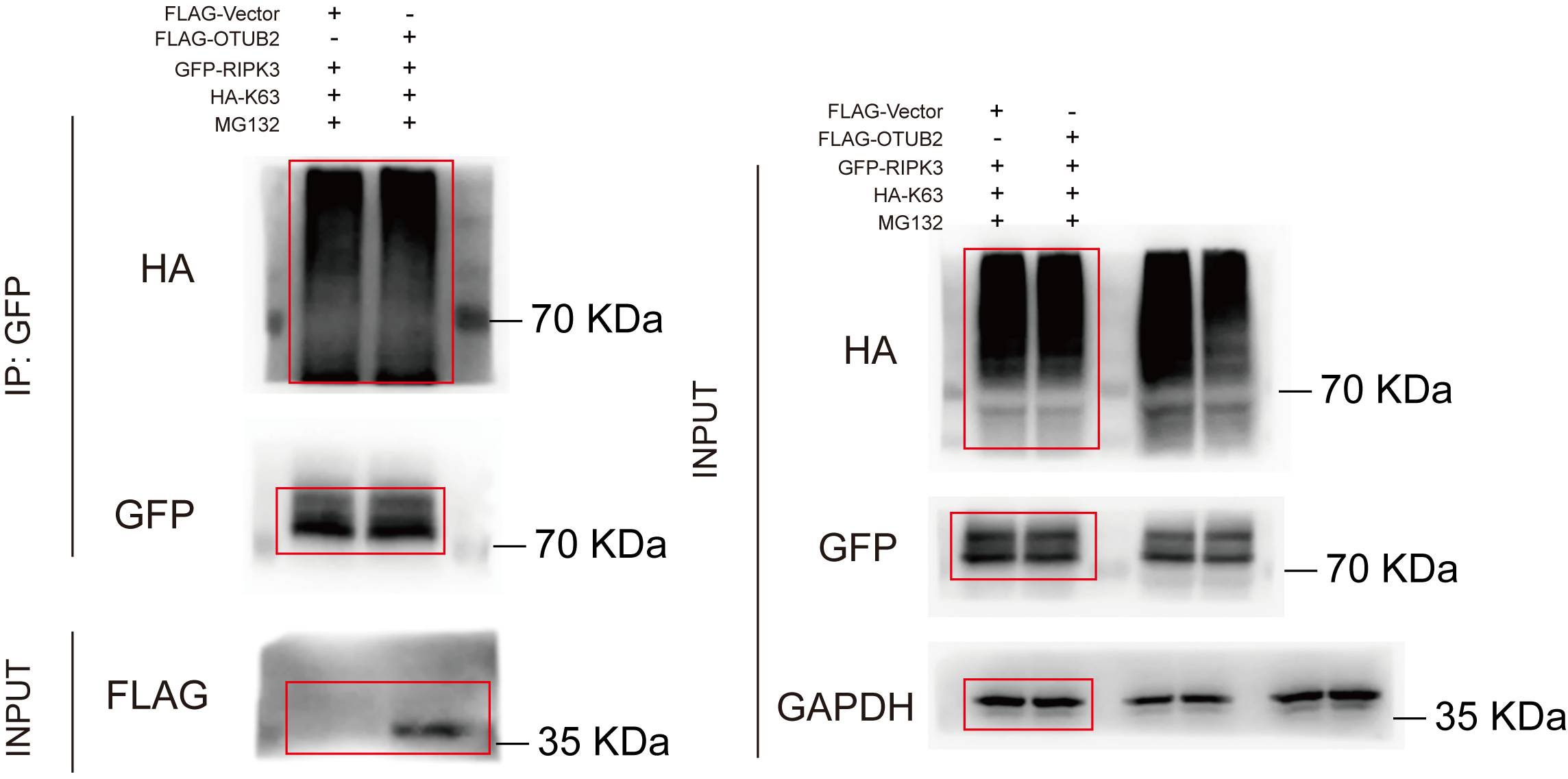

Supplement: Supplementary file 10 — Appendix Figure Source Data [file 44321_2025_206_MOESM10_ESM.zip › Appendix Figures Source Data/Appendix Fig. S10/S10-C.tif]

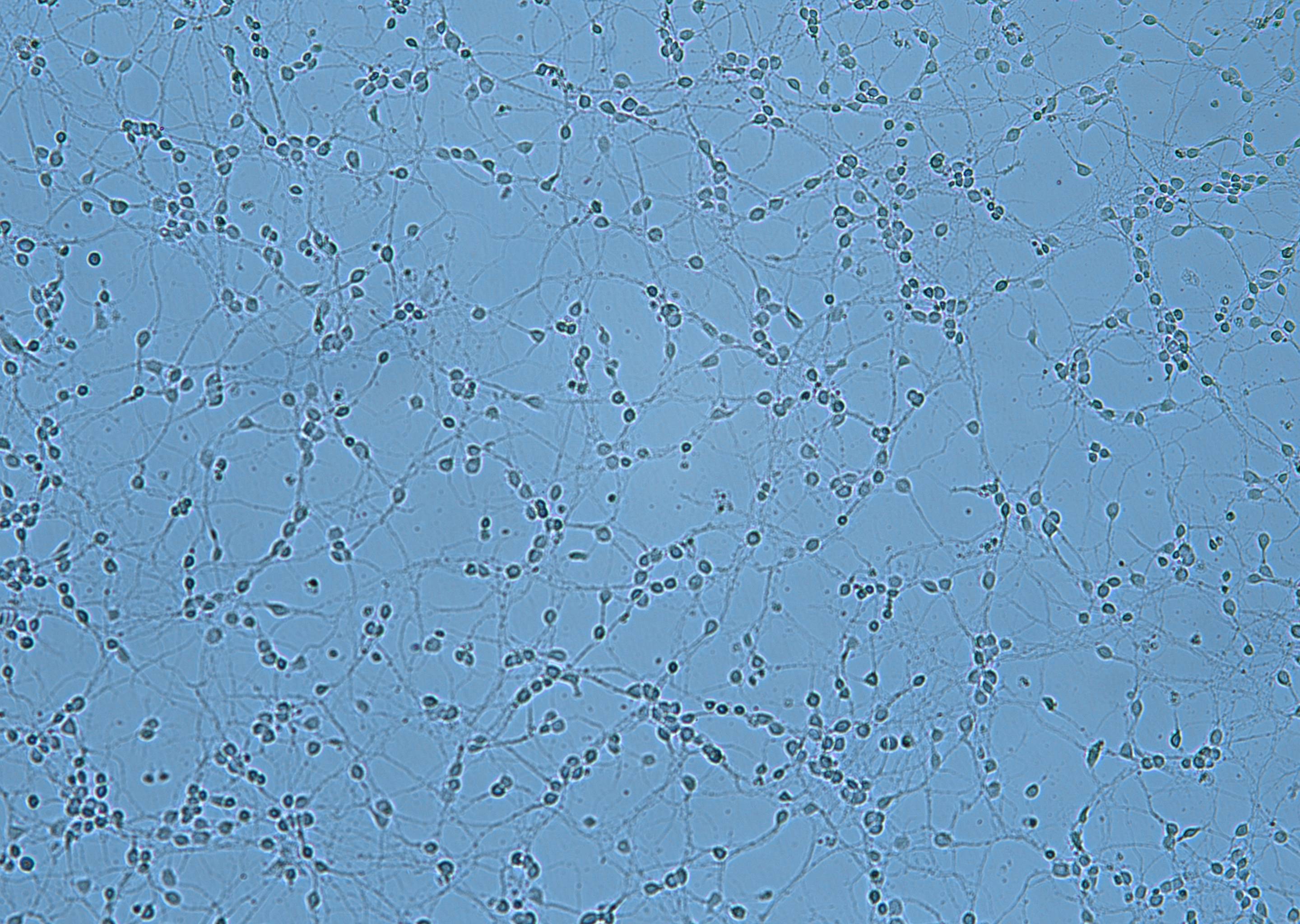

Supplement: Supplementary file 10 — Appendix Figure Source Data [file 44321_2025_206_MOESM10_ESM.zip › Appendix Figures Source Data/Appendix Fig. S11/S11-B-C/KO-Normoxia NEUN.tif]

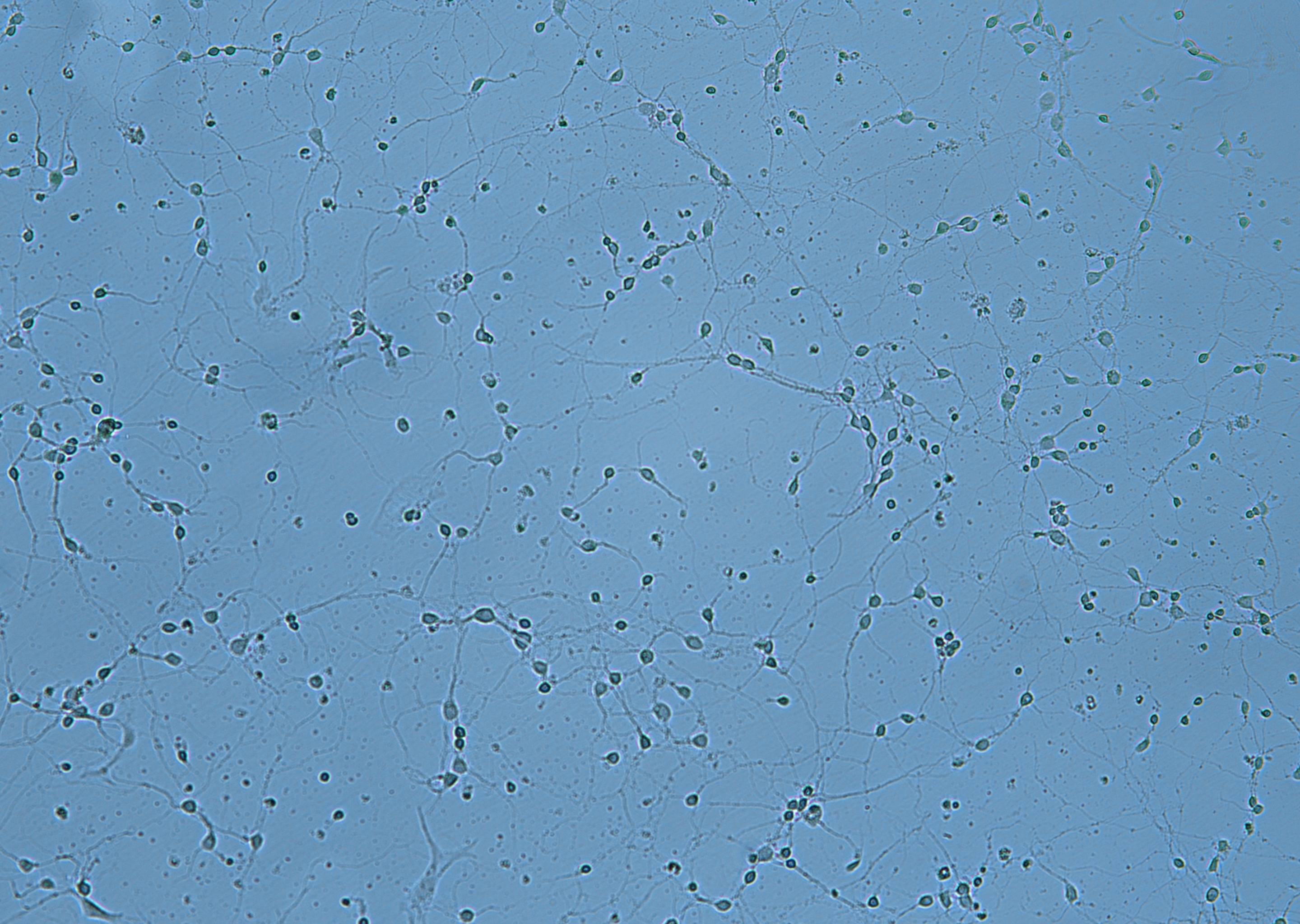

Supplement: Supplementary file 10 — Appendix Figure Source Data [file 44321_2025_206_MOESM10_ESM.zip › Appendix Figures Source Data/Appendix Fig. S11/S11-B-C/KO-OGD NEUN.tif]

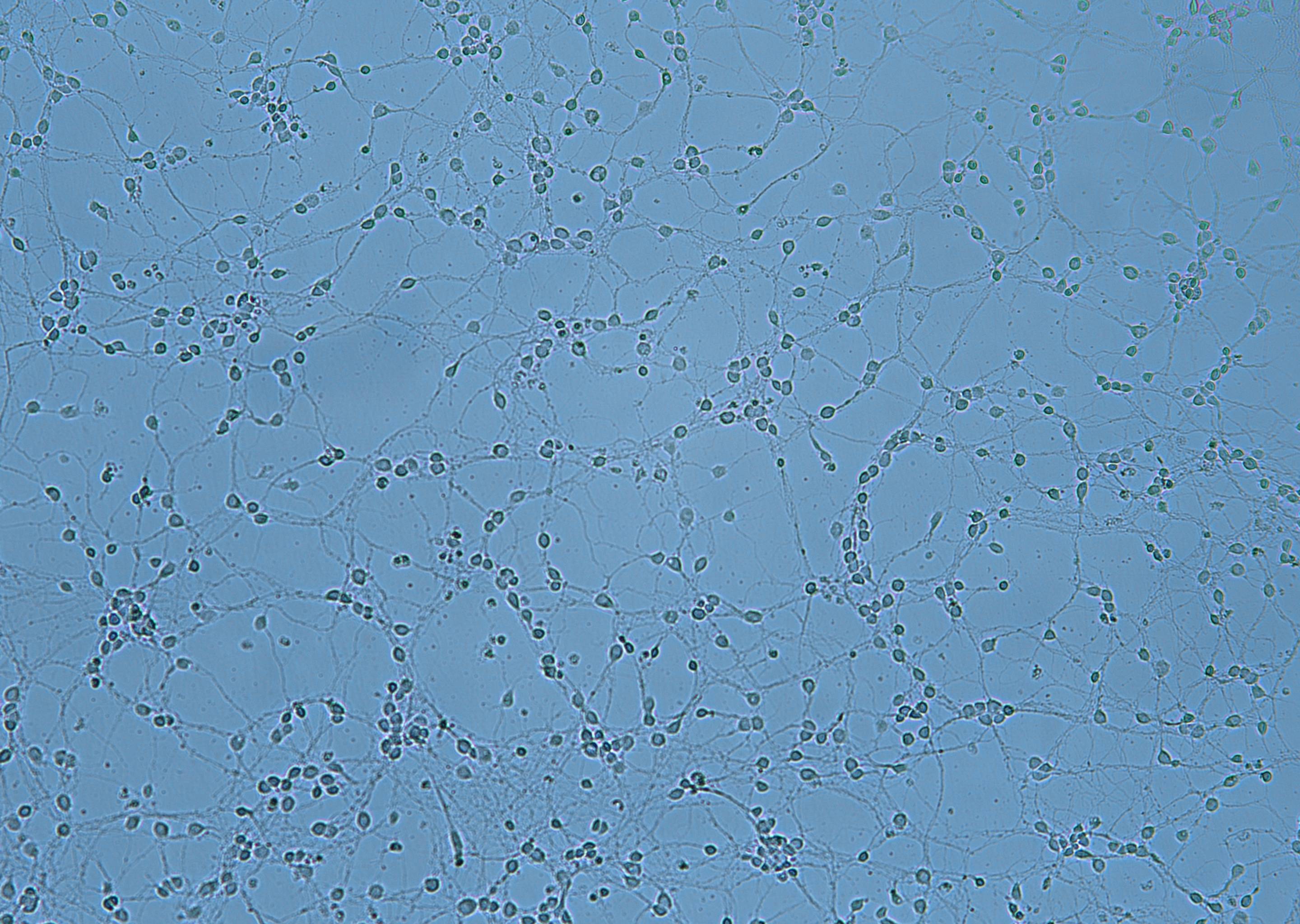

Supplement: Supplementary file 10 — Appendix Figure Source Data [file 44321_2025_206_MOESM10_ESM.zip › Appendix Figures Source Data/Appendix Fig. S11/S11-B-C/WT-Normoxia NEUN.tif]

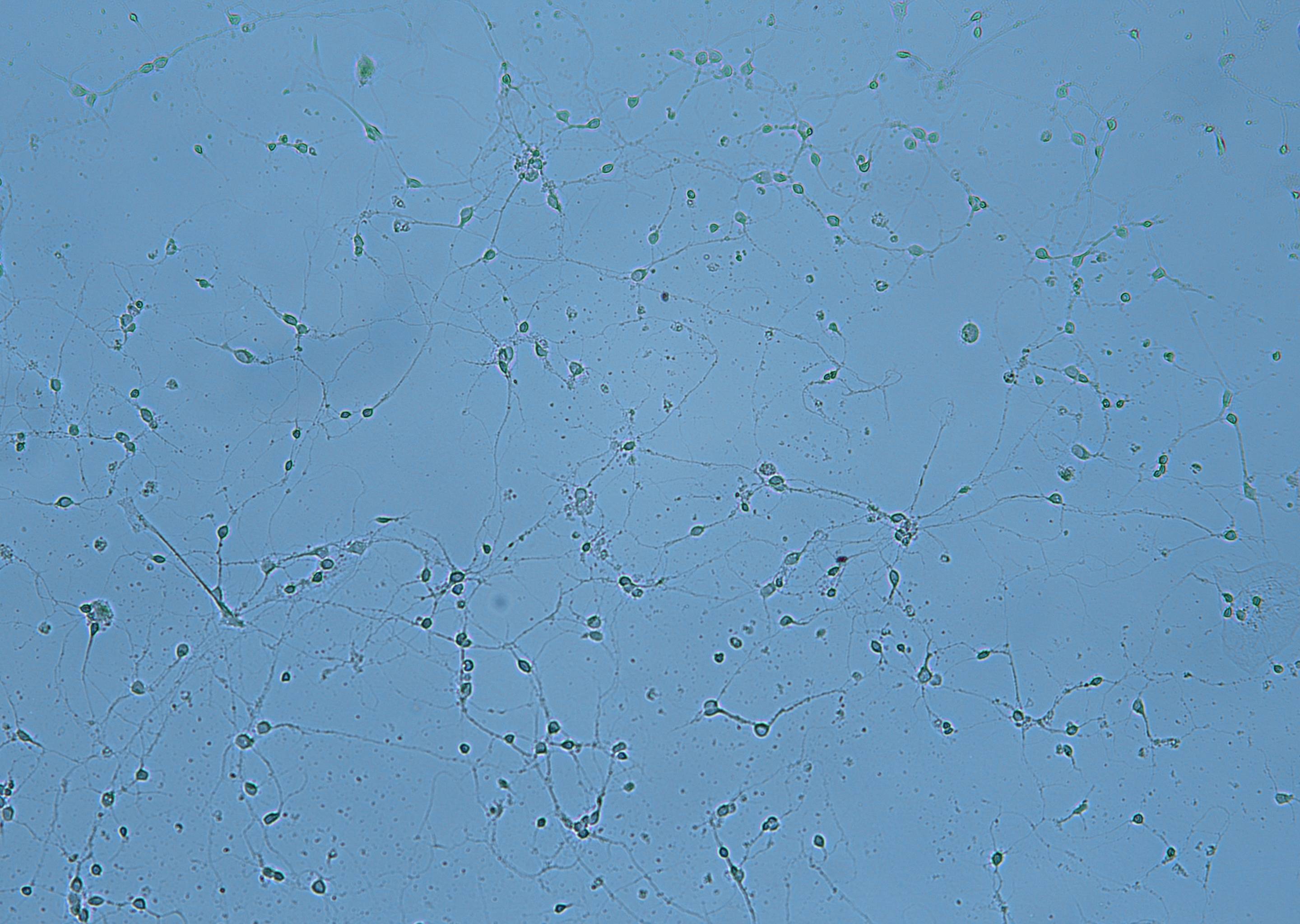

Supplement: Supplementary file 10 — Appendix Figure Source Data [file 44321_2025_206_MOESM10_ESM.zip › Appendix Figures Source Data/Appendix Fig. S11/S11-B-C/WT-OGD NEUN.tif]

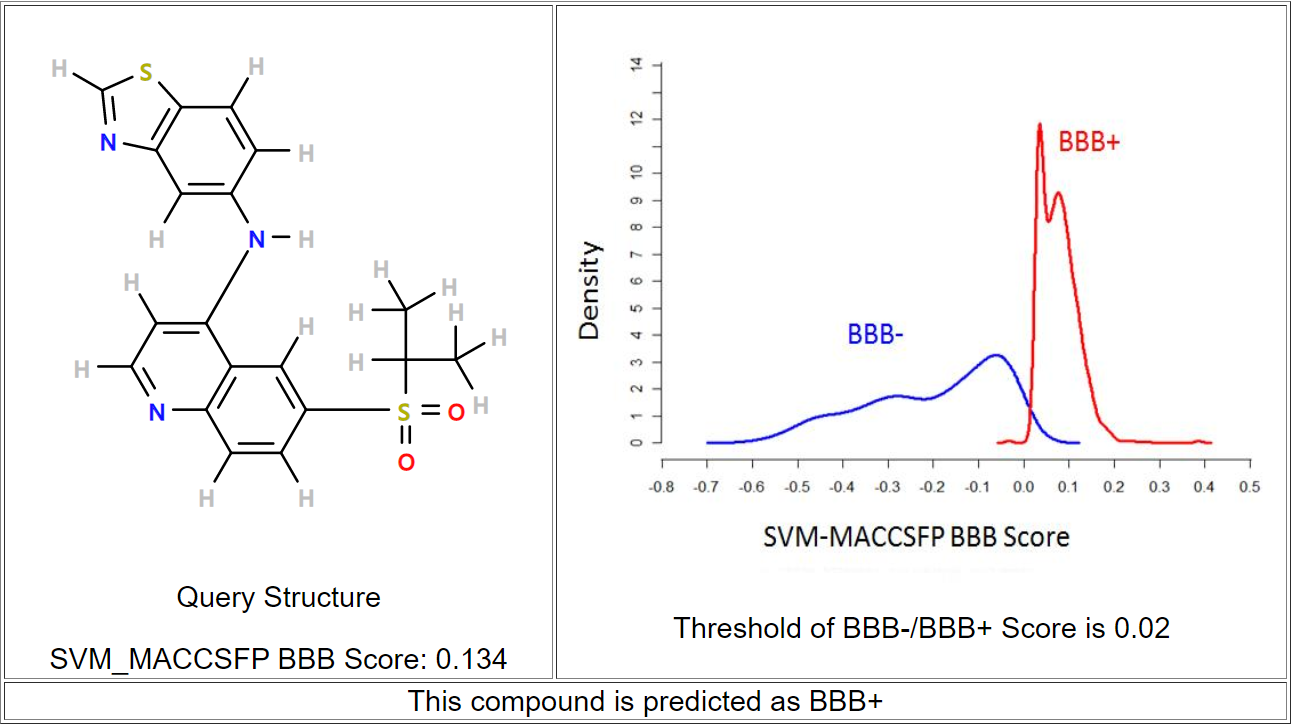

Supplement: Supplementary file 10 — Appendix Figure Source Data [file 44321_2025_206_MOESM10_ESM.zip › Appendix Figures Source Data/Appendix Fig. S12/S12-B.png]

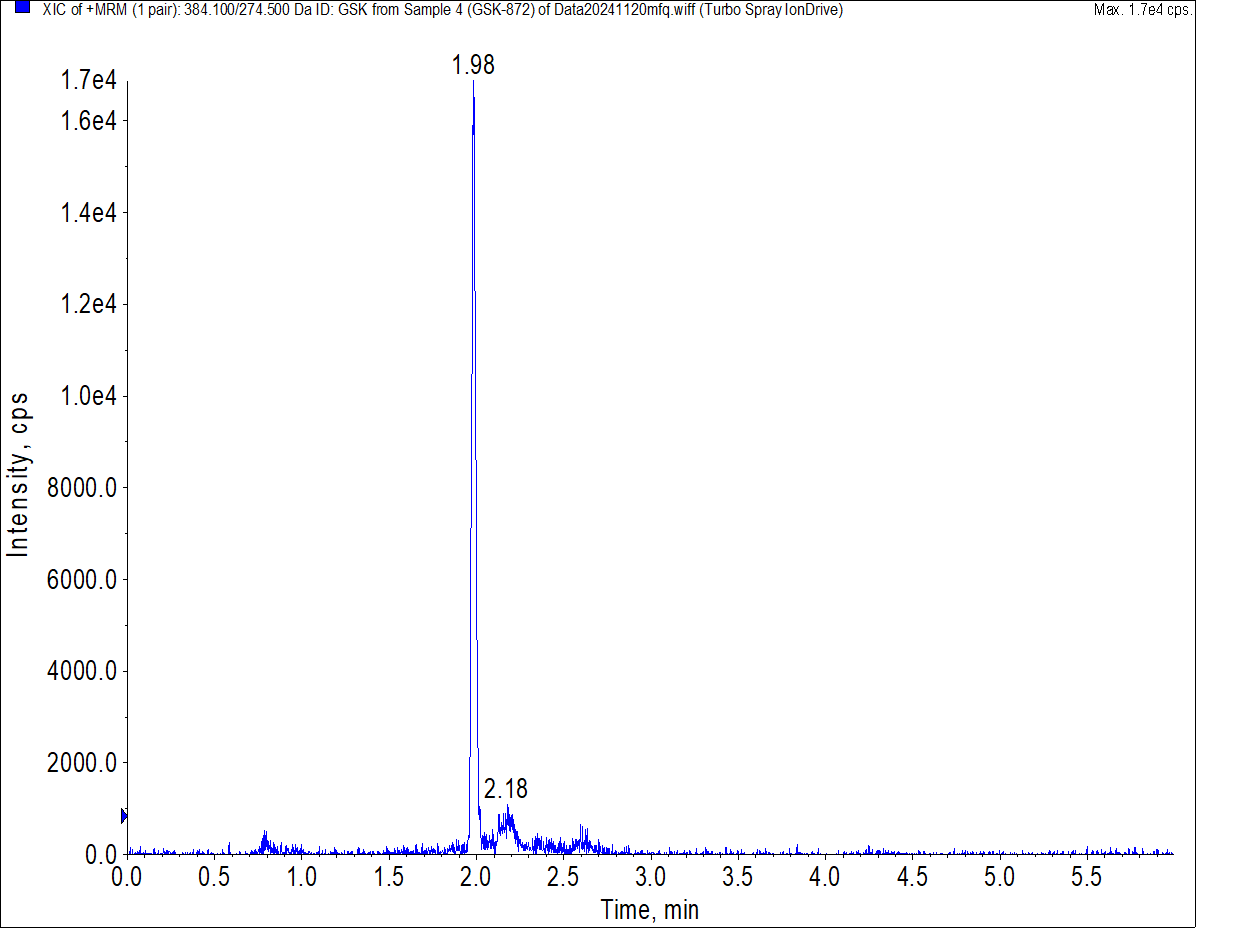

Supplement: Supplementary file 10 — Appendix Figure Source Data [file 44321_2025_206_MOESM10_ESM.zip › Appendix Figures Source Data/Appendix Fig. S12/S12-D/GSK-872-1.png]
