# Supplementary material for: Deubiquitination of RIPK3 by OTUB2 potentiates neuronal necroptosis after ischemic stroke
Source: EMBO Mol Med. 2025 Feb 28;17(4):679–95. doi: 10.1038/s44321-025-00206-6 (PMC11982199; doi:10.1038/s44321-025-00206-6)
Supplement: Supplementary file 10 — Appendix Figure Source Data [file 44321_2025_206_MOESM10_ESM.zip › Appendix Figures Source Data/Appendix Fig. S1/S1-G-H/WT-pO2.pdf]

患者 4	患者特征
记录 Auto Connected	日期/时间 2024/9/27 15:26:44

常规分析报告

记录资料

医生:　1, 1
助手:　1, 1
相关人员:　1, 1

曲线

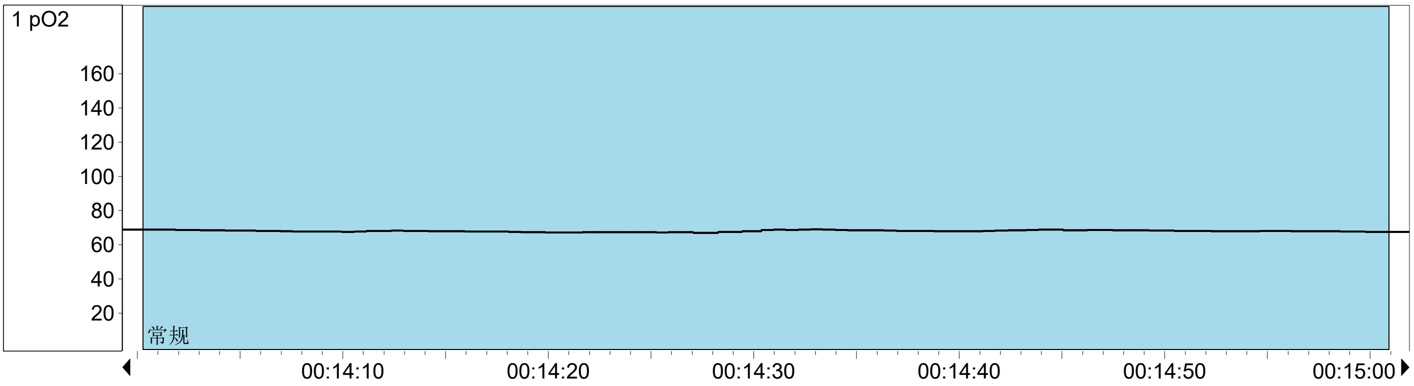

计算

项目		1 pO2
平均值	(单位)	68.05
SD值	(单位)	0.48
SE值	(单位)	0.01
最大值	(单位)	69.03
最小值	(单位)	67.02

项目		1 pO2
首值	(单位)	68.85
末值	(单位)	67.57
首值到末值的变化百分比	(%)	-1.86
曲线下面积	(单位*sec.)	4123.44
斜率	(单位/sec.)	-0.02

项目		1 pO2
持续时间	(sec.)	60.57
相对开始时刻	(时:分:ss.fr)	00:14:00.28
相对结束时刻	(时:分:ss.fr)	00:15:00.91
