# Supplementary figures and images for: Deubiquitination of RIPK3 by OTUB2 potentiates neuronal necroptosis after ischemic stroke (part 3 of 3)
Source: EMBO Mol Med. 2025 Feb 28;17(4):679–95. doi: 10.1038/s44321-025-00206-6 (PMC11982199; doi:10.1038/s44321-025-00206-6)

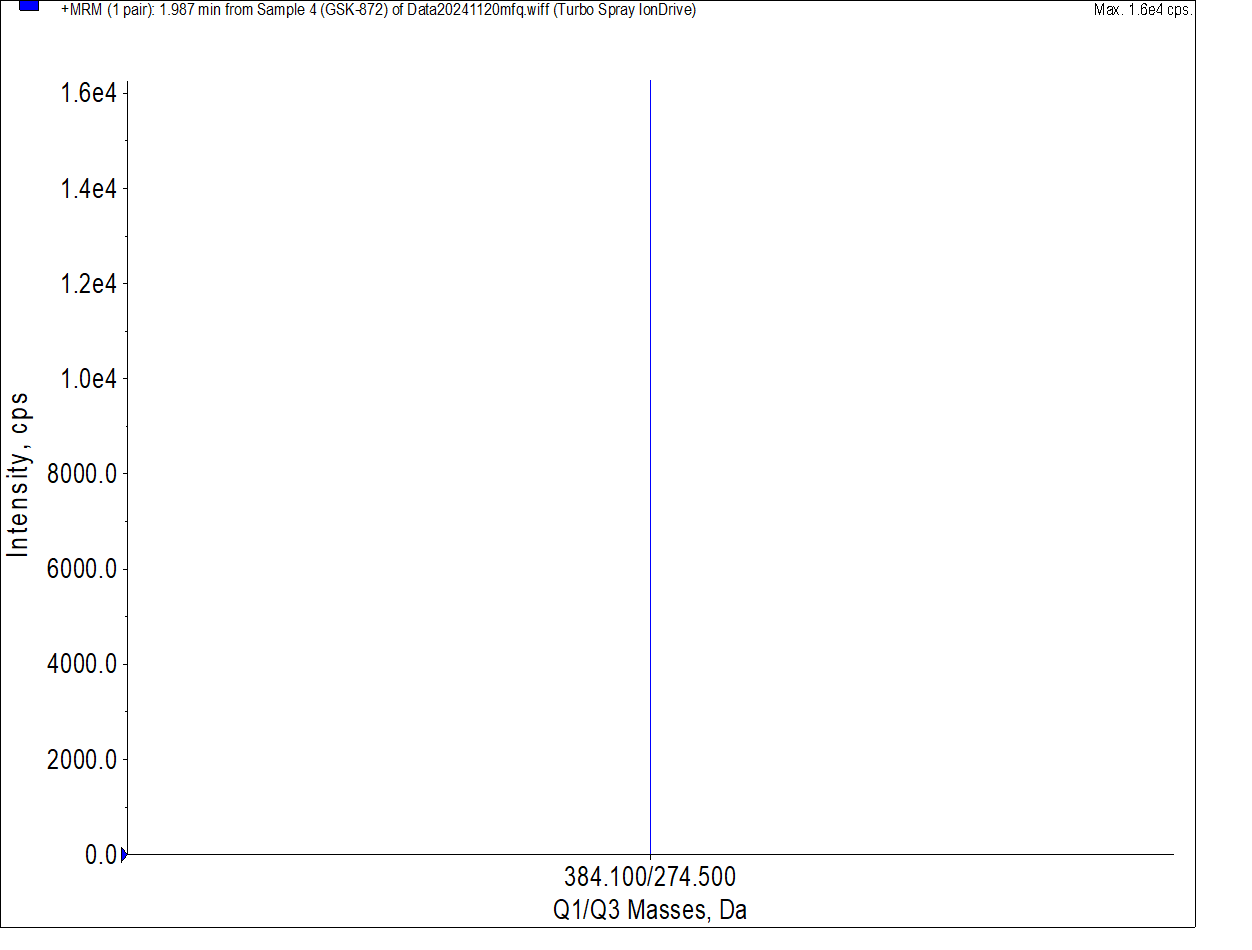

Supplement: Supplementary file 10 — Appendix Figure Source Data [file 44321_2025_206_MOESM10_ESM.zip › Appendix Figures Source Data/Appendix Fig. S12/S12-D/GSK-872-2.png]

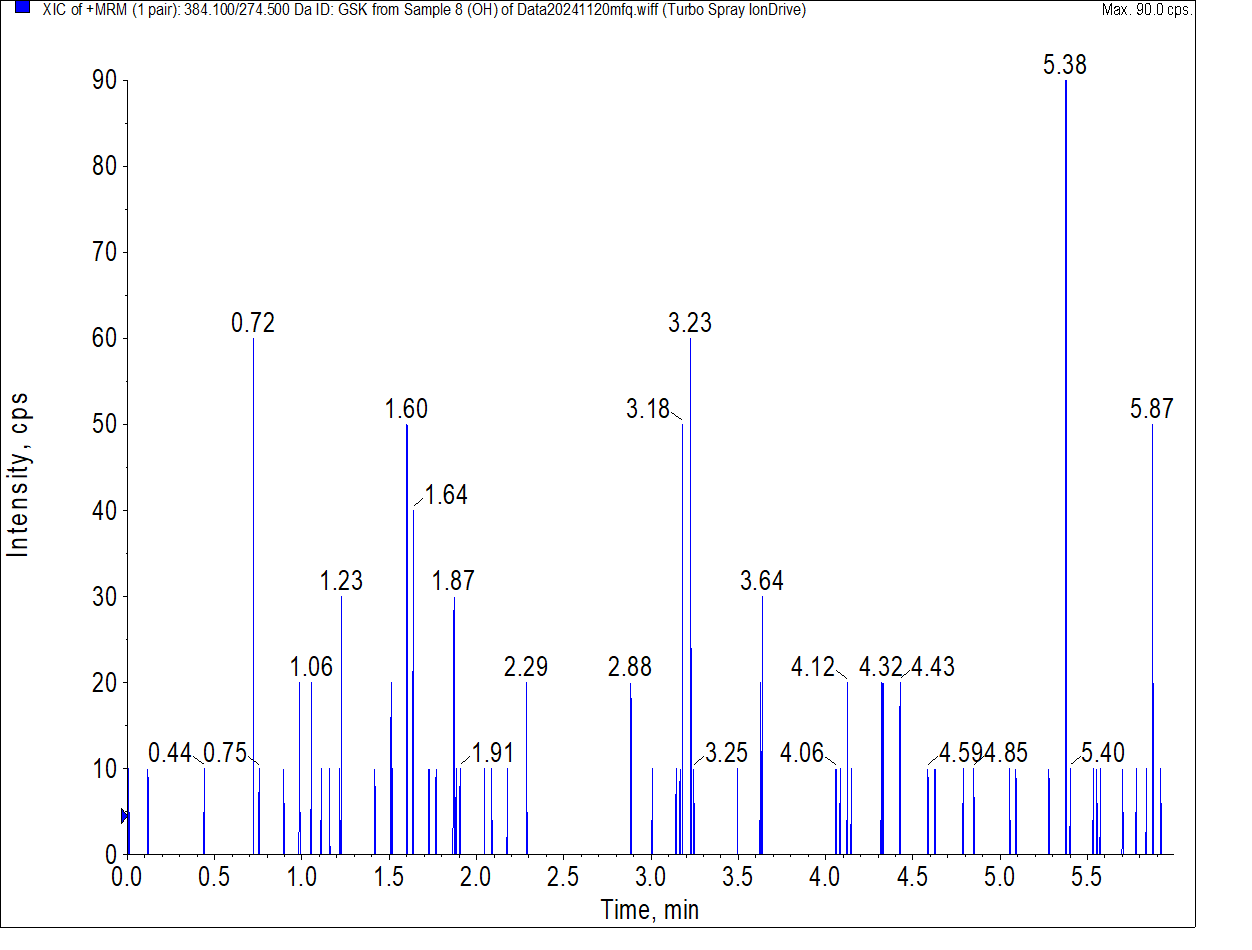

Supplement: Supplementary file 10 — Appendix Figure Source Data [file 44321_2025_206_MOESM10_ESM.zip › Appendix Figures Source Data/Appendix Fig. S12/S12-D/MCAO-1H-CON-1.png]

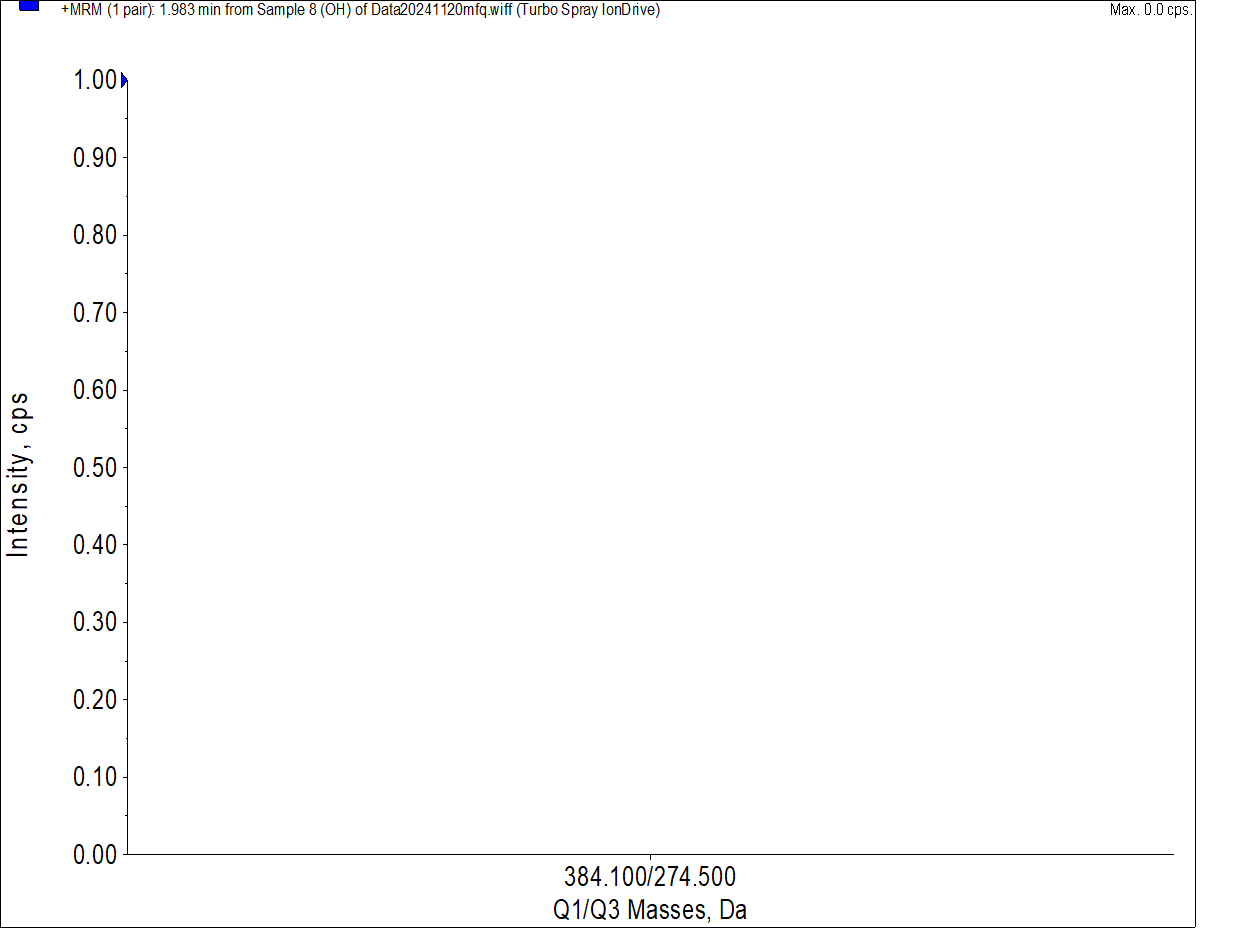

Supplement: Supplementary file 10 — Appendix Figure Source Data [file 44321_2025_206_MOESM10_ESM.zip › Appendix Figures Source Data/Appendix Fig. S12/S12-D/MCAO-1H-CON-2.png]

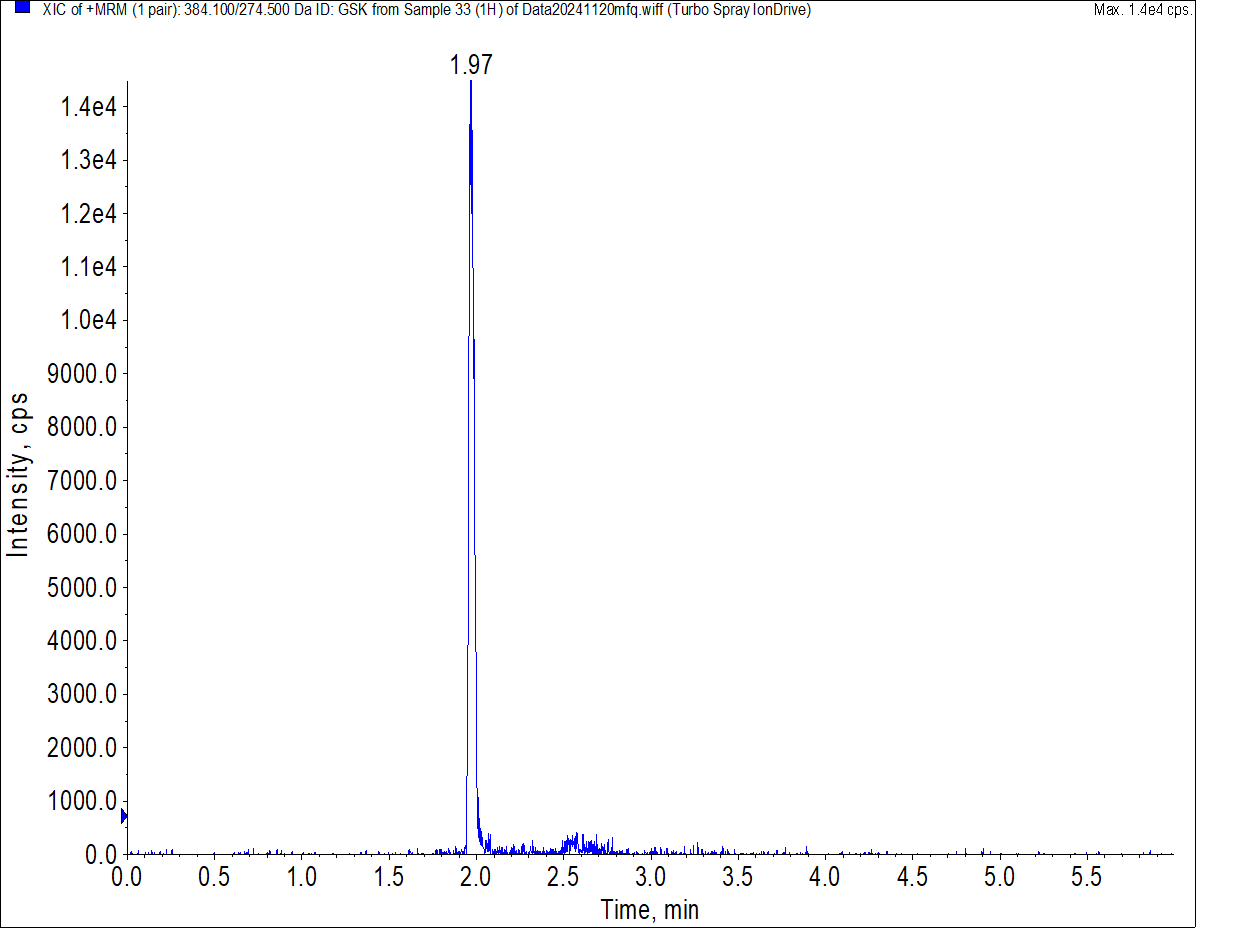

Supplement: Supplementary file 10 — Appendix Figure Source Data [file 44321_2025_206_MOESM10_ESM.zip › Appendix Figures Source Data/Appendix Fig. S12/S12-D/MCAO-1H-GSK-872-1.png]

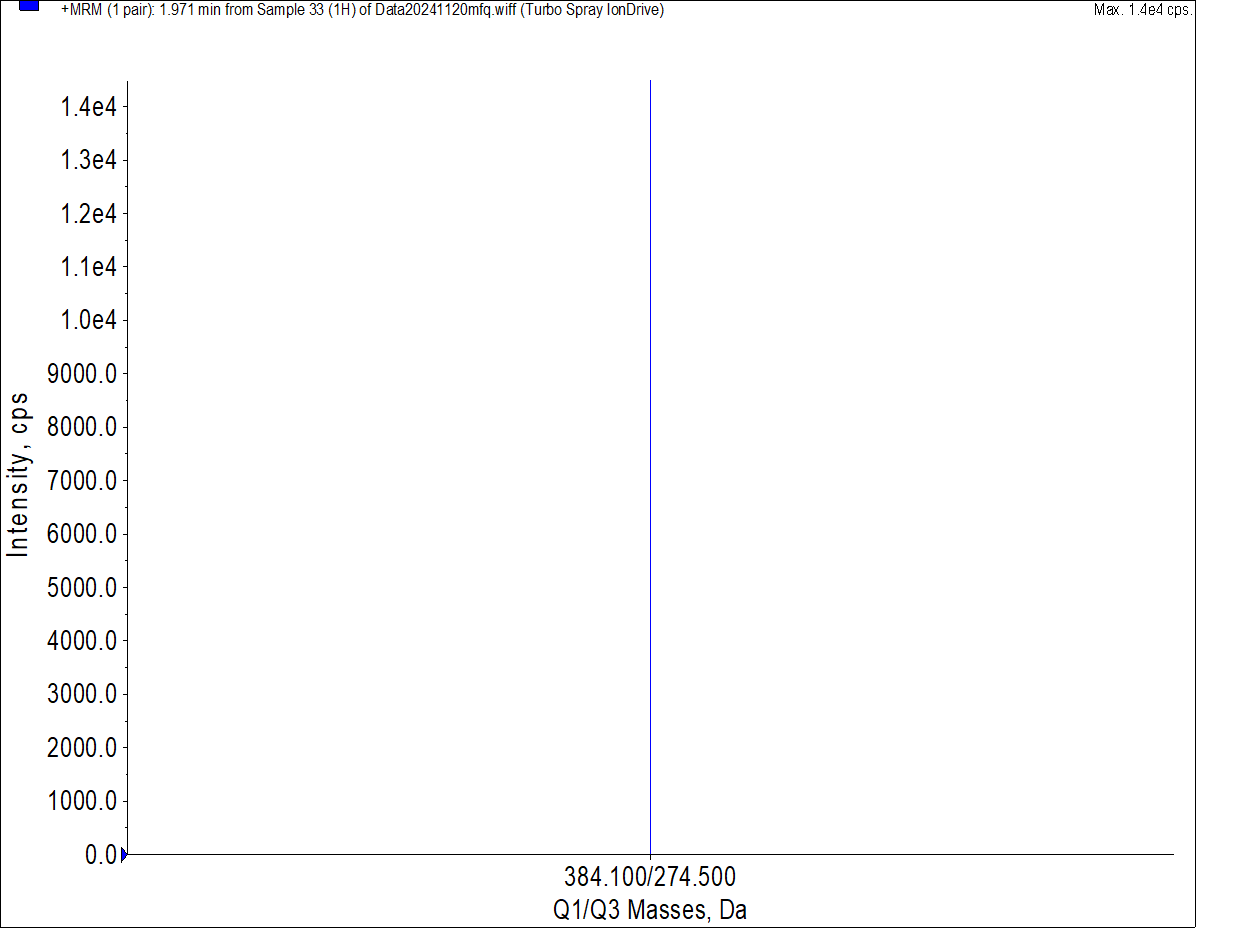

Supplement: Supplementary file 10 — Appendix Figure Source Data [file 44321_2025_206_MOESM10_ESM.zip › Appendix Figures Source Data/Appendix Fig. S12/S12-D/MCAO-1H-GSK-872-2.png]

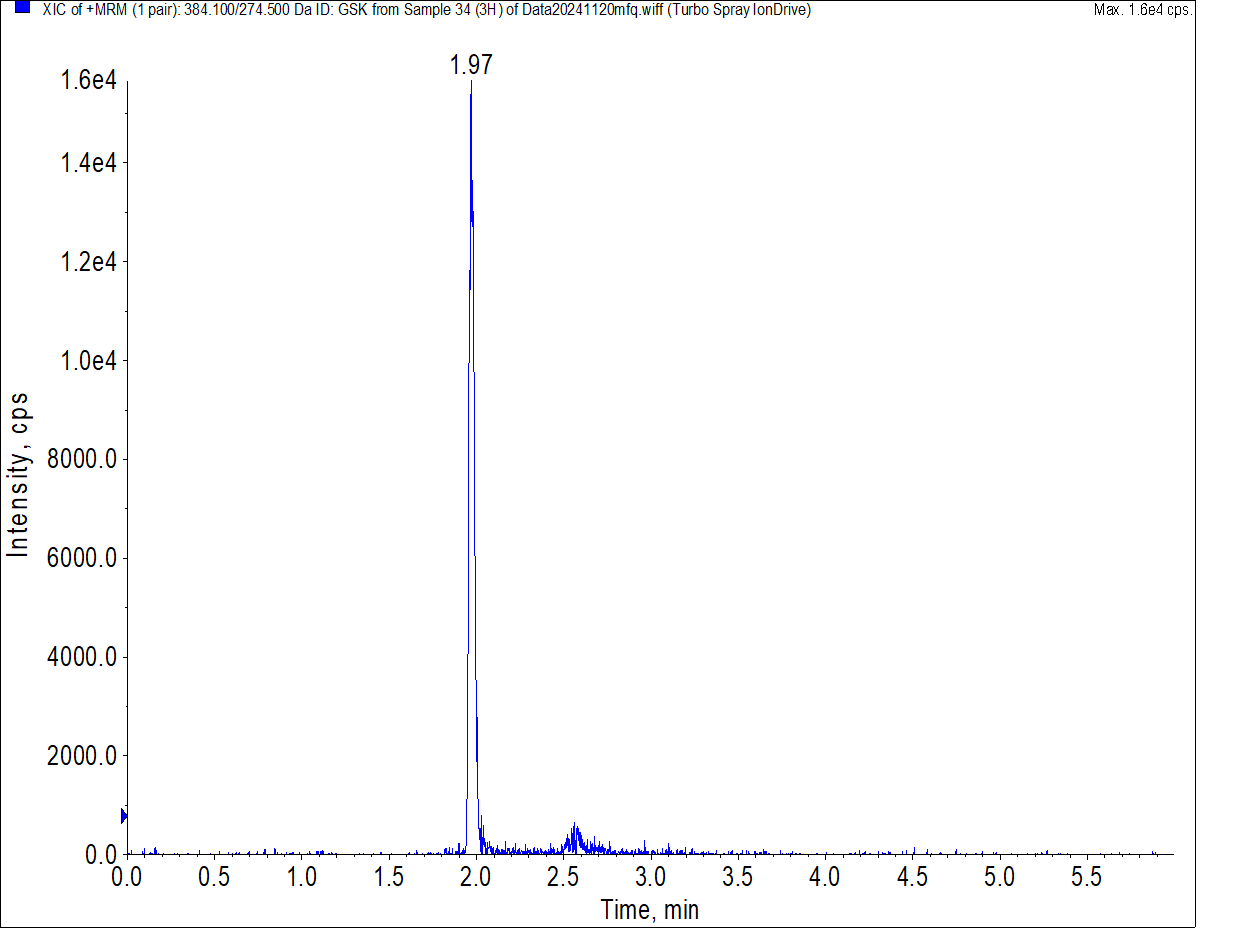

Supplement: Supplementary file 10 — Appendix Figure Source Data [file 44321_2025_206_MOESM10_ESM.zip › Appendix Figures Source Data/Appendix Fig. S12/S12-D/MCAO-3H-GSK-872-1.png]

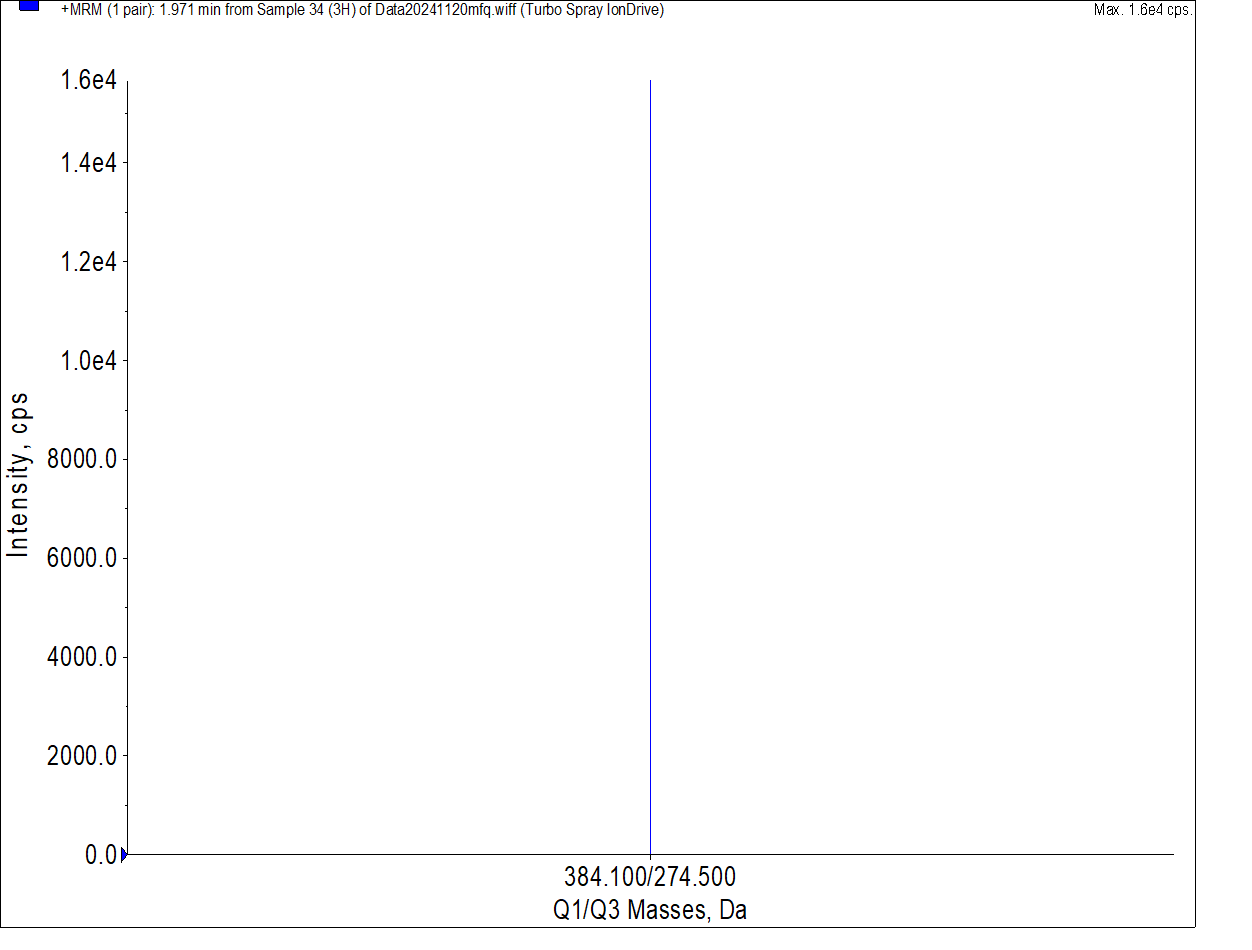

Supplement: Supplementary file 10 — Appendix Figure Source Data [file 44321_2025_206_MOESM10_ESM.zip › Appendix Figures Source Data/Appendix Fig. S12/S12-D/MCAO-3H-GSK-872-2.png]

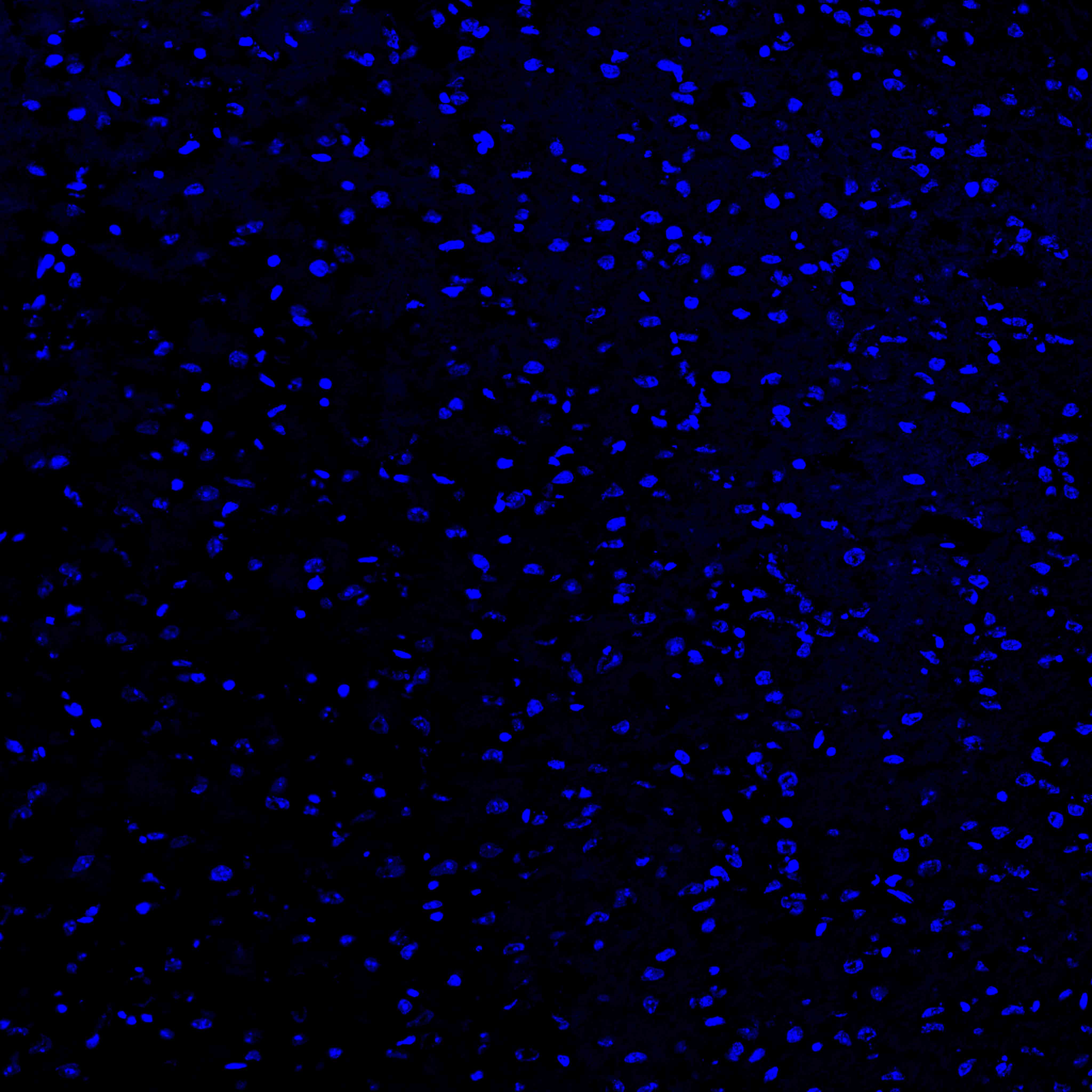

Supplement: Supplementary file 10 — Appendix Figure Source Data [file 44321_2025_206_MOESM10_ESM.zip › Appendix Figures Source Data/Appendix Fig. S13/S13-A/KO-MCAO-CON-DAPI.tif]

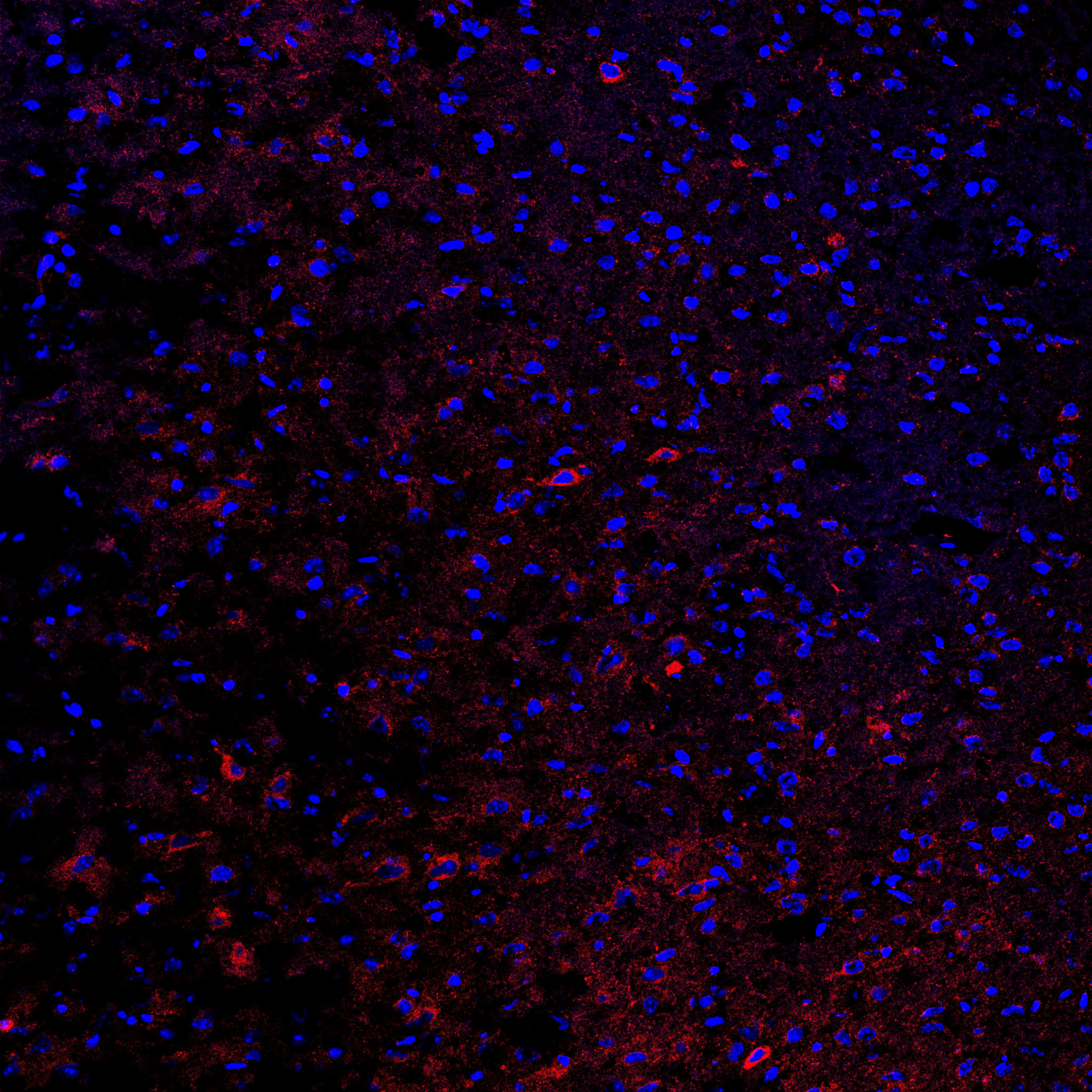

Supplement: Supplementary file 10 — Appendix Figure Source Data [file 44321_2025_206_MOESM10_ESM.zip › Appendix Figures Source Data/Appendix Fig. S13/S13-A/KO-MCAO-CON-MERGE.tif]

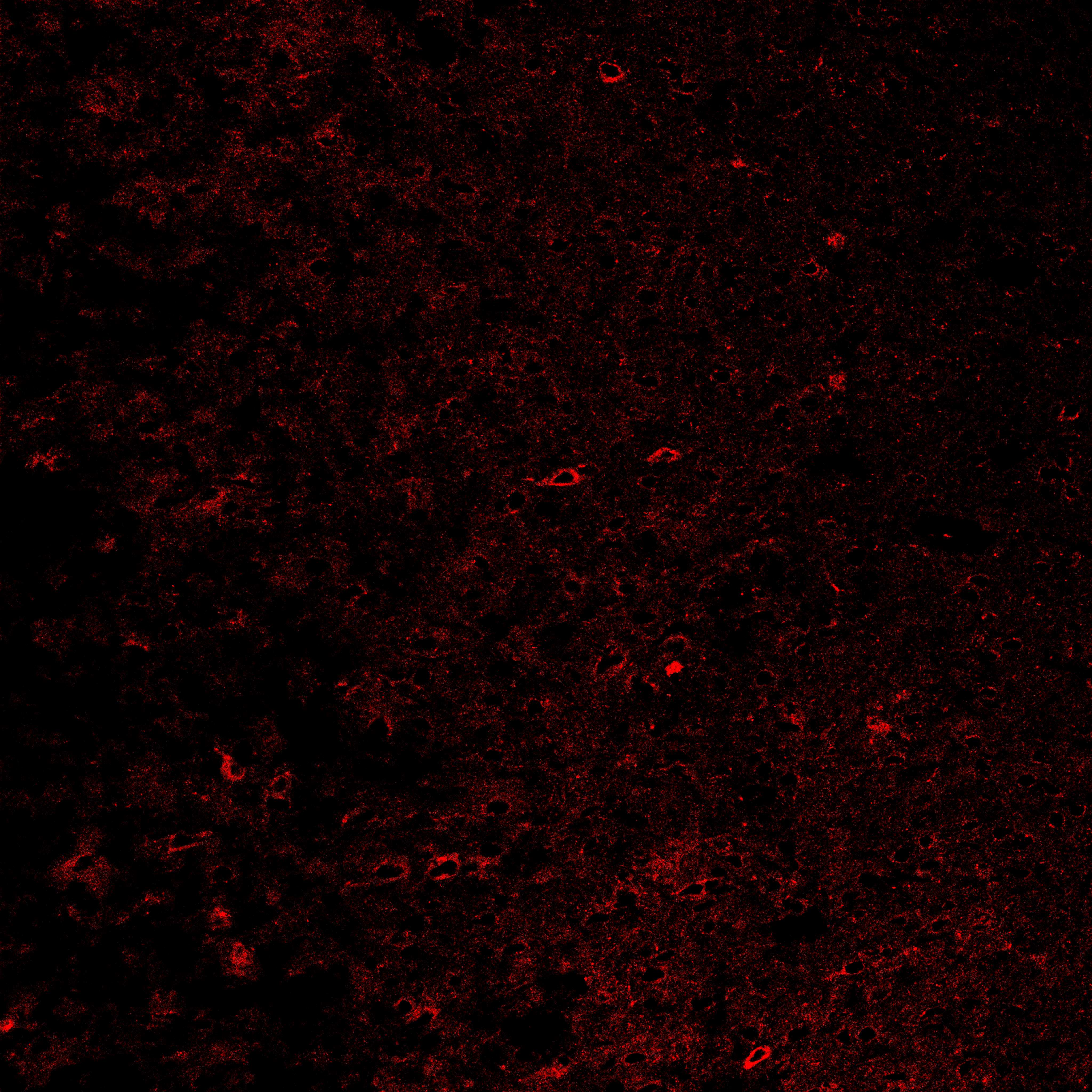

Supplement: Supplementary file 10 — Appendix Figure Source Data [file 44321_2025_206_MOESM10_ESM.zip › Appendix Figures Source Data/Appendix Fig. S13/S13-A/KO-MCAO-CON-P-RIPK3.tif]

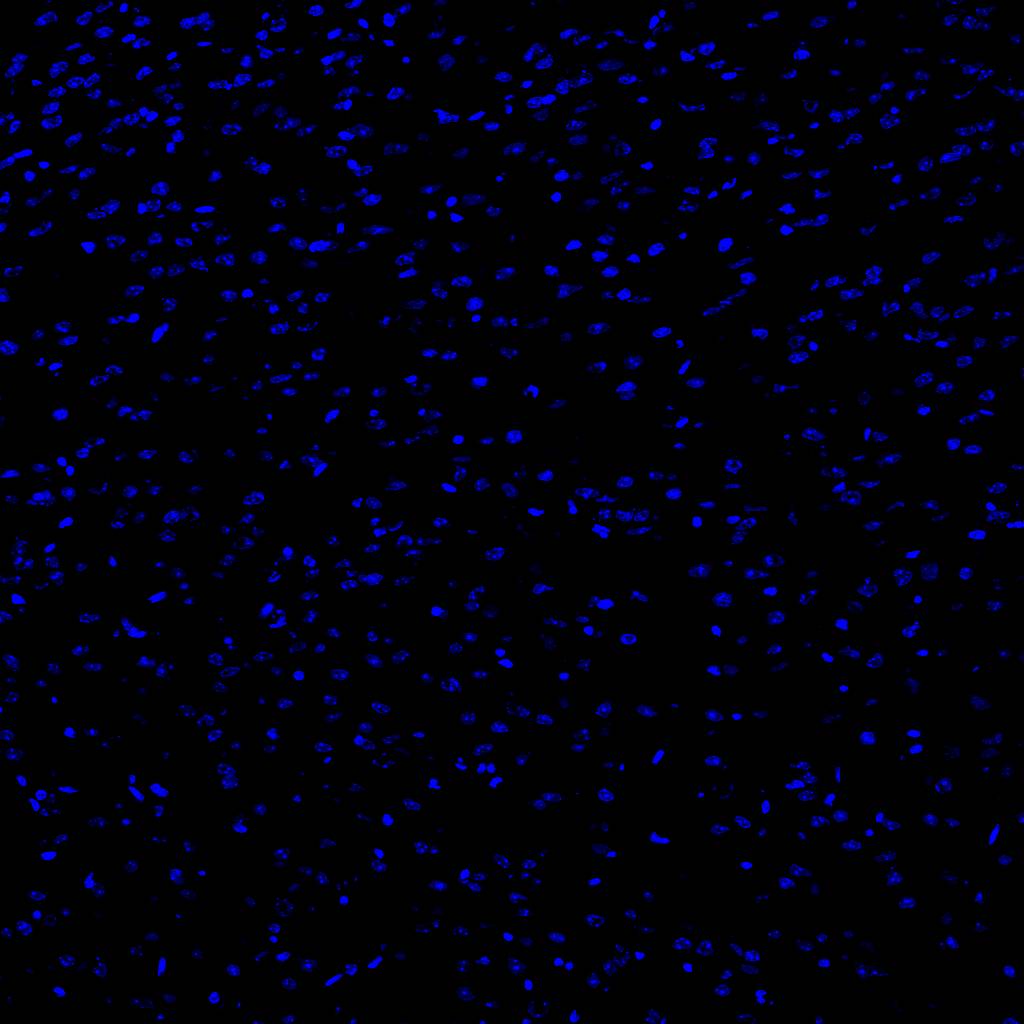

Supplement: Supplementary file 10 — Appendix Figure Source Data [file 44321_2025_206_MOESM10_ESM.zip › Appendix Figures Source Data/Appendix Fig. S13/S13-A/KO-MCAO-GSK-872-DAPI.tif]

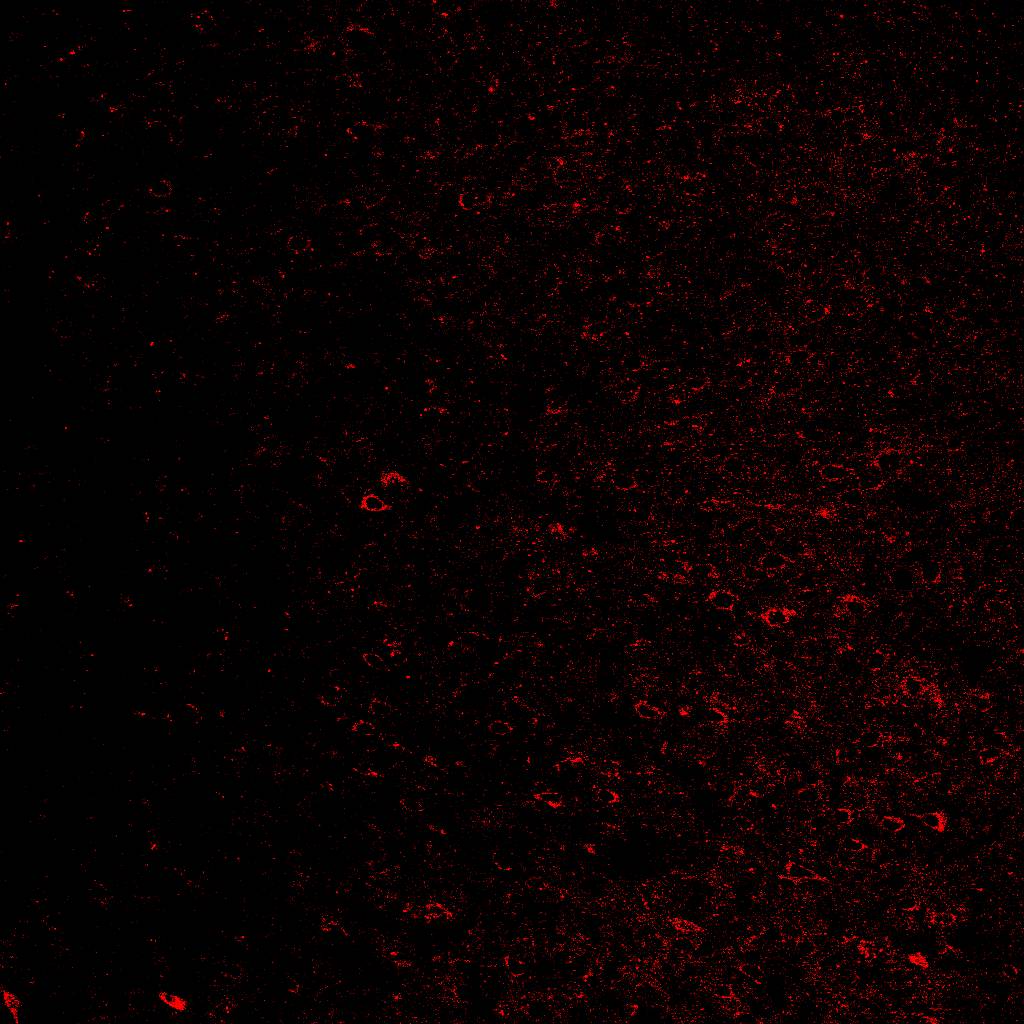

Supplement: Supplementary file 10 — Appendix Figure Source Data [file 44321_2025_206_MOESM10_ESM.zip › Appendix Figures Source Data/Appendix Fig. S13/S13-A/KO-MCAO-GSK-872-P-RIPK3.tif]

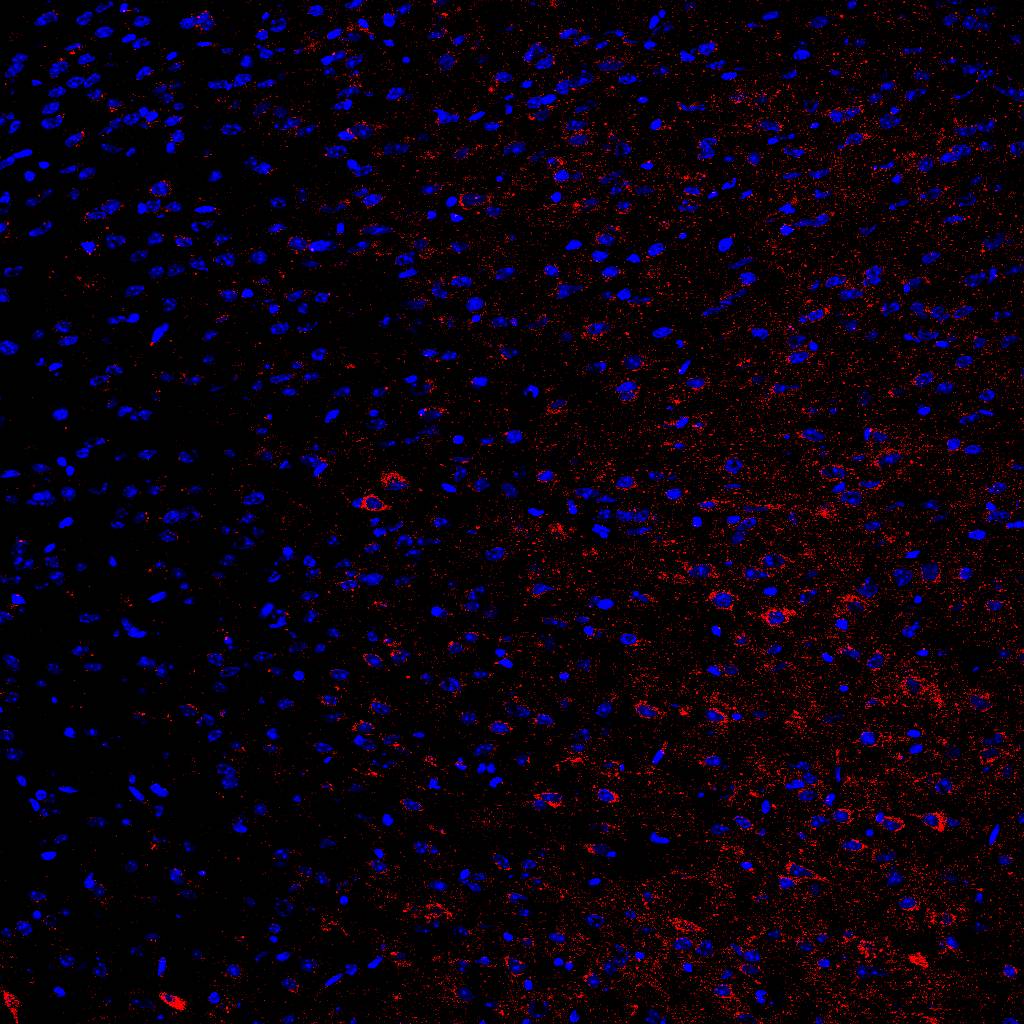

Supplement: Supplementary file 10 — Appendix Figure Source Data [file 44321_2025_206_MOESM10_ESM.zip › Appendix Figures Source Data/Appendix Fig. S13/S13-A/KO-MCAO-GSK-873-MERGE.tif]

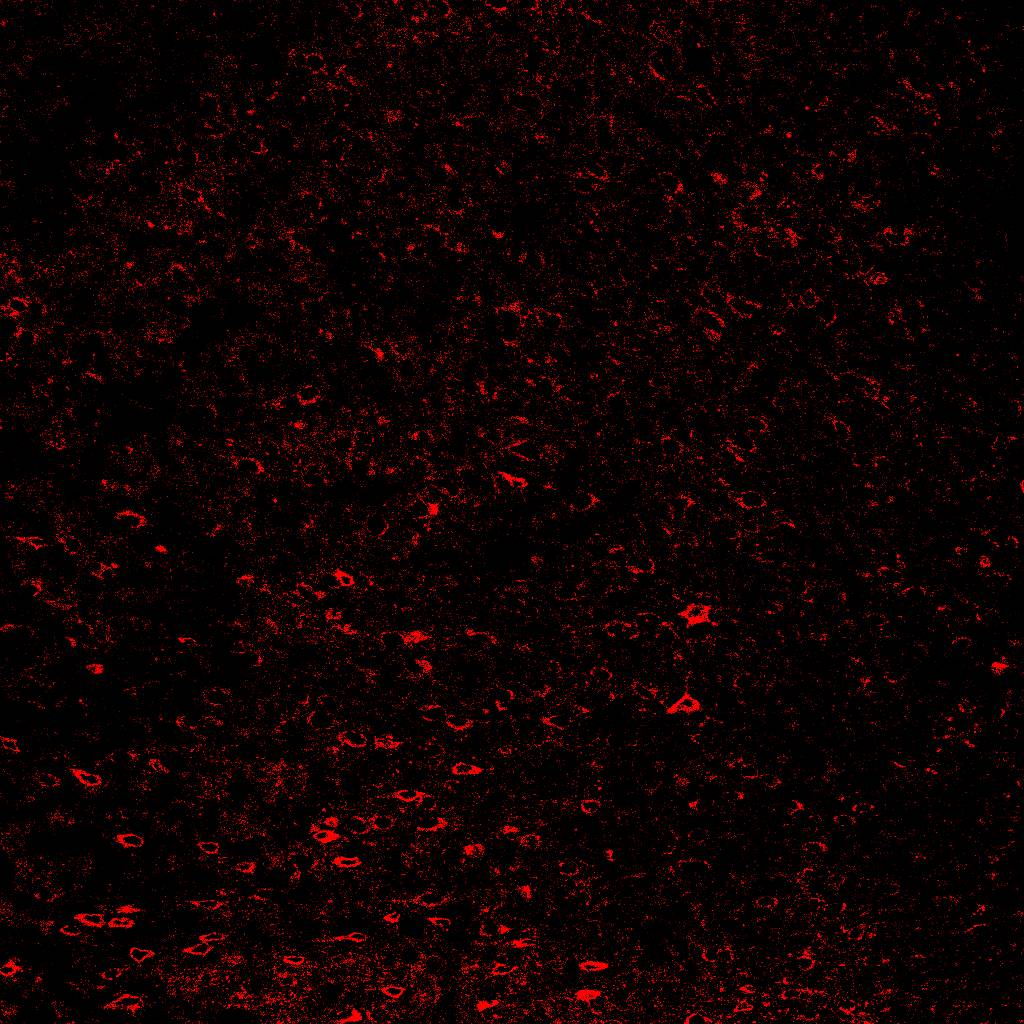

Supplement: Supplementary file 10 — Appendix Figure Source Data [file 44321_2025_206_MOESM10_ESM.zip › Appendix Figures Source Data/Appendix Fig. S13/S13-A/WT-MCAO -CON-P-RIPK3.tif]

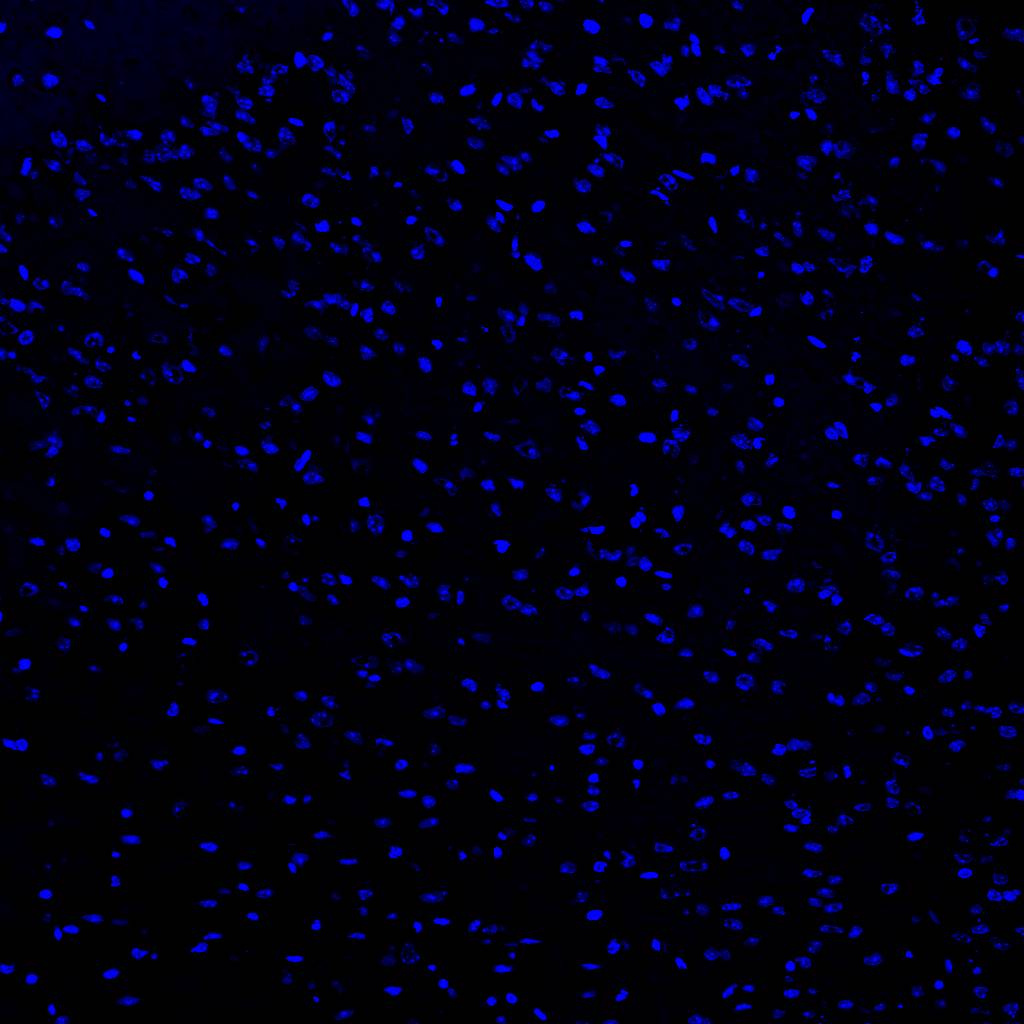

Supplement: Supplementary file 10 — Appendix Figure Source Data [file 44321_2025_206_MOESM10_ESM.zip › Appendix Figures Source Data/Appendix Fig. S13/S13-A/WT-MCAO-CON-DAPI.tif]

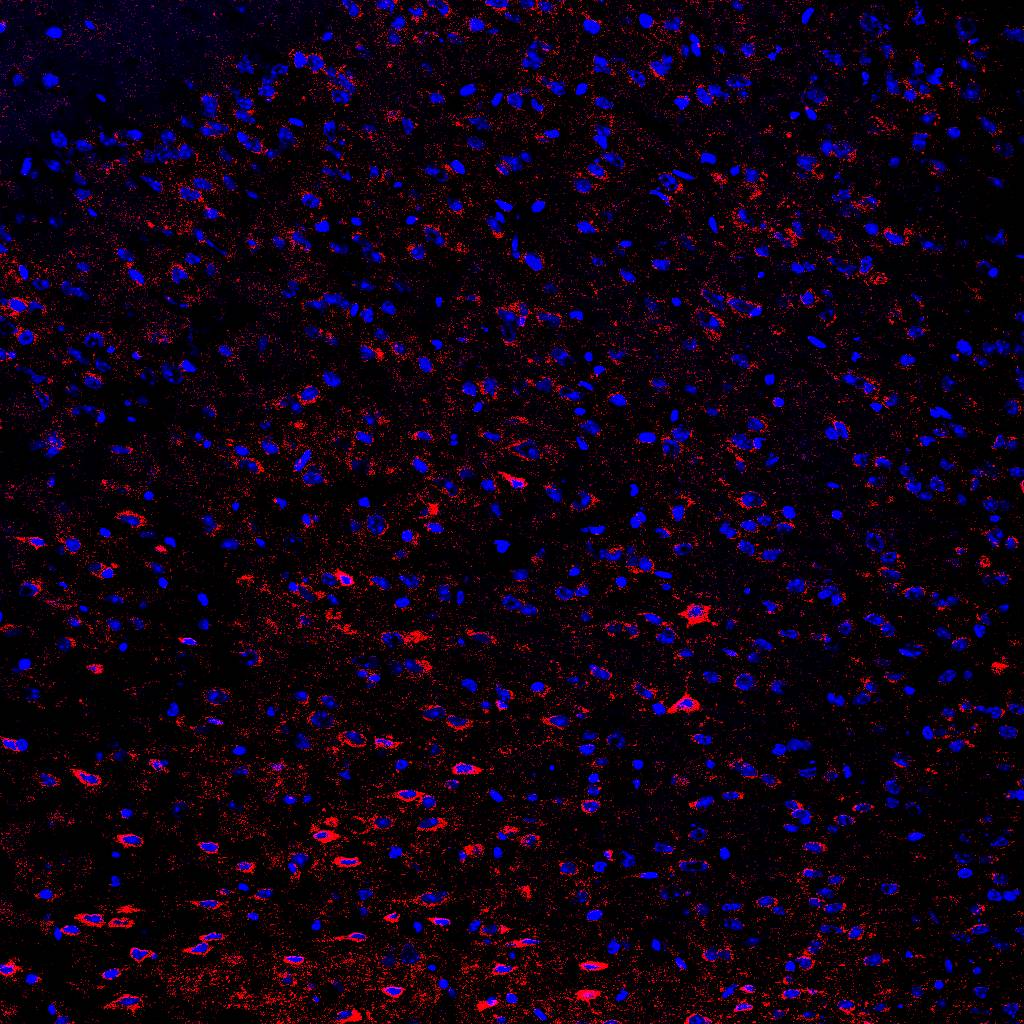

Supplement: Supplementary file 10 — Appendix Figure Source Data [file 44321_2025_206_MOESM10_ESM.zip › Appendix Figures Source Data/Appendix Fig. S13/S13-A/WT-MCAO-CON-MERGE.tif]

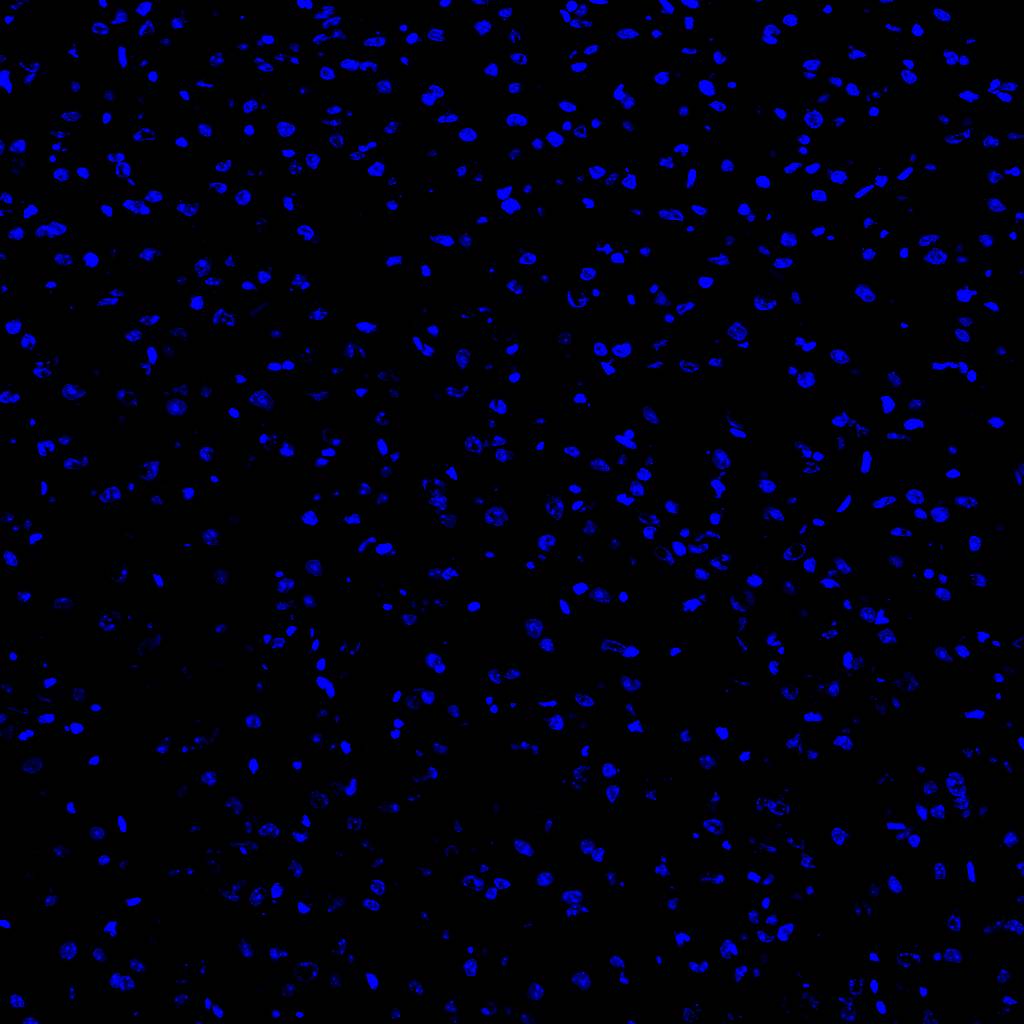

Supplement: Supplementary file 10 — Appendix Figure Source Data [file 44321_2025_206_MOESM10_ESM.zip › Appendix Figures Source Data/Appendix Fig. S13/S13-A/WT-MCAO-GSK-872-DAPI.tif]

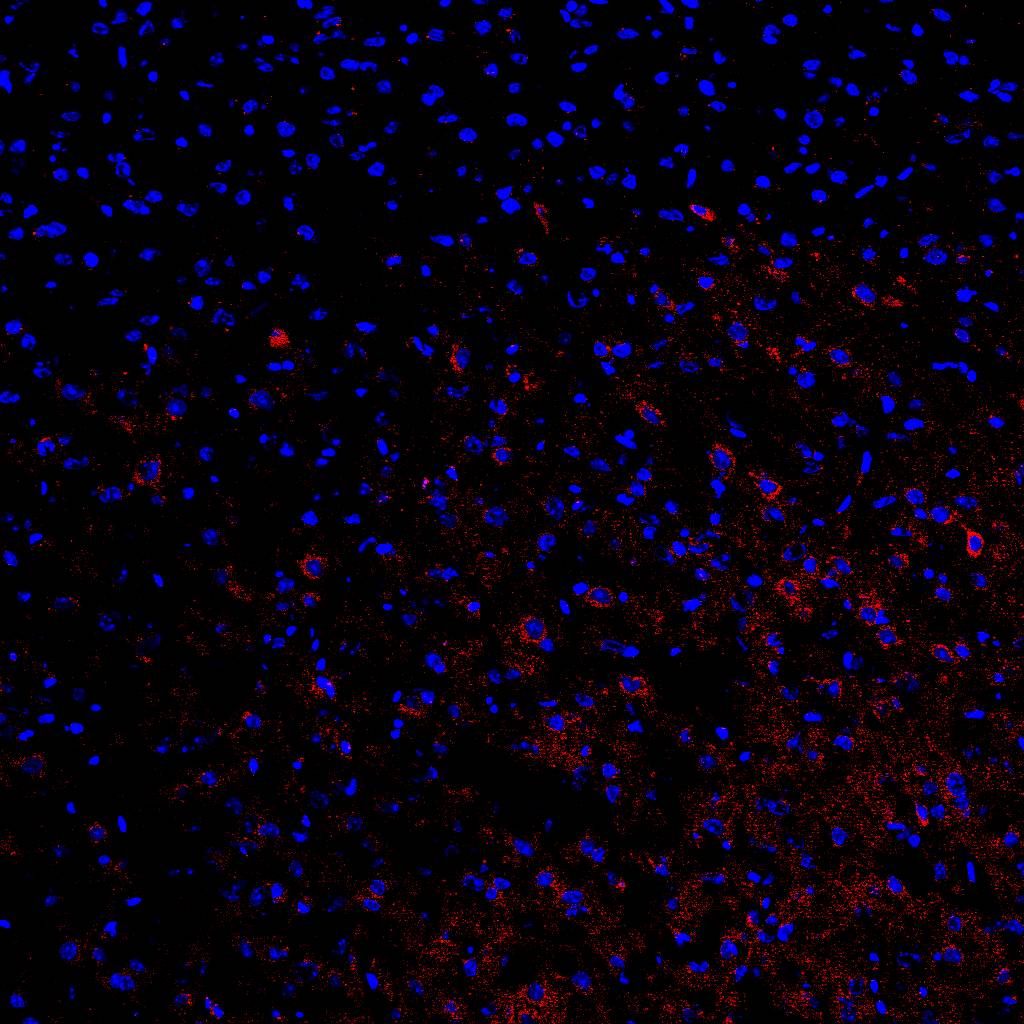

Supplement: Supplementary file 10 — Appendix Figure Source Data [file 44321_2025_206_MOESM10_ESM.zip › Appendix Figures Source Data/Appendix Fig. S13/S13-A/WT-MCAO-GSK-872-MERGE.tif]

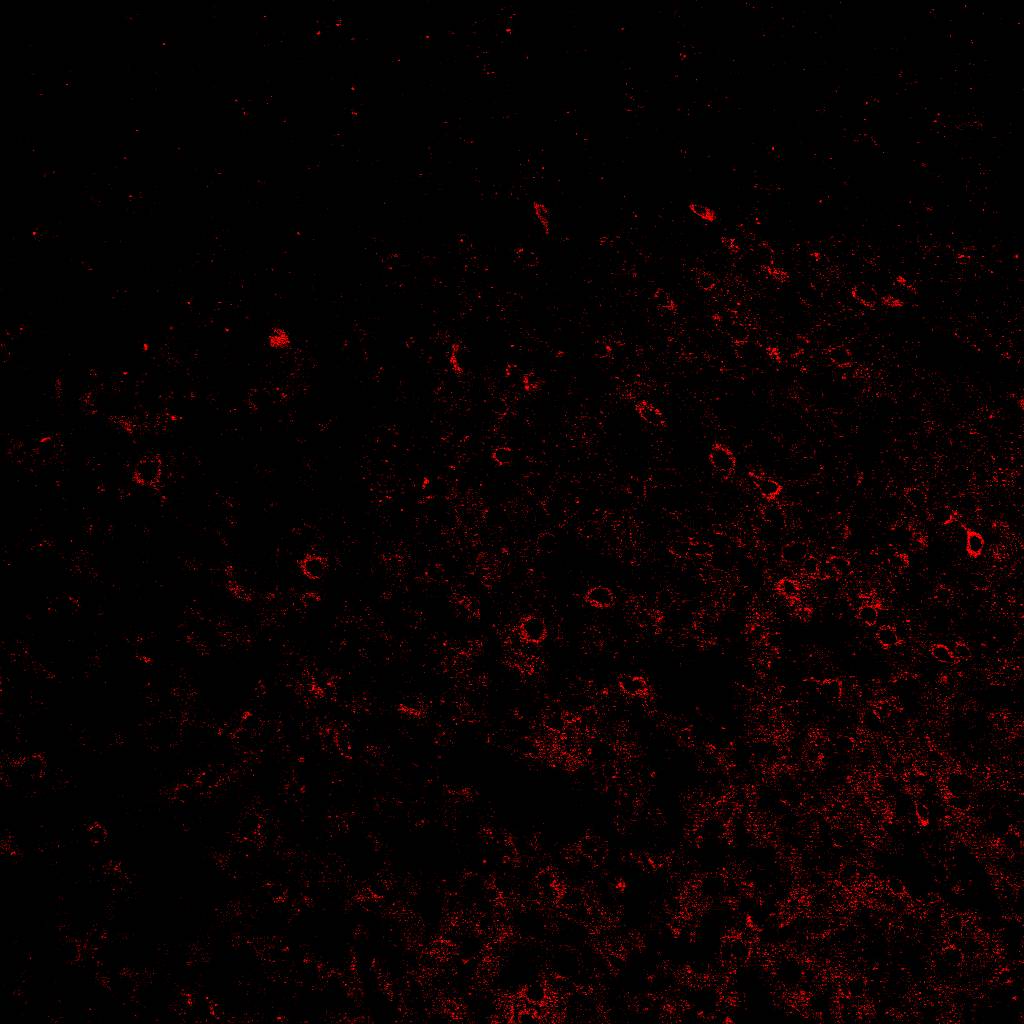

Supplement: Supplementary file 10 — Appendix Figure Source Data [file 44321_2025_206_MOESM10_ESM.zip › Appendix Figures Source Data/Appendix Fig. S13/S13-A/WT-MCAO-GSK-872-P-RIPK3.tif]

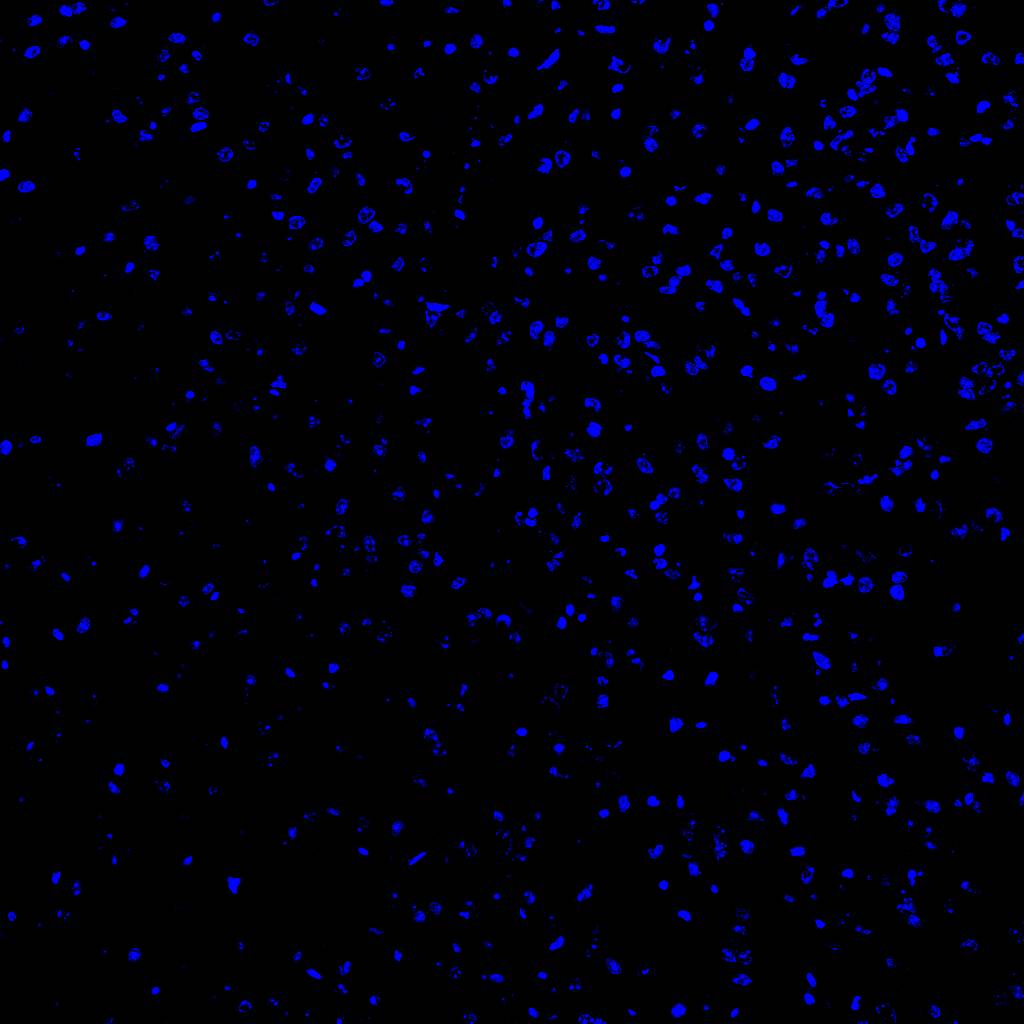

Supplement: Supplementary file 10 — Appendix Figure Source Data [file 44321_2025_206_MOESM10_ESM.zip › Appendix Figures Source Data/Appendix Fig. S13/S13-A/WT-MCAO-LN5P45-DAPI.tif]

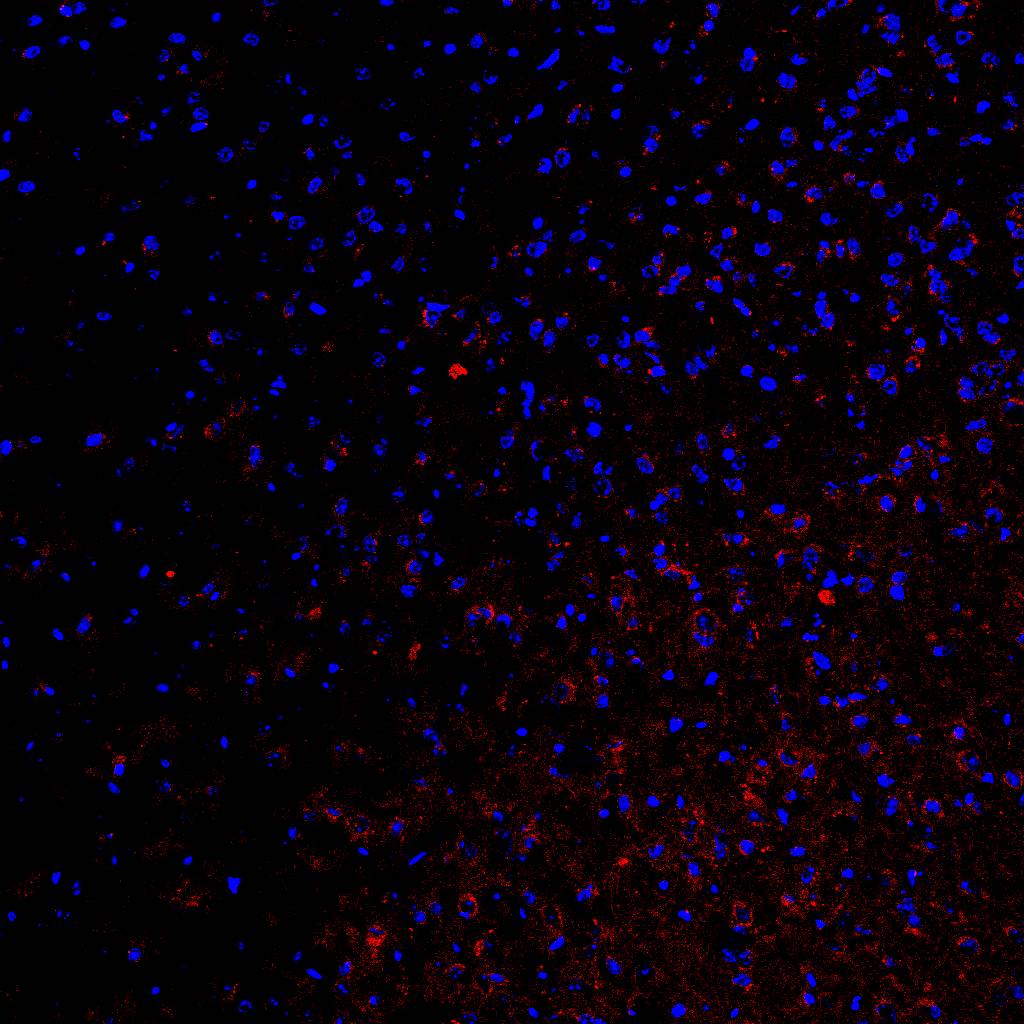

Supplement: Supplementary file 10 — Appendix Figure Source Data [file 44321_2025_206_MOESM10_ESM.zip › Appendix Figures Source Data/Appendix Fig. S13/S13-A/WT-MCAO-LN5P45-MERGE.tif]

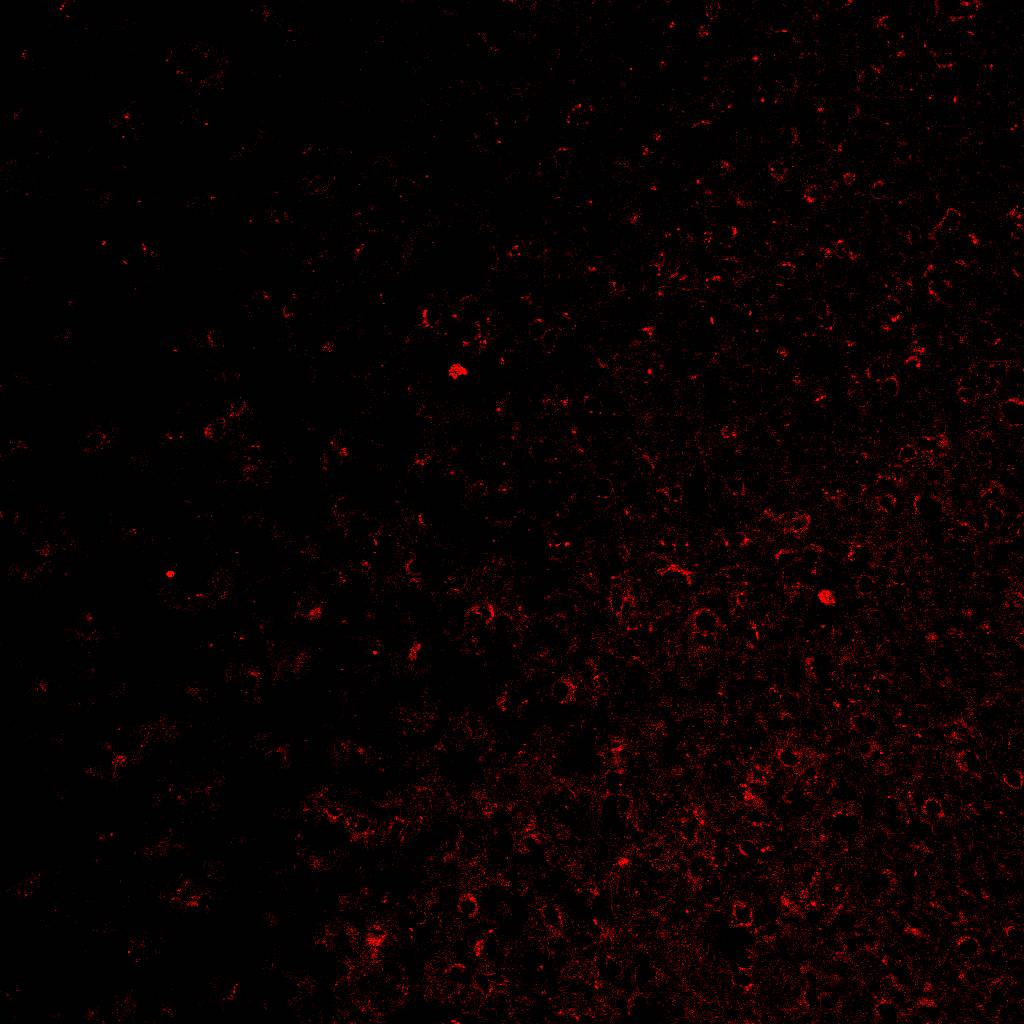

Supplement: Supplementary file 10 — Appendix Figure Source Data [file 44321_2025_206_MOESM10_ESM.zip › Appendix Figures Source Data/Appendix Fig. S13/S13-A/WT-MCAO-LN5P45-P-RIPK3.tif]

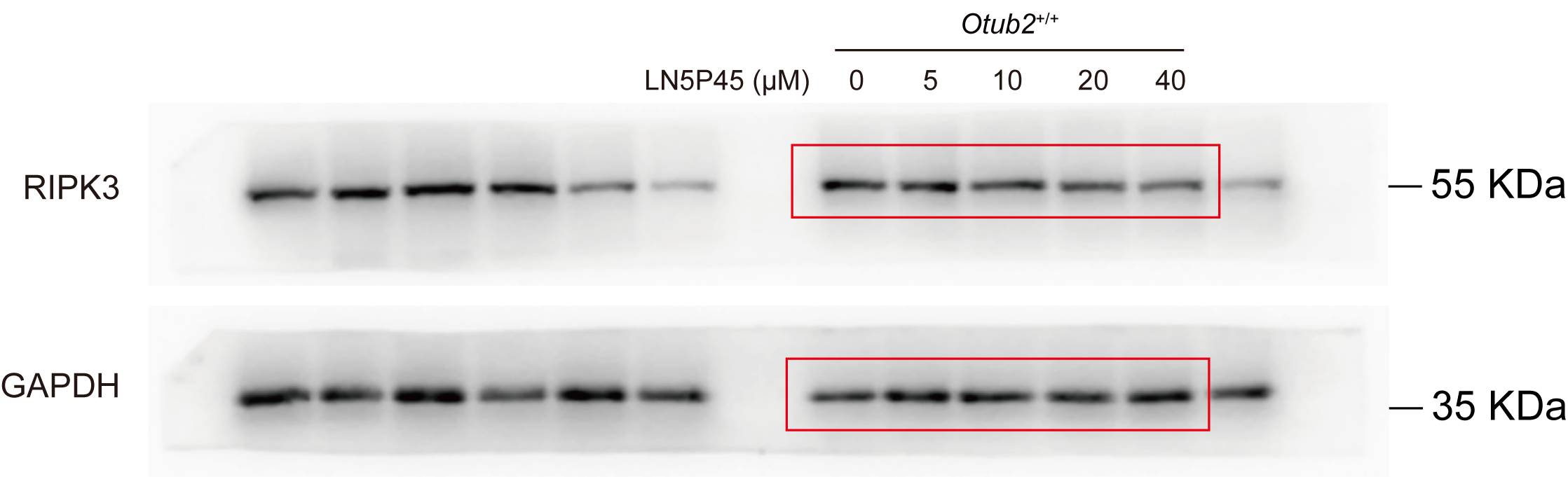

Supplement: Supplementary file 10 — Appendix Figure Source Data [file 44321_2025_206_MOESM10_ESM.zip › Appendix Figures Source Data/Appendix Fig. S13/S13-C/S13-C.tif]

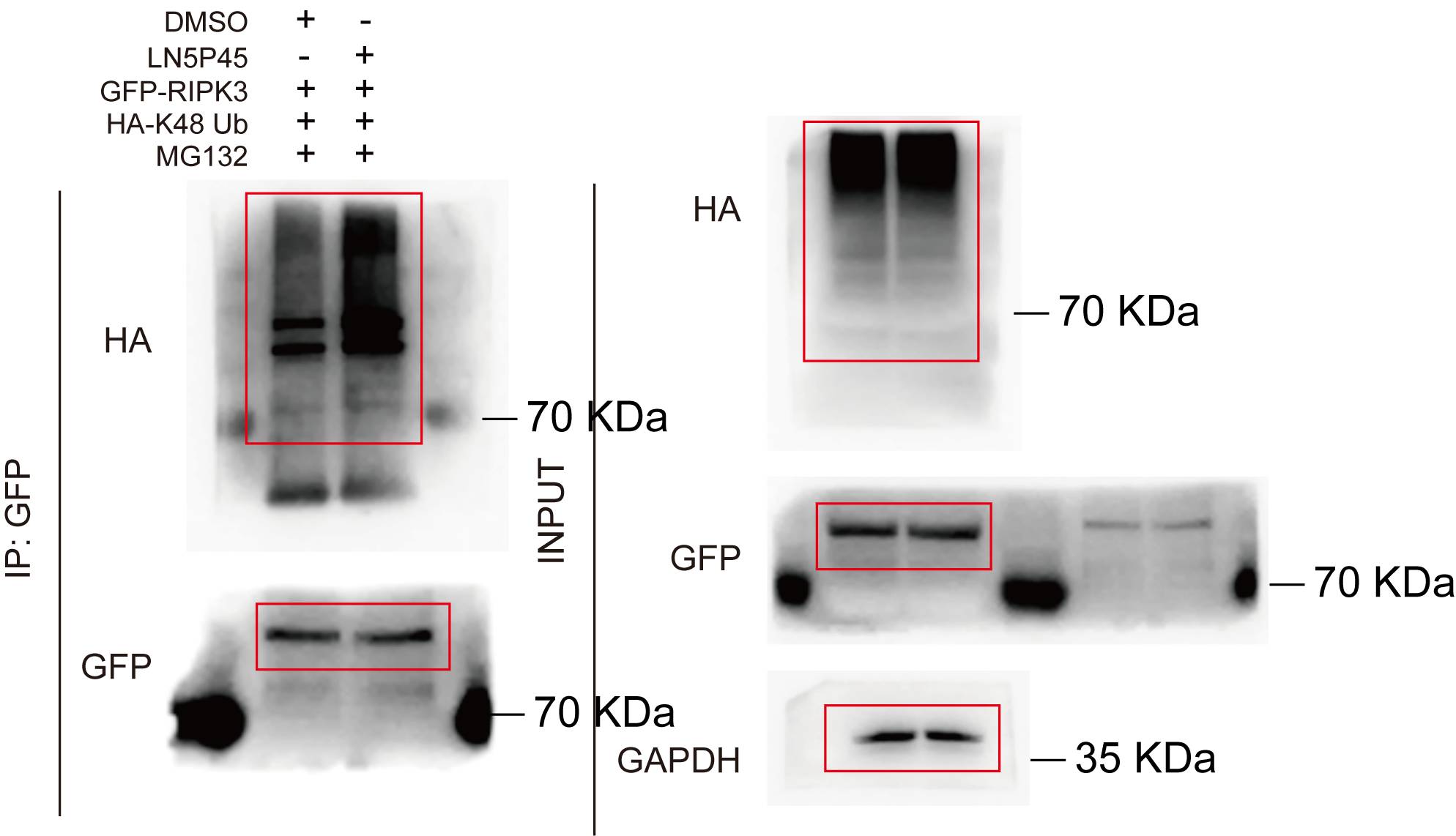

Supplement: Supplementary file 10 — Appendix Figure Source Data [file 44321_2025_206_MOESM10_ESM.zip › Appendix Figures Source Data/Appendix Fig. S13/S13-D/S13-D.tif]

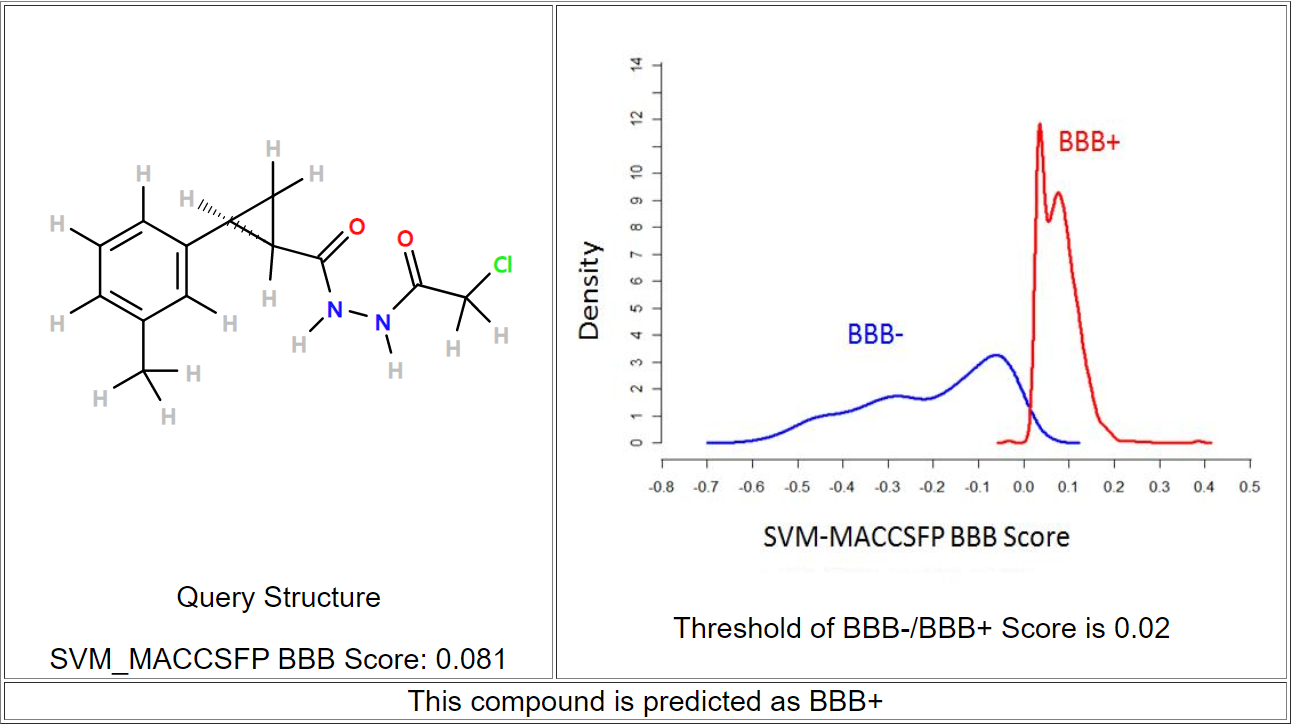

Supplement: Supplementary file 10 — Appendix Figure Source Data [file 44321_2025_206_MOESM10_ESM.zip › Appendix Figures Source Data/Appendix Fig. S14/S14-A.png]

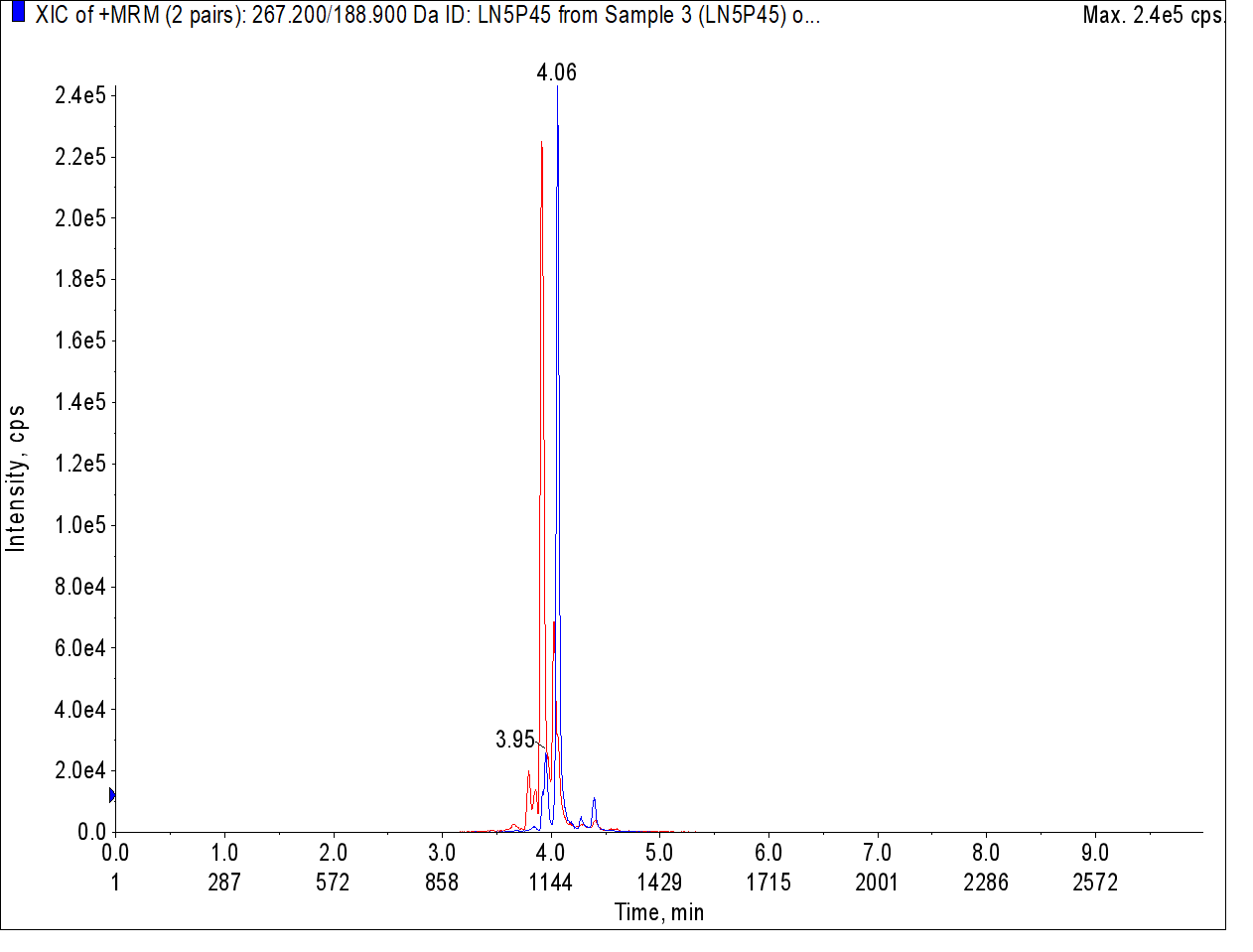

Supplement: Supplementary file 10 — Appendix Figure Source Data [file 44321_2025_206_MOESM10_ESM.zip › Appendix Figures Source Data/Appendix Fig. S14/S14-C/LN5P45-1.png]

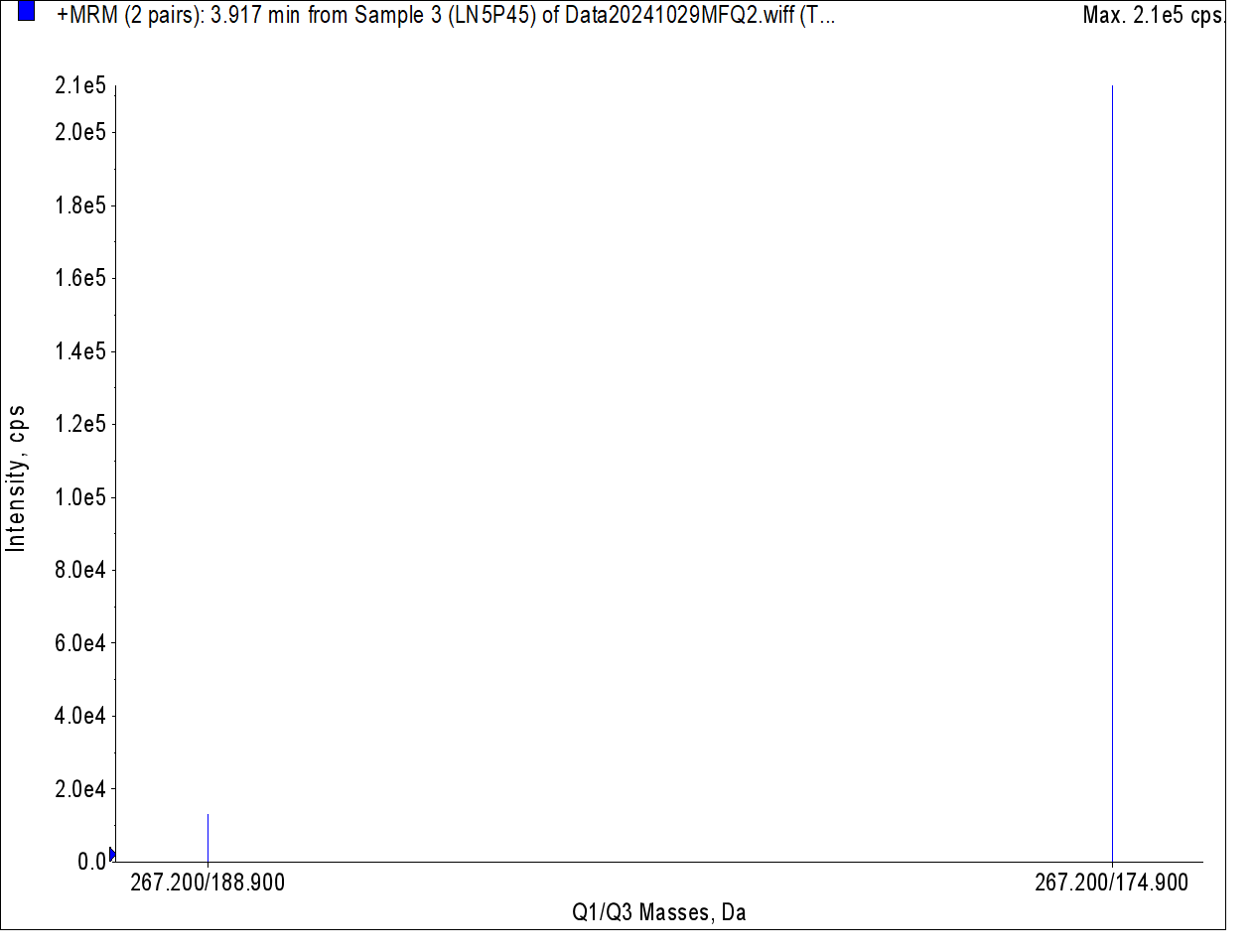

Supplement: Supplementary file 10 — Appendix Figure Source Data [file 44321_2025_206_MOESM10_ESM.zip › Appendix Figures Source Data/Appendix Fig. S14/S14-C/LN5P45-2.png]

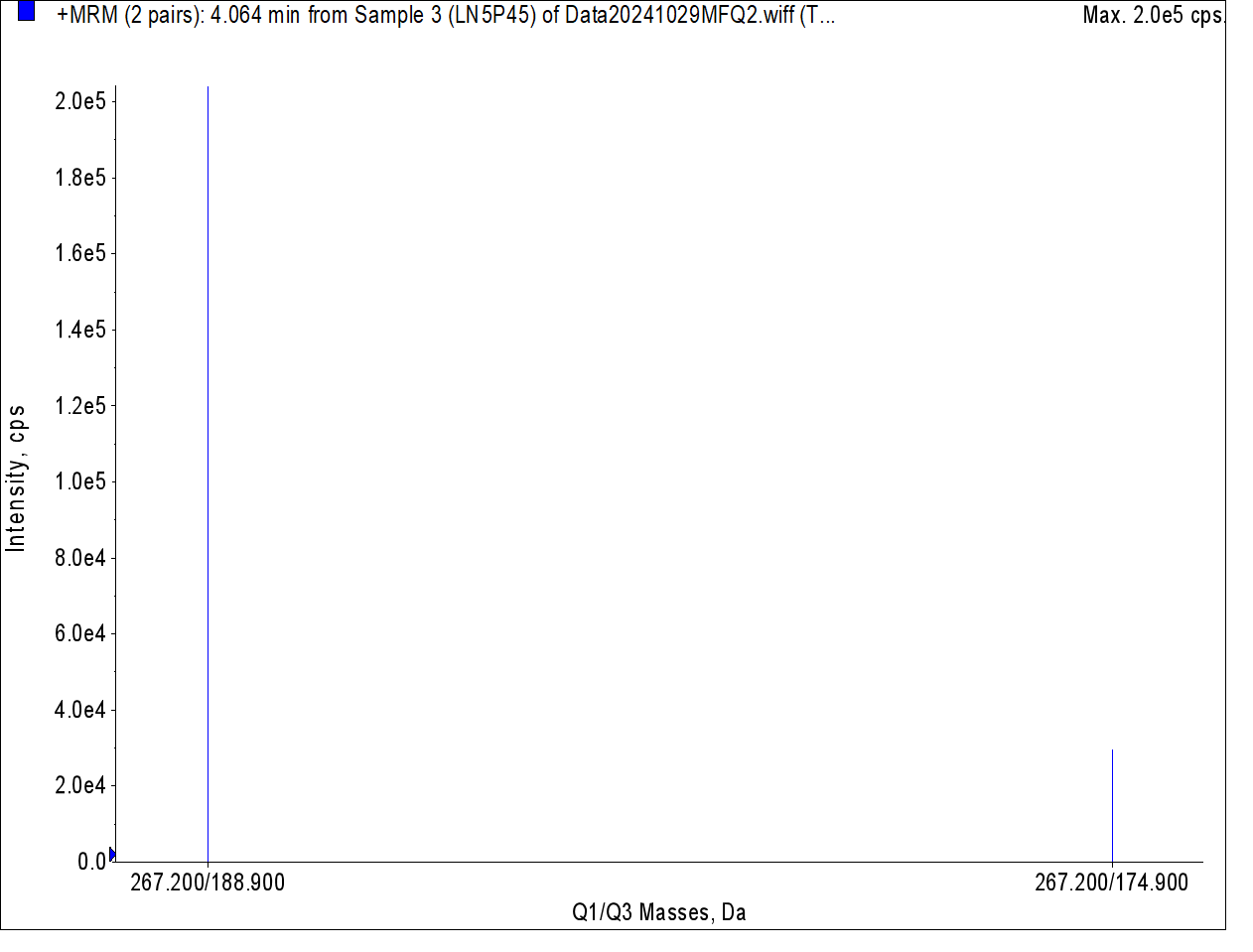

Supplement: Supplementary file 10 — Appendix Figure Source Data [file 44321_2025_206_MOESM10_ESM.zip › Appendix Figures Source Data/Appendix Fig. S14/S14-C/LN5P45-3.png]

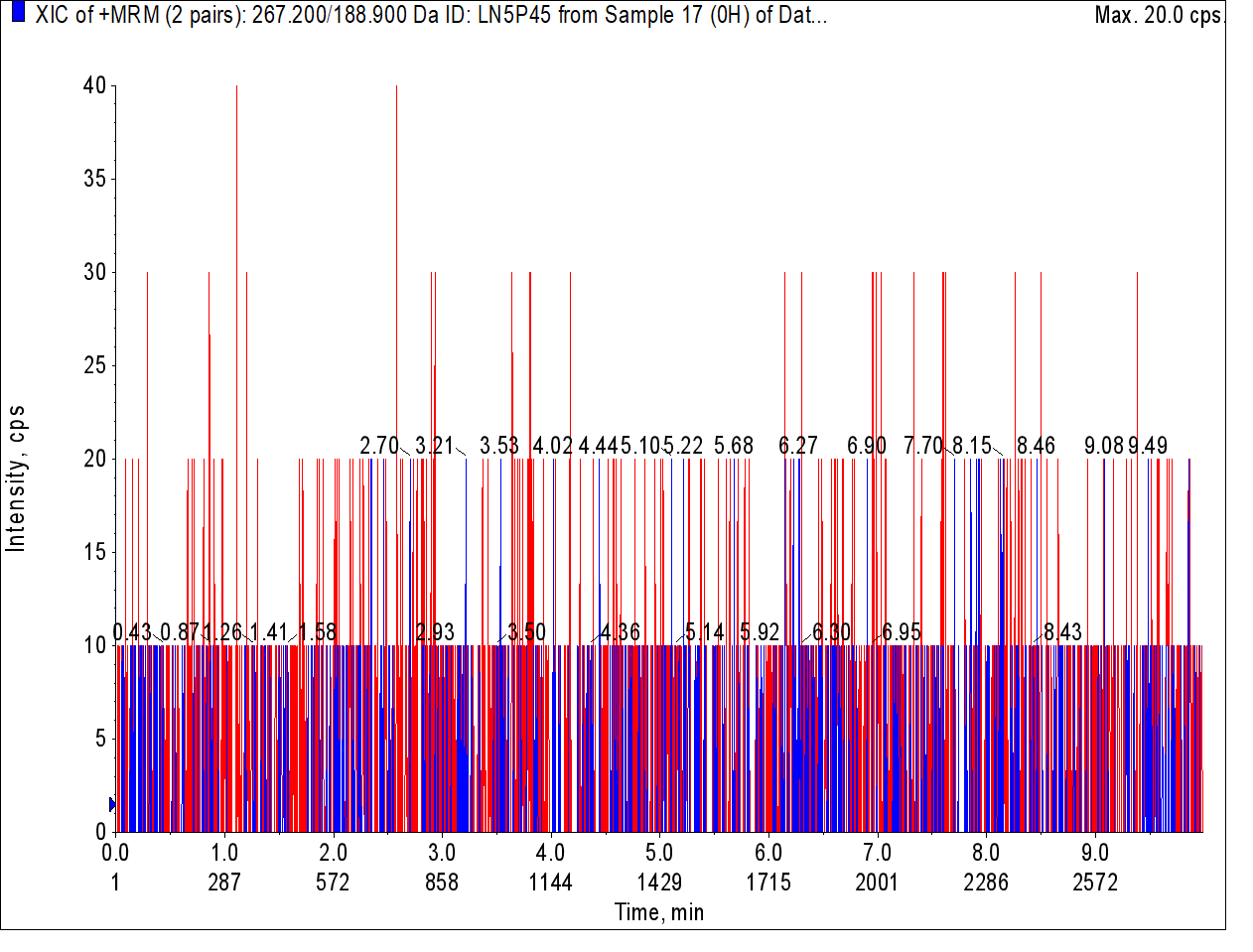

Supplement: Supplementary file 10 — Appendix Figure Source Data [file 44321_2025_206_MOESM10_ESM.zip › Appendix Figures Source Data/Appendix Fig. S14/S14-C/MCAO-1H-CON -1.png]

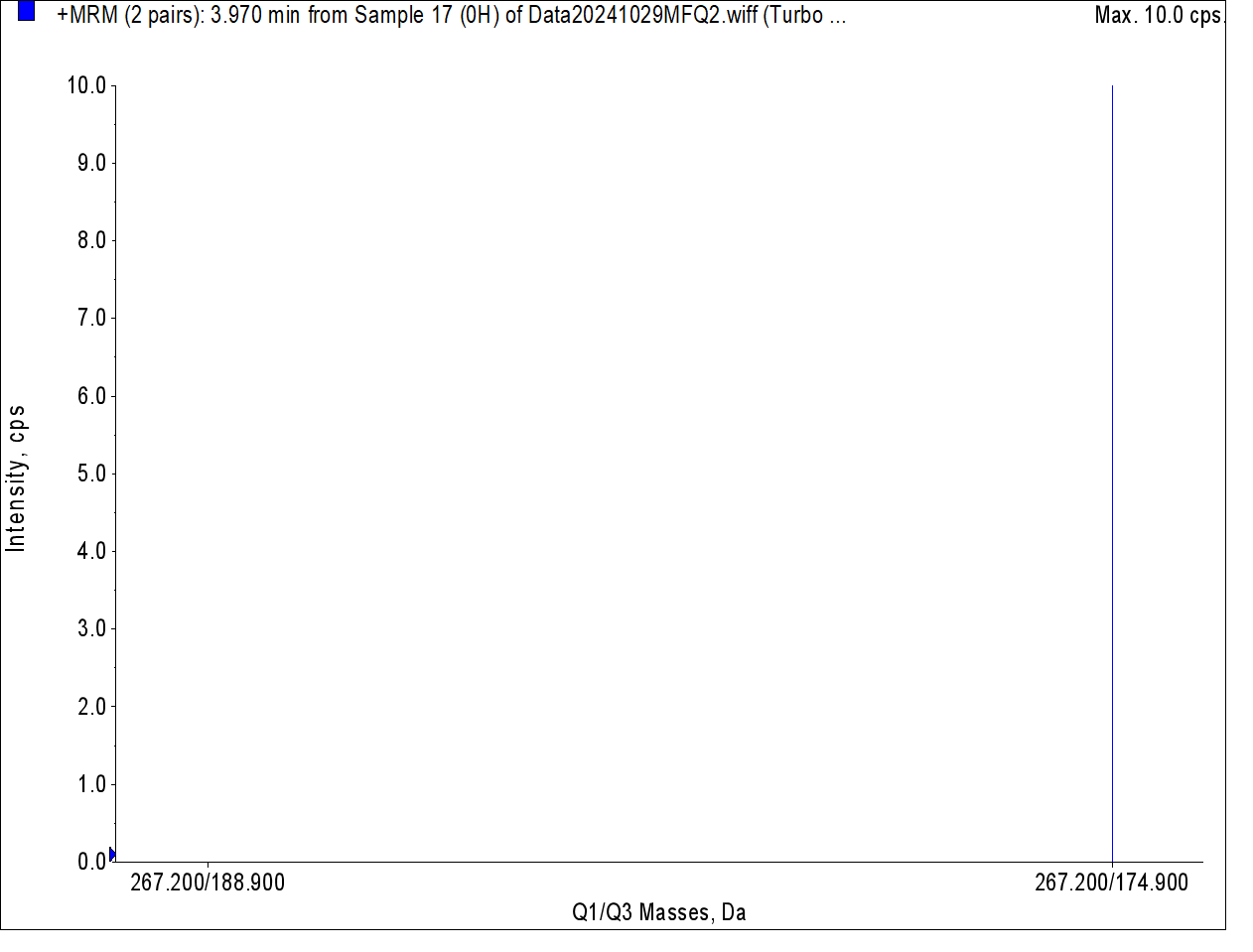

Supplement: Supplementary file 10 — Appendix Figure Source Data [file 44321_2025_206_MOESM10_ESM.zip › Appendix Figures Source Data/Appendix Fig. S14/S14-C/MCAO-1H-CON -2.png]

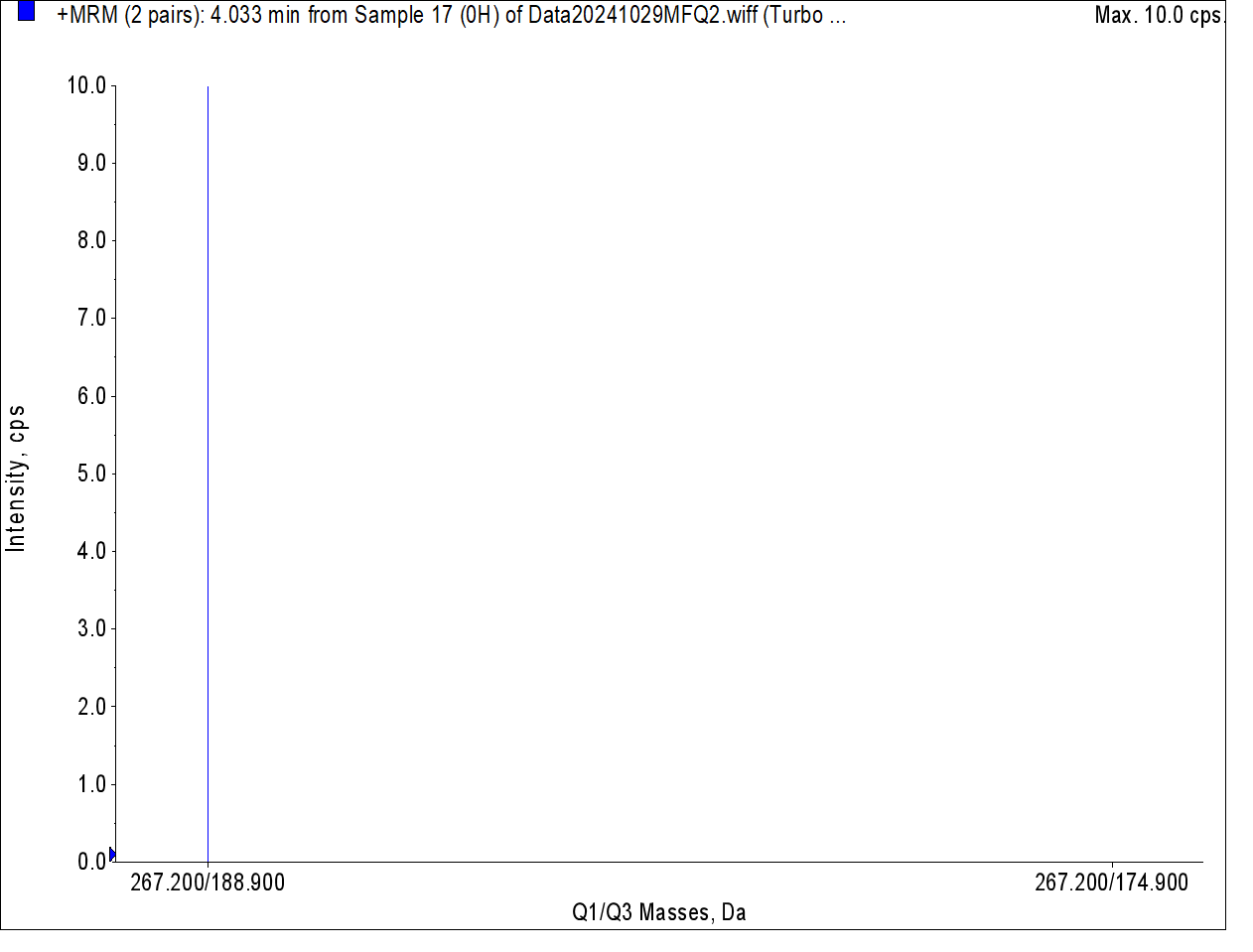

Supplement: Supplementary file 10 — Appendix Figure Source Data [file 44321_2025_206_MOESM10_ESM.zip › Appendix Figures Source Data/Appendix Fig. S14/S14-C/MCAO-1H-CON -3.png]

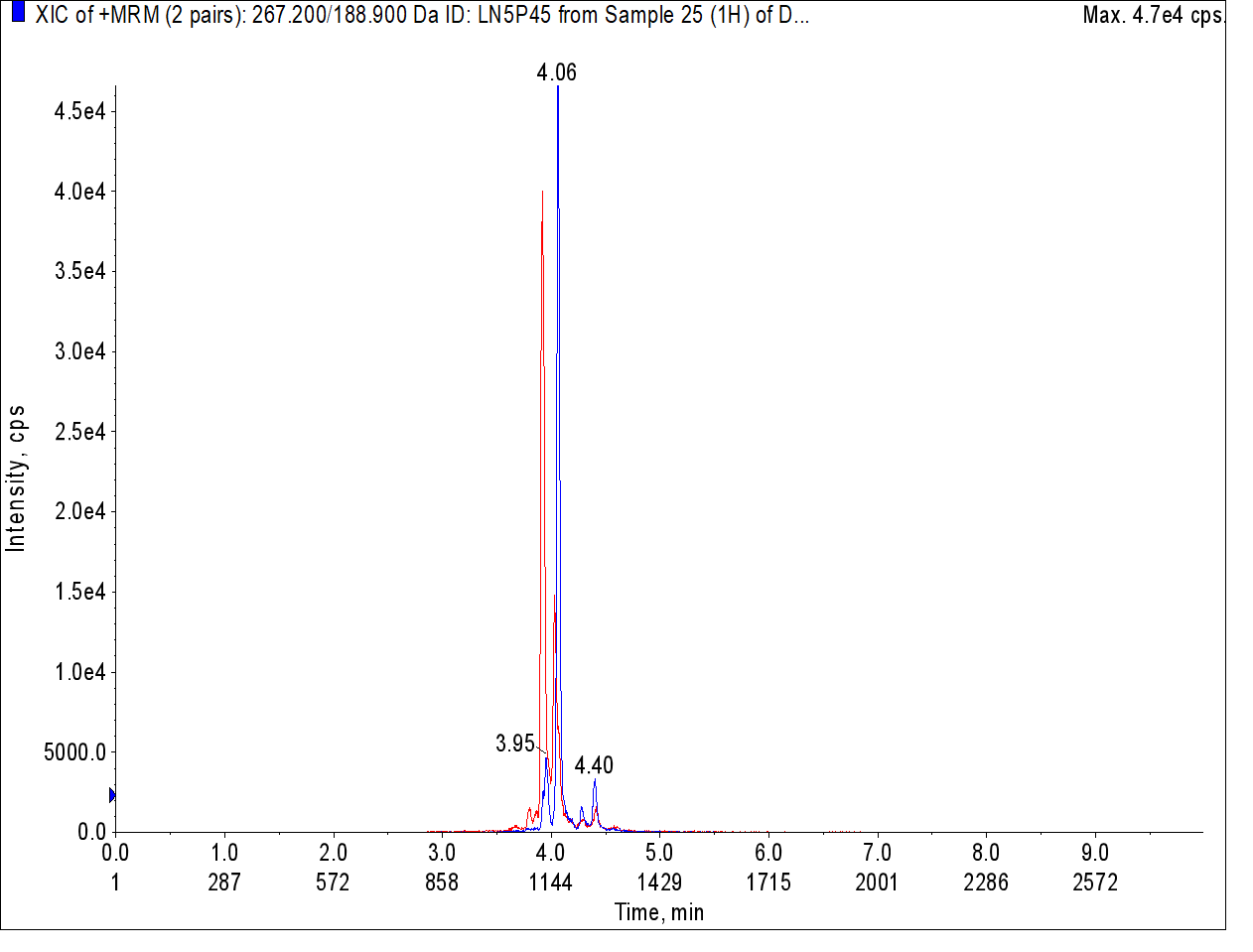

Supplement: Supplementary file 10 — Appendix Figure Source Data [file 44321_2025_206_MOESM10_ESM.zip › Appendix Figures Source Data/Appendix Fig. S14/S14-C/MCAO-1H-LN5P45-1.png]

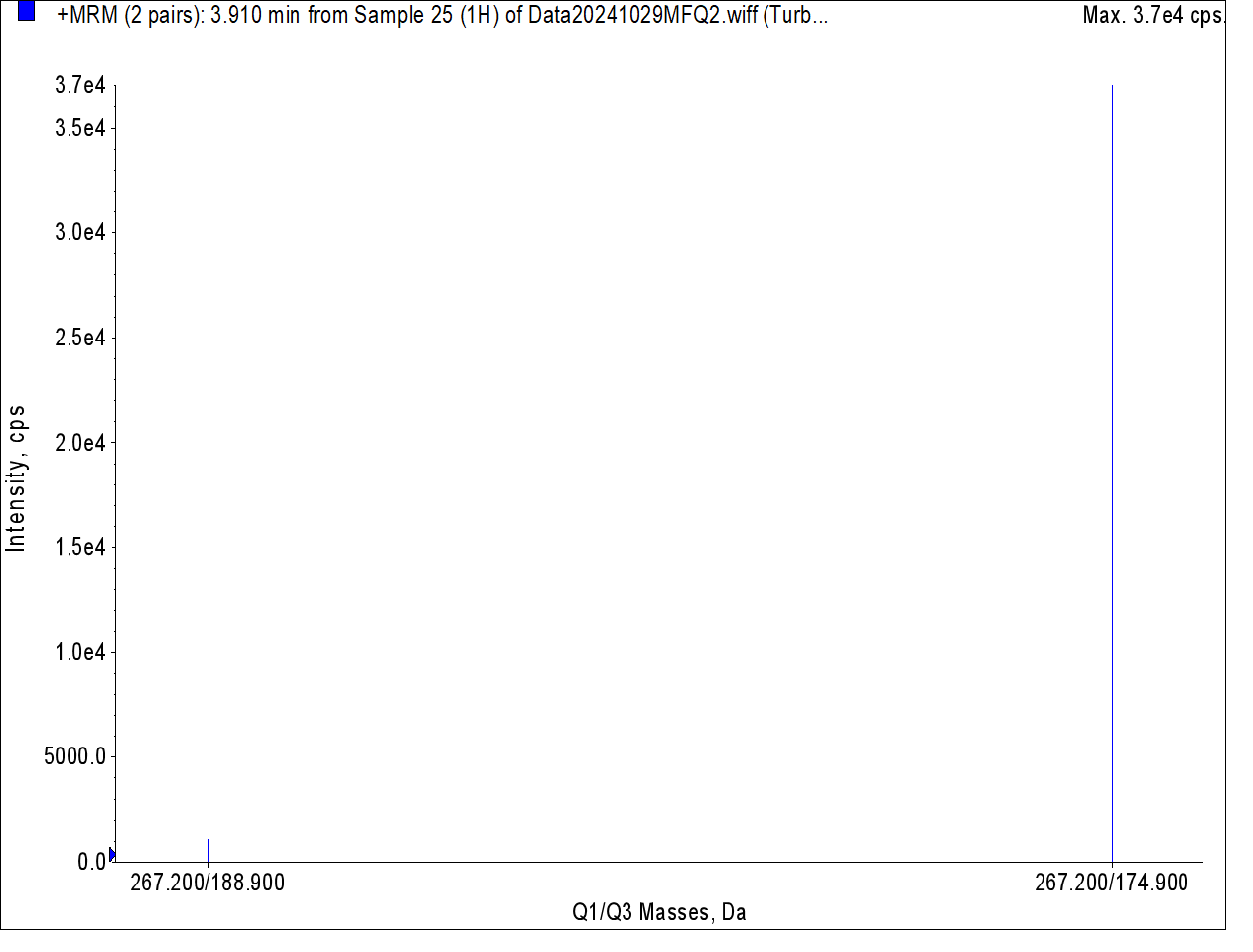

Supplement: Supplementary file 10 — Appendix Figure Source Data [file 44321_2025_206_MOESM10_ESM.zip › Appendix Figures Source Data/Appendix Fig. S14/S14-C/MCAO-1H-LN5P45-2.png]

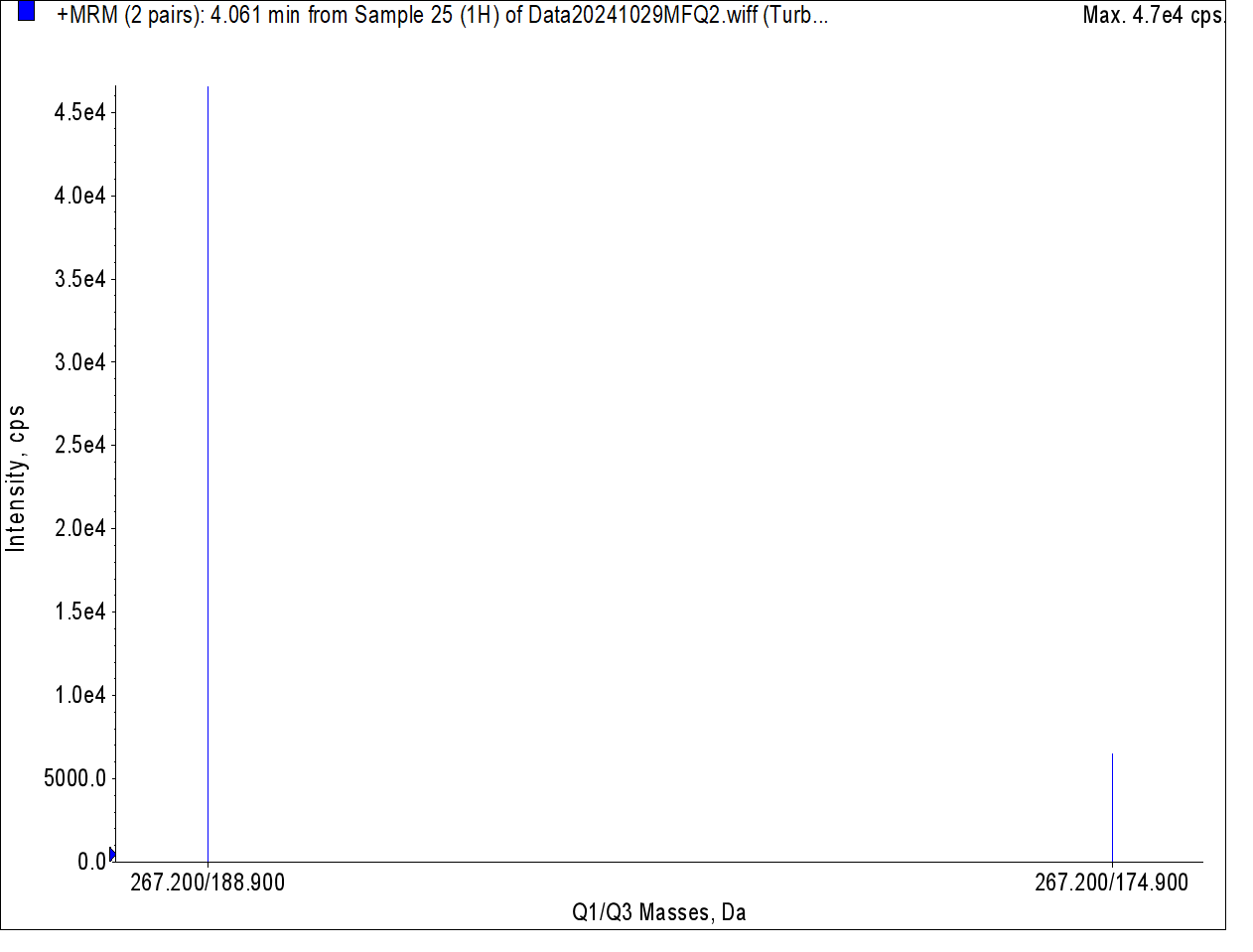

Supplement: Supplementary file 10 — Appendix Figure Source Data [file 44321_2025_206_MOESM10_ESM.zip › Appendix Figures Source Data/Appendix Fig. S14/S14-C/MCAO-1H-LN5P45-3.png]

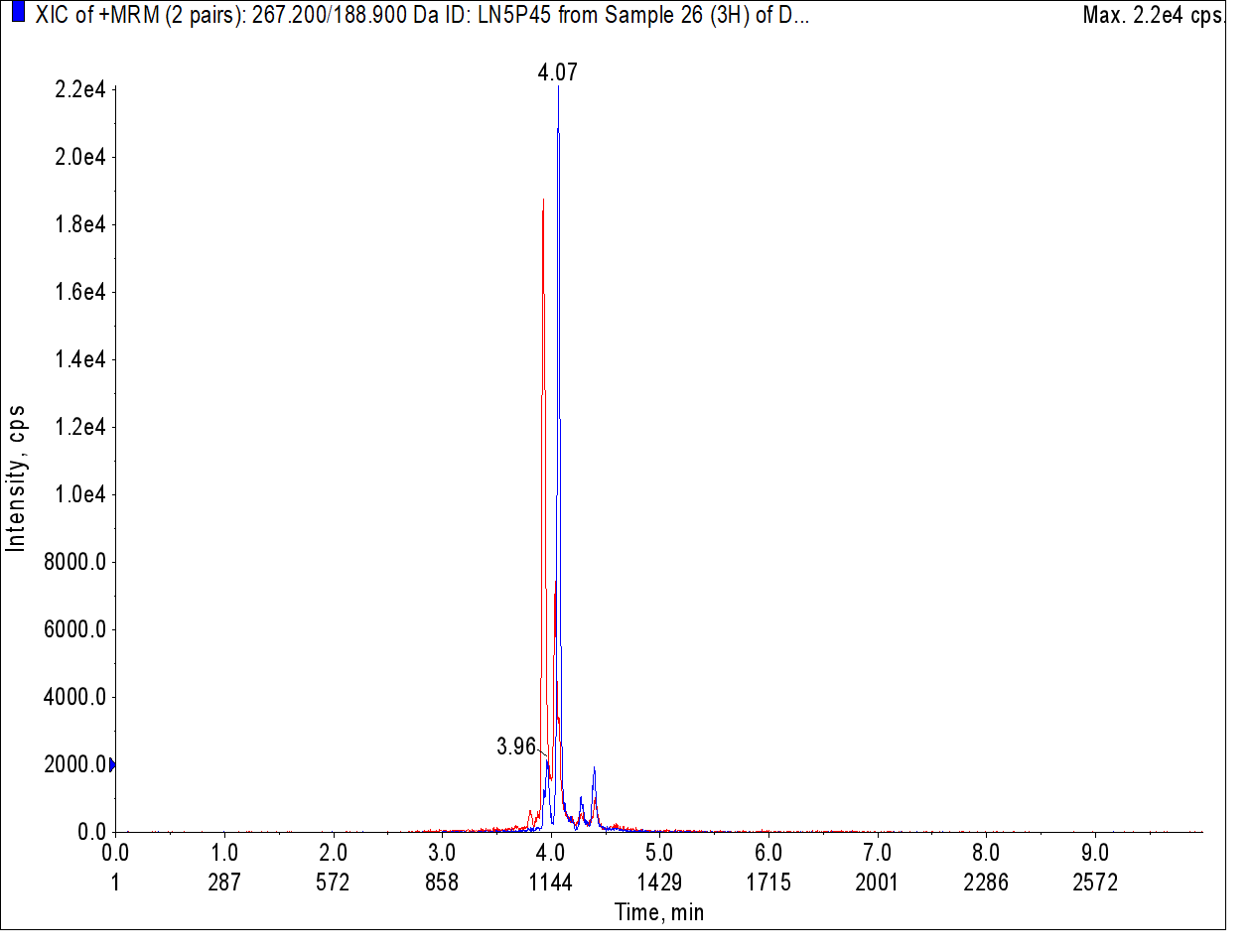

Supplement: Supplementary file 10 — Appendix Figure Source Data [file 44321_2025_206_MOESM10_ESM.zip › Appendix Figures Source Data/Appendix Fig. S14/S14-C/MCAO-3H-LN5P45-1.png]

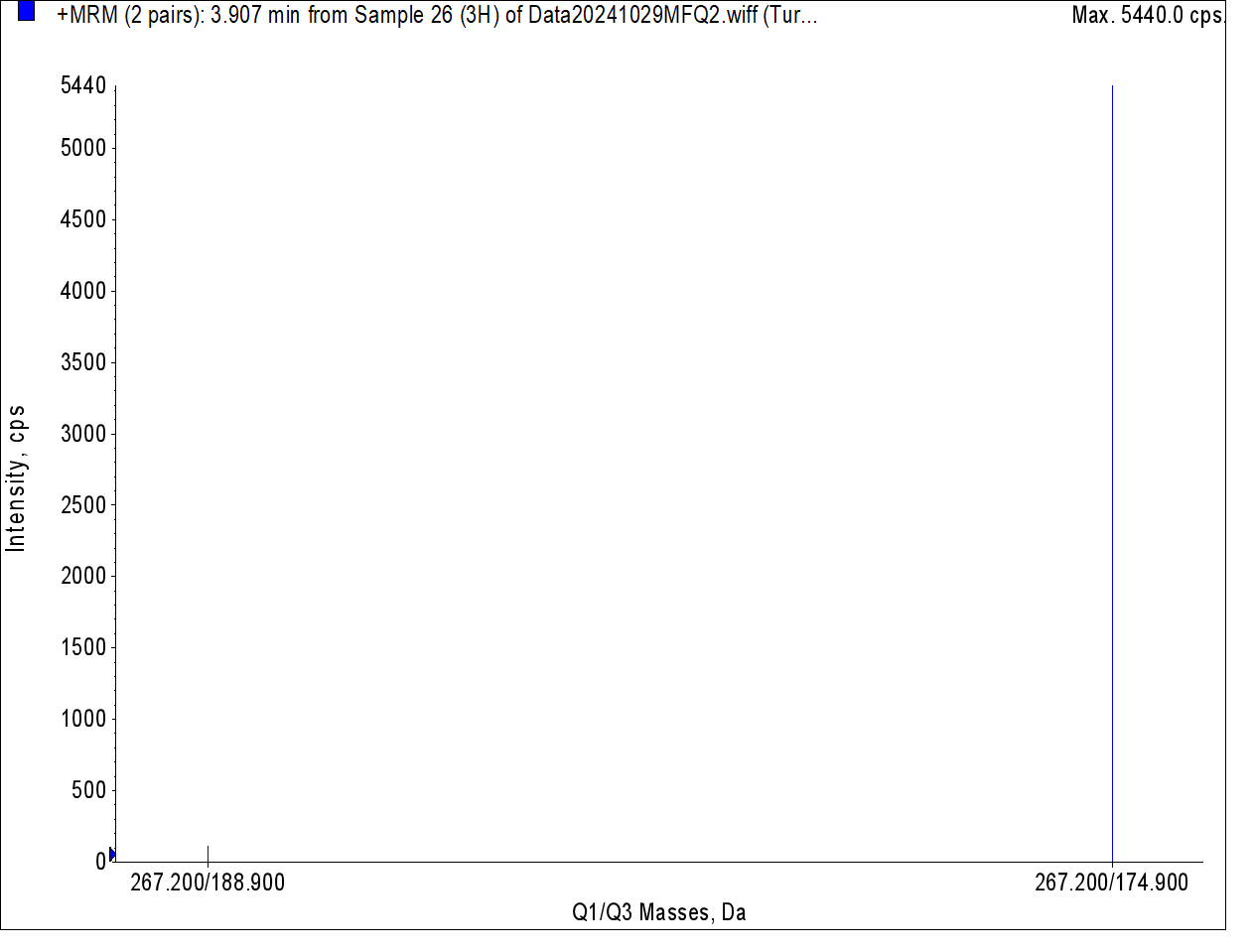

Supplement: Supplementary file 10 — Appendix Figure Source Data [file 44321_2025_206_MOESM10_ESM.zip › Appendix Figures Source Data/Appendix Fig. S14/S14-C/MCAO-3H-LN5P45-2.png]

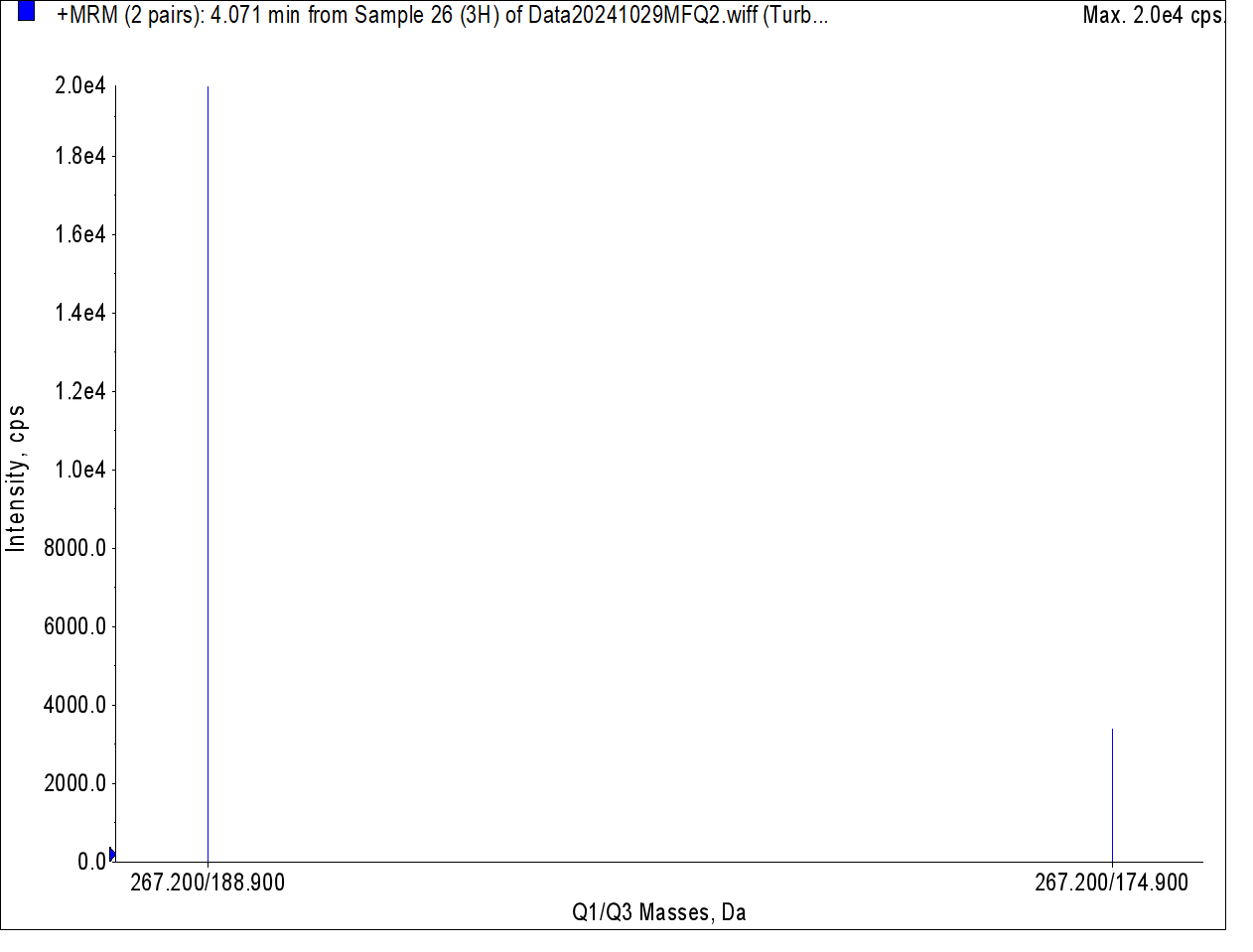

Supplement: Supplementary file 10 — Appendix Figure Source Data [file 44321_2025_206_MOESM10_ESM.zip › Appendix Figures Source Data/Appendix Fig. S14/S14-C/MCAO-3H-LN5P45-3.png]

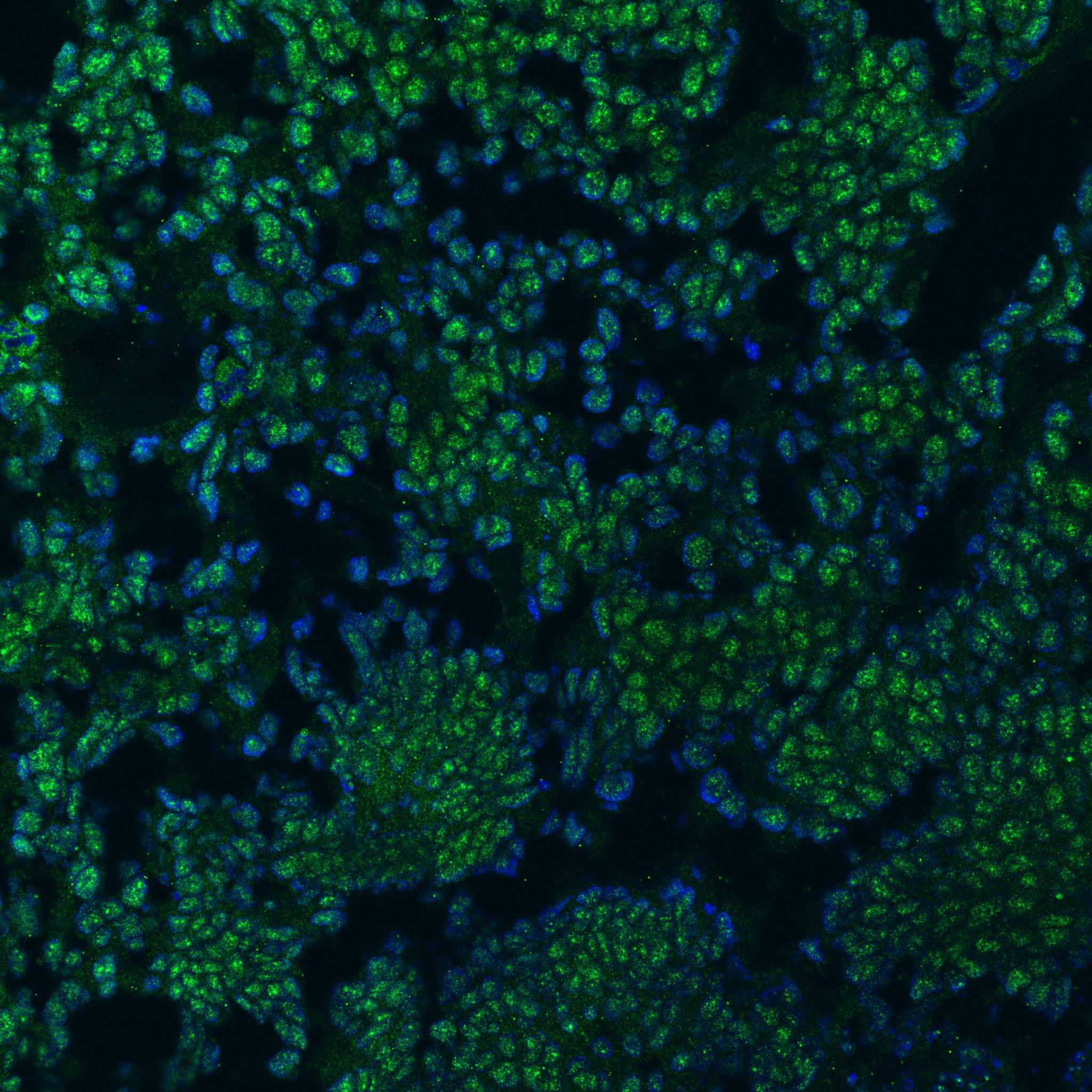

Supplement: Supplementary file 10 — Appendix Figure Source Data [file 44321_2025_206_MOESM10_ESM.zip › Appendix Figures Source Data/Appendix Fig. S15/LN5P45-Normoxia MERGE.tif]

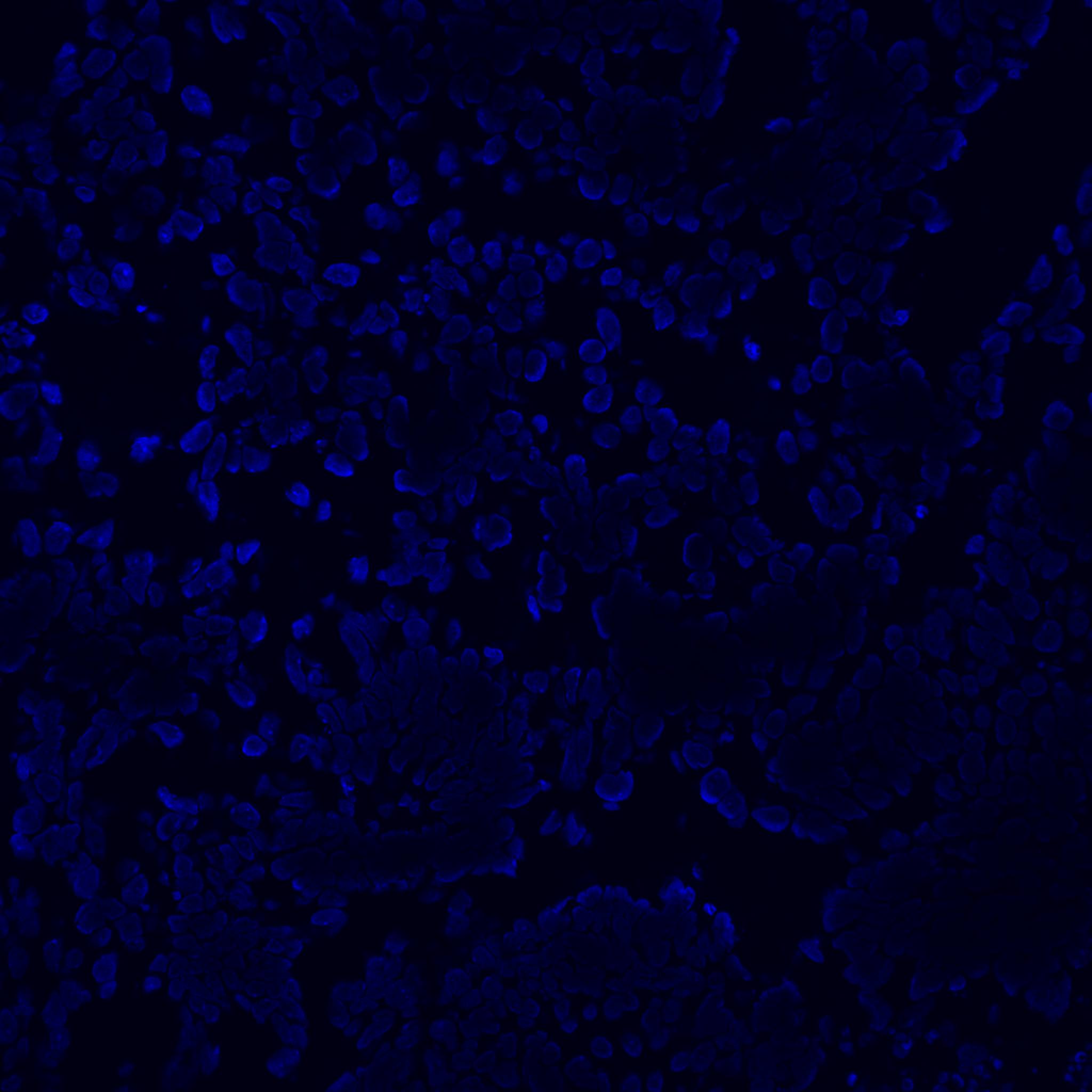

Supplement: Supplementary file 10 — Appendix Figure Source Data [file 44321_2025_206_MOESM10_ESM.zip › Appendix Figures Source Data/Appendix Fig. S15/LN5P45-Normoxia-DAPI.tif]

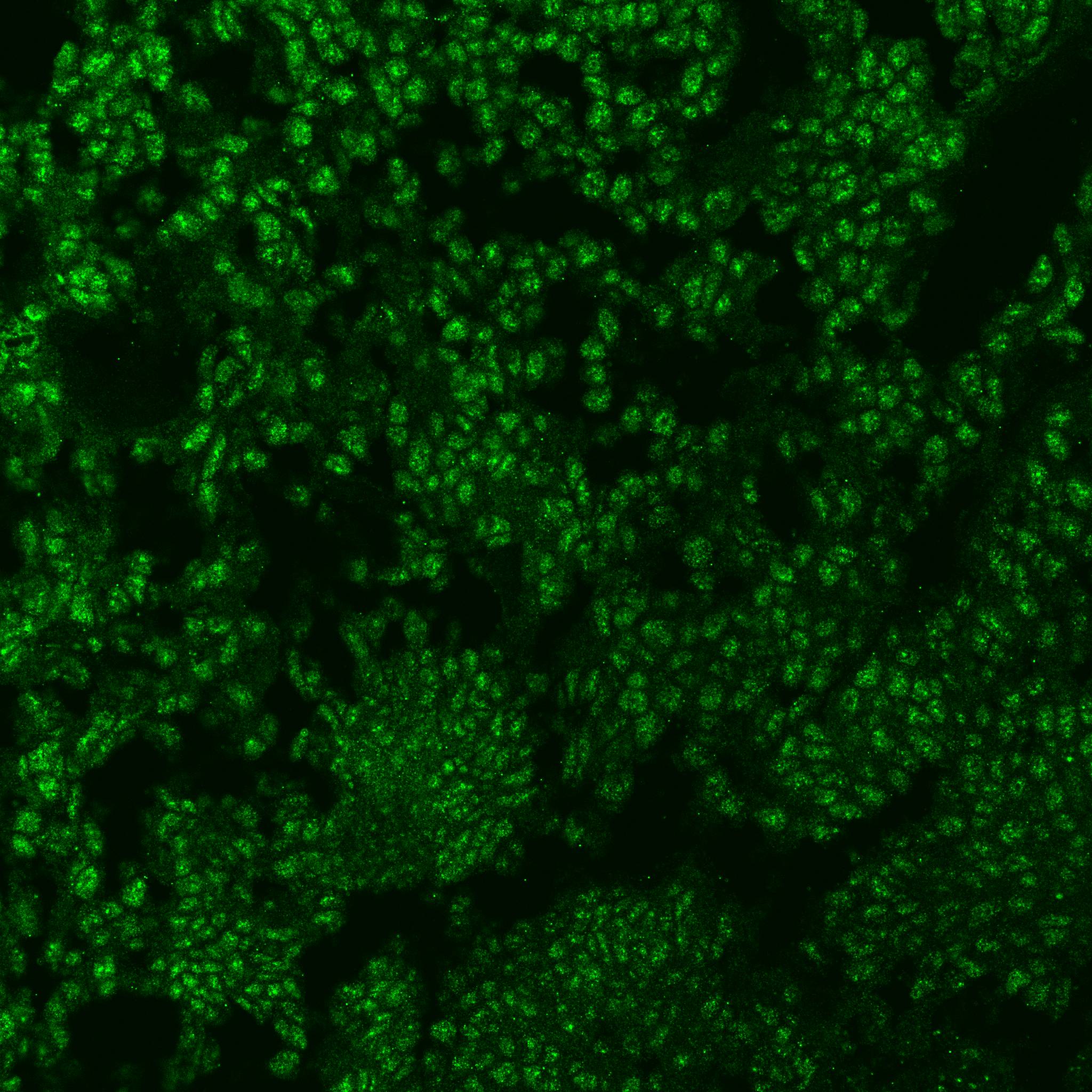

Supplement: Supplementary file 10 — Appendix Figure Source Data [file 44321_2025_206_MOESM10_ESM.zip › Appendix Figures Source Data/Appendix Fig. S15/LN5P45-Normoxia-NEUN.tif]

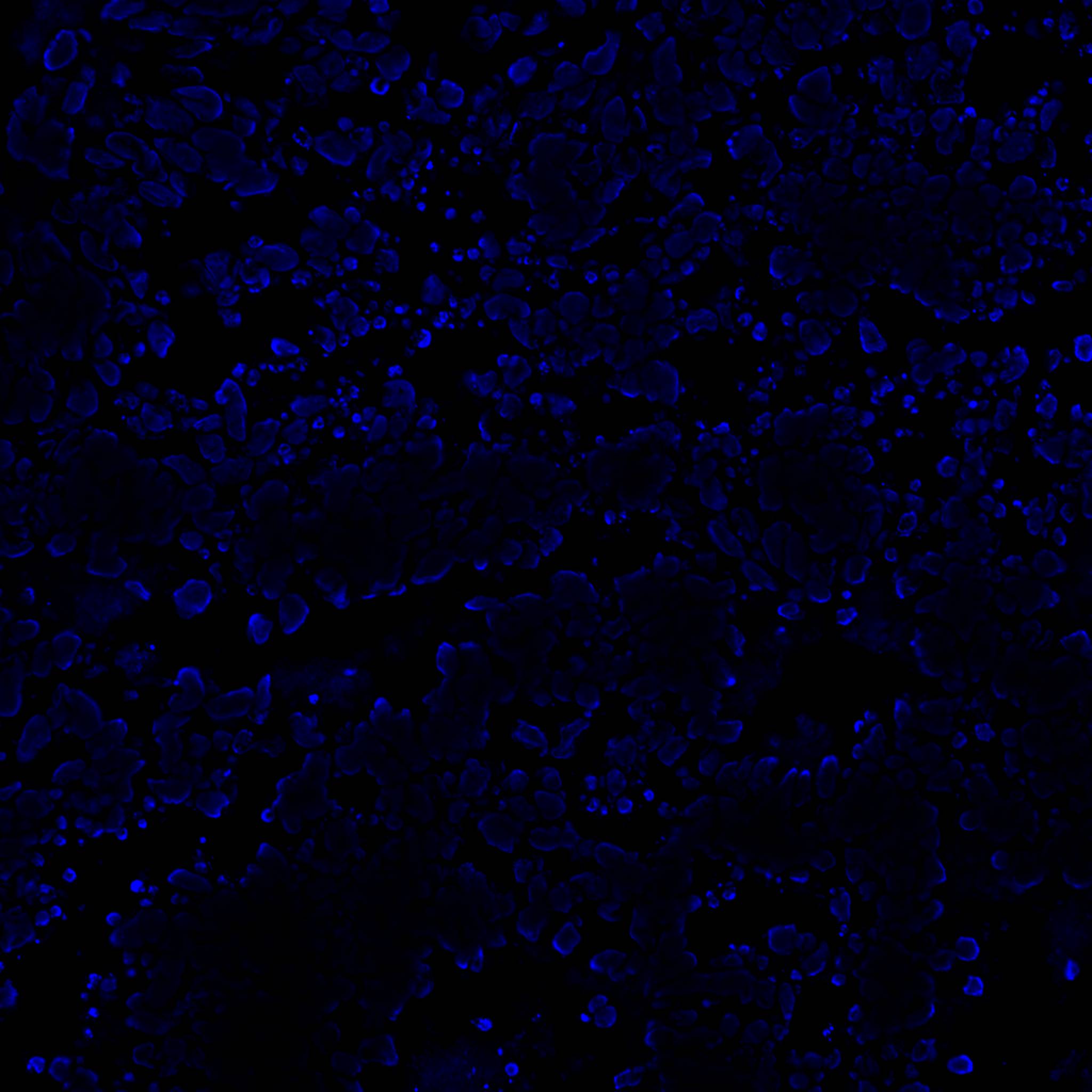

Supplement: Supplementary file 10 — Appendix Figure Source Data [file 44321_2025_206_MOESM10_ESM.zip › Appendix Figures Source Data/Appendix Fig. S15/LN5P45-OGD DAPI.tif]

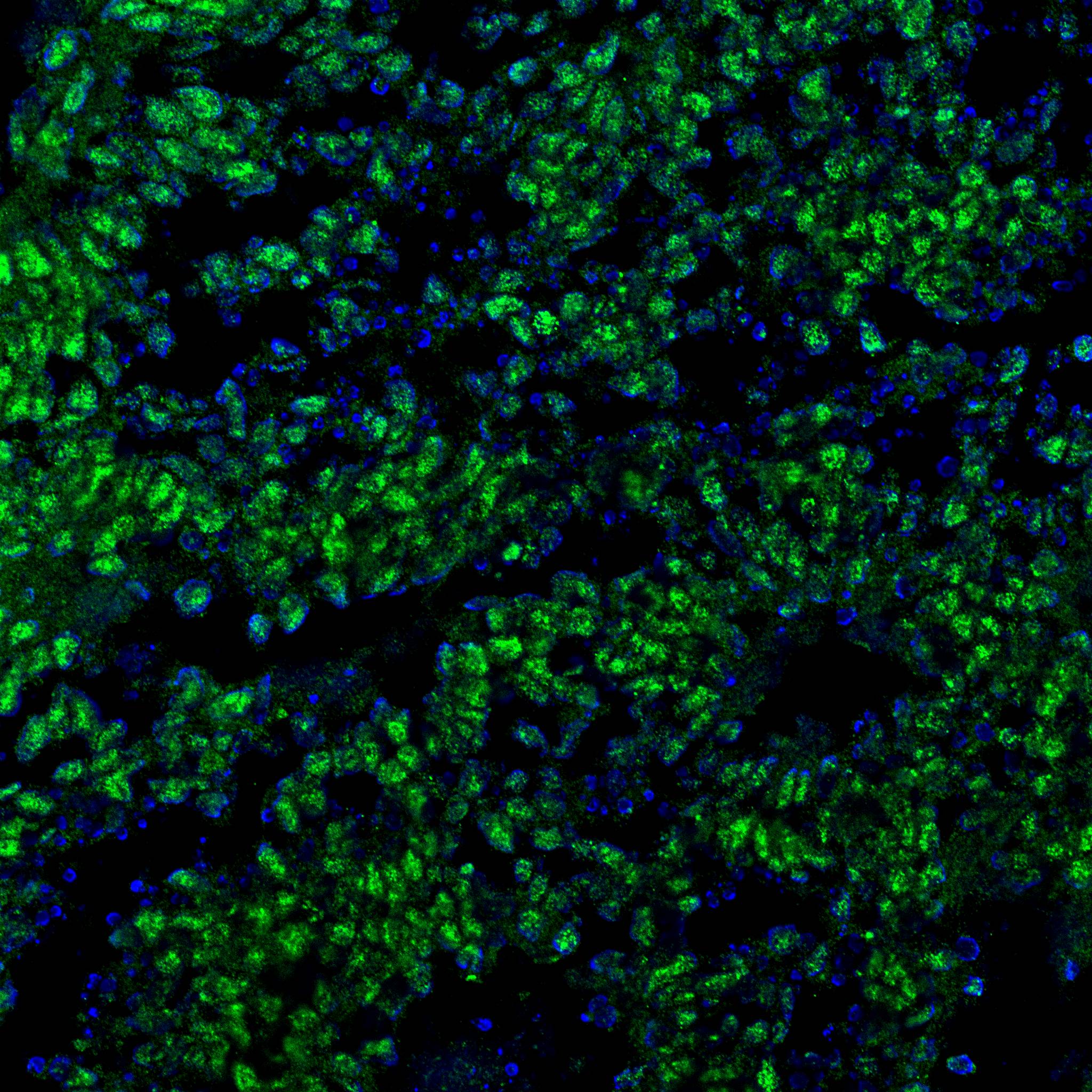

Supplement: Supplementary file 10 — Appendix Figure Source Data [file 44321_2025_206_MOESM10_ESM.zip › Appendix Figures Source Data/Appendix Fig. S15/LN5P45-OGD MERGE.tif]

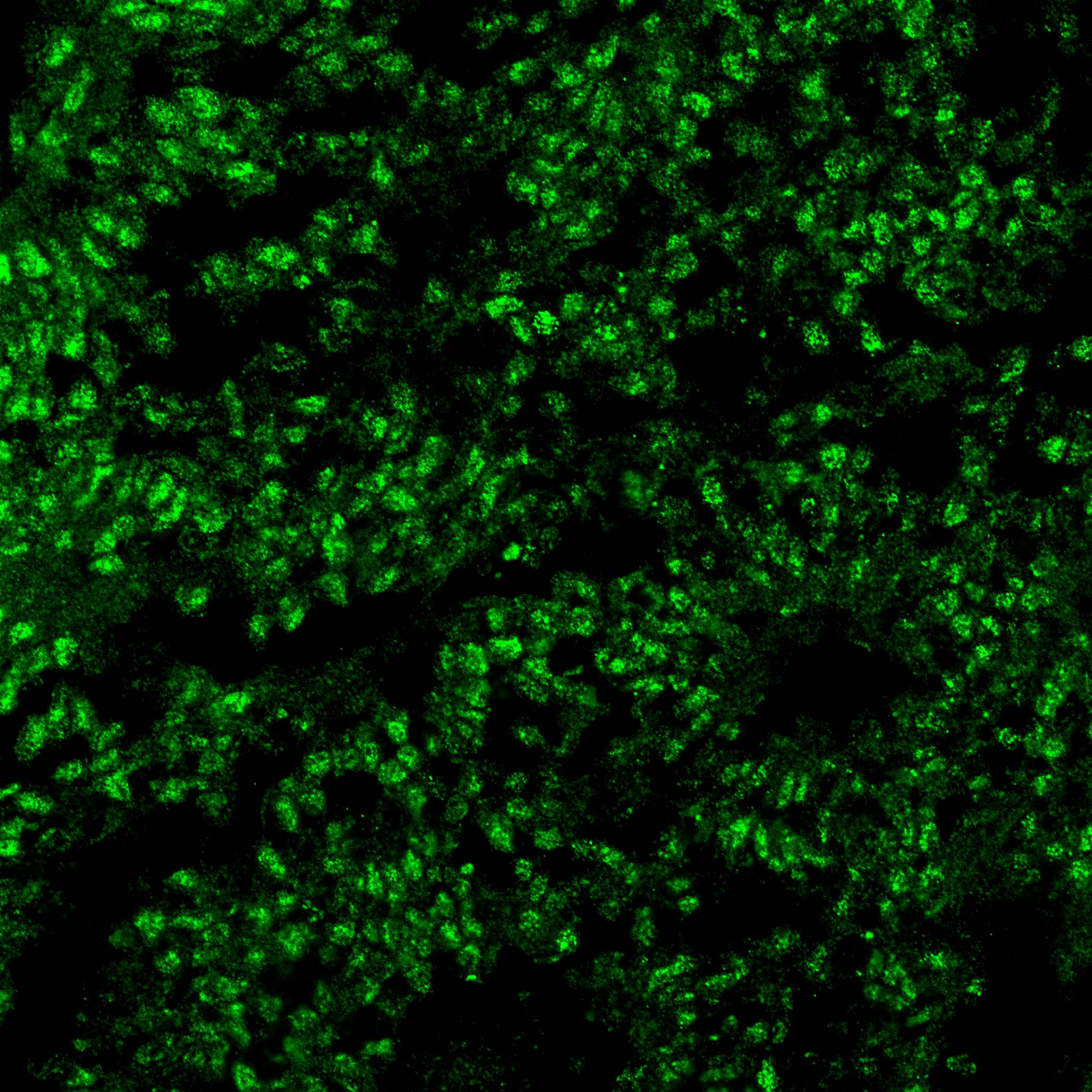

Supplement: Supplementary file 10 — Appendix Figure Source Data [file 44321_2025_206_MOESM10_ESM.zip › Appendix Figures Source Data/Appendix Fig. S15/LN5P45-OGD NEUN.tif]

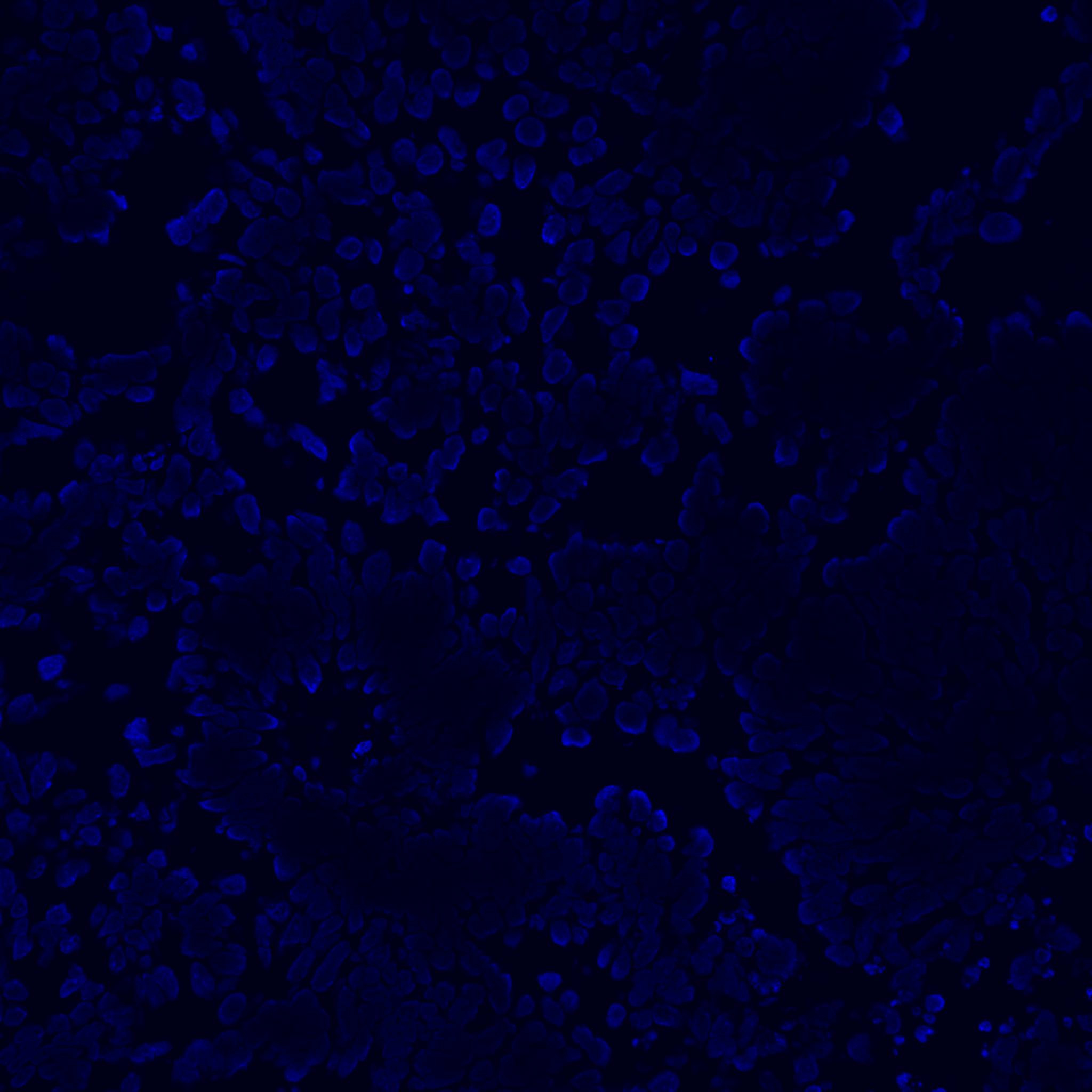

Supplement: Supplementary file 10 — Appendix Figure Source Data [file 44321_2025_206_MOESM10_ESM.zip › Appendix Figures Source Data/Appendix Fig. S15/WT-Normoxia DAPI.tif]

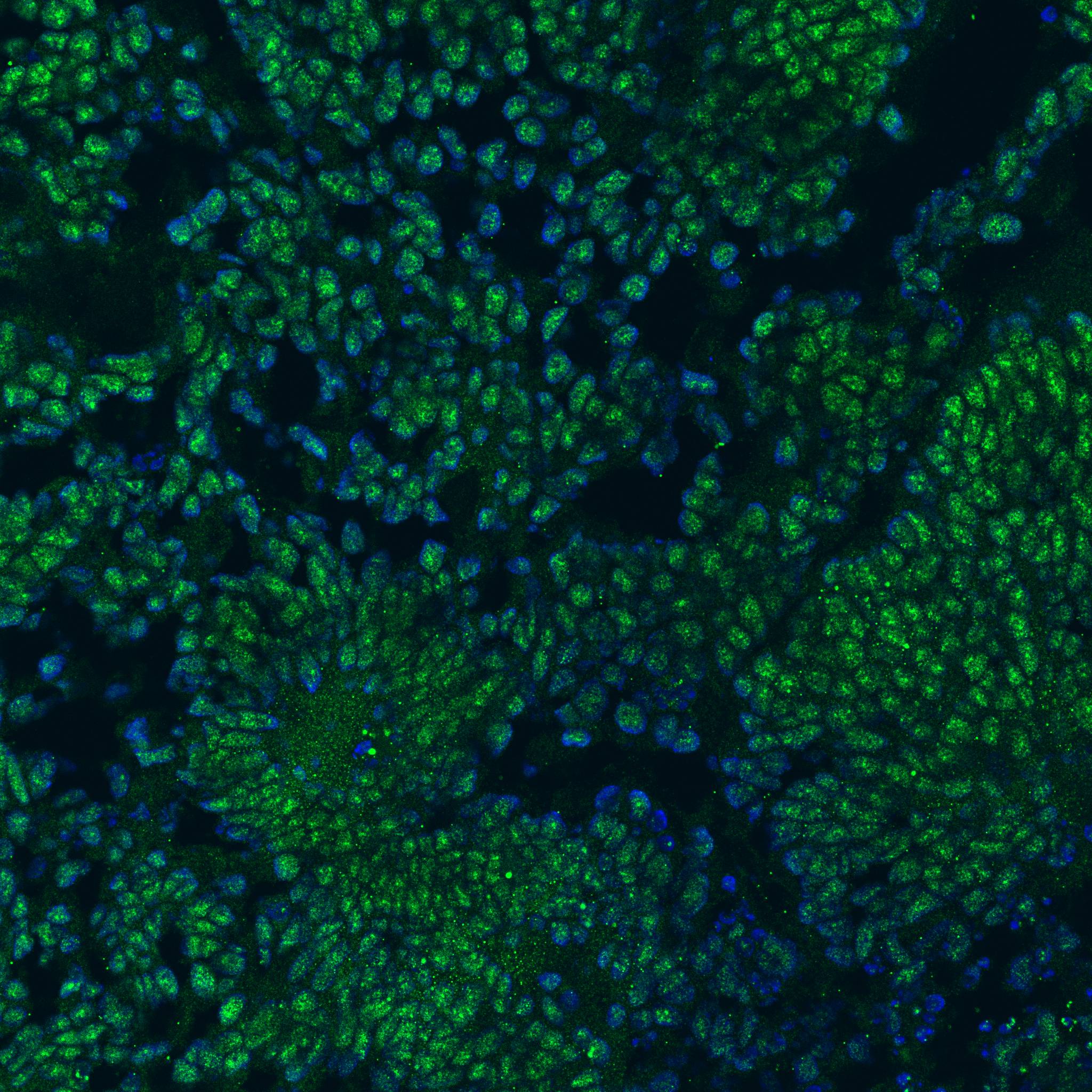

Supplement: Supplementary file 10 — Appendix Figure Source Data [file 44321_2025_206_MOESM10_ESM.zip › Appendix Figures Source Data/Appendix Fig. S15/WT-Normoxia MERGE.tif]

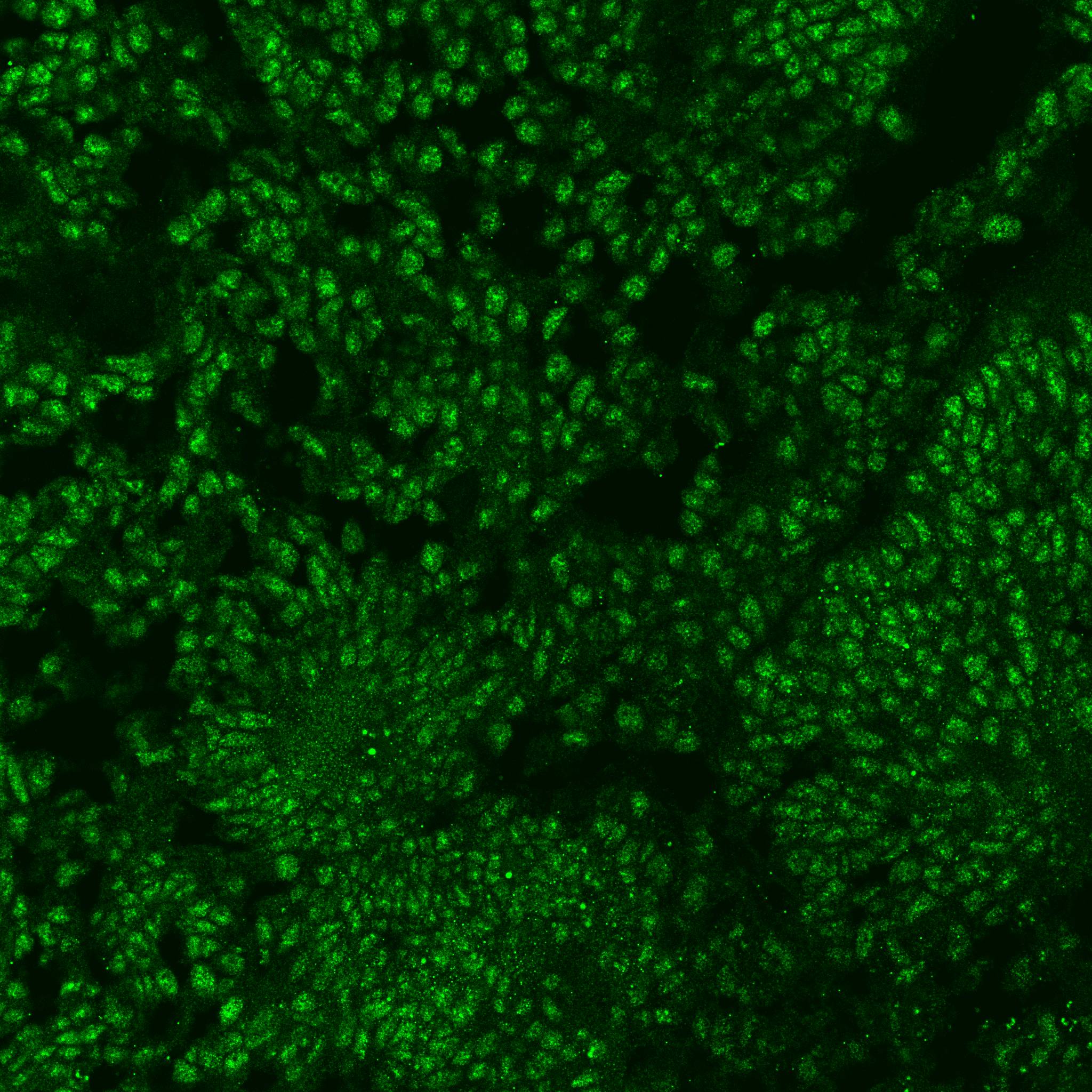

Supplement: Supplementary file 10 — Appendix Figure Source Data [file 44321_2025_206_MOESM10_ESM.zip › Appendix Figures Source Data/Appendix Fig. S15/WT-Normoxia-NEUN.tif]

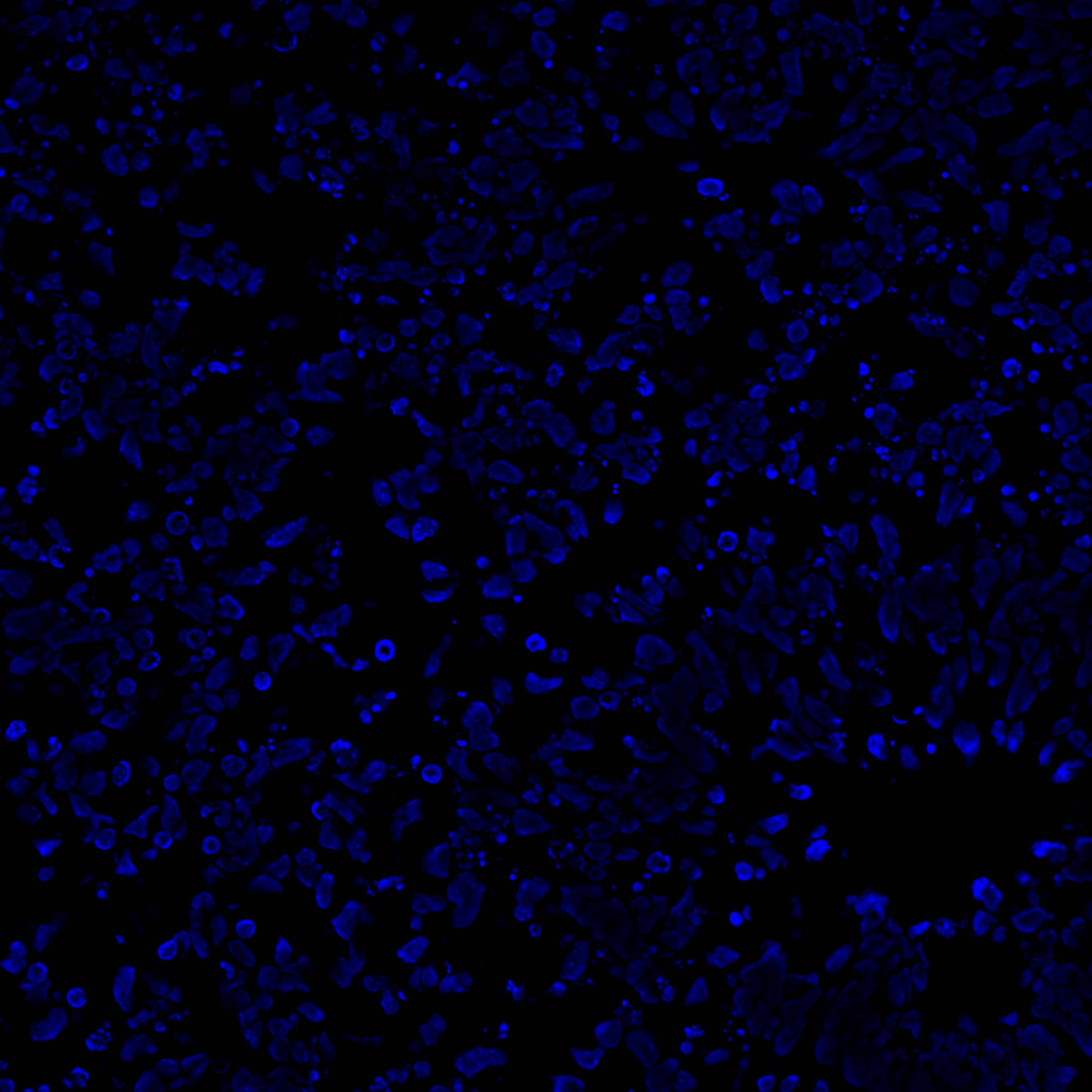

Supplement: Supplementary file 10 — Appendix Figure Source Data [file 44321_2025_206_MOESM10_ESM.zip › Appendix Figures Source Data/Appendix Fig. S15/WT-OGD DAPI.tif]

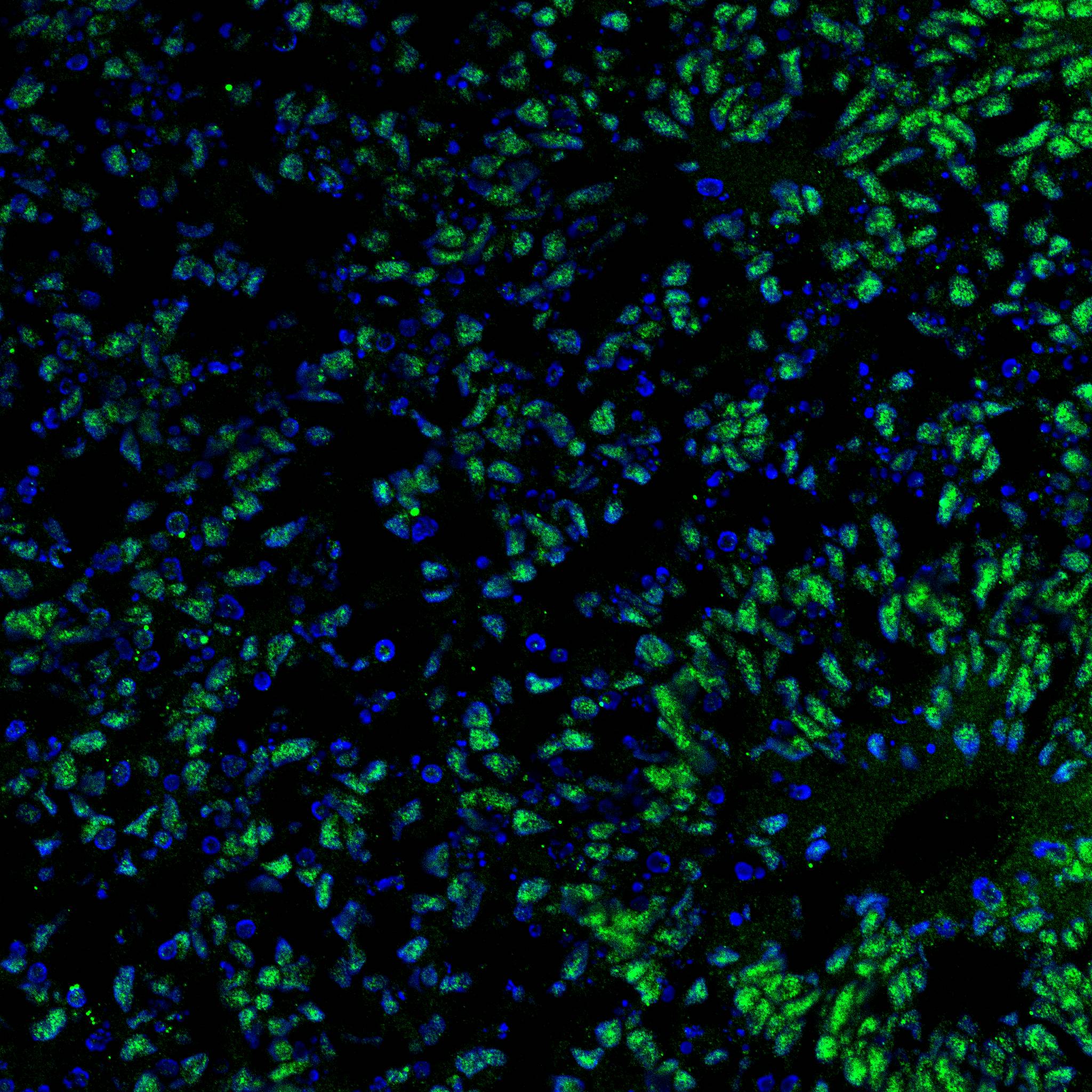

Supplement: Supplementary file 10 — Appendix Figure Source Data [file 44321_2025_206_MOESM10_ESM.zip › Appendix Figures Source Data/Appendix Fig. S15/WT-OGD MERGE.tif]

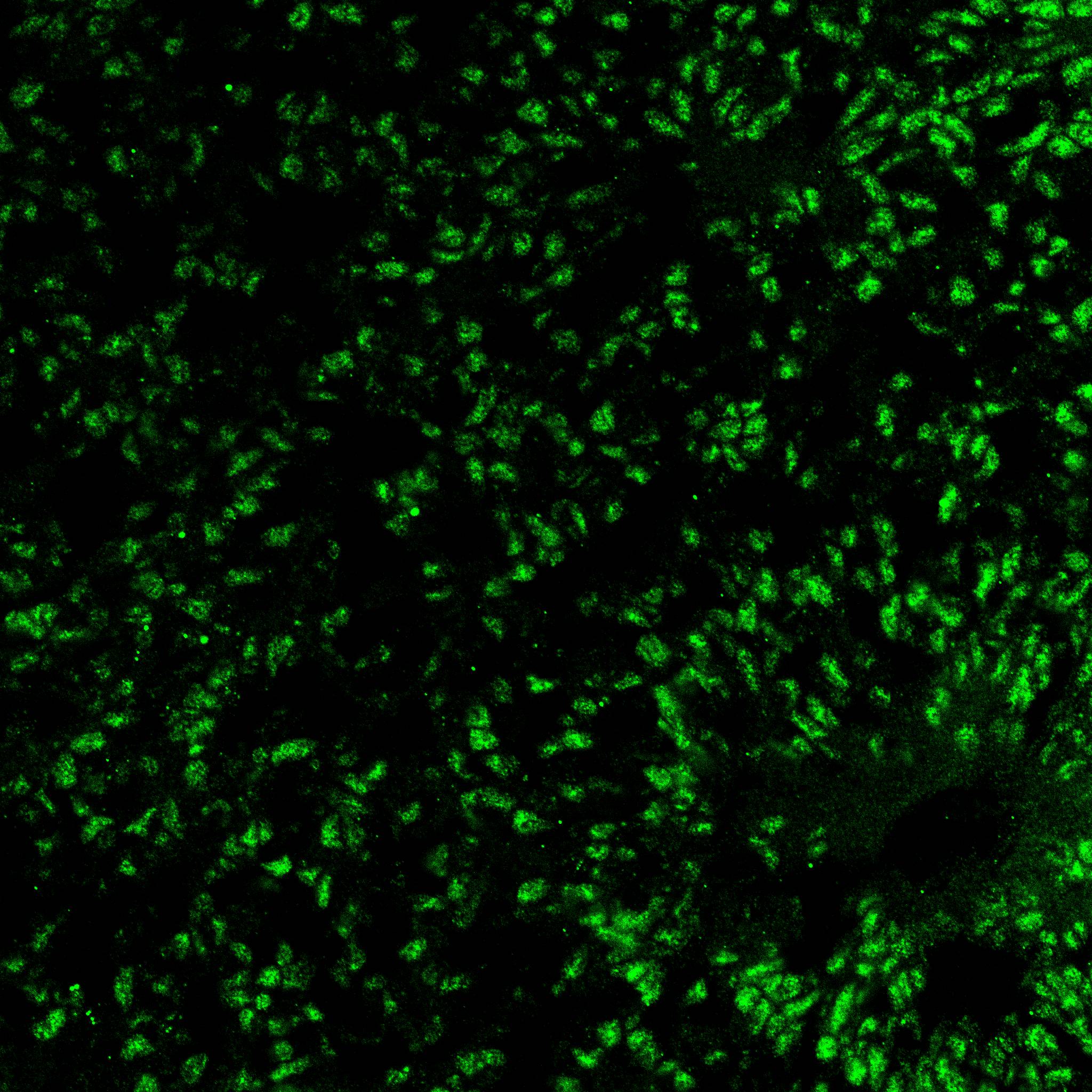

Supplement: Supplementary file 10 — Appendix Figure Source Data [file 44321_2025_206_MOESM10_ESM.zip › Appendix Figures Source Data/Appendix Fig. S15/WT-OGD NEUN.tif]

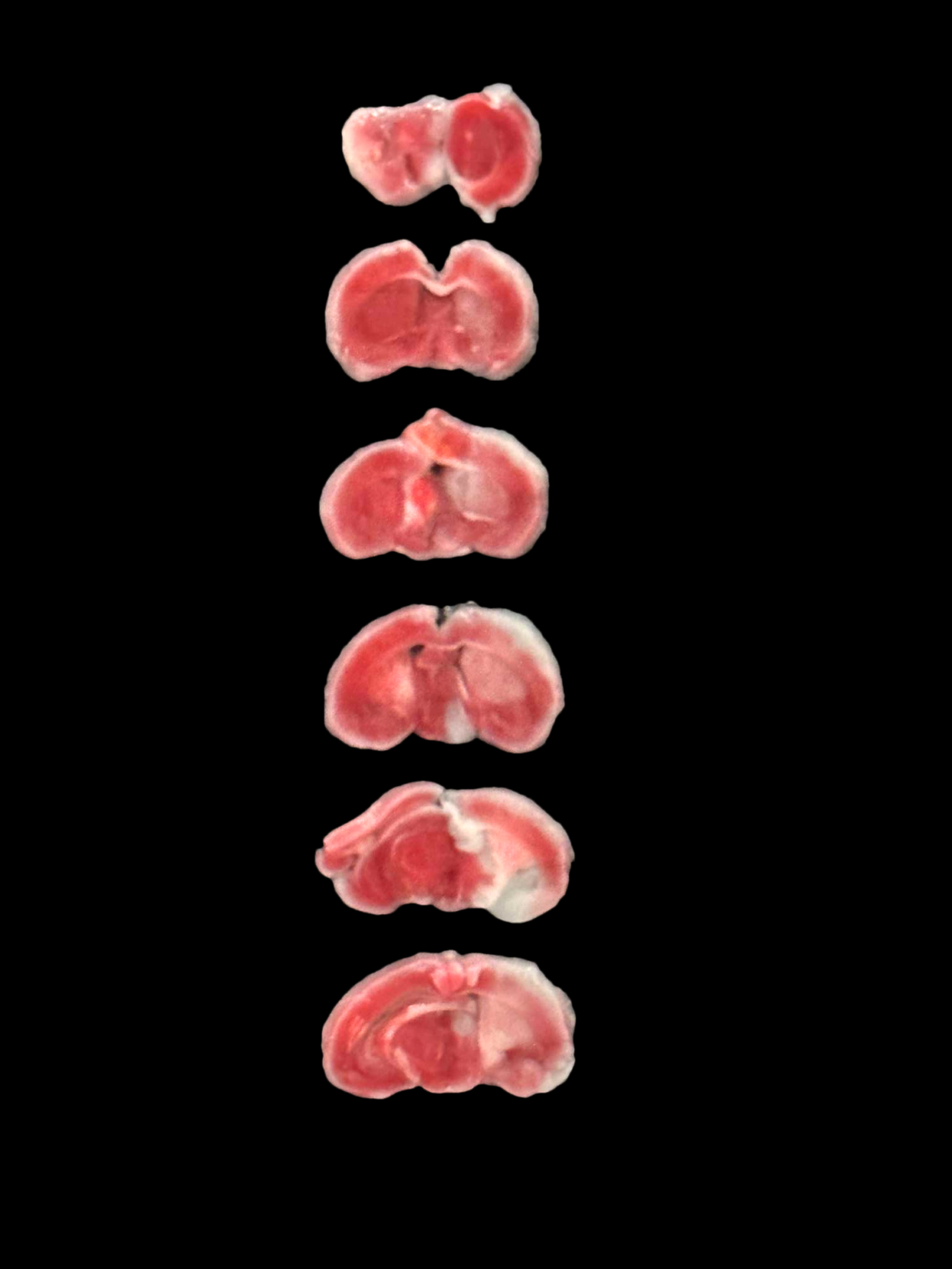

Supplement: Supplementary file 10 — Appendix Figure Source Data [file 44321_2025_206_MOESM10_ESM.zip › Appendix Figures Source Data/Appendix Fig. S2/KO-12M.tif]

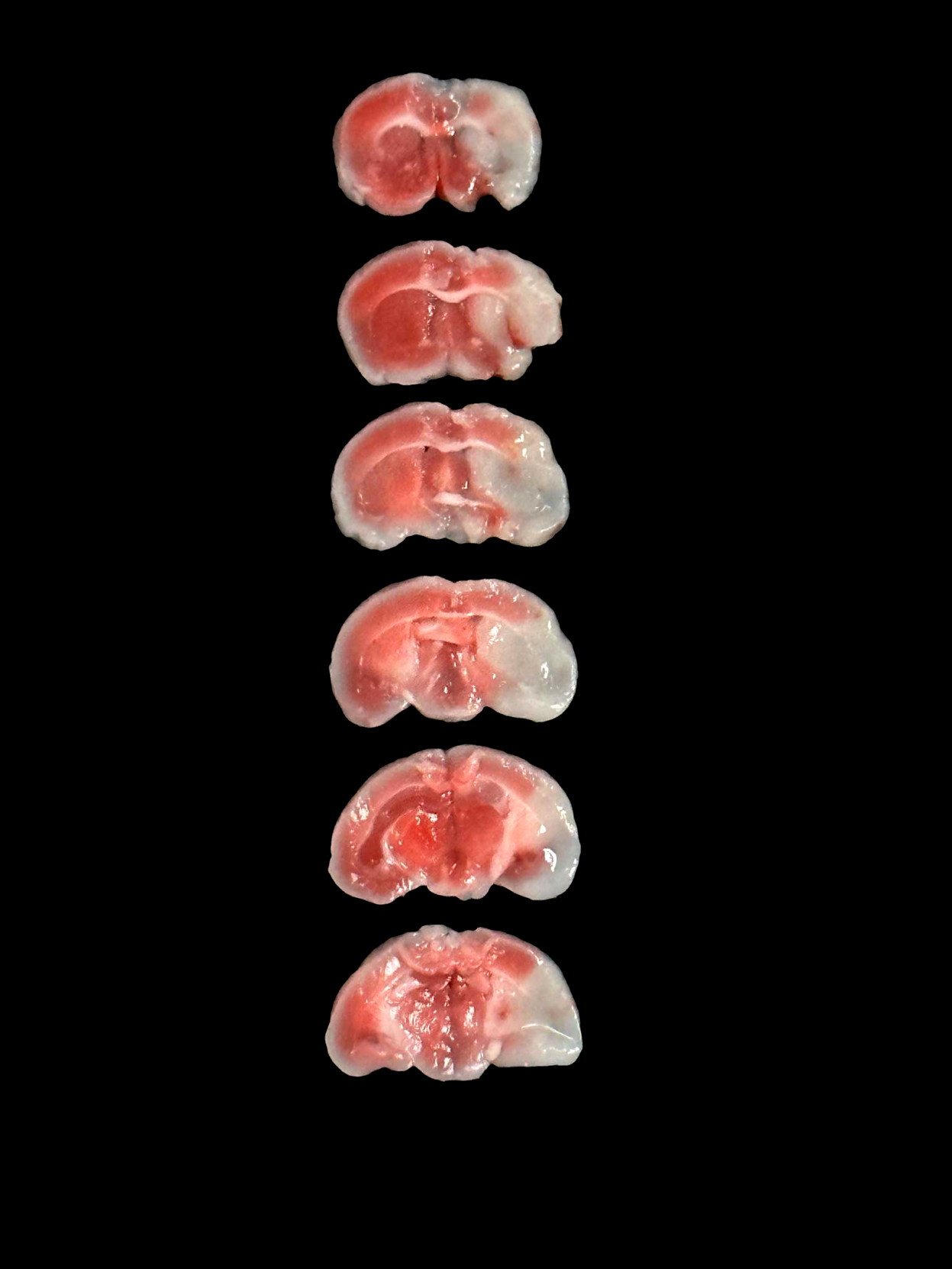

Supplement: Supplementary file 10 — Appendix Figure Source Data [file 44321_2025_206_MOESM10_ESM.zip › Appendix Figures Source Data/Appendix Fig. S2/WT-12M.tif]

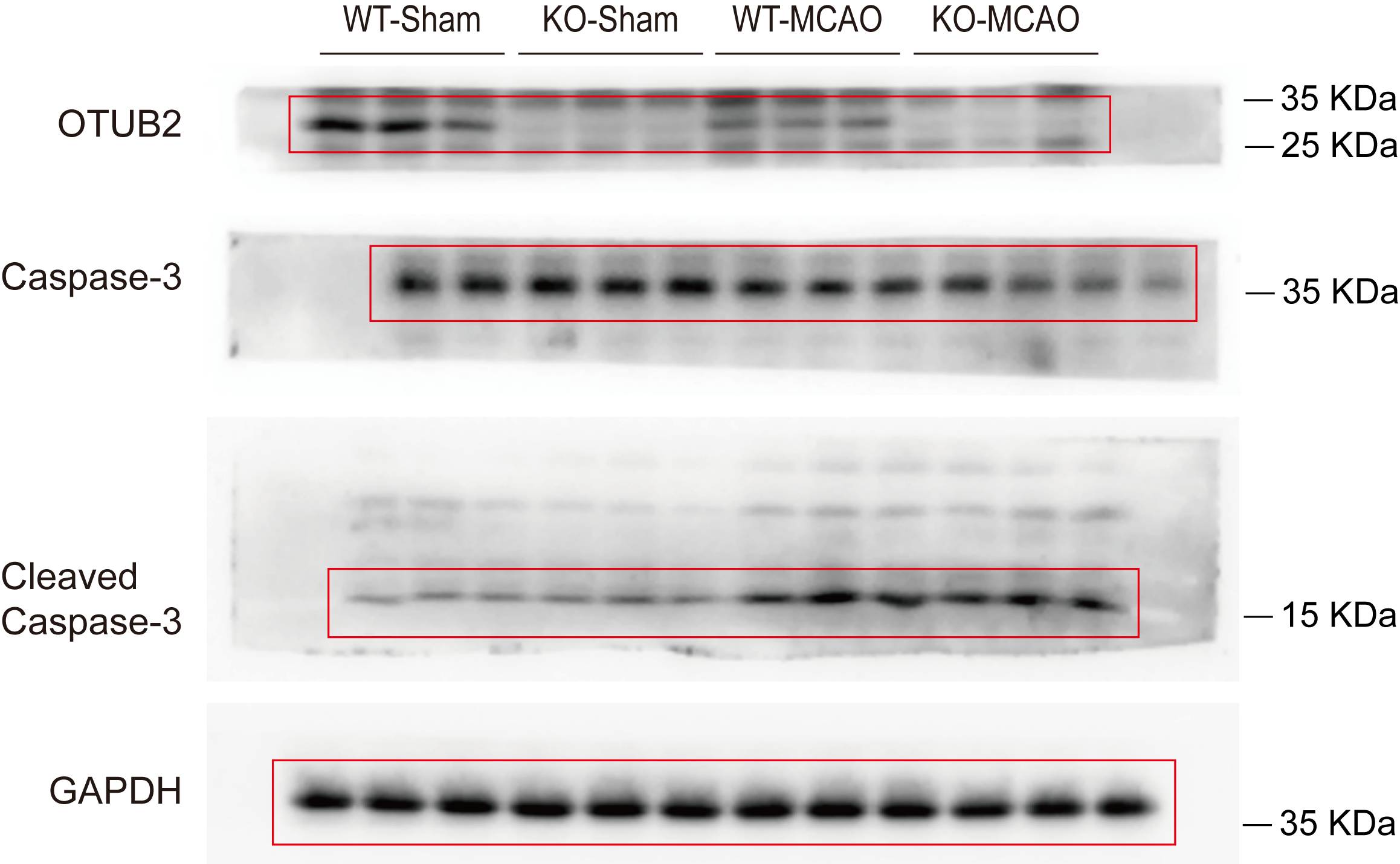

Supplement: Supplementary file 10 — Appendix Figure Source Data [file 44321_2025_206_MOESM10_ESM.zip › Appendix Figures Source Data/Appendix Fig. S4/S4-B/S4-B.tif]

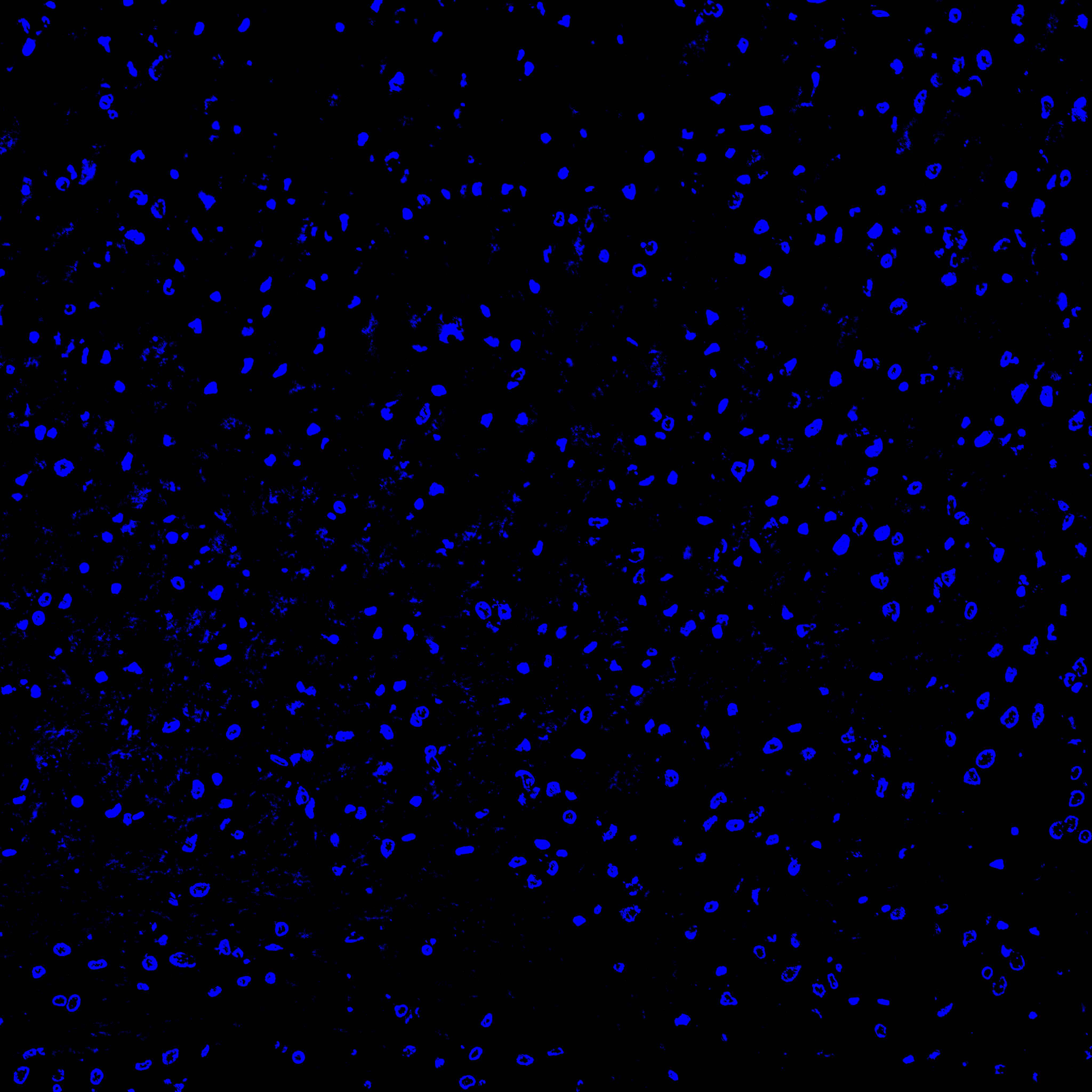

Supplement: Supplementary file 10 — Appendix Figure Source Data [file 44321_2025_206_MOESM10_ESM.zip › Appendix Figures Source Data/Appendix Fig. S4/S4-D/KO-MCAO DAPI.tif]

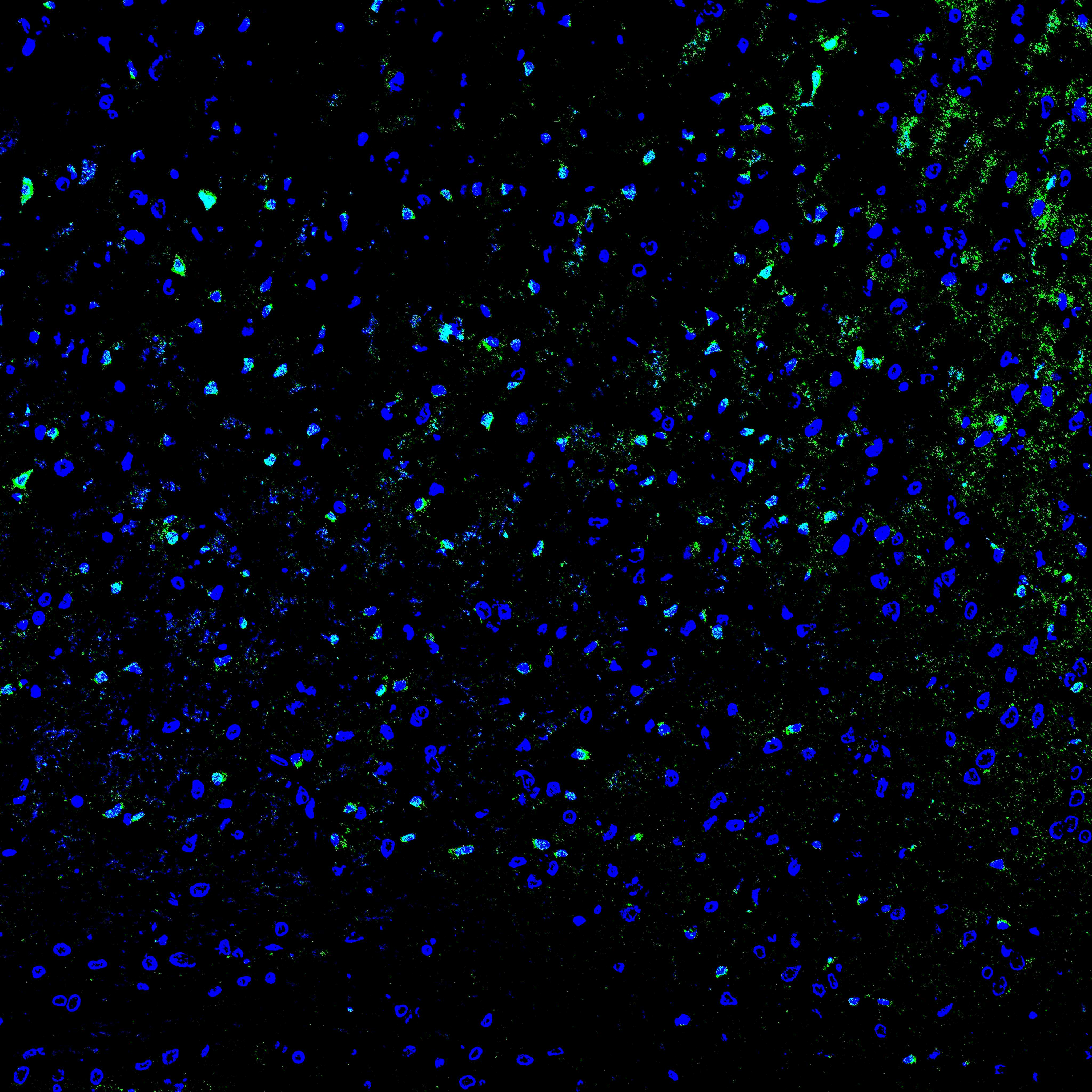

Supplement: Supplementary file 10 — Appendix Figure Source Data [file 44321_2025_206_MOESM10_ESM.zip › Appendix Figures Source Data/Appendix Fig. S4/S4-D/KO-MCAO Merge.tif]

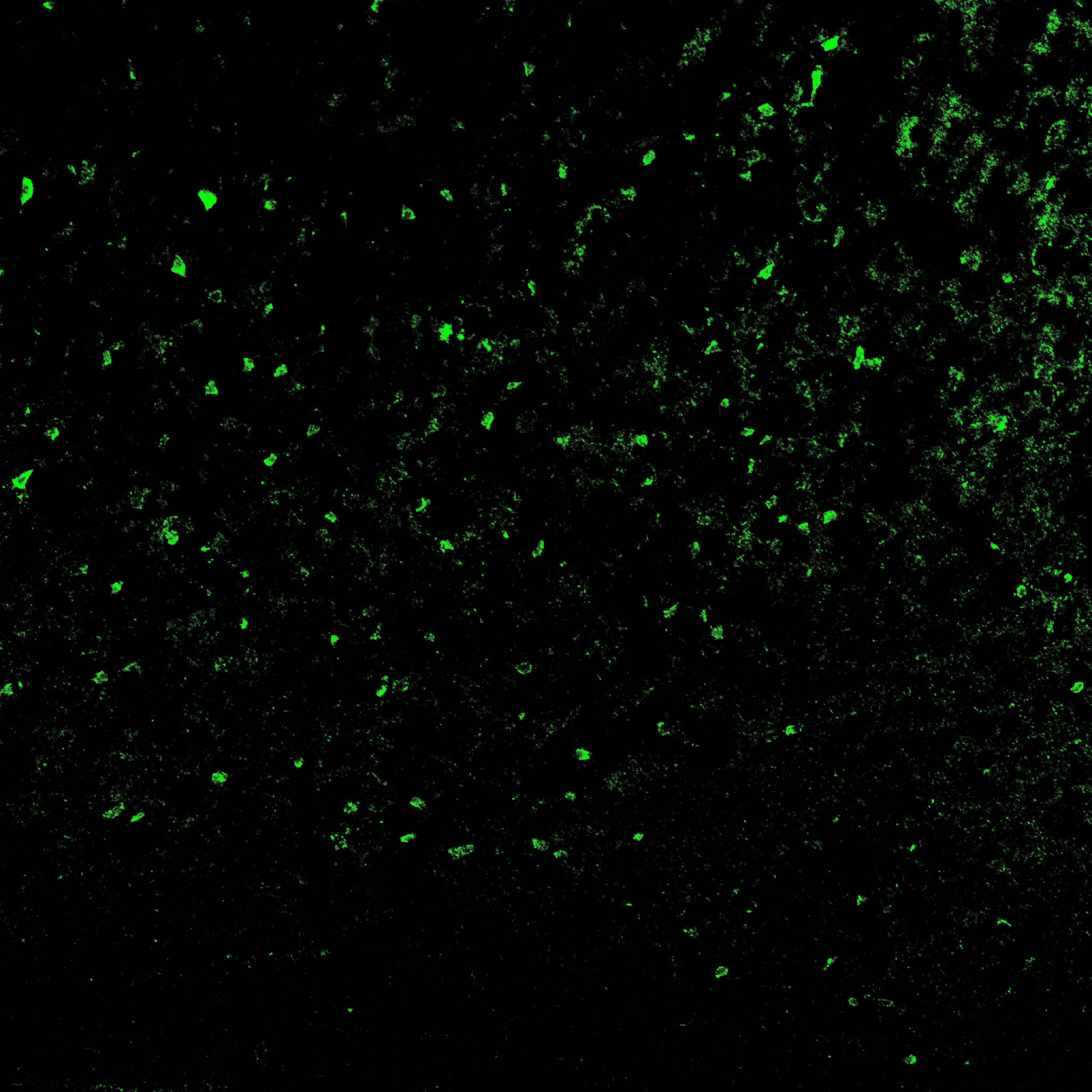

Supplement: Supplementary file 10 — Appendix Figure Source Data [file 44321_2025_206_MOESM10_ESM.zip › Appendix Figures Source Data/Appendix Fig. S4/S4-D/KO-MCAO TUNEL.tif]

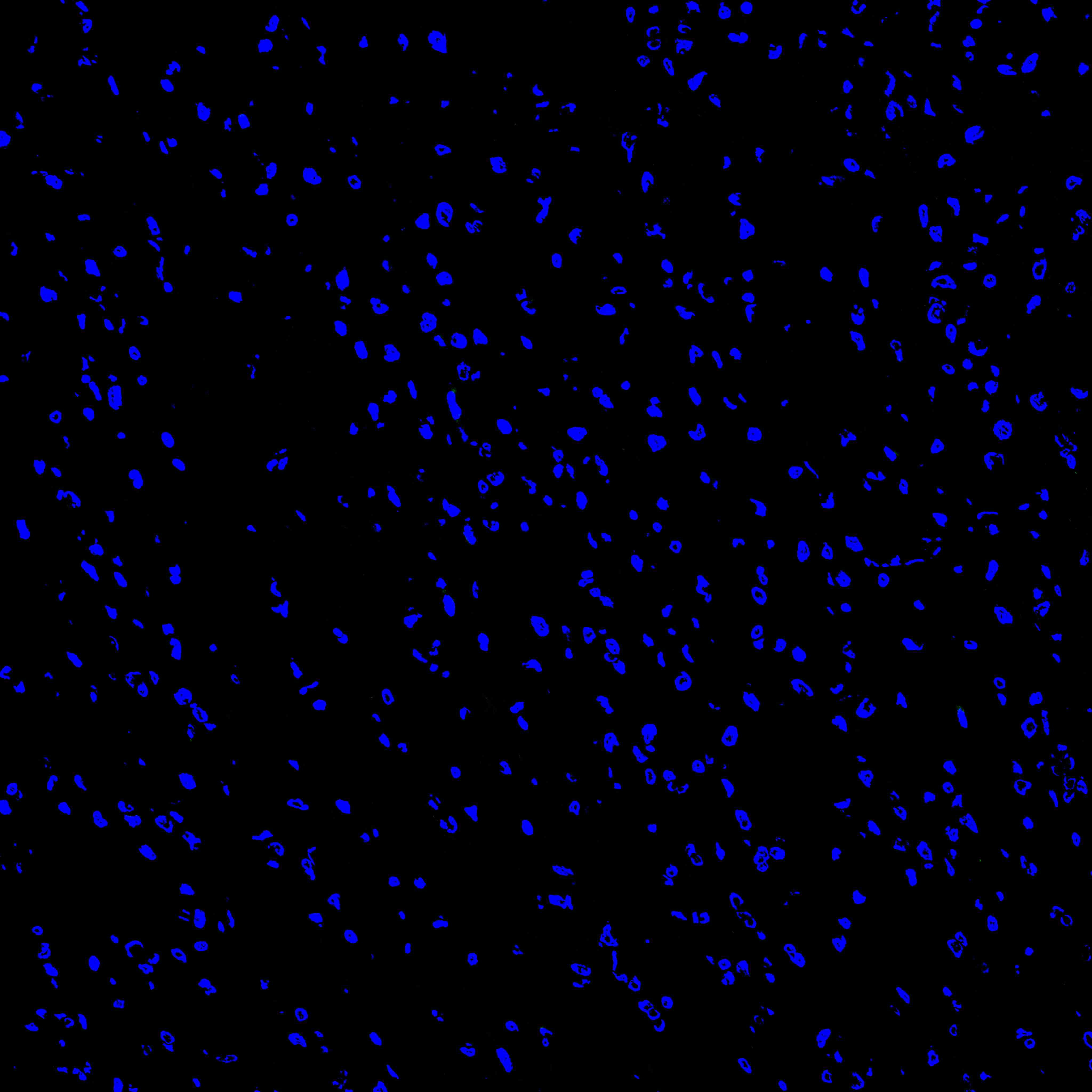

Supplement: Supplementary file 10 — Appendix Figure Source Data [file 44321_2025_206_MOESM10_ESM.zip › Appendix Figures Source Data/Appendix Fig. S4/S4-D/KO-Sham Merge.tif]

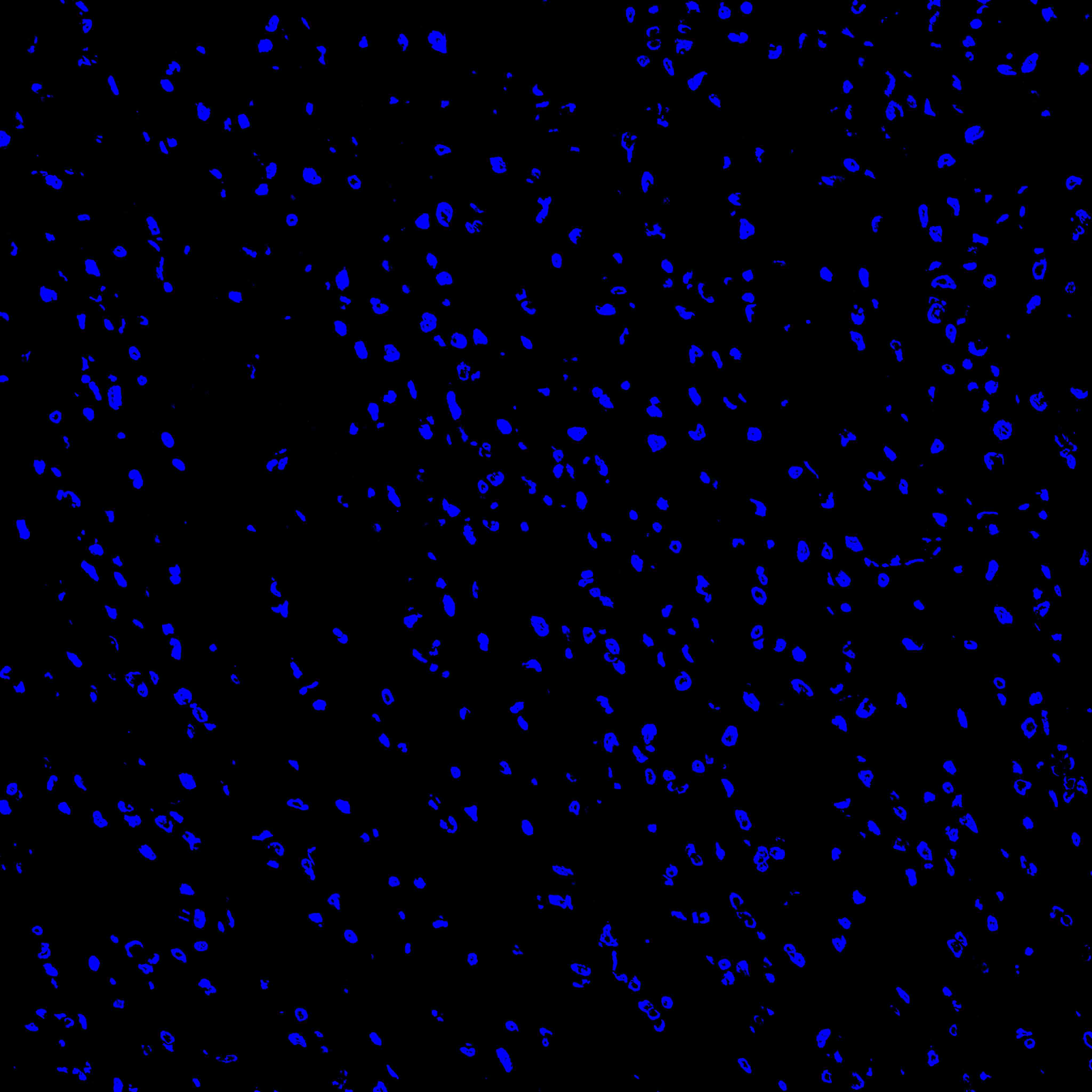

Supplement: Supplementary file 10 — Appendix Figure Source Data [file 44321_2025_206_MOESM10_ESM.zip › Appendix Figures Source Data/Appendix Fig. S4/S4-D/KO-Sham-DAPI.tif]

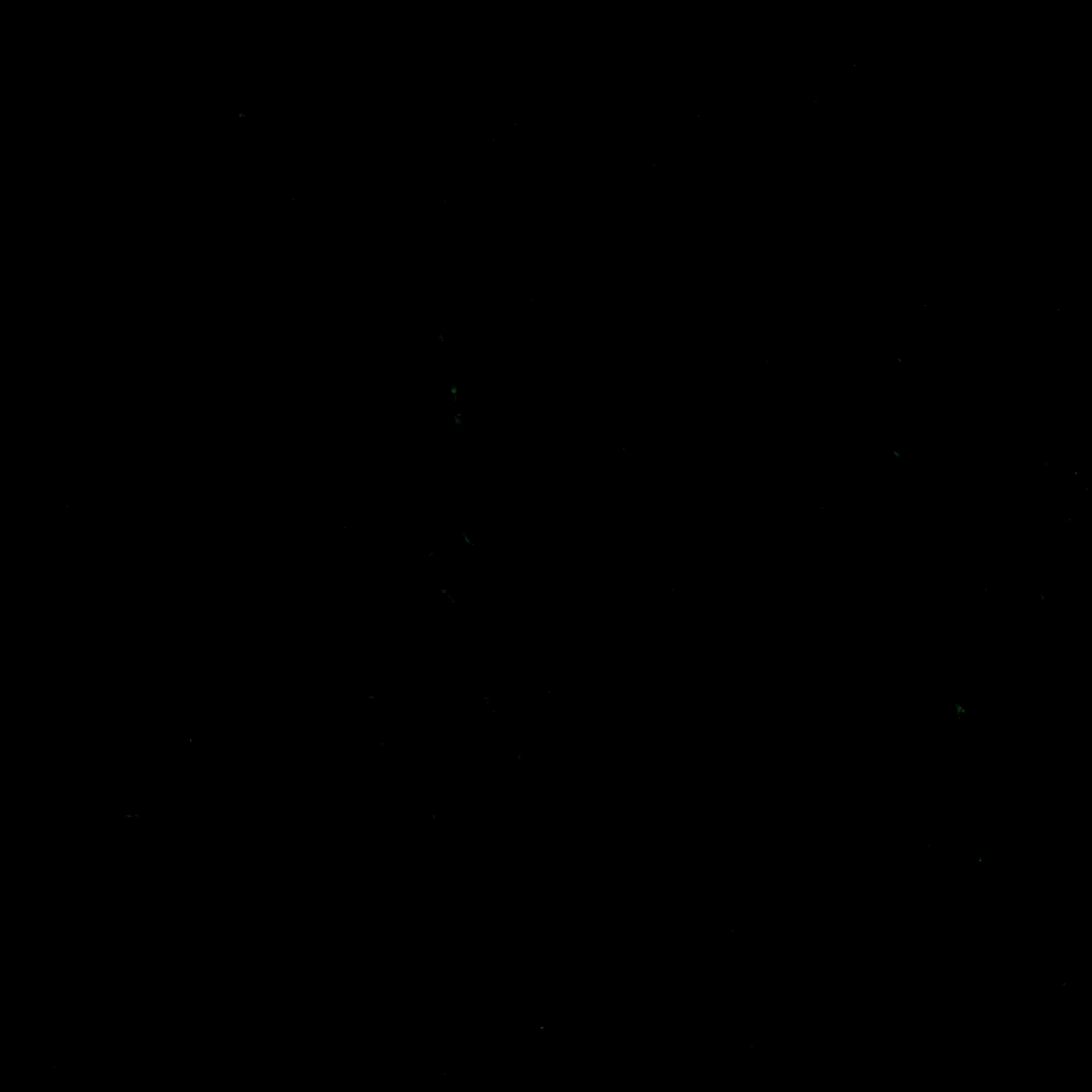

Supplement: Supplementary file 10 — Appendix Figure Source Data [file 44321_2025_206_MOESM10_ESM.zip › Appendix Figures Source Data/Appendix Fig. S4/S4-D/KO-Sham-TUNEL.tif]

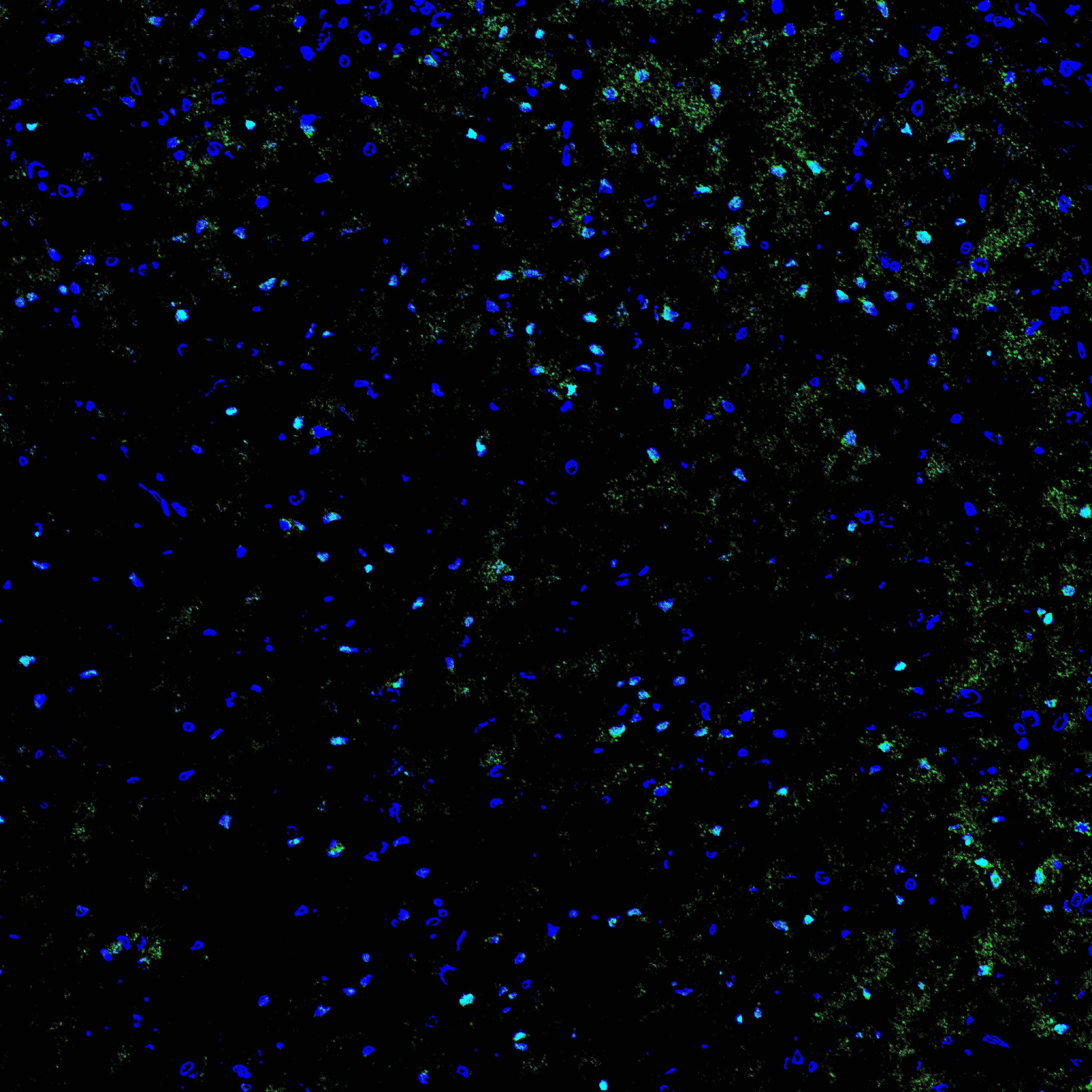

Supplement: Supplementary file 10 — Appendix Figure Source Data [file 44321_2025_206_MOESM10_ESM.zip › Appendix Figures Source Data/Appendix Fig. S4/S4-D/WT-MCAO Merge.tif]

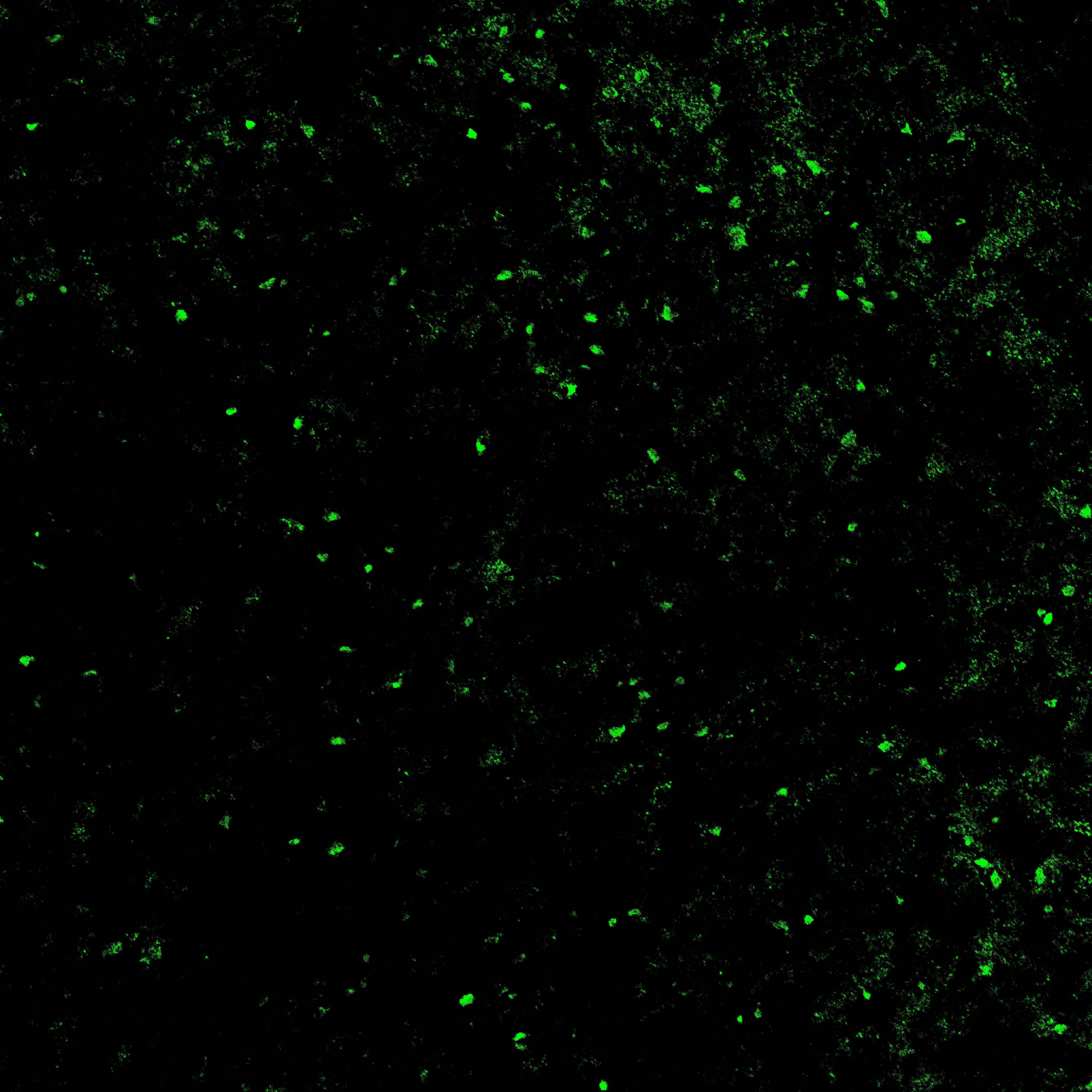

Supplement: Supplementary file 10 — Appendix Figure Source Data [file 44321_2025_206_MOESM10_ESM.zip › Appendix Figures Source Data/Appendix Fig. S4/S4-D/WT-MCAO TUNEL.tif]

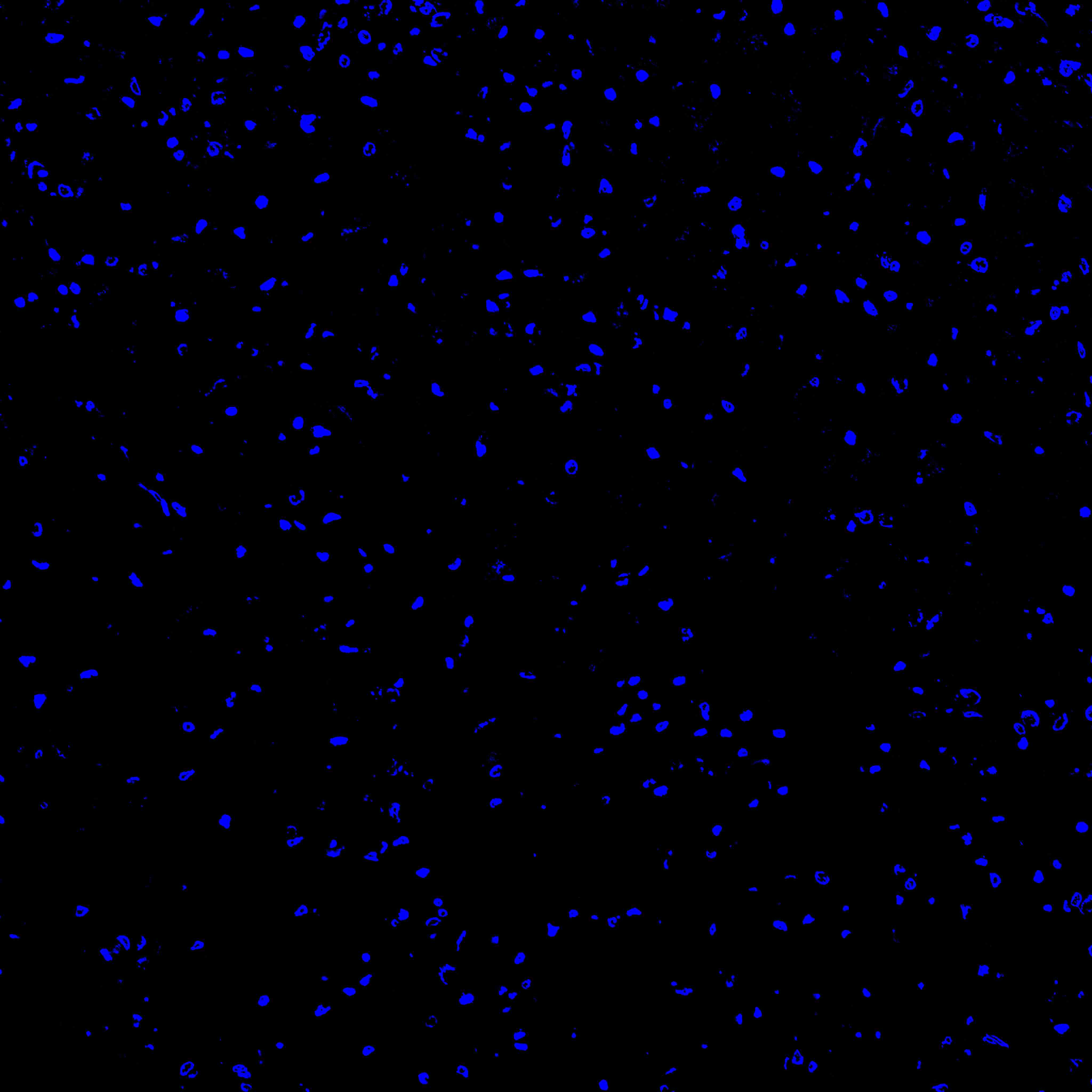

Supplement: Supplementary file 10 — Appendix Figure Source Data [file 44321_2025_206_MOESM10_ESM.zip › Appendix Figures Source Data/Appendix Fig. S4/S4-D/WT-MCAO -DAPI.tif]

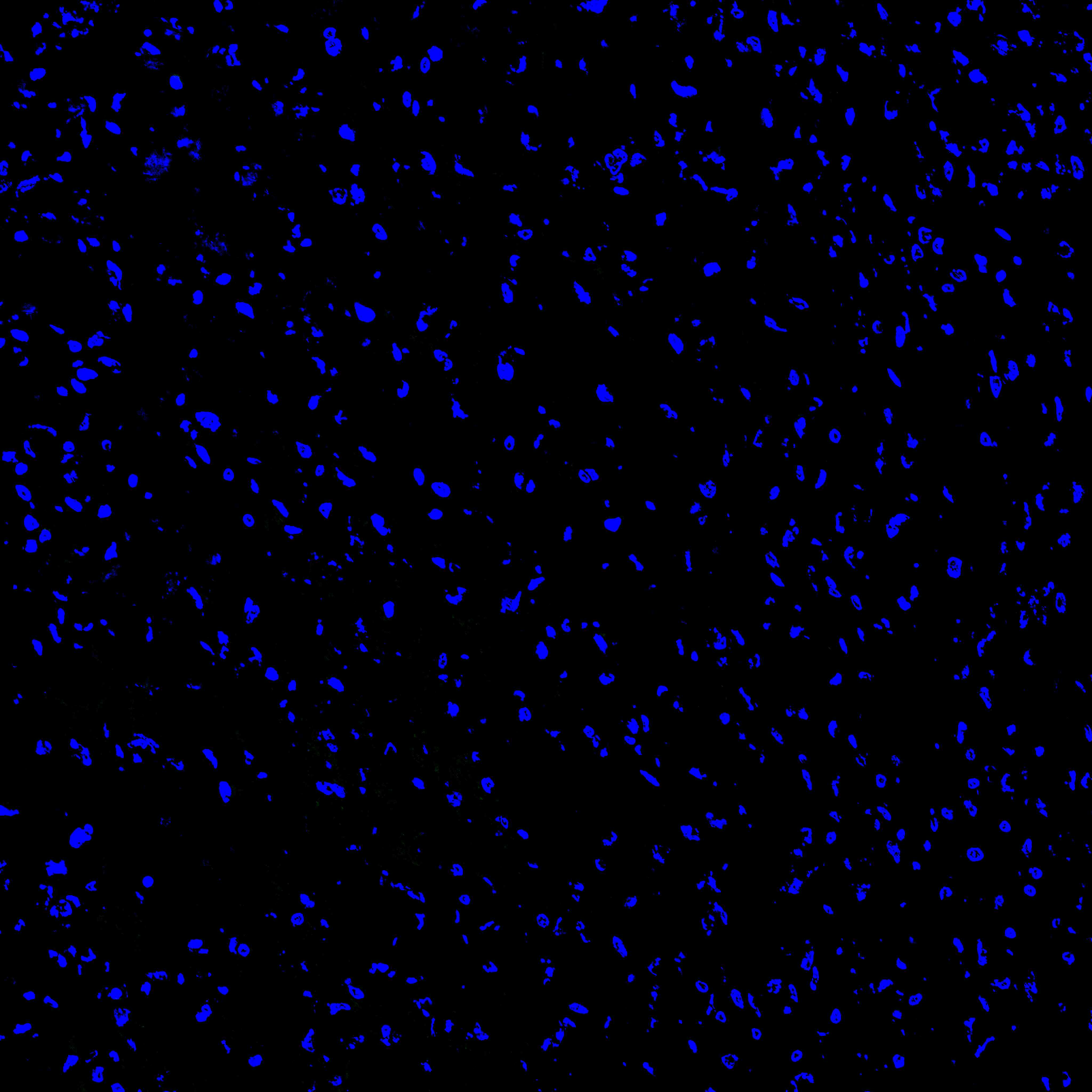

Supplement: Supplementary file 10 — Appendix Figure Source Data [file 44321_2025_206_MOESM10_ESM.zip › Appendix Figures Source Data/Appendix Fig. S4/S4-D/WT-Sham Merge.tif]

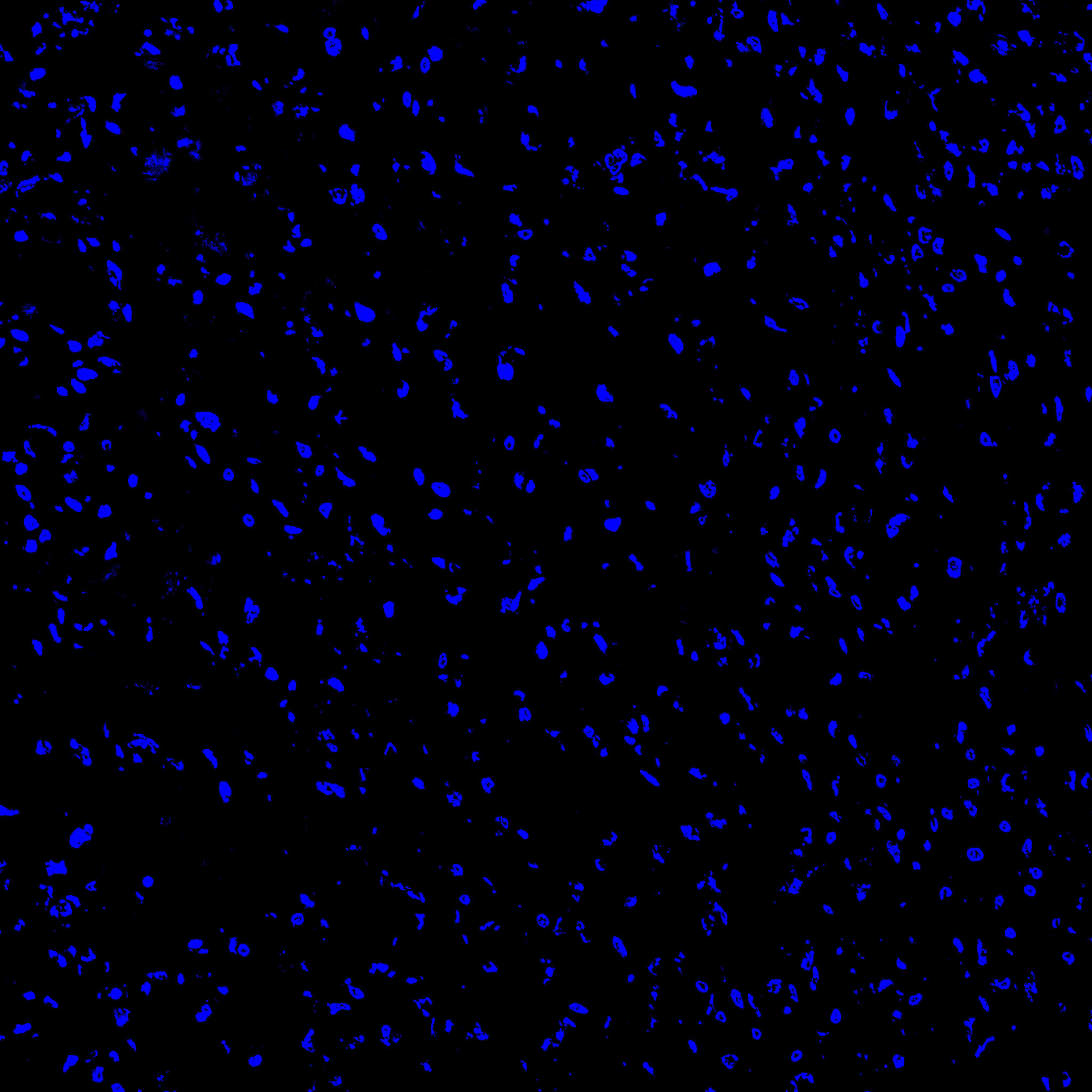

Supplement: Supplementary file 10 — Appendix Figure Source Data [file 44321_2025_206_MOESM10_ESM.zip › Appendix Figures Source Data/Appendix Fig. S4/S4-D/WT-Sham-DAPI.tif]

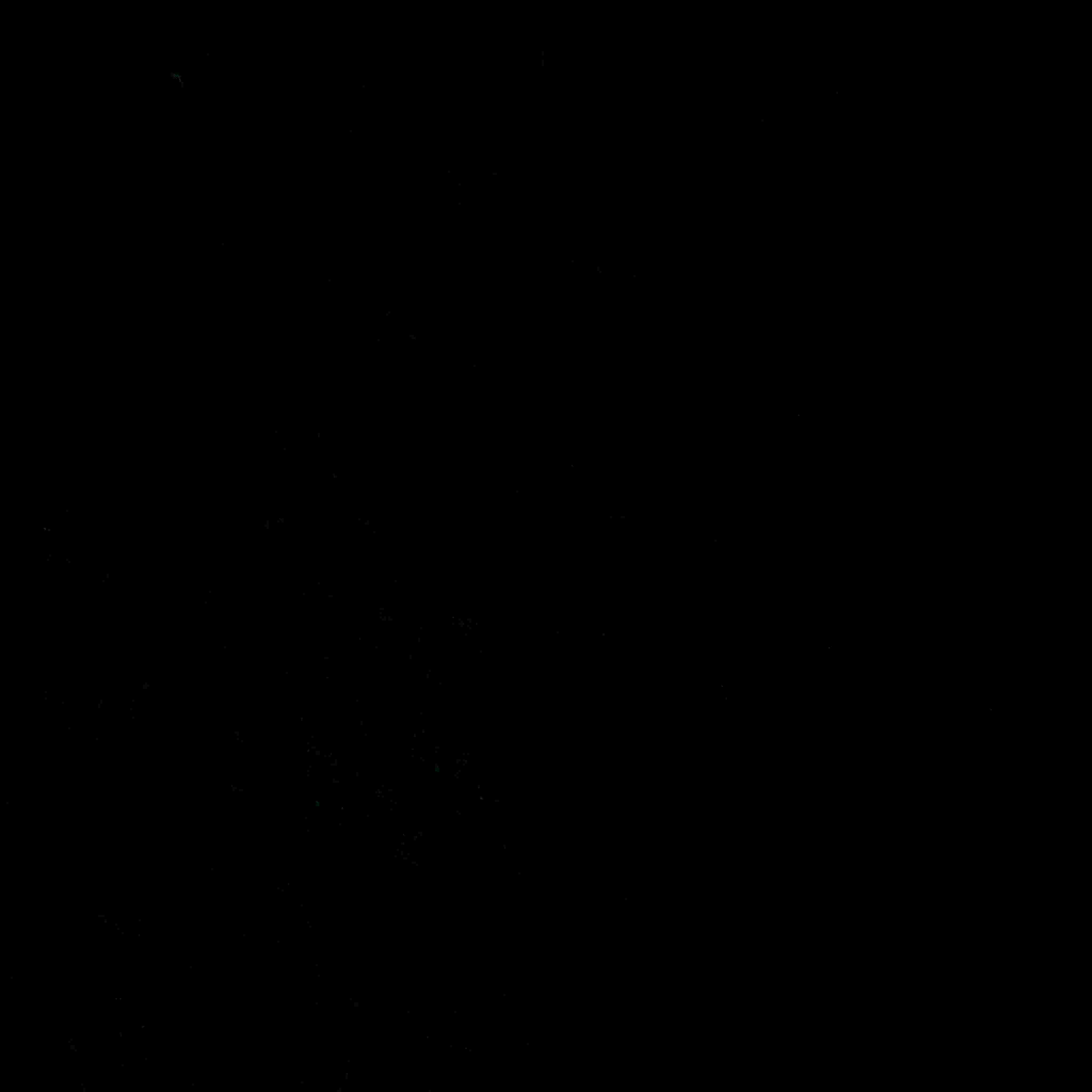

Supplement: Supplementary file 10 — Appendix Figure Source Data [file 44321_2025_206_MOESM10_ESM.zip › Appendix Figures Source Data/Appendix Fig. S4/S4-D/WT-Sham-TUNEL.tif]

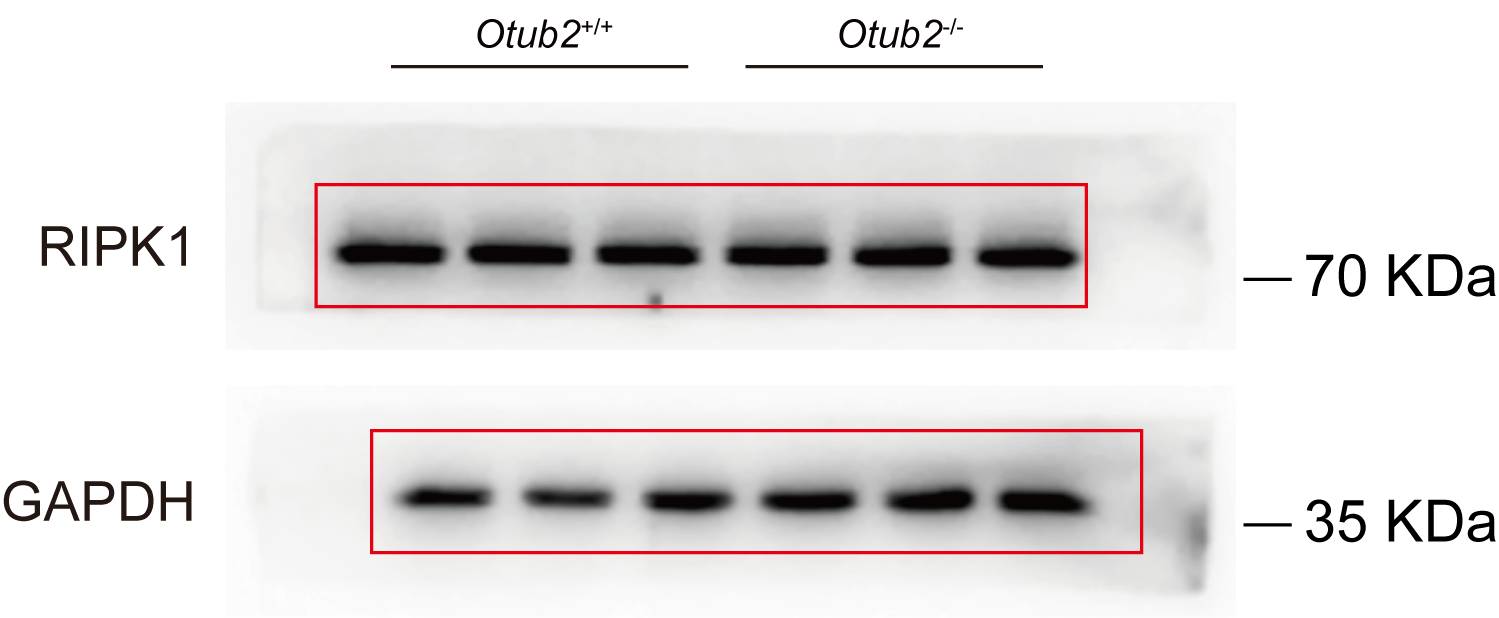

Supplement: Supplementary file 10 — Appendix Figure Source Data [file 44321_2025_206_MOESM10_ESM.zip › Appendix Figures Source Data/Appendix Fig. S6/S6-A-C/S6-A.tif]

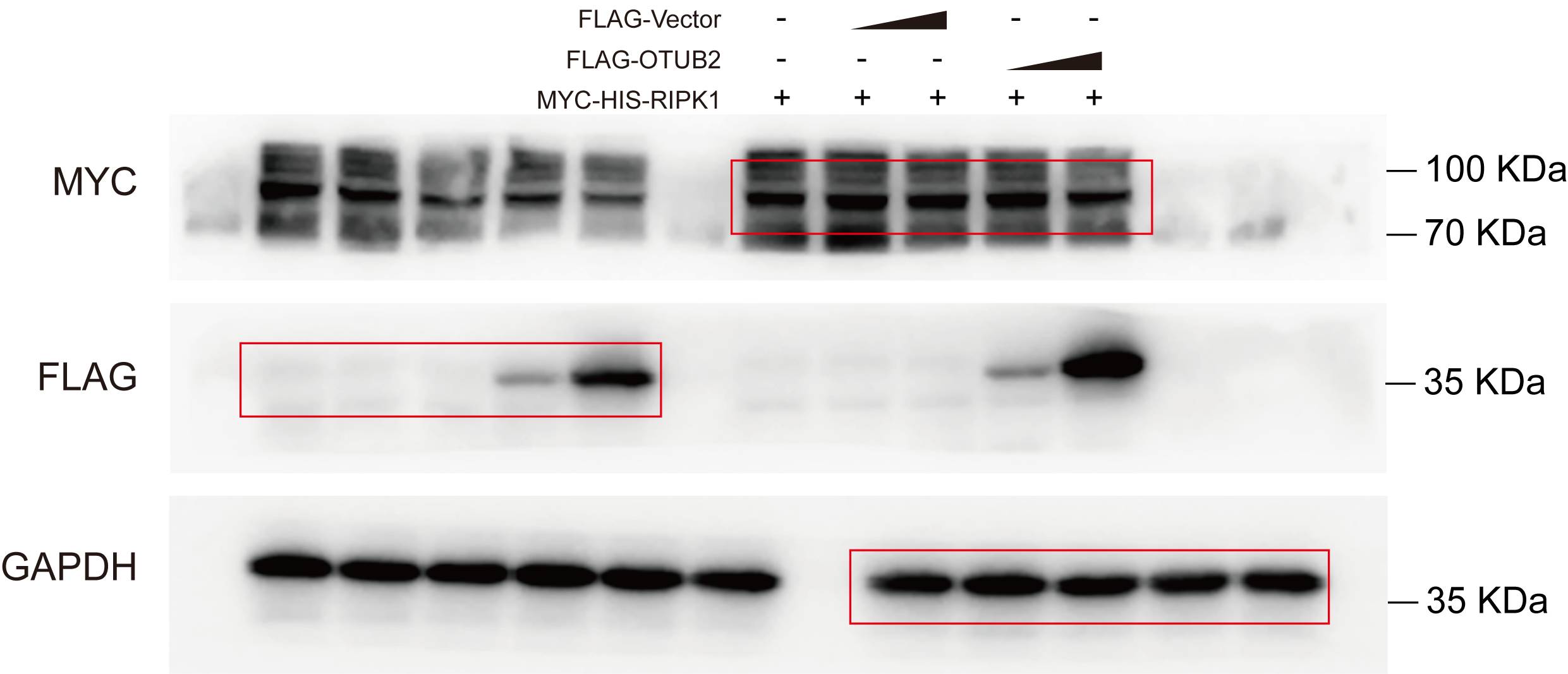

Supplement: Supplementary file 10 — Appendix Figure Source Data [file 44321_2025_206_MOESM10_ESM.zip › Appendix Figures Source Data/Appendix Fig. S6/S6-D/S6-D.tif]

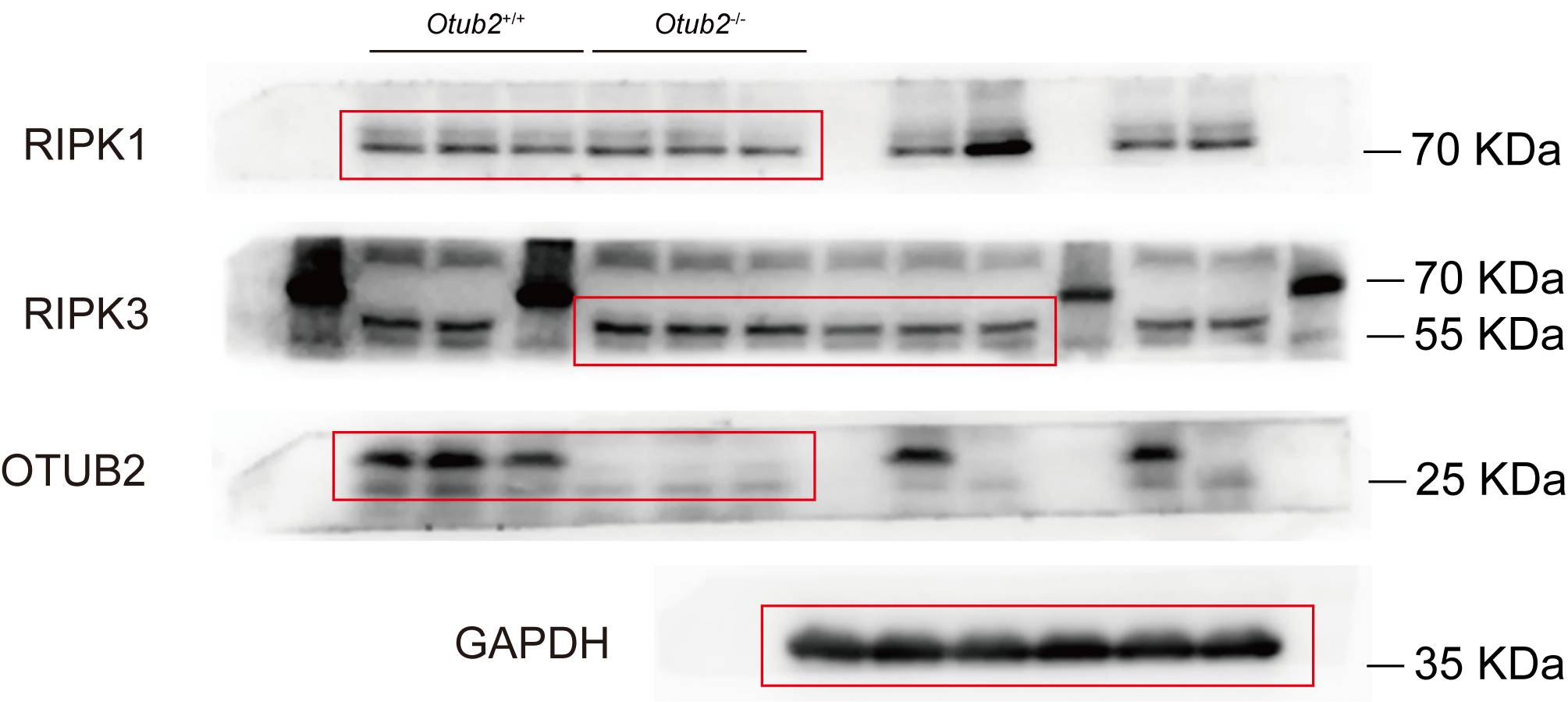

Supplement: Supplementary file 10 — Appendix Figure Source Data [file 44321_2025_206_MOESM10_ESM.zip › Appendix Figures Source Data/Appendix Fig. S6/S6-E-I/S6-E.tif]

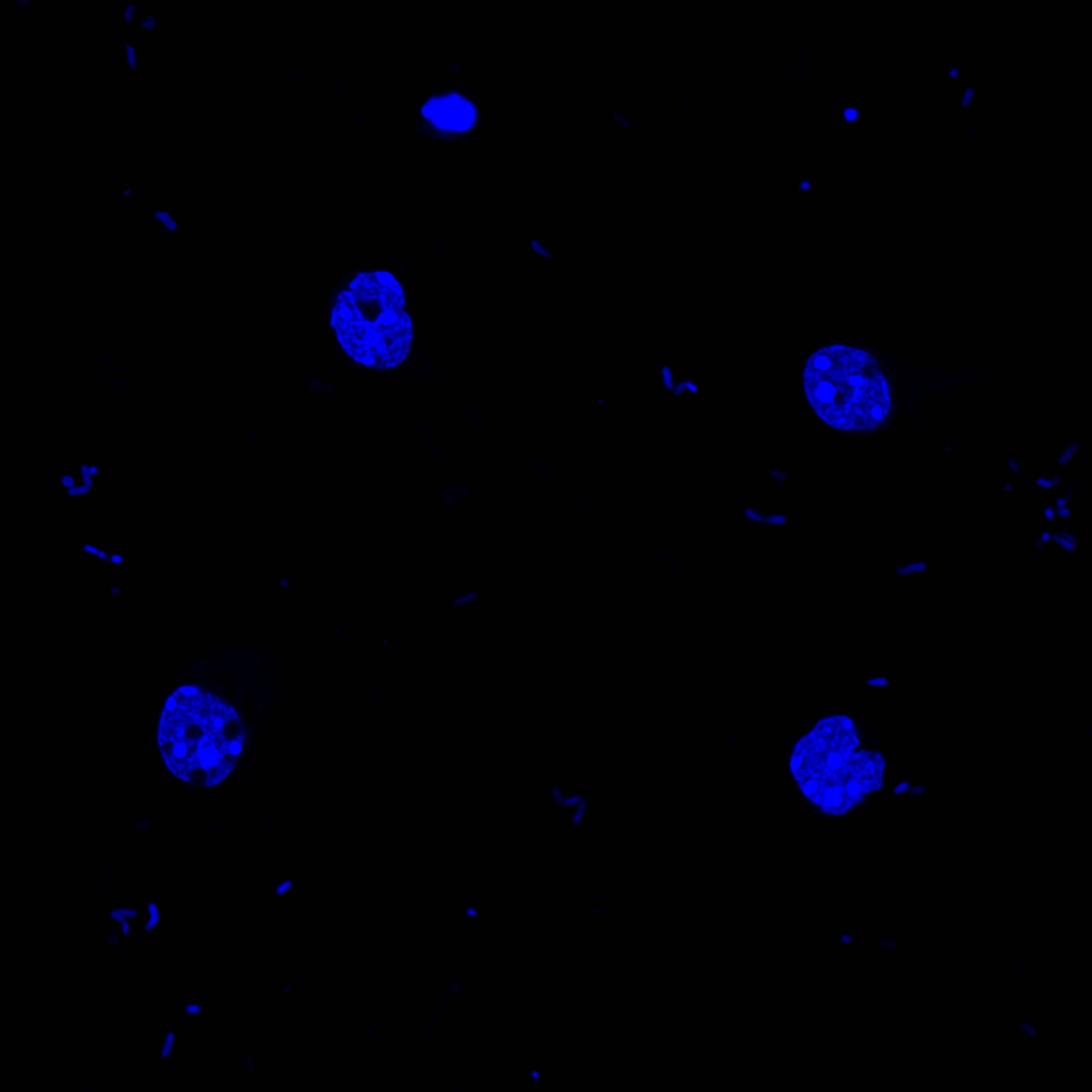

Supplement: Supplementary file 10 — Appendix Figure Source Data [file 44321_2025_206_MOESM10_ESM.zip › Appendix Figures Source Data/Appendix Fig. S7/DAPI.tif]

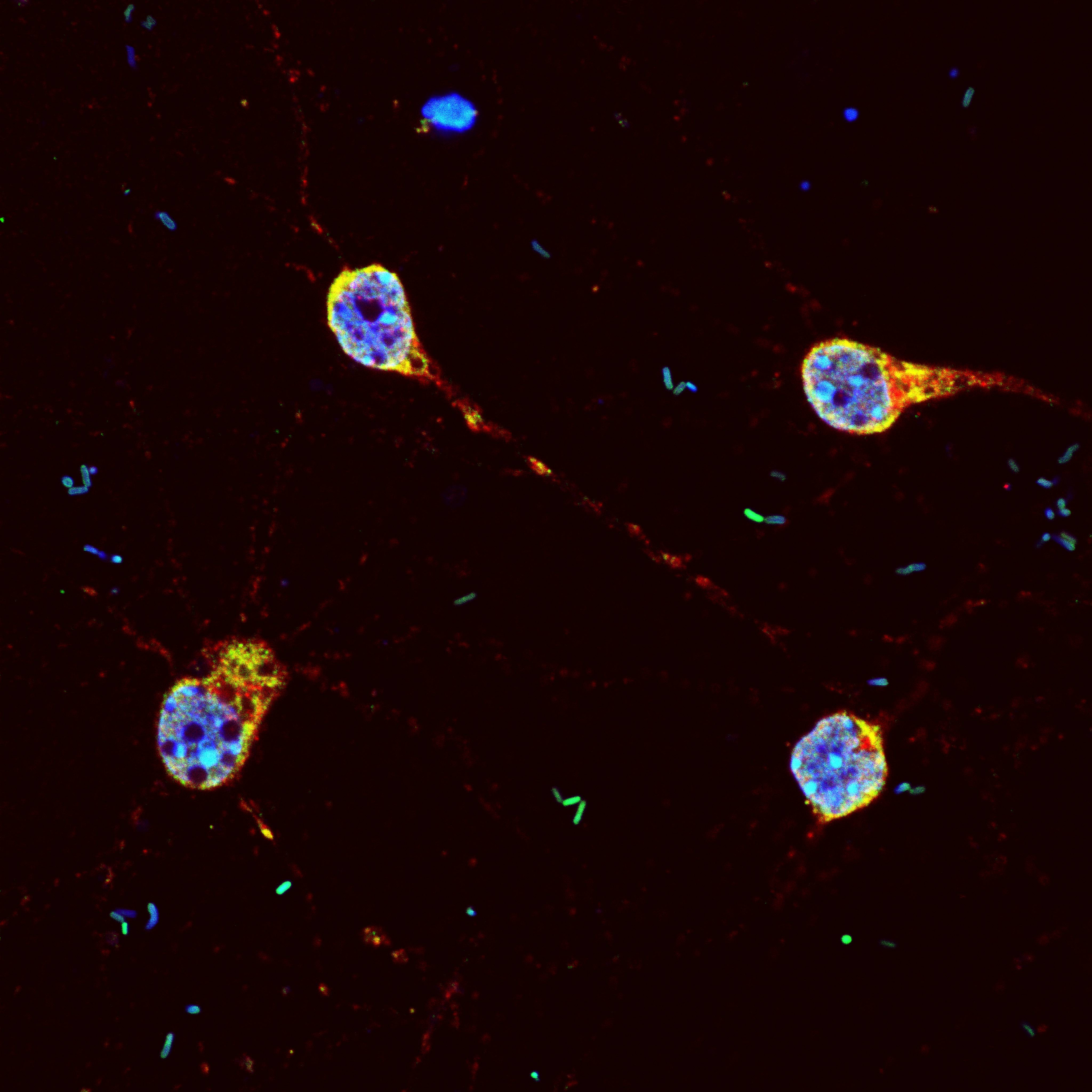

Supplement: Supplementary file 10 — Appendix Figure Source Data [file 44321_2025_206_MOESM10_ESM.zip › Appendix Figures Source Data/Appendix Fig. S7/Merge.jpg]

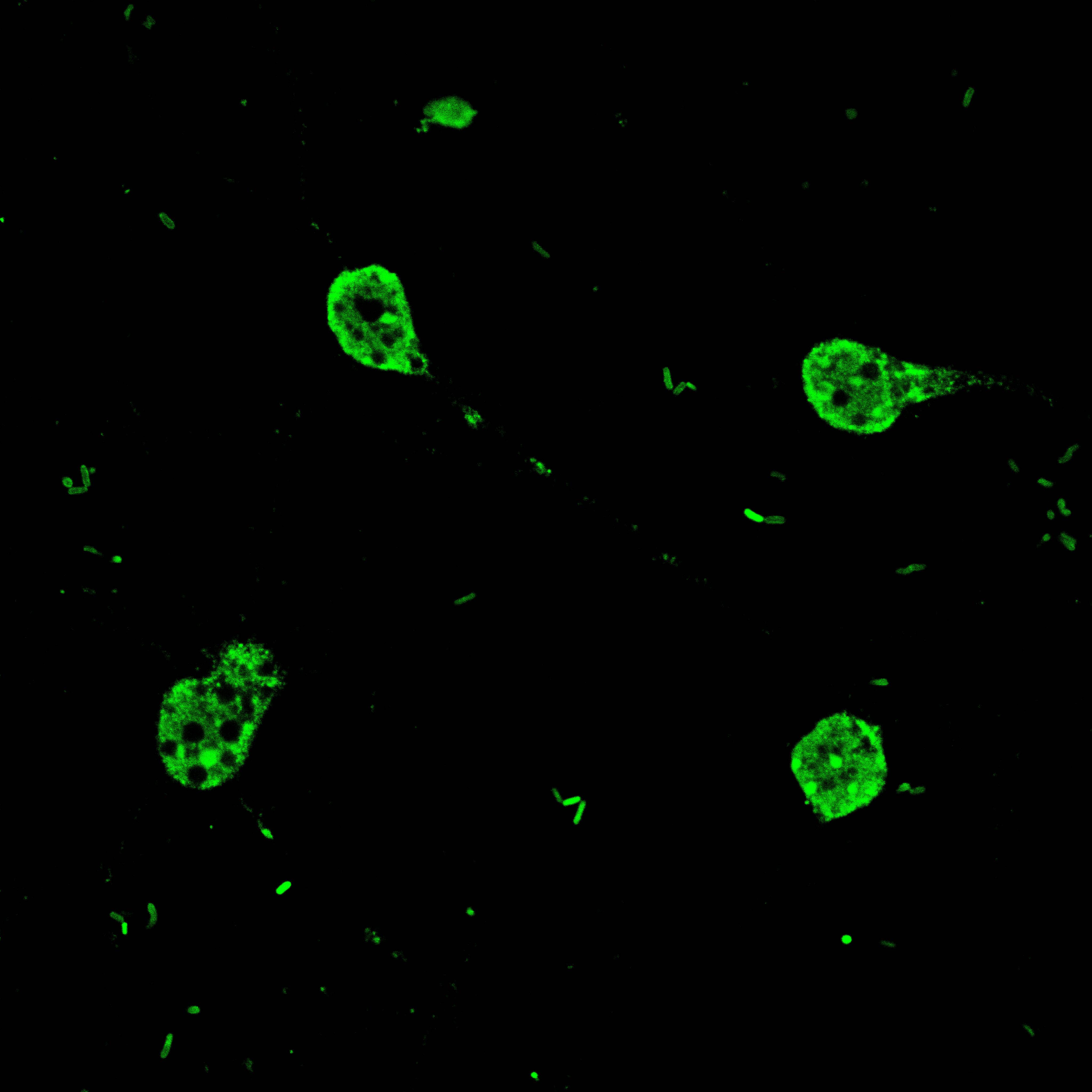

Supplement: Supplementary file 10 — Appendix Figure Source Data [file 44321_2025_206_MOESM10_ESM.zip › Appendix Figures Source Data/Appendix Fig. S7/OTUB2.tif]

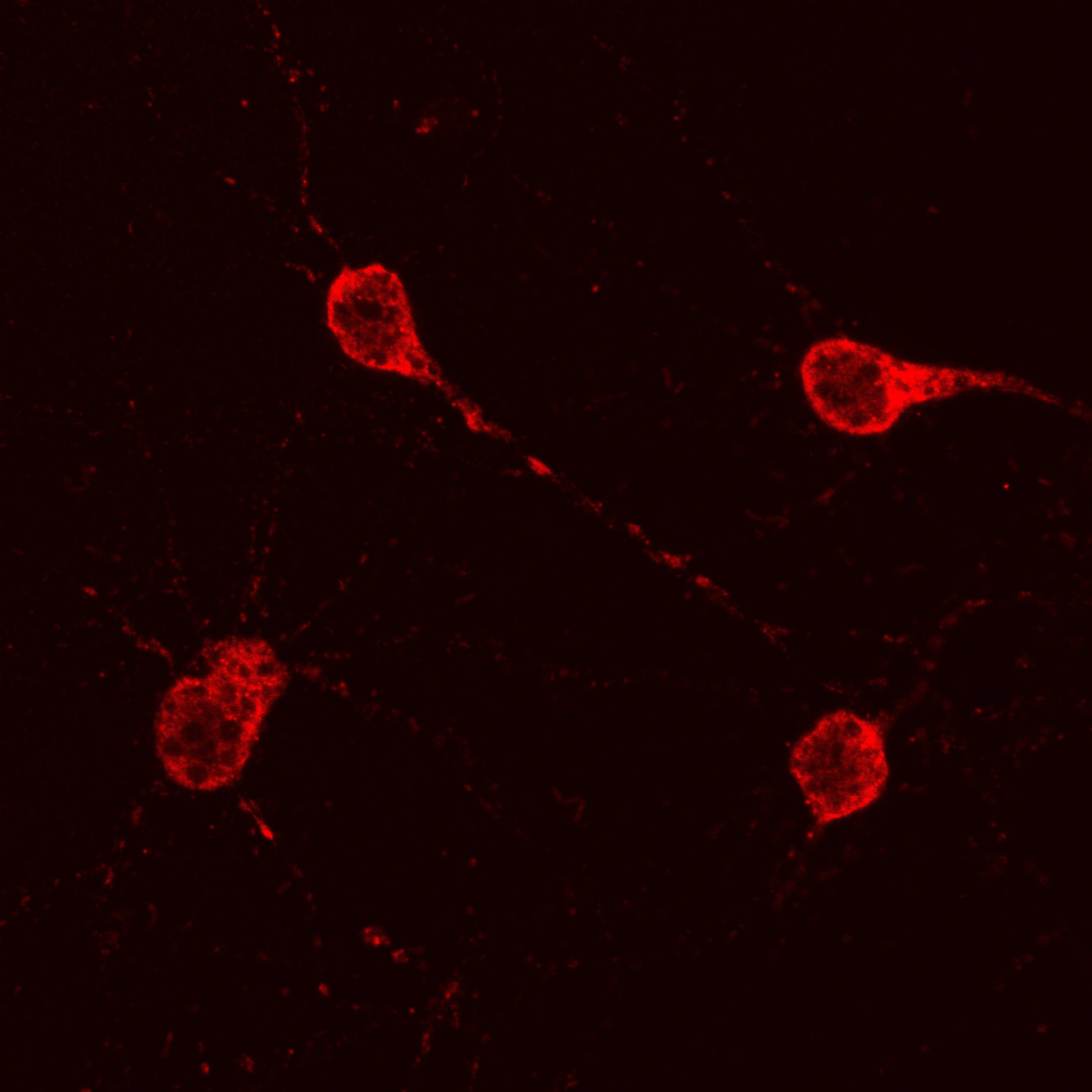

Supplement: Supplementary file 10 — Appendix Figure Source Data [file 44321_2025_206_MOESM10_ESM.zip › Appendix Figures Source Data/Appendix Fig. S7/RIPK3.tif]

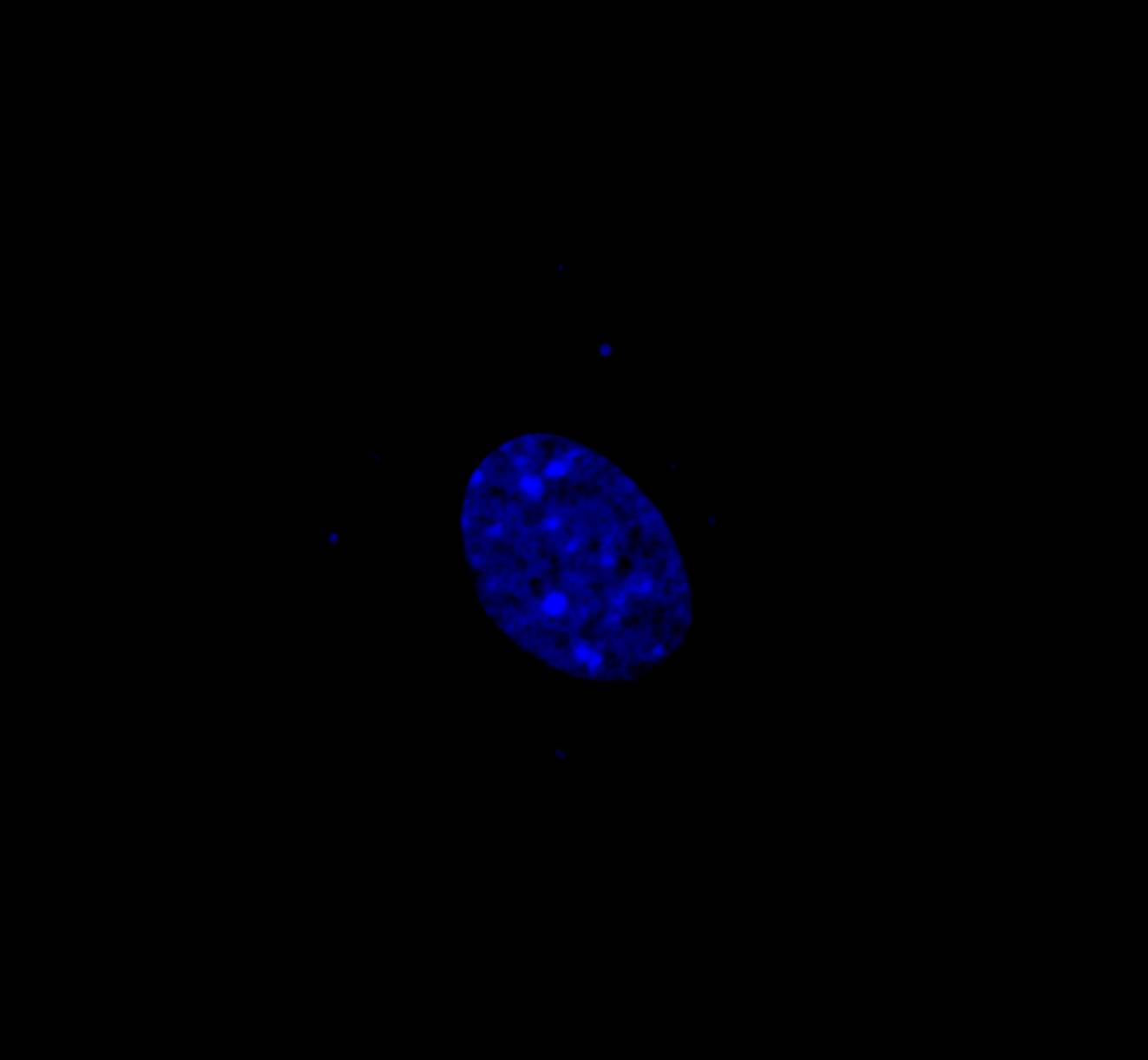

Supplement: Supplementary file 10 — Appendix Figure Source Data [file 44321_2025_206_MOESM10_ESM.zip › Appendix Figures Source Data/Appendix Fig. S9/KO Lamp DAPI.tif]

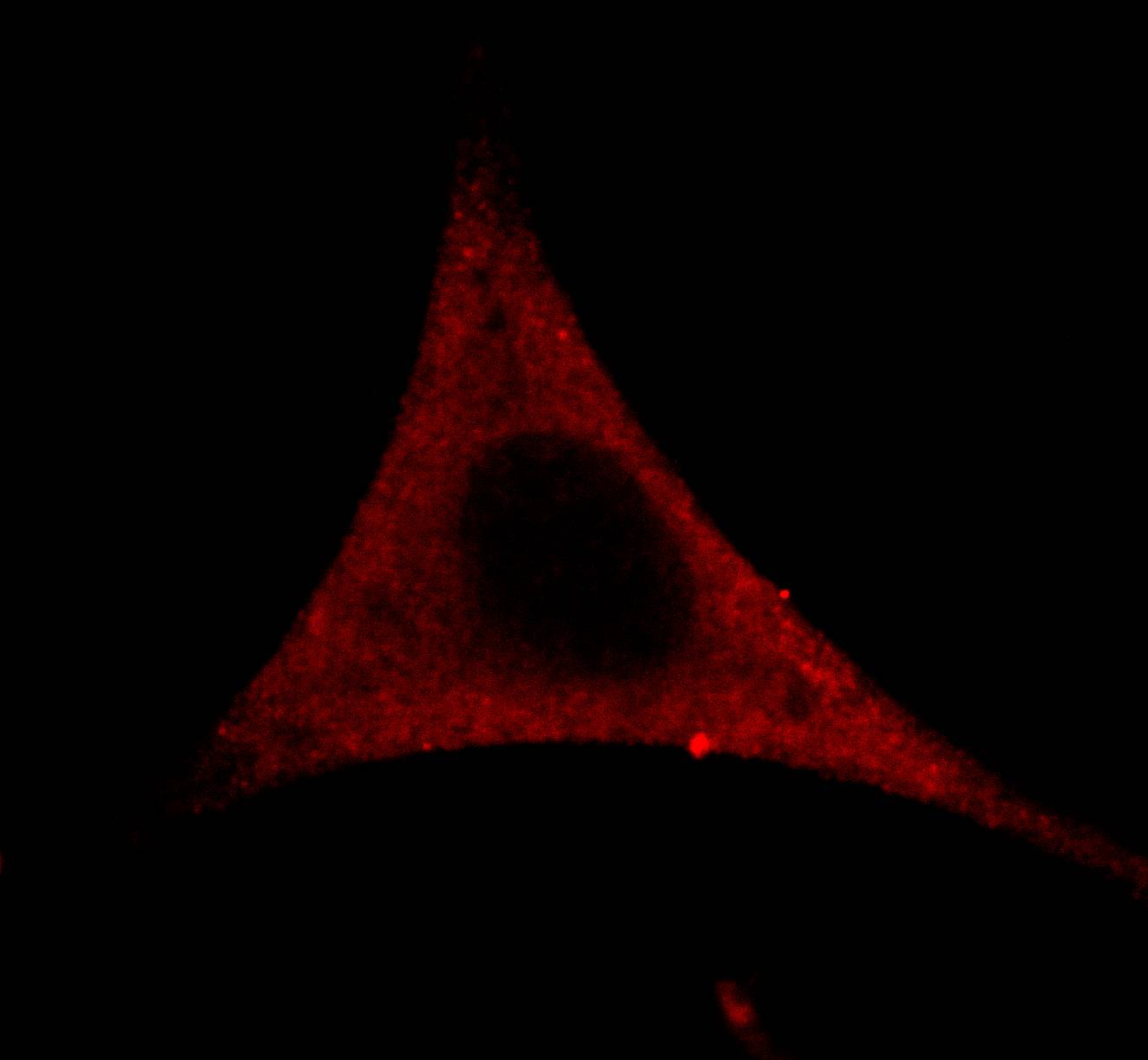

Supplement: Supplementary file 10 — Appendix Figure Source Data [file 44321_2025_206_MOESM10_ESM.zip › Appendix Figures Source Data/Appendix Fig. S9/KO Lamp Lamp1.tif]

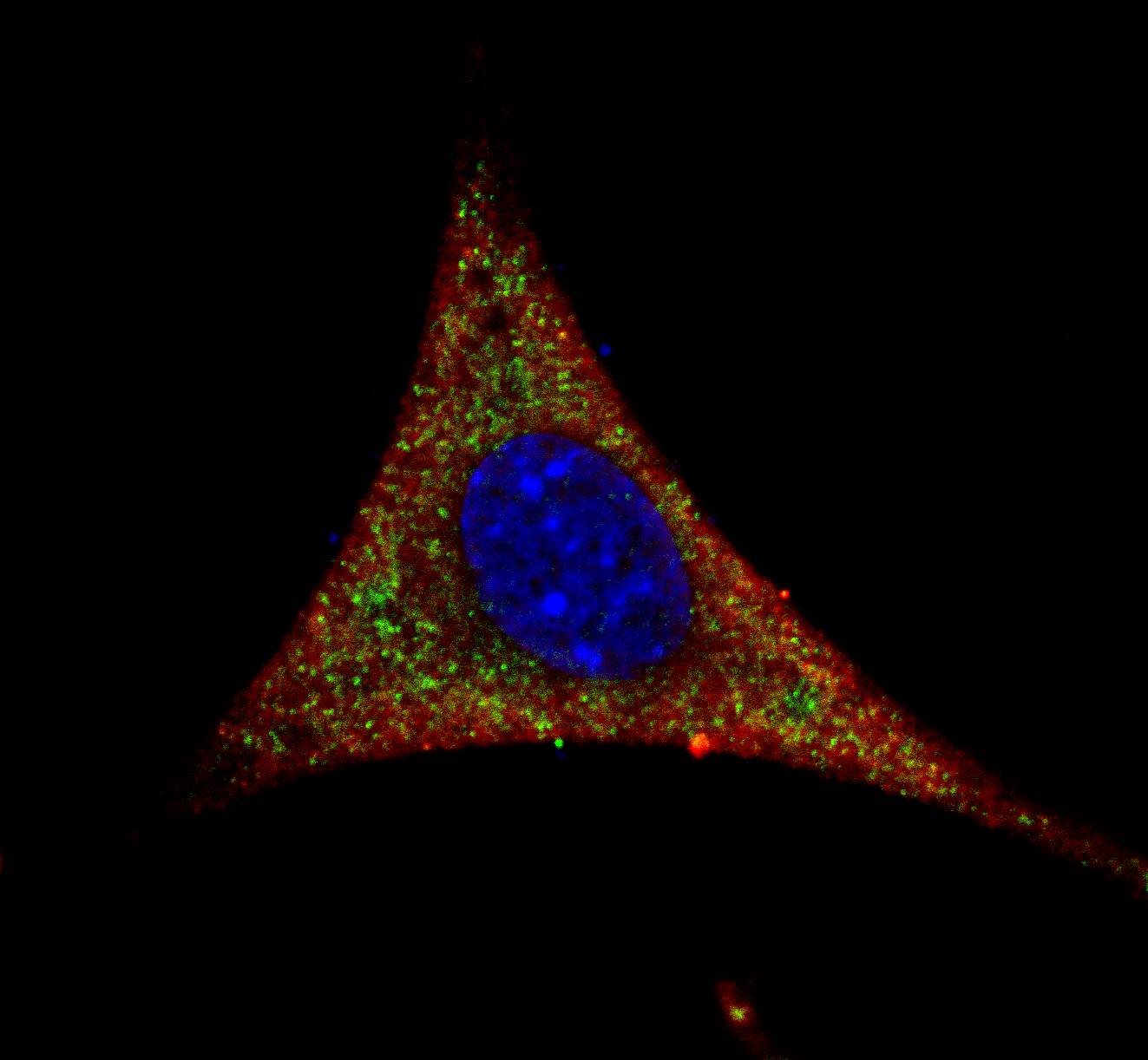

Supplement: Supplementary file 10 — Appendix Figure Source Data [file 44321_2025_206_MOESM10_ESM.zip › Appendix Figures Source Data/Appendix Fig. S9/KO Lamp Merge.tif]

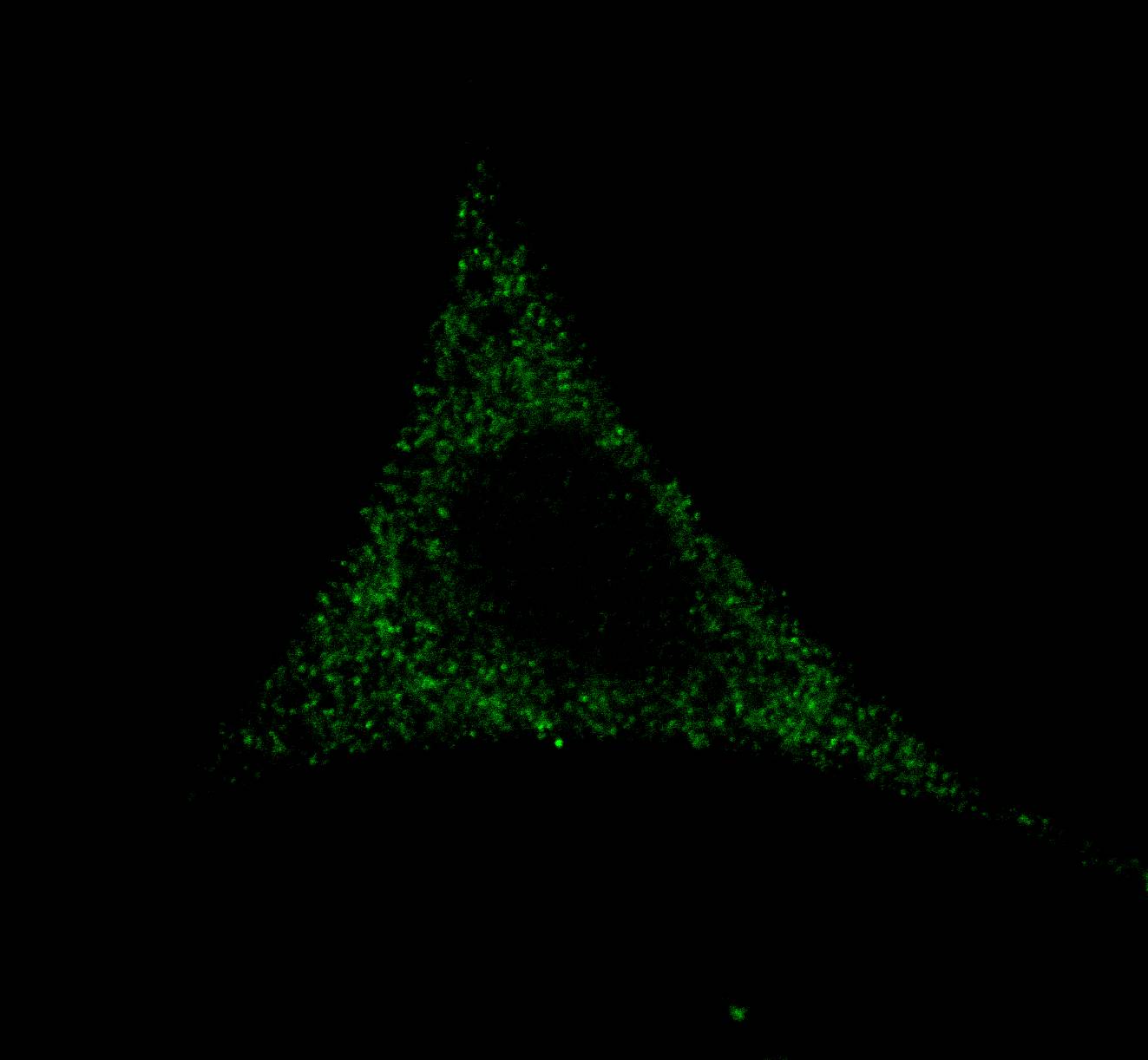

Supplement: Supplementary file 10 — Appendix Figure Source Data [file 44321_2025_206_MOESM10_ESM.zip › Appendix Figures Source Data/Appendix Fig. S9/KO Lamp RIPK3.tif]

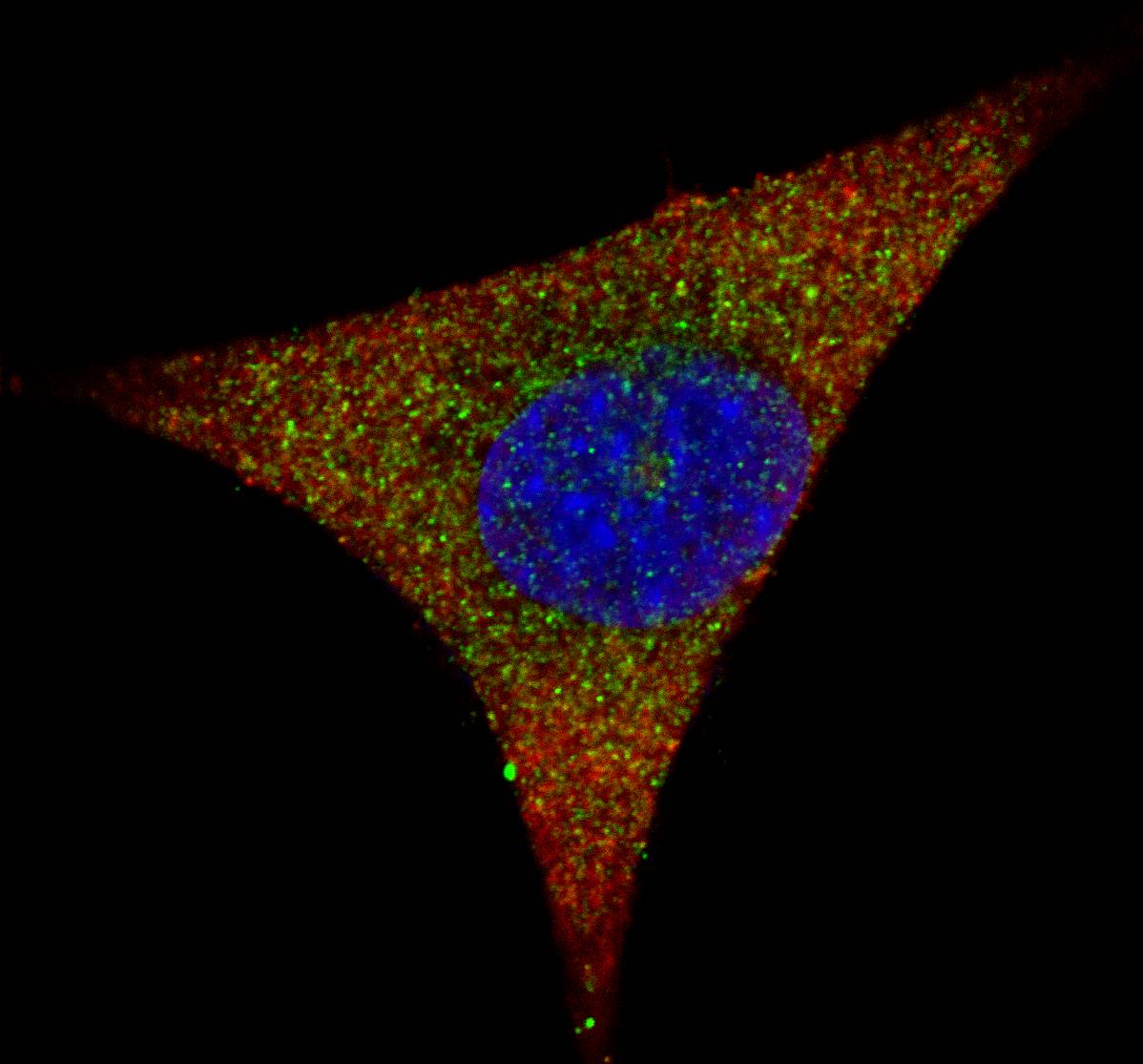

Supplement: Supplementary file 10 — Appendix Figure Source Data [file 44321_2025_206_MOESM10_ESM.zip › Appendix Figures Source Data/Appendix Fig. S9/WT Lamp Merge.tif]

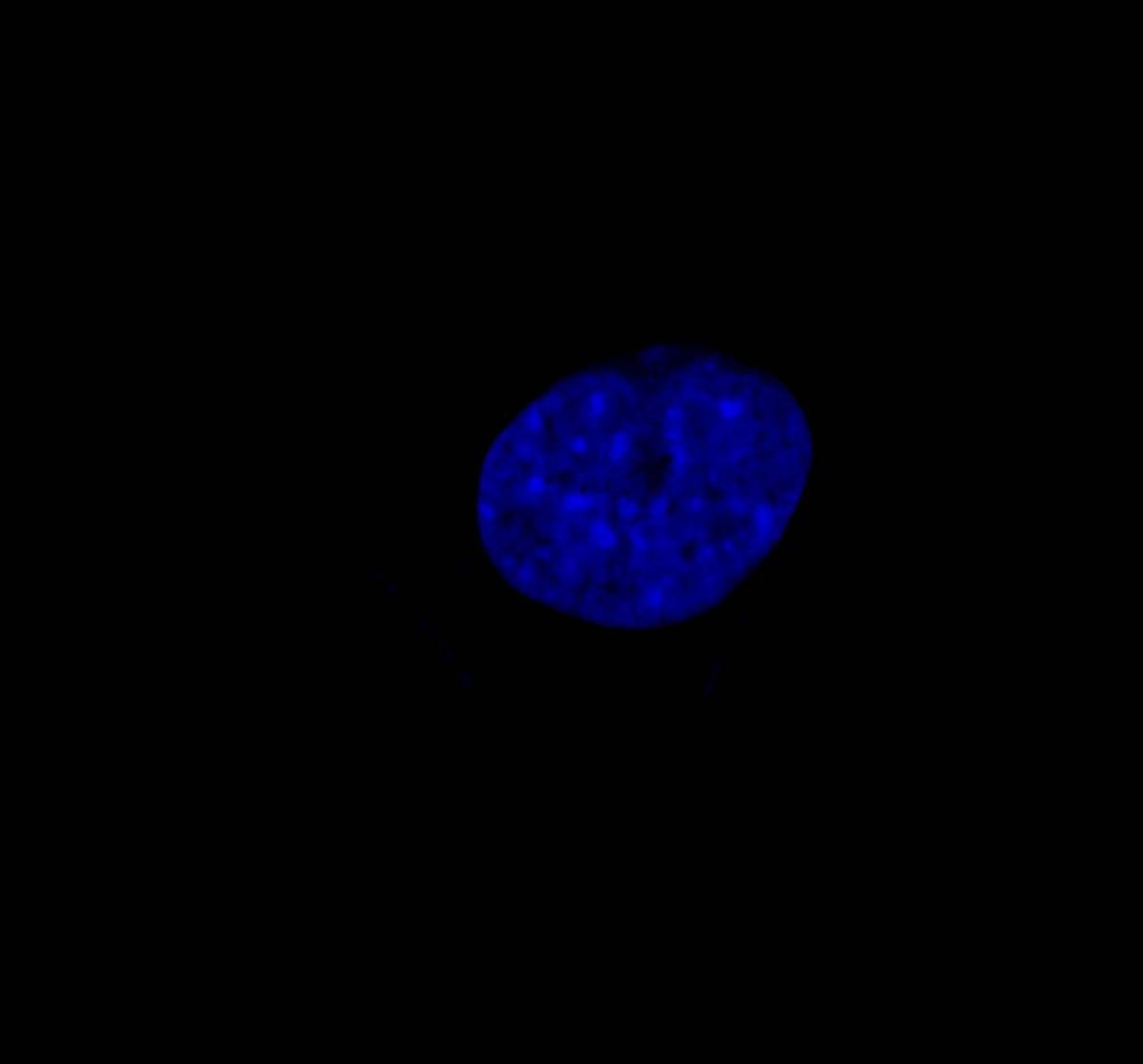

Supplement: Supplementary file 10 — Appendix Figure Source Data [file 44321_2025_206_MOESM10_ESM.zip › Appendix Figures Source Data/Appendix Fig. S9/WT Lamp DAPI.tif]

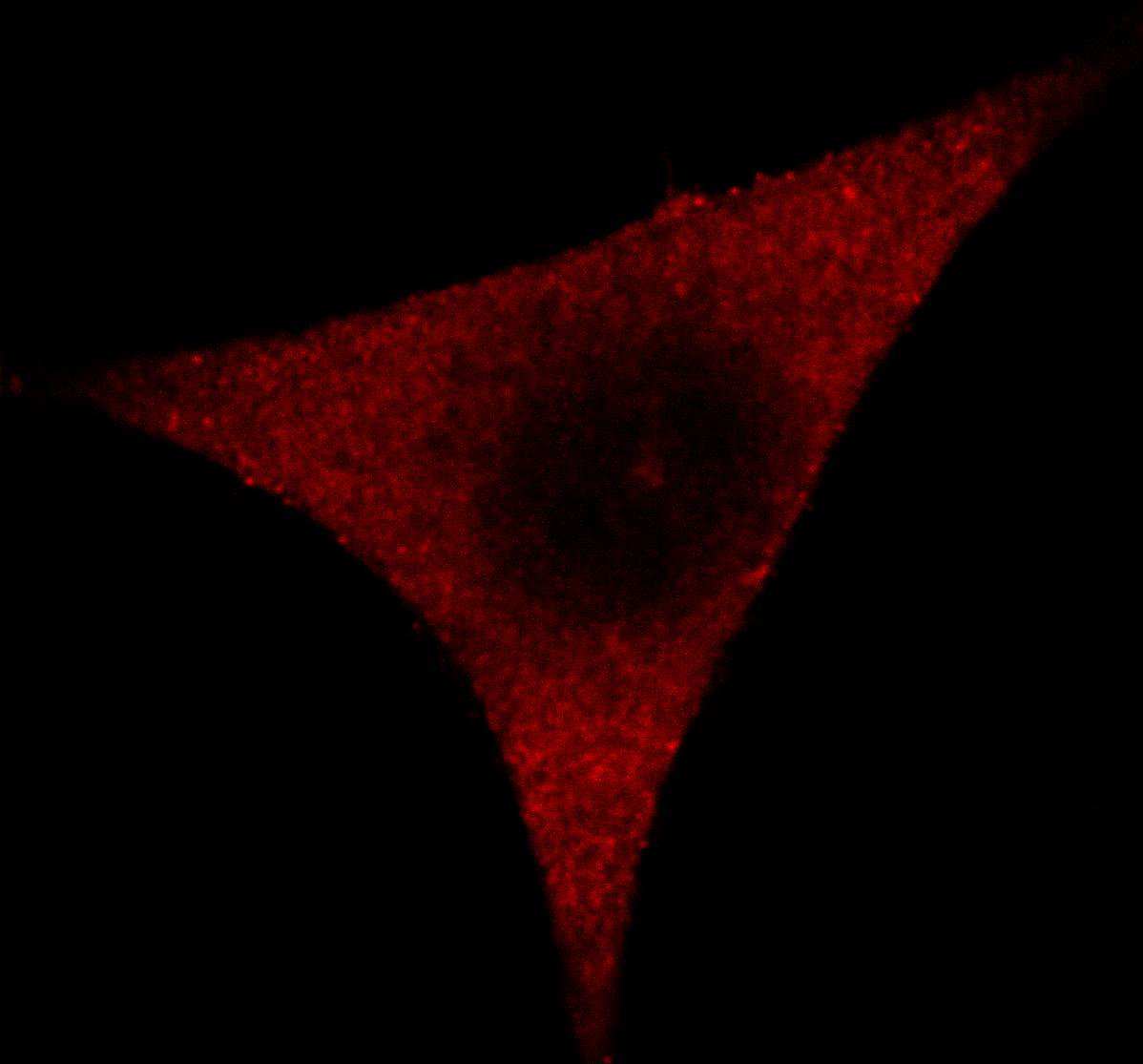

Supplement: Supplementary file 10 — Appendix Figure Source Data [file 44321_2025_206_MOESM10_ESM.zip › Appendix Figures Source Data/Appendix Fig. S9/WT Lamp Lamp1.tif]

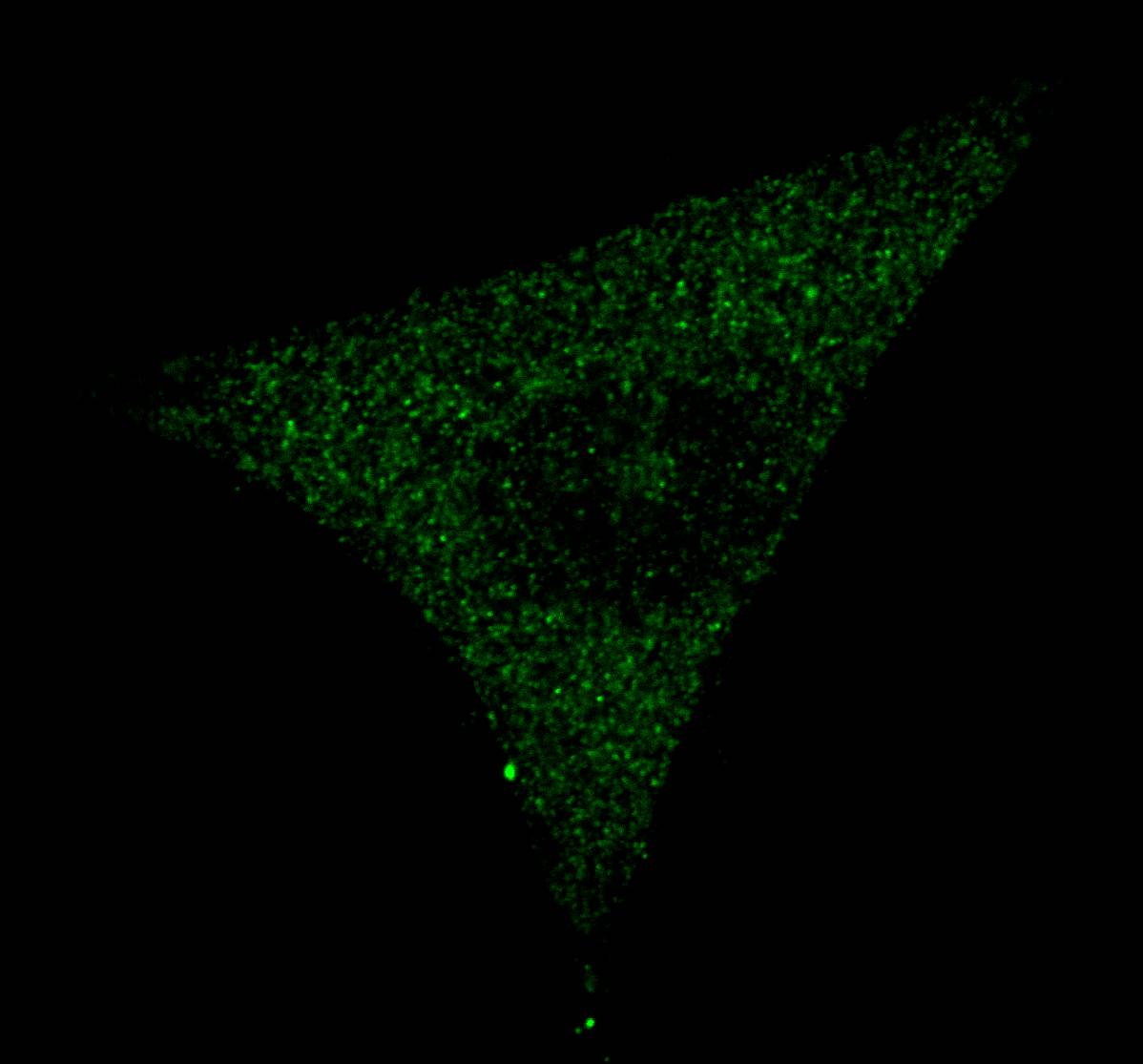

Supplement: Supplementary file 10 — Appendix Figure Source Data [file 44321_2025_206_MOESM10_ESM.zip › Appendix Figures Source Data/Appendix Fig. S9/WT Lamp RIPK3.tif]
